# The 2024 report of the *Lancet* Countdown on health and climate change: facing record-breaking threats from delayed action

**DOI:** 10.1016/S0140-6736(24)01822-1

**Published:** 2024-10-30

**Authors:** Marina Romanello, Maria Walawender, Shih-Che Hsu, Annalyse Moskeland, Yasna Palmeiro-Silva, Daniel Scamman, Zakari Ali, Nadia Ameli, Denitsa Angelova, Sonja Ayeb-Karlsson, Sara Basart, Jessica Beagley, Paul J Beggs, Luciana Blanco-Villafuerte, Wenjia Cai, Max Callaghan, Diarmid Campbell-Lendrum, Jonathan D Chambers, Victoria Chicmana-Zapata, Lingzhi Chu, Troy J Cross, Kim R van Daalen, Carole Dalin, Niheer Dasandi, Shouro Dasgupta, Michael Davies, Robert Dubrow, Matthew J Eckelman, James D Ford, Chris Freyberg, Olga Gasparyan, Georgiana Gordon-Strachan, Michael Grubb, Samuel H Gunther, Ian Hamilton, Yun Hang, Risto Hänninen, Stella Hartinger, Kehan He, Julian Heidecke, Jeremy J Hess, Louis Jamart, Slava Jankin, Harshavardhan Jatkar, Ollie Jay, Ilan Kelman, Harry Kennard, Gregor Kiesewetter, Patrick Kinney, Dominic Kniveton, Rostislav Kouznetsov, Pete Lampard, Jason KW Lee, Bruno Lemke, Bo Li, Yang Liu, Zhao Liu, Alba Llabrés-Brustenga, Melissa Lott, Rachel Lowe, Jaime Martinez-Urtaza, Mark Maslin, Lucy McAllister, Celia McMichael, Zhifu Mi, James Milner, Kelton Minor, Jan Minx, Nahid Mohajeri, Natalie C Momen, Maziar Moradi-Lakeh, Karyn Morrisey, Simon Munzert, Kris A Murray, Nick Obradovich, Megan B O’Hare, Camile Oliveira, Tadj Oreszczyn, Matthias Otto, Fereidoon Owfi, Olivia L Pearman, Frank Pega, Andrew J Perishing, Ana-Catarina Pinho-Gomes, Jamie Ponmattam, Mahnaz Rabbaniha, Jamie Rickman, Elizabeth Robinson, Joacim Rocklöv, David Rojas-Rueda, Renee N Salas, Jan C Semenza, Jodi D Sherman, Joy Shumake-Guillemot, Pratik Singh, Henrik Sjödin, Jessica Slater, Mikhail Sofiev, Cecilia Sorensen, Marco Springmann, Zélie Stalhandske, Jennifer D Stowell, Meisam Tabatabaei, Jonathon Taylor, Daniel Tong, Cathryn Tonne, Marina Treskova, Joaquin A Trinanes, Andreas Uppstu, Fabian Wagner, Laura Warnecke, Hannah Whitcombe, Peng Xian, Carol Zavaleta-Cortijo, Chi Zhang, Ran Zhang, Shihui Zhang, Ying Zhang, Qiao Zhu, Peng Gong, Hugh Montgomery, Anthony Costello

**Affiliations:** Institute for Global Health University College London, London, UK; Energy Institute, University College London, London, UK; Department of Geography and Environment, London School of Economics and Political Science, London, UK; Institute for Global Health, University College London, London, UK; Institute for Sustainable Resources, University College London, London, UK; Medical Research Council Unit, The Gambia, London School of Hygiene & Tropical Medicine, Serekunda, The Gambia; Institute for Sustainable Resources, University College London, London, UK; Department of Risk and Disaster Reduction, University College London, London, UK; World Metereological Organization, Geneva, Switzerland; Global Climate and Health Alliance, Richmond, CA, USA; School of Natural Sciences, Faculty of Science and Engineering, Macquarie University, Sydney, NSW, Australia; Centro Latino Americano de Excelencia en Cambio Climático y Salud, Universidad Peruana Cayetano Heredia, Lima, Peru; Department of Earth System Science, Tsinghua University, Beijing, China; Mercator Research Institute on Global Commons and Climate Change, Berlin, Germany; Department of Environment, Climate Change and Health, WHO, Geneva, Switzerland; University of Geneva, Geneva, Switzerland; Intercultural Citizenship and Indigenous Health Unit, Universidad Peruana Cayetano Heredia, Lima, Peru; Yale Center on Climate Change and Health, Yale University, New Haven, CT, USA; Heat and Health Research Centre, University of Sydney, Sydney, NSW, Australia; Barcelona Supercomputing Center, Barcelona, Spain; Institute for Sustainable Resources, University College London, London, UK; School of Government, University of Birmingham, Birmingham, UK; Euro-Mediterranean Center on Climate Change Foundation, Lecce, Italy; Institute for Environmental Design and Engineering, University College London, London, UK; Yale Center on Climate Change and Health, Yale University, New Haven, CT, USA; Department of Civil & Environmental Engineering, Northeastern University, Boston, MA, USA; Priestley Centre for Climate Futures, University of Leeds, Leeds, UK; Ruby Coast Research Group, Ruby Bay, New Zealand; Department of Political Science, Florida State University, Tallahassee, FL,USA; Tropical Metabolism Research Unit, Caribbean Institute for Health Research, University of the West Indies, Kingston, Jamaica; Institute for Sustainable Resources, University College London, London, UK; Yong Loo Lin School of Medicine, National University of Singapore, Singapore; Energy Institute, University College London, London, UK; Department of Environmental and Occupational Health Sciences, University of Texas Health Science Center at Houston, Houston, TX, USA; Finnish Meteorological Institute, Helsinki, Finland; School of Public Health and Administration, Universidad Peruana Cayetano Heredia, Lima, Peru; Institute for Climate and Carbon Neutrality, University of Hong Kong, Hong Kong Special Administrative Region, China; Interdisciplinary Centre for Scientific Computing, Heidelberg University, Heidelberg, Germany; Centre for Health and the Global Environment, University of Washington, Seattle, WA, USA; Institute for Global Health, University College London, London, UK; School of Government, University of Birmingham, Birmingham, UK; Energy Institute, University College London, London, UK; Heat and Health Research Centre, University of Sydney, Sydney, NSW, Australia; Institute for Global Health, University College London, London, UK; Center on Global Energy Policy, Columbia University, New York, NY, USA; Pollution Management Group, Program on Energy, Climate and the Environment, International Institute for Applied Systems Analysis, Laxenburg, Austria; Department of Environmental Health, Boston University, Boston, MA, USA; School of Global Studies, University of Sussex, Falmer, UK; Finnish Meteorological Institute, Helsinki, Finland; Department of Health Sciences, University of York, York, UK; Yong Loo Lin School of Medicine, National University of Singapore, Singapore; Nelson Marlborough Institute of Technology–Te Pukenga, Nelson, New Zealand; School of Management, Beijing Institute of Technology, Beijing, China; Emory University, Atlanta, GA, USA; Department of Earth System Science, Tsinghua University, Beijing, China; Barcelona Supercomputing Center, Barcelona, Spain; Center on Global Energy Policy, Columbia University, New York, NY, USA; Catalan Institution for Research and Advanced Studies, Barcelona, Spain; Department of Genetics and Microbiology, School of Biosciences, Universitat Autònoma de Barcelona, Barcelona, Spain; Department of Geography, University College London, London, UK; Environmental Studies Program, Denison University, Granville, OH, USA; School of Geography, Earth and Atmospheric Sciences, University of Melbourne, Melbourne, VIC, Australia; Bartlett School of Sustainable Construction, University College London, London, UK; Department of Public Health, Environments, and Society, London School of Hygiene and Tropical Medicine, London, UK; Data Science Institute, Columbia University, New York, NY, USA; Mercator Research Institute on Global Commons and Climate Change, Berlin, Germany; Institute for Environmental Design and Engineering, University College London, London, UK; Department of Environment, Climate Change and Health, WHO, Geneva, Switzerland; Gastrointestinal and Liver Diseases Research Center, Iran University of Medical Sciences, Tehran, Iran; Department of Technology, Management and Economics, Technical University of Denmark, Copenhagen, Denmark; Data Science Lab, Hertie School, Berlin, Germany; Medical Research Council Unit, The Gambia, London School of Hygiene & Tropical Medicine, Serekunda, The Gambia; Laureate Institute for Brain Research, Massachusetts Institute of Technology, Tulsa, OK, USA; Institute for Global Health, University College London, London, UK; Energy Institute, University College London, London, UK; Nelson Marlborough Institute of Technology– Te Pukenga, Nelson, New Zealand; Agricultural Research, Education and Extension Organization, Iranian Fisheries Science Research Institute, Tehran, Iran; Social and Economic Analysis Branch, US Geological Survey, Fort Collins, OH, USA; Department of Environment, Climate Change and Health, WHO, Geneva, Switzerland; Climate Central, Princeton, NJ, USA; Institute for Global Health, University College London, London, UK; Global Health and Population, Harvard TH Chan School of Public Health, Boston, MA, USA; Agricultural Research, Education, and Extension Organisation, Iranian Fisheries Science Research Institute, Tehran, Iran; Institute for Sustainable Resources, University College London, London, UK; Grantham Research Institute, London School of Economics, London, UK; Interdisciplinary Centre for Scientific Computing, Heidelberg University, Heidelberg, Germany; Department of Environmental and Radiological Health Sciences, Colorado State University, Fort Collins, CO, USA; Harvard Medical School, Harvard University, Boston, MA, USA; Department of Public Health and Clinical Medicine, Section of Sustainable Health, Umeå University, Umeå, Sweden; Department of Anesthesiology, Yale School of Medicine, Yale University, New Haven, CT, USA; WHO-WMO Joint Climate and Health Office, Geneva, Switzerland; Interdisciplinary Centre for Scientific Computing, Heidelberg University, Heidelberg, Germany; Department of Public Health and Clinical Medicine, Section of Sustainable Health, Umeå University, Umeå, Sweden; Pollution Management Group, Program on Energy, Climate and the Environment, International Institute for Applied Systems Analysis, Laxenburg, Austria; Finnish Meteorological Institute, Helsinki, Finland; Data Science Institute, Columbia University, New York, NY, USA; Mailman School of Public Health, Columbia University, New York, NY, USA; Nuffield Department of Population Health, University of Oxford, Oxford, UK; ETH Zürich, Zürich, Switzerland; Department of Environmental Health, School of Public Health, Boston University, Boston, MA, USA; Higher Institution Centre of Excellence, Institute of Tropical Aquaculture and Fisheries, Universiti Malaysia Terengganu, Terengganu, Malaysia; Department of Civil Engineering, Tampere University, Tampere, Finland; George Mason University, Fairfax, VA, USA; Barcelona Institute for Global Health, Barcelona, Spain; Heidelberg Institute of Global Health, Heidelberg University, Heidelberg, Germany; Department of Electronics and Computer Sciences, Universidade de Santiago de Compostela, Santiago de Compostela, Spain; Finnish Meteorological Institute, Helsinki, Finland; Pollution Management Group, Program on Energy, Climate and the Environment, International Institute for Applied Systems Analysis, Laxenburg, Austria; Institute for Global Health, University College London, London, UK; United States Navy Research Laboratory, Monterey, CA, USA; Intercultural Citizenship and Indigenous Health Unit, Universidad Peruana Cayetano Heredia, Lima, Peru; School of Management, Beijing Institute of Technology, Beijing, China; Natural Language Learning Group, University of Mannheim, Mannheim, Germany; Department of Earth System Science, Tsinghua University, Beijing, China; University of Sydney, Sydney, NSW, Australia; Emory University, Atlanta, GA, USA; Institute for Climate and Carbon Neutrality, University of Hong Kong, Hong Kong Special Administrative Region, China; Department of Geography University of Hong Kong, Hong Kong Special Administrative Region, China; Bartlett School of Sustainable Construction, University College London, London, UK; Centre for Human Health and Performane, University College London, London, UK; Institute for Global Health, University College London, London, UK

## Abstract

Despite the initial hope inspired by the 2015 Paris Agreement, the world is now dangerously close to breaching its target of limiting global multiyear mean heating to 1·5°C. Annual mean surface temperature reached a record high of 1·45°C above the pre-industrial baseline in 2023, and new temperature highs were recorded throughout 2024. The resulting climatic extremes are increasingly claiming lives and livelihoods worldwide.

The *Lancet* Countdown: tracking progress on health and climate change was established the same year the Paris Agreement entered into force, to monitor the health impacts and opportunities of the world’s response to this landmark agreement. Supported through strategic core funding from Wellcome, the collaboration brings together over 300 multidisciplinary researchers and health professionals from around the world to take stock annually of the evolving links between health and climate change at global, regional, and national levels.

The 2024 report of the *Lancet* Countdown, building on the expertise of 122 leading researchers from UN agencies and academic institutions worldwide, reveals the most concerning findings yet in the collaboration’s 8 years of monitoring.

**The record-breaking human costs of climate change:**

Data in this year’s report show that people all around the world are facing record-breaking threats to their wellbeing, health, and survival from the rapidly changing climate. Of the 15 indicators monitoring climate change-related health hazards, exposures, and impacts, ten reached concerning new records in their most recent year of data.

Heat-related mortality of people older than 65 years increased by a record-breaking 167%, compared with the 1990s, 102 percentage points higher than the 65% that would have been expected without temperature rise (indicator 1.1.5). Heat exposure is also increasingly affecting physical activity and sleep quality, in turn affecting physical and mental health. In 2023, heat exposure put people engaging in outdoor physical activity at risk of heat stress (moderate or higher) for a record high of 27·7% more hours than on average in the 1990s (indicator 1.1.2) and led to a record 6% more hours of sleep lost in 2023 than the average during 1986–2005 (indicator 1.1.4).

People worldwide are also increasingly at risk from life-threatening extreme weather events. Between 1961–90 and 2014–23, 61% of the global land area saw an increase in the number of days of extreme precipitation (indicator 1.2.3), which in turn increases the risk of flooding, infectious disease spread, and water contamination. In parallel, 48% of the global land area was affected by at least 1 month of extreme drought in 2023, the second largest affected area since 1951 (indicator 1.2.2). The increase in drought and heatwave events since 1981–2010 was, in turn, associated with 151 million more people experiencing moderate or severe food insecurity across 124 countries assessed in 2022, the highest recorded value (indicator 1.4.2).

The hotter and drier weather conditions are increasingly favouring the occurrence of sand and dust storms. This weather-environmental phenomenon contributed to a 31% increase in the number of people exposed to dangerously high particulate matter concentrations between 2003–07 and 2018–22 (indicator 1.2.4). Meanwhile, changing precipitation patterns and rising temperatures are favouring the transmission of deadly infectious diseases such as dengue, malaria, West Nile virus-related illness, and vibriosis, putting people at risk of transmission in previously unaffected locations (indicators 1.3.1–1.3.4).

Compounding these impacts, climate change is affecting the social and economic conditions on which health and wellbeing depend. The average annual economic losses from weather-related extreme events increased by 23% from 2010–14 to 2019–23, to US$227 billion (a value exceeding the gross domestic product [GDP] of about 60% of the world’s economies; indicator 4.1.1). Although 60·5% of losses in very high Human Development Index (HDI) countries were covered by insurance, the vast majority of those in countries with lower HDI levels were uninsured, with local communities bearing the brunt of the physical and economic losses (indicator 4.1.1). Extreme weather and climate change-related health impacts are also affecting labour productivity, with heat exposure leading to a record high loss of 512 billion potential labour hours in 2023, worth $835 billion in potential income losses (indicators 1.1.3 and 4.1.3). Low and medium HDI countries were most affected by these losses, which amounted to 7·6% and 4·4% of their GDP, respectively (indicator 4.1.3). With the most underserved communities most affected, these economic impacts further reduce their capacity to cope with and recover from the growing impacts of climate change, thereby amplifying global inequities.

Concerningly, multiple hazards revealed by individual indicators are likely to have simultaneous compounding and cascading impacts on the complex and inter-connected human systems that sustain good health, disproportionately threatening people’s health and survival with every fraction of a degree of increase in global mean temperature.

Despite years of monitoring exposing the imminent health threats of climate inaction, the health risks people face have been exacerbated by years of delays in adaptation, which have left people ill-protected from the growing threats of climate change. Only 68% of countries reported high-to-very-high implementation of legally mandated health emergency management capacities in 2023, of which just 11% were low HDI countries (indicator 2.2.5). Moreover, only 35% of countries reported having health early warning systems for heat-related illness, whereas 10% did so for mental and psychosocial conditions (indicator 2.2.1). Scarcity of financial resources was identified as a key barrier to adaptation, including by 50% of the cities that reported they were not planning to undertake climate change and health risk assessments (indicator 2.1.3). Indeed, adaptation projects with potential health benefits represented just 27% of all the Green Climate Fund’s adaptation funding in 2023, despite a 137% increase since 2021 (indicator 2.2.4). With universal health coverage still unattained in most countries, financial support is needed to strengthen health systems and ensure that they can protect people from growing climate change-related health hazards. The unequal distribution of financial resources and technical capacity is leaving the most vulnerable populations further unprotected from the growing health risks.

**Fuelling the fire:**

As well as exposing the inadequacy of adaptation efforts to date, this year’s report reveals a world veering away from the goal of limiting temperature rise to 1·5°C, with concerning new records broken across indicators monitoring greenhouse gas emissions and the conditions that enable them.

Far from declining, global energy-related CO_2_ emissions reached an all-time high in 2023 (indicator 3.1.1). Oil and gas companies are reinforcing the global dependence on fossil fuels and—partly fuelled by the high energy prices and windfall profits of the global energy crisis—most are further expanding their fossil fuel production plans. As of March, 2024, the 114 largest oil and gas companies were on track to exceed emissions consistent with 1·5°C of heating by 189% in 2040, up from 173% 1 year before (indicator 4.2.2). As a result, their strategies are pushing the world further off track from meeting the goals of the Paris Agreement, further threatening people’s health and survival.

Although renewable energy could provide power to remote locations, its adoption is lagging, particularly in the most vulnerable countries. The consequences of this delay reflect the human impacts of an unjust transition. Globally, 745 million people still lack access to electricity and are facing the harms of energy poverty on health and wellbeing. The burning of polluting biomass (eg, wood or dung) still accounts for 92% of the energy used in the home by people in low HDI countries (indicator 3.1.2), and only 2·3% of electricity in these countries comes from clean renewables, compared with 11·6% in very high HDI countries (indicators 3.1.1). This persistent burning of fossil fuel and biomass led to at least 3·33 million deaths from outdoor fine particulate matter (PM_2·5_) air pollution globally in 2021 alone (indicator 3.2.1), and the domestic use of dirty solid fuels caused 2·3 million deaths from indoor air pollution in 2020 across 65 countries analysed (indicator 3.2.2).

Compounding the growth in energy-related greenhouse gas emissions, almost 182 million hectares of forests were lost between 2016 and 2022 (indicator 3.4), reducing the world’s natural capacity to capture atmospheric CO_2_. In parallel, the consumption of red meat and dairy products, which contributed to 11·2 million deaths attributable to unhealthy diets in 2021 (indicator 3.3.2), has led to a 2·9% increase in agricultural greenhouse gas emissions since 2016 (indicator 3.3.1).

Health systems themselves, although essential to protect people’s health, are also increasingly contributing to the problem. Greenhouse gas emissions from health care have increased by 36% since 2016, making health systems increasingly unprepared to operate in a net zero emissions future and pushing health care further from its guiding principle of doing no harm (indicator 3.5).

The growing accumulation of greenhouse gases in the atmosphere is pushing the world to a future of increasingly dangerous health hazards and reducing the chances of survival of vulnerable people all around the globe.

**Health-threatening financial flows:**

With the availability of financial resources a key barrier to tackling climate change, a rapid growth in predictable and equitable investment is urgently needed to avoid the most dangerous impacts of climate change. A growing body of literature shows that the economic benefits of a transition to net zero greenhouse gas emissions will far exceed the costs of inaction. Healthier, more resilient populations will further support more prosperous and sustainable economies (indicators 4.1.2–4.1.4).

However, although funding to enable potentially life-saving climate change adaptation and mitigation activities remains scarce, substantial financial resources are being allocated to activities that harm health and perpetuate a fossil fuel-based economy. The resulting reliance on fossil fuel energy has meant many countries faced sharp increases in energy prices following Russia’s invasion of Ukraine and the resulting disruption of fossil fuel supplies. To keep energy affordable to local populations, many governments resorted to increasing their explicit fossil fuel subsidies. Consequently, 84% of countries studied still operated net negative carbon prices (explicit net fossil fuel subsidies) in 2022, for a record high net total of $1·4 trillion (indicator 4.3.3), with the sums involved often comparable to countries’ total health budgets. In addition, although clean energy investment grew by 10% globally in 2023—exceeding fossil fuel investment by 73%—considerable regional disparities exist. Clean energy investment is 38% lower than fossil fuel spending in emerging market and developing economies outside China. Clean energy spending in these countries only accounted for 17·4% of the global total. Moreover, investment in energy efficiency and end use, essential for a just transition, decreased by 1·3% in 2023 (indicator 4.3.1).

The resulting expansion of fossil fuel assets is increasingly jeopardising the economies on which people’s livelihoods depend. On the current trajectory, the world already faces potential global income losses ranging from 11% to 29% by 2050. The number of fossil fuel industry employees reached 11·8 million in 2022, increasing the size of a workforce whose employment cannot be sustained in a world that avoids the most catastrophic human impacts of climate change (indicator 4.2.1). Meanwhile, ongoing investments in coal power have pushed the value of coal-fired power generation assets that risk becoming stranded within 10 years (between 2025 and 2034) in a 1·5°C trajectory to a cumulative total of $164·5 billion—a value that will increase if coal investments persist (indicator 4.2.3). The prioritisation of fossil fuel-based systems means most countries remain ill-prepared for the vital transition to zero greenhouse gas emission economies. As a result of an unjust transition, the risk is unequally distributed: preparedness scores for the transition to a net zero greenhouse gas economy were below the global average in all countries with a low HDI, 96% of those with a medium HDI, and 84% of those with a high HDI, compared with just 7% of very high HDI countries (indicator 4.2.4).

**Defining the health profile of people worldwide:**

Following decades of delays in climate change action, avoiding the most severe health impacts of climate change now requires aligned, structural, and sustained changes across most human systems, including energy, transportation, agriculture, food, and health care. Importantly, a global transformation of financial systems is required, shifting resources away from the fossil fuel-based economy towards a zero emissions future. Putting people’s health at the centre of climate change policy making is key to ensuring this transition protects wellbeing, reduces health inequities, and maximises health gains. Some indicators reveal incipient progress and important opportunities for delivering this health-centred transformation.

As of December, 2023, 50 countries reported having formally assessed their health vulnerabilities and adaptation needs, up from 11 the previous year, and the number of countries that reported having a Health National Adaptation Plan increased from four in 2022 to 43 in 2023 (indicators 2.1.1 and 2.1.2). Additionally, 70% of 279 public health education institutions worldwide reported providing education in climate and health in 2023, essential to build capacities for health professionals to help shape this transition (indicator 2.2.6). Regarding the energy sector, the global share of electricity from clean modern renewables reached a record high of 10·5% in 2021 (indicator 3.1.1); clean energy investment exceeded fossil fuel investment by 73% in 2023 (indicator 4.3.1); and renewable energy-related employment has grown 35·6% since 2016, providing healthier and more sustainable employment opportunities than those in the fossil fuel industry (indicator 4.2.1). Importantly, mostly as a result of coal phase-down in high and very high HDI countries, deaths attributable to outdoor PM_2·5_ from fossil fuel combustion decreased by 6·9% between 2016 and 2021 (indicator 3.2.1), showing the life-saving potential of coal phase-out.

Important progress was made within international negotiations, which opened new opportunities to protect health in the face of climate change. After years of leadership from WHO on climate change and health, its Fourteenth General Programme of Work, adopted in May, 2024, made responding to climate change its first strategic priority. Within climate negotiations themselves, the 28th Conference of the Parties (COP28) of the United Nations Framework Convention on Climate Change (UNFCCC) featured the first health thematic day in 2023: 151 countries endorsed the COP28 United Arab Emirates Declaration on Climate and Health, and the Global Goal on Adaptation set a specific health target. The outcome of the first Global Stocktake of the Paris Agreement also recognised the right to health and a healthy environment, urging parties to take further health adaptation efforts, and opened a new opportunity for human survival, health, and wellbeing to be prioritised in the updated Nationally Determined Contributions (NDCs) due in 2025. The pending decision of how the Loss and Damage fund will be governed and the definition of the New Collective Quantified Goal on Climate Finance during COP29 provide further opportunities to secure the financial support crucial for a healthy net zero transition.

Although still insufficient to protect people’s health from climate change, these emerging signs of progress help open new opportunities to deliver a healthy, prosperous future. However, much remains to be done.

**Hanging in the balance:**

With climate change breaking dangerous new records and emissions persistently rising, preventing the most catastrophic consequences on human development, health, and survival now requires the support and will of all actors in society. However, data suggest that engagement with health and climate change could be declining across key sectors: the number of governments mentioning health and climate change in their annual UN General Debate statements fell from 50% in 2022 to 35% in 2023, and only 47% of the 58 NDCs updated as of February, 2024, referred to health (indicator 5.4.1). Media engagement also dropped, with the proportion of newspaper climate change articles mentioning health falling 10% between 2022 and 2023 (indicator 5.1).

The powerful and trusted leadership of the health community could hold the key to reversing these concerning trends and making people’s wellbeing, health, and survival a central priority of political and financial agendas. The engagement of health professionals at all levels of climate change decision making will be pivotal in informing the redirection of efforts and financial resources away from activities that jeopardise people’s health towards supporting healthy populations, prosperous economies, and a safer future. As concerning records continue to be broken and people face unprecedented risks from climate change, the wellbeing, health, and survival of individuals in every country now hang in the balance.

## Introduction

The devastation caused by record-breaking extreme weather events in 2023 and 2024 shows the human costs of a failure to curb greenhouse gas emissions and adapt to rapidly growing hazards. In 2023, annual global mean surface temperature broke all records, reaching 1·45°C above pre-industrial times; this 12-month record has also been breached again since then.^1,2^ Rapid attribution studies identified the influence of climate change in deadly events worldwide,^3^ including the floods that claimed over 300 lives in the Horn of Africa,^4^ the deadly heatwaves affecting much of the northern hemisphere,^5–7^ a record-breaking wildfire season in Canada,^8^ and many other events.^3^ At least 43 million child displacements were linked to extreme weather events over the past 6 years,^9^ and climate change-related extreme events are responsible for an estimated US$143 billion of annual losses.^10^ People in every country now face threats to their health and survival as climate hazards increase.

Current policies and actions, if sustained, put the world on track to 2·7°C of heating by 2100.^11^ The impacts seen to date could, therefore, be only the beginning of an increasingly dangerous future, with devastating impacts on the natural systems on which humanity depends.^12,13^

The outcome of the first Global Stocktake of the Paris Agreement, which culminated at 28th Conference of the Parties (COP28) of the United Nations Framework Convention on Climate Change (UNFCCC), noted with grave concern the growing impacts of climate change and the delays in necessary actions.^14^ Calling for a “transition away from fossil fuels in energy systems”, it was the first COP text in 30 years of negotiations to even acknowledge the need to address the use of fossil fuels in the energy system, which is the main driver of climate change. However, the final text reflected an over-reliance on carbon capture and storage—technologies that have not been developed or indeed proven to be safe at the necessary scale.

COP28 contributed to elevating health within global climate change negotiations with the first health thematic day. It also brought ministers of health and senior health officials to a UNFCCC COP for the first COP climate and health ministerial meeting, underscoring the imperative for health to be elevated in climate change negotiations. The inclusion of health in climate change negotiations was further bolstered by the endorsement of the COP28 Declaration on Climate and Health by 151 countries to date.^15,16^ The Global Stocktake recognised the right to health and to a healthy environment, and the Global Goal on Adaptation (GGA) set an overarching target towards the collective wellbeing of all people as well as a specific target for reducing the health impacts of climate change and promoting climate-resilient health services.^14^ Importantly, $1 billion was committed at COP28 to enable action on climate change and health. Although far from sufficient, this support could be an important enabler of progress. As countries work to update their Nationally Determined Contributions (NDCs) in response to the Global Stocktake, COP28 laid the grounds for countries to commit to ambitious, health-promoting climate change action tailored to the possibilities and needs of their people.

Complementing the health focus of climate negotiations, WHO’s Fourteenth General Programme of Work (GPW14) set the strategic objective of promoting health by responding to climate change and delivering climate-resilient health systems, as well as low greenhouse gas societies and health systems that contribute to better health and wellbeing. In addition, a new resolution on climate change and health adopted at the 77th World Health Assembly (WHA77) provides a platform for member states and WHO to develop and advance actions on climate change and health.

These milestones could provide new opportunities that pave the way to deliver a future of reduced life threats and improved health (panel 1). Nevertheless, much is still to be done to promote an integrated and health-centred response to the threats of climate change. Climate negotiations still largely feature health in the sidelines, without formal inclusion as agenda items, making people’s health and wellbeing a secondary and voluntary consideration. 2024 could also see a major geopolitical shift, with multiple armed conflicts and 64 countries—representing nearly half of the global population—holding major elections. Amid this geopolitical uncertainty and with misinformation increasing,^25,26^ upholding international agreements and driving evidence-informed action on climate change and health are imperative to protect the future of present and future generations.

### Advancing science and evidence for health-centred action on climate change

As the challenges of tackling climate change grow, robust scientific evidence is increasingly necessary to inform effective, health-protecting policies. In response to this need, the *Lancet* Countdown: tracking progress on health and climate change brings together over 300 leading researchers worldwide, to track the evolving links between health and climate change and help inform policies that enable a healthy, prosperous future. This effort, currently conducted through the global *Lancet* Countdown and its regional centres in Asia,^27^ Europe,^28^ Latin America,^27^ Oceania,^29^ and Small Island Developing States, will soon be expanded to Africa and South Asia.

The 2024 global report of the *Lancet* Countdown is the result of the expertise and dedication of 122 researchers, health professionals, and practitioners from 57 academic institutions and UN agencies globally. It provides a comprehensive assessment of the state of health and climate change, building on the 8 years of experience of indicator development and monitoring of the *Lancet* Countdown. Following the original framework and priorities laid out in the initial report of the *Lancet* Countdown^17,30^ and the priorities identified in consultation with global experts and policy makers,^30^ the set of indicators presented has been expanded and updated, harnessing the latest scientific developments. Following the *Lancet* Countdown’s indicator criteria, most indicators in this report feature improved methodologies or enhanced temporal or geographical coverage, and seven new indicators provide an increasingly comprehensive assessment of the global state of health and climate change. As in previous iterations, all new and substantially improved indicators were subjected to a review process whereby independent global experts evaluated their rigour and relevance before their inclusion in the present report.

The space constraints intrinsic to any academic publication limit the information that can be presented in this document. However, the 56 indicators (panel 2) can be explored in further detail in the *Lancet* Countdown’s online data visualisation platform. To support country-level decision making, a deeper assessment for specific countries is provided through a series of data sheets and policy briefs shared on the *Lancet* Countdown’s website. Complementing this report, the appendix provides further findings and methodological details and an in-depth description of indicator caveats, making it an essential document to adequately interpret the findings in this report.

### Rising to the challenge

In response to the escalating health threats of climate change, the *Lancet* Countdown is entering a new phase of increased capacity and a more comprehensive work programme, underpinned by a strategic partnership with *The Lancet* and WHO and enabled by 5 further years of generous funding and strategic support provided by Wellcome. Over the forthcoming 5 years, efforts will focus on ensuring that the collaboration’s rigorous scientific evidence can inform global and national progress on health and climate change. Metrics will be tailored to enable target setting and to monitor and evaluate national and international progress towards achieving the GPW14 goals and Paris Agreement ambitions. The *Lancet* Countdown’s indicator frameworks will be updated accordingly, to better reflect the priorities of all countries, harness the latest scientific developments, and address the needs of crucial policy processes.

This new phase will be enabled by an updated governance structure to increase transparency, scrutiny, and representation of researchers from different backgrounds in the collaboration. A new independent board will provide strategic guidance and oversight for the collaboration’s next phase of activities. In addition, the *Lancet* Countdown will continue to strengthen its regional centres, formally launching its Africa Regional Centre and expanding to new regions in the near future. This effort will support capacity building on health and climate change in some of the world’s most vulnerable regions, fostering international collaboration, supporting local policy makers, and increasing diversity and representation within the *Lancet* Countdown itself.

Throughout this new phase, the *Lancet* Countdown will operate an open and iterative process of indicator improvement, welcoming proposals for new indicators through its website and particularly encouraging the contributions of colleagues from minoritised communities and from the world’s most vulnerable countries, with the aim of increasing the diversity of voices in the *Lancet* Countdown’s work.

### Closing the data gap for a healthy future

A global scarcity of internationally standardised data hinders the capacity to optimally monitor the observed health impacts of climate change and evaluate the health-protective effect of implemented interventions. This scarcity also impedes the accurate assessment of progress against international commitments, hinders knowledge sharing, and undermines evidence-based planning and implementation of potentially life-saving interventions. The available data are rarely disaggregated by relevant groups (eg, gender, age, indigeneity, ethnicity, and socioeconomic level), impeding an optimal assessment of vulnerabilities and inequities. Additionally, Indigenous knowledge is often overlooked, and Indigenous populations are seldom taken into consideration in the production and reporting of evidence and data, increasing their marginalisation and vulnerability (panel 3).

With increased international commitments on climate change and health, improved data will be essential to evaluate progress and optimise resource allocation. In support of this effort, the *Lancet* Countdown will partner with WHO to bridge the data gap by improving the availability of national-level data and delivering guidance, blueprints, and tools to support countries in standardised data collection and reporting. Throughout the next 5 years, the priority for the collaboration will be to deliver rigorous and actionable scientific data and to move from tracking the soaring health threats of climate change to informing policies that enable a healthy future for all.

## section 1: health hazards, exposures, and impacts

Complex interactions between growing climate-related hazards, exposures, and vulnerability are resulting in the health impacts of climate change. Decades of delay in climate change mitigation and adaptation have intensified these impacts. Record-breaking extreme weather events were registered worldwide in 2023, with extreme heatwaves, wildfires, storms, floods, and droughts affecting people and the systems and economies on which their health depends.

section 1 details the evolving health hazards, exposures, and impacts of climate change, covering the effects of increasing heat, extreme weather, transmission of climate-sensitive infectious disease, and food insecurity. Three new indicators offer an increasingly comprehensive picture, measuring exposure to extreme precipitation, exposure to desert dust, and the effect of rising night-time temperatures on sleep loss.

Throughout this section, the continued scarcity of data stratified by vulnerable population groups limits the capacity to reflect the disproportionate impact of climate change on minoritised groups, including Indigenous peoples, women, children, people from minoritised ethnic backgrounds, and underserved communities—a global challenge that hampers an efficient and equitable response to climate change.^75–79^

The uneven temporal coverage of the data sources of the indicators presented hampers the possibility of adopting a consistent year range as a baseline. Therefore, in this section, and given available input data, indicator baselines were selected to best represent earlier conditions against which changes can be detected and measured. Baseline years are clarified for each indicator presented, and further information is provided in the appendix (pp 8–117).

### 1.1 Heat and health

Global mean surface temperatures reached a record-breaking 1·61°C above pre-industrial times between May, 2023, and April, 2024,^1^ with increasingly frequent and intense extreme heat events globally.^80,81^ The following indicators track the risks that heat exposure poses to people’s survival, health, and wellbeing.

#### Indicator 1.1.1: exposure of vulnerable populations to heatwaves—headline finding: in 2023, infants and adults older than 65 years experienced a new record high of 13·8 days of heatwave per person, on average

Heatwaves represent an acute health hazard, especially for older people, very young children, and those living with underlying chronic cardiovascular, respiratory, or kidney diseases.^82^ They also increase the risk of adverse pregnancy and birth outcomes and exacerbate adverse neurological conditions.^83,84^

This indicator tracks the exposure of vulnerable age groups (<1 year and >65 years) to heatwave days. For the purpose of this indicator, heatwaves were defined as a period of 2 or more days on which both the minimum and maximum temperatures were above the 95th percentile of the local climatology (defined on the 1986–2005 baseline).^85^ In an improvement from previous years, this indicator uses updated demographic data and,^85–88^ to distinguish the influence of an increase in the number of heatwaves from the influence of demographic changes, a counterfactual scenario was created, keeping heatwave incidence constant at baseline levels.^85–88^

In 2023, people from vulnerable age groups experienced a record total of 13·4 billion person-days of heatwaves (and a record average of 13·8 heatwave days per person), exceeding the previous high of 11·1 billion days (2022) by over 20%. If heatwave incidence had remained constant since 1986–2005, vulnerable people would have experienced 4·7 heatwave days per person on average per year in 2004–23, 45% less than observed in this period. Each infant experienced, on average, 8·2 more days of heatwaves in 2023 than in 1986–2005, and adults older than 65 years experienced an extra 9·3 days of heatwaves.

#### Indicator 1.1.2: heat and physical activity—headline finding: on average, in 2023, people were exposed to a record 27·7% more hours per year during which ambient heat posed at least a moderate risk of heat stress during light outdoor exercise, compared with 1990–99

Regular exercise provides physical and mental health benefits,^89,90^ and walking and cycling can contribute to decreasing transport-related greenhouse gas emissions and air pollution when acting as substitutes for fossil fuel-based transportation (indicators 3.1.3 and 3.2.1).^91^ However, heat stress can reduce the willingness to engage in physical activity and increase the health risks for those exercising outdoors.^92^ This indicator uses ambient temperature, humidity, and solar radiation to estimate the number of hours during which light outdoor physical activity (eg, walking) presents a risk of heat stress.

In 2023, people were exposed, on average, to a record high of 1512 hours during which ambient heat posed at least a moderate risk of heat stress during light outdoor exercise—328 hours (27·7%) above the 1990–99 annual average. In 2014–23, the average number of hours per year entailing this same risk was 262 hours (22·1%) more than in 1990–1999. The greatest percentage increase from 1990–1999 to 2014–2023 was observed in very high HDI countries (150 hours per person; 36·0%), and the largest absolute increase was observed in medium HDI countries (255 hours per person; 12·3%).

#### Indicator 1.1.3: change in labour capacity—headline finding: a record high of 512 billion potential work hours were lost in 2023, 49% above the 1990–99 average

Heat exposure outdoors or in non–cooled indoor environments puts workers’ health at risk.^93^ In addition, heat exposure reduces labour productivity and harms the livelihoods of workers and their dependents, particularly when affecting access to quality nutrition, health care, housing, or health-supporting services.^94,95^

This indicator has two distinct parts. The first monitors the number of outdoor workers (a population at risk), with estimates produced by WHO staff. The second part tracks potential work hours lost because of heat exposure, by considering temperature, humidity, solar radiation (via wet bulb globe temperature), and the typical metabolic rate of workers in specific economic sectors, through well established epidemiological models.^93,96^

Globally, in 2023, an estimated 1·6 billion people, or 25·9% of the working-age population, worked outdoors. The proportion of outdoor workers is highest in low HDI countries (30·8% of the workforce), followed by medium HDI (27·7%), high HDI (25·0%), and very high HDI countries (22·7%). These figures reflect the disproportionate impact on workers in the world’s most underserved regions.

Heat exposure led to a record high of 512 billion potential work hours lost in 2023 due to heat exposure, 49% above the 1990–99 average. Low and medium HDI countries were worst affected, with averages of 221 and 291 potential work hours lost per worker in 2023, respectively; high and very high HDI countries lost an average of 89 and 41 potential work hours per worker, respectively. Low and medium HDI countries bear a growing share of the world’s potential work hours lost due to heat, up from 57% in 1990 to 71% in 2023.

Of the global potential work hours lost in 2023, 63% occurred in the agricultural sector. This proportion is even higher for low (80·5%) and medium (64·8%) HDI countries, disproportionately affecting the most vulnerable agricultural workers, on whom local food availability often depends.

#### Indicator 1.1.4: rising night-time temperatures and sleep loss—headline finding: sleep hours lost due to high temperatures increased by 5% between 1986–2005 and 2019–23, reaching a record 6% in 2023

Sleep of adequate duration and quality is important for good human physical and mental health.^97–100^ High ambient temperatures are associated with worse sleep quantity and quality. ^101–106^ With climate change resulting in night-time temperatures rising faster than daytime temperatures in many world regions, the risk of adverse health outcomes from poor sleep quality is rising globally.^107^

**Figure 1:**
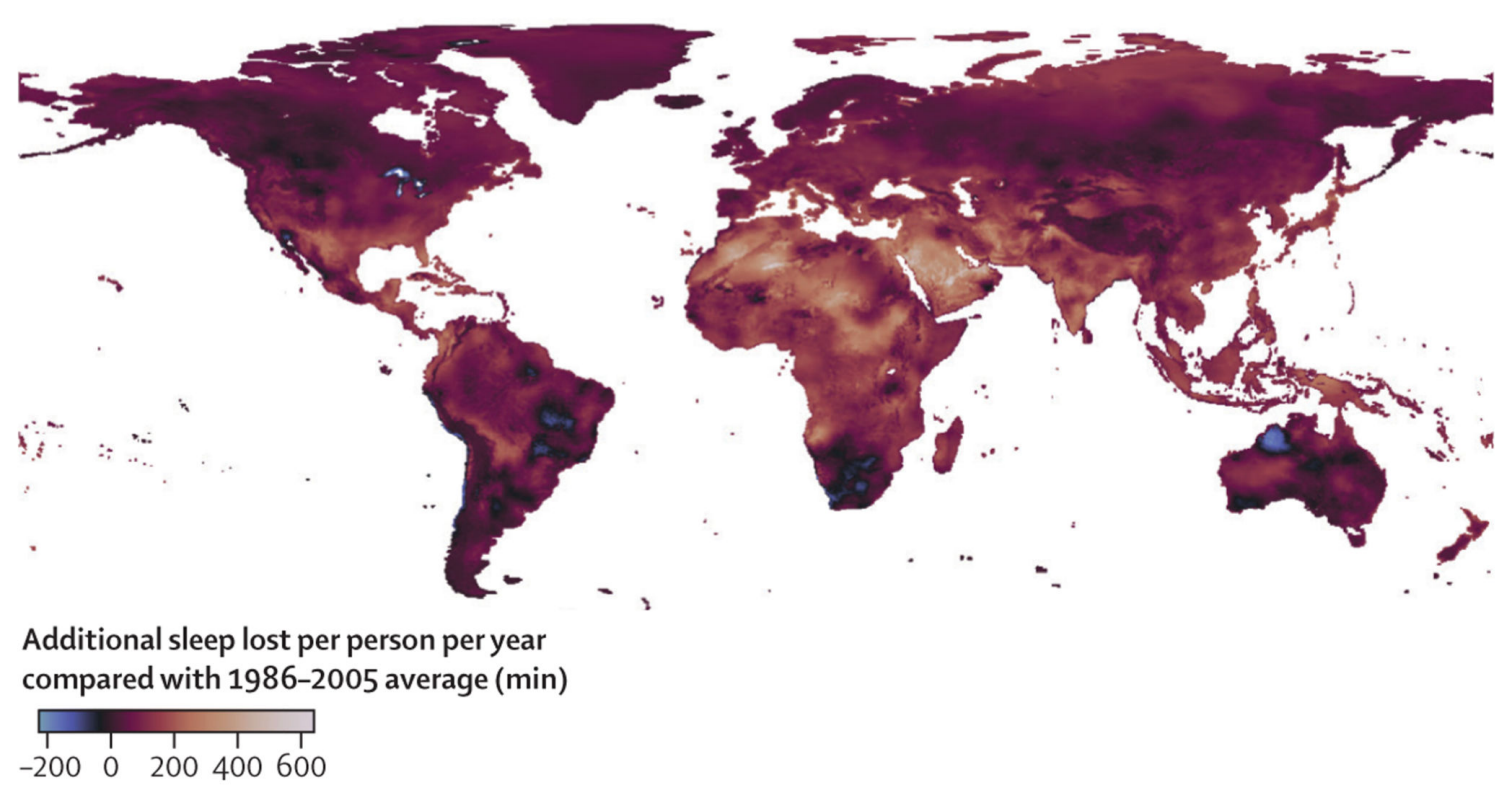
The average net change in annual temperature-attributed sleep loss in 2019–23, compared with 1986–2005

This indicator—new to this year’s report—tracks the impact of suboptimal night-time temperatures on sleep loss. It combines the global functional sleep response to night-time temperature identified in a multicountry sleep study^106^ with night-time temperature data^88^ and statistically controls for individual-level demographic and environmental factors, including access to air conditioning. Findings suggest that high night-time temperatures led to an average estimated 5% more sleep hours lost in 2019–23 than in 1986–2005, reaching a record high of 6% more sleep hours lost in 2023 (figure 1).

#### Indicator 1.1.5: heat-related mortality—headline finding: because of climate change, people faced, on average, a record 50 more days of health-threatening heat in 2023 than expected without temperature change

Rising temperatures are increasing the risk of heat-related morbidity and mortality. Although cold-related deaths currently exceed heat-related deaths, heat-related deaths are expected to exceed cold-related deaths in a high-warming scenario.^108,109^ This indicator, therefore, monitors the growing heat-related mortality risk. For this purpose, minimum mortality temperature is conservatively defined as the 84·5th percentile of the 1986–2005 daily average. As temperatures rise above this threshold, the risk of health impacts, including death, increases. The first part of this indicator monitors exposure to health-threatening days, defined as those in which temperature exceeds the locally defined minimum mortality temperature, and compares it with the number of days exceeding the temperature threshold that would have been expected without anthropogenic climate change. The second part estimates the change in heat-related mortality by combining the change in demographics and temperature in an epidemiological model.^110,111^

**Figure 2:**
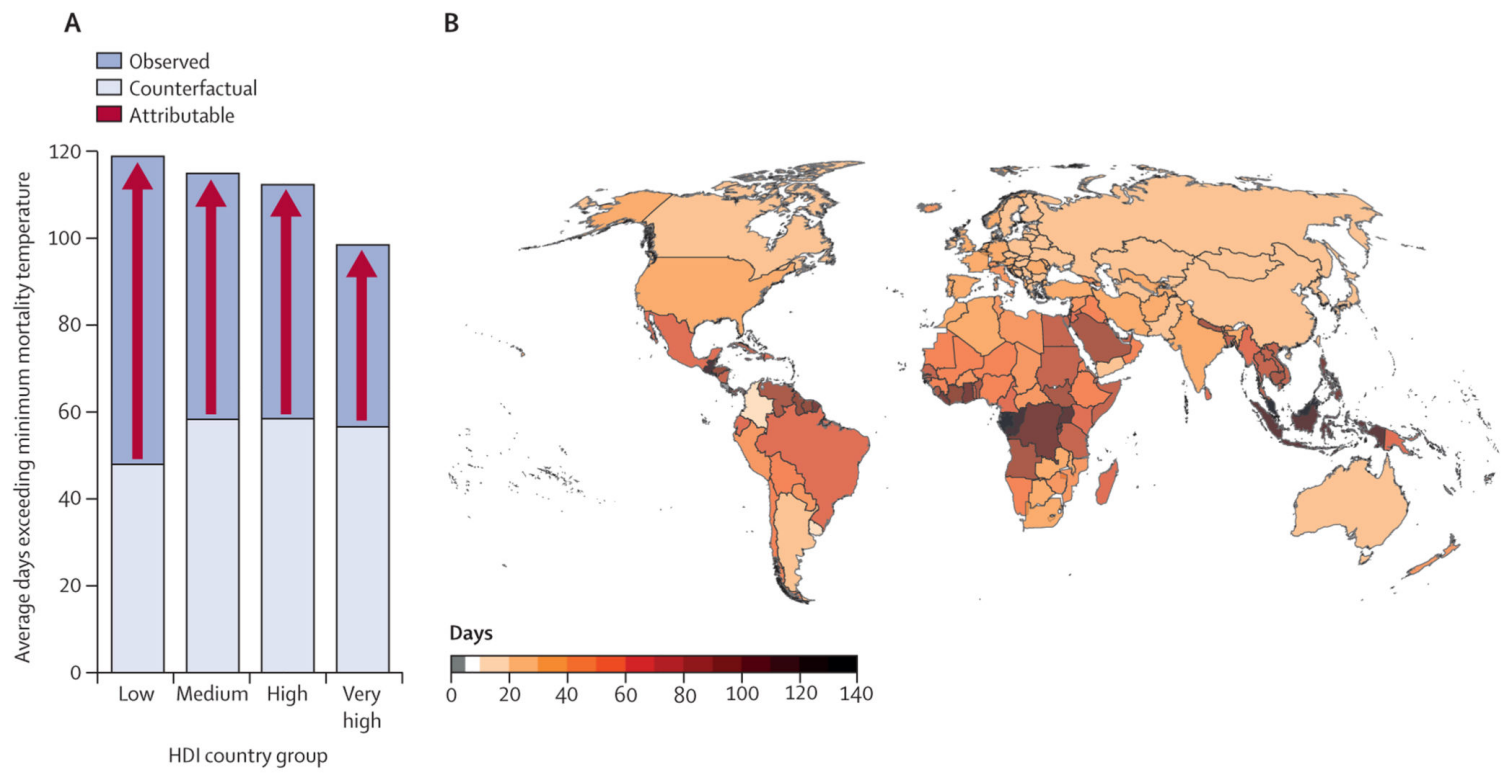
Days of health-threatening temperature in 2019–23 (A) Stressful heat days in 2019–23, by HDI category. The darker blue indicates observed total stressful heat days, and the lighter blue indicates the number of stressful heat days that would have been expected without human-caused warming. Red arrows indicate heat days attributable to climate change. (B) Average number of days with health-threatening temperature attributable to climate change in 2019–23, by country. HDI=Human Development Index.

In 2019–23, people were exposed, on average, to 46 more days of health-threatening heat than would have been expected without climate change, a value that reached a record high of 50 more days in 2023 (figure 2). In 2023, 31 countries experienced at least 100 more days of health-threatening heat than would have been expected with no climate change. The number of health-threatening heat days added by climate change decreases with increasing HDI level, reflecting strong global inequalities in heat exposure (figure 2).

Rising temperatures and ageing populations resulted in a 106% increase in the number of average annual heat-related deaths of adults older than 65 years between 1990–99 and 2014–23, 139% higher than the 44% increase expected if temperatures had not changed from baseline levels. In 2023, heat-related deaths in this age group reached the highest level recorded, 167% higher than in 1990–99 and more than double the 65% increase expected if temperatures had not changed since the 1990s.

### 1.2 Health and extreme weather-related events

Climate change is increasing the frequency and intensity of extreme weather events. Compounded by delays in adaptation and vulnerable human systems and infrastructure, there were widespread health impacts and deaths from extreme weather globally in 2023. This set of indicators monitors the growing health hazards from extreme weather events, human exposure to such hazards, and the resulting health impacts, with new indicators monitoring extreme precipitation and exposure to sand and dust storms.

#### Indicator 1.2.1: wildfires—headline finding: the average number of days of human exposure to very high or extremely high fire danger increased in 124 (66%) countries from 2003–07 to 2019–23

Higher temperatures and more frequent and intense droughts linked to climate change increase the risk of wildfires, which affect physical and mental health directly through burns and smoke exposure and indirectly through infrastructure damage, service disruption, and loss of assets. In 2023, wildfires caused devastation in Canada, Greece, the USA, Algeria, Chile, and Kazakhstan.

The first part of this indicator tracks exposure to the meteorological risk of wildfire and exposure to active wildfires by overlaying population data with the Copernicus Emergency Management Service fire danger indices^112^ and with satellite observations of active wildfires. The second part models the mean annual exposure to wildfire smoke, combining satellite data and atmospheric modelling.

In 2019–23, people were exposed to an average of 10 additional days (11% more) of very high—or extremely high—risk of wildfires, compared with 2003–07. Mean exposure to days of very high or extremely high risk of wildfires increased in 124 countries between 2003–07 and 2019–23, decreasing in only 45 countries. The annual average exposure of people to active wildfires was higher in 95 countries in 2019–23 than in 2003–07, whereas 106 countries saw a decrease.

Annually, people experienced 5% fewer days (0·07 days) of exposure to atmospheric concentrations of wildfire-related PM_2·5_ that exceeded the WHO threshold of 15 µg/m^3^ in 2014–23, compared with 2003–12. Across this same period, 119 countries saw a decrease in the number of days above this threshold, whereas 64 saw an increase. The reduction of wildfire exposure could be attributed to prevention and management actions, reduced availability of burning material due to previous wildfires or land use change, or a change in population distribution. However, as the climate changes and the risk of wildfires escalates, increased control and management of wildfires are essential to protect people from their harms.

#### Indicator 1.2.2: drought—headline finding: in 2023, 48% of the global land area was affected by at least 1 month of extreme drought, the second-highest level since 1951

Anthropogenic climate change increases the likelihood and severity of droughts,^4,113,114^ which can affect vector-borne and water-borne disease transmission, jeopardise water supply, food security, and livelihoods, and disrupt power generation and the transport of goods via inland waterways.^115–117^ 2023 saw record droughts in parts of South America, triggering a critical water shortage in Uruguay and the loss of 15% of cereal production in Argentina,^80,118^ and the drought in Somalia was linked to 531 000 displacements.^80^

This indicator uses the Standardised Precipitation Evapotranspiration Index to monitor the intensity and length of droughts on all land areas.^119,120^ The total proportion of global land affected by extreme drought for at least 1 month per year increased from 15% in 1951–60 to 44% in 2013–24. In 2023, 48% of the global land area was affected by at least 1 month of extreme drought—the second-highest level since 1951 and only 2% less than the record in 2020.

#### Indicator 1.2.3: extreme precipitation—headline finding: in 2014–23, 61% of all global land saw an increase in extreme precipitation events, compared with the 1961–90 average

Climate change alters the hydrological cycle, increasing the frequency and intensity of extreme precipitation over most land areas.^121–124^ Extreme precipitation is associated with adverse physical and mental health outcomes.^125–127^ When leading to floods, it can increase the risk of injury or drowning,^128^ infrastructural damage, environmental degradation, waterborne disease outbreaks, and disruption to social, ecological, and economic life support systems, affecting lives and livelihoods.^129–131^

**Figure 3:**
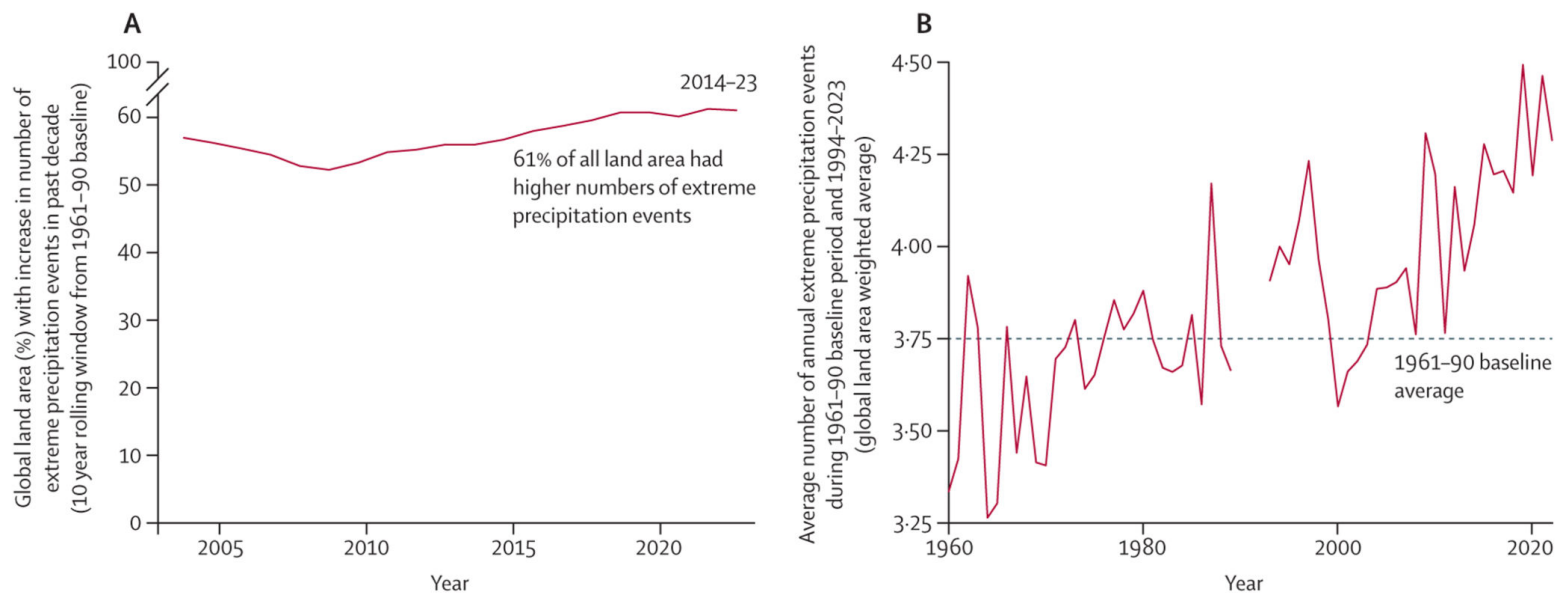
Extreme precipitation events over time Extreme precipitation events are defined as those exceeding the 99th percentile of daily precipitation during the baseline period of 1961–90. (A) Percentage of global land area where the number of extreme precipitation events increased during the previous decade. The red line depicts rolling 10-year averages—ie, the point above the year 2023 represents the percentage of global land cover where the average number of extreme precipitation events observed during the most recent decade (2014–23) exceeded the decadal average during the baseline period (1961–90). (B) Average number of annual extreme precipitation events per 79 km^2^ average land area in baseline years (1961–90) and during the most recent 30-year period (1994–2023).

This indicator—new in the 2024 report—tracks changes in extreme precipitation events, defined as those exceeding the 99th percentile of 1961–90 precipitation events, using ERA5-Land data.^132^ Compared with 1961–90, extreme precipitation events over land increased by a global average of 9·7% during 1994–2023, equivalent to an average 3·6 additional extreme precipitation events per 0·1°×0·1° grid-cell area (79 km^2^ mean land area) per decade. During the last decade (2014–23), extreme precipitation events increased over 61% of land areas (figure 3).

#### Indicator 1.2.4: sand and dust storms—headline finding: on average, during 2018–22, 3·8 billion people were exposed to mean annual concentrations of PM_10_ from sand and desert dust that exceeded WHO guideline levels, up by 31% from 2003–07

Drought, poor land management, and increased wildfire-burned areas are increasing the risk of sand and dust storms.^3,22^ The major component of particulate matter during a sand and dust storm is the mineral (also known as crustal) fraction. Mineral dust contributes to air pollution from particles with a diameter of 10 µm or less (PM_10_), exposure to which increases the risks of asthma, cardio-vascular disease, and premature death.^133–135^ Transported mineral dust can also spread soil-dwelling pathogens^136,137^ and cause transportation accidents through reduced visibility.^138,139^

**Figure 4:**
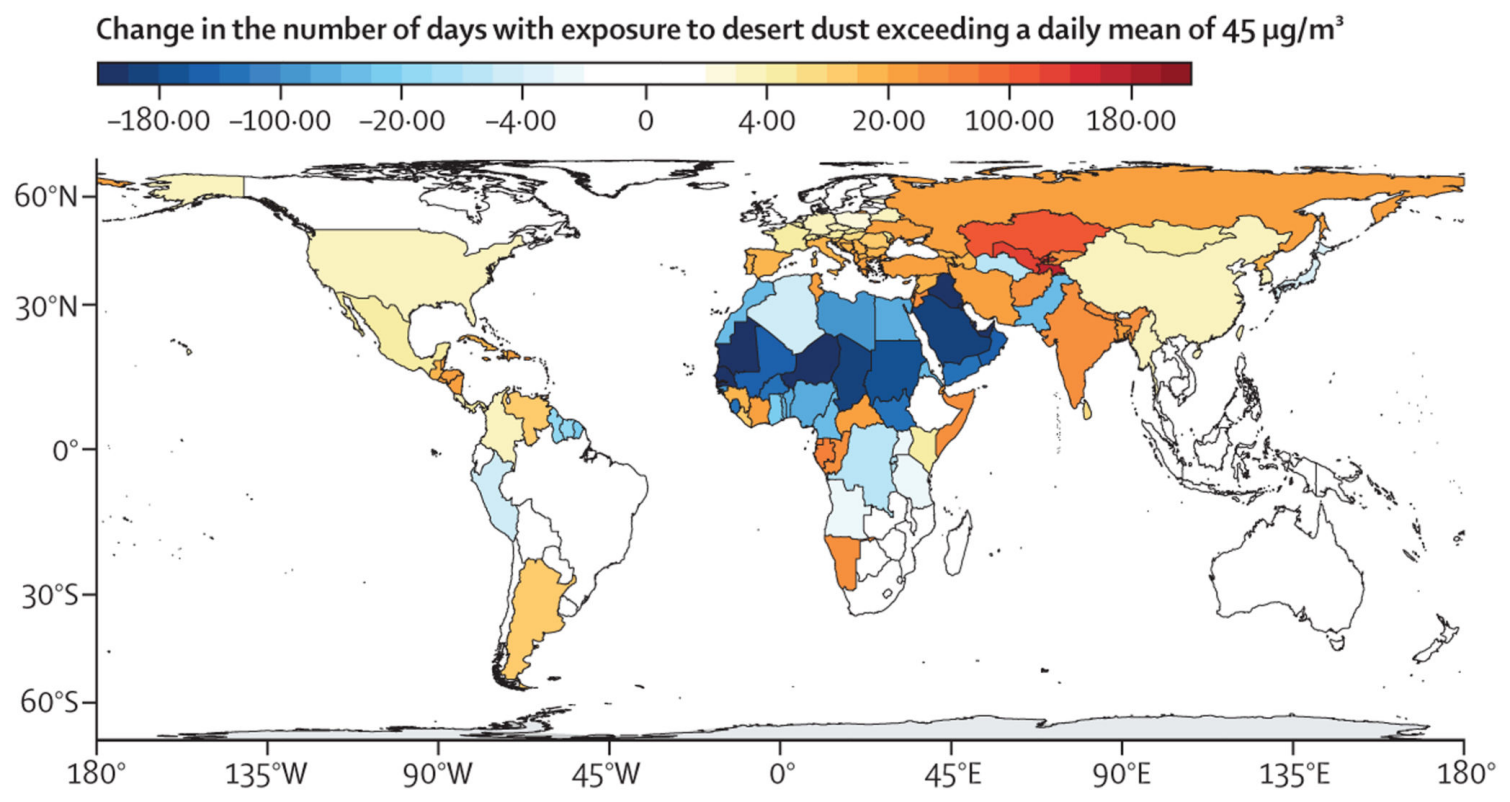
Difference in average population-weighted days when exposure to desert dust was higher than 45 µg/m^3^, comparing 2018–22 with 2003–07

This indicator uses a state-of-the-art multimodel reanalysis ensemble to estimate PM_10_ emissions from arid and semi-arid regions (referred to hereafter as dust-PM_10_),^140^ overlaying it with population data to estimate human exposure. Globally, in 2018–22, 3·8 billion people (48·9% of the world’s population) were exposed to average annual concentrations of dust-PM_10_ exceeding WHO’s annual threshold of 15 μg/m^3^ of total PM_10_—up by 31% from 2·9 billion (44·5% of the world population) in 2003–07. From 2003–07 to 2018–22, the number of days people were exposed to dust-PM_10_ concentrations higher than WHO’s daily PM_10_ guidance threshold (45 μg/m^3^) increased in 42% of countries and decreased in 36% (figure 4).^141^ The number of days people were exposed to these unhealthy concentrations ranged from zero to a few days in unaffected areas to more than 87% of days (1600 days during the 5-year periods) in the dustiest regions. Two-thirds of the countries with higher mineral dust exposure are high or very high HDI countries, and 47% of the countries with lower mineral dust exposure are low or medium HDI countries.

#### Indicator 1.2.5: extreme weather and sentiment—headline finding: in 2023, extreme heat events cumulatively worsened human sentiment by a record 53% more than the baseline average effect between 2006 and 2022

Extreme heat can affect human mental health outcomes across a continuum of severity, from subclinical to life-altering.^102,142–147^ This year’s indicator introduces a modified methodology. First, it links geolocalised X (formerly Twitter) posts with coincident meteorological data to estimate the effect of heat exposure on expressed sentiment using a multivariate fixed-effects regression.^147^ Second, it overlays this response effect with observed temperatures, to estimate the change in annual heat-attributable online sentiment expression.

Over the last 10 years, on average, extreme heat events worsened sentiment by 18% (mean 95% CI: 2%–33%) more than the estimated baseline effect. These findings suggest that the annual sentiment-worsening impacts of heat have increased globally. The largest estimated annual sentiment burden of the last decade was evident in 2023, at 53% above baseline.

### 1.3 Climate suitability for infectious disease transmission

The changing climate, alongside changes in land use (often induced by or contributing to climate change) and human movement, is affecting the risk of water-borne, vector-borne, food-borne, and air-borne disease transmission, undermining disease control efforts.^148,149^ The following indicators track the changing environmental suitability for the transmission of important—and potentially deadly—climate-sensitive infectious diseases.

#### Indicator 1.3.1: dengue—headline finding: the climatic suitability for the transmission of dengue by Aedes albopictus and Aedes aegypti increased by 46·3% and 10·7%, respectively, between 1951–60 and 2014–23

The global burden of dengue has increased sharply over the last two decades, mostly driven by ever more suitable climatic conditions, increased human mobility, and urbanisation.^150–152^ Over 5 million cases of dengue were reported globally in 2023.^153^ Transmission is largely driven by changing distributions of mosquito vectors of the genus *Aedes*, primarily *Aedes albopictus* and *Aedes aegypti*.

This indicator uses an updated and validated mechanistic model incorporating data on temperature, rainfall, daylight duration, and human population density to assess dengue transmission dynamics.^154–156^

The annual average transmission risk (basic reproduction number [R0]) of *Ae albopictus* and *Ae aegypti* increased by 46·3% and 10·7%, respectively, from 1951–60 to 2014–23. The change was more pronounced in the high HDI country group (60·4% for *Ae albopictus* and 23·8% for *Ae aegypti*). Low, medium, and very high HDI country groups observed increases of 11·5%, 59·3%, and 20·1%, respectively, for *Ae albopictus*. Medium and very high HDI countries saw 8·6% and 12·8% increases, respectively, for *Ae aegypti*, but the low HDI group saw a 5·1% decrease in transmission risk. Similar trends were also observed for the transmission suitability of chikungunya and Zika viruses. Overall, the R0 for chikungunya transmission by *Ae albopictus* increased by 46%, and the R0 for Zika transmission by *Ae aegypti* increased by 10·8% globally in 2014–23.

#### Indicator 1.3.2: malaria—headline finding: between 1951–60 and 2014–23, an extra 17·1% of the global land area became suitable for the transmission of Plasmodium falciparum and an extra 21·8% for the transmission of Plasmodium vivax

This indicator uses temperature, precipitation, and relative humidity thresholds to track the length of the transmission season for the two malaria-causing parasites that pose the greatest threat to human health (*Plasmodium vivax* and *Plasmodium falciparum*), transmitted by *Anopheles* mosquitos.^157^ Between 1951–60 and 2014–23, the global average length of transmission season for *P falciparum* malaria remained stable, changing from 2·6 months to 2·7 months per year, on average. However, there was considerable heterogeneity across the globe. The length of the transmission season for *P falciparum* increased particularly sharply in the highland areas of low HDI countries (63·9% increase, about 40 additional days) and increased by 32·2% (an additional 0·9 months) in those of medium HDI countries and by 48·7% (an additional 0·7 months) in the highland areas of high HDI countries, putting health systems and individuals in these areas at risk; the increase was negligible in very high HDI countries. However, some endemic regions (eg, in parts of sub-Saharan Africa) had a reduction in transmission season length over the time period, as temperatures exceeded the threshold of survival of the parasite and mosquito vector. Overall, an extra 17·1% of the global land area became suitable for the transmission of *P falciparum*, and an extra 21·8% became suitable for the transmission of *P vivax*.

#### Indicator 1.3.3: Vibrio —headline finding: the environmental suitability for Vibrio transmission reached a record high in 2023, with 88 348 km of coastline with waters suitable for transmission in 2023, up by 14·8% from the previous record in 2018

Changes in the temperature and salinity of water bodies are affecting the transmission potential of water-borne diseases.^158^ Pathogenic non-cholera *Vibrio* bacteria can cause severe skin, ear, and gastrointestinal infections and life-threatening sepsis. They are transmitted through direct contact with contaminated brackish waters or through the consumption of contaminated seafood. As water temperatures rise, they become more suitable for *Vibrio* transmission. This indicator uses a mechanistic model that incorporates data on sea surface temperature and salinity to monitor the suitability for *Vibrio* transmission in coastal water.

A record 83 countries showed coastal water conditions suitable for the transmission of *Vibrio* pathogens at any one time in 2023, and the length of coastlines with suitable conditions reached a new record high of 88 348 km in 2023—up by 14·8% from the previous high in 2018, and 32% above the 1990–99 average. The total population living within 100 km of coastal waters with conditions suitable for *Vibrio* transmission reached a record high of 1·42 billion, and 2023 saw an estimated 692 000 vibriosis cases, setting a new record, increased by 13·5% from the previous record high in 2022.

#### Indicator 1.3.4: West Nile virus—headline finding: the temperature suitability for the transmission of West Nile virus increased by 4·3% from 1951–60 to 2014–23

West Nile virus is a mosquito-transmitted virus that can cause lethal neurological disease in humans. Transmission is maintained in a cycle between birds and mosquitoes (primarily of the genus *Culex*), from which it can spill over into human and other mammalian populations.^159^ The virus is found across the globe, with its range expanding in some world regions.^160^ This indicator considers three primary Culex West Nile virus vectors and tracks changes in the relative basic reproduction number of West Nile virus (WNV–R0), based on the response of vector–pathogen traits to temperature. These response relationships are derived from experimental studies.^161^ Driven by changes in temperature, WNV–R0 was, on average, 4·3% higher in 2014–23, compared with 1951–60, in the regions where the three *Culex* mosquitoes occur. Increases in WNV–R0 in the same period occurred in very high (8·3%), high (6·2%), and medium (4·2%) HDI countries, whereas there was a decrease in low HDI countries (–1·1%).

### 1.4 Food security and undernutrition

#### Headline finding: the higher frequency of heatwave days and drought months in 2022, compared with 1981–2010, was associated with 151 million more people experiencing moderate or severe food insecurity across 124 countries

In 2023, 733 million people were undernourished, and 2·83 billion (35·5%) were unable to afford a healthy diet in 2022.^162^ Climate change is exacerbating food insecurity and undernutrition by reducing crop yields, labour capacity, and access to water and sanitation; disrupting supply chains; and compromising marine resources through higher coastal sea surface temperatures, reduced oxygenation, ocean acidification, and coral reef bleaching.^163,164^ Increased food insecurity contributes to malnutrition, which harms health and development.^165–167^ The impacts are especially acute for subsistence farmers and Indigenous peoples, for whom food availability is particularly sensitive to local climatic changes.^38,168–170^ The risk is also especially important for Indigenous children, who experience higher levels of malnutrition compared with non-Indigenous children, with severe implications for their health throughout the lifecourse.^38,168^

The first part of this indicator combines data from the Food and Agriculture Organization Food Insecurity Experience Scale^171–173^ from 124 countries (up from 122 in 2023) with the frequency of heatwave days and drought months (12-month Standardised Precipitation Evapotranspiration Index)^132^ during the growing seasons of maize, rice, sorghum, and wheat, using a time-varying panel regression. Compared with 1981–2010, a higher number of heatwave days was associated with 4·4 percentage points higher moderate or severe food insecurity in 2022, and increasing frequency of droughts was associated with 2·0 percentage points higher food insecurity. The combined effect is equivalent to approximately 151 million more people experiencing food insecurity in 2022 due to climate change, suggesting insufficient adaptation.

The second part of this indicator monitors the growing risk to marine yields by tracking sea surface temperature variations in coastal waters across 148 territories,^174^ with the finding that in 2021–23, average global sea coastal temperature exceeded the 1981–2010 average by 0·54°C. Moreover, 2023 marked a milestone, as average global coastal sea surface temperatures exceeded 20°C for the first time in recorded history. This shift underscores the global threat to marine food security induced by climate change.

### Conclusion

The rapidly growing health risks and impacts of climate change present complex public health challenges. Record-breaking temperatures in 2023 resulted in an unprecedented increase in heat-related deaths and caused a record loss of hours of potential labour, safe outdoor exercise, and quality sleep (indicators 1.1–1.5). The incidence and intensity of droughts and extreme precipitation are also growing, as are the risks of wildfires and exposure to desert dust in most countries (indicators 1.2.1–1.2.4). Record coastal water temperatures in 2023 put marine food yields at risk, and the impact of heatwaves and droughts on food insecurity has continued to increase (indicator 1.4). In parallel, the environmental suitability for the transmission of deadly diseases such as dengue, West Nile virus-related illness, malaria, and vibriosis has continued to increase (indicator 1.3). Although these evolving risks and hazards are monitored individually, they are often affecting populations simultaneously, with compounding impacts that aggravate overall health outcomes.

Importantly, indicators that capture health outcomes suggest that adaptation is not keeping pace with the growing hazards. As climate risks escalate, countries will need to dedicate increasing effort and resources to avoiding the worst health impacts.

Despite substantial improvements over the past 8 years, important gaps remain in the *Lancet* Countdown’s monitoring of the health risks and impacts of climate change. These gaps are often due to technical difficulties and data scarcity. In its new phase, the *Lancet* Countdown will systematically identify these gaps and work to close them. Special efforts will be dedicated to capturing the mental health impacts of climate change, an effort that has so far been hindered by the persistent lack of standardised definitions, frequent stigmatisation and lack of recognition of mental health, and scarcity of globally relevant data on mental health impacts and care.^175^ Efforts will also be allocated to monitoring the observed impacts of climate change and formally measuring the attribution to anthropogenic climate change.

## Section 2: adaptation, planning, and resilience for health

With climate change increasingly threatening human health (section 1), suitable, effective, and well funded adaptation measures are urgently needed to minimise adverse mental and physical health impacts and limit health-related losses and damages.^176,177^

The Paris Agreement established the GGA of “enhancing adaptive capacity, strengthening resilience and reducing vulnerability to climate change”.^178^ At COP28, the United Arab Emirates (UAE) Framework for Global Climate Resilience was adopted, providing an official framework that established health adaptation as one of the GGA targets.^179^ In addition, COP28 saw the establishment of the UAE-Belém work programme, with the purpose of developing indicators for measuring progress towards the targets outlined in the framework—a crucial process that will add definition to the targets and level of ambition.

Major progress towards climate change adaptation has been made within the health sector itself in 2024. WHO’s new GPW14 set a specific goal of delivering resilient health systems, and the second World Health Assembly resolution on climate change called upon WHO member states to commit to strengthen, invest in, and implement further adaptation actions, including to deliver climate-resilient health systems through multisectoral cooperation.

In support of these global efforts, this section reports progress and challenges in assessing, planning, and delivering climate change adaptation for health. It also presents conditions that facilitate health adaptation, both within and beyond the health sector.

### 2.1 Assessment and planning of health adaptation

A thorough assessment of health-related climate change risks and vulnerabilities is crucial to inform the planning of effective adaptation interventions that protect people’s health. This set of indicators tracks the progress on risk assessments and health adaptation planning.

#### Indicator 2.1.1: national assessments of climate change impacts, vulnerability, and adaptation for health—headline finding: as of December, 2023, 61% of the WHO member states that committed to building climate-resilient health systems through the COP26 Health Programme reported having completed a vulnerability and adaptation assessment, up from 17% the year before

Within the COP26 Health Programme in 2021, countries, territories, and areas (hereafter referred to as members) committed to building climate-resilient health systems. This commitment included conducting climate change and health vulnerability and adaptation assessments to inform Health National Adaptation Plans (HNAPs) and facilitate access to climate change funding for health. The Alliance for Transformative Action on Climate and Health (ATACH), led by WHO, supports members in meeting these commitments.^180^

As of December, 2023, 82 ATACH members have committed to building climate-resilient health systems through this programme. Of these, 50 (61%) have conducted a vulnerability and adaptation assessment, with 32 of these having done so since January, 2020, a substantial increase from the 11 (17%) of 64 countries that conducted assessments the year before. In 2023, 11 members reported having their first vulnerability and adaptation assessment under development, and 11 were updating previous assessments. Of all 82 ATACH members, 67% of low HDI countries, 76% of medium HDI countries, 53% of high HDI countries, and 56% of very high HDI countries had developed a vulnerability and adaptation assessment.

#### Indicator 2.1.2: National Adaptation Plans for health—headline finding: as of December, 2023, 52% of WHO members that committed to building climate-resilient health systems through the COP26 Health Programme reported having developed an HNAP, up from just 6% the year before

In 2010, COP16 established a process for the development of National Adaptation Plans, with the aim of reducing vulnerabilities to climate change and facilitating the integration of climate change adaptation into policies, programmes, and planning processes.^181^ HNAPs build on this initiative, focusing specifically on preparing for and adapting to the threats of a changing climate to health systems and people’s health. As of December, 2023, 43 (52%) of 82 ATACH members had developed an HNAP, 23 of which had been developed since 2020, an increase from the four (6%) of 64 of ATACH members that had developed an HNAP by 2022. In 2023, 14 countries reported having their first HNAP under development, and six countries were updating previous HNAPs. Of all 82 ATACH members, 61% with low HDI, 47% with medium HDI, 55% with high HDI, and 52% with very high HDI had developed an HNAP.

#### Indicator 2.1.3: city-level climate change risk assessments—headline finding: in 2023, 937 (96%) of 979 cities reported having completed or expecting to soon complete city-level climate change risk assessments

Home to 56% of the world’s population, cities have a major role in protecting health amid growing climate change risks.^182^ This indicator uses data from the CDP (formerly the Carbon Disclosure Project) to report on city-level assessments of climate change risks.^183^ In 2023, of the 979 cities responding to the climate risk assessment module, 937 (96%, 2% higher than in 2022) reported they had completed, were in the process of conducting, or were planning to conduct city-level climate change risk assessments within 2 years.

Of the 556 (57%) cities responding to the health module, 454 (82%) noted that climate change is affecting health outcomes, 173 (31%) noted impacts on health systems, and 49 (9%) noted impacts on other sectors relevant to health. Leading climate-related health hazards identified included extreme heat (412 [74%] cities), urban flooding (232 [42%]), heavy precipitation (216 [39%]), and infectious diseases (204 [37%]). Heat-related illnesses (447 [80%]), exacerbation of respiratory disease (294 [53%]), and vector-borne infections and illnesses (290 [52%]) were the leading public health issues identified.

Of the 42 (4%) cities reporting that they were not doing a climate risk assessment, ten (24%) indicated it was due to insufficient financial resources, 16 (38%) that it was due to technical capacity, and 11 (26%) indicated that both factors had a role.

### 2.2 Enabling conditions, adaptation delivery, and implementation

Strong governance, financing mechanisms, and access to education, information, and technology are essential for efficient adaptation. The following indicators track progress on health adaptation implementation and the conditions that enable it, including a new indicator on climate and health education for adaptation.

#### Indicator 2.2.1: climate information for health—headline finding: among World Meteorological Organization members, only 23% of ministries of health reported having public health surveillance systems that integrate meteorological information

Climate data and information services are crucial for establishing climate-informed public health surveillance, early warning, and response systems, which are vital for effectively anticipating and responding to climate-related health risks. Establishing these systems requires close collaboration between meteorological and health services.^184^

In 2021, 157 (81%) of World Meteorological Organization members reported providing climate services for health, but only 44 (23%) had at least one climate-informed public health surveillance system. Regarding health early warning systems, implementation ranged from 30 (35%) of 85 countries for vector-borne diseases and 28 (33%) of 84 for heat-related illness, to just eight (10%) of 83 for mental and psychosocial health, 12 (14%) of 84 for non-communicable diseases, and 12 (14%) of 83 for malnutrition and foodborne diseases.^184^ Notably, only 12 (15%) of 80 countries reported a health early warning system for impacts on health-care facilities. These data reflect the need for stronger collaboration between the health and climate sectors to increase the implementation of these potentially life-saving systems.

#### Indicator 2.2.2: benefits and harms of air conditioning—headline finding: greenhouse gas emissions from air conditioning use increased by 8% from 2016 to 2021; 48·4% of households in very high HDI countries had air conditioning in 2021 but only 4·7% of those in low HDI countries

Air conditioning is an effective technology for reducing heat exposure.^185^ However, it is expensive and energy-intensive, overwhelms energy grids on hot days, and can contribute to greenhouse gas emissions, air pollution, and the urban heat island effect.^186–188^ Therefore, although air conditioning can be a suitable option for vulnerable individuals if powered by renewable energy and used alongside passive and low-energy cooling solutions, it often represents a maladaptive response.

This indicator draws on International Energy Agency (IEA) data on air conditioning usage at a more granular geographical level than in previous years. It also builds on studies on the protective effect of air conditioning against heat-related mortality, using data from indicator 1.1.5 to estimate heat-related deaths of people older than 65 years potentially saved by air conditioning use.

The global proportion of households with air conditioning increased from 19·3% in 2000 to 30·4% in 2016 (the year the Paris Agreement entered into force) and to a record 35·3% in 2021. The average annual potential heat-related deaths of people older than 65 years averted by air conditioning increased by 36% from 2015–17 to 2019–21. In parallel, air conditioning-related CO_2_ emissions increased by 8% from 2016 to 2021.

Marked inequities exist in global access to air conditioning and the resultant benefits. In 2021, the proportion of households with air conditioning reached 43·8% in high HDI countries and 48·4% in very high HDI countries, compared with just 4·7% and 14·3% in low and medium HDI countries, respectively. Accordingly, the ratio of potential heat-related deaths prevented by air conditioning to actual heat-related deaths among those older than 65 years was 0·04 for low HDI countries, 0·12 for medium HDI countries, 0·74 for high HDI countries, and 0·76 for very high HDI countries. These data reflect the urgency of implementing equitable, affordable, and sustainable health-protecting cooling solutions to save lives.

#### Indicator 2.2.3: urban greenspace—headline finding: between 2015 and 2023, the proportion of urban centres with at least moderate levels of greenness remained constant, at 28%

Increasing equitable access to safe, adequately designed, and high-quality urban green spaces can help reduce the negative health impacts of climate change, reducing heat exposure and flood risk while offering physical and mental health co-benefits by improving air quality and offering spaces for exercise, social interaction, and connection with nature (panel 4).^195,196,199–202^ This indicator calculates a population-weighted average of Landsat’s normalised difference vegetation index to estimate greenspace exposure for 1041 urban centres (>500 000 inhabitants) across 174 countries.

**Figure 5:**
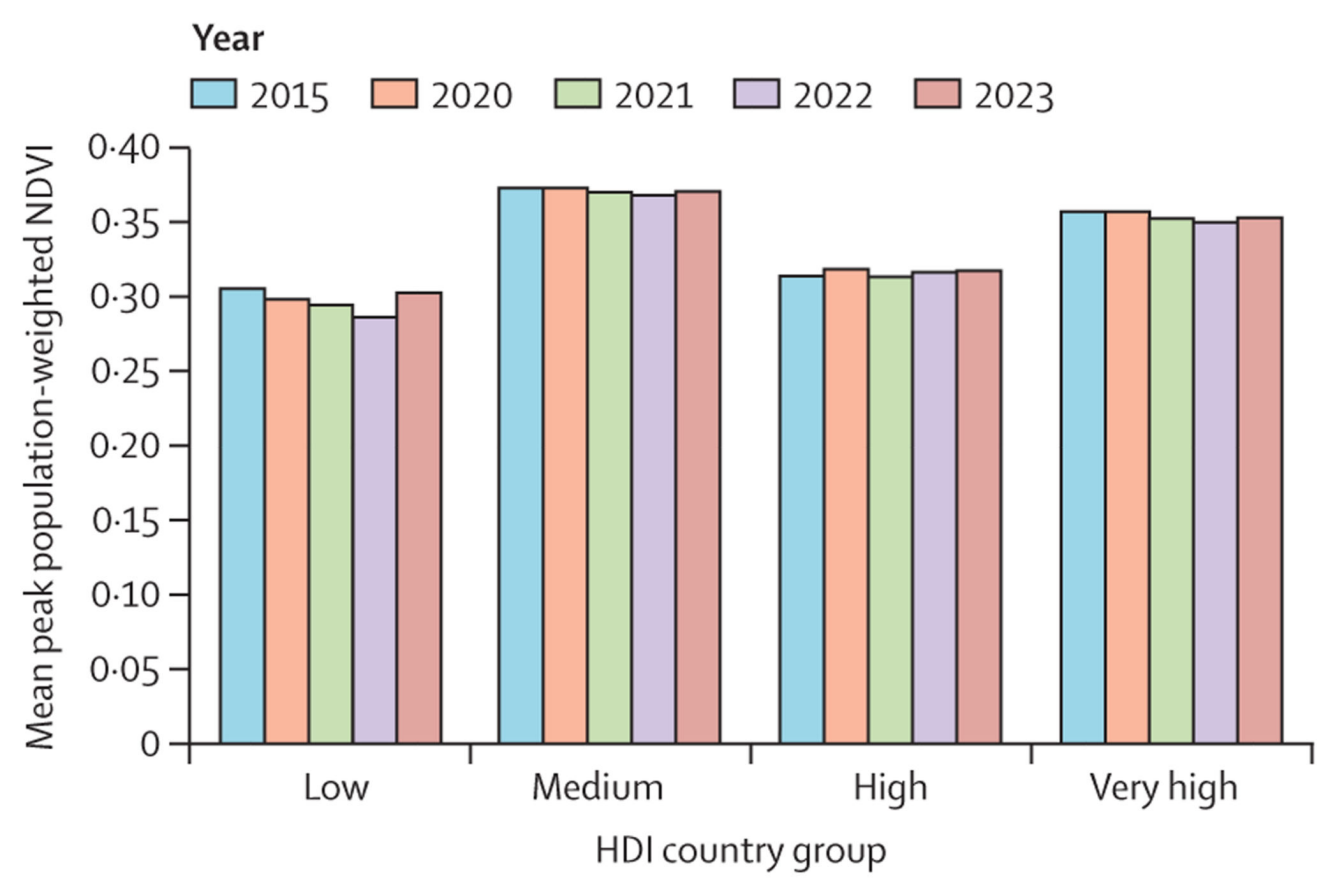
Mean population-weighted peak-season NDVI of urban centres by HDI and year NDVI=normalised difference vegetation index. HDI=Human Development Index.

The global average urban population-weighted normalised difference vegetation index has remained at 0·34 since 2015, the year the Paris Agreement was signed, and the percentage of cities with at least moderate exposure to greenness has remained constant at 28%. There have not been substantial changes across any HDI group (figure 5).

Although urban green spaces should be expanded with care to avoid potential unintended harms (eg, providing habitats for disease vectors, limiting cooling by reducing air flow, or introducing allergenic pollens),^192,203^ these data show that cities are not expanding urban green spaces at scale, neglecting a measure that could increase the resilience of urban populations in the face of climate change.

#### Indicator 2.2.4: global multilateral funding for health adaptation programmes—headline finding: in 2023, the Green Climate Fund (GCF) approved adaptation projects with potential health benefits for $423 million—up by 137% from 2021

Sustainable and just funding is essential to enable health-supportive climate adaptation and health system resilience, particularly in low-income and middle-income countries (panel 5). In support of this goal, the GCF enables the Paris Agreement to operationalise financial support to the developing countries (as defined by the UN),^212^ making it a key financial mechanism to support a just transition. This indicator tracks funding allocated by the GCF to health-related adaptation projects^213^ and the funding reported by members of ATACH in support of health adaptation and resilience.

The funding allocated by the GCF that identified adaptation outcomes increased from $1·05 billion in 2021 to $1·56 billion in 2023, following a dip to $0·68 billion in 2022. Of these, the proportion dedicated to projects with identified adaptation outcomes in health, food, and water security increased from 17% ($178 million) in 2021 to 27% in 2023 (US$ 423 million)—a 137% increase in total funding for projects with potential health adaptation benefits. Additionally, the GCF approved the first adaptation project aimed at strengthening the health system’s climate resilience in 2023, for $28·2 million.^214^

Complementarily, as of December, 2023, 25 ATACH members had reported 32 projects aimed at strengthening climate change and health resilience, totalling $550 million. During COP28, in the context of the first COP Health Day, US$1 billion was announced in support of climate change and health over the next 3–5 years. Although still grossly insufficient, this funding represents a step in the right direction. In upcoming years, the *Lancet* Countdown will seek to monitor the roll-out of this funding.

#### Indicator 2.2.5: detection of, preparedness for, and response to health emergencies—headline finding: from 2022 to 2023, 48 (26%) of 185 WHO member states reported an increase in the implementation of health emergency management capacity, whereas 54 (29%) reported a decrease

Climate-related health risks, particularly those related to infectious diseases, require robust health emergency preparedness and response systems to reduce the risk of outbreaks, epidemics, and pandemics.^215^ This indicator uses data from the electronic States Parties Self-Assessment Annual Reporting tool to monitor the self-reported level of implementation of the legally-binding International Health Regulations core capacities 7 (health emergency management) and—an improvement this year—3.2 (financing for public health emergency response), which are legally binding.^216^

In 2023, 131 (68%) of 193 countries reported high-to-very-high implementation (a capacity 7 score of 61–100) of health emergency management, of which 51 (39%) were very high HDI countries, 39 (30%) were high HDI countries, 23 (18%) were medium HDI countries, and only 15 (11%) were low HDI countries. Of the 185 countries that had also reported their implementation status in 2022, 48 had increased their implementation, whereas 54 had decreased their capacity. The low HDI country group showed the least progress, with just eight (17%) of these countries increasing their capacity.

The implementation of capacity 7 is positively associated with that of capacity 3.2, with very high and high HDI countries having high levels of implementation for both. Low HDI countries tend to have low-to-medium levels of implementation for both capacities.

#### Indicator 2.2.6: climate and health education and training—headline finding: in 2023, 70% (196) of 279 public health education institutions worldwide reported providing education in climate and health

Public health professionals have a crucial role in developing and implementing health-promoting adaptation and mitigation interventions.^217^ However, the integration of climate change education and training is largely not mandated in public health curricula, leaving many public health professionals ill-prepared for this purpose.^218^

This indicator builds on an international survey of degree-granting public health education institutions to assess the current state of climate and health education and training among them. Of the 279 public health education institutions responding to the survey in 2023, 196 (70%) reported providing education in climate and health, and 108 (39%) reported that training in climate was a mandatory component of their curriculum, covering approximately 45 000 students. Most (60%) of those providing climate change and health education did so through master’s degree programmes.

**Figure 6:**
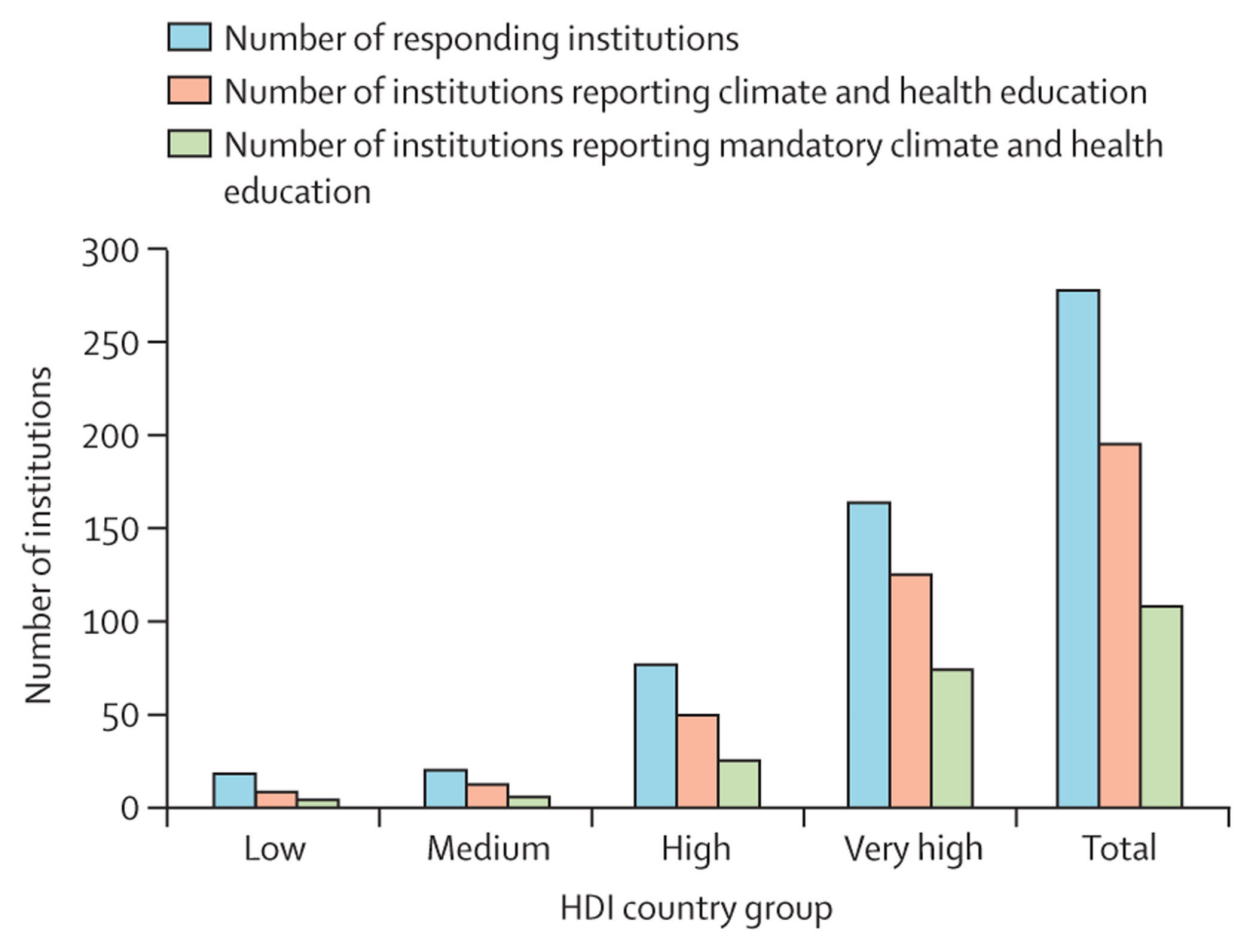
Number of responding institutions reporting providing climate and health education, by HDI country group HDI=Human Development Index.

Very high HDI country-based institutions accounted for 59% of total respondents, whereas 28% were high HDI, 7% were medium HDI, and 6% were low HDI institutions (figure 6). This selection bias impedes a reliable analysis by HDI group, and efforts will focus on increasing the representation of lower HDI countries in this survey in upcoming years. However, a preliminary analysis suggests that low HDI countries had the lowest proportion of responding institutions offering climate change and health education, which could mean that the most vulnerable countries might lag in building adaptive capacity for health, amplifying the inequities driven by climate change. In upcoming years, this indicator will be further expanded to evaluate climate change and health education in medical education.

### 2.3 Vulnerabilities, health risk, and resilience to climate change

A core goal of adaptation is to build resilience and reduce vulnerabilities to growing climate change-related health hazards. This group of indicators tracks vulnerabilities and risks to climate hazards and adaptation responses.

#### Indicator 2.3.1: vulnerability to severe mosquito-borne disease—headline finding: the very high HDI country group was the only group in which vulnerability to severe Aedes-borne disease increased between 1990–99 and 2014–23, rising by 5·4%

Dengue incidence is growing globally, driven by increasingly favourable climatic conditions, population mobility, urbanisation, and susceptibility to circulating serotypes (indicator 1.3).^219,220^ An estimated 40 000 individuals die annually from severe dengue.^221^ However, adequate medical care and early intervention can reduce the fatality rate to less than 1%.^222^ This indicator captures relative vulnerability to severe dengue by combining increased susceptibility from urbanisation and coping capacity from improved health-care access and quality.

Mostly due to improvements in health care, vulnerability to dengue was reduced by 46% and 32% in low and medium HDI countries, respectively, between 1990–99 and 2014–23. However, rapidly increasing urbanisation limited the reduction in vulnerability to just 2% in high HDI countries and drove an increase in vulnerability of 5·4% in very high HDI countries. With exposure to dengue growing as climate change increases the environmental suitability for its transmission (indicator 1.3.1), interventions to reduce vulnerability, including dengue response capacity within health systems, integrated vector control measures to control mosquito populations, early warning and early response systems, and population awareness campaigns, are urgently needed.

#### Indicator 2.3.2: lethality of extreme weather events—headline finding: the mortality of extreme weather events decreased by 73% between 2000–09 and 2014–23 in countries with climate-informed health early warning systems but only decreased by 21% in countries without such systems

Under a changing climate, extreme weather events are increasing in frequency, intensity, and duration,^223^ threatening the health, wellbeing, and survival of individuals globally.^177^ However, the implementation and community uptake of health early warning systems could reduce the risk of the most severe health outcomes and death.^224^

This indicator combines data registered in the Centre for Research on the Epidemiology of Disasters’ emergency events database, EM-DAT, with data from the 2021 WHO Health and Climate Change Survey Report^225^ to explore the relationship between mortality rates associated with disasters involving floods or storms and the implementation of climate-informed health early warning systems. The change in mortality for countries reporting the existence of a health early warning system was also assessed by HDI country group. Poisson regression models were fitted to evaluate statistical significance of the observed changes in mortality.

The mortality rates associated with disasters relating to floods and storms in countries that replied to the 2021 WHO Health and Climate Change Global Survey have decreased since 2000. Countries with health early warning systems saw a 73% decrease in mortality, from an average 1·68 deaths per million people per event in 2000–09 to 0·46 deaths per million people per event in 2014–23. In countries without such early warning systems, however, the decrease was only 21%, decreasing from 2·84 to 2·23 deaths per million people per event between 2000–09 and 2014–23, on average. Although the reduction cannot be directly attributed to the implementation of health early warning systems, it is likely that the state of implementation of these systems correlates with a more widespread engagement with climate change adaptation efforts, which could be collectively contributing to the observed reduction in lethality. Irrespective of implementation, the analysis of mortality in countries with health early warning systems level reported by HDI group shows that, from 2000–09 to 2014–23, the largest absolute decreases in mean mortality rate per event occurred in low HDI countries (from 10·71 deaths to 1·71 deaths per million people) and medium HDI countries (from 6·61 deaths to 1·67 deaths per million people), followed by high HDI countries (0·65 deaths to 0·3 deaths per million people). In contrast, over this time period, the recorded mean mortality rate in very high HDI countries increased from 0·37 deaths to 2·93 deaths per million people. However, only the decrease recorded for the low HDI country group was statistically significant. These data show that the positive association between the implementation of health early warning systems and the reduction in mortality is not related to HDI level. Rather, it shows that countries with low HDI have had the biggest reduction in mortality since the 2000s, with mortality from floods and storms now similar to that in other HDI groups.

#### Indicator 2.3.3: rising sea levels, migration, and displacement—headline finding: in 2023, 157·3 million people were living less than 1 m above current sea levels

Global mean sea level increased by 0·20 m between 1901 and 2018 and is projected to rise 0·28–1·88 m by 2100 (relative to 1995–2014 levels), with major local variations.^22,226^ Sea level rise can lead to permanent inundation, episodic flooding, coastal erosion, saltwater intrusion, vector-borne and waterborne disease risk, and disrupted coastal livelihoods, with resulting adverse effects on physical and mental health.^227,228^ Using land elevation and population data, this indicator estimates that 157·3 million people were living less than 1 m above sea level in 2023 and were therefore at risk of exposure to rising sea level—up 11% from 2010.

Populations can adapt to sea level rise through coastal infrastructure, ecosystems (eg, mangroves and wetlands), land reclamation, managed realignment, living in elevated built structures, health early warning systems, improved health care, and diversified food, freshwater supplies, and livelihoods. Where in situ adaptation limits are reached, human mobility could be a response, including planned relocation, temporary or permanent migration, or forced displacement. However, some people might be unable, unwilling, or not permitted to move, becoming trapped.^37^ Mobility or immobility can lead to health benefits and risks in sites of origin, migration routes, and destinations. The risk–benefit balance largely depends on the policies and protective measures in place. The second part of this indicator, therefore, monitors the availability of policies on climate change, migration, and health.

As of December, 2023, 54 policies identified across 40 countries connected climate change and migration. Policies at all governance levels rarely acknowledged the scientific links (or lack thereof) between climate change, mobility or immobility, and health. Policies generally assumed that migration would occur due to in situ adaptation limits, without acknowledging immobile populations or their health risks. They predominantly highlighted the negative effects of migration, downplaying the potential positive effect of effective, climate-adapted health systems and of policies that support mobile and immobile populations and in situ adaptation. In parallel, if in situ adaptation limits are reached (panel 5), policies to ensure migration is available as a safe, desirable, health-supporting option will be urgently needed.

### Conclusion

There has been a persistent failure to adequately adapt to the rapidly growing health threats of climate change, and limits to adaptation are looming (panel 5). Only 61% of ATACH countries carried out a vulnerability and adaptation assessment (indicator 2.1.1), only 52% had HNAPs (indicator 2.1.2), and cities in low and medium HDI countries are lagging in assessing their climate change and health risks (indicator 2.1.3). These delays limit the capacity to implement effective, evidence-based health adaptation policies. The paucity of intersectoral collaboration, especially between meteorological and health institutions (indicator 2.2.1), further hinders adaptation efforts. Some effective adaptation measures are underused, including nature-based solutions (panel 4) such as urban greenspaces (indicator 2.2.3). Instead, people turn to maladaptive responses, with a growing proportion of households using air conditioning (indicator 2.2.2). Importantly, universal health coverage remains unattained, with 4·5 billion people (more than half the world’s population) still not covered by essential health services in 2021.^229^ This deficit leaves billions without access to the basic care needed to face health threats, including those of climate change—an aspect we will seek to monitor in future reports.

Despite these failures, delays, and shortcomings, there is some movement in the right direction. GCF funding for projects with potential health adaptation outcomes increased by 137% between 2021 and 2023 (indicator 2.2.4); more than two-thirds of countries self-reported high-to-very-high implementation of health emergency management capacities in 2023 (indicator 2.2.5); and since 1990, there has been a 46% decrease in vulnerability to severe *Aedes*-borne disease in low HDI countries thanks to health-care improvements (indicator 2.3.1). Additionally, the lethality of floods and storms has declined since the Paris Agreement entered into force, particularly in countries with climate-informed health early warning systems (indicator 2.3.2), and 70% of 279 public health institutions surveyed provide climate and health education, building capacity to protect health in the face of climate challenges—albeit largely in high and very high HDI countries (indicator 2.2.6).

The new 2-year UAE-Belém work programme will strengthen the UAE Framework for Global Climate Resilience^167^ by outlining and developing indicators for measuring progress towards adaptation targets, including health.^179^ Following the commitment of $1 billion to climate and health, improved metrics to monitor vulnerabilities, climate change impacts, and adaptation can help to inform funding allocations, policy decisions, and life-saving adaptation. Acknowledging this need, the *Lancet* Countdown will allocate increased resources towards refining its metrics, working with WHO and other partners to put the best available science at the service of improved climate change adaptation policies.

## Section 3: mitigation actions and health co-benefits

Despite 31 years of international climate negotiations, global emissions are nowhere near meeting the Paris Agreement goal of limiting heating to 1·5°C. Indeed, current policies and actions, if sustained, would put the world on track to a potentially catastrophic 2·7°C of heating.^11^ Efforts to reduce greenhouse gas emissions are, therefore, essential to protect the wellbeing, health, and survival of individuals in every country. Importantly, many such actions can also deliver direct health benefits in the short and long terms.^230^

This section provides an updated overview of progress in climate change mitigation and associated health outcomes. It examines progress towards mitigation in the energy and agriculture sector and the opportunities for promoting health through improved air quality and diet. A new indicator monitors tree cover loss, which draws a link between climate change, biodiversity, and health. Additionally, the last indicator monitors the emissions and associated health impacts from the health-care sector—an effort that will be crucial in tracking progress towards the health sector mitigation goals set out in WHO’s GPW14.

### 3.1 Energy use, energy generation, and health

A global transition offers the potential for major health benefits, including those resulting from improved air quality and energy access, safer employment opportunities, and reduced vulnerability of energy supply to uncertain geopolitics. The following indicators track energy system mitigation progress and related health impacts.

#### Indicator 3.1.1: energy systems and health—headline finding: global CO_2_ emissions from the energy system reached an all-time high in 2023, 1·1% above 2022 levels

With 67% of global greenhouse gas emissions from fossil fuel combustion,^231^ preventing the most dangerous climate change scenarios requires structural changes in the energy sector. In addition to emissions, the extraction and use of fossil fuels pose myriad health impacts throughout the fuels’ lifecycle (panel 6).

Drawing on data from the IEA to track mitigation in the energy sector, this indicator shows that, despite temporary changes during the COVID-19 pandemic, minimal progress has been made since the Paris Agreement entered into force in 2016.^231,261^ The proportion of fossil fuels in the global energy system increased for the first time in a decade during 2021, reaching 80·3% (up from 80·1% in 2020), and the carbon intensity of the energy sector has decreased by just 3·8% since 2016.^261^

Given the high greenhouse gas and air pollution emission intensity of coal, its phase-out is crucial to protect people’s health. However, the share of coal in the electricity system of low HDI countries increased from 0·5% in 2016 to 10·4% in 2021, and medium and high HDI countries maintained shares above 50%. Only very high HDI countries reduced this share, from 24·7% to 19·1%. These figures underscore the global inequities in the access to clean, healthy energy and the health trade-offs of an unjust transition as countries seek to meet the growing electricity demand.

Alongside increasing energy efficiency, a rapid transition to renewable energies is crucial to tackling climate change.^262^ The share of electricity generated from clean renewables reached a record high of 10·5% in 2021, almost doubling the share in 2016 (5·5%).^263^ In this period, the share grew from 6·7% to 11·6% in very high HDI countries, from 4·2% to 10·3% in high HDI countries, and from 4·6% to 8·2% in medium HDI countries. In low HDI countries, those most affected by energy poverty, the proportion grew from only 1·3% in 2016 to just 2·3% in 2021.^263^

#### Indicator 3.1.2: household energy use—headline finding: shares of harmful biomass energy use in homes have decreased minimally, from 32% in 2016 to 30% in 2021, remaining at around 92% in low HDI countries

Household energy use is a key determinant of health and is linked to economic development. However, almost 2·3 billion people still use dirty fuels and technologies for cooking.^264^ This indicator uses IEA data to track household energy use by fuel source. Between 2016 and 2021, per-capita household energy use increased by 3% globally. Meanwhile, mostly driven by medium and high HDI countries, the share of heavily polluting solid biomass decreased from 32% to 30%, and electricity use increased from 24% to 26%. However, low HDI countries saw almost no change in the share of solid biomass for domestic energy use, which has remained at around 92% since 2016. Indeed, although 15 million people gained access to electricity between 2022 and 2023, 745 million people still have no access to this essential service.^265^

The use of dirty fuels for cooking represents a substantial health hazard in lower HDI countries, exposing women and young children, in particular, to high levels of air pollution.^266^ Data from WHO to monitor progress against Sustainable Development Goal (SDG) 7 reveal that, globally, 60% of the rural population had access to so-called clean cooking fuels in 2023,^267^ compared with 77% of the urban population. Major disparities exist between countries, and only 13% of all low HDI countries had access to clean cooking fuels, contrasting with the near universal (98%) access observed in very high HDI countries. Importantly, for SDG 7, liquefied petroleum gas is considered a clean fuel;^267^ however, its NO_2_ emissions are still hazardous. In 2021, 35% of the global population used liquefied petroleum gas as the main fuel for cooking, with an average of 42% using this fuel in very high HDI countries, 56% in high HDI countries, 34% in medium HDI countries, and 7·4% in low HDI countries. These data underscore the opportunity for tackling energy poverty by increasing access to reliable, healthy, renewable energy, particularly in the most underserved countries.

#### Indicator 3.1.3: sustainable and healthy road transport—headline finding: from 2016 to 2021, the share of road transport from electricity increased by only 0·19 percentage points

Road transport contributes around 16% of global CO_2_ emissions.^268^ Transitioning to zero greenhouse gas emission transport systems is therefore crucial to tackle climate change. Shifting to electric vehicles while also reaching net zero greenhouse gas electricity supply is important in this transition, particularly when other limitations exist to the use of public transport and door-to-door transport modes are needed (eg, in the case of disabled people or those with mobility impairment).

The annual sale of electric cars increased from 0·7 million in 2016 to 17 million in 2024.^269^ However, global transport emissions almost returned to their pre-pandemic peak in 2022.^268^

This indicator, which uses IEA data, finds that the share of road transport energy supplied by electricity only increased from 0·09% in 2016 to 0·27% in 2021. Fossil fuels still accounted for 95·2% of all road transport energy in 2021, with biofuels supplying the remainder.

To maximise health gains, support a rapid reduction of emissions, and avoid the growing inequities that the unaffordability of electric vehicles can generate, a transition in transport systems is required to minimise the use of private vehicles. Instead, policies should favour affordable, accessible, and adequately available public transport systems with zero greenhouse gas emissions and safe active travel modes (eg, walking or cycling), which could also help reduce transport-related social exclusion. Such a shift would deliver substantial air pollution benefits, reduce inequities in access to transport services, and improve public health through increased physical activity.^270^

### 3.2 Air quality and health co-benefits

Exposure to air pollution increases the risk of respiratory and cardiovascular disease, cancer, diabetes, neurological disorders, and adverse pregnancy outcomes.^271^ Many major sources of greenhouse gas emissions also contribute to air pollution. The following indicators monitor the mortality associated with fuel-derived air pollution, to estimate the maximum potential health co-benefits that mitigation in the energy sector could deliver through improvements in air quality.

#### Indicator 3.2.1: mortality from ambient air pollution by sector—headline finding: deaths attributable to PM_2·5_ from fossil fuel combustion decreased 6·9% from 2·25 million in 2016 to 2·09 million in 2021

In the transition to a net zero future, countries can reap major public health benefits from prioritising interventions that reduce exposure to air pollution. This indicator combines the well established greenhouse gas–air pollution interactions and synergies (or GAINS) atmospheric model with information about activity in emitting sectors to produce validated estimates of anthropogenic PM_2·5_ air pollution and estimate the associated mortality. In an improvement from previous years, this indicator incorporates new concentration–response functions, published in 2022, resulting in more attributable deaths than in previous years.^272^

In 2021, there were 8·4 million deaths attributable to PM_2·5_, including both anthropogenic and natural sources of pollution. From 2016 to 2021, global average exposure to PM_2·5_ from all anthropogenic sources fell by 4·6%, driven predominantly by high and very high HDI countries. However, average global anthropogenic PM_2·5_ exposure remained over four times higher than the 5 μg/m^3^ WHO guideline threshold. Combined with demographic changes, deaths attributable to anthropogenic PM_2·5_ increased by 4·8%, reaching at least 6·4 million in 2021. The extensively validated model used in this indicator suggests that exposure to fossil fuel-derived outdoor PM_2·5_ pollution contributed to 2·09 million of these deaths, with coal burning accounting for nearly 980 000 deaths.^273^ Importantly, deaths attributable to fossil fuel-derived PM_2·5_ have fallen by an estimated 156 000 (6·9%) since 2016 (2·25 million). Of this reduction, 59% was due to reduced coal-related pollution, mostly in high and very high HDI countries. However, biomass burning caused 1·24 million deaths in 2021, an increase of 135 000 from 2016.

**Figure 7:**
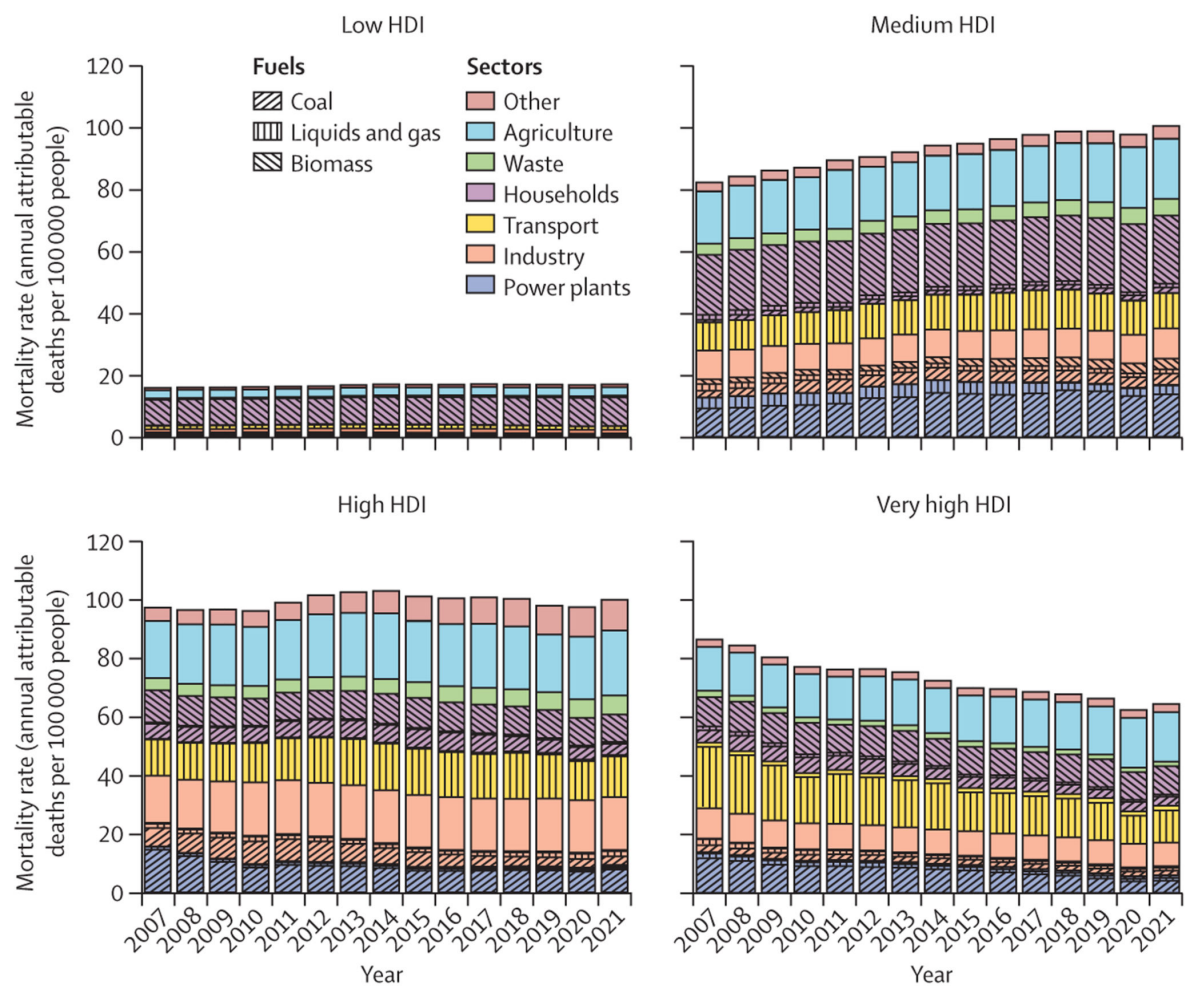
Annual mortality rates attributable to PM_2·5_ exposure from 2007–21, by fuel, sector, and HDI country level HDI=Human Development Index.

There were marked differences in progress between HDI groups. Although the mortality rate (deaths per 100 000 individuals) attributable to fossil fuels decreased by 22·8% in very high HDI countries, 17·5% in low HDI countries, and 11·7% in high HDI countries, they only declined 3·7% in medium HDI countries. Mortality rate remained highest in the medium HDI country group (36 deaths per 100 000 individuals) in 2021, as it did in 2016 (38 deaths per 100 000). Meanwhile, biomass-related mortality rate decreased by 1·6% in very high HDI countries but increased by 4·3%, 10·5%, and 8·9% across low, medium, and high HDI countries, respectively. The death rate from biomass remains highest in medium HDI countries (26 deaths per 100 000 individuals; figure 7).

#### Indicator 3.2.2 household air pollution—headline finding: indoor PM_2·5_ derived from the burning of solid household fuels resulted in 2·3 million deaths across 65 countries in 2020

Despite efforts to increase access to clean energy under SDG 7,^274^ 2·4 billion people worldwide still use dirty fuels and inefficient technologies to meet their household energy needs, leading to high concentrations of indoor air pollution and to other health harms from energy poverty.^267^ This indicator uses a Bayesian hierarchical model to estimate the deaths attributable to PM_2·5_ household air pollution by source of emission in 65 countries (42% of which are low HDI, 26% are medium HDI, 28% are high HID, and 5% are very high HDI).^17,275–278^

**Figure 8:**
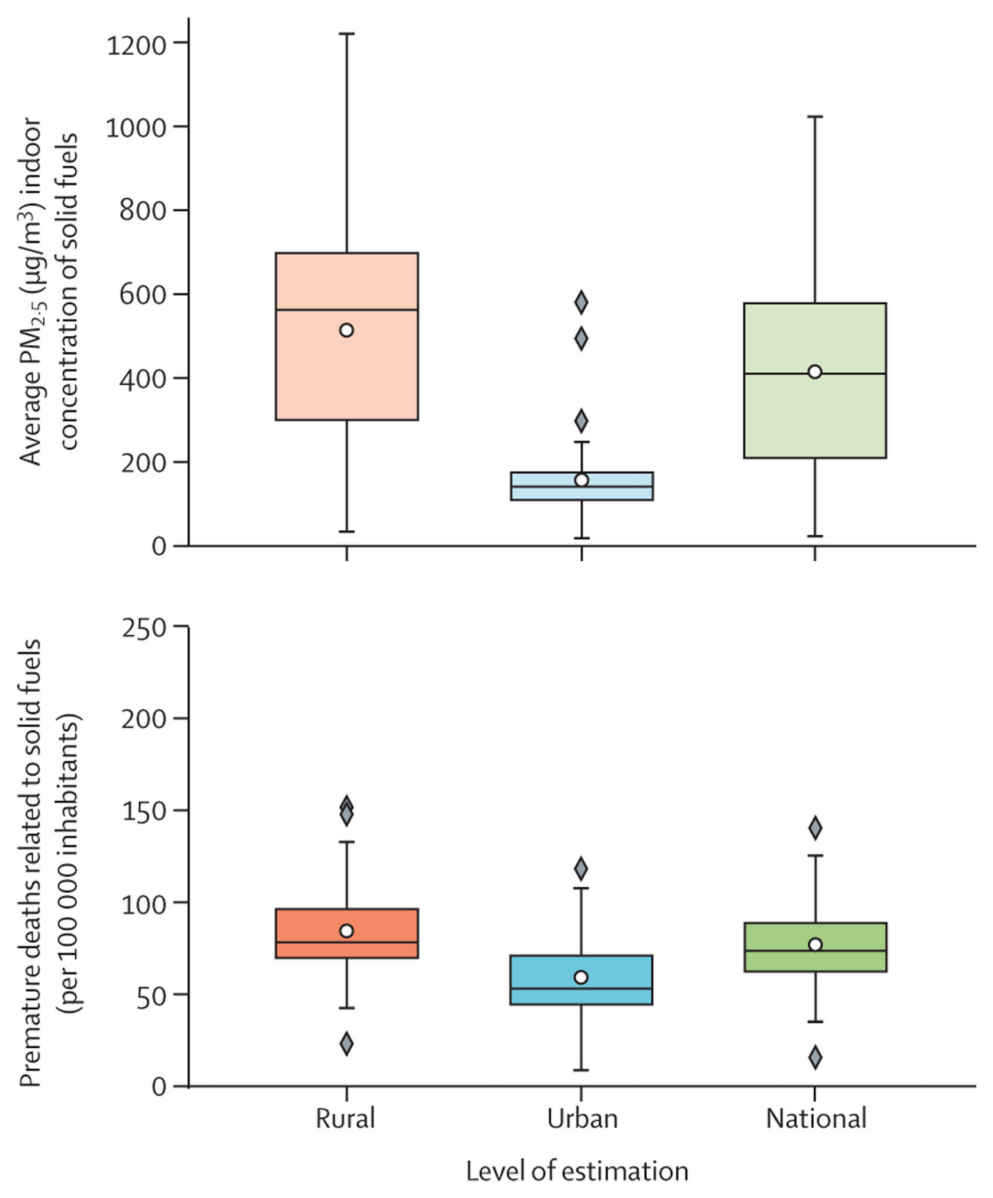
Estimated annual weighted average household indoor PM_2·5_ concentrations (in µg/m^3^) at the urban, rural, and national levels and the related premature death rate (per 100 000 population) attributable to polluting solid fuels

In 2020, household use of solid fuels for cooking and heating led to an estimated national-level annual average indoor PM_2·5_ concentration of 412 µg/m^3^ (95% CI 353–471), almost 12 times the WHO interim target of 35 μg/m^3^ and 82 times the maximum guideline concentration proposed by WHO (15 μg/m^3^).^279^ Concentrations were higher in rural households, with an average of 514 µg/m^3^ (95% CI 446–582), 3·4 times the urban household average (149 µg/m^3^, 95% CI 126–173; figure 8).^277,278^ This air pollution was responsible, on average, for 78 deaths (95% CI 72–84) per 100 000 inhabitants, with a rural average of 84 (78–91) and an urban average of 60 (54–66) per 100 000 inhabitants. In the 65 countries studied, indoor PM_2·5_ from solid household fuels was responsible for roughly 2.3 million deaths in 2020 alone—deaths that could be avoided by transitioning to renewable energy sources.

### 3.3 Food, agriculture, and health co-benefits

Food systems account for up to 30% of global greenhouse gas emissions.^280^ This set of indicators tracks progress towards transitioning to healthy, plant-forward diets and delivering the associated benefits to public health.

#### Indicator 3.3.1: emissions from agricultural production and consumption—headline finding: global agricultural emissions increased by 2·9% from 2016 to 2021; red meat and dairy contributed to 56% of agricultural emissions in 2021

Actions in the agricultural sector—a major contributor to greenhouse gas emissions and other environmental degradation—are essential to meet the goals of the Paris Agreement. This indicator combines data from the production and trade of agricultural products with their greenhouse gas emission intensities to estimate greenhouse gas emissions from agricultural products available in each country, excluding those from induced deforestation.

Globally, consumption-based agricultural emissions grew by 2·9% from 2016 to 2021, with 56% of 2021 emissions driven by the consumption of red meat and dairy. Per-person emissions were similar in low and medium HDI countries (0·69 and 0·76 tonnes CO_2_ equivalent [tCO_2_e] per person, respectively, in 2021). Per-person emissions in high HDI countries have increased since 1990, reaching 0·95 tCO_2_e per person in 2021, albeit with a slower pace of increase since 2016. In contrast, in very high HDI countries, consumption-based emissions have decreased by 6·2% from 2016 to 2021. However, these countries were still the biggest contributors to agricultural emissions, at 1·02 tCO_2_e per person in 2021, 60% of which derived from red meat and dairy consumption.

#### Indicator 3.3.2: diet and health co-benefits—headline finding: between 2016 and 2021, the burden of diet-related diseases increased from 141 deaths to 144 deaths per 100 000 people (+3%), including increases from 14 deaths to 16 deaths per 100 000 attributable to red meat intake (+9%)

Imbalanced diets with excessive intake of red and processed meat and low intake of high-quality plant-based foods are not only major drivers of greenhouse gas emissions (indicator 3.3.1) but also increase health risks.^381–384^ This indicator monitors the deaths attributable to unhealthy diets and inadequate caloric consumption that could be avoided through the transition to diets associated with lower greenhouse gas emissions, through a comparative risk assessment.^385,386^ Between 2016 and 2021, yearly diet-related deaths increased by 830 000 (+8%), from 10·4 to 11·2 million, an increase from 141 deaths to 144 deaths per 100 000 people. These figures included 315 000 additional deaths from low intake of whole grains, vegetables, and legumes, 150 000 from high red meat intake, and 70 000 from high dairy intake in 2021, compared with 2016. The increases in diet-related disease burden were greatest in countries with very high HDI (+13 deaths per 100 000 people), followed by countries with high HDI (+5 deaths per 100 000 people), medium HDI (+2 deaths per 100 000 people), and low HDI (+1 death per 100 000 people). Many of these deaths could be saved through dedicated dietary policies, which could play a major role in tackling climate change while building healthier, more resilient populations.^387–389^

### 3.4 Tree cover loss

#### Headline finding: between 2016 and 2022, the world lost almost 182 million hectares of forest cover, 5% of global tree cover

Trees and forests are crucial carbon sinks and biodiversity reservoirs. They can also be a source of food, medicine, and knowledge, especially for Indigenous peoples.^290^ Poor tree cover and inadequate forest conservation exacerbate climate change and increase the risk of forest fires, zoonotic diseases, and allergies.^290,291^ Understanding the patterns of tree cover loss is vital for supporting climate strategies and public health (panel 4).^290–292^ This indicator uses satellite data to track the loss of vegetation 5 m or taller in areas with at least 30% tree cover density. As such, it covers the loss of forests, open woodlands, trees in agricultural settings and urban areas, and small patches of trees.^293,294^

**Figure 9:**
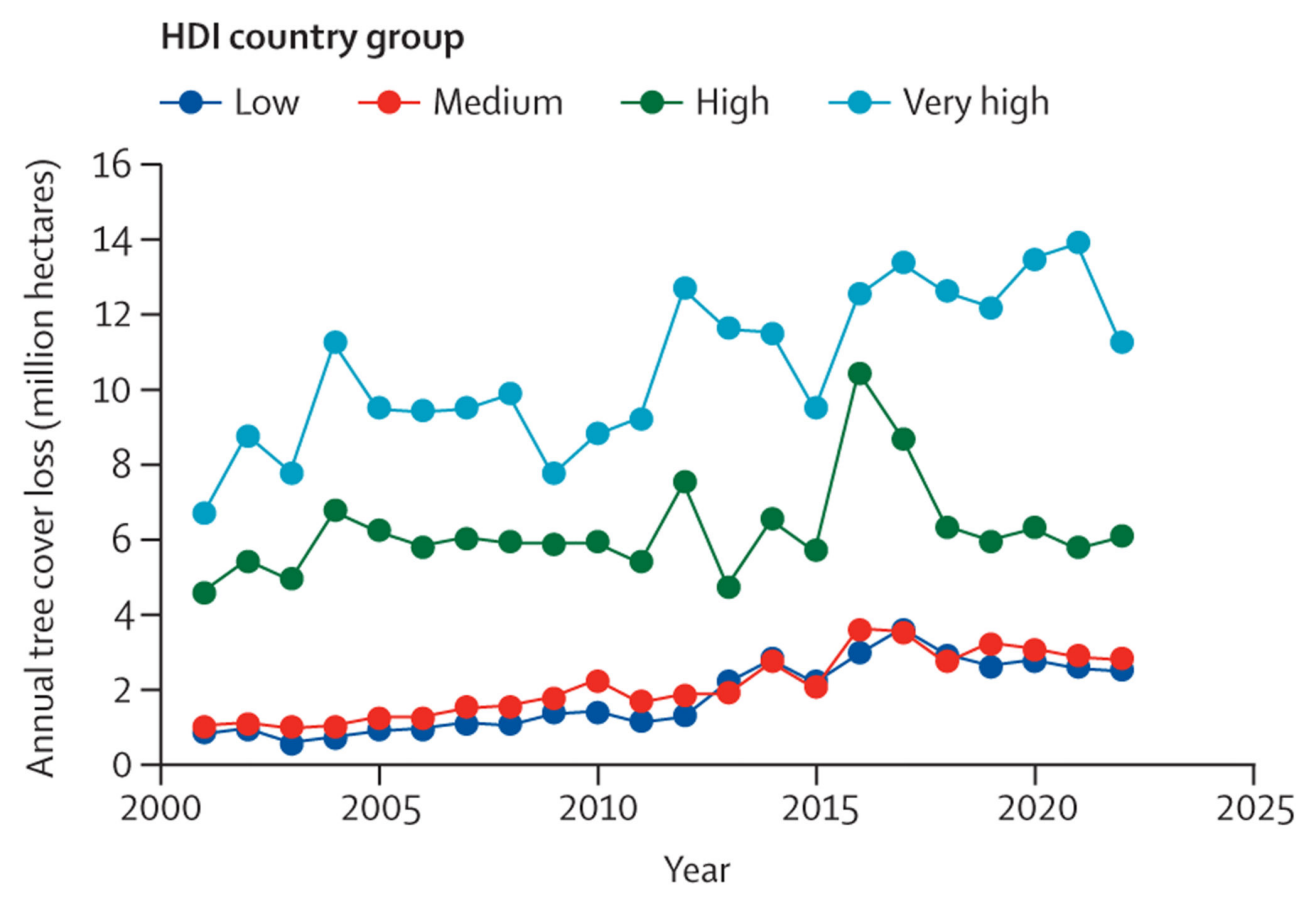
Annual global loss of tree cover from 2001 to 2022, stratified by HDI group HDI=Human Development Index.

Between 2001 and 2022, the world lost approximately 459 384 000 hectares (11·5%) of its tree cover. Of these losses, 40% (181 847 000 hectares [5%] of global tree cover) have occurred since 2016, the year the Paris Agreement entered into force. Tree cover loss was highest in very high HDI countries, which have lost almost 90 million hectares since 2016. Three of the four countries that have lost the most tree cover since 2016 are classified as very high HDI: Russia (35 837 000 hectares), the USA (14 993 000 hectares), and Canada (14 983 000 hectares). Brazil, a high HDI country, had the second-highest loss of tree cover globally in this period (25 136 000 hectares). Despite increasing, tree cover loss was lower in low and medium HDI countries, at 20 and 22 million hectares lost, respectively, since 2016 (figure 9). Forestry activities were the cause of 30% of the global tree cover loss between 2016 and 2022, followed by agriculture (27%), wildfires (22%), and commodity-driven deforestation (20%).

The continuous and increasing tree cover loss underscores the urgent need for concerted global and regional efforts to support tree conservation and the role of forests in promoting health and addressing climate change. With 36% of the world’s intact forests within Indigenous peoples’ lands, ensuring Indigenous peoples’ involvement in forest protection efforts is of crucial importance.^295^

### 3.5 Health-care sector emissions and harms

#### Headline finding: in 2021, health-care sector-related greenhouse gas emissions were 9·5% higher than in 2020 and 36% higher than in 2016, and associated air pollution contributed to 4·6 million disability-adjusted life years (DALYs) in 2021

Quality health care requires the use of energy, goods, services, and infrastructure, which consumes resources and currently contributes to greenhouse gas emissions and air pollution. Delivering low greenhouse gas-emitting and sustainable health systems is essential in a world that meets the goals of the Paris Agreement and enables a healthy future. Under WHO’s ATACH, 74 countries committed to developing health systems with net zero emissions or sustainable, low greenhouse gas emissions,^180^ and 151 countries signed the UAE Declaration on Climate and Health,^15^ committing to promoting steps to curb emissions in the health sector.

This indicator combines an environmentally extended multiregion input–output model with national health-care expenditure data to develop the world’s most comprehensive and regularly updated monitoring system on health-care sector greenhouse gas emissions. Health-care-related greenhouse gas emissions contributed to 4*·*6% of global greenhouse gas emissions in 2021, up by nearly 10% from 2020, largely due to COVID-19 pandemic-related shifts in patterns of health-care demand—a 36% increase in health-care-related greenhouse gas emissions since 2016. In 2021, health systems in high and very high HDI countries contributed to 91% of global health-care emissions, with average per-capita emissions in very high HDI countries 2·6 times higher than in medium HDI countries and 8·4 times higher than in low HDI countries. Air pollution (PM_25_ and ozone) from health-care operations and supply chains is estimated to have caused 4*·*6 million DALYs in 2021, a jump of nearly 20% from 2020.

**Figure 10:**
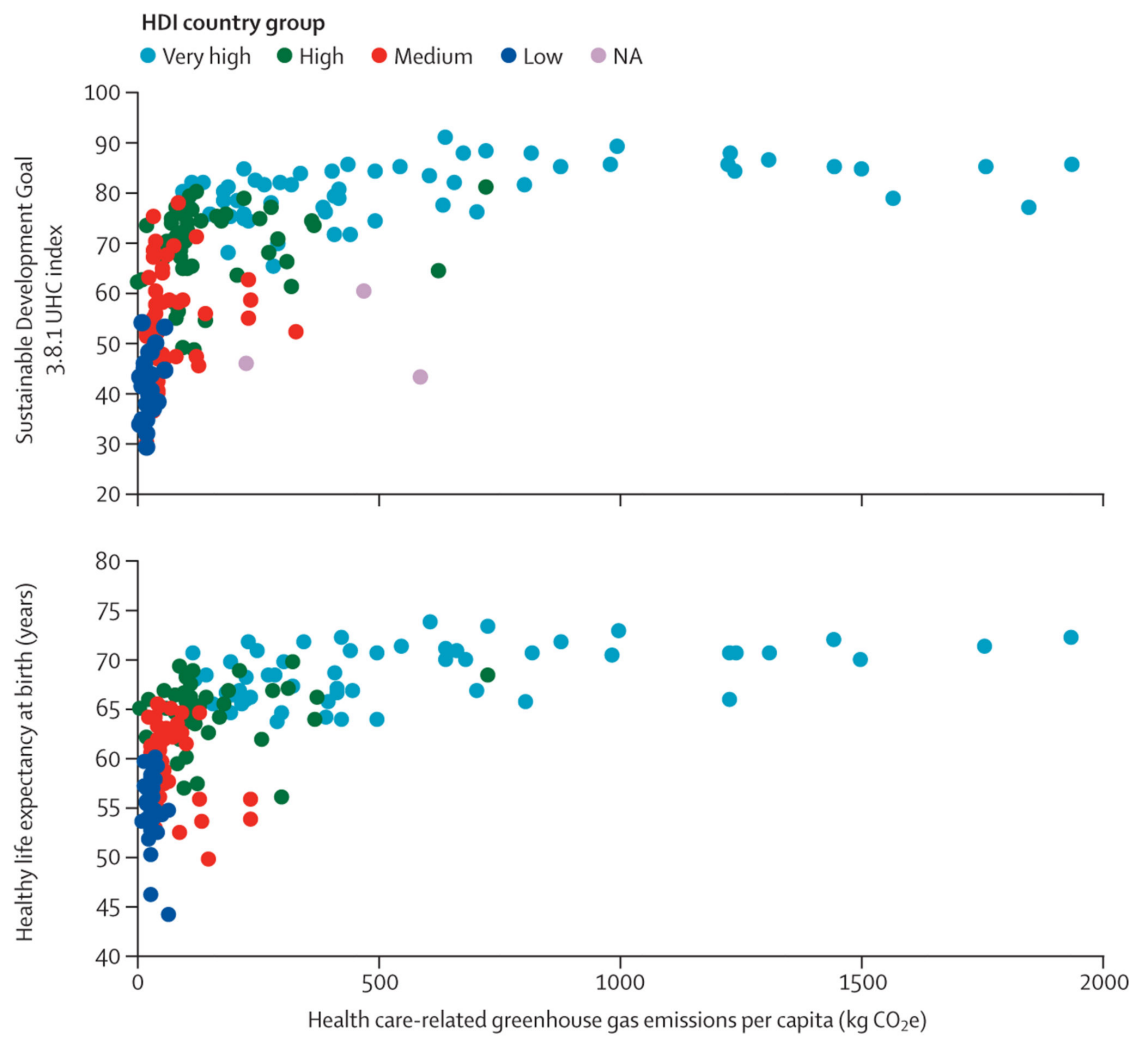
UHC index scores and healthy life expectancy at birth in relation to greenhouse gas emissions, by HDI National per-capita greenhouse gas emissions from the health-care sector in 2021 versus the UHC index score for 2021 (top) and national per-capita greenhouse gas emissions from the health-care sector in 2021 versus healthy life expectancy at birth in 2019 (bottom; countries without an HDI score are not included because they do not have values for healthy life expectancy at birth); six countries with extreme values (>2000 kg CO_2_e) were excluded. CO_2_e=CO_2_ equivalent. HDI=Human Development Index. NA=not available. UHC=universal health coverage.

As efforts to increase access to quality health care and achieve universal health coverage increase, so will the use of goods and services within the health sector and their associated greenhouse gas emissions. In a context in which health systems around the world have not yet achieved carbon neutrality, universal health coverage index scores correlate with health care-related greenhouse gas emissions, but only up to approximately 400 kg CO_2_e per capita. Similarly, higher greenhouse gas emissions are correlated with higher healthy life expectancy at birth up to approximately 400 kg CO_2_e per capita. Figure 10 shows these plateaus in both universal health coverage and healthy life expectancy at birth, highlighting that accessible high-quality health care can be delivered without high carbon intensity above this threshold. Reaching net zero greenhouse gas emission health care while working towards improving outcomes and achieving universal health coverage will require continuous and meaningful improvements to the environmental performance of health-care facilities, operations, energy use, and supply chains, as well as appropriate care delivery.

### Conclusion

Progress towards meeting the Paris Agreement goals has been concerningly inadequate. Persistent failure to implement the necessary structural changes has pushed emissions to their highest level yet, with fossil fuels accounting for 67% of greenhouse gas emissions in 2022. Moreover, the small amount of progress to date has been uneven and is exacerbating global health inequities, and the inaction in mitigation and associated risks and exposures has resulted in millions of avoidable deaths each year. Since the Paris Agreement entered into force, only very high HDI countries have reduced their coal use, and coal-related air pollution caused 977 000 deaths in 2021 (indicators 3.1.1 and 3.2.1). Global road transport emissions have nearly rebounded to pre-pandemic levels, and global agricultural emissions have risen by 2*·*9% since 2016, with red meat and dairy accounting for 56% of these emissions (indicators 3.1.3 and 3.3.1). The increase in meat and dairy intake pushed diet-related deaths up by 830 000 between 2016 and 2021 (indicator 3.3.2). Health-care sector emissions rose by nearly 10% between 2020 and 2021 (indicator 3.5). In 2021, health care accounted for 4*·*6% of global emissions, and associated PM_25_ and ozone pollution contributed to the loss of 4·6 million DALYs (indicator 3.5).

These data unequivocally show the health imperative for concerted and equity-driven efforts at the national and international levels to implement structural changes in energy systems, transportation, agriculture, diets, and health-care systems. Such changes will hold the key to achieving a sustainable and healthy future for all.

## Section 4: economics and finance

Climate change is profoundly affecting the global economy. Recent data suggest that the world economy is on track to an income reduction of 11–29% by 2050, threatening the social and economic systems on which human health and wellbeing often depend.^296,297^ The damages expected within the next 26 years vastly outweigh the mitigation costs required to limit global heating to 2°C (by a factor of six, according to a recent study).^298^ The health impacts of climate change contribute to economic losses, increase health system costs, limit labour productivity, and threaten important economic sectors, including tourism.^17,299,300^ The resulting deterioration of the socioeconomic conditions that support good health can aggravate health impacts and overburden the most vulnerable countries, widening global inequities.

A swift and just transition to a net zero greenhouse gas economy is, therefore, crucial to limit economic and health-related losses and damages but requires profound structural and economic changes and substantial capital investment.^297^ Years of delay in delivering the $100 billion promised annually to support the most vulnerable countries in a just transition have hampered progress and further exacerbated global inequities.^296^

COP28 saw the long-awaited operationalisation of the Loss and Damage fund, an important milestone for a just transition;^301^ additionally, $1 billion was committed to climate change and health.^302,303^ Details on how these funds will be disbursed and who will contribute to the Loss and Damage fund will be negotiated at COP29. COP29 will also be the stage for the negotiation of the New Collective Quantified Goal on Climate Finance, setting a new financial goal for supporting countries classified as developing in the context of UNFCCC, in excess of $100 billion.^297^

This section is presented with a new structure, to better reflect the different dimensions of these policy processes. The first set of indicators tracks the economic losses and damages associated with the health impacts of climate change; the second set monitors the extent to which countries are delivering a just, health-promoting restructuring of their economies; and the third set monitors the shift of financial systems away from fossil fuels and towards a health-supporting economy. This year, two new indicators highlight countries’ preparedness for such a transition and call out potential losses through stranded assets.

### 4.1 The economic impact of climate change and its mitigation

As the impacts of climate change grow, so do the associated economic losses. This set of indicators monitors the economic losses resulting from the health impacts of climate change.

#### Indicator 4.1.1: economic losses due to weather-related extreme events—headline finding: in 2023, weather-related extreme events caused $212 billion in global economic losses

In addition to direct health effects, extreme weather events can also damage health centres, impede access to health services, and cause economic losses that can undermine the social determinants of health. This indicator uses data provided by Swiss Re (a synthesis of these data and description of the methodology are provided in Swiss Re’s sigma explorer tool) to track the economic losses from extreme weather events.

From 2010–14 to 2019–23, average annual economic losses induced by weather-related extreme events increased by 23% in real terms, to $227 billion. The percentage of global losses that were uninsured fell from 67% to 55%. Although 60·5% of losses in very high HDI countries in 2023 were insured, none of the losses in low HDI countries and only 18·5% and 16·4% of those in medium and high HDI countries, respectively, were insured. As a result, the economic burden of climate change currently falls disproportionately on lower HDI countries, with replacement costs falling directly on those affected, or going unreplaced.

#### Indicator 4.1.2: costs of heat-related mortality—headline finding: the average annual monetised value of global heat-related mortality for 2019–2023 was $199 billion, an increase of 179% from 2000–04

In 2023, record-breaking high temperatures resulted in unprecedented heat-related mortality and associated economic losses globally. This indicator calculates the monetised value of heat-related deaths by combining data from indicator 1.1.5 with the value of a statistical life year. The global monetised value of heat-related deaths of people older than 65 years rose to $240 billion in 2023—the highest level in the observed period and 236% higher than the 2000–04 annual average. The average annual monetised value during 2019–23 was $199 billion, 179% higher than in 2000–04. Low HDI countries saw the greatest increase from 2000–04 to 2019–23, at 240%, with increases of 194% in medium HDI countries, 228% in high HDI countries, and 139% in very high HDI countries.

#### Indicator 4.1.3: loss of earnings from heat-related labour capacity reduction—headline finding: in 2023, the global potential income loss from labour capacity reduction due to extreme heat reached a record high of $835 billion

The loss of labour capacity due to heat exposure (indicator 1.1.3) leads to income losses, potentially harming the health and wellbeing of workers, their families, communities, and national economies. This indicator combines data from indicator 1.1.3 with the International Labour Organization’s wage data to quantify the potential loss of earnings resulting from heat-related loss of labour capacity.^304^

**Figure 11:**
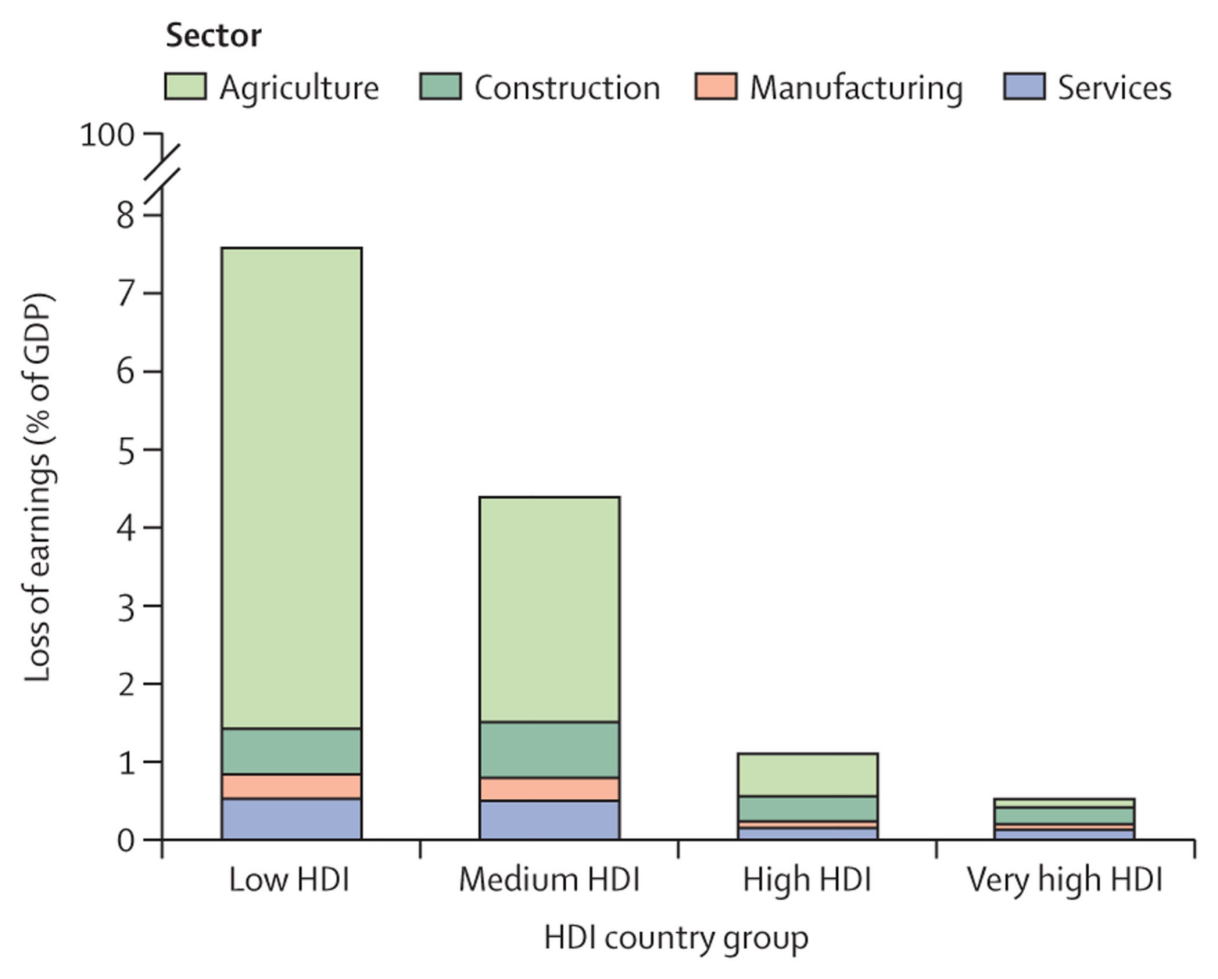
Average potential loss of earnings in 2023 of countries in each HDI group as a result of potential labour loss due to heat exposure Losses are presented as share of GDP and sector of employment. GDP=gross domestic product. HDI=Human Development Index.

In 2023, the global potential loss of income reached a record high of $835 billion, equivalent to 0·82% of gross world product. Average potential income lost was highest in low and medium HDI countries, equivalent to 7·6% of the GDP of low HDI countries (increased from 6·1% in 2022) and 4·4% of the GDP of medium HDI countries (increased from 3·8% in 2022; figure 11). Losses in the agricultural sector accounted for 37% of all global losses, with 31% in construction. Agricultural workers in low and medium HDI countries are often among the world’s poorest and least resilient to major economic shocks,^305–307^ and agricultural losses accounted for an average of 81% of the potential losses in low HDI countries and 65% in medium HDI countries (figure 11).

#### Indicator 4.1.4: costs of the health impacts of air pollution—headline finding: the monetised value of premature mortality due to air pollution reached a record high in 2021, amounting to $4·95 trillion, 14% above 2016 levels

The millions of deaths associated with anthropogenic PM_2·5_ pollution annually (indicator 3.2.1) result in economic losses, which could be reduced through robust mitigation. Building on indicator 3.2.1, this indicator places a monetised value on the years of lost life from exposure to anthropogenic ambient PM_2·5_. This value reached a record high of $4·95 trillion in 2021, a 14% increase since the Paris Agreement entered into force, and a 22·6% increase since 2007. The monetised values have risen by 1·3%, 21·4%, and 33·3% in low, medium, and high HDI countries, respectively, since 2016, but have fallen by 0·3% in very high HDI countries.

### 4.2 The transition to net zero carbon, health-supporting economies

Fossil fuels are deeply entrenched in the global economy. Consequently, a safe transition to a healthy, net zero future requires countries to prepare for a profound economic transformation while avoiding unintended harms. This set of indicators monitors the progress countries are making towards such transformation, introducing two new indicators tracking country preparedness for net zero and the impact on the value of coal-power assets becoming stranded in the transition to a healthy future.

#### Indicator 4.2.1: employment in low-carbon and high-carbon industries—headline finding: global direct employment in fossil fuel extraction increased by 0·4% in 2022 to 11·8 million employees, and in the same year, direct and indirect employment in renewable energy grew by 8·1%, to 13·7 million employees

Health risks are generally greater for employees in the fossil fuel sector than for those in the renewable energy sector (panel 6).^308,309^ Thus, the renewable energy sector presents new and healthier local job opportunities. Using data from the International Renewable Energy Agency and IBISWorld, this indicator compares employment in renewable energy and fossil fuel extraction.

Globally, the renewable energy industry employed 13·7 million people directly or indirectly in 2022, marking a 35·6% increase since 2016 (+3·6 million jobs), and an 8·1% increase from 2021 levels. Of the total employees in the renewables sector, 62% were in Asia (40% in China). Although direct employment in fossil fuel extraction increased by 0·4% from 2021 to 2022, mostly in response to the disruption of fossil fuel supplies following the invasion of Ukraine, it has declined by 10·3% (–1·35 million jobs) since 2016, suggesting the response to the Paris Agreement might have influenced employment trends.

#### Indicator 4.2.2: compatibility of fossil fuel company strategies with the Paris Agreement—headline finding: as of March, 2024, the strategies of the 114 largest oil and gas companies have put them on track to exceed their share of greenhouse gas emissions consistent with limiting global heating to 1·5°C by 189% in 2040, up from the 173% excess projected in March, 2023

To limit global heating and avoid the most harmful effects of climate change, oil and gas emissions need to be reduced dramatically. This indicator assesses the alignment of oil and gas companies’ production strategies with the Paris Agreement goals, using the Rystad Energy database of projected production based on current commercial activities, regardless of pledges. In an improvement from last year, the number of companies covered has been increased to 114, now covering 80% of all production projected by 2040. Projected emissions are compared with the IEA’s Net Zero Emissions pathway compliant with 1·5°C of heating, assuming current market shares.^310^

As of March, 2024, the strategies of the world’s largest 114 oil and gas companies indicate that they are on a trajectory to exceed the share of greenhouse gas emissions compatible with 1·5°C by 59% in 2030 (up from 43% in November, 2016, and 52% in March, 2023) and 189% in 2040 (up from 120% in 2016 and 173% in 2023), on average. The strategies of 33 of these companies put them on track to exceeding their 1·5°C-compatible share of emissions by 300% in 2040. Eight of the largest nine companies are state-owned national oil and gas companies, which, together, are projected to generate 30·2% of global production in 2040, exceeding their 1·5°C-compatible share by 226%.

**Figure 12:**
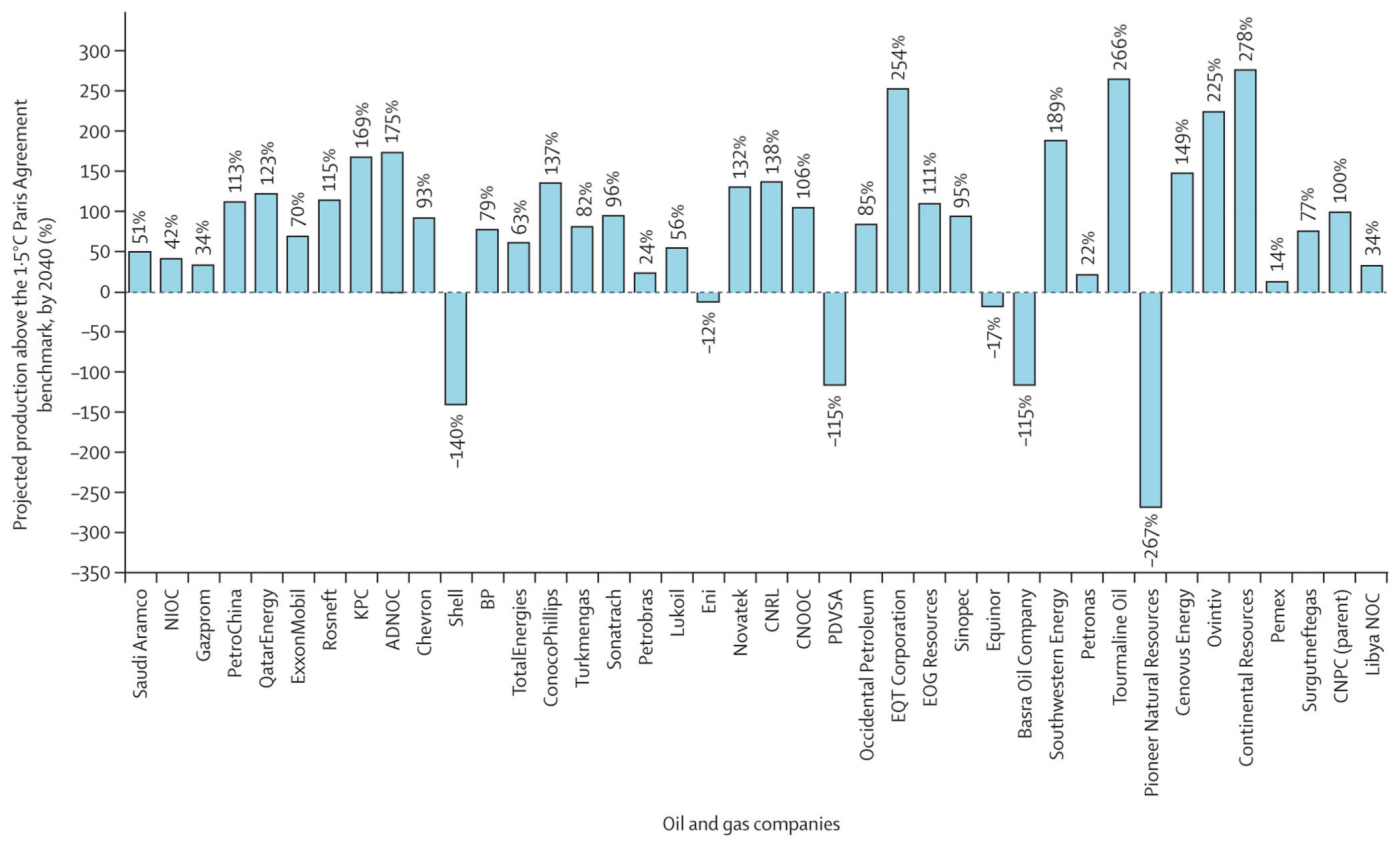
Difference in excess oil and gas production projected in 2040 based on shares compatible with 1·5°C of warming, comparing company strategies and activities in November, 2016, with those in March, 2024 Only the 40 largest oil and gas companies are shown, ranked in order of projected 2040 production (the largest on the left-hand side). ADNOC=Abu Dhabi National Oil Company. CNOOC=China National Offshore Oil Corporation. CNPC=China National Petroleum Corporation. CNRL=Canadian Natural Resources Limited. KPC=Kuwait Petroleum Company. NIOC= National Iranian Oil Company. NOC=national oil company. PDVSA=Petróleos de Venezuela SA.

Of the 40 oil and gas companies with the largest production projected by 2040, 34 (85%) have further increased their 2040 excess production since November, 2016, when the Paris Agreement went into effect (figure 12), and for 16 of these companies, this increase was greater than 100%.

#### Indicator 4.2.3: stranded coal assets from the energy transition—headline finding: the cumulative value of current assets in the global coal-fired power generation sector projected to be stranded between 2025 and 2034 will reach $164·5 billion

On a path that supports a healthy future and does not exceed the 1·5°C goal of the Paris Agreement,^311,312^ many of today’s fossil fuel assets must cease operating. Cessation must often occur well before the assets’ economic life ends, thus stranding the remaining capital investment. Continuing to invest in fossil fuels, therefore, not only hampers mitigation efforts and causes millions of deaths each year from exposure to air pollution but also harms the economy by increasing the economic value of stranded assets.^313,314^

The phase-out of coal is particularly important in the transition to a healthy future, not only because of its high greenhouse gas intensity but also because of the nearly 980 000 deaths associated with coal combustion annually (indicator 3.2.1). Using data from Global Energy Monitor on nearly 14 000 coal-fired units, this indicator tracks the annual value and spatial distribution of current coal-fired power generation assets that would be stranded under the carbon allowances limits of the 1·5°C goal. Carbon allowance limits for 2019–2100 are calculated on the basis of fairness principles of historical responsibility, capability to pay, and equal per capita convergence.^315–318^

The value of current coal-fired power generation assets that will be stranded in a path to 1·5°C of heating is expected to reach $15·6 billion in 2030, assuming the annual hours of use of each unit remain unchanged. Of these assets, 26·8% are based in very high HDI countries, with 59·0%, 13·9%, and 0·2% in high, medium, and low HDI countries, respectively. The total cumulative economic value that would be lost between 2025 and 2034 is expected to reach $164·5 billion. Of these assets, 32% are based in very high HDI countries, with 54·4%, 13·1%, and 0·5% in high, medium, and low HDI countries, respectively. These losses will be even higher if investments in coal power capacity continue. These results underline the importance of policy makers refraining from opening further coal-fired power plant to limit future stranding losses. In future years, this indicator will track progress towards transitioning to a healthy, sustainable economy by monitoring the value of current assets that will become stranded in a 1·5°C trajectory, as investments in fossil fuel assets change.

#### Indicator 4.2.4: country preparedness for the transition to net zero—headline finding: in 2023, all low HDI countries had transition preparedness scores below the global average, whereas 93% of very high HDI countries had scores above average

The transition towards a net zero greenhouse gas economy is essential for a healthy future. However, this transition requires major structural changes, which could yield both profound health benefits and unintended harms. Countries reliant on fossil fuel exports or with emissions-intensive energy production, manufacturing, transportation, and construction, with a large portion of the labour force employed in such activities and high social inequality, face high transition risks. This indicator assesses countries’ transition risk through a complex index incorporating 25 subindicators (appendix pp 246–251) that monitor institutional performance (eg, absence of political violence and terrorism and governmental effectiveness), economic situation (eg, macroeconomic stability, availability of finance for the transition, and gross national income per capita), and societal (eg, proportion of labour force in highly exposed sectors and human capital) and technological factors (eg, technology absorption and carbon intensity of manufacturing), weighted to derive a final preparedness score ranging from 0 and 1.

**Figure 13:**
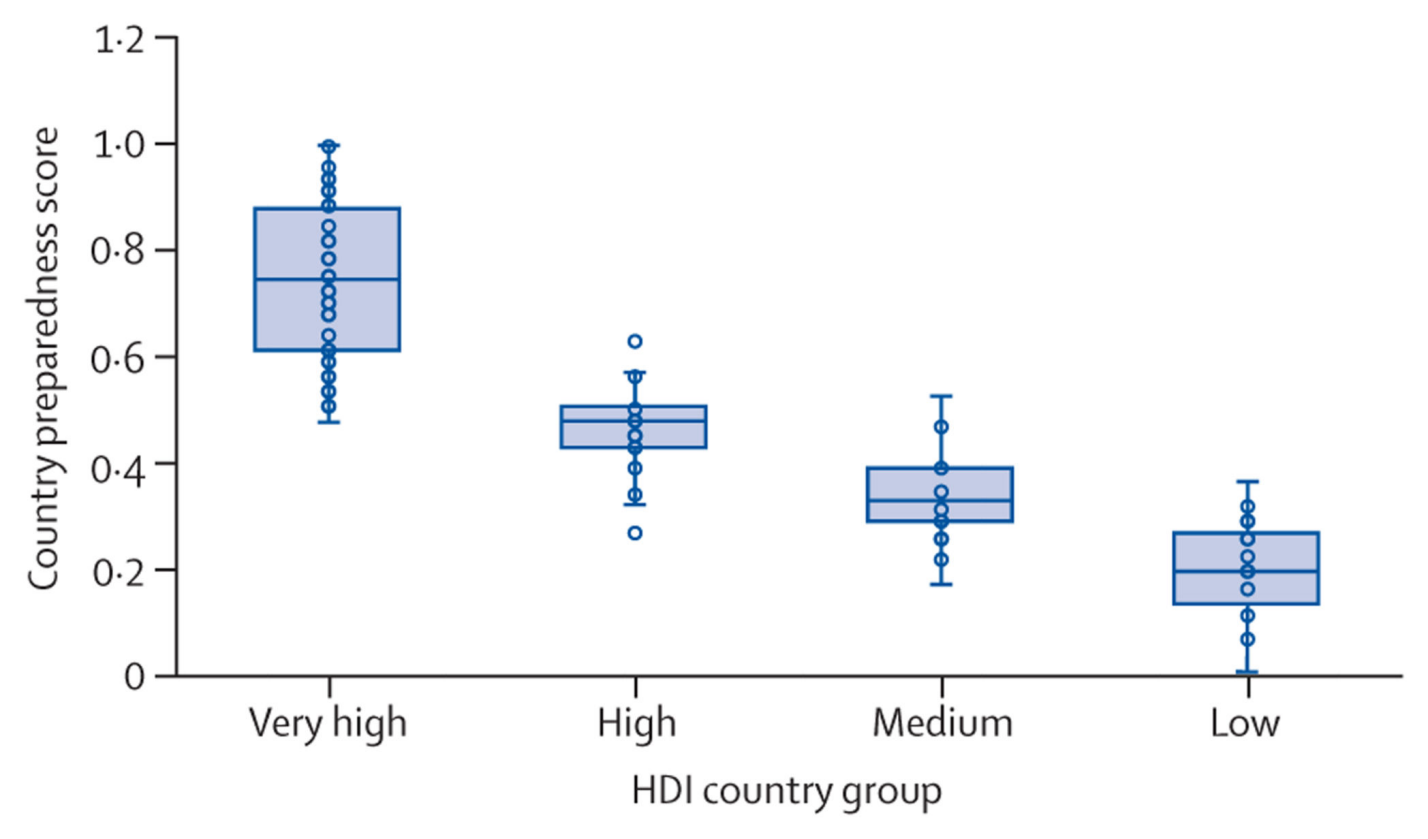
2023 preparedness scores for transition to net zero, by HDI group HDI=Human Development Index.

There is a strong correlation between countries’ transition preparedness and their HDI (figure 13). The global average preparedness score in 2023 was 0·52. Countries with a very high HDI had an average preparedness score of 0·74, whereas those with medium and low HDI scored 0·35 and 0·20, respectively, on average. All countries with a low HDI, 96% of those with a medium HDI, and 84% of those with a high HDI had scores below the global average, whereas 93% of very high HDI countries had scores above this value.

These findings underscore the inequalities in preparedness among economies and human systems for transitioning to a healthy, net zero greenhouse gas future, exposing people in the most vulnerable countries to substantial risks. Supporting countries in their transition to net zero is essential for ensuring a just transition that minimises unintended consequences.

#### Indicator 4.2.5: production-based and consumption-based attribution of CO_2_ and PM_2·5_ emissions—headline finding: the very high HDI country group remained the only group with higher consumption-based emissions than production-based emissions for both CO_2_ and PM^2·5^ in 2022, with differences accounting for 3·7% and 6·1% of global total emissions, respectively

Due to international trade, the consumption of imported goods and services in one country can contribute to greenhouse gas emissions and air pollution in foreign producing countries. This indicator uses an environmentally extended multiregion input–output model^319,320^ to quantify countries’ contribution to CO_2_ and PM_2·5_ emissions, examining production-based accounting (where physical emissions occur) and consumption-based accounting (allocating emissions to countries on the basis of their consumption of goods and services).

In 2022, 18·7% of global CO_2_ emissions and 19·4% of global PM_2·5_ emissions occurred in the production of goods and services traded between countries in different HDI groups. Despite accounting for only 19·9% of the world’s population, very high HDI countries were responsible for nearly half (48·1%) of the world’s total consumption-based CO_2_ emissions. Very high HDI countries remain the only group for which consumption-based emissions are higher than production-based emissions for both CO_2_ and PM_2·5_, with consumption accounting for 3·7% and 6·1% more emissions of CO_2_ and PM_2·5_, respectively, than their local production alone. Between 2021 and 2022, for very high HDI countries, production-based and consumption-based emissions of PM_2·5_ increased by 6·1% and 8·9%, respectively. However, high and medium HDI countries had the highest level of in situ production-based PM_2·5_ emissions, with net totals of 13·7% and 9·9% of their local PM_2·5_ emissions, respectively, induced by consumption in very high HDI countries. China and the USA accounted for nearly half of global CO_2_ emissions, with 32·9% of production-based emissions and 29·8% of consumption-based emissions produced by China and 13·5% of production-based emissions and 15·9% of consumption-based emissions produced by the USA.

### 4.3 Financial transitions for a healthy future

Shifting finance away from fossil fuels and towards clean energy and health-promoting activities is the most effective way to drive the transition to net zero. The financial transition will take centre stage at COP29, offering an opportunity for a healthier future. This set of indicators monitors the extent to which finance is supporting a healthy transition.

#### Indicator 4.3.1: clean energy investment—headline finding: global clean energy investment grew 10% in 2023 to $1·9 trillion, exceeding fossil fuel investment by 73%

Investing in clean energy is essential for both mitigating climate change and reducing air pollution. Reaching net zero emissions could lead to economic growth, which could, in turn, lead to further investment in clean energy.^321^ Drawing on data from the IEA, this indicator monitors trends in global investment in energy supply, electricity grids, and energy efficiency.^322^

Global clean energy investment reached $1·88 billion in 2023, an increase of 10·4% since 2022 and 72·9% higher than fossil fuel investment of $1·09 billion. Clean energy investment has grown by 55·9% since the Paris Agreement was signed in 2016, whereas fossil fuel investment has fallen by 4·9%. However, fossil fuel investment still attracted 36·6% of global energy investment in 2023. Power sector investment in solar photovoltaic technology reached $480 billion in 2023, more investment than in all other generation sources combined, and global investment in electricity grids and storage grew by 14% to $415 million. However, investment in energy efficiency and end use decreased by 1·3% to $646 million. Clean energy investment exceeded fossil fuel spending in China and other advanced economies by 161% in 2023 but lagged behind fossil fuel spending by 38% in emerging market and developing economies outside China, where clean energy spending only accounted for 17·4% of the global total. To keep 1·5°C within reach, global investments in renewables, grids, and storage need to be tripled by 2030 to double capacity, and spending on energy efficiency needs to be tripled to double the rate of improvement.^322^

#### Indicator 4.3.2: funds divested from fossil fuels—headline finding: between 2008 and the end of 2023, $40·67 trillion was committed to fossil fuel divestment, with health-care institutions accounting for $54·3 billion

Maintaining investments in fossil fuel companies contributes to their expansion and increases the risk of assets becoming stranded as the world shifts to a net zero future. This indicator uses a divestment commitments database managed by Stand.earth to track the value of funds divested from fossil fuels.

From January, 2008, to December, 2023, 1613 organisations, with assets worth at least $40·67 trillion, committed to divest from fossil fuels. Of these, only 28 were health-care institutions, with assets totalling $54·3 billion. In 2023, there were 52 additional recorded commitments to divestment amounting to $154 billion, with only one of these being made by a health-care institution.^323^

Nearly 90% of all divestment ($36·3 trillion) has been announced since the beginning of 2017, suggesting the Paris Agreement might have catalysed movement away from fossil fuel companies. However, although 22·4% of total divestment by health-care institutions occurred in 2017, divestment has stalled since then, with a cumulative total of only 2·1% divested between 2018 and 2023.

#### Indicator 4.3.3: net value of fossil fuel subsidies and carbon prices—headline finding: 84% of the 86 countries reviewed had a net-negative carbon price in 2022, generating a record net subsidy of $1·4 trillion to fossil fuels

Fossil fuel subsidies encourage the use of these fuels and hinder the transition to healthier options, whereas carbon pricing promotes this transition.^324,325^ This indicator calculates net economy-wide average carbon prices and revenues, comparing carbon prices and monetary fossil fuel subsidies across 86 countries responsible for 93% of global CO_2_ emissions.

The energy crisis triggered by Russia’s invasion of Ukraine in 2022 caused a sharp increase in international energy prices. With most countries’ energy systems still heavily reliant on fossil fuels, most resorted to heavy fossil fuel subsidies to control local energy prices. Indeed, in 2022, 44 countries operated a carbon pricing mechanism, but only 14—almost all of which were very high HDI countries—generated a net positive carbon price, a decrease from 22 in 2021. The 72 countries (84%) with net negative carbon prices (ie, net subsidy) allocated $1·4 trillion in 2022 alone, up from $715 billion in 2021, mainly due to much higher international energy prices. Net subsidies exceeded 10% of national health spending in 47 countries and 100% in 23 countries.

Redirecting funds away from fossil fuels and towards activities that promote human health and wellbeing would yield net positive benefits for local communities.^264,311^ However, to ensure energy access, promote health, and lessen disparities, it is crucial that countries reduce their reliance on fossil fuels in favour of more diversified and locally available renewable energy solutions. In the phase-out of fossil fuel subsidies, it is also essential that low-income countries, which are particularly vulnerable to shifting energy costs, are adequately supported.^326^

#### Indicator 4.3.4: fossil fuel and green sector bank lending—headline finding: after a decade of growth, green sector lending declined by 8% from 2021 to 2022, and fossil fuel lending fell by 14%

Redirecting finance away from fossil fuels and towards equitable deployment of low-greenhouse gas emission technologies and infrastructures is essential for a just transition.^264^ This indicator uses Bloomberg data to monitor fossil fuel and green sector debt provided or facilitated by banks.

In 2022, fossil fuel lending still exceeded green lending by $11 billion. Compared with the 2011–16 average ($578 billion), the average annual lending to the fossil fuel sector decreased by 3·8% to $556 billion in the years after the Paris Agreement entered into force (2017–22). Fossil fuel lending fell by 14% from 2021 to 2022, probably reflecting the record profits of the oil and gas sector in 2022.^327^ However, finance levels remain similar to those of 2016—a year in which a historical drop in the price of oil caused a pronounced dip in lending.^328^

Green lending grew rapidly, from $7 billion in 2012 to $498 billion in 2021. In 2021, green lending reached 6·6 times the level it attained in 2016, when the Paris Agreement entered into force. However, it dropped by 8% from 2021 to 2022, probably reflecting an uncertain investment environment in the face of high inflation and rising interest rates, which pose particular challenges for new green projects due to the associated high capital costs.^329^

### Conclusion

The economic losses associated with the health effects of climate change are growing (indicators 4.1.1–4.1.4), increasingly threatening the socioeconomic conditions on which good health and wellbeing depend and thereby compounding the health harms of climate change.

Preventing the worst health harms of climate change requires a transformation of the global economy. However, although there have been some steps away from fossil fuels and towards clean renewable energy, financial and economic support of the fossil fuel industry continues, hampering transition efforts. With their energy systems still heavily reliant on fossil fuels, many countries increased their fossil fuel subsidies in response to the soaring fossil fuel prices that followed Russia’s invasion of Ukraine. As a result, 84% of the 86 countries reviewed had a net negative carbon price in 2022, for a net total of $1·4 trillion (indicator 4.3.3). Bolstered by record profits and permissive policies, fossil fuel companies have persisted in increasing their fossil fuel production plans and were on track to exceed emissions consistent with 1·5°C of heating by 189% in 2040, up from 173% as of March, 2023 (indicator 4.2.2). Moreover, bank lending to the green sector fell for the first time from 2021 to 2022 (indicator 4.3.4).

Concerningly, most countries’ economies and societies are unprepared for the transition to a healthy future, particularly in the lower HDI country groups (indicator 4.2.4). Moreover, the perpetuation of fossil fuels has pushed the cumulative total value of current global coal-fired power generation assets projected to be stranded between 2025 and 2034 to $164·5 billion (indicator 4.2.3).

There is an urgent need for financial mechanisms to support countries in the transition to net zero greenhouse gas emissions. With the Loss and Damage fund now in place, it is crucial that the health impacts of climate change and potential unintended harms to health and to the economy are factored into its disbursement to enable a just transition towards a healthier future.

## Section 5: public and political engagement with health and climate change

The previous sections show that delay in implementing the necessary actions to tackle climate change in line with the goals of the Paris Agreement is increasingly harming people’s health. The countries that have contributed least to rising temperatures are often most affected, and climate change is thereby exacerbating global inequities.^330,331^ The implementation of measures that accelerate the transition away from health-harming fossil fuels and greenhouse gas emission-intensive activities is, therefore, essential to protect people’s health and survival from the growing threats of climate change.^17,332,333^ However, such action requires global and national environments in which different parts of society are engaged with health and climate change.^334,335^

This section focuses on the engagement with health and climate change of societal actors that are crucial for driving action, including media, individuals, scientists, governments, international organisations, and corporations. In addition to tracking changes over the past year, this section reflects on broader shifts in engagement by these actors since 2016, when the Paris Agreement came into effect.^178^ New to this year, the media coverage and scientific engagement indicators also assess the extent to which engagement with health and climate change in these two domains explicitly references fossil fuels.

### 5.1 Media engagement with health and climate change

#### Headline finding: in 2023, 24% of all newspaper articles on climate change mentioned health, a slight decline from 2022

Newspapers have an important role in influencing public engagement with health and climate change^336,337^ and in setting the political agenda.^338^ This indicator tracks coverage of health and climate change in 63 newspapers across 35 countries using a method based on keyword searches of relevant newspaper databases. The sample includes widely read newspapers in English, Chinese, German, Portuguese, and Spanish, covering all six WHO regions and at least one newspaper in each HDI group.

**Figure 14:**
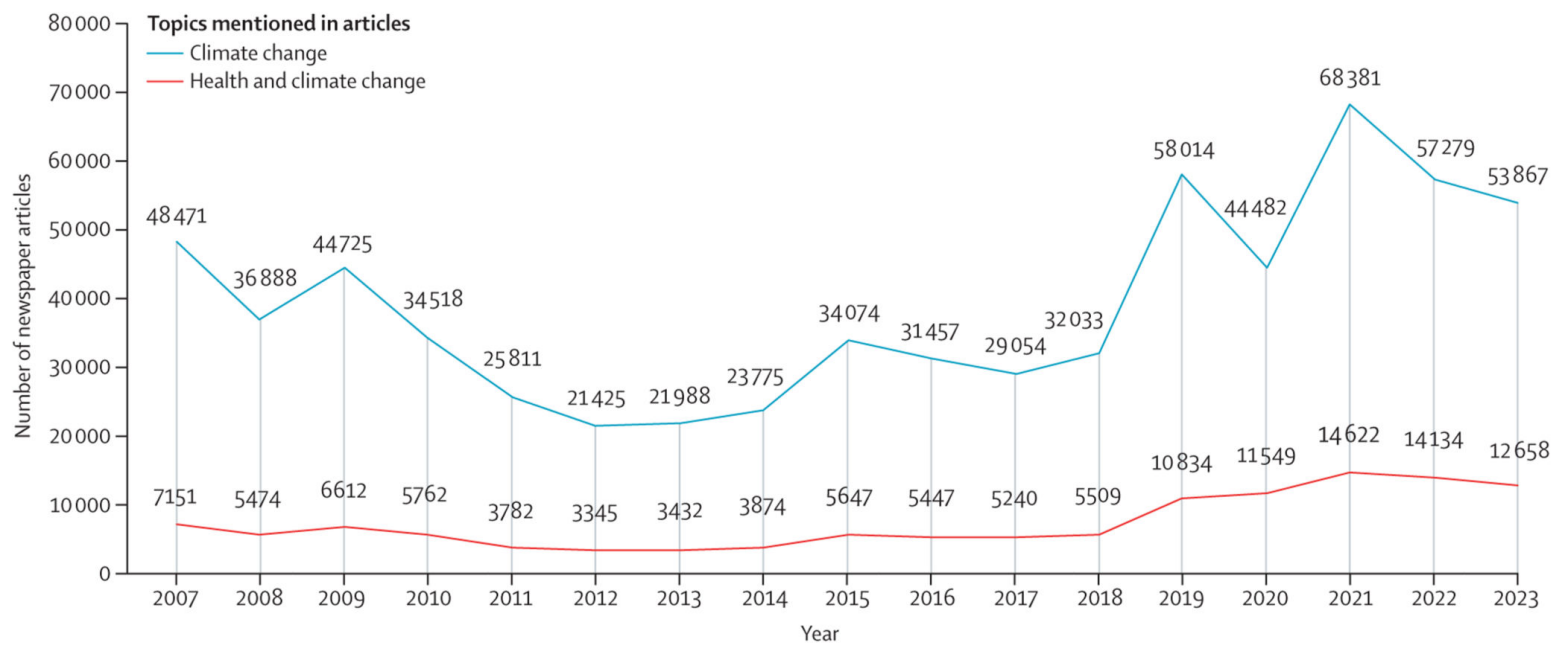
Number of newspaper articles mentioning climate change and number mentioning health and climate change combined in 62 newspapers from 35 countries, 2007–23 (not including China’s People’s Daily)

In 2023, 12 658 (24%) of 53 867 climate change articles referred to health—a decrease of 10% since 2022 (14 134 articles). Despite this decrease, media coverage of health and climate change (and climate change more broadly) has grown substantially since 2016 (figure 14). In 2016, there were 5447 articles discussing health and climate change, whereas in 2023, this figure had risen by 132%, to 12 658. The number of newspaper articles referencing health, climate change, and fossil fuels also increased by 113% between 2016 (1814 articles) and 2023 (3859 articles), promoting greater awareness of the health harms of fossil fuels.

### 5.2 Individual engagement with health and climate change

#### Headline finding: although individual engagement with health and climate change remained low in 2023, the number of views of Wikipedia articles on the human health effects of climate change has increased by 40% since 2022

This indicator measures individual engagement with health and climate change through searches on the online encyclopaedia Wikipedia—a major source of trusted information globally and one of the most visited websites in the world.^339,340^ The indicator tracks individuals’ clicks between articles on health and articles on climate change and vice versa (ie, clickstream activity), focusing on English-language Wikipedia, which represents around 50% of global traffic to Wikipedia.^341^

The indicator finds that individuals rarely move between articles on health and articles on climate change. In 2023, only 0·03% of all click views leading to a health-related article came from a climate change-related article, and only 0·32% of all click views leading to a climate change-related article came from a health-related article; this low clickstream activity is observable across the 2018–23 period. However, with more articles now dedicated to the health–climate change nexus, 2023 saw a 40% increase in average views of the human health effects of climate change articles, compared with 2022.

### 5.3 Scientific engagement with health and climate change

Peer-reviewed journal articles are the primary source of scientific evidence for governments, international organisations, the media, and the public, providing the basis for action on climate change.^342,343^ This set of indicators tracks engagement with health and climate change in the scientific literature.

#### Indicator 5.3.1: scientific articles on health and climate change—headline finding: the number of scientific papers investigating the links between health and climate change increased by 7·4% in 2023, compared with 2022, reaching its highest recorded level

**Figure 15:**
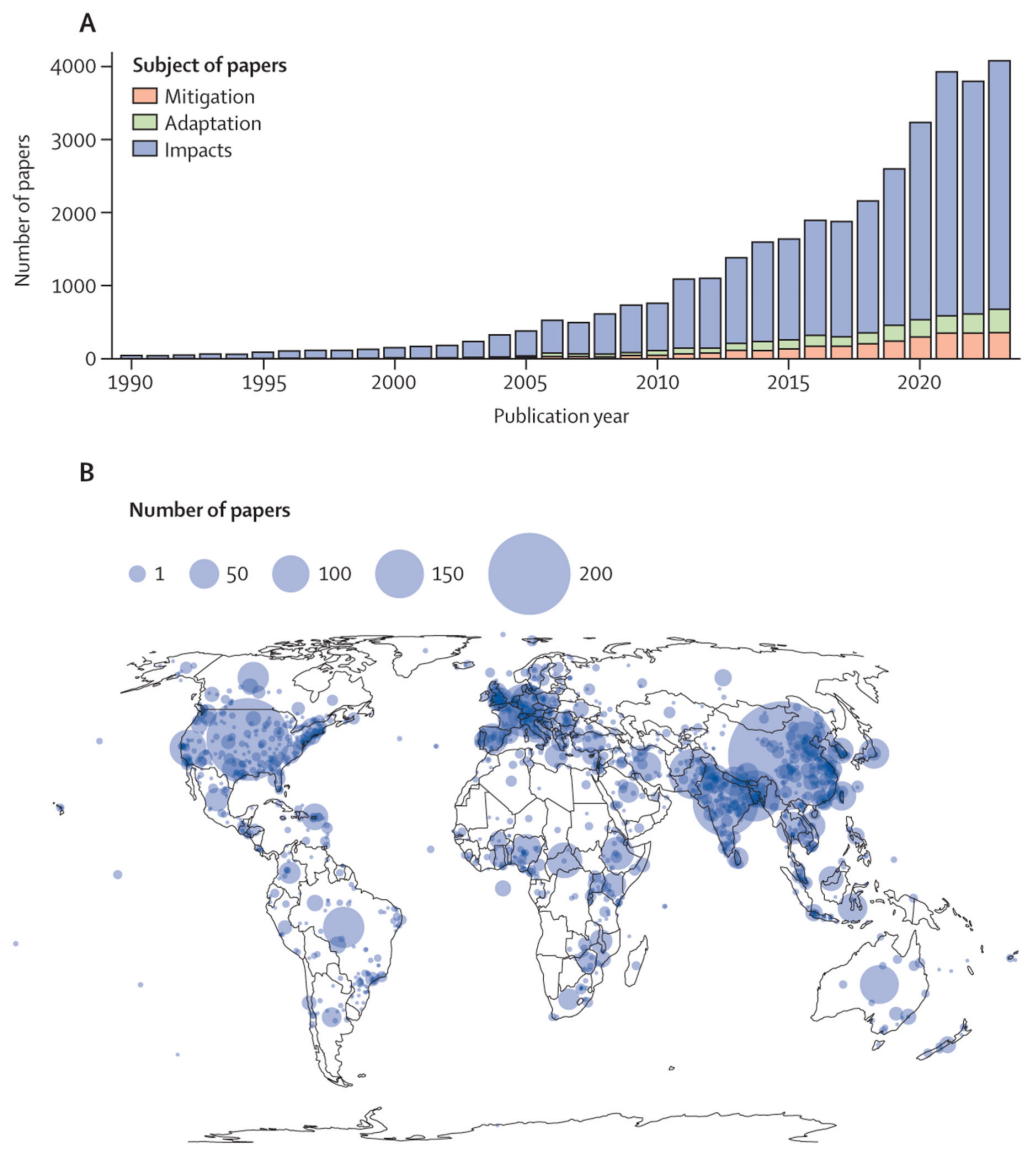
Scientific publications on the nexus of climate and health (A) Number of publications on the nexus of climate and health. (B) Locations of studies on the nexus of climate and health in 2023.

Funding for research on the links between health and climate change has grown in recent years, contributing to greater scientific engagement and understanding.^344^ This indicator uses a machine-learning approach to monitor and classify peer-reviewed academic articles on health and climate change.^345^ The scientific literature has expanded rapidly, with 66% of the approximately 35 802 articles on health and climate change as of December, 2023, having been published since 2016 (figure 15). In 2023, there were 4080 publications on health and climate change, an increase of 7·4% from 2022. Most articles focus on very high and high HDI countries (53% and 32%, respectively), with much less research on medium and low HDI countries (16% and 8%, respectively), and with some articles covering multiple locations. Although 2023 saw an increase in articles on mitigation and adaptation, 3403 (83%) of 4080 articles focused on health impacts (figure 15). Interestingly, only 4% of health and climate change articles explicitly reference fossil fuels in the title or abstract, instead focusing on emissions or heating to understand climate change and its health consequences. Indeed, less than 1% of studies on either health effects or health links to adaptation mention fossil fuels. In contrast, 28% of mitigation studies explicitly reference fossil fuels—particularly coal and diesel—because the focus on the health effects of mitigation is often linked to air pollution from combustion processes. In addition, the predominant language of publications remains English, which creates restrictions for speakers of other languages to access or contribute to the scientific knowledge on health and climate change.

#### Indicator 5.3.2: scientific engagement with the health impacts of climate change—headline finding: 31% of the 54 414 studies published on health impacts of climate variables as of 2023 focus on cases in which changes in climate variables can be attributed to human influence, up by 117% from 2016

Increasing the understanding of the health impacts of anthropogenic climate change is essential for the characterisation of health risks and design of efficient measures to protect and promote health. This indicator tracks the number of scientific studies of the health effects of changes in climate variables in cases in which the changes in those variables can be attributed to anthropogenic climate change.^346^

The cumulative number of articles identified in the chosen databases, which studied events in which changes in the climate variables driving health effects can be attributed to anthropogenic climate change, increased by 117% from the cumulative total as of 2016 (7806) to the total as of 2023 (16 926). However, as a proportion of all articles covering the health impact of climate variables, the proportion of articles studying events in which change in the climate variable driving the health effects can be partially attributable to climate change (hereafter referred to as partially attributable studies) remained largely constant, at 30% (of 25 761) in 2016 and 31% (of 54 414) in 2023. The most common health outcome across all studies published as of 2023 was infectious disease (32% of studies). However, there have been rapid increases in the cumulative number of studies of mental health (210% increase) and water security (217% increase) since 2016, with these topics reaching 9% and 5%, respectively, of all partially attributable studies published as of 2023. Inequalities in terms of the locations of these partially attributable studies persist. Across all publication years, there were 6·6 studies per million people in very high HDI countries and 2·1 studies per million people in high HDI countries, whereas there were 1·5 studies per million people in both medium and low HDI countries. Importantly, 88·0% of first author affiliations were from countries with a very high (63·8%) or high HDI (24·2%), with just 12·0% from countries with a medium (8·2%) or low (4·0%) HDI. These data reflect how scientific research on health and climate change remains heavily dominated by researchers in very high HDI countries and expose the under-representation of researchers from the countries that are most heavily affected by the health impacts of climate change.

### 5.4 Political engagement with health and climate change

Engagement by governments and political leaders is central to delivering climate change action that protects human health.^347,348^ This set of indicators monitors political engagement with health and climate change through national leaders and international organisations.

#### Indicator 5.4.1: government engagement—headline finding: in 2023, 35% of governments mentioned health and climate change in their annual UN General Debate statements, compared with 50% in 2022

The annual UN General Debate provides a global forum for national governments to address the UN General Assembly and discuss priority issues in world politics requiring international action.^349,350^ This indicator monitors references to health and climate change in UN General Debate speeches.

The proportion of countries referencing health and climate change in these speeches declined in 2023—from 97 (50%) of 193 countries in 2022 to 68 (35%) of 192. This proportion, however, is still much higher than in 2016, when only 30 (15%) of 194 governments discussed health and climate change. Engagement continues to be led by the countries least responsible for but most affected by climate change—particularly Small Island Developing States, which represented 39 (57%) of 68 governments discussing the intersection of health and climate change in 2023. These discussions had a strong emphasis on the human devastation of extreme weather events.^351^ The public health costs of climate change were also emphasised, with Fiji’s statement explaining that “for small island developing states, the triple burden of non-communicable diseases, mental health and the climate emergency are straining the health infrastructure and resources”.^352^

The second part of this indicator tracks engagement with health and climate change in the NDCs. As the major policy instrument of the Paris Agreement, countries are required to periodically report more ambitious contributions towards international climate commitments, with updated NDCs.^178,353,354^ The initial increase in countries mentioning health keywords in their NDCs (from 135 [70%] of 192 in the first round of NDCs to 162 [94%] of 172 in the second round) has not continued into the third round of NDCs, with less than half of NDCs submitted as of February, 2024, mentioning a health keyword (27 [47%] of 58). Although these figures indicate a substantial decline in health engagement, fewer than a third of countries, to date, have submitted a third NDC. Across the three rounds of NDCs, engagement with health has been highest among low HDI countries (94–100%) and medium HDI countries (86–97%), followed by high HDI countries (78–97%). Engagement is lowest among very high HDI countries (28–89%).

#### Indicator 5.4.2: engagement by international organisations—headline finding: international organisations focused on climate mitigation and adaptation referred to the health co-benefits of climate mitigation in a record 20% of their X posts in 2023

International organisations (eg, UN agencies, international and regional financial institutions, and supranational bodies such as the EU and African Union) are increasingly at the forefront of action on climate change.^355–357^ This indicator tracks engagement on the health co-benefits of climate mitigation on the official X (formerly Twitter) accounts of international organisations, which remains a key platform for their public communication.^358,359^

The indicator measures engagement with the health co-benefits of climate mitigation using a dataset of 1 406 037 English-language tweets made between 2010 and 2023 by 41 international organisations that have an operational focus on climate mitigation or adaptation across wide-ranging sectors (eg, trade and finance, development and disaster risk management, or food and agriculture). There has been a marked increase in the proportion of tweets mentioning the health co-benefits of climate mitigation since 2016, increasing from 13 040 (11%) of 120 478 tweets that year to 15 525 (18%) of 88 034 tweets in 2022 and reaching a record high of 10 069 (20%) of 51 113 tweets in 2023.

### 5.5 Corporate sector engagement with health and climate change

#### Headline finding: corporate sector engagement with health and climate change increased to its highest level in 2023, with 60% of companies referring to the health dimensions of climate change in their UN Global Compact reports

Corporations have substantial influence over efforts to tackle climate change. A recent report found that 57 public and private corporations produced 80% of all global emissions between 2016 and 2022.^360^ Corporate engagement with health and climate change is, therefore, crucial in the transition to a healthy future.

Over 24 000 companies from 168 countries have signed up to the UN Global Compact, making it the largest global corporate sustainability initiative. Although the UN Global Compact has been criticised for enabling so-called greenwashing, recent evidence suggests companies’ involvement in the UN Global Compact is associated with improved environmental and social responsibility.^361–363^ This indicator measures corporate sector engagement by tracking references to health and climate change in the annual Communication of Progress reports submitted by companies.

Corporate sector engagement with the health-climate change nexus has seen an upward trend since 2016, with the largest increase in engagement occurring over the past year. The proportion of companies mentioning health and climate change in their Communication of Progress reports grew from 559 (16%) of 3573 companies in 2016 to 2314 (38%) of 6089 companies in 2022 and 2744 (60%) of 4567 companies in 2023. A higher proportion of companies continued to engage with health (93% in 2023) and climate change (89% in 2023) separately.

### Conclusion

Engagement with health and climate change has grown substantially since 2016, when the Paris Agreement came into effect. Across different societal actors—the media, scientific community, governments, international organisations, and corporations—engagement with the health–climate change nexus is higher than in 2016, and there are signs that individual engagement is also increasing. This growing awareness was reflected in the unprecedented focus on health at COP28 in December, 2023, which included a health and climate thematic day and the first health and climate ministerial meeting; moreover, 151 countries have now endorsed the COP28 health and climate declaration.^15,364,365^

However, this section also shows that engagement across societal domains remains lower than engagement with health and climate change as separate issues, as evidenced by media coverage, government engagement, and Wikipedia users’ digital footprints. Furthermore, the indicators continue to highlight stark global differences in engagement with health and climate change. The generation of relevant scientific knowledge is lagging for the world’s most affected and vulnerable regions, and calls for action on health and climate change are led the by governments of the countries most exposed to climate change, rather than those most responsible for greenhouse gas emissions. Some indicators, such as media coverage and government engagement, also saw reductions in engagement in 2023, demonstrating that progress can be reversed. This reduction in engagement is especially important given the growing signs in some countries of a backlash from parts of society against specific mitigation policies, despite their health co-benefits.^366–368^ Such political contestation underscores the need to promote people-centred and inclusive society-wide actions to tackle climate change and protect the health and survival of people worldwide.^369^

## Conclusion: the 2024 report of the *Lancet* Countdown

**Figure 16:**
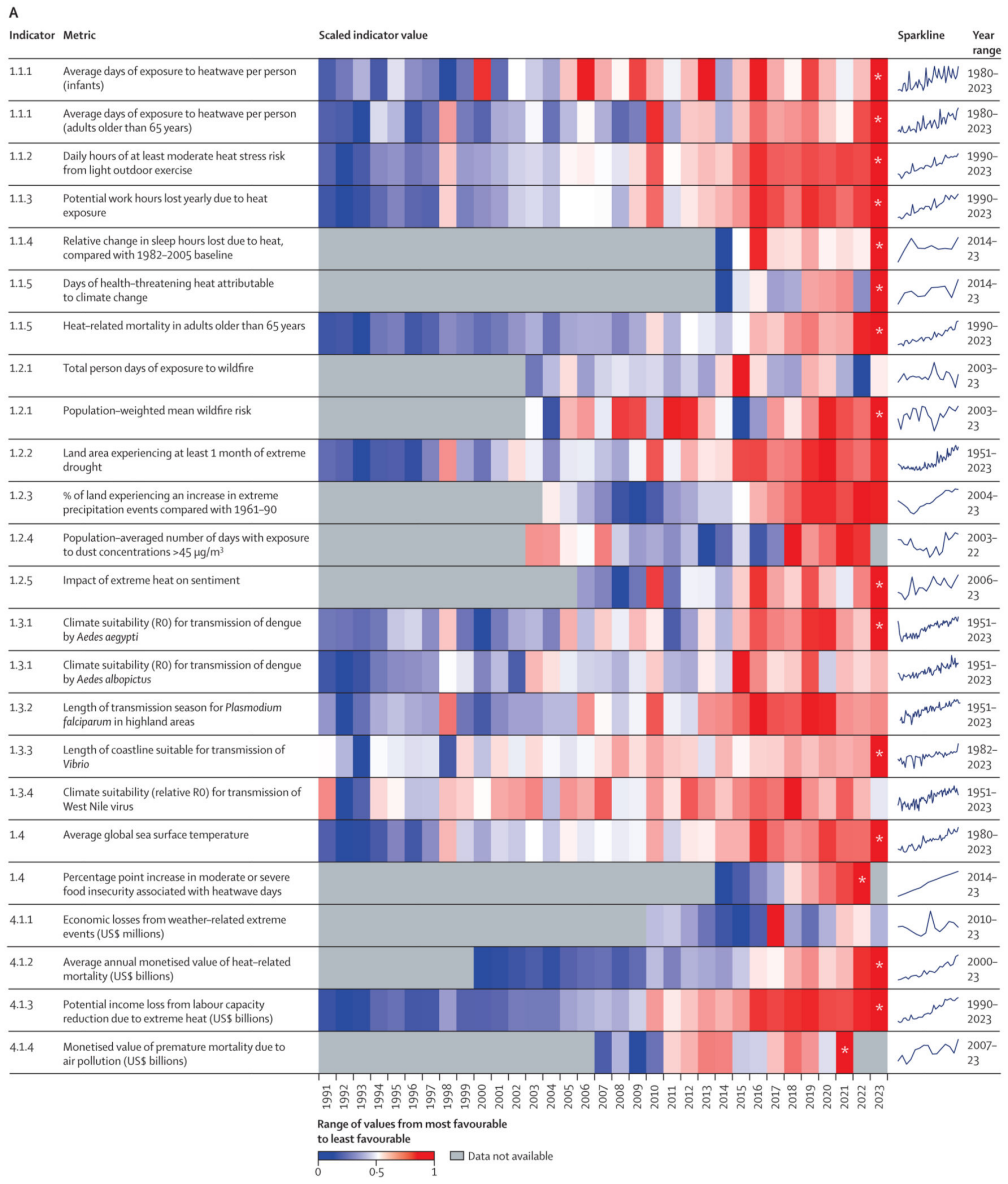

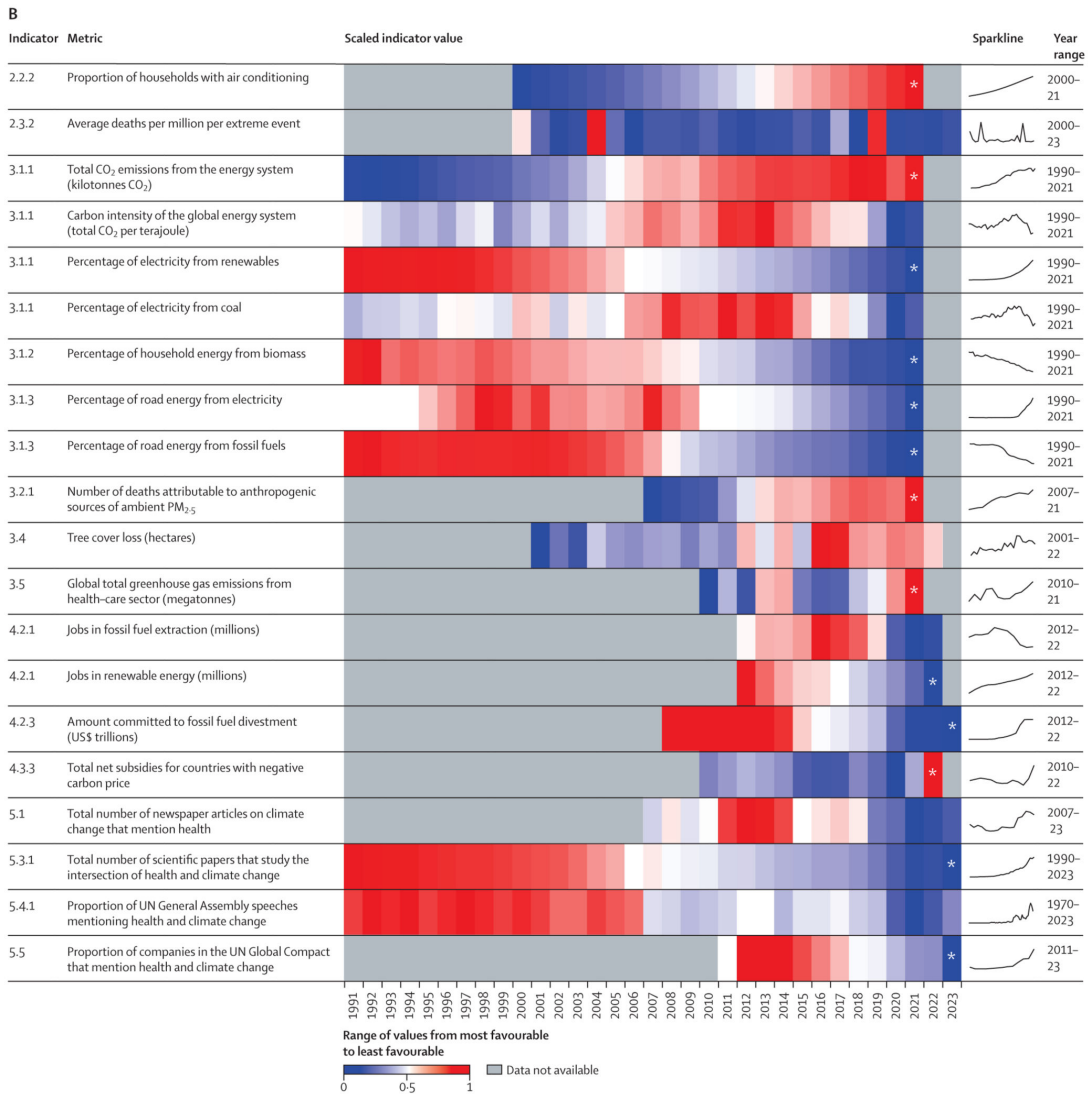
Summary of the evolving links between health and climate change Summary of values of the indicators in the 2024 report of the *Lancet* Countdown for which quantitative data per year are available. The heatmaps present the time series for each indicator, with values linearly scaled into the range 0–1, such that 0 and 1 represent the minimum or maximum values in the time series shown (after 1991), and 0·5 represents the median. Asterisks denote that the indicator reached a record value in the most recent year of data. The sparklines present a line graph with the indicator value in the y-axis and the full range of years for which data are available for each indicator in the x-axis (specified in final column). The scaling and colouring are principally for visualisation, meaning that changes can be overemphasised even if they are not statistically significant. Values do not reflect whether the level of progress made is adequate or offer a comparison between the magnitude of different risks faced. For accurate interpretation, please refer to the data presented in the indicator and in the *Lancet* Countdown’s data visualisation platform. (A) Indicators of health hazards, exposures and impacts (section 1 and section 4.1); higher values (red tones) denote higher levels of health hazards, exposures, or impacts within the time series; lower values (blue tones) denote lower levels of health hazards, exposures, or impacts within the time series. (B) Indicators reflecting responses to climate change (sections 2–5); the scaling was adjusted such that higher values (red tones) denote conditions within the time series that are less favourable towards efforts for tackling climate change and its health risks and lower numbers (blue tones) denote conditions within the time series that are more favourable towards efforts for tackling climate change and its health risks (inverting indicator values as necessary).

Data in this report show that many of the health threats and impacts of climate change are exceeding all previous records (figure 16). In 2023, people were exposed, on average, to an unprecedented 50 more days of health-threatening heat than expected without climate change, resulting in 167% more annual deaths of adults older than 65 years than in the 1990s (indicators 1.1.1 and 1.1.5). The hours of sleep lost due to heat exposure reached 6% above hours lost in 1986–2005, and heat exposure led to record losses of the hours available for safe outdoor physical activity and labour (indicators 1.1.3 and 1.1.4). Meanwhile, heat exposure resulted in a record worsening of online sentiment expressions globally (indicator 1.2.5). In 2023, extreme drought affected 48% of global land area—the second-highest proportion recorded (indicator 1.2.2). Extreme drought contributed to human exposure to dangerous amounts of desert dust, which increased in 48% of countries between 2003–07 and 2018–22, and to the risk of wildfires increasing in 66% of countries between 2003–07 and 2019–23 (indicators 1.2.1 and 1.2.4). The increased frequency of droughts and heatwaves has resulted in a record 151 million more people experiencing moderate or severe food insecurity in 2022 than in 1986–2010 (indicator 1.4). Additionally, the changing climate is making environmental conditions increasingly suitable for the transmission of deadly infectious diseases such as dengue, malaria, vibriosis, and West Nile virus-related illness in new parts of the world (indicators 1.3.1–1.3.4).

Persistent delays in adaptation compound the health effects. As of December, 2023, only 61% of countries that committed to building climate-resilient health systems reported having completed a vulnerability and adaptation assessment, and only 52% had developed an HNAP (indicators 2.1.1 and 2.1.2). Inadequate adaptation has driven more households to use polluting air conditioning; moreover, nature-based solutions, including urban greenspaces, remain underused (indicators 2.2.2–2.2.3, panel 4).

With current policies and actions putting the world on track to 2·7°C of heating by 2100 if maintained,^11^ limits to adaptation are looming closer (panel 5). Transformative, sustained mitigation efforts would not only avoid the most catastrophic impacts of climate change but also the multiple health harms of fossil fuels (panel 6). The transition to clean energy sources could prevent at least 2·3 million deaths annually through reduced solid fuel-derived indoor air pollution and 3.3 million through reduced fossil fuel-derived and biomass-derived outdoor air pollution (indicators 3.2.1 and 3.2.2). Mitigation in the agricultural sector could additionally save 11·2 million lives annually through healthier, more plant-based diets (indicator 3.3.2), and a people-centred transformation could enable healthier cities and lifestyles.

However, the world is increasingly off-track from meeting the goals of the Paris Agreement and, despite some progress in adoption of renewable energy, many key indicators point to a world moving in the wrong direction, with many showing a reversal of progress in the last year of data (figure 16). The carbon intensity of the energy system has remained practically unchanged, and energy-related emissions reached an all-time high in 2023 (indicator 3.1.1), with agricultural emissions growing by 2·8% since 2016. Within the health-care sector itself, emissions increased by 10% between 2020 and 2021 (indicator 3.5).

Delays in implementing the required transformative actions mean that most countries are grossly unprepared for a healthy, net zero greenhouse gas emission future, with people in low and medium HDI countries most at risk (indicator 4.2.4). An entrenched fossil fuel dependence increasingly threatens national economies, with the losses associated with current coal-fired power generation sector assets that are expected to be stranded amounting to a cumulative total of $164·5 billion between 2025 and 2034 (indicator 4.2.3). Meanwhile, the most underserved countries are lagging in the adoption of clean, renewable energy and remain exposed to the harms of energy poverty (indicators 3.1.1 and 3.1.2).

Governments and corporations around the world are exacerbating the risks. Fuelled by record profits, oil and gas giants have expanded their production plans, and, as of March, 2024, were on track to exceed their emissions compatible with 1·5°C by 189% in 2040, 16 percentage points above the year before (indicator 4.2.2). In addition, as energy prices soared and countries’ energy systems remained reliant on fossil fuels in 2022, governments allocated a record-breaking $1·4 trillion to net fossil fuel subsidies (indicator 4.3.3), dwarfing any financial commitments in support of climate action made at COP28.

Against this concerning background, an increased focus on health within UNFCCC negotiations in COP28 and the prioritisation of climate change within the WHO’s GPW 14 mark important progress. The engagement of individuals, corporations, scientists, and international organisations with climate change and health is growing (indicators 5.2, 5.3.1, 5.3.2, 5.4.2, and 5.5), raising hopes that a healthy, prosperous future could still be within reach.

However, avoiding a catastrophic increase in death, disease, and destruction will require urgent, decisive, and health-focused actions, exceeding the ambition of international commitments. Entering a new phase of activities, the *Lancet* Countdown will update its indicator frameworks and increase its efforts to ensure indicators are relevant to inform decision making. Such efforts will include monitoring progress towards the delivery and outcomes of those actions that have been shown to have the potential for delivering a prosperous, healthy future for all.

## Supplementary Material

Supplementary appendix

## Panel 1 New opportunities to put health at the centre of the world’s response to climate change

The 2023 report of the *Lancet* Countdown presented 11 priorities for a healthy future.^17^ These recommendations remain relevant. Over the past year, new opportunities have emerged to enact these recommendations and put health at the centre of the world’s response to climate change. Indicators in this 2024 report inform the following seven near-term opportunities to act on the priorities presented in the 2023 report of the *Lancet* Countdown and deliver a healthy future for all.

- Put health at the centre of national commitments to meet the goals of the Paris Agreement—oportunity: countries are due to update their Nationally Determined Contributions (NDCs) by 2025 in response to the first Global Stocktake. As countries lay out their plans to meet the goals of the Paris Agreement, there is an opportunity to ensure that the health impacts and costs of climate change are accounted for, the health gains of actions to tackle climate change are prioritised, and the health benefits considered and maximised within all commitments put forward in the NDCs, thus ensuring national-level climate change actions to promote and protect people’s health and survival. With mentions of health decreasing in the NDCs submitted as of February, 2024 (indicator 5.4.1), increased engagement of health professionals (including health system leaders, ministries of health, medical practitioners and nurses, public health experts, and academics) will be crucial to ensuring NDCs can promote improved health and wellbeing by meaningfully incorporating health considerations throughout.
- Invest in a healthy future and healthy populations—opportunity: fossil fuel subsidies reached record-high levels in 2022 as energy prices soared, and fossil fuels still attract 36·6% of global energy investment. Fossil fuel funding could be redirected to support a just transition and healthier, more resilient populations. Delivering the actions that protect and promote people’s health in the face of climate change requires sufficient, stable, and predictable financial resources. Financial support for action on health and climate change is still scarce (indicator 2.2.4), and financial resources continue to be allocated to activities that hinder the transition to net zero greenhouse gas emissions, including the allocation of 36·6% of all global energy investments towards fossil fuels (indicators 4.2.3, 4.2.4, and 4.3.1–4.3.4). These resources could be redirected to accelerate a just transition. By phasing out fossil fuel subsidies, savings could be reinvested directly into renewable energy projects (particularly those that support equitable access to and uptake of clean energy), as well as towards targeted support for vulnerable groups that could be affected by subsidy removal (eg, through cash transfers, social protection programmes, and targeted subsidies for clean energy access; indicators 3.1.2 and 3.2.2). Investing in technology transfers and cheaper finance for low-income and middle-income countries (LMICs) that still rely heavily on fossil fuels could also support a global, just, and healthy transition. The associated benefits to human health and wellbeing could, in turn, help build more resilient populations and ultimately benefit the whole economy.
- Put human health at the centre of climate change finance—oppportunity: at COP29, the New Collective Quantified Goal on Climate Finance is due to be adopted, and governing arrangements for the Loss and Damage fund will be considered and approved. The engagement of the health sector with these processes will be essential to ensure that the funding mechanisms optimise the health gains of climate action and account for the economic and non-economic losses and damages associated with the health impacts of climate change and the financial needs of health-centred and just climate change action. This engagement and the outcomes described will be key to ensure the funding can fully support the most affected countries, helping to address and minimise the inequities of climate change. Importantly, the processes for accessing and allocating funding must be simplified and tailored to accommodate the potential requirements of the countries and communities that need it most.^18^
- Set the protection and promotion of human health and wellbeing as the primary measure of climate action—opportunity: the metrics to monitor progress against the Global Goal on Adaptation (GGA) and Fourteenth General Programme of Work (GPW14) are being defined in 2024–25. In addition to the health gains of mitigation across sectors, adaptation in water and sanitation systems, food and agriculture, energy generation, health systems, human infrastructure, natural ecosystems, and the economy hold the potential to improve health outcomes. As the indicators to measure progress against the GGA and GPW14 are defined, indicators that monitor climate-sensitive morbidity and mortality could help ensure adaptation efforts are evaluated against their capacity to protect the most fundamental aspect of human wellbeing, while, in the case of GPW14, also quantifying the health co-benefits of mitigation. These indicators could also help identify and avoid potential unintended health harms of climate action and guide adaptation actions to reduce health inequities.
- Shape societies to promote health, equity, and climate justice—opportunity: at COP28, parties agreed to hold biannual dialogues under the United Nations Framework Convention on Climate Change Just Transition Work Programme. Inadequate and unjust climate change action is leaving the most underserved communities most exposed to the health effects of climate change (Indicators 2.2.2 and 2.2.4–2.2.6), most unprepared for the transition away from fossil fuels (indicator 4.2.4), and most exposed to the health harms of energy poverty and fossil fuel-derived air pollution (indicators 3.1.1–3.1.2). A persistent increase in investment in fossil fuels is increasing the value of assets that will become stranded and expanding the size of a workforce whose employment opportunities will wane as the world transitions to healthy, renewable energy in line with international agreements (indicators 4.2.1, 4.2.3, and 4.3.1). Additionally, an unjust transition to renewable energy could perpetuate harmful global power dynamics, leaving the most vulnerable populations, including Indigenous peoples, exposed to the health harms of extractive industries. Embedding health considerations in the evolving Just Transition Work Programme offers the opportunity to deliver a transition that enables sustainable, equitable, and healthy development pathways and deliver benefits to the health and wellbeing of people worldwide.
- Build future-proofed health systems—opportunities: WHO’s GPW14 and 77th World Health Assembly (WHA77) resolution on health and climate change established responding to climate change as a core priority, including through low-carbon and climate-resilient health systems. Building climate-resilient health systems is essential to ensure that health systems can deliver quality care in the face of climate change. However, to meet global climate targets and avoid causing harm to the health of the populations they serve, and beyond, health systems will simultaneously need to remove the 4·6% of greenhouse gas emissions they contribute (indicator 3.5), which will be a substantial challenge. The GPW14 offers a framework to deliver low-carbon, climate-resilient health systems that are fit for the future, an effort for which WHO’s Alliance for Transformative Action on Climate and Health is providing support.^19^ Ensuring countries engage with these processes will be essential to deliver health systems that are fit to operate in future climates.
- Tackle climate change through public health interventions—opportunities: WHO’s GPW14 and WHA77 resolution established a priority to address health determinants and the root causes of ill health in key policies across sectors, including by tackling air pollution and unhealthy diets. Tackling greenhouse gas emissions and delivering adaptation to climate change are core priorities of climate change action and will help build a safer, healthier future for all. However, public health interventions that address the root causes of ill-health could also contribute to these goals: public health interventions aimed at reducing exposure to air pollution could save millions of deaths annually while promoting the shift to clean energy sources (indicators 3.2.1 and 3.2.2). Promoting and enabling healthier diets could save millions of deaths each year while aiding mitigation in the agri-food sector (indicators 3.3.1 and 3.3.2).

Responding to these new opportunities could help drive transformative changes to tackle climate change and deliver major improvements to people’s health. However, the success of this transformation requires simultaneous and sustained efforts to prepare for a health-centred response to climate change. These efforts include:

- Building capacity on health and climate change by providing formal training in climate change and health; making climate and health education part of core curricula within health education programmes would represent an important step to meet this goal (indicator 2.2.6).
- Engaging with, respecting, and elevating the knowledge and leadership of Indigenous peoples, frontline communities, and minoritised groups in the design and implementation of health and climate change policies, to ensure that they can protect people’s health and avoid unintended harms; these communities are often the ones most affected by the actions needed to tackle climate change and, in many cases, hold the key to their effective implementation (sections 2 and 3).^20,21^
- Integrating health in all climate change policies at the international, national, and local levels, through cross-sectoral cooperation, as a key to ensure climate change actions, can protect and promote people’s health and survival.

These priorities are, however, insufficient on their own. To be effective, they rely on the world meeting the mitigation goals laid out in the Paris Agreement, for which other initiatives have provided detailed roadmaps.^22–24^ Unless that basic prerequisite is met, these priorities will have little effect, if any, in protecting people’s health.

## Panel 2 The indicators of the 2024 report of the *Lancet* Countdown

**1 Health hazards, exposures, and impacts**
  1.1 Heat and health
    1.1.1 Exposure of vulnerable populations to heatwaves
    1.1.2 Heat and physical activity
    1.1.3 Change in labour capacity
    1.1.4 Rising night-time temperatures and sleep loss
    1.1.5 Heat-related mortality
  1.2 Health and extreme weather-related events
    1.2.1 Wildfires
    1.2.2 Drought
    1.2.3 Extreme precipitation
    1.2.4 Sand and dust storms
    1.2.5 Extreme weather and sentiment
  1.3 Climate suitability for infectious disease transmission
    1.3.1 Dengue
    1.3.2 Malaria
    1.3.3 *Vibrio*
    1.3.4 West Nile virus
  1.4 Food security and undernutrition
**2 Adaptation, planning, and resilience for health**
  2.1 Assessment and planning of health adaptation
    2.1.1 National assessments of climate change impacts, vulnerability, and adaptation for health
    2.1.2 National Adaptation Plans for health
    2.1.3 City-level climate change risk assessments
  2.2 Enabling conditions, adaptation delivery, and implementation
    2.2.1 Climate information for health
    2.2.2 Benefits and harms of air conditioning
    2.2.3 Urban greenspace
    2.2.4 Global multilateral funding for health adaptation programmes
    2.2.5 Detection of, preparedness for, and response to health emergencies
    2.2.6 Climate and health education and training
  2.3 Vulnerabilities, health risk, and resilience to climate change
    2.3.1 Vulnerability to severe mosquito-borne disease
    2.3.2 Lethality of extreme weather events
    2.3.3 Rising sea levels, migration, and displacement
**3 Mitigation actions and health co-benefits**
  3.1 Energy use, energy generation, and health
    3.1.1 Energy systems and health
    3.1.2 Household energy use
    3.1.3 Sustainable and healthy road transport
  3.2 Air quality and health co-benefits
    3.2.1 Mortality from ambient air pollution by sector
    3.2.2 Household air pollution
  3.3 Food, agriculture, and health co-benefits
    3.3.1 Emissions from agricultural production and consumption
    3.3.2 Diet and health co-benefits
  3.4 Tree cover loss
  3.5 Health-care sector emissions and harms
**4 Economics and finance**
  4.1 The economic impact of climate change and its mitigation
    4.1.1 Economic losses due to weather-related extreme events
    4.1.2 Costs of heat-related mortality
    4.1.3 Loss of earnings from heat-related labour capacity reduction
    4.1.4 Costs of the health impacts of air pollution
  4.2 The transition to net zero carbon, health-supporting economies
    4.2.1 Employment in low-carbon and high-carbon industries
    4.2.2 Compatibility of fossil fuel company strategies with the Paris Agreement
    4.2.3 Stranded coal assets from the energy transition
    4.2.4 Country preparedness for the transition to net zero
    4.2.5 Production-based and consumption-based attribution of CO_2_ and PM_2·5_ emissions
  4.3 Financial transitions for a healthy future
    4.3.1 Clean energy investment
    4.3.2 Funds divested from fossil fuels
    4.3.3 Net value of fossil fuel subsidies and carbon prices
    4.3.4 Fossil fuel and green sector bank lending
**5 Public and political engagement with health and climate change**
  5.1 Media engagement with health and climate change
  5.2 Individual engagement with health and climate change
  5.3 Scientific engagement with health and climate change
    5.3.1 Scientific articles on health and climate change
    5.3.2 Scientific engagement with the health impacts of climate change
  5.4 Political engagement with health and climate change
    5.4.1 Government engagement
    5.4.2 Engagement by international organisations
  5.5 Corporate sector engagement with health and climate change

## Panel 3 Indigenous knowledge for a healthy future

Indigenous peoples maintain deep connections with the natural environment that are important for the social, livelihood, cultural, and spiritual practices that underpin their health and wellbeing.^31^ This close relationship makes Indigenous peoples particularly susceptible to climate change-related hazards. The effects of climate change threaten the natural resources on which they depend for food, medicine, and cultural practices,^32^ affecting health-seeking behaviours,^33–35^ jeopardising livelihoods, and increasing the risk of climate-induced mobility or immobility, which, in itself, can affect the sense of identity and physical and mental health.^36,37^

Indigenous peoples’ physical and mental health is often poorer than that of their non-Indigenous counterparts, an inequity that could be perpetuated or aggravated if not addressed when implementing climate change policies.^38,39^ Although Indigenous peoples represent 6% of the global population,^40^ they are often excluded and minoritised within national and international political systems, with little recognition of their needs within policy making.^21^ Indigenous health systems and cosmogonies are rarely represented within most countries’ dominant health systems, making them inadequate to meet their health needs. Additionally, Indigenous medicine and worldviews are rarely considered within health care or health risk preparedness and response.^41,42^ Despite Indigenous peoples’ knowledge often being neglected in these processes, it has an essential role in avoiding, reducing, and managing climate-related health threats, including by informing effective actions to safeguard the food security, natural resources (such as medicinal plants, forest and farm food), and housing of Indigenous peoples.^31,36,43–46^ Knowledge frameworks such as atua matua, a Māori concept, and buen vivir, from the Quechua people, contribute to caring about the environment.^47,48^ This knowledge is based on a deep and ancestral connection with natural resources that has been passed from generation to generation.^48–50^ The capability of Indigenous peoples and their knowledge to support climate change action is also seldom acknowledged within climate adaptation programmes at national and international levels.^51–53^ The magnitude of current climate change impacts, persistent socioeconomic exclusion of Indigenous peoples, and scarce guarantee of Indigenous collective rights, safety, and integrity, jeopardise the opportunities of Indigenous peoples to respond, adapt to, and protect their health systems and wellbeing from environmental changes.^46,54^ This marginalisation often also results in profound negative impacts on physical and mental health.^38,55–57^ For example, without accounting for Indigenous systems, climate mitigation policies have created conflict over land ownership, affecting Indigenous eople’s identity, wellbeing, and health systems.^58–60^

To be effective for all peoples, climate change policies must respect Indigenous peoples’ rights and dignity, so that they can design responses that truly protect and promote their identity, culture, health, and wellbeing.^61–64^ For example, some Indigenous peoples in the Amazon mostly drink a fermented casava beverage, which generally contains less coliform bacteria than water directly collected from the environment.^65^ This practice could be a culturally appropriate adaptation measure to reduced water availability as climate impacts worsen. Some studies have also documented a change in the timing of planting and in the crops used by Indigenous peoples.^66^ Indigenous peoples’ knowledge has been shown to be the key to protect Indigenous health in times of health emergencies when official health systems and governments are unable to provide assistance to Indigenous communities.^67^ Furthermore, respecting, recognising, and promoting the use and transmission of Indigenous knowledge is not only crucial to safeguard Indigenous peoples’ health in the face of climate change but can also inform further adaptation policies that benefit society as a whole.^67,68^ Indigenous practices and knowledge could also substantially contribute to meeting global mitigation targets: Indigenous peoples’ lands store around 300 billion metric tons of carbon,^69^ and Indigenous knowledge of protection of natural environments could inform a transition to more sustainable social systems.^61,70^

However, valuable Indigenous knowledge has been jeopardised by the disruption of traditional systems of knowledge transmission, including through colonisation, enforcement of non-Indigenous schooling, land dispossession, and environmental change itself.^45,71–73^ The implementation of formal systems to embed and elevate Indigenous knowledge in climate change policy making and enable and promote active participation of Indigenous peoples in the design of global, national, and local climate change policies is, therefore, urgent.^74^ In response, international research networks are engaging with Indigenous peoples to co-create knowledge to respond to the compounding threats of health crises, climate change, and food insecurity, proposing new paradigms for increasing the representation and consideration of Indigenous knowledge in planetary health.^67^ Climate change and health researchers, policy makers, and practitioners have the opportunity to drive a response to climate change that can create better and fairer conditions for all peoples, including Indigenous peoples. This approach is the only way forward to deliver a just, healthy future for all.

## Panel 4 Nature-based solutions

Nature-based solutions are actions that address climate change and other environmental challenges by using and enhancing the capacity of natural ecosystems. Adequately implemented, they could be powerful tools for mitigating and adapting to climate change as they provide a range of benefits that support sustainable development, including climate resilience, carbon sequestration, and biodiversity conservation.^189^ Crucially, nature-based solutions can also result in improved human health and wellbeing.

The restoration of degraded landscapes, such as forests and grasslands, is a prime example of a nature-based solution with high potential for health co-benefits.^190^ Restoration of degraded forests and other ecosystems can significantly increase carbon sequestration, contributing to climate change mitigation while also improving resilience by supporting biodiversity conservation, and providing livelihood opportunities for local communities, thereby benefitting their socioeconomic conditions and physical and mental health.^191^ Restoration can also improve local air quality, with trees and other plants absorbing pollutants from the air. It might also help reduce the risk of respiratory and infectious diseases, particularly where land degradation has previously favoured disease hosts or vectors or exacerbated land erosion and exposure to dust (indicator 1.2.3).^192^

Nature-based solutions include the promotion of agroforestry, which involves integrating trees and other native or high-value plants into agricultural landscapes. Agroforestry practices can increase the resilience of agricultural systems to climate change by improving soil health, increasing water retention, and providing shade and windbreaks for crops.^193^ Agroforestry can also contribute to carbon sequestration, biodiversity conservation, and the provision of additional sources of income for farmers. These processes, in turn, can boost food security and dietary diversity, with important nutritional benefits that can help combat the growing food insecurity and malnutrition (indicator 1.4.2).^194^

Implementation of nature-based solutions, nevertheless, faces several challenges, particularly in low-income and middle-income countries, where there are difficulties obtaining access to finance, technical expertise, and supportive policies. In many cases, local communities and Indigenous peoples, who often have the most knowledge and experience in nature-based solutions, are excluded from decision-making processes and are not adequately compensated for their contributions or losses (panel 3).^70^ Additionally, nature-based solutions might not be prioritised alongside other socioeconomic development imperatives in the places that might have the most to gain from them. For instance, urban green spaces such as parks and gardens are often overlooked or underfunded in rapidly growing urban centres (indicator 2.2.3),^195^ despite evidence showing that urban green space can benefit mental health, including by reducing stress, anxiety, and depression, and reduce urban heat stress.^196,197^

To overcome these challenges, there is a need for increased investment in nature-based solutions and a better understanding of the range and magnitude of health benefits (or, in some cases, harms) they might provide. This investment includes financing for large-scale restoration and conservation programmes, support for local communities to implement nature-based solutions, and policy reforms that promote sustainable land use and management. There is also a need for increased collaboration between different stakeholders, including governments, Indigenous peoples, civil society, and the private sector, to ensure that nature-based solutions are maximally beneficial, in line with local goals, and effectively implemented and scaled up.^198^ By enhancing the capacity of natural ecosystems, nature-based solutions can support healthier and more resilient communities, which is particularly important in the context of climate change and other environmental challenges.

## Panel 5 Limits to adaptation

The current challenges of climate change to people’s health and survival are unprecedented (section 1), with new records breached as global temperatures rise. With further heating unavoidable and current measures falling short of their potential, urgent and robust adaptation is necessary to prevent a rapid escalation of climate-related health impacts.^204^

However, although the need for adaptation is urgent, its capacity to effectively safeguard people’s health is finite. According to the Intergovernmental Panel on Climate Change, limits to adaptation represent points at which systems’ needs cannot be secured from intolerable risks through adaptive action, differentiating so-called soft and hard limits.^205^ Soft limits exist because of the current unavailability of some existing adaptation actions and stem from weak leadership and governance, institutional constraints, or inadequate funding. Hard limits occur when it is no longer feasible to adapt, resulting in an increased risk of irreversible losses and damages.^177^

From a health adaptation perspective, reaching these limits is becoming increasingly likely as climate change continues, challenging the capacity to effectively adapt to climate-related health impacts. Based on the *Lancet* Countdown’s evidence and expertise, it is possible to identify several limits to health adaptation:

- Evidence and knowledge limits—evidence and knowledge are enablers of health adaptation and help to identify effective ways to protect people’s health. However, there are limits to the generation and implementation of useful and actionable evidence, which originate from human limits to understanding the interaction between the natural world and health outcomes.
- Financial limits—under the current mitigation course, adaptation alone will be very costly and require the reallocation of funds from other sectors. These limits are more marked in lower income countries and settings, particularly given the paucity of international funds allocated to supporting adaptation actions, especially for health (indicator 2.2.4).
- Governance or policy limits—most health-related adaptation measures require actions in sectors that support people’s health, including sanitation, urban planning, transport, and energy, to optimise resources, maximise benefits, and optimise trade-offs. These actions require articulated cooperation across governmental sectors, effective executive functions, and adequate funding flows at the international, national, and sub-national levels, all of which rarely occur.
- Physiological limits—human physiological functions require a narrow range of environmental conditions, including with respect to heat stress. As climate change drives environmental changes at a pace faster than humans have ever been exposed to,^206,207^ these limits are increasingly likely to be reached.^208^ More severe extremes of heat and humidity, for example, will probably cause the limits of human survivability to be exceeded in some regions, due to heat stroke. Although these limits have traditionally been defined as a 6-h exposure to a wet bulb temperature of 35°C,^209^ recent physiological studies indicate that the true limit is lower, especially in older people.^210,211^
- Physical or infrastructure limits—in some cases, the physical environment exacerbates the risks of climate change to people’s health, but there are often limits to modifying it. These include, for example, physical limits to protect low-lying coastal areas, flood protection barriers, or built elevation as sea levels continue to rise. Similarly, modifying the built environment to make it more climate-resilient can be very expensive, disruptive, or slow, making it an unavailable option. Such modifications include large-scale engineering works to improve water or sewage systems, flood protection, or increasing urban greenspaces when space is scarce.
- Social, cultural, and behavioural limits—behavioural changes could help build resilience to climate change. Such changes include, for example, adopting behaviours to shield from heat and seek cooling solutions in cases of heatwaves, seeking safety during extreme events, or migrating. However, people’s capacity or willingness to adopt protective behavioural changes is often limited by their habits, culture, beliefs, or social networks.

Overcoming these limits is theoretically possible through major societal, institutional, and technological shifts, requiring a combination of adaptation and mitigation measures. Whole-of-society and cross-sectoral actions (eg, formal and sustained financing, strong governance, and political commitment) are urgently required to face current and future climate hazards to overcome soft limits and avoid reaching hard limits in the short and long terms.

## Panel 6 The health impacts of the energy production lifecycle

Clean and affordable energy access is fundamental for good health. It helps keep indoor temperatures within healthy limits, contributes to food and medicine preservation, enables the delivery of quality health-care services, and supports health and development through access to information and education.^232^ However, the energy system can also adversely affect health throughout its whole life cycle, including extraction, production, operation, and disposal of energy sources.

Fossil fuels are the largest contributors to the global energy system and can affect health throughout their life cycle. Workers and inhabitants in proximity to fossil fuel extraction sites are most affected compared with the wider population. Some occupational health hazards are caused by physical working conditions such as poor lighting, high noise levels, and low oxygen levels in mining, fracking, or drilling during fossil fuel extraction.^233^ Substantial population health risks originate from exposure to air, soil, and water pollution at different stages of the fossil fuel life cycle,^234^ with children being disproportionately affected; Indigenous populations, who are over-represented near extraction sites, remain at greater risk than the general population.^235^

Health impacts of fossil fuel extraction and energy production:

- Exposure to hazardous air pollutants produced during coal combustion in power plants, industrial production, and unconventional gas drilling causes respiratory symptoms, cardiovascular diseases, higher risk of cancer, and preterm delivery among workers and populations living near extraction sites and power plants^234,236^
- Oil and gas extraction processes expose workers to various hazardous chemicals and particulate matter, which can cause long-term health effects on the skin, eyes, and brain and nervous, respiratory, and gastrointestinal systems^237,238^
- Exposure to oil through contaminated water can produce neurological and haematological disorders and skin and eye irritation in inhabitants near oil fields; genotoxicity has been observed consistently among residents living near oil fields^239^
- Exposure to air pollution from fossil fuels at the point of end use (eg, heating, lighting, and cooking), particularly nitrogen dioxide and particulate matter, has substantial negative health impacts
- Household-level coal combustion can cause fluorosis, arsenism, selenosis, and lung cancer and affect child development^234^
- Natural gas combustion can cause bronchoconstriction, airway hyper-responsiveness, and airway inflammation, with increased risk of asthma, bronchitis, and wheezing^240^

The end of life for fossil fuels includes further risks and exposures:

- Coal combustion produces coal ash containing radioactive elements, minerals, and heavy metals, with reported health impacts on children living near coal ash impoundments^234^
- Oil spill cleanup workers have developed chronic conditions of psychological disorders, lower respiratory tract symptoms, reduction of lung function, genotoxicity, and alterations in hormonal status^239^

For non-fossil-fuel-based energy, which includes energy from biomass, nuclear power, and electricity stored in batteries and from renewable sources (eg, solar energy, wind power, hydropower, geothermal energy, and hydrogen), health effects happen at the stage of extraction and production, primarily among the workers involved. In addition to typical workplace hazards, a few types of occupational hazards and diseases are known to be associated with energy industry workers and communities living nearby. Although workplace hazards are not unique to the energy industry, the health impacts might be exclusive when hazards are in conjunction with extreme working conditions.^241^

Risks for non-fossil-fuel workers:

- Falling and other accidents—e.g, during the installation of turbines in the wind industry^241^ and the installation of photovoltaic panels for solar energy^242^
- Exposure to hazardous substances—eg, toxic fumes from heat or other volatile compounds generated in the wind industry,^241^ toxic methylmercury converted from mercury in soils when dams are built for hydropower,^243^ and toxic organic materials used in the processes of biofuel manufacture^244–246^ and making photovoltaics;^247^ heavy metals, radioactive materials, and hydrogen sulphide occur in the development of geothermal energy^248^
- Fire and explosion, freeze burns, and electrical shocks are observed in workers in the hydrogen industry, battery industry, and biofuel manufacture sector; many deaths and injuries have been caused by hydrogen explosions in creating hydrogen fuel cells^249^ and methanol explosions in the process of biofuel blending and alcohol denaturing^245^
- Health impacts can occur from the environmental risks of metal and rare earth ore extraction for the production of batteries, solar panels, and wind turbines;^250^ mining activities might contaminate soil, water, and air with dust, small particulate materials, harmful pollutants, and toxic chemicals, leading to health problems for people living in or near mining areas; ores might also contain radioactive material, causing cancers at high levels of long-term exposure^251,252^

For non-fossil fuels, the operation-to-use stage and end-of-life stage health effects include the following:

- Fire and explosion of lithium-ion batteries (thermal runaway phenomena) and leakage of chemical materials due to improper use, storage, and disposal^253,254^
- Exposure to hazardous groundwater and soil contaminants due to improper disposal of photovoltaics^255^ or exposure to radioactivity due to accidents during power generation and improper nuclear waste disposal
- Similar to fossil fuels, air pollution and aerosol particles released by burning biomass, mainly from traditional biofuels (eg, wood and waste), for cooking and heating might cause health impacts^256^
- No robust scientific evidence suggests wind turbines result in physiological health effects,^257^ but the annoyance generated by low-frequency noise might cause psychosocial harms;^253,254,258^ living near nuclear power plants might also cause psychological impacts due to various socio-psychological factors, such as fear of exposure, disasters, or lack of transparency regarding actual or potential leaks^259,260^

Taking all of the above impacts into account, the health effects of energy from non-fossil fuels are, overall, considerably lower than those of fossil fuels. However, it is important that, as the transition to zero carbon energy gets underway, the risks of renewable energies are carefully assessed and managed to avoid unintended harms and an unintentional exacerbation of global health inequities.

## References

[1] Copernicus Climate Change Service Copernicus: global temperature record streak continues—April 2024 was the hottest on record.

[2] World Metereological Organization (2024). Climate change indicators reached record levels in 2023.

[3] Zachariah M, Kotroni V, Kostas L (2023). Interplay of climate change-exacerbated rainfall, exposure and vulnerability led to widespread impacts in the Mediterranean region.

[4] Kimutai J, Barnes C, Zachariah M (2023). Compounding natural hazards and high vulnerability led to severe impacts from Horn of Africa flooding exacerbated by climate change and Indian Ocean Dipole.

[5] Zachariah M, Philip S, Pinto I (2023). Extreme heat in North America, Europe and China in July 2023 made much more likely by climate change.

[6] Zachariah M, Vautard R, Chandrasekaran R (2023). Extreme humid heat in South Asia in April 2023, largely driven by climate change, detrimental to vulnerable and disadvantaged communities.

[7] Philip S, Kew S, Vautard R (2023). Extreme April heat in Spain, Portugal, Morocco & Algeria almost impossible without climate change.

[8] Barnes C, Boulanger Y, Keeping T (2023). Climate change more than doubled the likelihood of extreme fire weather conditions in Eastern Canada.

[9] UNICEF (2023). Children displaced in a changing climate: preparing for a future already underway.

[10] Newman R, Noy I (2023). The global costs of extreme weather that are attributable to climate change. Nat Commun.

[11] Climate Action Tracker (2023). The CAT thermometer.

[12] Lenton TM, Armstrong McKay DI, Loriani S (2023). Global tipping points: summary report.

[13] McKay DIA, Staal A, Abrams JF (1979). Exceeding 1.5°C global warming could trigger multiple climate tipping points. Science.

[14] United Nations Framework Convention on Climate Change (2024). https://unfccc.int/sites/default/files/resource/cma2023_16a01_adv_.pdf.

[15] WHO (2023). COP28 UAE declaration on climate and health.

[16] Romanello M, Whitmee S, Mulcahy E, Costello A (2023). Further delays in tackling greenhouse gas emissions at COP28 will be an act of negligence. Lancet.

[17] Romanello M, Di Napoli C, Green C (2023). The 2023 report of the *Lancet* Countdown on health and climate change: the imperative for a health-centred response in a world facing irreversible harms. Lancet.

[18] Uri I, Robinson SA, Roberts JT, Ciplet D, Weikmans R, Khan M (2024). Equity and Justice in loss and damage finance: a narrative review of catalysts and obstacles. Curr Clim Change Rep.

[19] WHO (2023). Operational framework for building climate resilient health systems.

[20] Romanello M, McGushin A, MacGuire FAS (2021). Monitoring climate change and child health: the case for putting children in all policies. J Paediatr Child Health.

[21] Deivanayagam TA, English S, Hickel J (2023). Envisioning environmental equity: climate change, health, and racial justice. Lancet.

[22] Mukherji A, Thorne P, Cheung WWL (2023). Synthesis report of the IPCC sixth assessment report (AR6).

[23] International Renewable Energy Agency (2023). World energy transitions outlook 2023: 1 5°C pathway.

[24] International Energy Agency (2022). An updated roadmap to net zero emissions by 2050.

[25] Treen KMI, Williams HTP, O’Neill SJI (2020). Online misinformation about climate change. Wiley Interdiscip Rev Clim Change.

[26] West JD, Bergstrom CT (2021). Misinformation in and about science. Proc Natl Acad Sci USA.

[27] Hartinger SM, Palmeiro-Silva YK, Llerena-Cayo C (2024). The 2023 Latin America report of the *Lancet* Countdown on health and climate change: the imperative for health-centred climate-resilient development. Lancet Reg Health Am.

[28] van Daalen KR, Tonne C, Semenza JC (2024). The 2024 Europe report of the *Lancet* Countdown on health and climate change: unprecedented warming demands unprecedented action. Lancet Public Health.

[29] Beggs PJ, Trueck S, Linnenluecke MK (2024). The 2023 report of the MJA-*Lancet* Countdown on health and climate change: sustainability needed in Australia’s health care sector. Med J Aust.

[30] Watts N, Adger WN, Ayeb-Karlsson S (2017). The *Lancet* Countdown: tracking progress on health and climate change. Lancet.

[31] Food and Agriculture Organization of the UN (2021). The White/Wiphala Paper on Indigenous Peoples’ food systems.

[32] Brubacher LJ, Chen TTW, Longboat S (2024). Climate change, biodiversity loss, and Indigenous Peoples’ health and wellbeing: a systematic umbrella review protocol. Syst Rev.

[33] US Global Change Research Program (2016). Climate and Health Assessment.

[34] Justo-Chipana M, Moraes RM (2015). Plantas medicinales comercializadas por las chifleras de La Paz y El Alto (Bolivia). Ecol Boliv.

[35] Torrez V, Ruiz Sanguino MC, Chura Z, Davila A, Xavier Claros R (2019). Seguridad alimentaria en el Ayllu Corpa—Altiplano Norte De Bolivia.

[36] Ford JD (2012). Indigenous health and climate change. Am J Public Health.

[37] Ayeb-Karlsson S, Hoad A, Trueba ML (2024). ‘My appetite and mind would go’: Inuit perceptions of (im)mobility and wellbeing loss under climate change across Inuit Nunangat in the Canadian Arctic. Humanit Soc Sci Commun.

[38] Anderson I, Robson B, Connolly M (2016). Indigenous and tribal peoples’ health (The Lancet-Lowitja Institute Global Collaboration): a population study. Lancet.

[39] Pollock NJ, Naicker K, Loro A, Mulay S, Colman I (2018). Global incidence of suicide among Indigenous peoples: a systematic review. BMC Med.

[40] UN Indigenous Peoples: respect not dehumanization.

[41] Redvers N, Blondin B (2020). Traditional Indigenous medicine in North America: a scoping review. PLoS One.

[42] Asamoah GD, Khakpour M, Carr T, Groot G (2023). Exploring Indigenous traditional healing programs in Canada, Australia, and New Zealand: a scoping review. Explore (NY).

[43] Ford JD, King N, Galappaththi EK, Pearce T, McDowell G, Harper SL (2020). The resilience of indigenous peoples to environmental change. One Earth.

[44] Petzold J, Andrews N, Ford JD, Hedemann C, Postigo JC (2020). Indigenous knowledge on climate change adaptation: a global evidence map of academic literature. Environ Res Lett.

[45] Kuhnlein HV, Chotiboriboon S (2022). Why and how to strengthen Indigenous Peoples’ food systems with examples from two unique Indigenous communities. Front Sustain Food Syst.

[46] Whyte K (2020). Too late for indigenous climate justice: ecological and relational tipping points. Wiley Interdiscip Rev Clim Change.

[47] Heke I, Rees D, Swinburn B, Waititi RT, Stewart A (2019). Systems thinking and indigenous systems: native contributions to obesity prevention. AlterNative Int J Indig Peoples.

[48] Merino R (2016). An alternative to ‘alternative development’?: buen vivir and human development in Andean countries. Oxf Dev Stud.

[49] Zavaleta C, Berrang-Ford L, Llanos-Cuentas A (2017). Indigenous Shawi communities and national food security support: right direction, but not enough. Food Policy.

[50] Curtis E, Jones R, Tipene-Leach D (2019). Why cultural safety rather than cultural competency is required to achieve health equity: a literature review and recommended definition. Int J Equity Health.

[51] Leal Filho W, Barbir J, Gwenzi J (2022). The role of indigenous knowledge in climate change adaptation in Africa. Environ Sci Policy.

[52] Nyong A, Adesina F, Osman Elasha B (2007). The value of indigenous knowledge in climate change mitigation and adaptation strategies in the African Sahel. Mitig Adapt Strategies Glob Change.

[53] Carmona R, Reed G, Ford J (2024). Indigenous Peoples’ rights in national climate governance: an analysis of Nationally Determined Contributions (NDCs). Ambio.

[54] Whyte KL, Talley JD, Gibson J (2019). Indigenous mobility traditions, colonialism, and the anthropocene. Mobilities.

[55] Oster RT, Grier A, Lightning R, Mayan MJ, Toth EL (2014). Cultural continuity, traditional Indigenous language, and diabetes in Alberta First Nations: a mixed methods study. Int J Equity Health.

[56] Vecchio EA, Dickson M, Zhang Y (2022). Indigenous mental health and climate change: systematic literature review. J Clim Change Health.

[57] Ninomiya MEM, Burns N, Pollock NJ (2023). Indigenous communities and the mental health impacts of land dispossession related to industrial resource development: a systematic review. Lancet Planet Health.

[58] Paredes M, Kaulard A (2020). Fighting the climate crisis in persistently unequal land regimes: natural protected areas in the Peruvian Amazon. J Clean Prod.

[59] Brugnach M, Craps M, Dewulf A (2017). Including indigenous peoples in climate change mitigation: addressing issues of scale, knowledge and power. Clim Change.

[60] Carmona R (2023). Global guidelines, local interpretations: ethnography of climate policy implementation in Mapuche territory, Southern Chile. Clim Policy.

[61] Vijayan D, Ludwig D, Rybak C (2022). Indigenous knowledge in food system transformations. Commun Earth Environ.

[62] Galappaththi EK, Ford JD, Bennett EM, Berkes F (2019). Climate change and community fisheries in the arctic: a case study from Pangnirtung, Canada. J Environ Manage.

[63] The Lancet (2023). Indigenous health: self-determination is key. Lancet.

[64] Minor K, Jensen ML, Hamilton L, Bendixen M, Lassen DD, Rosing MT (2023). Experience exceeds awareness of anthropogenic climate change in Greenland. Nat Clim Chang.

[65] Bussalleu A, Di-Liberto A, Carcamo C (2020). Cultural Values and the coliform bacterial load of “Masato,” an Amazon Indigenous beverage. EcoHealth.

[66] Keleman Saxena A, Cadima Fuentes X, Gonzales Herbas R, Humphries DL (2016). Indigenous food systems and climate change: impacts of climatic shifts on the production and processing of native and traditional crops in the bolivian andes. Front Public Health.

[67] Zavaleta-Cortijo C, Ford JD, Galappaththi EK (2023). Indigenous knowledge, community resilience, and health emergency preparedness. Lancet Planet Health.

[68] Arotoma Rojas I, Chichmana V, Anza-Ramirez C (2023). Policy recomendations from the COVID-19 Observatories in Indigenous Peoples for Peruvian stake holders.

[69] Frechette A, Ginsburg C, Walker W (2018). A global baseline of carbon storage in collective lands.

[70] Cottrell C (2022). Avoiding a new era in biopiracy: including indigenous and local knowledge in nature-based solutions to climate change. Environ Sci Policy.

[71] MacDonald JP, Ford J, Willox AC (2015). Youth-led participatory video as a strategy to enhance Inuit youth adaptive capacities for dealing with climate change. Arctic.

[72] Zavaleta C, Berrang-Ford L, Ford J (2018). Multiple non-climatic drivers of food insecurity reinforce climate change maladaptation trajectories among Peruvian Indigenous Shawi in the Amazon. PLoS One.

[73] Arotoma-Rojas I, Berrang-Ford L, Zavaleta-Cortijo C, Ford JD, Cooke P (2022). Indigenous Peoples’ perceptions of their food system in the context of climate change: a case study of Shawi Men in the Peruvian Amazon. Sustainability (Basel).

[74] International Work Group for Indigenous Affairs (2022). A new paradigm of climate partnership with Indigenous Peoples: an analysis of the recognition of Indigenous Peoples in the IPCC report on mitigation.

[75] Norton-Smith K, Lynn K, Chief K (2016). Climate change and indigenous peoples: a synthesis of current impacts and experiences.

[76] Salick J, Ross N, Hamin Infield EM, Abunnasr Y, Ryan RL (2018). Planning for climate change: a reader in green infrastructure and sustainable design for resilient cities.

[77] Abid Z, Abid M, Zafar Q, Mehmood S (2018). Detrimental effects of climate change on women. Earth Syst Environ.

[78] Ebi KL, Paulson JA (2007). Climate change and children. Pediatr Clin North Am.

[79] Hansen A, Bi L, Saniotis A, Nitschke M (2013). Vulnerability to extreme heat and climate change: is ethnicity a factor?. Glob Health Action.

[80] World Meteorological Organization (2023). WMO state of the global climate 2023.

[81] Chambers J (2020). Global and cross-country analysis of exposure of vulnerable populations to heatwaves from 1980 to 2018. Clim Change.

[82] Ebi KL, Capon A, Berry P (2021). Hot weather and heat extremes: health risks. Lancet.

[83] Chersich MF, Pham MD, Areal A (2020). Associations between high temperatures in pregnancy and risk of preterm birth, low birth weight, and stillbirths: systematic review and meta-analysis. BMJ.

[84] Sisodiya SM, Gulcebi MI, Fortunato F (2024). Climate change and disorders of the nervous system. Lancet Neurol.

[85] de Perez EC, van Aalst M, Bischiniotis K (2018). Global predictability of temperature extremes. Environ Res Lett.

[86] Chambers J (2020). Global and cross-country analysis of exposure of vulnerable populations to heatwaves from 1980 to 2018. Clim Change.

[87] Center for International Earth Science Information Network (2018). WorldPop.

[88] Hersbach H, Bell B, Berrisford P (2020). The ERA5 global reanalysis. Q J R Meteorol Soc.

[89] Klarenberg H, van der Velde JHPM, Peeters CFW (2024). Leisure time physical activity is associated with improved diastolic heart function and is partly mediated by unsupervised quantified metabolic health. BMJ Open Sport Exerc Med.

[90] Skurvydas A, Istomina N, Dadeliene R (2024). Mood profile in men and women of all ages is improved by leisure-time physical activity rather than work-related physical activity. BMC Public Health.

[91] Li Y, Tian C (2024). Does active transport create a win-win situation for environmental and human health: the moderating effect of leisure and tourism activity. Environ Sci Pollut Res Int.

[92] Vecellio DJ, Cottle RM, Tony Wolf S, Larry Kenney W (2023). Critical environmental limits for human thermoregulation in the context of a changing climate. Exerc Sport Mov.

[93] Kjellstrom T, Freyberg C, Lemke B, Otto M, Briggs D (2018). Estimating population heat exposure and impacts on working people in conjunction with climate change. Int J Biometeorol.

[94] Flouris AD, Dinas PC, Ioannou LG (2018). Workers’ health and productivity under occupational heat strain: a systematic review and meta-analysis. Lancet Planet Health.

[95] International Labour Organization (2019). Working on a warmer planet: the impact of heat stress on labour productivity and decent work.

[96] Liljegren JC, Carhart RA, Lawday P, Tschopp S, Sharp R (2008). Modeling the wet bulb globe temperature using standard meteorological measurements. J Occup Environ Hyg.

[97] Krause AJ, Simon EB, Mander BA (2017). The sleep-deprived human brain. Nat Rev Neurosci.

[98] Cappuccio FP, D’Elia L, Strazzullo P, Miller MA (2010). Sleep duration and all-cause mortality: a systematic review and meta-analysis of prospective studies. Sleep.

[99] Cappuccio FP, Cooper D, D’Elia L, Strazzullo P, Miller MA (2011). Sleep duration predicts cardiovascular outcomes: a systematic review and meta-analysis of prospective studies. Eur Heart J.

[100] Irwin MR (2015). Why sleep is important for health: a psychoneuroimmunology perspective. Annu Rev Psychol.

[101] Obradovich N, Migliorini R, Mednick SC, Fowler JH (2017). temperature and human sleep loss in a changing climate. Sci Adv.

[102] Mullins JT, White C (2019). Temperature and mental health: evidence from the spectrum of mental health outcomes. J Health Econ.

[103] Chevance G, Minor K, Vielma C (2024). A systematic review of ambient heat and sleep in a warming climate. Sleep Med Rev.

[104] Rifkin DI, Long MW, Perry MJ (2018). Climate change and sleep: a systematic review of the literature and conceptual framework. Sleep Med Rev.

[105] Obradovich N, Migliorini R (2018). Sleep and the human impacts of climate change. Sleep Med Rev.

[106] Minor K, Bjerre-Nielsen A, Jonasdottir SS, Lehmann S, Obradovich N (2022). Rising temperatures erode human sleep globally. One Earth.

[107] Cox DTC, Maclean IMD, Gardner AS, Gaston KJ (2020). Global variation in diurnal asymmetry in temperature, cloud cover, specific humidity and precipitation and its association with leaf area index. Glob Change Biol.

[108] Bressler RD, Moore FC, Rennert K (2021). Estimates of country level temperature-related mortality damage functions. Sci Rep.

[109] Gasparrini A, Guo Y, Sera F (2017). Projections of temperature-related excess mortality under climate change scenarios. Lancet Planet Health.

[110] Romanello M, McGushin A, Di Napoli C (2021). The 2021 report of the *Lancet* Countdown on health and climate change: code red for a healthy future. Lancet.

[111] Honda Y, Kondo M, McGregor G (2014). Heat-related mortality risk model for climate change impact projection. Environ Health Prev Med.

[112] Copernicus Climate Change Service (2019). Fire danger indices historical data from the Copernicus Emergency Management Service.

[113] Ben Clarke CB, Rodrigues R (2024). Climate change, not El Niño, main driver of exceptional drought in highly vulnerable Amazon River Basin.

[114] Otto Friederike EL, Clarke Ben, Rahimi Mohammad (2023). Human-induced climate change compounded by socio-economic water stressors increased severity of drought in Syria, Iraq and Iran.

[115] Stanke C, Kerac M, Prudhomme C, Medlock J, Murray V (2013). Health effects of drought: a systematic review of the evidence. PLoS Curr.

[116] Vins H, Bell J, Saha S, Hess JJ (2015). The mental health outcomes of drought: a systematic review and causal process diagram. Int J Environ Res Public Health.

[117] National Integrated Drought Information System Navigation and transportation.

[118] Food and Agriculture Organization of the United Nations (2023). The impact of disasters on agriculture and food security 2023—voiding and reducing losses through investment in resilience.

[119] Beguería S, Vicente-Serrano SM, Reig F, Latorre B (2014). Standardized precipitation evapotranspiration index (SPEI) revisited: parameter fitting, evapotranspiration models, tools, datasets and drought monitoring. Int J Climatol.

[120] Consejo Superior de Investigaciones Cientificas Global SPEI database.

[121] Allan RP, Arias PA, Armour K Intergovernmental Panel on Climate Change, 2021: summary for policymakers.

[122] Donat MG, Lowry AL, Alexander LV, O’Gorman PA, Maher N (2016). More extreme precipitation in the world’s dry and wet regions. Nat Clim Chang.

[123] Allen MR, Ingram WJ (2002). Constraints on future changes in climate and the hydrologic cycle. Nature.

[124] Trenberth KE, Dai A, Rasmussen RM, Parsons DB (2003). The changing character of precipitation. Bull Am Meteorol Soc.

[125] Saulnier DD, Brolin Ribacke K, von Schreeb J (2017). No calm after the storm: a systematic review of human health following flood and storm disasters. Prehosp Disaster Med.

[126] He C, Kim H, Hashizume M (2024). The overlooked health impacts of extreme rainfall exposure in 30 East Asian cities. Nat Sustain.

[127] Robin C, Beck C, Armstrong B, Waite TD, Rubin GJ, Oliver I (2020). Impact of flooding on health-related quality of life in England: results from the National Study of Flooding and Health. Eur J Public Health.

[128] Paterson DL, Wright H, Harris PNA (2018). Health risks of flood disasters. Clin Infect Dis.

[129] Ebi KL, Vanos J, Baldwin JW (2021). Extreme weather and climate change: population health and health system implications. Annu Rev Public Health.

[130] Myers S, Frumkin H (2020). Planetary health: protecting nature to protect ourselves.

[131] Prentice CM, Vergunst F, Minor K, Berry HL (2024). Education outcomes in the era of global climate change. Nat Clim Chang.

[132] Muñoz-Sabater J, Dutra E, Agustí-Panareda A (2021). ERA5-Land: a state-of-the-art global reanalysis dataset for land applications. Earth Syst Sci Data.

[133] Zhang X, Zhao L, Tong DQ, Wu G, Dan M, Teng B (2016). A systematic review of global desert dust and associated human health effects. Atmosphere.

[134] Tobias A, Karanasiou A, Amato F, Roqué M, Querol X (2019). Health effects of desert dust and sand storms: a systematic review and meta-analysis protocol. BMJ Open.

[135] Lwin KS, Tobias A, Chua PL (2023). Effects of desert dust and sandstorms on human health: a scoping review. Geohealth.

[136] Tong DQ, Wang JXL, Gill TE, Lei H, Wang B (2017). Intensified dust storm activity and Valley fever infection in the southwestern United States. Geophys Res Lett.

[137] Tong DQ, Gill TE, Sprigg WA (2023). Health and safety effects of airborne soil dust in the americas and beyond. Rev Geophys.

[138] Nickovic S, Cvetkovic B, Petković S (2021). Publisher correction: cloud icing by mineral dust and impacts to aviation safety. Sci Rep.

[139] Tong D, Feng I, Gill TE, Schepanski K, Wang J (2023). How many people were killed by windblown dust events in the United States?. Bull Am Meteorol Soc.

[140] Xian P, Reid JS, Ades M (2024). Intercomparison of aerosol optical depths from four reanalyses and their multi-reanalysis-consensus. Atmos Chem Phys.

[141] WHO (2021). WHO global air quality guidelines.

[142] Thompson R, Lawrance EL, Roberts LF (2023). Ambient temperature and mental health: a systematic review and meta-analysis. Lancet Planet Health.

[143] Obradovich N, Minor K (2022). Identifying and preparing for the mental health burden of climate change. JAMA Psychiatry.

[144] Obradovich N, Migliorini R, Paulus MP, Rahwan I (2018). Empirical evidence of mental health risks posed by climate change. Proc Natl Acad Sci USA.

[145] Nori-Sarma A, Sun S, Sun Y (2022). Association between ambient heat and risk of emergency department visits for mental health among US adults, 2010 to 2019. JAMA Psychiatry.

[146] Burke M, González F, Baylis P (2018). Higher temperatures increase suicide rates in the United States and Mexico. Nat Clim Chang.

[147] Baylis P, Obradovich N, Kryvasheyeu Y (2018). Weather impacts expressed sentiment. PLoS One.

[148] Semenza JC, Rocklöv J, Ebi KL (2022). Climate change and cascading risks from infectious disease. Infect Dis Ther.

[149] Mora C, McKenzie T, Gaw IM (2022). Over half of known human pathogenic diseases can be aggravated by climate change. Nat Clim Chang.

[150] Stanaway JD, Shepard DS, Undurraga EA (2016). The global burden of dengue: an analysis from the Global Burden of Disease Study 2013. Lancet Infect Dis.

[151] Bhatt S, Gething PW, Brady OJ (2013). The global distribution and burden of dengue. Nature.

[152] Clarke J, Lim A, Gupte P, Pigott DM, van Panhuis WG, Brady OJ (2024). A global dataset of publicly available dengue case count data. Sci Data.

[153] WHO Dengue—global situation.

[154] Colón-González FJ, Sewe MO, Tompkins AM (2021). Projecting the risk of mosquito-borne diseases in a warmer and more populated world: a multi-model, multi-scenario intercomparison modelling study. Lancet Planet Health.

[155] DiSera L, Sjödin H, Rocklöv J (2020). The mosquito, the virus, the climate: an unforeseen Réunion in 2018. Geohealth.

[156] Metelmann S, Caminade C, Jones AE, Medlock JM, Baylis M, Morse AP (2019). The UK’s suitability for *Aedes albopictus* in current and future climates. J R Soc Interface.

[157] Grover-Kopec EK, Blumenthal MB, Ceccato P, Dinku T, Omumbo JA, Connor SJ (2006). Web-based climate information resources for malaria control in Africa. Malar J.

[158] Dupke S, Buchholz U, Fastner J (2023). Impact of climate change on waterborne infections and intoxications. J Health Monit.

[159] Heidecke J, Lavarello Schettini A, Rocklöv J (2023). West Nile virus eco-epidemiology and climate change. PLOS Clim.

[160] Erazo D, Grant L, Ghisbain G (2024). Contribution of climate change to the spatial expansion of West Nile virus in Europe. Nat Commun.

[161] Shocket MS, Verwillow AB, Numazu MG (2020). Transmission of West Nile and five other temperate mosquito-borne viruses peaks at temperatures between 23°C and 26°C. eLife.

[162] Food and Agriculture Organization of the United Nations, International Fund for Agricultural Development, UNICEF, World Food Programme, WHO (2023). The state of food security and nutrition in the world 2023.

[163] Food and Agriculture Organization of the United Nations (2020). Climate change: unpacking the burden on food safety.

[164] Food and Agriculture Organization of the United Nations (2021). The impact of disasters and crises on agriculture and food security.

[165] Dasgupta S, Robinson EJZ (2023). Climate, weather and child health in Burkina Faso. Aust J Agric Resour Econ.

[166] Dasgupta S, Robinson EJZ (2021). Improving food policies for a climate insecure world: evidence from Ethiopia. Natl Inst Econ Rev.

[167] Dasgupta S, Robinson EJZ (2022). Attributing changes in food insecurity to a changing climate. Sci Rep.

[168] Hutchinson RN, Shin S (2014). Systematic review of health disparities for cardiovascular diseases and associated factors among American Indian and Alaska Native populations. PLoS One.

[169] Ghosh-Jerath S, Kapoor R, Ghosh U, Singh A, Downs S, Fanzo J (2021). Pathways of climate change impact on agroforestry, food consumption pattern, and dietary diversity among Indigenous subsistence farmers of Sauria Paharia Tribal community of India: a mixed methods study. Front Sustain Food Syst.

[170] Shrestha RP, Nepal N (2016). An assessment by subsistence farmers of the risks to food security attributable to climate change in Makwanpur, Nepal. Food Secur.

[171] Cafiero C, Viviani S, Nord M (2018). Food security measurement in a global context: the food insecurity experience scale. Measurement.

[172] Ballard T, Kepple A, Cafiero C (2013). The food insecurity experience scale: development of a global standard for monitoring hunger worldwide.

[173] Ballard T, Kepple A, Cafiero C (2014). Better measurement of food insecurity in the context of enhancing nutrition. Ernahr-Umsch.

[174] Copernicus Climate Change Service (2021). ORAS5 global ocean reanalysis monthly data from 1958 to present.

[175] Romanello M, Di Napoli C, Drummond P (2022). The 2022 report of the *Lancet* Countdown on health and climate change: health at the mercy of fossil fuels. Lancet.

[176] United Nations Framework Convention on Climate Change (2023). Outcome of the first Global Stocktake.

[177] Intergovernmental Panel on Climate Change (2022). Climate change 2022: impacts, adaptation, and vulnerability Contribution of Working Group II to the Sixth Assessment Report of the Intergovernmental Panel on Climate Change.

[178] United Nations Framework Convention on Climate Change (2015). Paris Agreement.

[179] United Nations Framework Convention on Climate Change (2023). Glasgow–Sharm el-Sheikh work programme on the global goal on adaptation referred to in decision 7/CMA 3 2023.

[180] WHO (2022). Alliance for Transformative Action on Climate and Health.

[181] WHO (2021). Criterios de calidad para los planes nacionales de adaptación de la salud.

[182] World Bank Group (2023). Urban development overview.

[183] Carbon Disclosure Project (2024). 2023 cities climate risk and vulnerability assessments.

[184] World Meteorological Organization (2023). 2023 state of climate services: health.

[185] Bouchama A, Dehbi M, Mohamed G, Matthies F, Shoukri M, Menne B (2007). Prognostic factors in heat wave related deaths: a meta-analysis. Arch Intern Med.

[186] International Energy Agency (2023). Sustainable, affordable cooling can save tens of thousands of lives each year.

[187] Salamanca F, Georgescu M, Mahalov A, Moustaoui M, Wang M (2014). Anthropogenic heating of the urban environment due to air conditioning. J Geophys Res Atmos.

[188] Randazzo T, De Cian E, Mistry MN (2020). Air conditioning and electricity expenditure: the role of climate in temperate countries. Econ Model.

[189] Cohen-Shacham E, Walters G, Janzen C, Maginnis S (2016). Nature-based solutions to address global societal challenges.

[190] Sun J, Wang Y, Lee TM (2024). Nature-based solutions can help restore degraded grasslands and increase carbon sequestration in the Tibetan Plateau. Commun Earth Environ.

[191] Fischer HW, Chhatre A, Duddu A, Pradhan N, Agrawal A (2023). Community forest governance and synergies among carbon, biodiversity and livelihoods. Nat Clim Chang.

[192] Fournet F, Simard F, Fontenille D (2024). Green cities and vector-borne diseases: emerging concerns and opportunities. Euro Surveill.

[193] Plieninger T, Muñoz-Rojas J, Buck LE, Scherr SJ (2020). Agroforestry for sustainable landscape management. Sustain Sci.

[194] Mbow C, Van Noordwijk M, Luedeling E, Neufeldt H, Minang PA, Kowero G (2014). Agroforestry solutions to address food security and climate change challenges in Africa. Curr Opin Environ Sustain.

[195] Chen B, Wu S, Song Y, Webster C, Xu B, Gong P (2022). Contrasting inequality in human exposure to greenspace between cities of Global North and Global South. Nat Commun.

[196] Callaghan A, McCombe G, Harrold A (2021). The impact of green spaces on mental health in urban settings: a scoping review. J Ment Health.

[197] Frumkin H, Bratman GN, Breslow SJ (2017). Nature Contact and Human Health: A Research Agenda. Environ Health Perspect.

[198] Garnett ST, Burgess ND, Fa JE (2018). A spatial overview of the global importance of Indigenous lands for conservation. Nat Sustain.

[199] Iungman T, Cirach M, Marando F (2023). Cooling cities through urban green infrastructure: a health impact assessment of European cities. Lancet.

[200] Gago EJ, Roldan J, Pacheco-Torres R, Ordóñez J (2013). The city and urban heat islands: a review of strategies to mitigate adverse effects. Renew Sustain Energy Rev.

[201] Green D, O’Donnell E, Johnson M (2021). Green infrastructure: the future of urban flood risk management?. Wiley Interdiscip Rev Clim Change.

[202] Kim HY (2021). Analyzing green space as a flooding mitigation—storm Chaba case in South Korea. Geomatics Nat Hazards Risk.

[203] Cariñanos P, Casares-Porcel M (2011). Urban green zones and related pollen allergy: a review. Some guidelines for designing spaces with low allergy impact. Landsc Urban Plan.

[204] Magnan AK, Bell R, Duvat VKE (2023). Status of global coastal adaptation. Nat Clim Chang.

[205] Intergovernmental Panel on Climate Change (2022). FAQ 4: How are people adapting to the effects of climate change and what are the known limits to adaptation? Climate Change 2022: impacts, adaptation and vulnerability.

[206] McMichael AJ (2012). Insights from past millennia into climatic impacts on human health and survival. Proc Natl Acad Sci USA.

[207] Burke KD, Williams JW, Chandler MA, Haywood AM, Lunt DJ, Otto-Bliesner BL (2018). Pliocene and Eocene provide best analogs for near-future climates. Proc Natl Acad Sci USA.

[208] Vanos J, Guzman-Echavarria G, Baldwin JW, Bongers C, Ebi KL, Jay O (2023). A physiological approach for assessing human survivability and liveability to heat in a changing climate. Nat Commun.

[209] Sherwood SC, Huber M (2010). An adaptability limit to climate change due to heat stress. Proc Natl Acad Sci USA.

[210] Vanos J, Guzman-Echavarria G, Baldwin JW, Bongers C, Ebi KL, Jay O (2023). A physiological approach for assessing human survivability and liveability to heat in a changing climate. Nat Commun.

[211] Vecellio DJ, Tony Wolf S, Cottle RM, Larry Kenney W (1985). Evaluating the 35°C wet-bulb temperature adaptability threshold for young, healthy subjects (PSU HEAT Project. J Appl Physiol.

[212] United Nations Framework Convention on Climate Change (2024). Green climate fund.

[213] Amerasinghe N, Thwaites J, Larsen G, Ballesteros A (2017). The future of the funds: exploring the architecture of multilateral climate finance.

[214] Green Climate Fund (2023). SAP030: Strengthening climate resilience of the Lao People’s Democratic Republic (PDR) health system.

[215] WHO (2005). International Health Regulations.

[216] World Health Organization (2024). States party self-assessment annual reporting tool.

[217] Fox M, Zuidema C, Bauman B, Burke T, Sheehan M (2019). Integrating public health into climate change policy and planning: state of practice update. Int J Environ Res Public Health.

[218] Sorensen C, Campbell H, Depoux A (2023). Core competencies to prepare health professionals to respond to the climate crisis. PLoS Clim.

[219] Rocklöv J, Tozan Y (2019). Climate change and the rising infectiousness of dengue. Emerg Top Life Sci.

[220] Kraemer MUG, Reiner RC, Brady OJ (2019). Past and future spread of the arbovirus vectors *Aedes aegypti* and *Aedes albopictus*. Nat Microbiol.

[221] US Centers for Disease Control and Prevention (2024). Data and statistics on dengue in the United States.

[222] WHO (2023). Dengue and severe dengue.

[223] Intergovernmental Panel on Climate Change (2023). Climate change 2021: the physical science basis. Contribution of Working Group I to the Sixth Assessment Report of the Intergovernmental Panel on Climate Change.

[224] Ebi KL, Schmier JK (2005). A stitch in time: improving public health early warning systems for extreme weather events. Epidemiol Rev.

[225] WHO (2021). 2021 WHO health and climate change survey report.

[226] Kulp SA, Strauss BH (2019). New elevation data triple estimates of global vulnerability to sea-level rise and coastal flooding. Nat Commun.

[227] Hauer ME, Fussell E, Mueller V (2019). Sea-level rise and human migration. Nat Rev Earth Environ.

[228] Martyr-Koller R, Thomas A, Schleussner C-F, Nauels A, Lissner T (2021). Loss and damage implications of sea-level rise on Small Island Developing States. Curr Opin Environ Sustain.

[229] WHO (2023). Universal health coverage (UHC).

[230] Whitmee S, Green R, Belesova K (2024). Pathways to a healthy net-zero future: report of the *Lancet* Pathfinder Commission. Lancet.

[231] United Nations Environment Programme (2023). Emissions gap report 2023: broken record—temperatures hit new highs, yet world fails to cut emissions (again).

[232] Smith KR, Frumkin H, Balakrishnan K (2013). Energy and human health. Annu Rev Public Health.

[233] Cho KS, Lee SH (1978). Occupational health hazards of mine workers. Bull World Health Organ.

[234] Hendryx M, Zullig KJ, Luo J (2020). Impacts of coal use on health. Annu Rev Public Health.

[235] Landrigan PJ, Fuller R, Acosta NJR (2018). The *Lancet* Commission on pollution and health. Lancet.

[236] McKenzie LM, Witter RZ, Newman LS, Adgate JL (2012). Human health risk assessment of air emissions from development of unconventional natural gas resources. Sci Total Environ.

[237] Colborn T, Kwiatkowski C, Schultz K, Bachran M (2011). Hazard assessment articles natural gas operations from a public health perspective. Hum Ecol Risk Assess.

[238] Witter RZ, Tenney L, Clark S, Newman LS (2014). Occupational exposures in the oil and gas extraction industry: state of the science and research recommendations. Am J Ind Med.

[239] O’Callaghan-Gordo C, Orta-Martínez M, Kogevinas M (2016). Health effects of non-occupational exposure to oil extraction. Environ Health.

[240] Balmes JR, Holm SM, McCormack MC, Hansel NN, Gerald LB, Krishnan JA (2023). Cooking with natural gas: just the facts, please. Am J Respir Crit Care Med.

[241] Karanikas N, Steele S, Bruschi K (2021). Occupational health hazards and risks in the wind industry. Energy Rep.

[242] Duroha JC, Brownson JRS, Macht GA, Middleton P, Hebert E, Bortman D, Foster R, Cipolla C, Rixham C (2020). Occupational risks associated with solar installations: a review.

[243] Calder RSD, Schartup AT, Li M, Valberg AP, Balcom PH, Sunderland EM (2016). Future impacts of hydroelectric power development on methylmercury exposures of Canadian Indigenous communities. Environ Sci Technol.

[244] Banerjee S, Ghosh S, Bhowmick GD, Pandey D, Pandey BK, Dadheech P (2023). Handbook of research on safe disposal methods of municipal solid wastes for a sustainable environment.

[245] Occupational Safety and Health Administration, US Department of Labor Green job hazards—biofuels: toxicity hazards.

[246] Roundtable on Environmental Health Sciences, Research, and Medicine, Board on Population Health and Public Health Practice, Institute of Medicine (2014). Occupational health and biofuels production.

[247] Bakhiyi B, Labrèche F, Zayed J (2014). The photovoltaic industry on the path to a sustainable future—environmental and occupational health issues. Environ Int.

[248] Chen S, Zhang Q, Andrews-Speed P, Mclellan B (2020). Quantitative assessment of the environmental risks of geothermal energy: a review. J Environ Manage.

[249] Occupational Safety and Health Administration, US Department of Labor Green job hazards—hydrogen fuel cells.

[250] Nayar J (2021). Not so “green” technology: the complicated legacy of rare earth mining.

[251] Oladipo HJ, Tajudeen YA, Taiwo EO (2023). Global environmental health impacts of rare earth metals: insights for research and policy making in Africa. Challenges.

[252] Organisation for Economic Co-operation and Development (2020). Reducing the health risks of the copper, rare earth and cobalt industries: transition to a circular low-carbon economy.

[253] Chen Y, Kang Y, Zhao Y (2021). A review of lithium-ion battery safety concerns: the issues, strategies, and testing standards. J Energy Chem.

[254] Canadian Centre for Occupational Health and Safety Battery charging—lithium-ion batteries.

[255] Nain P, Kumar A (2020). Ecological and human health risk assessment of metals leached from end-of-life solar photovoltaics. Environ Pollut.

[256] Chen J, Li C, Ristovski Z (2017). A review of biomass burning: emissions and impacts on air quality, health and climate in China. Sci Total Environ.

[257] Knopper LD, Ollson CA (2011). Health effects and wind turbines: a review of the literature. Environ Health.

[258] Knopper LD, Ollson CA, McCallum LC (2014). Wind turbines and human health. Front Public Health.

[259] International Atomic Energy Agency (1999). Health and environmental impacts of electricity generation systems.

[260] Edwards MW, Schweitzer RD, Shakespeare-Finch J, Byrne A, Gordon-King K (2019). Living with nuclear energy: a systematic review of the psychological consequences of nuclear power. Energy Res Soc Sci.

[261] International Energy Agency (2023). Energy statistics data browser.

[262] International Renewable Energy Agency (2023). Tracking energy transition progress.

[263] International Energy Agency (2024). CO2 Emissions in 2023.

[264] International Energy Agency (2023). World energy outlook 2023.

[265] Cozzi L, Wetzel D, Tonolo G, Diarra N, Roge A (2023). Access to electricity improves slightly in 2023, but still far from the pace needed to meet SDG7.

[266] WHO (2016). Burning opportunity: clean household energy for health, sustainable development, and wellbeing of women and children.

[267] International Energy Agency, International Renewable Energy Agency, UN Statistics Division, World Bank, WHO (2023). Tracking SDG7: the energy progress report 2023.

[268] International Energy Agency (2023). Energy system: transport.

[269] International Energy Agency (2024). Global EV outlook 2024.

[270] Woodcock J, Khreis H, Goel R (2022). Transport and health on the path to a net zero carbon world. BMJ.

[271] GBD 2019 Risk Factors Collaborators (2020). Global burden of 87 risk factors in 204 countries and territories, 1990–2019: a systematic analysis for the Global Burden of Disease Study 2019. Lancet.

[272] Burnett RT, Spadaro JV, Garcia GR, Pope CA (2022). Designing health impact functions to assess marginal changes in outdoor fine particulate matter. Environ Res.

[273] Lelieveld J, Haines A, Burnett R (2023). Air pollution deaths attributable to fossil fuels: observational and modelling study. BMJ.

[274] United Nations Department of Economic and Social Affairs Sustainable Development Goal 7: ensure access to affordable, reliable, sustainable and modern energy for all.

[275] WHO (2018). Global database of household air pollution measurements.

[276] Mohajeri N, Hsu SC, Milner J (2023). Urban-rural disparity in global estimation of PM_2·5_ household air pollution and its attributable health burden. Lancet Planet Health.

[277] Klepeis NE, Nelson WC, Ott WR (2001). The National Human Activity Pattern Survey (NHAPS): a resource for assessing exposure to environmental pollutants. J Expo Anal Environ Epidemiol.

[278] Liu Y, Ma H, Zhang N, Li Q (2022). A systematic literature review on indoor PM_2.5_ concentrations and personal exposure in urban residential buildings. Heliyon.

[279] WHO (2021). WHO global air quality guidelines: particulate matter (PM2.5 and PM10), ozone, nitrogen dioxide, sulfur dioxide and carbon monoxide.

[280] Li M, Jia N, Lenzen M (2022). Global food-miles account for nearly 20% of total food-systems emissions. Nat Food.

[281] Willett W, Rockström J, Loken B (2019). Food in the Anthropocene: the EAT-Lancet Commission on healthy diets from sustainable food systems. Lancet.

[282] Springmann M, Wiebe K, Mason-D’Croz D, Sulser TB, Rayner M, Scarborough P (2018). Health and nutritional aspects of sustainable diet strategies and their association with environmental impacts: a global modelling analysis with country-level detail. Lancet Planet Health.

[283] Bechthold A, Boeing H, Schwedhelm C (2019). Food groups and risk of coronary heart disease, stroke and heart failure: a systematic review and dose-response meta-analysis of prospective studies. Crit Rev Food Sci Nutr.

[284] Di Angelantonio E, Bhupathiraju SN, Wormser D (2016). Body-mass index and all-cause mortality: individual-participant-data meta-analysis of 239 prospective studies in four continents. Lancet.

[285] Springmann M, Spajic L, Clark MA (2020). The healthiness and sustainability of national and global food based dietary guidelines: modelling study. BMJ.

[286] WHO (2023). The diet impact assessment model: a tool for analyzing the health, environmental and affordability implications of dietary change.

[287] Springmann M, Freund F (2022). Options for reforming agricultural subsidies from health, climate, and economic perspectives. Nat Commun.

[288] Springmann M, Godfray HCJ, Rayner M, Scarborough P (2016). Analysis and valuation of the health and climate change cobenefits of dietary change. Proc Natl Acad Sci USA.

[289] Springmann M, Clark M, Mason-D’Croz D (2018). Options for keeping the food system within environmental limits. Nature.

[290] Konijnendijk C, Devkota D, Mansourian S, Wildburger C (2023). Forests and trees for human health: pathways, impacts, challenges and response options.

[291] Turner-Skoff JB, Cavender N (2019). The benefits of trees for livable and sustainable communities. Plants People Planet.

[292] Karjalainen E, Sarjala T, Raitio H (2010). Promoting human health through forests: overview and major challenges. Environ Health Prev Med.

[293] Hansen MC, Potapov PV, Moore R (2013). High-resolution global maps of 21st-century forest cover change. Science.

[294] Curtis PG, Slay CM, Harris NL, Tyukavina A, Hansen MC (2018). Classifying drivers of global forest loss. Science.

[295] Fa JE, Watson JEM, Leiper I (2020). Importance of Indigenous Peoples’ lands for the conservation of intact forest landscapes. Front Ecol Environ.

[296] Organisation for Economic Co-operation and Development Climate finance and the USD 100 billion goal.

[297] United Nations Framework Convention on Climate Change New Collective Quantified Goal on Climate Finance.

[298] Kotz M, Levermann A, Wenz L (2024). The economic commitment of climate change. Nature.

[299] Day J, Chin N, Sydnor S, Widhalm M, Shah KU, Dorworth L (2021). Implications of climate change for tourism and outdoor recreation: an Indiana, USA, case study. Clim Change.

[300] Yu DD, Matthews L, Scott D, Li S, Guo ZY (2022). Climate suitability for tourism in China in an era of climate change: a multiscale analysis using holiday climate index. Curr Issues Tour.

[301] United Nations Framework Convention on Climate Change (2023). Fund for responding to loss and damage.

[302] United Nations Framework Convention on Climate Change (2024). COP 28: what was achieved and what happens next?.

[303] The Lancet (2023). Health Day at COP28: a hard-won (partial) gain. Lancet.

[304] International Labour Organization (2024). Statistics on Wages.

[305] Hurst P, Termine P, Kar M (2007). Agricultural workers and their contributions to sustainable agriculture and rural development.

[306] Rodriguez L, Singer C (2018). Too many agricultural workers can’t afford to eat, UN Says.

[307] World Bank (2022). Living standards measurement study: agricultural labor.

[308] Finkelman RB, Wolfe AL, Hendryx MS (2020). The future environmental and health impacts of coal. Energy Geoscience.

[309] Johnston JE, Lim E, Roh H (2019). Impact of upstream oil extraction and environmental public health: a review of the evidence. Sci Total Environ.

[310] International Energy Agency (2021). Net zero by 2050—a roadmap for the global energy sector.

[311] Rogelj J, den Elzen M, Höhne N (2016). Paris Agreement climate proposals need a boost to keep warming well below 2°C. Nature.

[312] Tanaka K, O’Neill BC (2018). The Paris Agreement zero-emissions goal is not always consistent with the 1.5°c and 2°c temperature targets. Nat Clim Chang.

[313] Oh TH (2010). Carbon capture and storage potential in coal-fired plant in Malaysia—a review. Renew Sustain Energy Rev.

[314] Jakob M, Steckel JC, Jotzo F (2020). The future of coal in a carbon-constrained climate. Nat Clim Chang.

[315] McMullin B, Price P, Jones MB, McGeever AH (2020). Assessing negative carbon dioxide emissions from the perspective of a national “fair share” of the remaining global carbon budget. Mitig Adapt Strategies Glob Change.

[316] Zhang N, Pang J (2022). The economic impacts of introducing CCER trading and offset mechanism into the national carbon market of China. Climate Change Research.

[317] Monjon S, Quirion P (2011). A border adjustment for the EU ETS: reconciling WTO rules and capacity to tackle carbon leakage. Clim Policy.

[318] Li W, Lu C, Ding Y, Zhang YW (2017). The impacts of policy mix for resolving overcapacity in heavy chemical industry and operating national carbon emission trading market in China. Appl Energy.

[319] Stadler K, Wood R, Bulavskaya T (2018). EXIOBASE 3: developing a time series of detailed environmentally extended multi-regional input-output tables. J Ind Ecol.

[320] He K, Mi Z, Zhang J, Li J, Coffman D (2023). The polarizing trend of regional CO2 emissions in China and its implications. Environ Sci Technol.

[321] Ekins P, Drummond P, Scamman D, Paroussos L, Keppo I (2022). The 1.5°C climate and energy scenarios: impacts on economic growth. Oxford Open Energy.

[322] International Energy Agency (2024). World energy investment 2024.

[323] Climate Safe Pensions (2023). National Academy of Medicine commits to fossil fuel divestment, massive momentum for global divestment movement.

[324] Stiglitz JE (2019). Addressing climate change through price and non-price interventions. Eur Econ Rev.

[325] Zapf M, Pengg H, Weindl C (2019). How to comply with the Paris Agreement temperature goal: global carbon pricing according to carbon budgets. Energies.

[326] UN, Inter-agency Task Force on Financing for Development (2020). Financing for sustainable development report 2020.

[327] Rainforest Action Network, BankTrack, Indigenous Environmental Network (2023). Banking on climate chaos: fossil fuel finance report 2023.

[328] Stocker M, Baffes J, Vorisek D (2018). What triggered the oil price plunge of 2014–2016 and why it failed to deliver an economic impetus in eight charts.

[329] Aguila N, Wullweber J (2024). Greener and cheaper: green monetary policy in the era of inflation and high interest rates. Eurasian Economic Review.

[330] Patz JA, Gibbs HK, Foley JA, Rogers JV, Smith KR (2007). Climate change and global health: quantifying a growing ethical crisis. EcoHealth.

[331] Chancel L, Bothe P, Voituriez T (2023). Climate inequality report 2023.

[332] Jackson RB, Friedlingstein P, Andrew RM, Canadell JG, Le Quéré C, Peters GP (2019). Persistent fossil fuel growth threatens the Paris Agreement and planetary health. Environ Res Lett.

[333] Rauner S, Bauer N, Dirnaichner A, Van Dingenen R, Mutel C, Luderer G (2020). Coal-exit health and environmental damage reductions outweigh economic impacts. Nat Clim Change.

[334] Pan X, den Elzen M, Höhne N, Teng F, Wang L (2017). Exploring fair and ambitious mitigation contributions under the Paris Agreement goals. Environ Sci Policy.

[335] Falkner R (2016). The Paris Agreement and the new logic of international climate politics. Int Aff.

[336] Schmidt A, Ivanova A, Schäfer MS (2013). Media attention for climate change around the world: a comparative analysis of newspaper coverage in 27 countries. Glob Environ Change.

[337] Weathers MR, Kendall BE (2016). Developments in the framing of climate change as a public health issue in US newspapers. Environ Commun.

[338] Langer AI, Gruber JB (2021). Political agenda setting in the hybrid media system: why legacy media still matter a great deal. Int J Press/Polit.

[339] Giles J (2005). Internet encyclopaedias go head to head. Nature.

[340] Similarweb Top websites ranking—most visited websites in February 2024.

[341] Wikipedia Commons contributors (2023). Wikipedia page views by language over time.

[342] Bornmann L (2011). Scientific peer review. Annu Rev Inform Sci Tech.

[343] Watts N, Amann M, Ayeb-Karlsson S (2017). The *Lancet* Countdown on health and climate change: from 25 years of inaction to a global transformation for public health. Lancet.

[344] Arasaradnam RP, Hillman T (2022). Climate change and health research—lessons from COP26. Clin Med.

[345] Berrang-Ford L, Sietsma AJ, Callaghan M (2021). Systematic mapping of global research on climate and health: a machine learning review. Lancet Planet Health.

[346] Callaghan M, Schleussner CF, Nath S (2021). Machine-learning-based evidence and attribution mapping of 100,000 climate impact studies. Nat Clim Chang.

[347] Dasandi N, Graham H, Lampard P, Mikhaylov SJ (2021). Intergovernmental engagement on health impacts of climate change. Bull World Health Organ.

[348] Abbas A, Ekowati D, Suhariadi F, Fenitra RM, Chatterjee U, Oyilieze Akanwa A, Kumar S, Kumar Singh S, Dutta Roy A (2022). Ecological footprints of climate change.

[349] Baturo A, Dasandi N, Mikhaylov SJ (2017). Understanding state preferences with text as data: introducing the UN general debate corpus. Research and Politics.

[350] Chelotti N, Dasandi N, Jankin Mikhaylov S (2022). Do intergovernmental organizations have a socialization effect on member state preferences? Evidence from the UN General Debate. Int Stud Q.

[351] UN (2023). Zimbabwe—president addresses General Debate.

[352] UN (2023). Fiji: his Excellency Sitiveni Ligamamada Rabuka, Prime Minister.

[353] Dasandi N, Graham H, Lampard P, Jankin Mikhaylov S (2021). Engagement with health in national climate change commitments under the Paris Agreement: a global mixed-methods analysis of the nationally determined contributions. Lancet Planet Health.

[354] Vogt-Schilb A, Hallegatte S (2017). Climate policies and nationally determined contributions: reconciling the needed ambition with the political economy. Wiley Interdiscip Rev Energy Environ.

[355] Kural E, Dellmuth LM, Gustafsson MT (2021). International organizations and climate change adaptation: a new dataset for the social scientific study of adaptation, 1990–2017. PLoS One.

[356] Dörfler T, Heinzel M (2023). Greening global governance: INGO secretariats and environmental mainstreaming of IOs, 1950 to 2017. Rev Int Organ.

[357] Maria DL, Maria-Therese G, Ece K (2020). Global adaptation governance: explaining the governance responses of international organizations to new issue linkages. Environ Sci Policy.

[358] Ecker-Ehrhardt M, Bjola C, Zaioti R (2020). Digital diplomacy and international organisations: autonomy, legitimacy and contestation.

[359] Goritz A, Schuster J, Jörgens H, Kolleck N (2022). International public administrations on twitter: a comparison of digital authority in global climate policy. J Comp Policy Anal.

[360] Carbon Majors (2024). The Carbon Majors database: launch report.

[361] Voegtlin C, Pless NM, Maak T, Pless NM, Orlitzky M, Sandhu S (2021). The Routledge companion to corporate social responsibility.

[362] Nicolo’ G, Zampone G, De Iorio S, Sannino G (2024). Does SDG disclosure reflect corporate underlying sustainability performance? Evidence from UN Global Compact participants. J Int Financ Manag Account.

[363] Msiska M, Ng A, Kimmel RK (2021). Doing well by doing good with the performance of United Nations Global Compact climate change champions. Humanit Soc Sci Commun.

[364] UN, COP28 UAE Climate health-ministerial.

[365] WHO (2023). COP28 Health Day.

[366] Patterson JJ (2023). Backlash to climate policy. Glob Environ Polit.

[367] Paterson M, Wilshire S, Tobin P (2023). The rise of anti-net zero populism in the UK: comparing rhetorical strategies for climate policy dismantling. J Comp Policy Anal: Res Pract.

[368] Atkins E (2022). ‘Bigger than Brexit’: exploring right-wing populism and net-zero policies in the United Kingdom. Energy Res Soc Sci.

[369] Kukowski CA, Garnett EE (2023). Tackling inequality is essential for behaviour change for net zero. Nat Clim Change.

