# Supplementary material for: The 2024 report of the Lancet Countdown on health and climate change: facing record-breaking threats from delayed action
Source: Lancet. Author manuscript; Available in PMC 2024 Nov 16. (PMC7616816; doi:10.1016/S0140-6736(24)01822-1)
Supplement: Supplementary appendix [file EMS200006-supplement-Supplementary_appendix.pdf]

THE LANCET

Supplementary appendix

This appendix formed part of the original submission and has been peer reviewed. We post it as supplied by the authors.

Supplement to: Romanello M, Walawender M, Hsu S-C, et al. The 2024 report of the Lancet Countdown on health and climate change: facing record-breaking threats from delayed action. *Lancet* 2024; published online Oct 30. https://doi.org/10.1016/ S0140-6736(24)01822-1.

The 2024 Report of the *Lancet* Countdown on Health and Climate Change

Appendix

Contents

The 2024 Report of the *Lancet* Countdown on Health and Climate Change ... 4
Section 1: Health Hazards, Exposures, and Impact ... 10
1.1: Health and heat ... 10
Indicator 1.1.1: exposure of vulnerable populations to heatwaves ... 10
Indicator 1.1.2: heat and physical activity ... 17
Indicator 1.1.3: change in labour capacity ... 22
Indicator 1.1.4: sleep loss from higher nighttime temperatures ... 38
Indicator 1.1.5: heat-related mortality ... 43
1.2: Health and extreme weather-related events ... 47
Indicator 1.2.1: wildfires ... 47
Indicator 1.2.2: drought ... 59
Indicator 1.2.3: extreme precipitation ... 62
Indicator 1.2.4: sand and dust storms ... 65
Indicator 1.2.5: extreme weather and sentiment ... 66
1.3: Climate suitability for infectious disease transmission ... 72
Indicator 1.3.1: dengue ... 72
Indicator 1.3.2: malaria ... 75
Indicator 1.3.3: vibrio ... 84
Indicator 1.3.4: West Nile virus ... 92
Indicator 1.4: Food security and undernutrition ... 99
Section 2: Adaptation, Planning, and Resilience for Health ... 120
2.1: Assessment and planning of health adaptation ... 120
Indicator 2.1.1: national assessments of climate change impacts, vulnerability, and adaptation for health 120
Indicator 2.1.2: national adaptation plans for health ... 125
Indicator 2.1.3: city-level climate change risk assessments ... 130
2.2: Enabling conditions, adaptation delivery, and implementation ... 135
Indicator 2.2.1: climate information for health ... 135
Indicator 2.2.2: benefits and harms of air conditioning ... 136
Indicator 2.2.3: urban green space ... 148
Indicator 2.2.4: global multilateral funding for health adaptation programmes ... 161
Indicator 2.2.5: detection, preparedness, and response to health emergencies ... 162
Indicator 2.2.6: climate and health education and training ... 168
2.3: Vulnerabilities, health risk, and resilience to climate change ... 176
Indicator 2.3.1: vulnerability to severe mosquito-borne disease ... 176
Indicator 2.3.2: lethality of extreme weather events ... 180
Indicator 2.3.3: rising sea levels, migration, and displacement ... 182
Section 3: Mitigation Actions and Health Co-Benefits ... 187
3.1: Energy use, energy generation, and health ... 187
Indicator 3.1.1: energy systems and health ... 187
Indicator 3.1.2: household energy use ... 191
Indicator 3.1.3: sustainable and healthy road transport ... 193
3.2: Air quality and health co-benefits ... 194
Indicator 3.2.1: mortality from ambient air pollution by sector ... 194
Indicator 3.2.2: household air pollution ... 197
3.3: Food, agriculture, and health co-benefits ... 199
Indicator 3.3.1: emissions from agricultural production and consumption ... 199
Indicator 3.3.2: diet and health co-benefits ... 203
Indicator 3.4: tree cover loss ... 210
Indicator 3.5: healthcare sector emissions and harms ... 212
Section 4: Economics and Finance ... 214
4.1: The economic impact of climate change and its mitigation ... 214
Indicator 4.1.1: economic losses due to weather-related extreme events ... 214
Indicator 4.1.2: costs of heat-related mortality ... 217
Indicator 4.1.3: loss of earnings from heat-related labour capacity reduction ... 221
Indicator 4.1.4: costs of the health impacts of air pollution ... 232
4.2: The transition to net zero-carbon, health-supporting economies ... 235
Indicator 4.2.1: employment in low-carbon and high-carbon industries ... 235
Indicator 4.2.2: compatibility of fossil fuel company strategies with the Paris Agreement ... 237

Indicator 4.2.3: stranded coal assets from the energy transition......242
Indicator 4.2.4: country preparedness for the transition to net zero......248
Indicator 4.2.5: production-based and consumption-based attribution of CO_2 and $PM_{2.5}$ emissions......265
4.3: Financial transitions for a healthy future......275
Indicator 4.3.1: clean energy investment......275
Indicator 4.3.2: funds divested from fossil fuels......280
Indicator 4.3.3: net value of fossil fuel subsidies and carbon prices......283
Indicator 4.3.4: fossil fuel and green sector bank lending......292
Section 5: Public and Political Engagement......295
Indicator 5.1: Media Engagement with Health and Climate Change......295
Indicator 5.2: individual engagement with health and climate change......309
5.3: Scientific engagement with health and climate change......334
Indicator 5.3.1: scientific articles on health and climate change 1990–2022......334
Indicator 5.3.2: scientific engagement on the health impacts of climate change......339
5.4: Political engagement with health and climate change......344
Indicator 5.4.1: government engagement......344
Indicator 5.4.2: engagement by international organisations......363
Indicator 5.5: corporate sector engagement with health and climate change......371
References......383

The 2024 Report of the *Lancet* Countdown on Health and Climate Change

This appendix provides methodological details on each of the *Lancet* Countdown's indicators, alongside data sources used and caveats. Wherever suitable, future plans for the indicators and further analysis are also presented.

While methodologies of the indicators might be unchanged from previous Lancet Countdown reports, they are reproduced in full below to aid the interpretation of the findings in the report. Please note that the text is, in many cases, the same as has been published in previous reports. Wherever suitable, references to the original articles describing the methodologies have been added.

Wherever possible and appropriate, indicators are disaggregated into very high, high, medium, and low human development index (HDI) country groups, as defined by the UNDP.[1] The attained level of HDI in the latest year of data available during the writing of this report (2021) is used, acknowledging that the achievement of a HDI level is the product of several years of work towards improving the parameters that define it (Table 1). The HDI captures three core dimensions: a long and healthy life (using life expectancy as a proxy), education (monitored by the mean of years of Schooling in a given country), and standard of living (using per-capita gross national income as a proxy).

Many indicators are also disaggregated by world region (often referred to as "LC Regions" in analyses and figures), as shown in

Table 2. To allow for global coverage, the country groupings often differ from the regions covered by the *Lancet* Countdown's regional centres. Analysis by World Health Organization groups is also provided where relevant (

Table 3).

Unless otherwise specified, the indicators that incorporate retrospective climate data make use of the climate reanalysis datasets, mostly ERA5, but also including ERA5-Land and ORAS5. These datasets incorporate vast amounts of historical observations, including those from satellites, to provide the most complete description of the observed climate as it has evolved during recent decades. Due to their temporal and geographical coverage, these are the most appropriate data for the purposes of the *Lancet* Countdown indicators. Slight discrepancies might exist between reanalysis datasets, and other types of retrospective climatological modelling, which however would only have slight impacts on findings of the indicators here presented. All monetary values in the *Lancet* Countdown are expressed in 2023 US dollars, unless stated otherwise in the main text or cited sources.

Table 1: List of countries included in each HDI group, according to their 2021 HDI level[2,3]

**Very High**	Bahrain, Brunei Darussalam, Cyprus, Georgia, Israel, Japan, Kazakhstan, Kuwait, Malaysia, Oman, Qatar, Republic of Korea, Saudi Arabia, Thailand, Turkey, United Arab Emirates, Andorra, Austria, Belarus, Belgium, Croatia, Czechia, Denmark, Estonia, Finland, France, Germany, Greece, Hungary, Iceland, Ireland, Italy, Latvia, Liechtenstein, Lithuania, Luxembourg, Malta, Montenegro, Netherlands, Norway, Poland, Portugal, Romania, Russian Federation, San Marino, Serbia, Slovakia, Slovenia, Spain, Sweden, Switzerland, United Kingdom, Canada, United States of America, Australia, New Zealand, Bahamas, Mauritius, Singapore, Trinidad and Tobago, Argentina, Chile, Costa Rica, Panama, Uruguay.
**High**	Algeria, Egypt, Gabon, Libya, South Africa, Tunisia, Armenia, Azerbaijan, China, Indonesia, Islamic Republic of Iran, Jordan, Lebanon, Mongolia, Occupied Palestinian territory, Sri Lanka, Turkmenistan, Uzbekistan, Vietnam, Albania, Bosnia and Herzegovina, Bulgaria, North Macedonia, Republic of Moldova, Ukraine, Antigua and Barbuda, Barbados, Cuba, Dominica, Dominican Republic, Fiji, Grenada, Guyana, Jamaica, Maldives, Palau, Saint Kitts and Nevis, Saint Lucia, Saint Vincent and the Grenadines, Samoa, Seychelles, Suriname, Tonga, Brazil, Colombia, Ecuador, Mexico, Paraguay, Peru
**Medium**	Angola, Botswana, Cameroon, Congo, Cote d'Ivoire, Equatorial Guinea, Eswatini, Ghana, Kenya, Mauritania, Morocco, Namibia, Zambia, Zimbabwe, Bangladesh, Bhutan, Cambodia, India, Iraq, Kyrgyzstan, Lao People's Democratic Republic, Myanmar, Nepal, Philippines, Syrian Arab Republic, Tajikistan, Belize, Cabo Verde, Comoros, Federated States of Micronesia, Kiribati, Marshall Islands, Papua New Guinea, Sao Tome and Principe, Solomon Islands, Timor-Leste, Tuvalu, Vanuatu, Bolivarian Republic of Venezuela, Bolivia, El Salvador, Guatemala, Honduras, Nicaragua
**Low**	Benin, Burkina Faso, Burundi, Central African Republic, Chad, Democratic Republic of the Congo, Djibouti, Eritrea, Ethiopia, Gambia, Guinea, Lesotho, Liberia, Madagascar, Malawi, Mali, Mozambique, Niger, Nigeria, Rwanda, Senegal, Sierra Leone, South Sudan, Sudan, Togo, Uganda, United Republic of Tanzania, Afghanistan, Pakistan, Yemen, Guinea Bissau, Haiti

Table 2: List of countries included in each global region for regional analyses

**Region**	Countries Included
**Africa**	Algeria, Angola, Benin, Botswana, Burkina Faso, Burundi, Cameroon, Central African Republic, Chad, Congo, Cote d'Ivoire, Democratic Republic of the Congo, Djibouti, Egypt, Equatorial Guinea, Eritrea, Eswatini, Ethiopia, Gabon, Gambia, Ghana, Guinea, Kenya, Lesotho, Liberia, Libya, Madagascar, Malawi, Mali, Mauritania, Morocco, Mozambique, Namibia, Niger, Nigeria, Rwanda, Senegal, Sierra Leone, Somalia, South Africa, South Sudan, Sudan, Togo, Tunisia, Uganda, United Republic of Tanzania, Zambia, Zimbabwe
**Asia**	Afghanistan, Armenia, Azerbaijan, Bahrain, Bangladesh, Bhutan, Brunei Darussalam, Cambodia, China, Cyprus, Democratic People's Republic of Korea, Georgia, India, Indonesia, Iraq, Islamic Republic of Iran, Israel, Japan, Jordan, Kazakhstan, Kuwait, Kyrgyzstan, Lao People's Democratic Republic, Lebanon, Malaysia, Mongolia, Myanmar, Nepal, Occupied Palestinian territory, Oman, Pakistan, Philippines, Qatar, Republic of Korea, Saudi Arabia, Sri Lanka, Syrian Arab Republic, Tajikistan, Thailand, Turkey, Turkmenistan, United Arab Emirates, Uzbekistan, Vietnam, Yemen
**Europe**	Albania, Andorra, Austria, Belarus, Belgium, Bosnia and Herzegovina, Bulgaria, Croatia, Czechia, Denmark, Estonia, Finland, France, Germany, Greece, Hungary, Iceland, Ireland, Italy, Latvia, Liechtenstein, Lithuania, Luxembourg, Malta, Monaco, Montenegro, Netherlands, North Macedonia, Norway, Poland, Portugal, Republic of Moldova, Romania, Russian Federation, San Marino, Serbia, Slovakia, Slovenia, Spain, Sweden, Switzerland, Ukraine, United Kingdom
**Northern America**	Canada and United States of America
**Oceania**	Australia and New Zealand
**SIDS**	Anguilla, Antigua and Barbuda, Aruba, Bahamas, Barbados, Belize, Bermuda, British Virgin Islands, Cabo Verde, Cayman Islands, Comoros, Cook Islands, Cuba, Curaçao, Dominica, Dominican Republic, Federated States of Micronesia, Fiji, Grenada, Guadeloupe, Guinea Bissau, Guyana, Haiti, Jamaica, Kiribati, Maldives, Marshall Islands, Martinique, Mauritius, Montserrat, Nauru, Niue, Palau, Papua New Guinea, Puerto Rico, Saint Kitts and Nevis, Saint Lucia, Saint Vincent and the Grenadines, Samoa, Sao Tome and Principe, Seychelles, Singapore, Sint Maarten, Solomon Islands, Suriname, Timor-Leste, Tonga, Trinidad and Tobago, Turks and Caicos, Tuvalu, US Virgin Islands, Vanuatu
**South and Central America**	Argentina, Bolivarian Republic of Venezuela, Bolivia, Brazil, Chile, Colombia, Costa Rica, Ecuador, El Salvador, French Guiana, Guatemala, Honduras, Mexico, Nicaragua, Panama, Paraguay, Peru, Uruguay

Table 3: List of countries included in each WHO regions

**African Region (AFR)**	Algeria, Angola, Benin, Botswana, Burkina Faso, Burundi, Cameroon, Cape Verde, Central African Republic, Chad, Comoros, Ivory Coast, Democratic Republic of the Congo, Equatorial Guinea, Eritrea, Ethiopia, Gabon, Gambia, Ghana, Guinea, Guinea-Bissau, Kenya, Lesotho, Liberia, Madagascar, Malawi, Mali, Mauritania, Mauritius, Mozambique, Namibia, Niger, Nigeria, Republic of the Congo, Rwanda, São Tomé and Príncipe, Senegal, Seychelles, Sierra Leone, South Africa, South Sudan, Eswatini, Togo, Uganda, Tanzania, Zambia, Zimbabwe
**Region of the Americas (AMR)**	Antigua and Barbuda, Argentina, Bahamas, Barbados, Belize, Bolivia, Brazil, Canada, Chile, Colombia, Costa Rica, Cuba, Dominica, Dominican Republic, Ecuador, El Salvador, Grenada, Guatemala, Guyana, Haiti, Honduras, Jamaica, Mexico, Nicaragua, Panama, Paraguay, Peru, Saint Kitts and Nevis, Saint Lucia, Saint Vincent and the Grenadines, Suriname, Trinidad and Tobago, United States, Uruguay, Venezuela
**South-East Asian Region (SEAR)**	Afghanistan, Bangladesh, Bhutan, North Korea, India, Indonesia, Maldives, Myanmar, Nepal, Sri Lanka, Thailand, Timor-Leste
**European Region (EUR)**	Albania, Andorra, Armenia, Austria, Azerbaijan, Belarus, Belgium, Bosnia and Herzegovina, Bulgaria, Croatia, Cyprus, Czech Republic, Denmark, Estonia, Finland, France, Georgia, Germany, Greece, Hungary, Iceland, Ireland, Israel, Italy, Kazakhstan, Kyrgyzstan, Latvia, Lithuania, Luxembourg, Malta, Moldova, Monaco, Montenegro, Netherlands, North Macedonia, Norway, Poland, Portugal, Romania, Russia, San Marino, Serbia, Slovakia, Slovenia, Spain, Sweden, Switzerland, Tajikistan, Turkey, Turkmenistan, Ukraine, United Kingdom, Uzbekistan.
**Eastern Mediterranean Region (EMR)**	Lebanon, Libya, Morocco, Oman, Pakistan, Qatar, Saudi Arabia, Somalia, Sudan, Syria, Tunisia, United Arab Emirates, Yemen
**Western Pacific Region (WPR)**	Australia, Brunei, Cambodia, China, Cook Islands, Fiji, Japan, Kiribati, Laos, Malaysia, Marshall Islands, Micronesia, Mongolia, Nauru, New Zealand, Niue, Palau, Papua New Guinea, Philippines, Samoa, Singapore, Solomon Islands, South Korea, Tonga, Tuvalu, Vanuatu, Vietnam

Section 1: Health Hazards, Exposures, and Impact

Section Lead: Prof Elizabeth J. Z. Robinson
Research Fellow: Annalyse Moskeland

1.1: Health and heat

Indicator 1.1.1: exposure of vulnerable populations to heatwaves

Indicator Authors

Dr Jonathan D. Chambers and Zélie Stalhandske

Methods

A frozen version of the python code used to produce these results has been made available.[4]
The input data for this indicator have been improved and extended for the 2024 report.
The indicator defines a heatwave as a period of two or more days where both the minimum and maximum temperatures are above the 95th percentile of the local climatology (defined on the 1986–2005 baseline). This reflects the definition from published scientific literature on the topic.[5] It also aims to capture the health effects of both direct heat extremes (i.e., caused by high maximum temperatures) and the problems associated with lack of recovery (i.e., caused by high minimum temperatures) over persisting hot periods.[6] The gridded 95th percentile of daily minimum and maximum temperatures, taken from the European Centre for Medium-Range Weather Forecasts (ECMWF) ERA5 dataset, were calculated on a 0.25° x 0.25° global grid for 1986–2005. For each year from 1980 to 2023, the number of heatwave events and total days of heatwaves per year was calculated according to the definition above.

Vulnerable populations are defined as those above the age of 65 and infants between 0 and one years old. Previous research has identified these groups as being particularly vulnerable to heatwave impacts on health.[7]

Data inspection has shown that increasing heatwave length can result in fewer discrete heatwave events as they merge into single long events – this is therefore better captured by the person-days metric. To reflect this the exposure of vulnerable populations to heatwaves is computed as person-days, i.e., by multiplying the number of heatwave days by vulnerable population count. In this way, the indicator captures both the changes in duration and in frequency of heatwaves, as well as the changing demographics that might mean more vulnerable people are at risk. [8]

The WorldPop project provides detailed global gridded data on age and sex structures at a 1km x 1km resolution for the period 2000–2020, based on the "top-down unconstrained approach" (see section on Caveats below for more details on the methodology).[9] This data is utilised to identify two categories of vulnerable populations. For infants under one year of age, WorldPop offers separate datasets for females and males, which are aggregated by summing the values for each year at every grid point. For the elderly population over 65, datasets for age groups 65–70, 70–75, 75–80, and above 80, for both females and males, are merged by summing the values at each grid point.

These aggregated datasets are then up-scaled by decreasing their resolution from 1km x 1km to match the ERA5 grid's 0.25° x 0.25° resolution. This is achieved by identifying the nearest neighbour for each point on the ERA5 grid and aggregating all corresponding values for each ERA5 grid point. For

historical data prior to 2000, the data generated for the *Lancet* Countdown 2023 is used. This dataset was generated based on the ISIMIP Histsoc dataset, by resampling it to a 0.25° x 0.25° resolution through a 2D linear interpolation method that incorporates population densities and NASA GPWv4 land area data.[10] For projections beyond 2020 of the WorldPop data, linear interpolation at each grid point is used to estimate population for more recent years.
Given the discontinuous nature of the population data, discrepancies may arise between the pre- and post- 2000 figures. Consequently, the analysis is framed in terms of exposure to change rather than a direct measurement of change in exposure. This approach mitigates the issue of calculating population changes across periods of data discontinuity.

Data

- Climate data from the European Centre for Medium-Range Weather Forecasts (ECMWF) ERA5 reanalysis
- Hybrid gridded demographic data for the world from the Lancet Countdown 2023, use for years 1980–2000, 0.25˚ resolution[10]
- WorldPop Age and Sex Structure Unconstrained Global Mosaic data for 2000–2020[9]

Caveats

To accurately assess demographic trends over time, data were integrated from various sources to capture both spatial and temporal dimensions of population dynamics. However, this integrated dataset has undergone limited validation. Challenges arise in regions with sparse demographic data or where shifts in political boundaries lead to inconsistencies in the spatial distribution of demographic information. For instance, the division of Sudan is reflected in our data as gaps or missing information for infant populations. This example highlights the complexities involved in maintaining continuous demographic datasets amidst changing geopolitical landscapes.

For the WorldPop data, the yearly estimates are available for the "Top-down unconstrained method". This population mapping approach estimates population distribution without limiting the allocation of people to areas identified as having residential structures, as opposed to the "constrained" approach. Instead of relying on detailed satellite imagery to pinpoint where people live, this method broadly distributes population data across all land areas, based on available demographic and geographic information. This approach assumes a continuous spread of population (non-zero allocation of population to all land grid cells) without the precise delineation of inhabited versus uninhabited land, effectively covering entire regions or countries without the constraint of identifying individual buildings or settlements. This may lead to an overestimation of population in low density areas, and to an underestimation in high density areas.

Future form of the indicator

Future versions of the indicator aim to use ECMWF ERA5-Land data at 0.1˚ x 0.1° spatial resolution. The increased data volume at the global level, plus the need to adapt corresponding population data, requires upgrades to the data processing.

Additional analysis

Figure 1 summarises the change in number of heatwave days in 2023 relative to the baseline. Intense events in the USA, Southern Europe, in the Middle east, Central Africa, as well as Central and Eastern Europe are evident. Figure 2 highlights that exposures are larger in the over-65 age group, and new records have been reached in the average number of heatwave days experienced this year by both age groups. In Figure 5 in the ‘low’ HDI class countries the exposure is lower than the other classes, especially for the over 65 age group. This is likely related to lower life expectancy in countries in the ‘low’ HDI class. In the WHO region disaggregation as shown in Figure 6, the European region appears to be the most affected, due to the higher occurrence of heatwave as defined in this report.

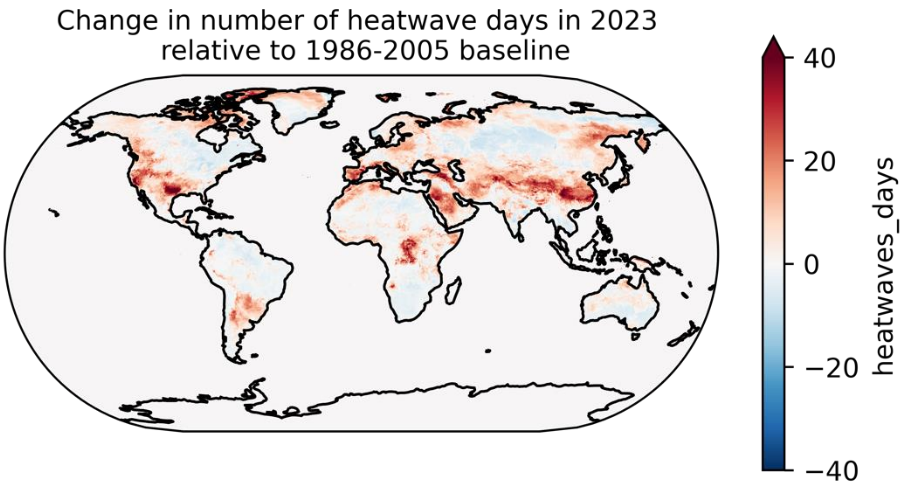

Figure 1: Map of the change in number of heatwave days over land in 2023 relative to the 1986–2005 baseline.

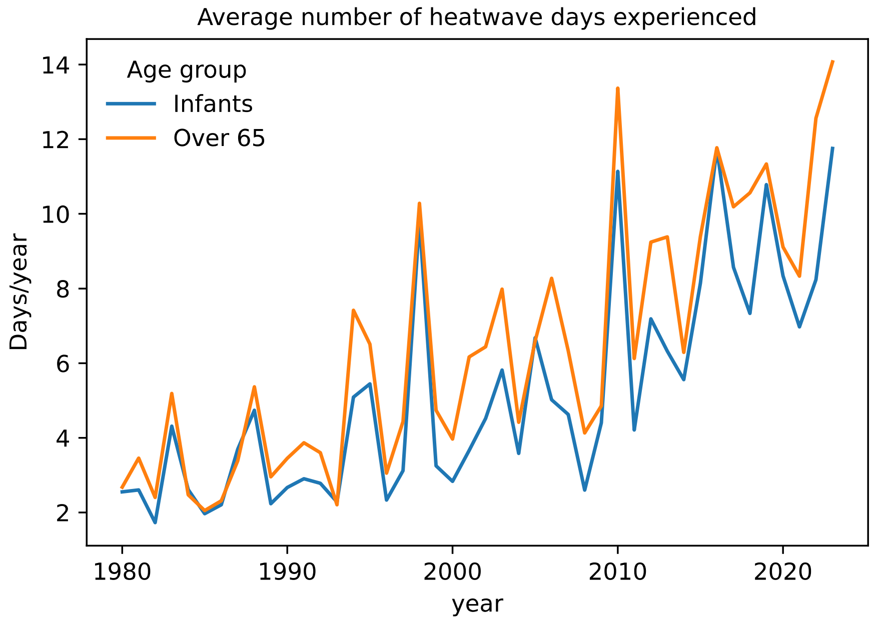

Figure 2: Average days of heatwave experienced by people over 65 and infants under one year old. ISIMIP population data is used for 1980–2000, WorldPop population data is used for 2000–2023.

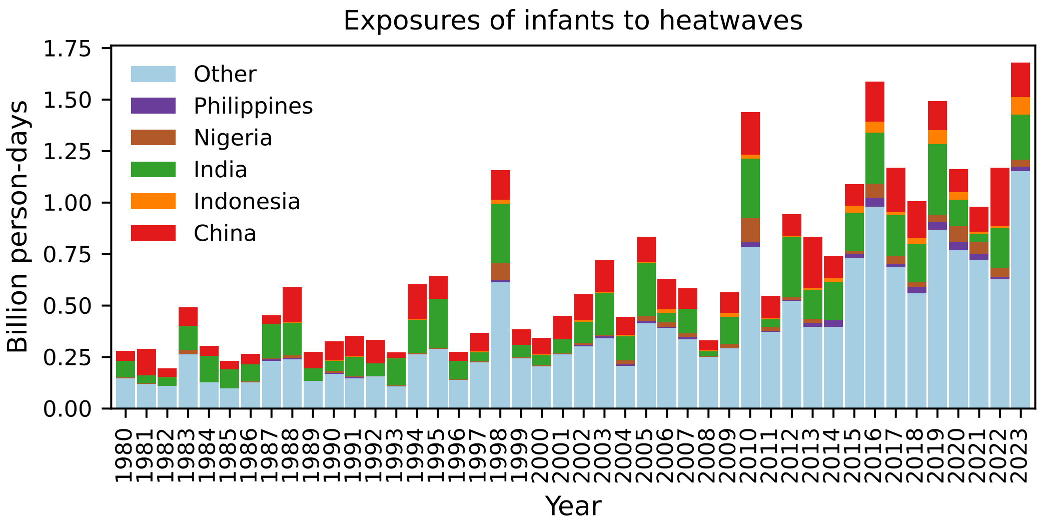

Figure 3: Total person-days of heatwave experienced by infants under one year old, by most affected country and by year.

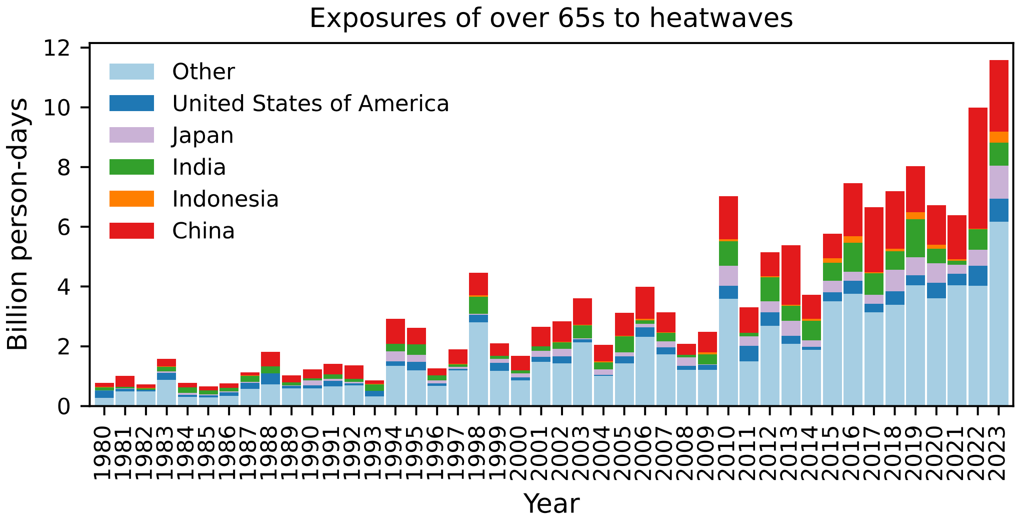

Figure 4: Total person-days of heatwave experienced by people over 65-year-old, by most affected country and by year.

When disaggregating by country as shown on Figure 3 and Figure 4, a high number of people tend to be affected in both categories in China and India, due to the high population. In 2023, many people over 65 were additionally affected in Japan, in the United States of America and in Indonesia, while infants were particularly affected in Indonesia and Nigeria.

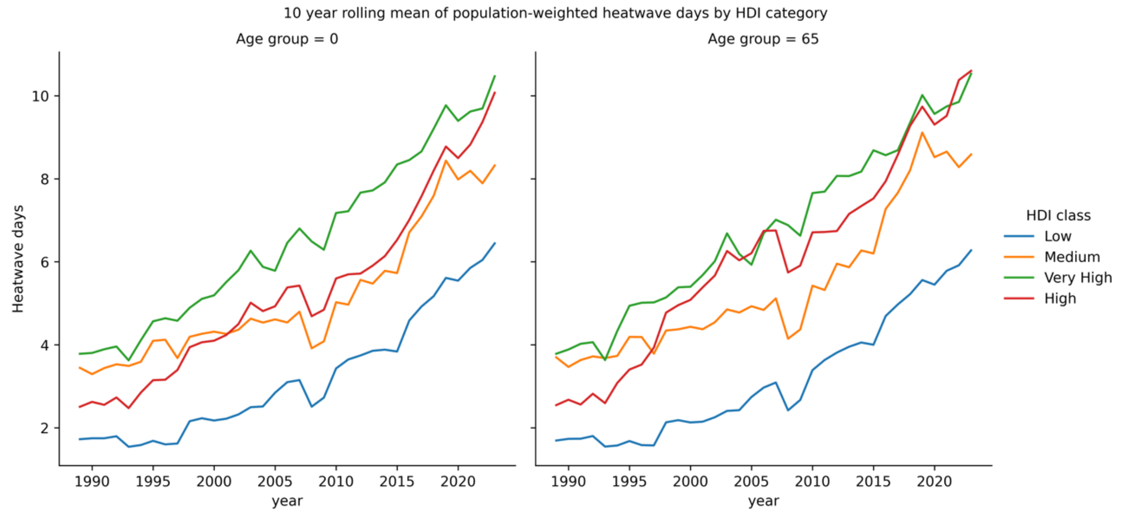

Figure 5: 10 year rolling mean of exposure weighted heatwave days aggregated by HDI level.

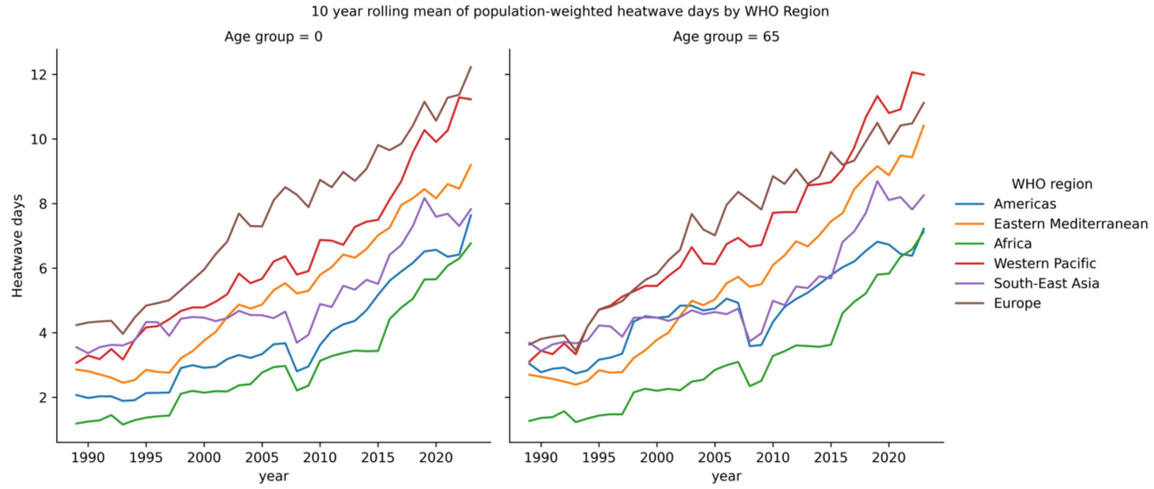

Figure 6: 10 year rolling mean of exposure weighted heatwave days aggregated by WHO region.

Differences in population data compared to the 2023 report

The population data in 2024 differs from the 2023 report due to the change of data used, as shown in Figure 5 and Figure 6. While the GPW data tends to agree with the UN data, the WorldPop's estimates of demographic evolution can differ primarily due to differences in methods used. The GPW data makes use of census data that is disaggregated, while the WorldPop methodology integrates diverse data, including census information, surveys, and remote sensing, which can vary in quality and timelines across regions. This approach, especially in data-scarce or politically fluctuating areas, necessitates heavier reliance on modelling. These differences in methodology and data utilisation may lead to discrepancies between WorldPop and UN demographic estimates. The change in population data do not however lead to large differences when looking at the difference in the number of heatwave days * persons, even at country level (see Figure 9).

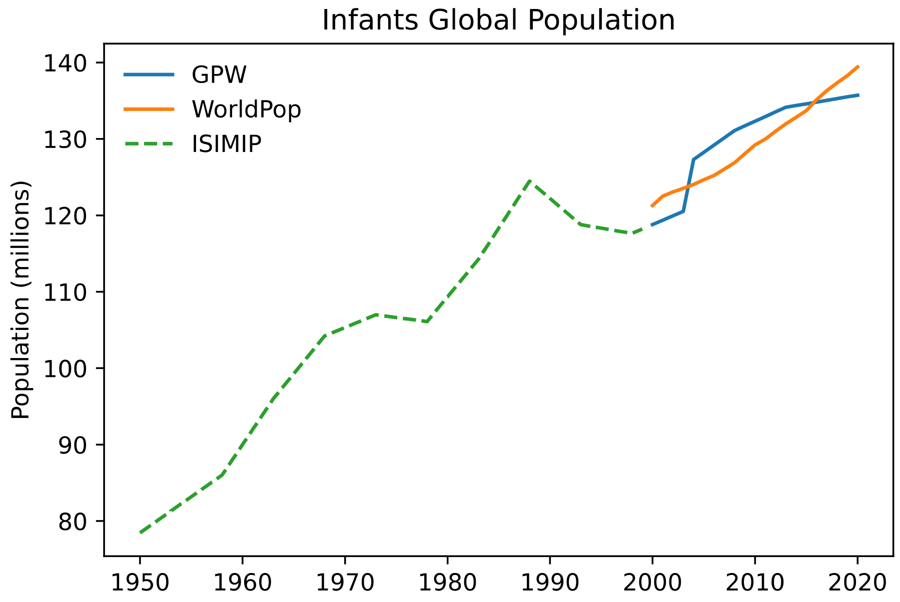

Figure 7: Global infant population trend using WorldPop-hybrid population data compared to using the GPW-hybrid for 2000–2020. The ISIMIP data used for before 2000 remains the same.

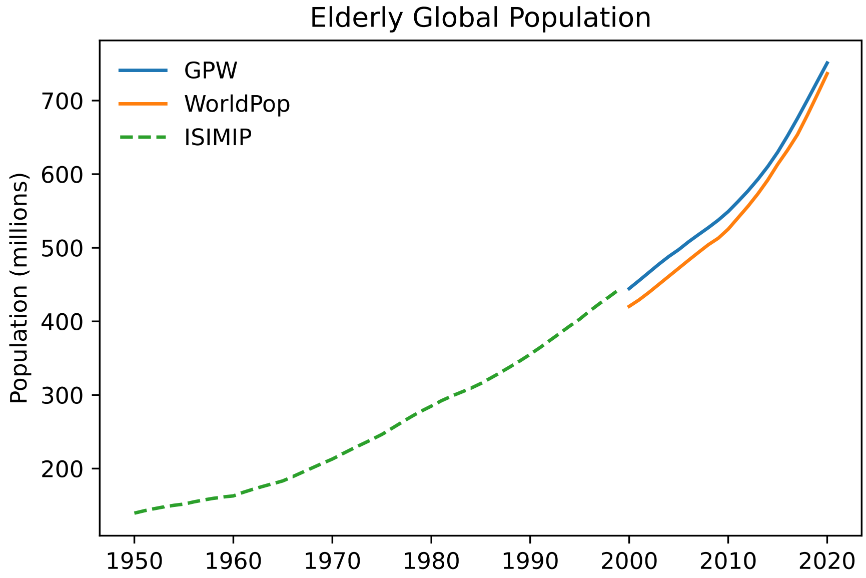

Figure 8: Global over 65 population trend using WorldPop-hybrid population data compared to using the GPW-hybrid for 2000–2020. The ISIMIP data used for before 2000 remains the same.

Effect of climate change compared to effect of population growth

While climate change influences the occurrence of heatwave days, the change in population is also affecting the number of heatwave day * persons. In this section, the years 1986–2005 to the years 2004–2023 are compared to assess to how many heatwaves vulnerable population would have been exposed to on average if there was no climate change, but only population growth.

For each geographic coordinate, the average annual heatwave days for both the elderly and infant populations are computed, for period 2004–2023. This calculation is replicated for the second period, while holding the average annual heatwave days constant to 1986–2005 levels, thus isolating the effects of climate change.

By comparing the resultant increase in heatwave days per person across each location, the aim is to identify how many heatwave days vulnerable population would have been exposed to without climate change, but by still including demographic changes.

Keeping the average incidence of heatwave days constant at baseline levels, vulnerable populations would have only experienced 4.7 heatwave days per person on average per year in 2004–2023 – 45% less than observed. Infants saw each, on average, 3.2 days of heatwave days more on average in 2004–2023 than in 1986–2005. Similarly, adults over-65, a group which has grown fast, saw an extra 3.9 days of heatwaves days each on average per year in 2004–2023. For both groups, a slight decrease in the average heatwave day per person would have been observed under a constant heatwave incidence at 1986–2005 level. This due to changes in where the highest number of vulnerable are located.

Comparison with results from the 2023 report

With updates to population data, minor discrepancies are noticeable in comparison to findings from prior years. For instance, as shown for infants on Figure 9, the analysis at the country level indicates that, although the ranking of the most impacted countries remains unchanged, the specific number of heatwave days experienced annually may exhibit slight variations across some nations. However, these variations do not alter the primary conclusions drawn from these data.

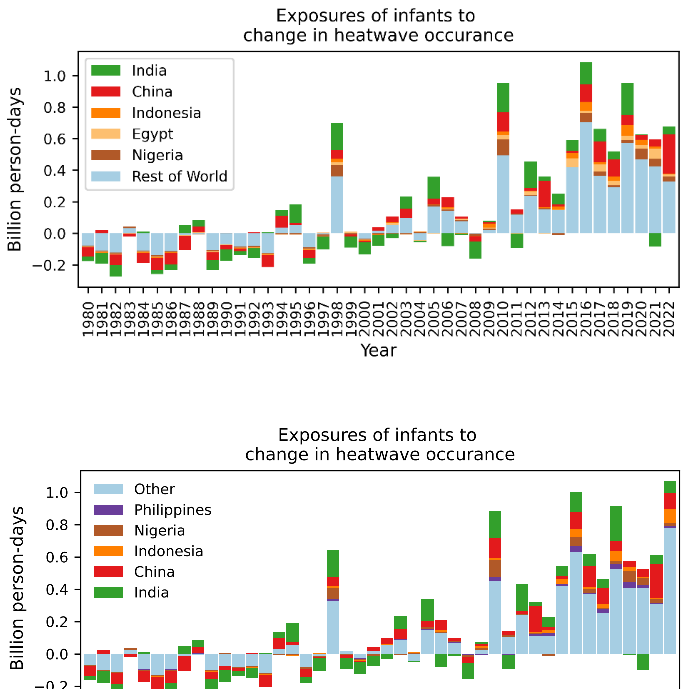

Figure 9: Comparison of total heatwave days experienced by most affected country in the 2023 report (a) compared to the 2024 report (b). The heatwave data remained unchanged until 2022, while the population data source was changed to WorldPop.

Indicator 1.1.2: heat and physical activity

Indicator Authors

Dr Troy J Cross, Dr Samuel H Gunther, Prof Ollie Jay, Prof Jason KW Lee

Methods

The methodology for this indicator has been updated from the 2023 report of the *Lancet* Countdown.[11] Our estimation of moderate–, high–, and extreme–risk hours incorporates the international boundaries for each country based on the Detailed Boundaries ADM0 shapefiles provided by the WHO, as opposed to those provided by the National Identifier Grid packaged with the UN–adjusted Gridded Population of the World.

Hourly 2–metre temperature and 2–metre dew point temperature data were retrieved from European Centre for Medium–Range Weather Forecasts ERA5 climate reanalysis datasets. This spatiotemporal data provided hourly slices of a rectangular 0.25° x 0.25° grid of the world for each climate variable. While ERA5 data are available from 1940, data from the years 1990 to 2023 were considered for the purposes of this analysis. Relative humidity was derived from 2–metre dew point temperature by applying Antoine's equation to estimate water vapour pressure and expressing it relative to saturation vapour pressure at the given hourly 2–metre temperature. A solar calendar was created using the *suncalc* R package to determine if a given hour within a given ERA5 grid cell was between sunrise and sunset times (i.e., daylight).

Heat stress risk was estimated from these climate variables in accordance with the 2021 Sports Medicine Australia (SMA) Extreme Heat Policy, which stratifies estimated heat stress risk in to four categories – low, moderate, high, and extreme – based on ambient temperature and relative humidity.[12] Sports and activities are further classified into five risk classification groups, ranging from leisurely walking to mountain biking, based on intensity of the activity and clothing worn.[12] The present analysis includes assessments of heat stress risk for Risk Classification 1, which is termed "low intensity" for this analysis (e.g., leisurely walking) and Risk Classification 3, which is termed "moderate intensity" for this analysis (e.g., jogging, cycling).

The number of daylight hours in each ERA5 grid cell with a recorded temperature and humidity combination that exceeded at least the threshold for "moderate", "high", and "extreme" heat stress risk for Risk Classifications 1 and 3 were tabulated for each year from 1990 to 2023. The total number of sunlight hours per year exceeding each threshold in each grid cell was then weighted by population.

Population weighting was performed by multiplying the number of daylight hours per year that at least exceeded each threshold by the population, as provided by the GPWv4.11 (UN) WPP Adjusted population count dataset,[13] in the respective grid cell. The population–weighted hours at least exceeding each threshold in a single year were added up for all grid cells in a given country, and these values were divided by the total population of the country in that year to calculate the number of hours per person that at least exceeded the "moderate", "high", and "extreme" heat stress risk thresholds for each Risk Classification group.

The temperature–dependent humidity thresholds were defined using the following functions:

<u>For Risk Classification 1 (light intensity):</u>

Moderate heat stress risk:

$f(x) = 2762.19706058 – 381.32569858*x + 22.54042858*x^2 – 0.68633536*x^3 + 0.01057218*x^4 –0.00006556*x^5$

High heat stress risk:

$f(x) = 580.94555243 – 34.94707422*x + 0.86136002*x^2 – 0.01202855*x^3 + 0.00012645*x^4 – 0.00000100*x^5$

Extreme heat stress risk:

$f(x) = 526.92566942 – 26.92074280*x + 0.49204120*x^2 – 0.00291451*x^3 – 0.00000974*x^4 – 0.00000001*x^5$

<u>For Risk Classification 3 (moderate intensity):</u>

Moderate heat stress risk:

$f(x) = 1329.05763259 – 133.97879449*x + 5.77944375*x^2 – 0.12867880*x^3 + 0.00142458*x^4 – 0.00000618*x^5$

High heat stress risk:

$f(x) = 1242.42231049 – 116.61462820*x + 4.71301279*x^2 – 0.09819257*x^3 + 0.00101106*x^4 – 0.00000406*x^5$

Extreme heat stress risk:

$f(x) = 1507.77713966 – 144.87110578*x + 5.95209387*x^2 – 0.12442233*x^3 + 0.00127595*x^4 – 0.00000508*x^5$

where x is 2–metre temperature in a given hour and $f(x)$ is 2–metre relative humidity derived from dew point temperature in a given hour.

These threshold functions are defined by SMA as the boundary above which the risk of exertional heat illness changes, and the following preventive actions should be taken:[12]

- moderate heat stress risk: additional rest breaks should be undertaken;
- high heat stress risk: active cooling strategies (e.g., water dousing) should be implemented;
- extreme heat stress risk: activities should be suspended due to excessive heat stress risk

The functions in the 2021 SMA Extreme Heat Policy extend to a minimum ambient temperature of 26°C. Accordingly, any values recorded below this temperature, irrespective of ambient humidity, were determined as presenting a “low” heat stress risk.

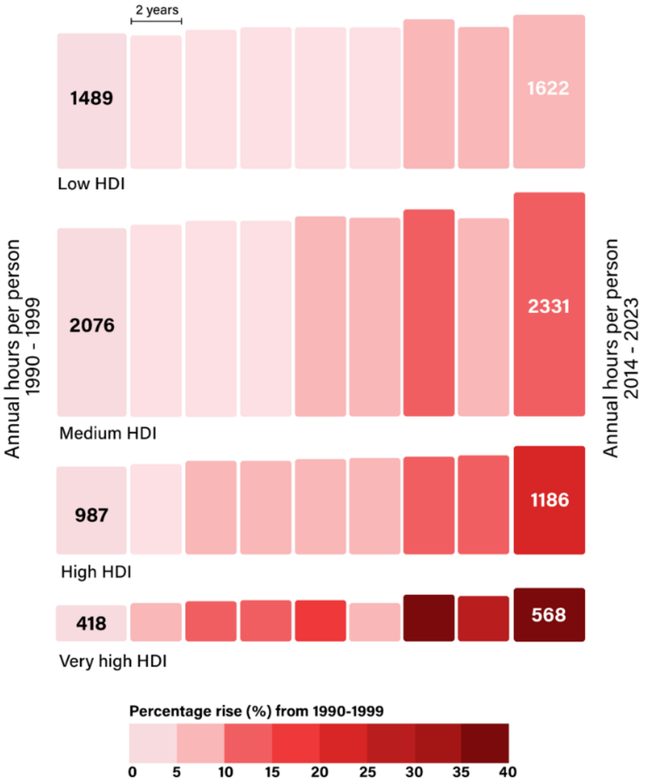

Figure 10: Change in annual hours per person from baseline (1990–1999) to 2014–2023 when light physical activity entailed at least a moderate heat stress risk, grouped by HDI. Blocks represent two–year intervals. The height of the block represents the absolute number of annual hours per person.

Data

- World Health Organization Detailed Boundary ADM0 Shapefiles
- Population data from the NASA Socioeconomic Data and Applications Center Gridded Population of the World (GPWv4) – UN WPP – Adjusted Population Count, v4.11[3]
- Climate data from the European Centre for Medium–Range Weather Forecasts ERA5 reanalysis[144]

Caveats

Heat stress risk for each exercise intensity classification may differ among people due to various risk factors. For example, older adults or people taking certain medications may experience reduction in sweating which compromises their ability to keep cool and could elevate their exertional heat stress risk at a given combination of temperature and humidity.[15] Other groups that may have greater heat stress risk include young children, people wearing heavy clothing or living with disabilities or chronic diseases. A more detailed interpretation model of the heat effects of exercise would incorporate individual factors such as age, health status, and clothing.[16] SMA policy assumes summertime strength of solar radiation. Heat stress risk in early spring and later autumn months may therefore be overestimated. It is also assumed that population averages for an entire year were applicable to each hourly grid cell, which may not be true, but still provides a rough estimate of population assuming an even rate of influx and outflux from each cell at the country level.

Future form of the indicator

Results will be updated using each new year of available climate data and, as sports authorities issue their updated threshold guidelines, they will be expressed according to the latest policy developments. Subsequent versions of the indicator will integrate seasonal changes in solar radiation to overcome the current assumption of summertime levels of solar radiation intensity year–round. Future versions will also explore the derivation of different heat stress risk thresholds for different subpopulation groups, e.g., acclimatised, unacclimatised, elderly, young children.

Additional analyses

<u>Risk Classification 3 – global analysis:</u>
In 2014–2023, compared with 1990–1999, the hours of at least moderate heat stress risk during moderate outdoor activities (e.g., jogging) increased globally by an average of 277 hours per person (21.1%). The largest absolute increase was observed in medium HDI countries (244 hours per person; 10.7%), whereas the greatest percent increase was observed in very high HDI countries (169 hours per person; 34.8%) (*Figure 10: Change in annual hours per person from baseline (1990–1999) to 2014–2023 when light physical activity entailed at least a moderate heat stress risk, grouped by HDI. Blocks represent two–year intervals. The height of the block represents the absolute number of annual hours per person.*).

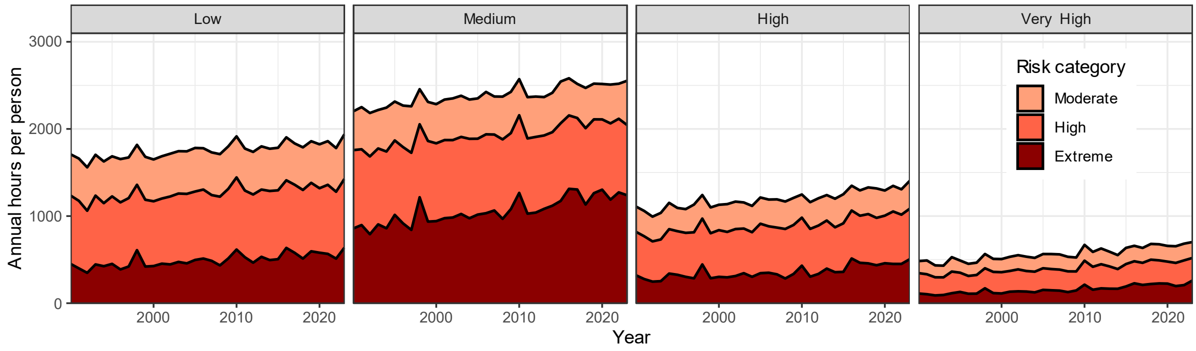

Figure 11: Average annual hours per person that moderate physical activity (SMA Risk Classification 3) entailed at least a moderate, high, or extreme heat stress risk by HDI (Low, Medium, High, Very High) grouping, 1990–2023).

Analyses were also carried out at the level of *Lancet* Countdown country group and WHO regional group.

<u>*Lancet* Countdown country grouping</u>
For Risk Classification 1, when separated by *Lancet* Countdown country group, relative to a baseline of 1990 to 1999 (inclusive), the number of hours that exceeded the threshold for moderate heat stress risk increased in the period between 2014 and 2023 (inclusive) as follows ()

- *Europe*: by 34 hours per person per year (81.8% increase)
- *Oceania*: by 26 hours per person per year (17.0% increase)
- *Northern America*: by 115 hours per person per year (27.0% increase)
- *Latin America*: by 201 hours per person per year (20.5% increase)
- *SIDS*: by 438 hours per person per year (20.9% increase)
- *Africa*: by 247 hours per person per year (21.2% increase)

- *Asia*: by 272 hours per person per year (18.1% increase)

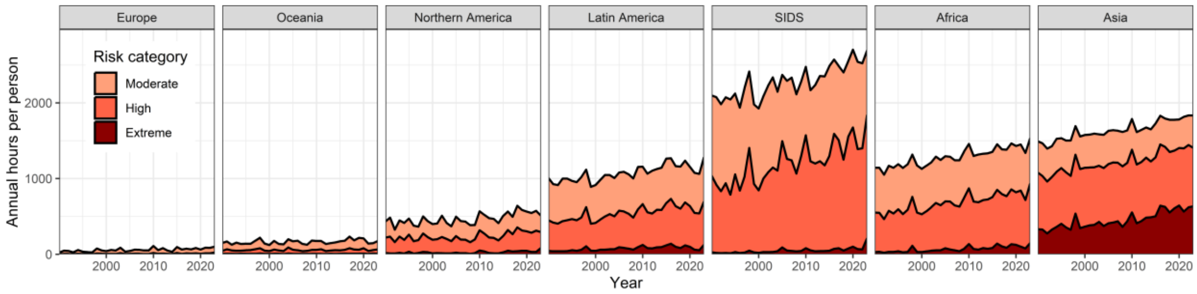

Figure 12: *Average annual hours per person that light physical activity (SMA Risk Classification 1) entailed at least a moderate, high, or extreme heat stress risk by Lancet Countdown country (Europe, Oceania, Northern America, Latin America, SIDS, Africa, Asia) grouping, 1990–2023.*

For Risk Classification 3, when separated by *Lancet* Countdown country group, relative to a baseline of 1990 to 1999 (inclusive), the number of hours exceeding the threshold for moderate heat stress risk increased in the period between 2014 and 2023 (inclusive) as follows (figure 13):

- *Europe*: by 54 hours per person per year (65.5% increase)
- *Oceania*: by 42 hours per person per year (19.3% increase)
- *Northern America*: by 135 hours per person per year (26.4% increase)
- *Latin America*: by 207 hours per person per year (17.9% increase)
- *SIDS*: by 410 hours per person per year (17.6% increase)
- *Africa*: by 268 hours per person per year (19.6% increase)
- *Asia*: by 276 hours per person per year (16.8% increase)

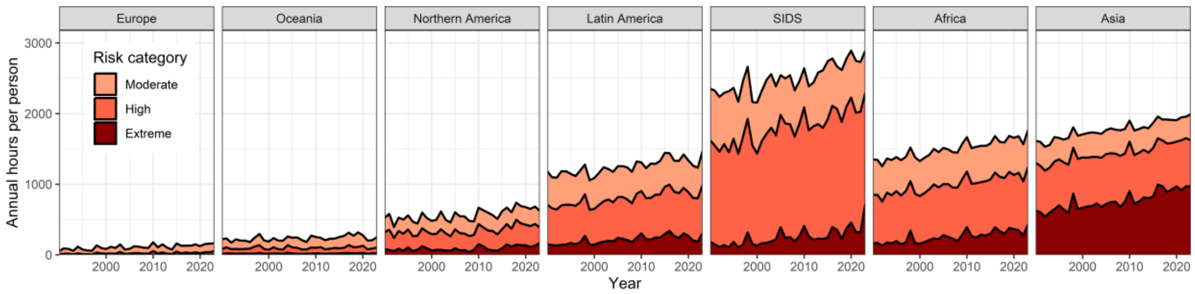

Figure 14: Average annual hours per person that moderate physical activity (SMA Risk Classification 3) entailed at least a moderate, high, or extreme heat stress risk by LC country (Europe, Oceania, Northern America, Latin America, SIDS, Africa, Asia) grouping, 1990–2023.

WHO regional grouping:

For Risk Classification 1, when separated by WHO regional group, relative to a baseline of 1990 to 1999 (inclusive), the number of hours that exceeded the threshold for moderate heat stress risk increased in the period between 2014 and 2023 (inclusive) as follows (figure 15):

- *Africa*: by 232 hours per person per year (19.0% increase)
- *Americas*: by 190 hours per person per year (23.2% increase)
- *Eastern Mediterranean*: by 220 hours per person per year (20.4% increase)
- *Europe*: by 51 hours per person per year (80.4% increase)
- *South–East Asia*: by 277 hours per person per year (12.1% increase)

- *Western Pacific*: by 214 hours per person per year (21.8% increase)

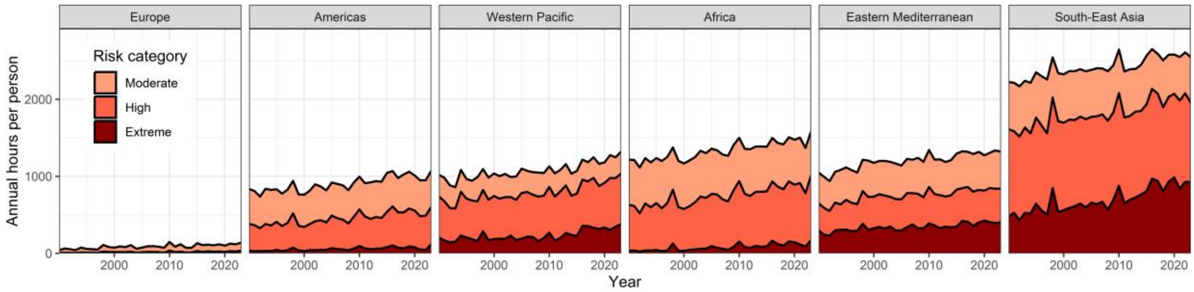

Figure 16: Average annual hours per person that light physical activity (SMA Risk Classification 1) entailed at least a moderate, high, or extreme heat stress risk by WHO country (Africa, Americas, Eastern Mediterranean, Europe, SE Asia, Western Pacific) grouping, 1990–2023.

For Risk Classification 3, when separated by WHO regional group, relative to a baseline of 1990 to 1999 (inclusive), the number of hours that exceeded the threshold for moderate heat stress risk increased in the period between 2014 and 2023 (inclusive) as follows (figure 17):

- *Africa*: by 261 hours per person per year (18.7% increase)
- *Americas*: by 201 hours per person per year (20.8% increase)
- *Eastern Mediterranean*: by 229 hours per person per year (17.7% increase)
- *Europe*: by 75 hours per person per year (64.5% increase)
- *South–East Asia*: by 260 hours per person per year (10.5% increase)
- *Western Pacific*: by 221 hours per person per year (21.0% increase)

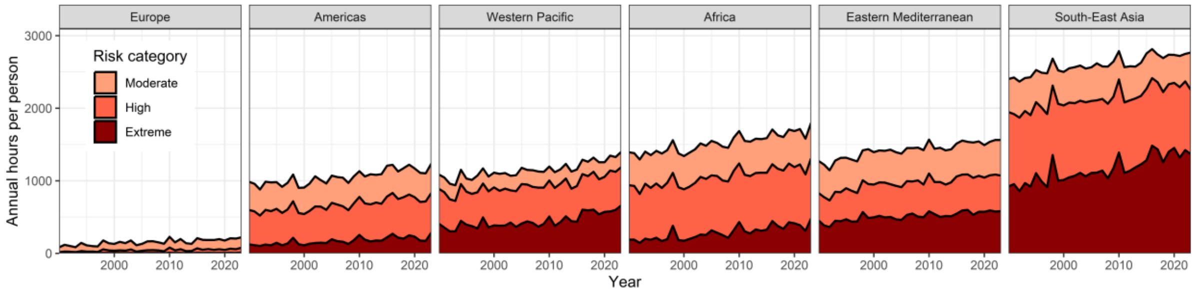

Figure 18: Average annual hours per person that moderate physical activity (SMA Risk Classification 1) entailed at least a moderate, high, or extreme heat stress risk by WHO country (Africa, Americas, Eastern Mediterranean, Europe, SE Asia, Western Pacific) grouping, 1990–2023.

Indicator 1.1.3: change in labour capacity

1.1.3.1 Loss of potential work hours

Indicator authors

Chris Freyberg, Dr Bruno Lemke, Matthias Otto

Methods

This indicator is based geographically on climate and population data for 68,940 grid cells 0.5 x 0.5 degrees with boundaries exactly on the degree and half degree co-ordinates. Its focus is on trends since the end of the 20th century and on a method that can estimate labour capacity loss — the loss of opportunity to produce or earn because of heat exposure — at country level. The climate data chosen for the analysis was the European Centre for Medium-Range Weather Forecasts (ECMWF) ERA5 hourly reanalysis (single levels), and the analysis method is described in detail in the paper by Kjellstrom et al., 2018.[17]

Analysis starts from hourly ambient (t2m) and dew point temperatures (d2m), together with short wave solar radiation downward (ssrd). From these hourly values of the heat stress index Wet Bulb Globe Temperature (WBGT) are derived, and from those hourly work loss fractions (WLF) for human activity at different metabolic rates and situations. The analysis considers - three metabolic rates: 200W (light work, sitting or moving around slowly), 300W (medium intensity work), and 400W (heavy labour) and two work situations: in shade and in full sun.

For indoor work, exposure was assumed to be atmospheric heat in the shade without effective air conditioning. As the impact of heat on labour capacity also depends on clothing, light clothing for all was assumed, and a metabolic rate related to the required physical activity.

For outdoor work, it is also necessary to take the heating effect of the sun into account. The full Liljegren formula for calculating WBGT in the sun was employed for one year (2010) for all grid cells.[18] This additionally required hourly ERA5 surface pressure, surface solar radiation downwards, and total sky direct solar radiation at surface. A good approximation was found for the difference between the value of WBGT in the sun and WBGT in the shade (a WBGT *uplift)*. Tested in warm to hot Koppen climate regions, this uplift was 0.0035 * ssrd, which matched the Liljegren WBGT in-sun calculation to ±0.2 C. As the Liljegren WBGT calculation also requires air speed, 1 m/s was taken as the minimum apparent wind speed generated by the movement of arms and legs during work.

The function relating WLF (the fraction of potential work time lost to heat exposure) to an hourly WBGT level is given by the cumulative normal distribution (ERF) function:

$$Loss\ fraction = \frac{1}{2}\left(1 + ERF\left(\frac{WBGT_{hourly} - WBGT_{avg}}{WBGT_{SD} \times \sqrt{2}}\right)\right)$$

where $WBGT_{avg}$ and $WBGT_{SD}$ are the constants in the function for a given activity level

Table 4.

Table 4: Constant values for labour loss fraction calculation.

**Metabolic rate**	**$WBGT_{avg}$**	**$WBGT_{SD}$**
200 Watts	35.5	3.9
300 Watts	33.5	3.9
400 Watts	32.5	4.2

For each grid cell, each of the six hourly WLFs (three different metabolic rates, two different working situations) was summed between 7am and 6pm solar time, every day of the year, to derive estimates of annual WHL (potential work hours lost annually) per worker using that metabolic rate and in that exposure situation.

For example:

$$AnnualWHLpp200Wshade_{cell} = \sum_{day=1}^{365} \sum_{hour=07}^{18} WLF200W_{day,hour}$$

Also for each cell, an estimate of the number of workers in each sector is derived from the cell's working age population (15+ years old; commensurate with ILOSTAT data) together with the (country's) proportion of those working in each of the four sectors: agriculture, construction, manufacturing, and "other" sectors (which includes workers in the service sector). In grid cells that overlap country borders, the working age population is apportioned to each of the countries involved based on each country's share of the total population within the cell.

For example:

$$\text{ManufPopn}_{country,cell} = \text{Popn15plus}_{cell} \times \text{PopnFraction}_{country,cell} \times \text{ManufFraction}_{country}$$

To calculate potential work hours lost by sector, each sector is assigned to a metabolic rate and to sun, or indoors/shade conditions as shown in Table 5.

Table 5: Employment sector to metabolic rate assignment.

**Metabolic rate:**	200W (shade),<br>light work	300W (shade),<br>moderate work	400W (sun),<br>heavy labour
**Employment sector:**	Other<br>(mainly services)	Manufacturing	Agriculture +<br>Construction

Finally, cell-by-cell annual data is aggregated to country annual worker-population-weighted WHL totals for each sector. Country populations, including the number of workers in each sector are aggregated at the same time.
For example:

$$\text{AnnualWHL}_{country,service} = \sum_{cell=all}^{in-country} \left(\text{ServicePopn}_{country,cell} \times \text{AnnualWHL200Wshade}_{cell} \right)$$

And:

$$\text{Total AnnualWHL}_{country} = \text{AnnualWHL}_{country,service} + \text{AnnualWHL}_{country,manufacturing} + \text{AnnualWHL}_{country,agriculture} + \text{AnnualWHL}_{country,construction}$$

For each country and year, the average annual potential work hours lost per employed person (WHLpp) is arrived at by dividing the total annual country WHL by number of employed people in all four sectors for that year. The same method can be used to derive for WHLpp for groups of countries, all useful because they exclude the signals generated by increasing population.

Data

- Climate data from the European Centre for Medium-Range Weather Forecasts (ECMWF) ERA5 reanalysis.[19]
- Population data from the NASA Socioeconomic Data and Applications Center (SEDAC) Gridded Population of the World (GPWv4).[20]
- Sector employment data from ILOSTAT.[21]
- Future climate projections from ISIMIP 3b protocol.[22]

Caveats

The relative proportions of agricultural, construction, manufacturing, and other sector workers are only reported at country level. These proportions are applied unmodified to working age populations in all grid cells within a country and thus the analysis does not capture probable differences in the proportions between one grid cell and next within the same country.

Analysis performed with the above-described methodology has shown that the ERA5 reanalysis data regularly understate maximum air temperatures. The ERA5 deviation from the ensemble average of several other data sources varies by location, but is generally in the order of 1–4°C lower and is especially pronounced in some coastal regions.[23] Combined with often high population concentrations near the coast, the WHL results presented here are conservative. As a comparison, when applying the WHL calculations to climate data input sourced from ISIMIP or weather stations, WHL estimates increase by 40%.

Differences between disparate climate datasets, ERA5 reanalysis single levels and ISIMIP5, have been compensated for when combining projections with historical data (see tail-end in the Methods section).

Future form of the indicator

With climate change now prominent, there is renewed interest in safe work hours (here SWH) and threshold-limit values for working in the heat [ILO 2019, World Economic Forum 2023].[24,25] Both potential work hours lost (WHL) and SWH limits as specified by ISO [2017] and NIOSH [2016] take account of both ambient WBGT and metabolic rate so it might be assumed that they are related in a simple way.[26,27] Perhaps, for example, SWH and work hours not lost should be comparable. This isn't the case, although the underlying science is similar.

The exposure-response curve used here in calculating productivity (measured as a fraction of "potential work hours lost") comes from epidemiological data for groups of acclimatised workers that measured productivity directly while the SWH threshold-limits are based on individual data determined in a laboratory.[28,29] International standards (ISO and NIOSH) both use limits (RELs) for example as a (regulatory, managerial, or public health) protection measure, somewhat similar to uses of the simpler heat indices HI and Humidex.

However, because the NIOSH and ISO international standards for occupational exposure to heat and hot environment are recognised world-wide (though their widespread application in the workplace is

limited) it is expected that this indicator will be based on their underlying science in future years. Productivity makes a good indicator for similar reasons.[30]

Additional analysis

Occupational heat exposure leads to another aspect of social and health inequity: the difference between the impacts on the people in labouring jobs that require high physical intensity and those in office or service jobs with less straining work. The impact of heat exposure on labour capacity increases significantly with the physical intensity of the work and significantly again if the work is out under the sun (Figure 20). Agricultural workers are the worst affected in many countries, with the burden often shifting to those in construction in higher income countries, such as the USA.

In 2023 the average potential work hours lost per employed person is lower than in 1990-1999 in several countries, for example in Bangladesh (

Table 6). While weather variability year on year can be expected, and 2023 data on its own cannot be taken as an estimate of trends, a reduction in the agricultural workforce, mainly in favour of the service industry, is the main driver of long-term static or negative trends in WHL in a number of countries.

Table 6: Annual heat-related potential work hours lost (WHL) per employed person in populous countries.

	ISO3 code	Human Development Index (Level)	Work hours lost per employed person 1991-2000	Work hours lost per employed person in 2023	Billions of work hours lost in 2023	% of global
Global			140·2	148·3	512·4	
Bangladesh	BGD	Medium	392·0	358·3	26·4	5·2%
Pakistan	PAK	Low	337·2	345·8	26·2	5·1%
India	IND	Medium	338·6	332·4	181·6	35·4%
Nigeria	NGA	Low	269·6	259·6	19·9	3·9%
Philippines	PHL	Medium	264·3	231·3	11·2	2·2%
Indonesia	IDN	High	241·3	210·5	27·4	5·3%
Brazil	BRA	High	88·5	67·6	6·6	1·3%
China	CHN	High	88·0	61·0	45·0	8·8%
Ethiopia*	ETH	Low	40·1	41·5	2·1	0·4%
Japan	JPN	Very High	22·7	36·1	2·2	0·4%
United States of America	USA	Very High	15·2	20·0	3·4	0·7%
Russian Federation	RUS	Very High	2·1	2·4	0·2	0·0%
Rest of the world			101·2	121·4	160·2	31·3%

*The low impact here reflects the cooler high–altitude climate of much of Ethiopia

A comparison of global total potential work hours lost (WHL) across the workforce sectors chosen shows that Agriculture is still predominant (Figure 19). However total annual Agricultural WHL now stays largely constant due to reductions of the agricultural workforce in many Low and Middle HDI countries. Of the four sectors, the impact of increasing heat is being felt more in Construction and the "Other" sector (mainly in the service industry).

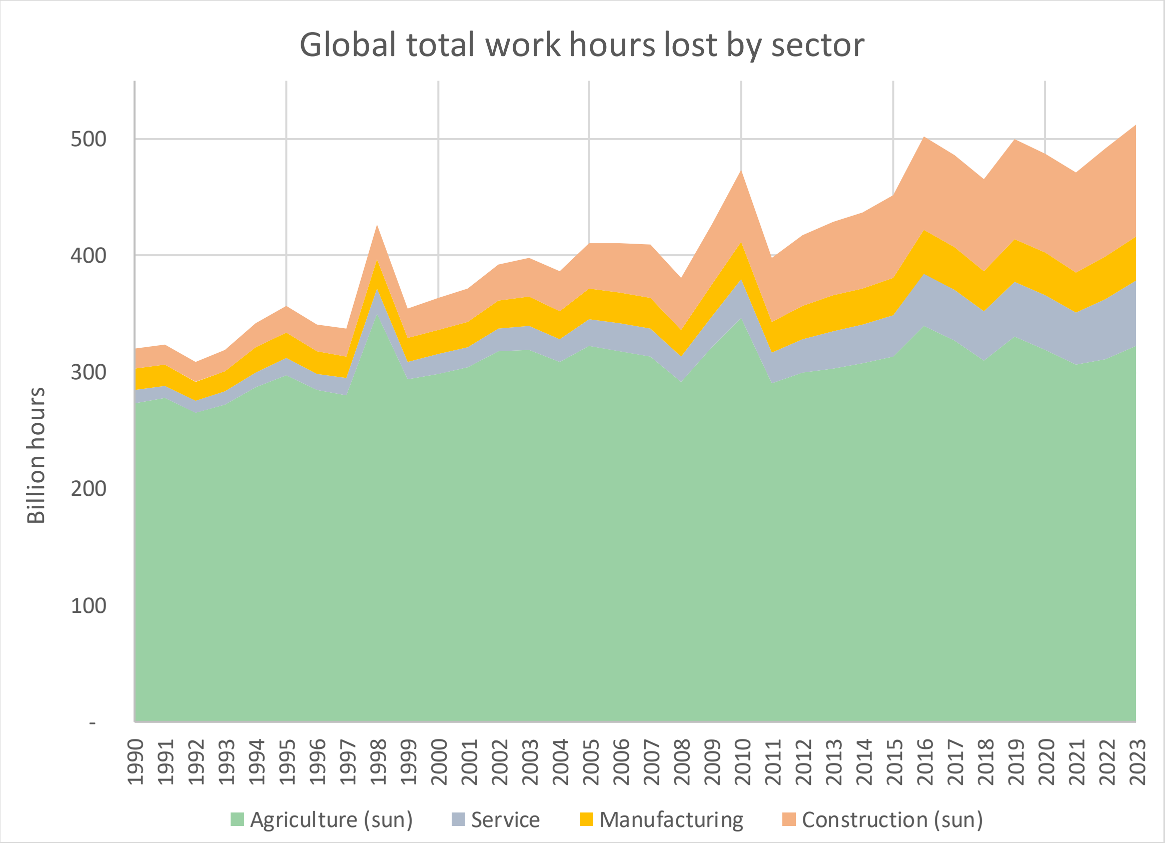

Figure 19: Global potential work hours lost (billions) due to heat by employment sector, 1990–2023.

Because of its definition, WHL is influenced by changes in population numbers and the distribution of the workforce within countries as well as climate change. Figure 20 shows trends in global WHLpp attributable to climate alone. In addition, this chart shows different trends when an agricultural worker (400W metabolic rate) is working in the shade or in the sun. The doubling of the loss when solar radiation is included provides an illustration of how disproportionate an increase by a few degrees of WBGT (solar *uplift* outlined above, typically between 1 and 2.5 degrees) disproportionately affects WHL and WHLpp.

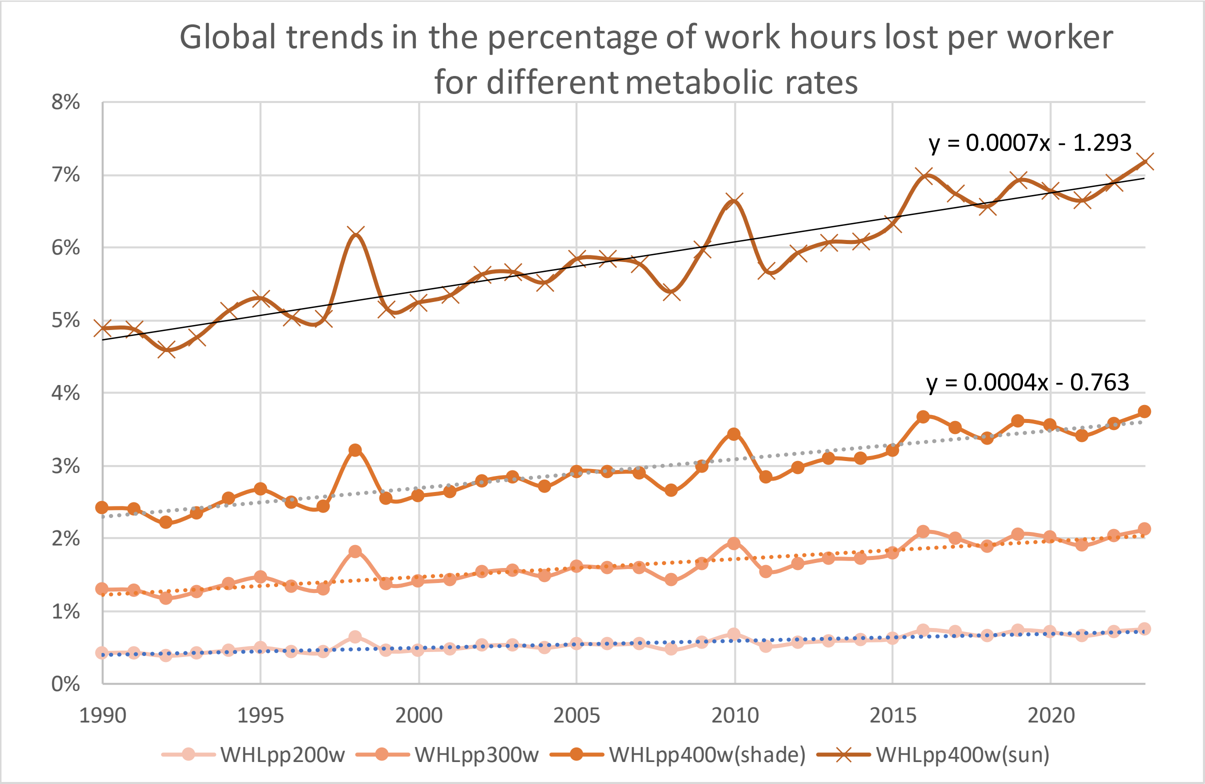

Figure 20: Trends in global mean potential work hours lost per employed person (population signal excluded).

Looking now at trends in global potential work hours lost by HDI groupings, Figure 21 provides a striking illustration of how Low and Middle HDI countries are bearing an increasing share of an increasing problem. Low and Middle level HDI countries' combined share of the world's potential working hours lost due to heat in 2023 is now 70%, up from 60% in 1990.

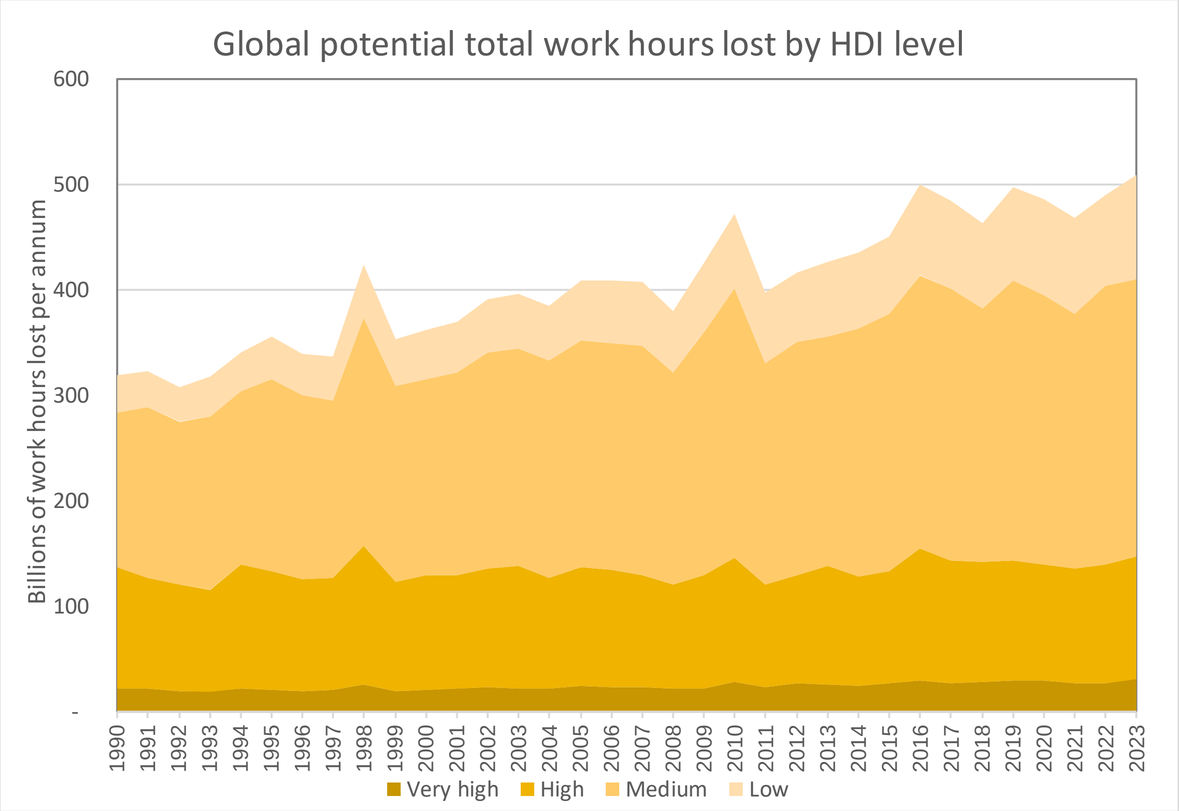

Figure 21: Trends in global total potential work hours lost by HDI group.

1.1.3.2 Outdoor workers

Indicator Authors

Dr Natalie C Momen and Dr Frank Pega

Methods

Since their establishment in 2016, the Joint Estimates of the World Health Organization (WHO) and the International Labour Organization (ILO) of the Work-related Burden of Disease and Injury (WHO/ILO Joint Estimates) are the official occupational burden of disease estimates of the United Nations.[31–33] WHO and the ILO produce these estimates at the global, regional and national/area levels, disaggregated by both sex and age group.[31–33] In 2023, the organisations launched a new set of WHO/ILO Joint Estimates that reported the first official estimates of the proportion of the global, regional, and national/area-level populations who are occupationally exposed to solar ultraviolet radiation for 195 countries/areas and for the years 2000, 2010, and 2019.[34] The data sources and methods for this estimation have previously been described in detail,[11,34] but briefly, WHO and the ILO, with the support of the individual experts in the WHO Technical Advisory Group on Occupational Burden of Disease Estimation,[35] have estimated this exposure via proxy of occupation with job tasks conducted outdoors.[34] Using a job-exposure matrix[36] developed specifically for the WHO/ILO Joint Estimates (see annex 1 in Pega et al 2023),[34] codes of the International Standard Classification of Occupations 2008 (ISCO-08)[37] (cross-walked to the International Standard Classification of Occupations 1988 codes),[38] were used to identify occupations with outdoor work

(assigned to the exposure category of "any [or high] occupational exposure to solar ultraviolet radiation", or outdoor worker) versus those without outdoor work (assigned to the exposure category of "no [or low] occupational exposure to solar ultraviolet radiation", or indoor worker).[5]

Standard multi-level models[39] (applied by WHO in previous estimations[40–43] and to produce Sustainable Development Goal indicators)[44] were used to estimate the proportion of the population of working age (defined as ≥15 years) who were occupationally exposed to solar ultraviolet radiation for the estimation year.[34] Input data included 166 million observations from 763 official labour force surveys collected by national statistical offices in 96 countries/areas between 01 January 1996 and 31 December 2021 (for a list of the included surveys by country/area and year see annex 1 in Pega et al 2023)[34]. Globally, data from at least one survey were available for almost half (49.2%) of all countries/areas (Table 7) and over one third (35.9%) of the total working-age population. In each WHO Region, data from at least one survey covered over one third (38.0%) of countries/areas (Table 7) and almost one third (30.7%) of the population; the only exception was that data were available for only 6.0% of the population in the WHO Western Pacific Region.

Table 7: Numbers of surveys and countries/areas covered by the WHO/ILO Joint Estimates of occupational exposure to solar ultraviolet radiation.

	**WHO region**						**World**
	**African Region**	**Region of the Americas**	**Eastern Mediter-ranean Region**	**European Region**	**South-East Asia Region**	**Western Pacific Region**	
Countries/areas (N)	47	35	22	53	11	27	195
Surveys (N)	69	168	41	391	49	45	763
Countries/areas with ≥1 survey (N) (% of countries)	18 (38.0%)	15 (42.9%)	9 (40.9%)	33 (62.3%)	8 (72.7%)	13 (48.1%)	96 (49.2%)

Source: annex 1 in Pega et al 2023.4

As in the 2023 report of the *Lancet* Countdown on health and climate change,[11] to calculate the percentage of the working-age population who were outdoor workers for the year 2023, point prevalences of the proportion of the population occupationally exposed to solar ultraviolet radiation were sourced from annex 1 in Pega et al 2023[34] for the years 2000, 2010 and 2019 and modelled at the level of the individual cohort defined by sex and five-year age group applying a linear function. Also as in the 2023 report,[11] to calculate the number of the working-age population who were outdoor workers for the year 2023, for each individual cohort, its proportion of outdoor workers was multiplied with its total number of population, sourced from the United Nations' official population estimates.[45] This produced these two sets of estimates of the proportion and number of outdoor workers for the world, the six WHO Regions, the seven *Lancet* Countdown regions, and 195 countries/areas (Table 8). These sets of estimates were also produced disaggregated by sex (three categories: females and males; females; males) and by age group (≥15 years, and 17 categories of five-year age groups: 15-19, 20-24, ..., 90-94, ≥95 years).

Table 8: Countries/areas covered by the indicator by WHO region.

WHO Region (Number of countries/areas)	Country or area
**African Region (47)**	Algeria; Angola; Benin; Botswana; Burkina Faso; Burundi; Cabo Verde; Cameroon; Central African Republic; Chad; Comoros; Congo; Côte d'Ivoire; Democratic Republic of the Congo; Equatorial Guinea; Eritrea; Eswatini; Ethiopia; Gabon; Gambia; Ghana; Guinea; Guinea-Bissau; Kenya; Lesotho; Liberia; Madagascar; Malawi; Mali; Mauritania; Mauritius; Mozambique; Namibia; Niger; Nigeria; Rwanda; Sao Tome and Principe; Senegal; Seychelles; Sierra Leone; South Africa; South Sudan; Togo; Uganda; United Republic of Tanzania; Zambia; and Zimbabwe
**Region of the Americas (35)**	Antigua and Barbuda; Argentina; Bahamas; Barbados; Belize; Bolivia (Plurinational State of); Brazil; Canada; Chile; Colombia; Costa Rica; Cuba; Dominica; Dominican Republic; Ecuador; El Salvador; Grenada; Guatemala; Guyana; Haiti; Honduras; Jamaica; Mexico; Nicaragua; Panama; Paraguay; Peru; Saint Kitts and Nevis; Saint Lucia; Saint Vincent and the Grenadines; Suriname; Trinidad and Tobago; United States of America; Uruguay; and Venezuela (Bolivarian Republic of)
**Eastern Mediterranean Region (22)**	Afghanistan; Bahrain; Djibouti; Egypt; Iran (Islamic Republic of); Iraq; Jordan; Kuwait; Lebanon; Libya; Morocco; occupied Palestinian territory, including east Jerusalem; Oman; Pakistan; Qatar; Saudi Arabia; Somalia; Sudan; Syrian Arab Republic; Tunisia; United Arab Emirates; and Yemen
**European Region (53)**	Albania; Andorra; Armenia; Austria; Azerbaijan; Belarus; Belgium; Bosnia and Herzegovina; Bulgaria; Croatia; Cyprus; Czechia; Denmark; Estonia; Finland; France; Georgia; Germany; Greece; Hungary; Iceland; Ireland; Israel; Italy; Kazakhstan; Kyrgyzstan; Latvia; Lithuania; Luxembourg; Malta; Monaco; Montenegro; Netherlands; North Macedonia; Norway; Poland; Portugal; Republic of Moldova; Romania; Russian Federation; San Marino; Serbia; Slovakia; Slovenia; Spain; Sweden; Switzerland; Tajikistan; Türkiye; Turkmenistan; Ukraine; United Kingdom; and Uzbekistan
**South-East Asia Region (11)**	Bangladesh; Bhutan; Democratic People's Republic of Korea; India; Indonesia; Maldives; Myanmar; Nepal; Sri Lanka; Thailand; and Timor-Leste
**Western Pacific Region (27)**	Australia; Brunei Darussalam; Cambodia; China; Cook Islands; Fiji; Japan; Kiribati; Lao People's Democratic Republic; Malaysia; Marshall Islands; Micronesia (Federated States of); Mongolia; Nauru; New Zealand; Niue; Palau; Papua New Guinea; Philippines; Republic of Korea; Samoa; Singapore; Solomon Islands; Tonga; Tuvalu; Vanuatu; and Viet Nam

Data

- WHO/ILO Joint Estimates of the working-age population occupationally exposed to solar ultraviolet radiation, sourced from Pega et al 2023[34]
- United Nations projections under the medium scenario of the total number of the working-age population, sourced from the 2022 Revision of the World Population Prospects[45].

Caveats

The percentage and the number of outdoor workers for the year 2023 were estimated under the assumption that these variables have a linear trend over time. This assumption is commonly applied across many official and non-official estimations of occupational and non-occupational risk factor exposures, including in the WHO/ILO Joint Estimates across various and diverse occupational risk factor exposures, such as occupational exposures to solar ultraviolet radiation[34] and to long working hours.[43]

All labour force surveys used as input data to produce the estimates capture workers in the formal economy, but only some of these surveys also collect data from workers in the informal economy. The estimates are consequently only representative of workers in the formal and informal economies for countries with collections of data from both workers in the formal and those in the informal economy.

Additional analysis

A total of 1.6 billion people of working age were estimated to be outdoor workers globally in 2023, representing 25.9% of working-age people. However, the number and the percentage of outdoor workers varied by WHO Region, country/area, sex and age group.

In absolute terms, the largest number of outdoor workers of working age was estimated to be in the WHO South-East Asia (0.5 billion) and the WHO Western Pacific Regions (0.4 billion) (figure 22). However, the largest percentage of the population working outdoors were estimated to be in the WHO African (31.8%) and South-East Asia Regions (28.9%) (figure 23). The number and proportion of outdoor workers by country/area are presented in figure 24 and figure 25, respectively.

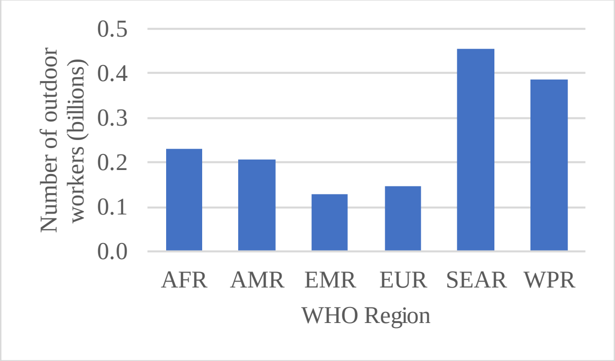

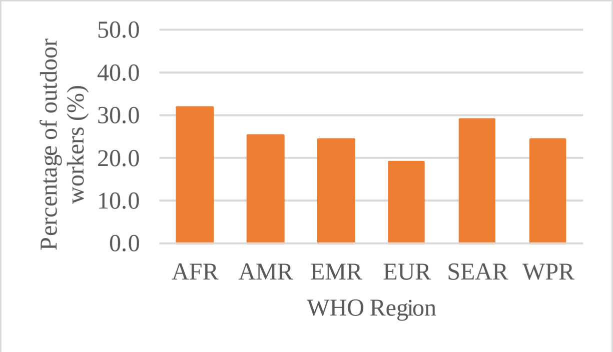

Figure 26: Number and percentage of outdoor workers by WHO Region, population of working age (≥15 years), 195 countries/areas, 2023 (WHO estimates).

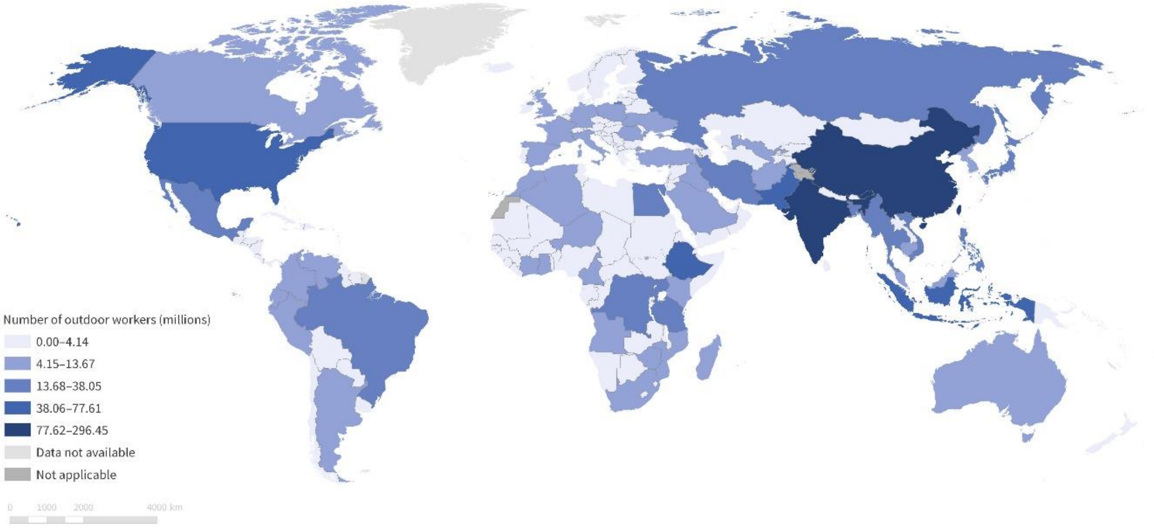

Figure 27: Number of outdoor workers, population of working age (≥15 years), 195 countries/areas, 2023 (WHO estimates).

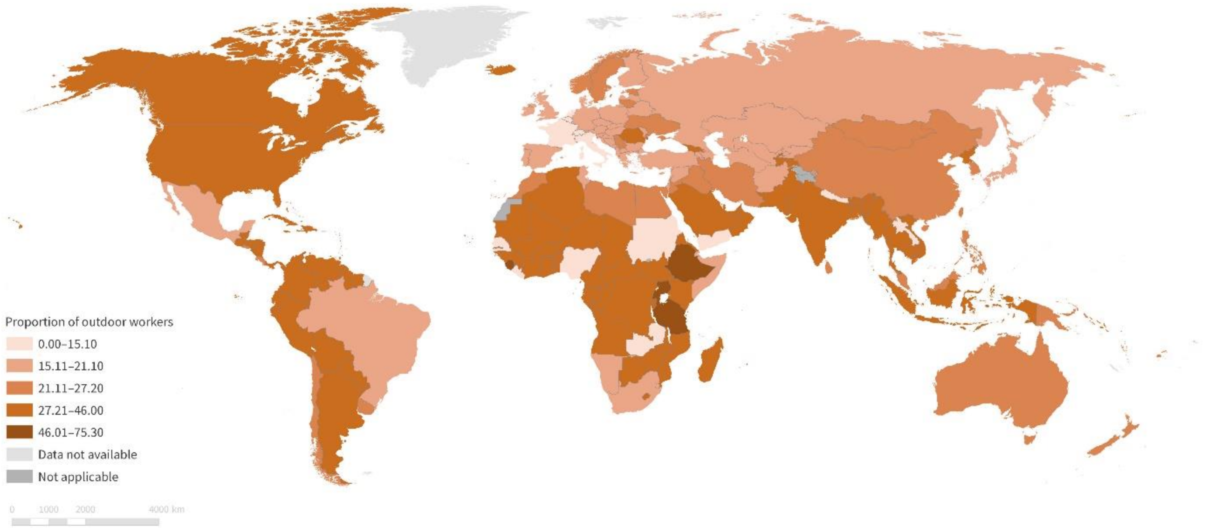

Figure 28: Percentage of outdoor workers, population of working age (≥15 years), 195 countries/areas, 2023 (WHO estimates).

Globally in 2023, being an outdoor worker was more common among males than among females. Three of every four outdoor workers were estimated to be males (1.1 billion), and only one of every four was estimated to be female (0.4 billion). This represented 37.9% of all working-age males, compared with only 14.0% of all working-age females. Regionally in 2023, sex differences in the numbers and the percentages of outdoor workers were also observed (figure 29 and figure 30). The WHO South-East Asia and the WHO Western Pacific Regions had the largest differences between females and males for the number of outdoor workers in absolute terms, whereas the WHO Eastern Mediterranean Region and the WHO Region of the Americas had the largest differences in relative terms (figure 31). Similarly, the WHO Eastern Mediterranean Region and the WHO Region of the Americas had the largest sex differences in the percentage of outdoor workers, both in absolute and in relative terms (figure 32). The smallest differences by sex in the number and the percentage of outdoor workers were estimated for the WHO African Region. Compared with in the other WHO Regions, a higher percentage of females worked outdoors in this WHO Region, resulting in equally high exposure point prevalence among the female sex as among the male sex (figure 33 and figure 34). However, in all regions, outdoor work was consistently more common in terms of numbers and of percentages among males than among females.

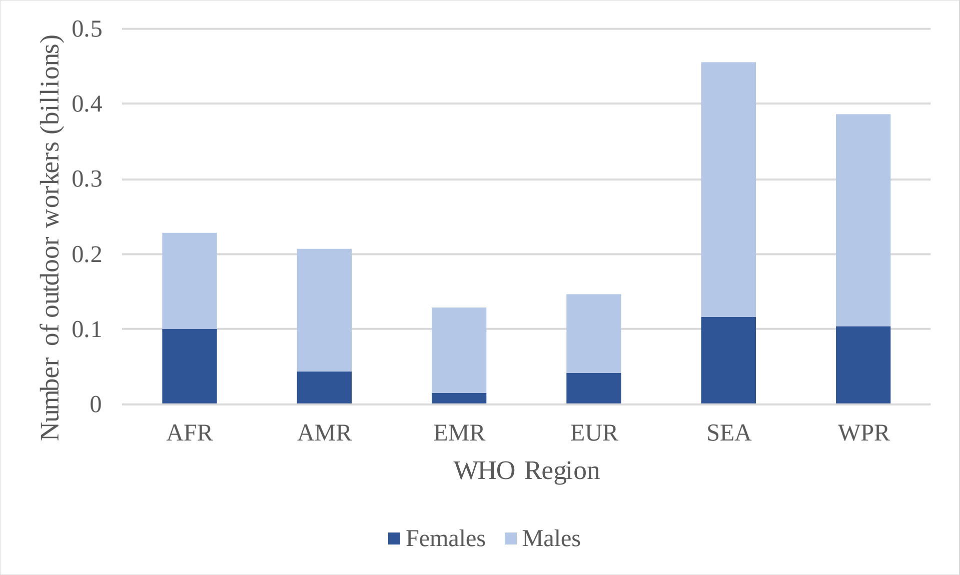

Figure 35: Number of outdoor workers by WHO Region and by sex, population of working age (≥15 years), 195 countries/areas, 2023 (WHO estimates).

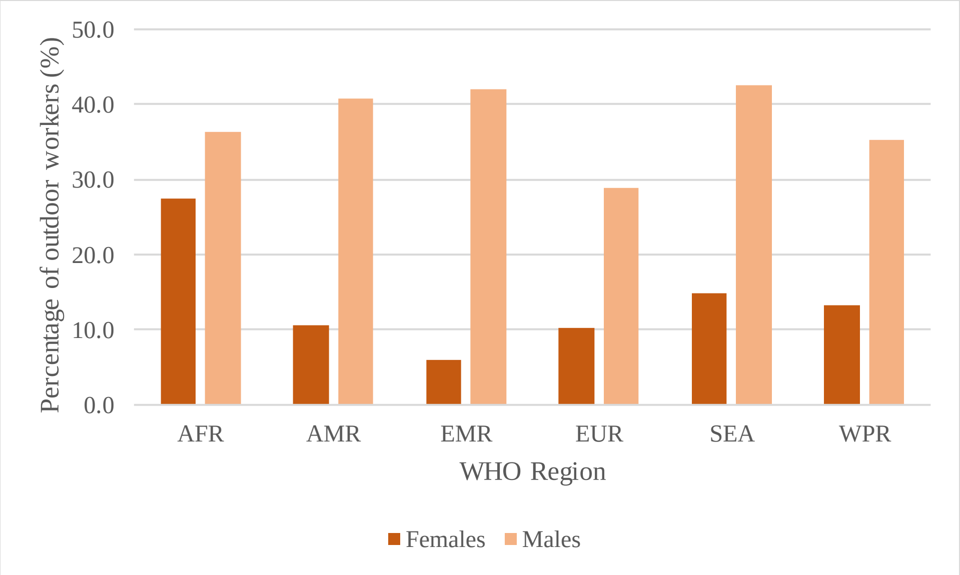

Figure 36: Percentage of outdoor workers by WHO Region and by sex, working-age population (≥15 years), 195 countries/areas, 2023 (WHO estimates).

Furthermore, clear differences existed in outdoor worker numbers and percentages by age group. The largest numbers and percentages of outdoor workers were estimated in the age groups of early and middle adulthood, in all WHO Regions (figure 37 and figure 38).

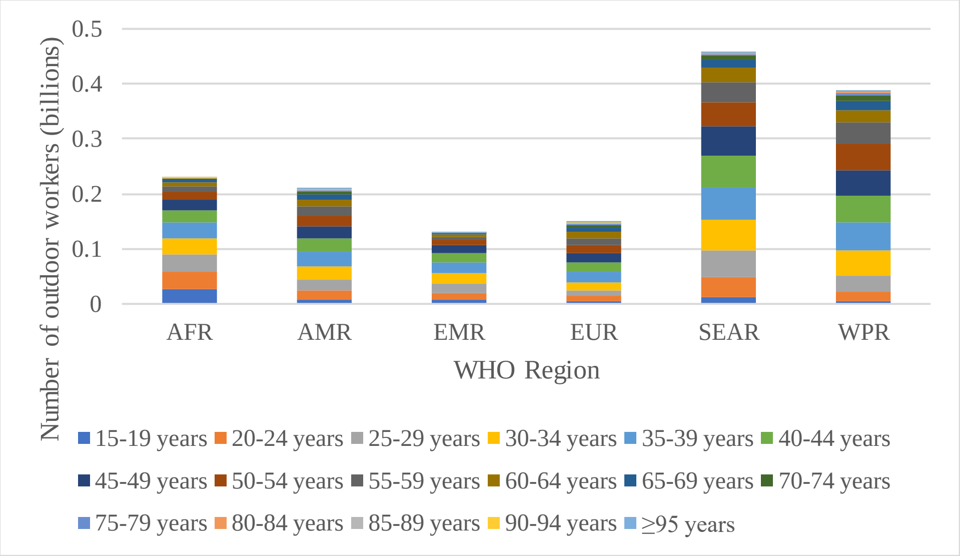

Figure 39: Number of outdoor workers by WHO Region and by age group, working-age population (≥15 years), 195 countries/areas, 2023 (WHO estimates).

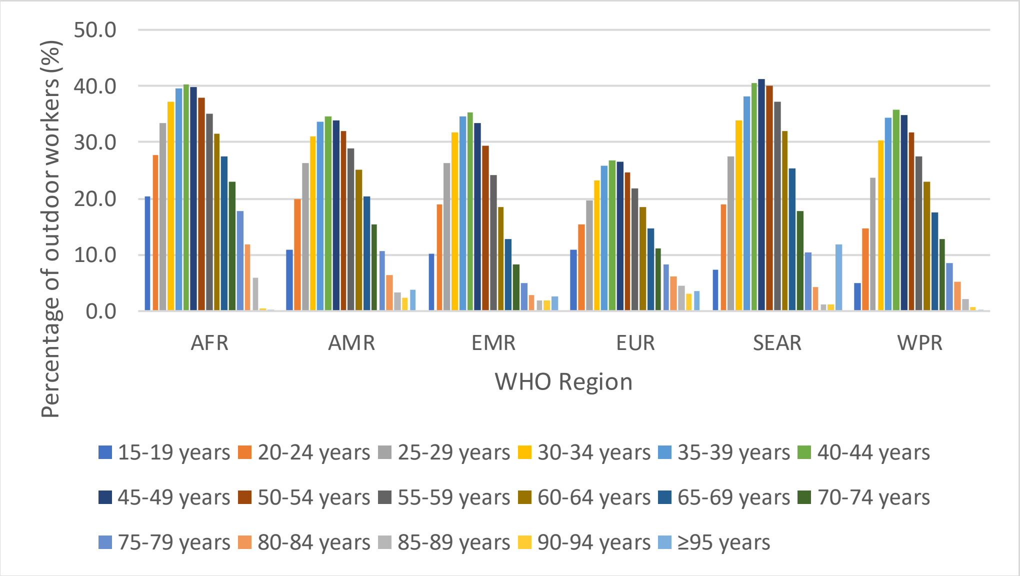

Figure 40: Percentage of outdoor workers by WHO Region and by age group, working-age population (≥15 years), 195 countries/areas, 2023 (WHO estimates).

Globally between the years 2000 and 2023, reductions were estimated in both the number (-0.2 billion) and the percentage (-15.8 percentage points) of outdoor workers of working age. These changes differed by WHO Region. In terms of absolute numbers, the reduction in the number of outdoor workers by 0.3 billion outdoor workers in the WHO Western Pacific Region drove the global reduction (figure 41), which was almost entirely due to the reduction in the number of outdoor workers in one country: the People's Republic of China (-0.3 billion) (figure 42). There were increases in the numbers of outdoor workers by 0.1 billion in the WHO Eastern Mediterranean Region and by <0.1 billion in the WHO's European Region, Region of the Americas and African Region.

The WHO Western Pacific Region had the largest estimated reduction in the proportion of outdoor workers (32.9 percentage points), followed by the WHO African Region (24.1 percentage points) (Figure 44). Again, trends in specific countries drove these regional trends. A 33.6 percentage point reduction was estimated for the People's Republic of China, and a 90.7 percentage point reduction

was estimated for Nigeria. Increases were estimated for the WHO Regions of Europe (by 4.1 percentage points) and the Eastern Mediterranean (by 0.6 percentage points). Estimated changes in the percentages of outdoor workers at the national level are shown in figure 43.

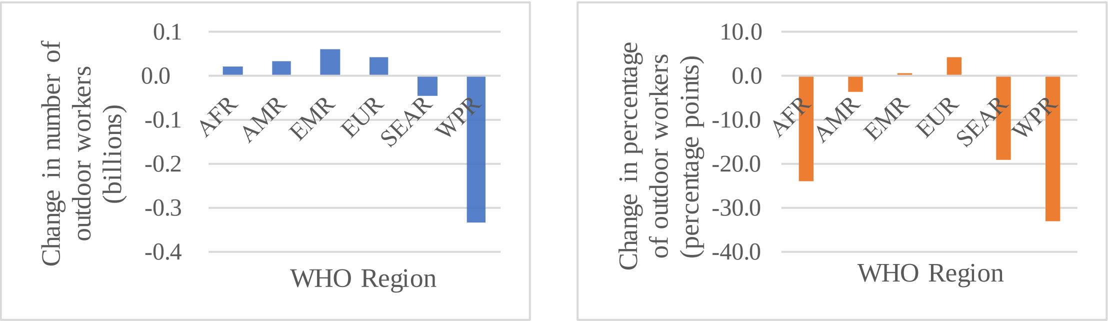

Figure 44: Change in the number and percentage of outdoor workers between the years 2000 and 2023 by WHO region, working-age population (≥15 years), 195 countries/areas (WHO estimates).

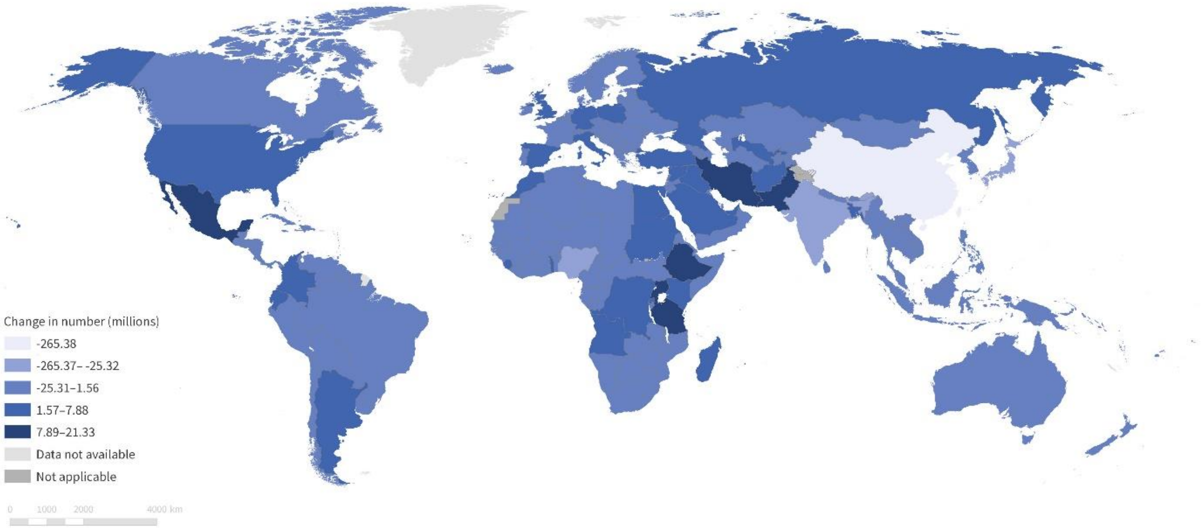

Figure 45: Change in the number of outdoor workers between the years 2000 and 2023, working-age population (≥15 years), 195 countries/areas (WHO estimates).

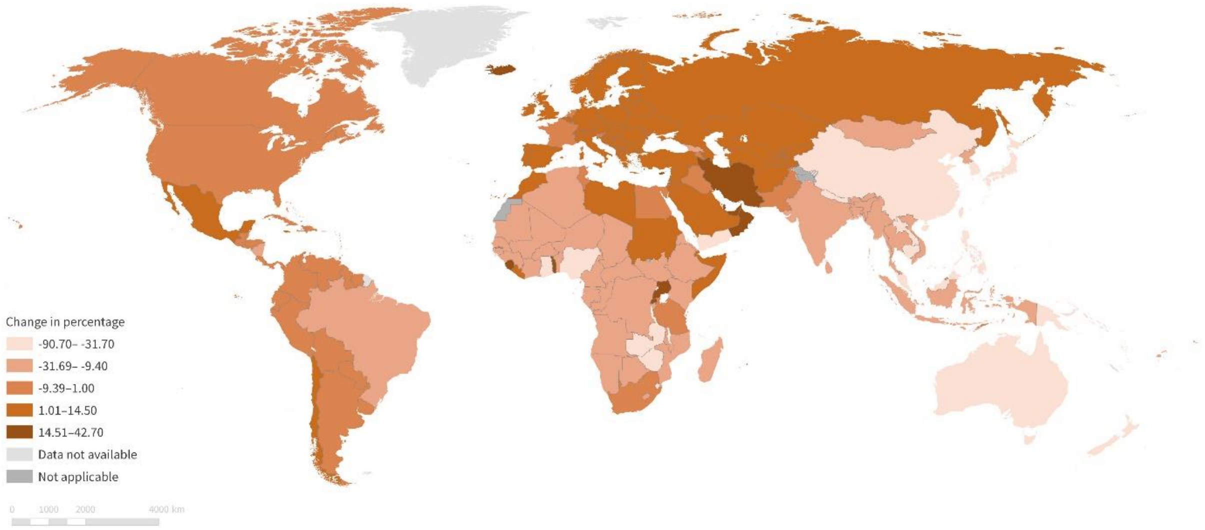

Figure 46: Change in the percentage of outdoor workers between the years 2000 and 2023, working-age population (≥15 years), 195 countries/areas (WHO estimates).

Indicator 1.1.4: sleep loss from higher nighttime temperatures

Indicator Authors

Dr Kelton Minor, Dr Nick Obradovich

Methods

This indicator tracks the relationship between human sleep and warmer nighttime temperatures. Across multiple settings, warmer nighttime temperatures have been shown to produce significant harms to the duration and timing of human sleep patterns. Given that sleep is a vital component of overall human health and well-being[46–52], tracking the effect of warming nighttime temperatures on human sleep is an important step in tracking the overall burden of climatic changes on human health and wellbeing. Further, sleep interventions are one of the more concrete policy measures that can be taken to reduce the health hazards presented by hot nighttime temperatures. And given that sleep mediates a myriad of other health-related outcomes, tracking the impact of warming temperatures on sleep over time can provide useful insight into its potential linkages to other health-related variables of concern — e.g., climate-related decrements to mental and physical health.[53–61]

In the course of its analysis, this indicator employs estimates from published research that employs fitness tracking bands to measure over ten billion sleep observations across over 40,000 individuals living in 68 countries around the world from 2015–2017 to estimate the precise impact of nighttime temperatures on human sleep.[62] This work built off of previous research that established the causal link between warmer nighttime temperatures and human sleep in the context of the United States.[63]

Additional prior research has corroborated the causal nature of the effect: warmer ambient temperatures produce worsened sleep.[60,64,65] Further, a systematic review of the temperature and sleep epidemiological literature finds globally consistent evidence that higher temperatures worsen sleep, a relationship that persists across different sleep outcomes (sleep duration, sleep efficiency, sleep

quality) different methods of sleep measurement (device-based, self-report, sensor-based), different climate regions, and different study designs including a growing body of quasi-experimental research.[66] This indicator employs precisely measured and rigorously estimated parameters linking nighttime temperatures to sleep outcomes to track the impact of warmer nighttime temperatures on human sleep globally.

The manuscript underlying this work flexibly estimated the nighttime temperature and sleep relationship with separate binned temperature indicator terms and followed best practices in climate econometrics[67] — including the careful selection of a broad range of fixed effects in the models — to ensure that estimates isolated the causal relationship between nighttime temperatures and human sleep. This indicator statistically controls for individual-level demographic and environmental factors including air conditioning access across the globally extensive sample of wristband users and climate regions including many areas with high AC penetration. A recent systematic review also summarized evidence that sleep loss associated with rising temperatures is evident in areas and among households with AC,[68] and prior evidence indicates that sleep loss also occurs due to warmer temperatures at levels well below those associated with typical AC use.[69,70] These methods are detailed extensively in the manuscript[62] (under the Experimental Procedures section) and also in the supplementary materials for the manuscript. The results of this prior estimation are depicted in figure 47.

Specifically, this indicator tracks the estimated percentage change in annual total number of hours of sleep lost globally due to warmer than optimal nighttime temperatures relative to the 1986–2005 baseline average in this metric. Because the statistical estimates from the empirical work underlying this indicator were markedly similar in form between climatic regions and between countries, those empirical results enable extrapolation — with some definite limitations, detailed further below — to the global population of humans.

This indicator employs meteorological data from the ERA5 reanalysis project[71] from 1986–present (with a resolution of ~31km), population weights derived from gridded population data from NASA SEDAC[72]matched in resolution and extent to the ERA5 data), and semi-parametric estimates derived from the recent global study linking nighttime temperatures to human sleep loss.[62]

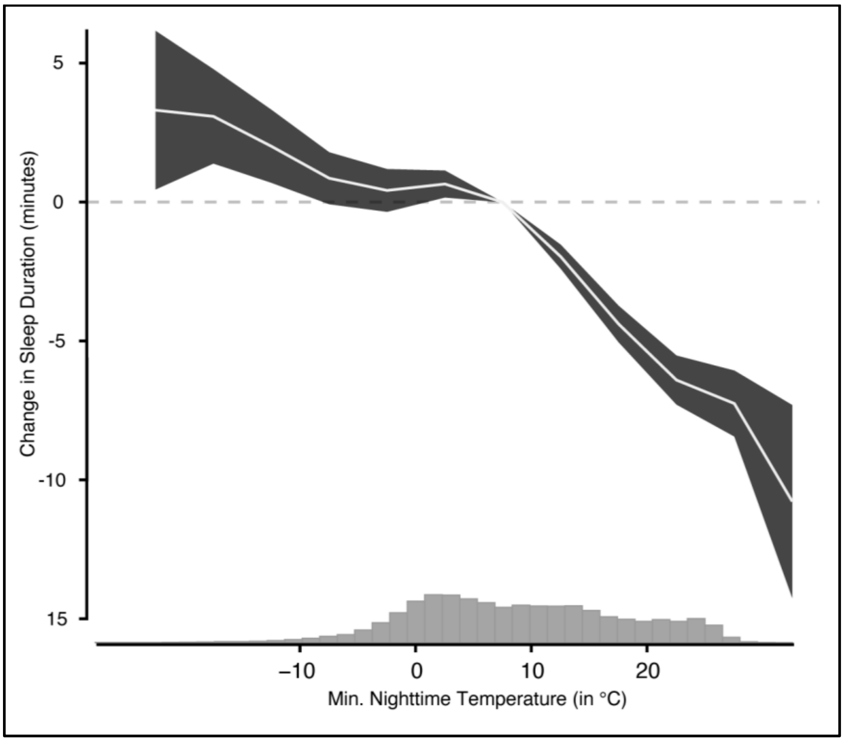

Figure 48: Reproduction of results from the main empirical specification[73] in depicting the relationship between nighttime temperatures and change in sleep duration. The empirical model employed 5°C nighttime temperature bins to semi-parametrically estimate the relationship of interest.

This indicator employs parameters drawn from this prior empirical work — and the coefficients from its linear spline estimated model — alongside ERA5 nighttime temperatures (daily minimum of hourly 2m temperatures) associated with each grid-cell-day globally from 1986–present. It then employs the linear spline coefficients from the underlying empirical model to calculate an estimated sleep loss value for each cell-day. It then sums these cell-day values to the cell-year to produce an unweighted cumulative annual impact of the nighttime temperatures across the year in that cell on sleep loss.

To ensure that the impacts are pertinent to the locations in which people live, the indicator then weights these cell-year values by employing population proportions derived from the 2000–2020 average of the global gridded population data — harmonised in projection and extent with the ERA5 raster data — producing an annual population-weighted nighttime temperature induced sleep loss value for each cell-year, globally. These values are calculated for the most recent decade in the data, as well as for the baseline period (1986–2005), and compute, for the most recent year, a percentage change relative to baseline sleep loss value that the indicator reports in its main raster image.

The indicator's grid-cell-year values are then cumulated globally for each year, resulting in an estimate of the global population-weighted nighttime temperature induced sleep loss for each year of interest. This statistic from the indicator is then employed to calculate the percentage change, compared to baseline, in the planetary nighttime temperature-to-sleep impact for the most recent year. The plain language translation for this indicator is: 'To what degree did warmer nighttime temperatures alter planetary sleep this year as compared to baseline effects?'

Data

- Climate data from the European Centre for Medium-Range Weather Forecasts (ECMWF) ERA5 reanalysis
- Population count data from the Gridded Population of the World, version 4, from the Center for International Earth Science Information Network at Columbia University
- Linear spline parameters drawn from the empirical estimation contained in[62]

Caveats

While this indicator has numerous upsides, particularly given that it is derived from a global high-resolution measure of the relationship between human sleep and nighttime temperatures, it has limitations as compared to the ideal indicator derived from the ideal data. First, the indicator's analytic approach assumes that the estimates obtained in the underlying global study of tens of thousands of individuals are indeed likely to apply to the individuals not within the sample. This is not an uncommon assumption, but it bears note in this context as the underlying original data may be less likely to include the world's poorest than a truly representative sample. The included sample of individuals is likely to have been wealthier on average than those who weren't in sample as these individuals were able to afford fitness trackers. Importantly, both of the two largest inferential studies on the topic,[62,63] observe that poorer individuals within the sample have larger relationships between warmer nighttime temperatures and their sleep. Thus, this caveat indicates that, if anything, this indicator may be *underestimating* the total amount of lost sleep globally due to nighttime warming.

Second, the relationship between ambient temperatures and human sleep may change somewhat in the future as individuals adapt and/or decompensate to altered future temperatures. An increased availability of air conditioning in the future, for example, might alter the global response curve. This caveat becomes more of a concern the further away future indicator years are from the underlying empirical model's fairly recent estimates. That said, since the underlying global sleep study included a spatially extensive panel featuring several global areas where air conditioning/fan penetration is already quite high, it is plausible that the estimates already reflect some expected adaptation, particularly at the higher end of the temperature distribution.[74]

The best way to address these caveats is to continue to expand upon research in this area. Scholars can continue to gather fitness band data to track sleep going forward, reaching an ever larger segment of the human population and allowing the indicator to use updated estimates as newer or better data is procured.

Future form of the indicator

Future versions of this indicator can, for example, incorporate the analysis of the impact of specific extreme heat events (e.g., Heatwave in India, etc.) on human sleep following the attribution methods and protocol developed for the sentiment indicator in the 2022 *Lancet* Countdown report. Further, future versions could update the estimates of the relationship between human sleep and temperature as new empirical studies become available.

Additional Analysis

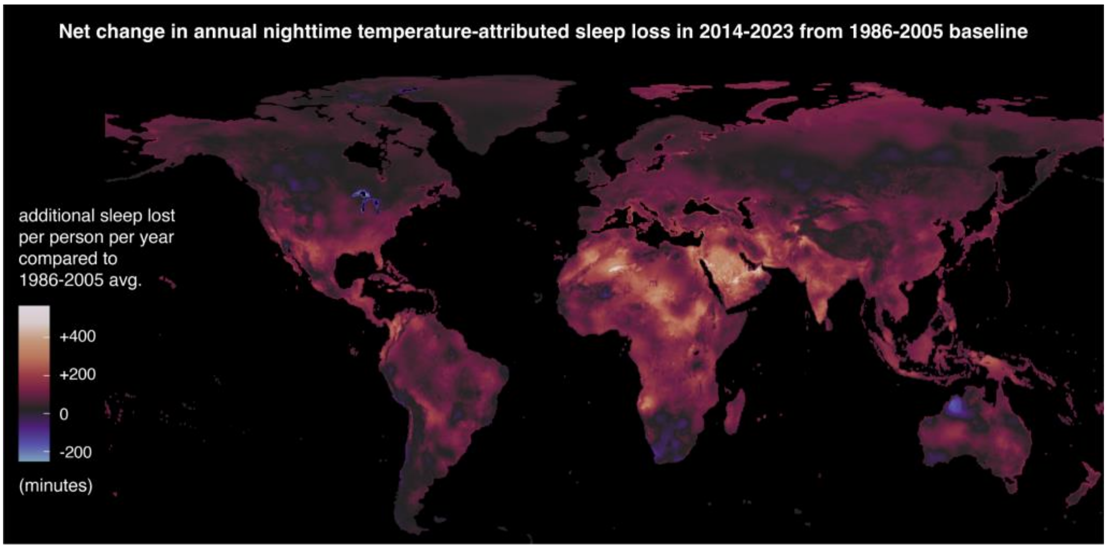

Figure 49: The average net change in annual temperature-attributed sleep loss in 2014–2023 compared to the 1986–2005 baseline. Values are presented in minutes for each raster cell in the map. As can be seen, because of the steeper slope of the relationship between warmer nighttime temperatures and sleep for already hotter regions, warmer regions of the world observed a greater estimated loss of sleep than in more temperate and cooler regions of the globe.

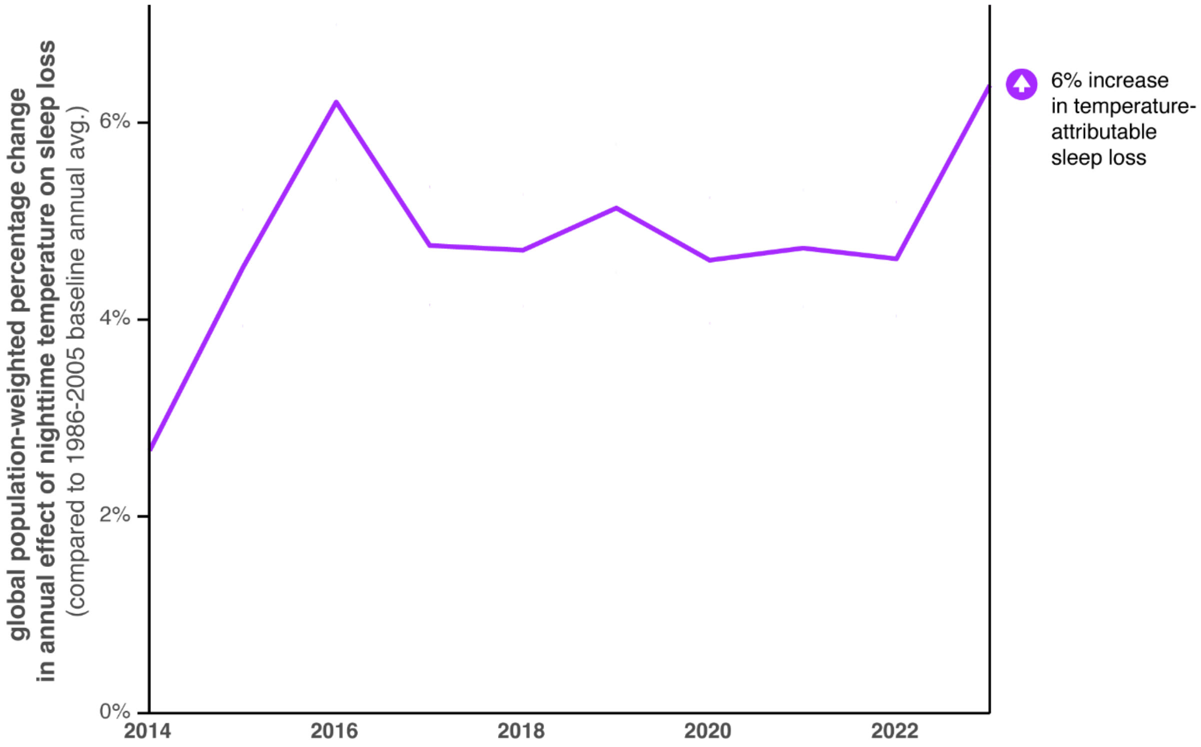

Figure 50: Global population-weighted percentage change in annual effect of nighttime temperature on sleep loss, per year.

Indicator 1.1.5: heat-related mortality

1.1.5.1 Attribution of human exposure to health-threatening temperatures

Indicator authors

Dr Andrew Pershing

Methods

Daily average 2m air temperature from ERA5 to calculate the 85th percentile temperature over the period 1986–2005 was used. This temperature is used as a proxy for the locally specific minimum mortality temperature (MMT). For each ERA5 grid point, the number of days in each year over the period 2014–2023 that exceed that threshold is calculated. The number of days above the MMT in the observed record is compared with the number in a counterfactual climate (described below) with no anthropogenic climate. The difference between the number of days in the observed and counterfactual datasets is the number that are attributable to human-caused climate change. For analysis, the number of people in that cell in 2020 according to the Gridded Population of the World-v4 is used to construct population-weighted averages for countries, regions, and human development index categories.

Daily temperatures are used from the Climate Shift Index attribution system[75] that implements a multi-method approach to climate change attribution.[76] The system begins by characterising the distribution of daily temperatures at each ERA5 grid point for the period 1991–2020. The distribution is estimated for 24 periods throughout the year and is parameterized as a skew-normal distribution.

The system then uses the observed linear relationship between the local temperature and global mean temperature (GMT) for each period. This relationship is calculated for 21 evenly-spaced quantiles between 0.1 and 0.99. By multiplying these slopes by GMT_{yr} - GMT_{ref}, the distribution can be shifted from the reference period (mean GMT 1991-2020 = 0.8°C) to the climate of year=*yr*. This is called the modern distribution. GMT_{yr} can also be replaced with GMT=0.0° to simulate the counterfactual climate. This strategy is used to calculate two pairs of modern and counterfactual climates. The first pair uses only the relationship between the median temperature and GMT. The resulting distributions have the same shape as the reference climate. The second pair uses the full set of quantiles. This allows the shape of the distribution to change.

In addition to the two empirical pathways, 24 paired climate model simulations are used from CMIP6. Each model pair consists of a single model forced with historical and projected radiative forcing (SSP3-7.0 or SSP5-8.5 if SSP3 is unavailable) and a model run under the pre-industrial control scenario. The modern climate for year=*yr* is the distribution of daily data in the forced simulation from a 31-year period centred on the year when the model's GMT first exceeds GMT_{yr}. The corresponding counterfactual climate is the same period from the control simulation. The 1991–2020 ERA5 data is used to de-bias each of the models.[76,77]

This procedure creates two empirical and 24 model-based pairs of modern and counterfactual climates. To compute the counterfactual for an observed temperature T, the quantile is found to be associated with this temperature in the modern climate. The temperature associated with this quantile in the counterfactual climate is found. This is the counterfactual temperature. The ensemble average counterfactual temperature is computed by averaging the temperatures from two empirical

approaches, averaging the temperatures from the 25 climate models, and then averaging the two averages.

Data

This indicator uses three data sources:

- ERA5 daily average 2m air temperatures[78]
- Daily counterfactual temperatures computed a multi-method approach
- Gridded Population of the World v4[79]
-

Caveats

The main caveat with this approach is related to the minimum mortality temperature. Using a single relative temperature (in this case, the 85th percentile) provides a convenient benchmark that can be applied everywhere. However, empirical studies find MMTs that differ slightly from city to city.[80] Furthermore, the analysis assumes that communities are adapted to the climate of 1986–2005 that was used to compute the MMT. The notion of a location specific MMT suggests that local adaptation is possible; however, it is not known how quickly a community's response to heat changes as it encounters warmer conditions. These complexities mean that our results are best interpreted as highlighting relative changes. Using a more recent reference period would increase the MMTs, reducing the number of MMT days. However, the relative patterns of exposure, for example, the relationship between attributable MMT days and HDI level.

1.1.5.2: Heat-related mortality

Indicator authors

Dr Zhao Liu

Methods

The methodology for this indicator, which tracks the global total number and spatial pattern of heat-related mortality from 2000 to 2023, remains similar to that described in the 2023 report of the *Lancet* Countdown.

The heat-related excess mortality in one day E is expressed as:

$$E = y_0 \times Pop \times AF \qquad (1)$$

where y_0 is the non-injury mortality rate on that day, *Pop* is the population size and *AF* is the attributable fraction on that day. Because every day's mortality rate is hard to obtain, y_0 is computed as the yearly non-injury mortality rate from the Global Burden of Disease data, divided by 365.[81]

AF is calculated via the relative risk (*RR*) which represents the increase in the risk of mortality resulting from the temperature increase. *RR* is regressed as $RR = exp^{\beta(t-OT)}$, so *AF* is calculated as:

$$AF = \frac{RR-1}{RR} = 1 - exp^{-\beta(t-OT)} \qquad (2)$$

where t is the daily maximum temperature, β is the exposure-response factor and OT is optimum temperature, and both parameters were adopted from Honda et al. (2014).[82] The method was applied to gridded daily temperature data from ECMWF ERA5 dataset, and gridded population data from NASA GPWv4 population dataset and ISIMIP Histsoc records, as with Indicator 1.1.1.[83] As the indicator focuses on a population that is 65 years old or older, age-structure data from United Nation World Population Prospects was also used. Because the mortality rate data of 2020–2022 has not yet been released, and the real data were highly affected by Covid-19 affecting the accuracy of the results, 2019 data were used instead.

The heat-related mortality was first calculated at grid level at 0.5° spatial resolution. Then it was accumulated to global level to produce a time-series analysis.

The calculation of the counterfactual scenario contains two aspects. On one of the counterfactual scenario, which kept the population and all-cause mortality rate (y_0) unchanged at the baseline period level (1990–1999), the average population and y_0 during 1990–1999 and annual temperature data were substituted into equation (1) to get the annual heat-related mortality with only climate effect; on the other counterfactual scenario, which kept the climate and y_0 unchanged at the baseline period level, which means the AF and y_0 data brought into equation (1) used the average AF and y_0 during 1990–1999, while the population data used the annual data. This allows the change in heat-related mortality for climate change only or for population change only to be calculated, respectively.

Data

- Climate data from the European Centre for Medium-Range Weather Forecasts (ECMWF) ERA5 reanalysis.[84]
- Population data from the NASA Socioeconomic Data and Applications Center (SEDAC) Gridded Population of the World (GPWv4) and The Inter-Sectoral Impact Model Intercomparison Project (ISIMIP) Histsoc dataset.[79,85]
- Demographic data from the United Nation World Population Prospects (UNWPP).[86]
- Mortality rate data are from the Global Burden of Disease.[81]
-

Caveats

This indicator applies a unique exposure-response function across all locations and times. While its use has been demonstrated in different geographies, it does not capture local differences in the health impacts from heat exposure, which can be significant. Also, this analysis assumes exposure-response function is constant. It does not capture changes in response to heat exposure that might happen over time, as a result of acclimation and adaptation. Not capturing these changes could result in an over-estimation of heat-related deaths in later calendar years. Annual average mortality rates are used, rather than daily mortality rates (y_0). Given baseline mortality can be higher in colder months, this may lead to an overestimation of overall mortalities. Nonetheless, the trends of change in mortality due to heat exposure should still be conserved.

Only the heat-related mortality of the 65-and-older population was calculated this time, but more work needs to be done to include working group people.

Additional analysis

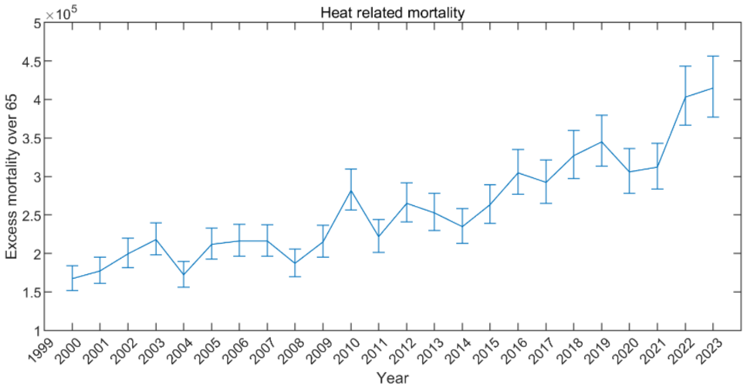

Figure 51: Annual heat-related mortality for people over 65.

Table 9: Heat-related mortality for people over 65 in different WHO regions.

WHO Region	2001-2010 average	2021	2022	2023	Growth rate
Americas	23,963	40,722	47,159	48,357	102%
Eastern Mediterranean	9,633	18,178	19,152	19,532	103%
Western Pacific	56,007	96,272	134,279	139,638	149%
Europe	79,433	107,319	132,277	135,126	70%
Africa	8,655	16,643	16,417	16,627	92%
South-East Asia	31,817	32,872	53,828	55,482	74%
Global	209,508	312,008	403,114	414,762	98%

Table 10: Heat-related mortality for people over 65 in different HDI.

Level of HDI	2001-2010 average	2021	2022	2023	Growth rate
High	67,794	120,189	149,751	155,342	129%
Low	9,993	16,751	18,834	19,025	90%
Medium	33,511	36,298	58,790	60,479	80%
Very High	97,394	137,234	174,330	178,461	83%

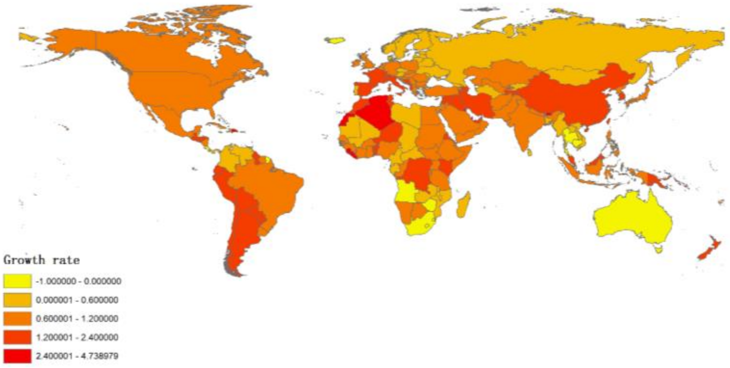

Figure 52: The growth rate of heat-related mortality for people over 65 from 2001–2010 average to 2023.

1.2: Health and extreme weather-related events

Indicator 1.2.1: wildfires

1.2.1.1 Exposure to wildfires and to wildfire risk days

Indicator authors

Prof Yun Hang, Prof Yang Liu, Dr Qiao Zhu

Methods

Based on the 2023 *Lancet* Countdown series report, this indicator has been improved in the following aspects: (1) for the calculation of fire danger risk, the latest version 4.1 updated by the Copernicus Emergency Management Service was adopted, and the calculation year was updated to 2023, (2) extended the satellite-based wildfire population exposure estimate with cloud corrections, (3) applied a precise filtration of non-fire hot spots reported by MODIS.

Wildfire:

The Fire Danger Risk Indicator, sourced from the ECMWF ERA5 atmospheric reanalysis and provided by the latest version 4.1 update from the Copernicus Emergency Management Service, involves downloading the Fire Weather Index (FWI) parameters. In the updated version, these parameters are transformed into six categories of fire danger index (FDI), according to the danger class levels definition defined by EFFIS. These categories correspond to FWI ranges as follows: very low: <5.2, low: 5.2–11.2, moderate: 11.2–21.3, high: 21.3–38.0, very high: 38.0–50.0, and extreme: ≥50.0. The changes in the mean number of days exposed to very high or extremely high fire danger risk (FDI categories of very high and extreme) were collected for the most recent available period, 2019 to 2023, and compared with a baseline period from 2003 to 2007. Population density data was derived from the NASA SEDAC Gridded Population of the World version 4 (GPWv4). The adjusted population data facilitated the computation of population-weighted average days of fire risk. For assessing changes in the mean number of days subject to very high or extremely high fire danger risk, grid cells with a population density of 400 or more people per square kilometer were omitted from the analysis.

The change in population exposure to wildfire is represented as the change in the average annual number of person-days exposed to wildfire in each country. Satellite-observed active fire spots were aggregated and spatially joined with gridded global population data from the NASA SEDAC GPW v4.11 dataset on a global 0.1° × 0.1° resolution grid. Grid cells with a population density ≥400 persons/km^2 were excluded to remove urban heat sources unrelated to wildfires. Cloud cover information was incorporated into each grid cell of the satellite observed active fire data to address the issue of fire spot underestimation due to cloud obscuration. The mean annual number of person-days exposed to wildfire during the most recent five years (2019–2023) was compared with the baseline period of 2003–2007.

Data

- Fire Weather Index historical data (v4.1) produced by the Copernicus Emergency Management Service for the European Forest Fire Information System (EFFIS).[87]
- Population data from the NASA Socioeconomic Data and Applications Center (SEDAC) Gridded Population of the World (GPWv4) and from the Hybrid gridded demographic data for the world, 1950–2020 (1.0) [88]
- MODIS Fire Radiative Power (FRP) observations MOD14/MYD14 from the NASA Fire Information for Resource Management System (FIRMS).[89]
- Cloud cover data from the EarthEnv Global 1-km Cloud dataset.[90,91]

Caveats

The Fire Weather Index (FWI) indicates the potential risk of fire based on weather conditions, rather than actual fire occurrences. Real-world fire incidents may be impacted by human activities, including changes in land use and cover, widespread fire prevention efforts, and ignition caused by humans. Furthermore, the FWI overlooks the possible impact of increased CO_2 levels, which might act as a fertilizer, altering vegetation patterns and consequently, the available combustible material. It also does not consider changes in the frequency of lightning strikes, which could be influenced by shifts in climate. There are two main caveats to the calculated wildfire exposure. First, the exposure was calculated based on active fire spots obtained from MODIS, which represent raw fire information and do not differentiate between wildfire and prescribed burns. Second, the spatial resolution of the indicator is 0.1°, which may underestimate wildfire exposure and introduce bias.

Future form of the indicator

Future research could focus on integrating the impacts of human activities, the effects of CO_2 levels on vegetation, and the influences of climate change on lightning frequency into assessments of fire risk danger days. If computational power allows, achieving a higher resolution global dataset at 0.01° could enhance the wildfire exposure estimate. This improvement could be realised by integrating high-resolution wildfire fire spots and cloud mask input.

Additional analysis

Wildfire risk indicator by HDI level, WHO region and Lancet *Countdown region*
The figures and tables below display the average changes in days with extremely high and very high risk of fire from 2019 to 2023, compared to 2003–2007, for each Human Development Index (HDI) category, and across different World Health Organization (WHO) regions or *Lancet* Countdown

regions. It is evident that countries with a low HDI, particularly those in Africa, have experienced the most significant increase in the climatological risk of wildfires.

Table 11: Population-weighted mean changes in very high or extremely high fire danger days in 2019–2023 compared with 2003–2007 by HDI level. The number and percentage of countries with increased exposure by HDI level are calculated. Large urban areas with population density ≥ 400 persons/km² are excluded.

HDI level	Population-weighted mean changes	
	Mean Change in Fire Danger Days	Number (%) of Countries with Increased Fire Danger Days
**Low**	7.2	27 (84%)
**Medium**	4.8	25 (57%)
**High**	4.8	27 (55%)
**Very High**	1.7	38 (58%)

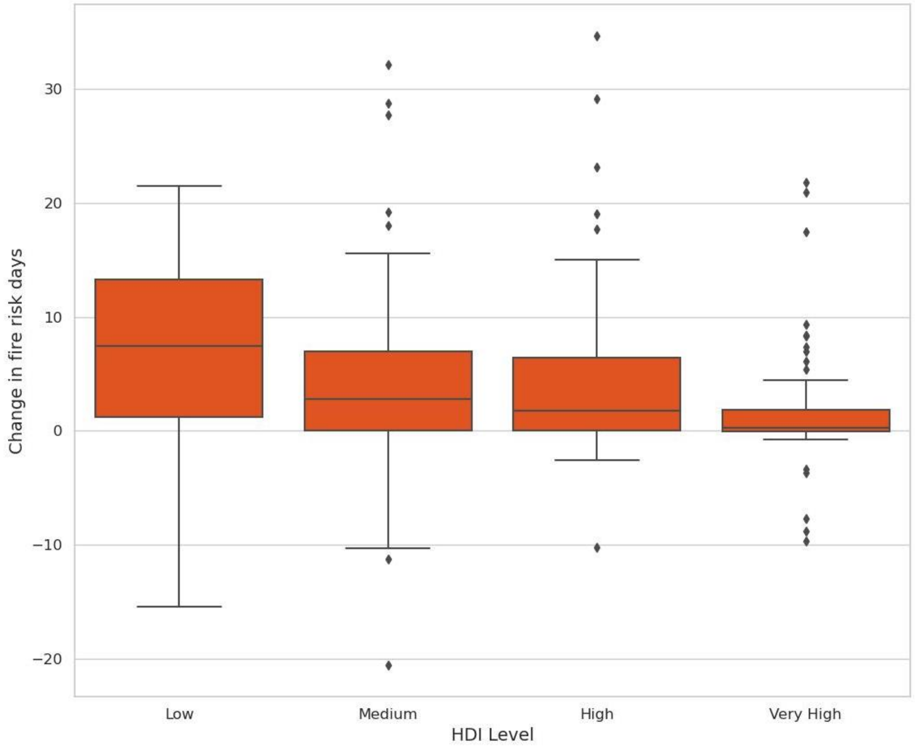

Figure 53: Population-weighted mean changes in very high or extremely high fire danger days in 2019-2023 compared with 2003-2007 by HDI level.

Table 12: Population-weighted mean changes in very high or extremely high fire danger days in 2019–2023 compared with 2003–2007 by WHO region. The number and percentage of countries with increased exposure by WHO region are calculated. Large urban areas with population density ≥ 400 persons/km² are excluded.

**WHO region**	**Population-weighted mean changes**	
	**Mean Change in Fire Danger Days**	**Number (%) of Countries with Increased Fire Danger Days**
African	7.7	36 (77%)
Americas	2.9	25 (52%)
Eastern Mediterranean	6.4	16 (73%)
European	3.6	37 (70%)
South-East Asian	-3.5	11 (36%)
Western Pacific	0	39 (15%)

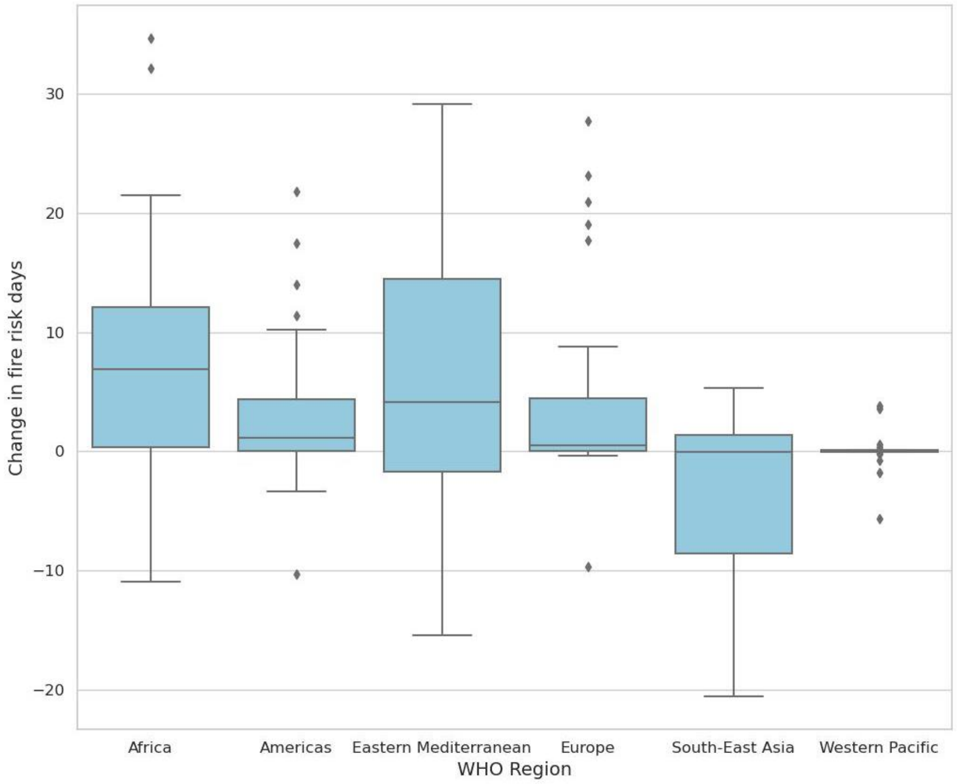

Figure 54: Population-weighted mean changes in very high or extremely high fire danger days in 2019-2023 compared with 2003-2007 by WHO region.

Table 13: Population-weighted mean changes in very high or extremely high fire danger days in 2018–2022 compared with 2001–2005 by Lancet Countdown region. The number and percentage of countries with increased exposure by WHO region are calculated. Large urban areas with population density ≥ 400 persons/km^2 are excluded.

***Lancet* Countdown region**	**Population-weighted mean changes**	
	**Mean Change in Fire Danger Days**	**Number (%) of Countries with Increased Fire Danger Days**
Africa	9.2	40 (83%)
Asia	2.8	27 (57%)
Europe	1.3	27 (63%)
Latin America	4.4	16 (89%)
Northern America	3.5	2 (100%)
Oceania	-0.3	1 (20%)
SIDS	1.6	11(19%)

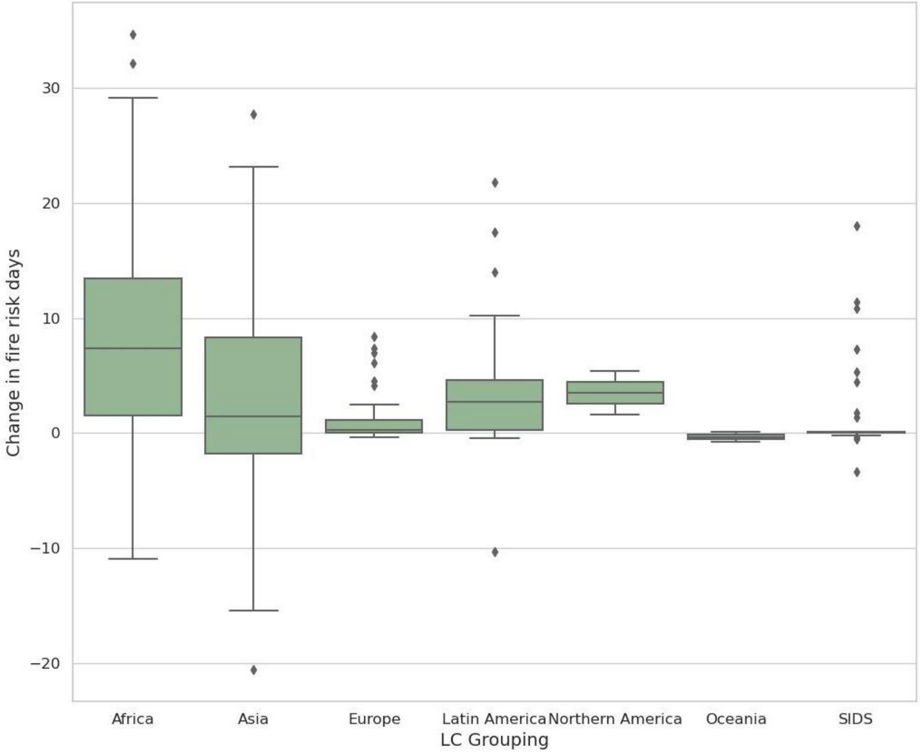

Figure 55: Population-weighted mean changes in very high or extremely high fire danger days in 2019–2023 compared with 2003–2007 by Lancet Countdown Region.

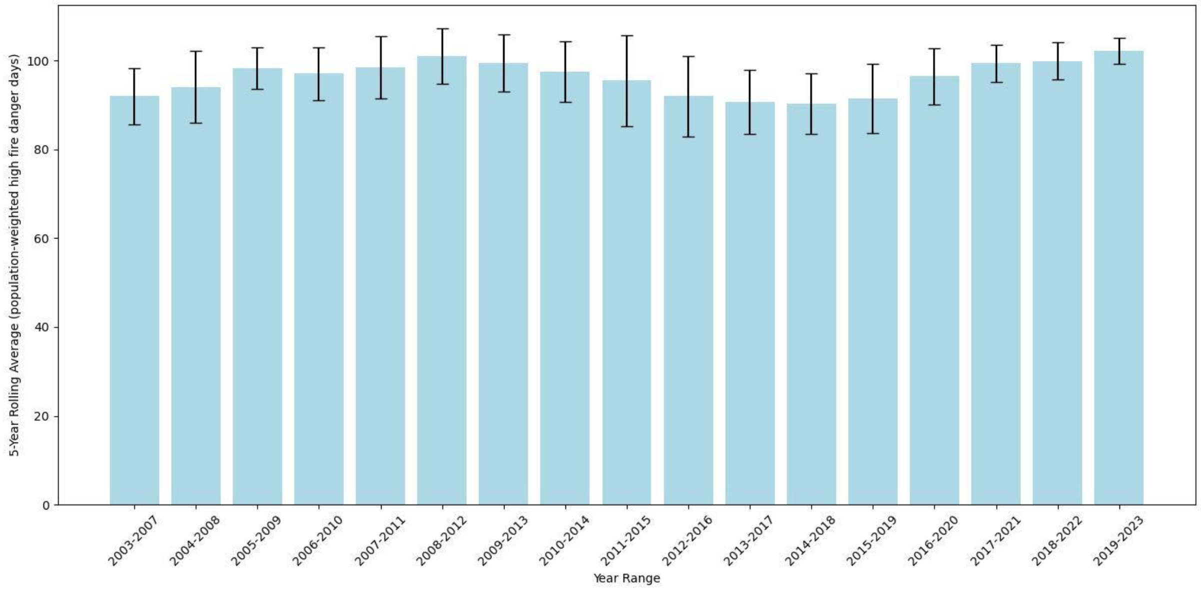

Figure 56: Five–Year Rolling Average of Population–Weighted Days with Very High or Extremely High Fire Danger (2003–2023), with Standard Deviation Represented by Black Vertical Lines.

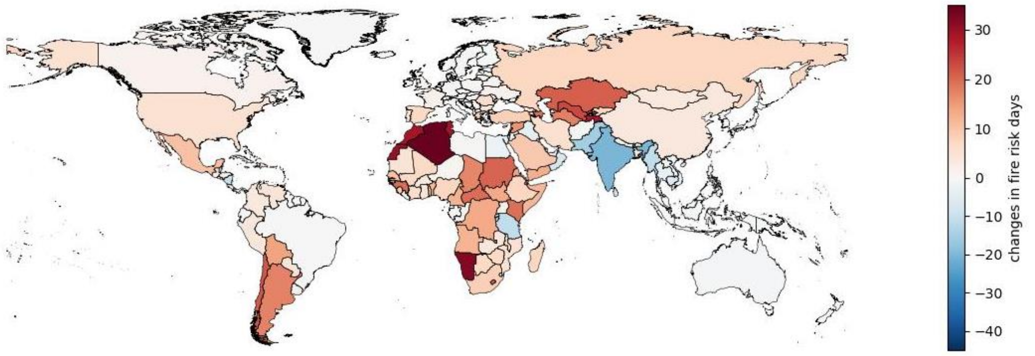

Figure 57: Population–weighted mean changes in very high and extremely high fire danger days in 2019–2023 compared with 2003–2007. Large urban areas with population density ≥ 400 persons/km2 are excluded.

Satellite-based wildfire exposure:

The changes in the annual mean number of person-days exposed to wildfire by *Lancet* Countdown (LC) region, WHO region, and HDI level in 2019–2023 compared with 2003–2007 are shown in Table 14. Concerning the LC and WHO regions, after correcting for global cloud cover, Africa appears to have experienced the largest increase in wildfire exposure (+0.3 million persons). Additionally, more than half of the Eastern Mediterranean (54.6%) and Western Pacific (51.5%) countries experienced an increase in wildfire exposure. South-East Asia (-9 million persons) and Europe (-1 million persons) appeared to have experienced a decrease in wildfire exposure. Low HDI countries seemed to have encountered the largest increase in wildfire exposure (+0.5 million persons). 62.5% of low HDI and 60.5% of medium HDI countries experienced an increase in wildfire exposure. High and very high HDI countries appeared to have experienced a decrease (-1 and -3 million persons) in wildfire exposure.

Table 14: The annual mean number of person-days exposed to wildfire by LC region (A), WHO region (B), and HDI level (C) in 2019–2023 compared with 2003–2007 (unit: 10,000 persons). The number and percentage of countries with increased exposure are calculated. Large urban areas with a population density ≥400 persons/km² are excluded.

A:

Lancet Countdown Region	Wildfire Exposure	Number (%) of Countries with Increased Exposure
Latin America	13.8	9 (50.0%)
Africa	30.4	30 (62.5%)
Asia	-12.8	18 (40.0%)
SIDS	-0.2	24 (43.6%)
Oceania	2.0	1 (33.3%)
Northern America	5.5	1 (50.0%)
Europe	-16.4	12 (28.6%)

:

WHO Region	Wildfire Exposure	Number (%) of Countries with Increased Exposure
Americas	5.3	19 (41.3%)
Africa	30.6	28 (59.6%)
South-East Asia	-89.9	4 (36.4%)
Eastern Mediterranean	21.7	12 (54.6%)
Western Pacific	0.3	17 (51.5%)
Europe	-14.1	15 (28.9%)

C:

HDI Level	Wildfire Exposure	Number (%) of Countries with Increased Exposure
High	-32.0	22 (44.9%)
Medium	31.1	26 (60.5%)
Low	51.7	20 (62.5%)
Very High	-14.1	23 (35.4%)

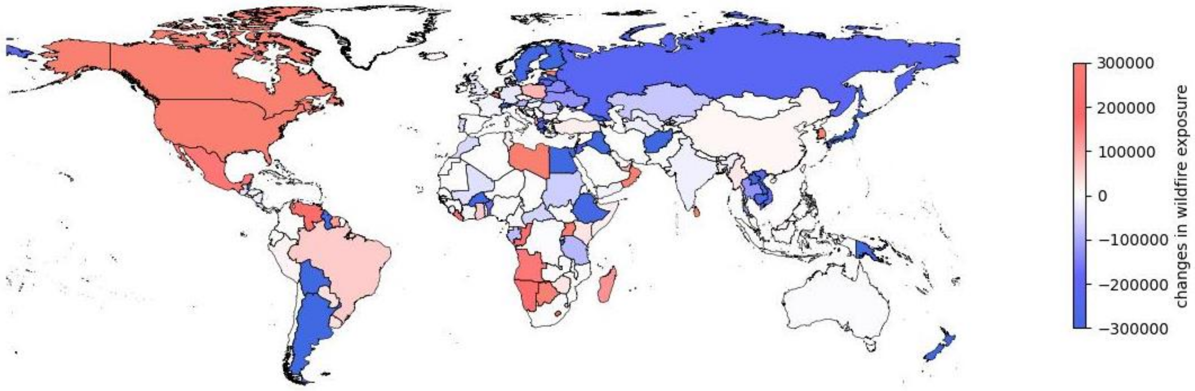

Figure 58: The annual mean number of person–days exposed to wildfire by country from 2019 to 2023 is compared with that from 2003 to 2007. Large urban areas with a population density of ≥ 400 persons/km² are excluded.

1.2.1.2 Wildfire smoke

Indicator authors

Dr Risto Hänninen, Dr Rostislav Kouznetsov, Prof Mikhail Sofiev

Methods

As in the previous 2023 report, the indicator shows the personal and population exposure to fire-originated fine particles ($PM_{2.5}$) at the global scale, now during the last 21 years, 2003–2023. This corresponds to the complete period available from MODIS instruments onboard Aqua and Terra satellites. The smoke dispersion is computed with resolution of 0.2°.[92] The atmospheric emission of fire-originated fine particles is computed by the Integrated System for vegetation fires IS4FIRES[93–96], which is interfaced to the System for Integrated modeLling of Atmospheric coMposition SILAM[92,97,98].

The input data for the SILAM computations are the active-fire retrievals of Fire Radiative Power (FRP) by MODIS instrument onboard Aqua and Terra satellites[99]. FRP serves as a proxy for estimating the amount of pollutants released by a fire to the atmosphere[100] and, combined with meteorological parameters, is used to calculate the vertical profile of the smoke injection in the air.[94,95] Subsequent transport, transformations, and removal from the atmosphere are computed by SILAM following the usual procedures of atmospheric composition modelling. The obtained hourly global concentration maps are averaged over time and space, weighted upon necessity with the population density, thus providing the set of output parameters: gridded and country-averaged time-resolving individual and population exposure to fire-originated smoke ($PM_{2.5}$).

There is a resolution increase for the 2024 fire smoke indicator in comparison with its 2023 version: 0.2° instead of 0.5° in 2023.

Data

- MODIS Fire Radiative Power (FRP) observations MOD14/MYD14 from the NASA Fire Information for Resource Management System (FIRMS)[89]
- Cloud cover data from the EarthEnv Global 1-km Cloud dataset[91,101]

- Global classification of land use from the ECOCLIMAP dataset[102]
- Population data from the NASA Socioeconomic Data and Applications Center (SEDAC) Gridded Population of the World (GPWv4) and from the Hybrid gridded demographic data for the world, 1950–2020 (1.0)[88]
- Daily surface concentration of fire-related $PM_{2.5}$ from the Finnish Meteorological Institute[103]
-

Caveats

MODIS fire radiative power and other fire products constitute the longest homogeneous global fire time series. However, low-orbit retrievals are available at each specific place only a couple of times per day. As a result, the instrument misses the fire if the scene is obscured by clouds, or the fire is too small. This omission error probability depends on region and season varying from ~20–30% in Europe in summer up to 70% in some equatorial areas. The smallest fire that can be detected by MODIS at night in clear-sky conditions and nadir view is about 4 MW, but the detection limit is close to 40 MW at the edge of the observed swath during the day. The detection limit was predicted to decrease due to lowering the orbits in 2022, but the difference was quite small (by ~10 km down to 694 km), so the overall effect is so-far negligible (~2%).

Future form of the indicator

In 2022, both Aqua and Terra satellites exited their constellation orbits: the satellites no longer have fuel for correcting the orbits and maintaining the equatorial crossing time with accuracy of 2 minutes. By October 2022, Terra had reached 15 minutes shift from its usual timing. Also, both satellites were lowered in their orbits from 705 km, by about 7 km. Since fire intensity has a strong diurnal cycle and retrievals are sensitive to the observation geometry, starting from 2023, the MODIS data will no longer constitute a homogeneous fire dataset. However, the IS4FIRES system takes fires at the exact timing of the registration, thus accounting for the change. The diurnal variation is also explicitly applied to the observed fires, also with exact timing co-location. Therefore, the 2023 FRP retrievals were still usable for IS4FIRES. But the wildfire indicator needs to be rebased to VIIRS and SLSTR instruments in near future. These new satellites have been operational for a few years and provide similar variables, albeit with different features. Merging the new instruments into the existing time series and continuing the harmonized line will be a challenge for the forthcoming reports.

Additional analysis

In contrast to the year 2022, the year 2023 was globally a year of high exposure to fire-originated PM. A particularly extreme example is Canada, where the mean concentration of fire-PM was 2.3 $\mu g/m^3$, that is 711% higher than on average in 2003–2012, but also about three times larger than the previous record 0.76 $\mu g/m^3$ in 2018. Regional analysis reveals the differences between the mean individual and population exposure in different regions (figure 59) — in all regions the year 2023 was worse than 2022. However, general conclusions, formulated in the previous report, remain. The maximum individual exposure (concentration) is typically present in Africa and Latin America, episodically also in Oceania (driven by El-Nino and related droughts). This year the concentrations in Oceania are highest when considering the *Lancet* Countdown regions, but now the Northern America region shows second highest concentrations, mainly due to large fires in Canada as discussed above. The population exposure is additionally controlled by the population density: Asia and Africa show the highest levels.

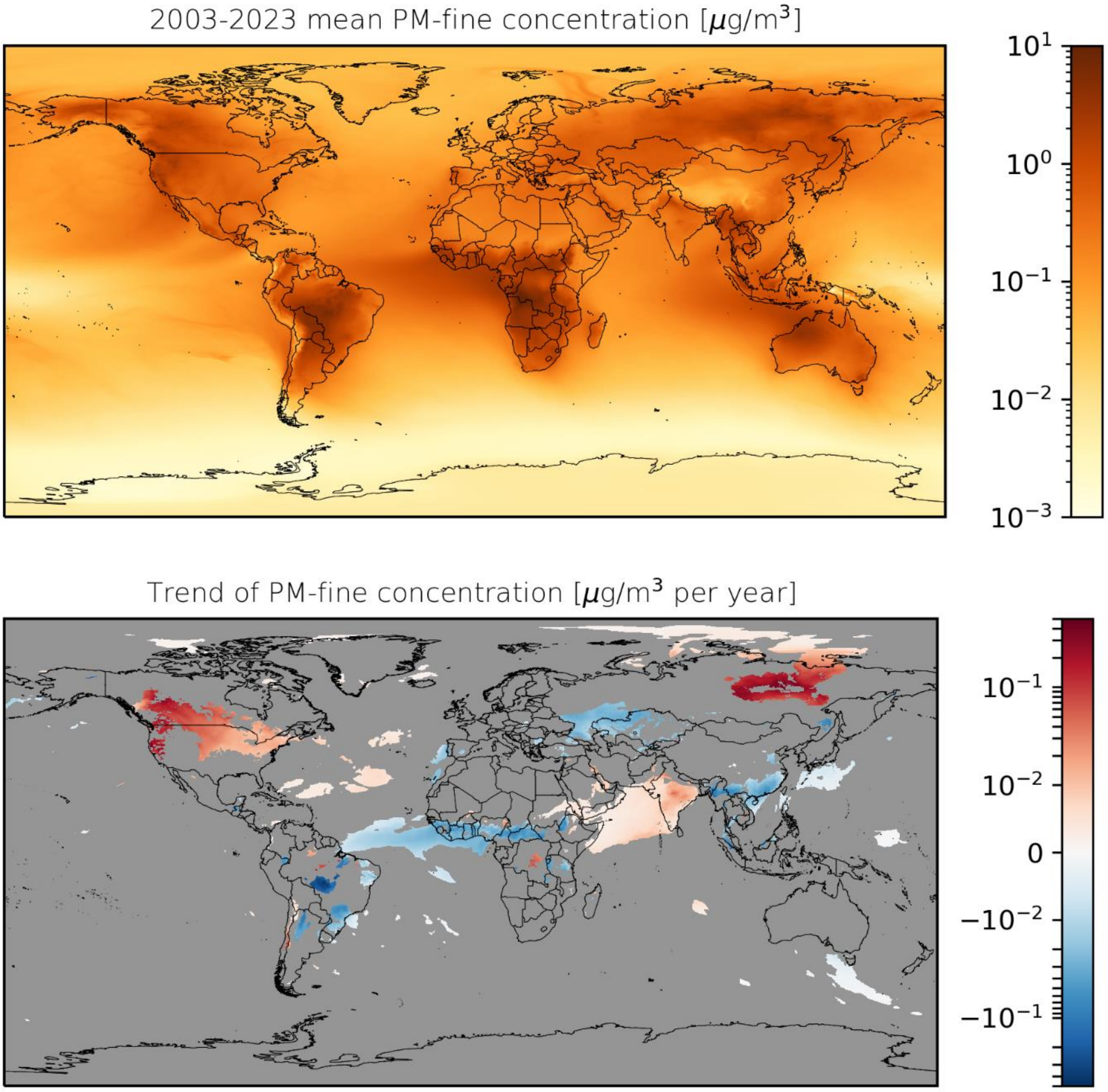

Figure 60. Gridded mean concentration of fire-induced PM (upper panel) and its trends (lower panel), 2003–2023. Only statistically significant trends ($p<0.05$) are shown.

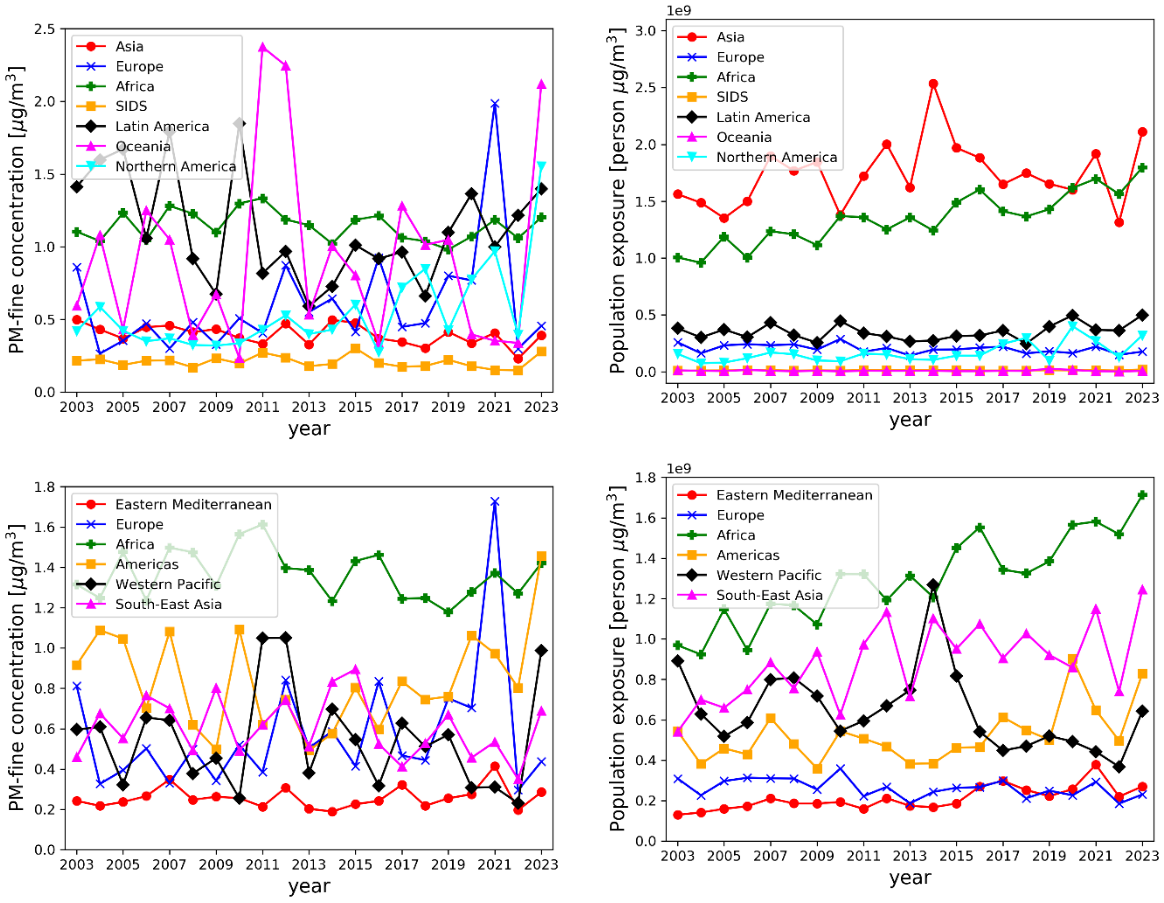

Figure 61. Mean individual (left column) and population (right column) exposure for the LC (upper row) and WHO (lower row) regions.

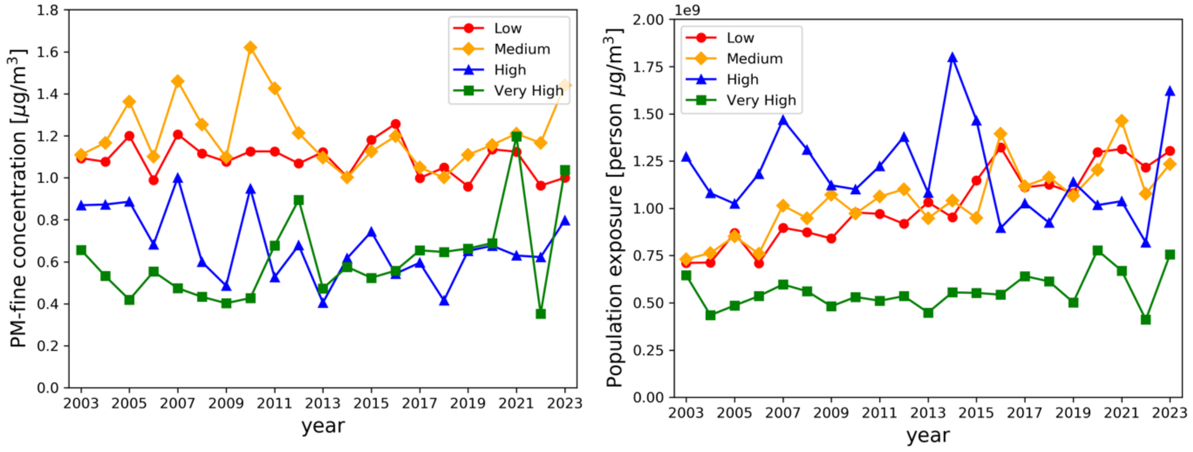

Figure 62. Area–weighted wildfire PM2.5 concentration (left panel) and population exposure (right panel) for countries with different Human Development Index.

Trends (figure 63 for gridded trends and figure 64 for country-averaged ones) computed over the 21 years with improved resolution mainly repeat the conclusions of the 2023 report. However, the large fires in 2023 in several countries cause the simple linear regression analysis to bring some of them into the positive trend, compared with the previous report. Still, the upward trend is currently significant ($p<0.05$) only for India, USA, and Canada.

Statistically significant negative trends remain in equatorial Africa, China, Kazakhstan, and Myanmar, but the negative trends, albeit not statistically significant, are visible in many countries, e.g., in South America. Without more sophisticated trend analysis procedures, the high variability of the fire occurrence, plus the rather short time series of the MODIS observations (albeit the longest homogeneous ones available), it becomes difficult to accurately and reliably determine the trends.

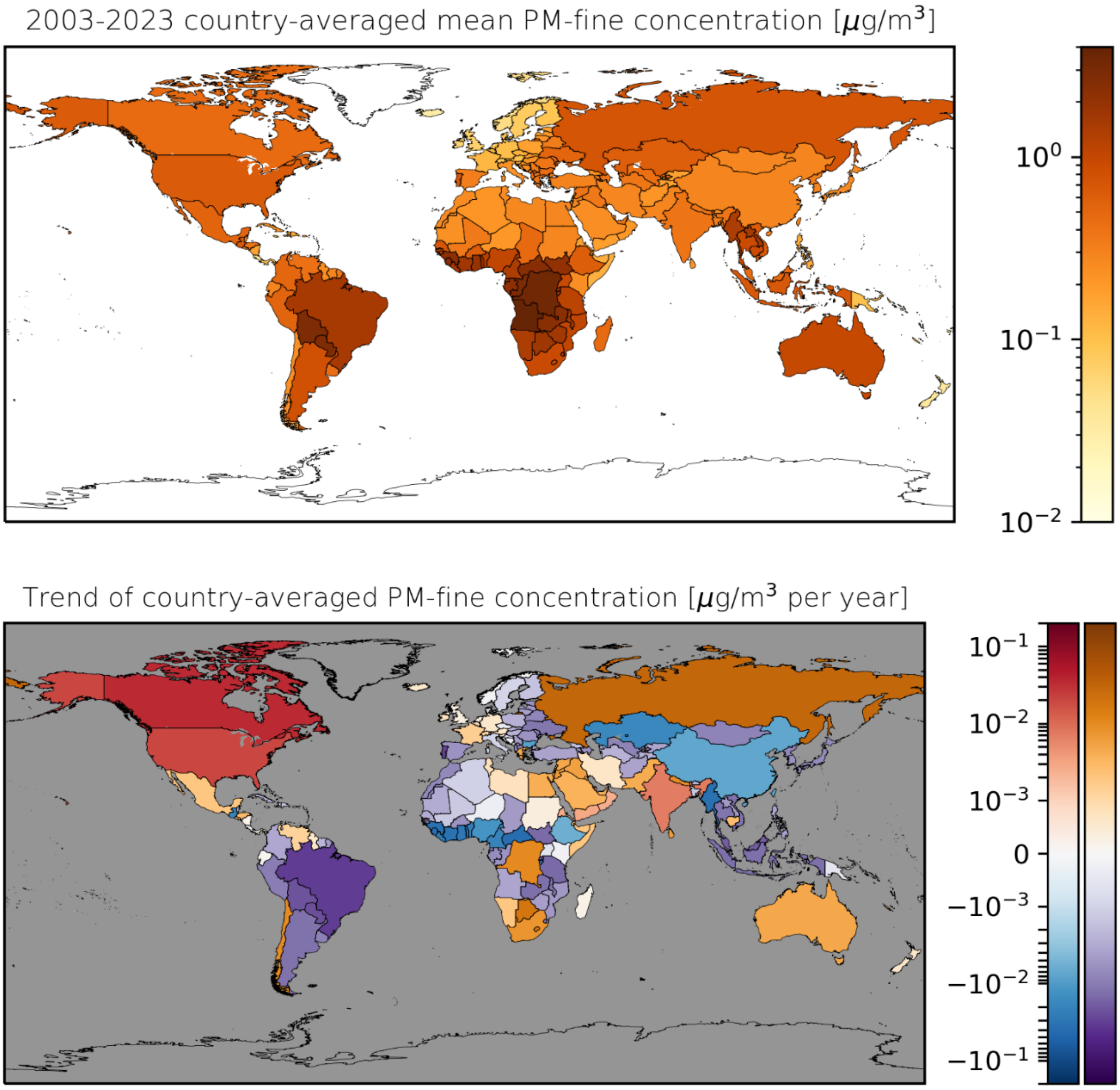

Figure 65. Country-averaged individual $PM_{2.5}$ exposure (upper panel) and its trend (lower panel). Statistically significant trends ($P<0.05$) are shown via blue-red colours, where not significant ones are shown via violet-brown colours.

Indicator 1.2.2: drought

Indicator authors

Dr Marina Romanello, Maria Walawender

Methods

The drought indicator uses the 6-monthly Standard Precipitation Evapotranspiration Index (SPEI6)[104] as a measure of the land surface affected by drought events. This index allows for both the intensity and the duration of droughts to be taken into account. It captures the influence of both altered precipitation patterns, and of potential evapotranspiration on drought severity.

SPEI6 data were obtained from the Global SPEI database. The Global SPEI database uses mean temperature data from the NOAA NCEP CPC GHCN-CAMS gridded dataset[105] and monthly precipitation data from the 'first guess' Global Precipitation Climatology Centre (GPCC).[106] GCPC data, which have an original spatial resolution of 0.5° x 0.5°, are interpolated to the resolution of 1° x 1°. Potential evapotranspiration is calculated using the FAO-56 Penman-Monteith estimation method. The Global SPEI database calculates SPEI values using constantly updated climate data at a global scale with a 1° x 1° spatial resolution and a monthly time resolution. SPEI time scales between 1 and 48 months are provided. For the indicator the 6-monthly SPEI value is used (SPEI6) and the calibration period is set to January 1950 to December 2010. SPEI6 data for 1950–present were downloaded from the Global SPEI Database.

Droughts were defined according to three severity levels using the SPEI thresholds indicated in Table 15, as defined by the Federal Office of Meteorology and Climatology MeteoSwiss.[107] In order to detect excess (unusual) drought events, "excess severe drought events" were defined as yearly counts of months in drought for each grid cell which exceed two standard deviations above the mean of the yearly counts of months in drought for the baseline period of 1986–2005. The excess events were defined for each SPEI severity level of drought independently, and the percentage of land area exposed to excess drought events at the different severity levels was calculated.

Table 15: Summary of drought severity thresholds as defined by the Federal Office of Meteorology and Climatology MeteoSwiss.

SPEI value	Description	Frequency of event in respective month
-1.3 to -1.59	severe drought	1–2 x in 20 years (i.e., 10% if the time)
-1.6 to -1.99	extreme drought	1–2 x in 40 years (i.e., 5% of the time)
< - 2	exceptional drought	1 x in 50 years or less (i.e., ≤2% of the time)

Data

- SPEI6 data from the Global SPEI Database, SPEIbase (Consejo Superior de Investigaciones Cientificas).[108]
-

Caveats

A limitation of this indicator is that it only captures the impacts of climate change on meteorological drought but does not capture the impacts of climate change on hydrological or agricultural drought,

which can have major health impacts too. Moreover, it does not measure the direct relationship between a drought and the population living in, or depending on, drought-affected areas. It is not possible to do a population-based weighting because many people affected by a drought may not live in the area affected, e.g., in the case of droughts affecting agricultural areas (which are generally sparsely populated) with impacts on the food supply. It is therefore difficult to determine the trends in persons affected by drought from the trends of severe drought areas. Further work is required to link reported drought damages in societies to climatic indicators. This would require a better understanding of the exposure factors of populations.

Future form of the indicator

Further development of the indicator will focus on using a combination of indices that capture agricultural hydrological drought, and meteorological drought, and better capture the health implication of drought events.

Additional analysis

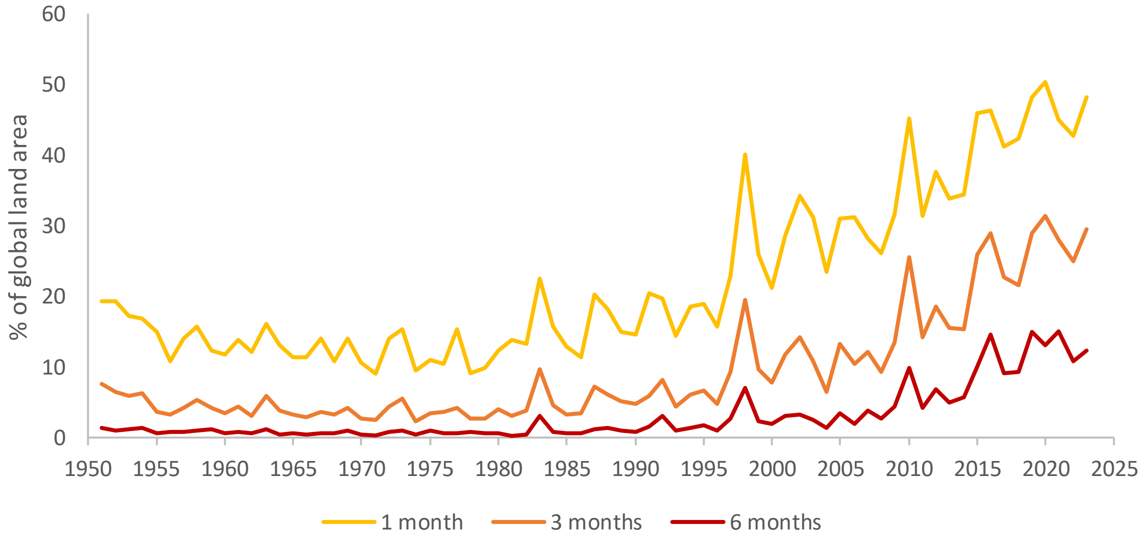

Figure 66: percentage of the global land area affected by at least 1, 3 or 6 months of extreme drought per year.

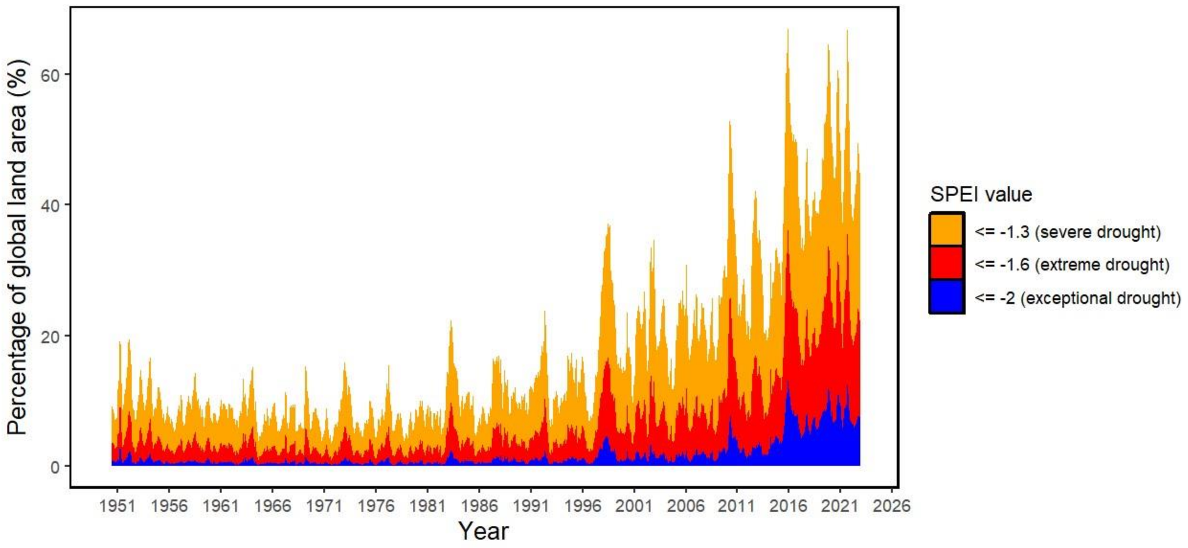

Figure 67: percentage of the global land area affected by severe, extreme, or exceptional drought, per month.

Indicator 1.2.3: extreme precipitation

Indicator authors

Dr Kelton Minor, Dr Nick Obradovich

Methods

Background:
This new indicator in the 2024 Report of the *Lancet* Countdown on Health and Climate Change monitors changes in extreme precipitation over all land globally. The indicator employs gridded, high resolution daily precipitation data from the Countdown's preferred reanalysis data source (ERA Land) and uses this data to track both the change in the average frequency of extreme precipitation events per decade over global land area in the past 30 years compared to the precipitation baseline of 1961-1990, as well as the average percentage of global land that has had an increase in the occurrence of extreme precipitation from this historical baseline. This indicator builds off of the extreme precipitation and sentiment indicator introduced in the 2022 Report of the Lancet Countdown on Health and Climate Change, while integrating longer time-series of higher resolution ERA Land data from 1961 until the most recent year.[109,110]

Intensifying extreme precipitation constitutes a primary climate-related stressor relevant for human health – one previously uncovered by the Countdown. Given the well-characterized health, economic, and ecological costs of extreme rainfall events[111,112] such as those recently seen in Pakistan, West Africa, Brazil, South Africa and Western Europe, that attribution studies found these events to be made more likely by human-caused climate change,[113] that climate change mitigation policy can limit planet-warming greenhouse gas emissions and thus plausibly help to constrain future intensification of extreme precipitation events,[114] tracking the change in land cover exposed to extreme precipitation provides a health-relevant global monitoring tool.

Data and Measures:
Gridded hourly meteorological reanalysis data from ERA Land[110,115] are employed from 1961–present. This ECMWF product provides globally consistent spatial and temporal coverage and has undergone several quality checks for precipitation.[116] ERA Land hourly precipitation data is updated on a monthly basis and is among the *Lancet* Countdown's preferred data sources. For the primary measures, these global hourly meteorological data are summed to compute gridcell daily totals (with a horizontal natural resolution of ~9km) for use in computing gridcell-specific R99p (> 99th percentile) precipitation extremes (see methodology). Since extreme precipitation can affect health outcomes at the site of occurrence as well as in adjacent and remote areas downstream, this indicator tracks changes in extreme precipitation globally over all land areas.

Computational Approach:
The indicator is calculated using a multi-step data processing pipeline. First, historical ERA Land global hourly precipitation data from 1961 through the most recent year is downloaded and structured. Second, daily sum rasters consisting of 24hr precipitation accumulations for each gridcell day is calculated. Third, it is computed whether each daily gridcell precipitation total meets the R99p extreme daily precipitation definition (daily total precipitation exceeding the grid-cell specific 99th percentile threshold for daily precipitation across the 1961–1990 baseline period). The R99p "extremely wet day" threshold is a common climatological index for extreme precipitation events and has been widely used to track global increases in extreme precipitation over land in recent decades, as well as human impacts.[117–121]

Fourth, these daily R99p event registration rasters to calculate the annual count of extreme precipitation events for each global gridcell are aggregated. Fifth, the decadal average of this metric for the recent 30-year period as well as the baseline period of 1961–1990 is computed. Sixth, the global gridcell land area-weighted average difference in R99p extreme precipitation events in the recent period from baseline is calculated, thereby capturing long-run trends in the cumulative number of extreme precipitation events experienced globally while statistically adjusting for raster gridcell areal distortions that scale with distance from the equator. Seventh, the global land area-weighted average percent change in the number of daily extreme precipitation events in the most recent 30-year period from baseline is calculated. Finally, the percentage of all global land area where the average number of extreme precipitation events increased in the recent period compared to the baseline decadal average using a 10-year rolling window average is also computed. Additionally computed is the percentage of all global land area where extreme precipitation events declined, as well as the percentage where no change was registered. Also reported is the net difference between the percent of global land area with increases versus decreases in extreme precipitation counts across these same periods (Figure 68).

Analytic Approach
The indicator provides information on extreme precipitation from 1961–present, with the topline computed as the percentage of global land area that saw an increased count of extreme daily precipitation events net of the baseline average. In plain language, this indicator can be communicated as: 'First, how much did the frequency of extreme precipitation events per decade change over land in the most recent 30-year period from the historical baseline?' 'Second, what percentage of all land globally saw an increase in extreme precipitation events from baseline?"

Data

- Gridded hourly meteorological reanalysis data from ERA Land from 1961–present.[110,115]
-

Caveats

There are several caveats associated with our indicator and other indicators that rely on ERA5 precipitation data, elaborated below.

First, the ERA5 precipitation reanalysis products — including ERA Land — do not directly assimilate any rain-gauge data, relying instead on ECMWF's Integrated Forecasting System (IFS) Cy41r2[115] to assimilate observations from short-range forecasts. Recent systematic comparisons contrasting contemporaneous rain gauge observational records and ERA5 reanalysis precipitation measurements have assessed the accuracy and biases of ERA5's performance capturing precipitation amounts, spatiotemporal precip trends, and extreme precipitation.[116,122–124] A brief review of this literature focussed on a study that specifically evaluated ERA5's ability to capture *daily extreme precipitation* events globally. This assessment found that ERA5 tended to slightly underestimate historical RX1 extreme precipitation values (max annual daily precipitation values were higher when measured with rain gauges), had more accurate performance in the extra-tropical regions than in the tropics, but accurately captured spatiotemporal trends.[116] The article concludes: "ERA5 cannot model the highest observed precipitation totals but that it can generally capture their locations and patterns" (Laver's et al., 2022). Given that this indicator tracks changes in extreme precip using high resolution ERA Land data over the entire period of observation, it is assumed that any systematic biases in ERA Land's ability to represent extreme precipitation (plausibly underestimating heavy precipitation amounts) are

consistent across the period of comparison, and thus only downward-adjust the gridcell-specific 99th percentile extreme precipitation threshold levels, rather than the registration of local extreme precipitation events in time and space, or changes in the % of global land cover affected by these extreme precipitation events. Given that this indicator tracks the occurrence of global daily precipitation extremes and changes in relative frequency, ERA5 is deemed to be sufficient for the task, pending identification of a superior alternative source with comparable spatiotemporal resolution and historical coverage.

Future form of the indicator

Future versions of this indicator can also track changes in alternative extreme precipitation metrics, including measures of more extreme R99.9p precipitation events. It is likely that ECMWF's ongoing development of ERA6 will seek to address many of the identified biases of ERA5 precipitation outlined above. To address these biases in the immediate future, the aim will be to conduct sensitivity analyses using alternative high resolution global precipitation datasets as they become available for the same historical span.

Additional analyses

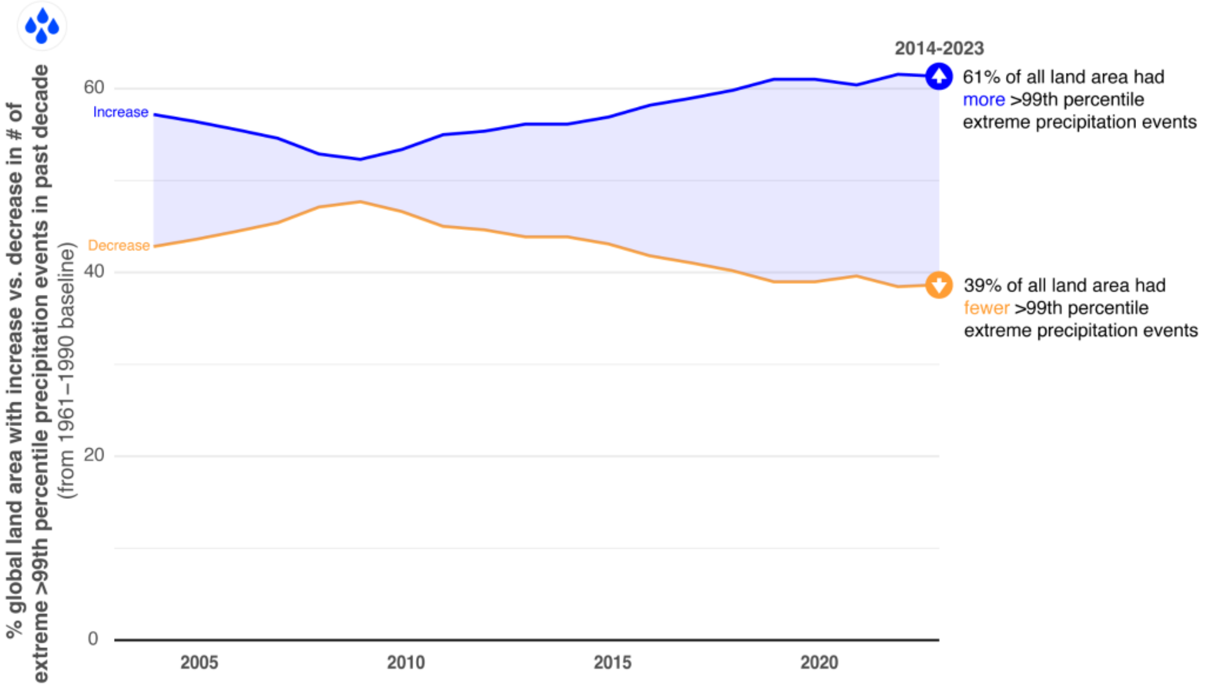

Figure 68: The net difference between the percent of global land area with increases versus decreases in extreme precipitation counts across these same periods.

During the last decade (2014-2023), extreme precipitation events increased over a majority (61.3%) of land areas, and decreased over 38.7% of global land, with a net difference of +22.6% more global land area exposed to increased extreme precipitation. Extreme precipitation events have increased on average globally, with more land area seeing increases in extreme precipitation occurrences than decreases. The percentage of global land exposed to increased extreme precipitation was greatest between 2013–2022 and remains elevated. Over the last 30 years, the percentage of land cover with

increases in extreme precipitation during the prior decade above the 1961–1990 baseline average has consistently exceeded the percentage of land cover with decreases in extreme precipitation.

Indicator 1.2.4: sand and dust storms

Indicator Authors

Dr Sara Basart, Dr Daniel Tong, Dr Andreas Uppstu, Dr Peng Xian

Methods

Global atmospheric reanalysis is being used in global air pollution health assessment studies because gaps in monitoring information exist in some regions of the world. Population exposure to mineral dust surface concentration used here (i.e., PM_{10}-dust) is estimated from an ensemble product produced from four state-of-the-art global aerosol reanalysis datasets. All data are processed to a standard unit (i.e., $\mu g/m^3$) and grid (horizontal spatial resolution of 0.1º x 0.1º) to produce a multi-model ensemble PM_{10}-dust product. The global dust product is the median of the four members, and it is available at daily, monthly, and annual intervals. Gridded PM_{10} dust is intersected with gridded population data. Population-weighted country-level exposures is then derived by averaging concentrations of all grids of the country, with weights proportional to the population.

Data

Source of the data:
The sand and dust storm (SDS) indicator is calculated based on four global aerosol/dust reanalysis datasets. Listed below are the data sources used to generate the dataset:

Global aerosol reanalysis:

- CAMS-RA (Copernicus Atmosphere Monitoring Service Reanalysis)
- NAAPS-RA (Naval Research Laboratory Navy Aerosol Analysis and Prediction System Reanalysis)
- NASA MERRA-2 (NASA Modern-Era Retrospective analysis for Research and Applications, Version 2);
- SILAM (Finnish Meteorological Institute System for Integrated modeLling of Atmospheric composition)

Demographic data are also used to calculate population exposure to dust

- Gridded Population of the World Version 4, 2021. Socioeconomic Data and Applications Center, National Aeronautics and Space Administration.

The datasets cover the period of 2003–2022 at global scale.

Caveats

Each reanalysis dataset bears their inherent caveats. Model prediction is limited by the physical mechanisms and quality of input parameters, satellite observations assimilated are limited by temporal coverage (once or twice a day) and cloud cover, and ground observations by spatial coverage. Several

efforts are being made to overcome these limitations. The atmospheric composition numerical modelling community is continuously working on improvements on the sand and dust storms characterization. These include more refined source mapping and the representation of local-scale and/or convective sand and dust storms (SDSs).

Apart from that, the ensemble method was designed to alleviate some of these caveats. Intercomparisons of ground, satellite with the individual model datasets and datasets prove this approach works effectively, outperforming all individual models. Also, the model evaluation shows have been performed to understand each dataset's strengths and limitations.

Future form of the indicator

The dust indicator can be enhanced in several ways, including adopting more WHO guidelines, such as the first interim target thresholds to assess the impacts of SDSs on ecosystem health. The global dust data also offers opportunities to associate mineral dust exposure to various health endpoints.

Additional analysis

Africa and Asia saw the highest number of days on which the population is exposed to high levels of PM_{10}-dust (i.e., 45 μg/m^3) ranging from 68 to 114 days per year (figure 69). The SIDS experienced the third highest levels of PM_{10}-dust exposure, while the population-weighted exposure in other regions is significantly lower, although high PM_{10}-dust days also existed for areas within these regions. Global PM_{10}-dust exposure shows an increasing trend from 2003 to 2022.

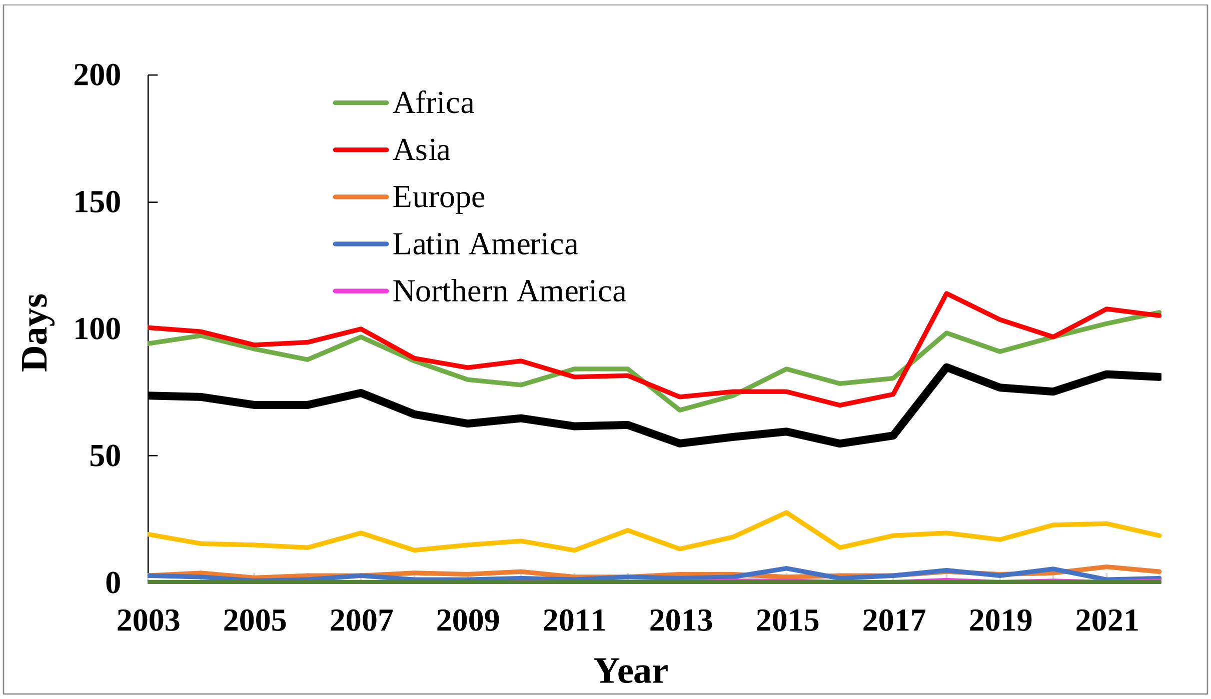

Figure 70: Population-weighted days of exposure to PM_{10}-dust above 45 μg/m^3 for 2003–2022.

Indicator 1.2.5: extreme weather and sentiment

Indicator Authors

Dr Kelton Minor, Dr Nick Obradovich

Methods

Many of the following methods, data, figures, caveats, and future form sections have been described previously in previous *Lancet* Countdown reports.[57,125]

This version of the indicator tracks the effect of heatwaves, as per indicator 1.1.2, on the sentiments of billions of geolocated expressions across millions of global Twitter users (Figure 72). The geo-tagged tweets constitute approximately two percent of all tweets and thus may be somewhat limited in their generalisability due to opt-in geo-localisation. In this year's report, this indicator has adopted an altered methodological approach driven by the fact that X — formerly Twitter — has ceased the provisioning of the publicly available stream of geo-located posts it provided through to 2022.

The empirical model employed by this indicator followed the methodological approach employed in multiple peer-reviewed publications.[54,56,57,126–128] Climate econometric methods were employed[67,129] to estimate the historical causal relationship between observationally measured sentiment expressions and exposure to varying ambient heat extremes.
As in previous years, Activity was highest in more populous and wealthier countries (figure 71).

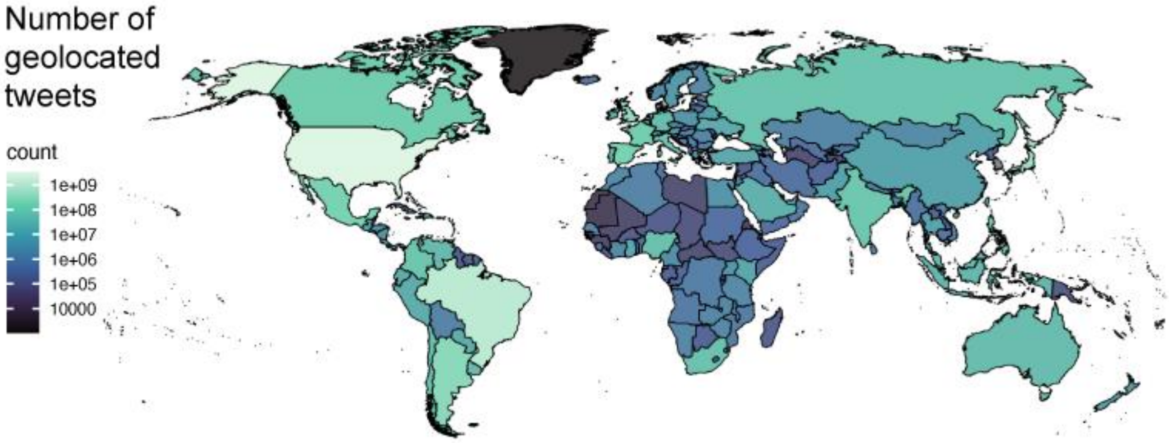

Figure 72 Country-level count of geolocated tweets, 2015-2022. Data includes posts from over 190 countries and ~44000 administrative-2 divisions (ex. counties).

The positive and negative valence[130] of each post was classified using the Linguistic Inquiry Word Count (LIWC) sentiment classification tool[131,132] across thirteen available languages: Dutch, English, French, German, Italian, Japanese, Mandarin, Portuguese, Romanian, Russian, Serbian, Spanish, and Ukrainian which provided broad geographic coverage for the sample. Table 16 presents the by-language breakdown in the distribution of tweets from the 2023 version of this indicator. Tweets with a 'lang' field matching each respective language are classified using that language's dictionary.

Table 16: By-language breakdown in the distribution of collected tweets.

Included language and LIWC dictionary	% of total geolocated tweets in data 2015–2022
Dutch	0.51%
English	65.25%
French	1.94%
German	0.51%
Italian	0.84%
Japanese	5.94%
Mandarin	0.17%
Portuguese	12.11%
Romanian	0.07%
Russian	1.15%
Serbian	0.02%
Spanish	11.44%
Ukrainian	0.06%

LIWC is one of the most highly validated psychometric sentiment classification tools and has been employed in multiple studies on the relationship between climatic variables and online emotional expressions. [131,133–136] Further, the effects observed via the LIWC classifier have also been observed via the use of alternative classifiers in both the U.S.[54] and Chinese[56] context.

To enable the estimation and analysis that underpins this indicator, geolocated social media posts were geo-spatiotemporally matched with daily ~30km gridded ECMWF ERA5 reanalysis ambient 2m air temperature data,[71] precipitation totals, and meteorological controls at the 2nd-administrative level (GADM version 3.6). This ECMWF product provides globally consistent spatial and temporal coverage. Daily gridded meteorological data were employed from the ECMWF ERA5 reanalysis product from 1986 to 2023. Heatwave metrics were calculated employing the methods used by indicator 1.1.2. Further, measures of r99p extreme daily precipitation (>99th percentile precipitation for a given location during the recent historical record using the same 1986–2005 climate normal used in the *Lancet* Countdown's heatwave definition[1]), cloud cover, relative humidity, diurnal temperature range and wind speed were incorporated from the ERA5 data as controls. The r99p "extremely wet day" threshold is an established climatological index for extreme precipitation events and has been widely used to track global increases in extreme precipitation over land in recent decades.[137–140] The calculation of the heatwave indicator followed the procedure outlined in: [57] To aggregate the meteorological variables, weather time-series were extracted from the gridded ERA5 raster data at the second administrative division-resolution for each day in the data.

The primary spatial unit of analysis for the statistical investigation was the second administrative division-level (ex. county-level). The temporal unit of analysis was the calendar date, resulting in second-administrative-unit-by-day-of-observation analyses.

To aggregate the sentiment measures to this unit of analysis, procedures previously described were followed.[54] Namely, for both positive and negative sentiment, each tweet was coded as either zero if the tweet contained no matching sentiment terms or one if it contained terms that match the corresponding sentiment. A tweet can express both positive and negative sentiment, only one of the two, or neither. For each day in the data, the average positive sentiment and the average negative sentiment was calculated for each unique user on that day,

multiplying by 100 to produce a percentage. Users' scores were then averaged within the same second-division administrative unit together to produce the daily administrative sentiment measures. These measures ranged between 0 and 100.

Models drawn from climate econometrics were employed to estimate the effect of exposure to heatwaves on positive and negative sentiment; modelling the dependent variables as positive and negative sentiment, respectively, the primary independent variable an indicator of whether an administrative-unit-day was experiencing a heatwave. The model additionally included an indicator variable for whether a location was experiencing an extremely (>99th percentile) wet day, and controls for other meteorological conditions. To control for potentially confounding factors that may vary over time across different locations, calendar-month-by-2nd-administrative region fixed effects were included in the models. Calendar date (ex. "2019-11-01", "2020-11-01") fixed effects for each unique date of observation was also included to account for idiosyncratic day-specific effects and global trends in internet and social media use. [53,62,67,141–143]

The multivariate fixed effects model estimated largely replicated that estimated in Baylis et al[54] and is as follows:

$$Y_{jmt} = \beta HEAT_{jmt} + \delta HPRCP_{jmt} + h(\mu) + \gamma_t + \nu_{jm} + \epsilon_{jmt}$$

Here j indexed 2nd-level administrative region units, m indexed unique calendar months, and t indexed unique calendar dates. Y_{jmt} represented dependent variables of positive and negative sentiment rates, respectively. $HEAT_{jmt}$ represented the binary heatwave measure, which equals one if the date is classified as a heatwave in location m and equals zero otherwise. $HPCP$ represented the extreme precipitation measure. β was the main coefficient of interest, the effect of a heatwave on positive and negative sentiment rates in percentage points. δ represents the effect of an extreme precipitation event on sentiment rates, $h(\mu)$ represented the meteorological controls, which included 20 percentage point percentile-bin controls for the temperature observations (with the omitted category of the 40th–60th temperature percentile bin serving as the omitted reference category for $HEAT_{jmt}$). $h(\mu)$ also included flexibly binned control variables for cloud cover percentages, relative humidity, and wind speed. Further, γ_t represented date-specific fixed effects that controlled for any idiosyncratic shocks in the data as well as factors that trended similarly over time across all locations. ν_{jm}indicated second-administrative-unit-by-calendar-month fixed effects that controlled for any location-specific seasonal and secular trends that might confound inference. ϵ_{jmt}represented the error term. Based on methodology in Baylis et al,[54] errors on administrative-unit-by-month and date were clustered and the regressions were weighted by the number of unique twitter posts in each administrative-unit-day.

The model for this year's indicator was estimated across all years in the data, 2015–2022, giving β, which represents the average effect of a heatwave day on positive and negative sentiment, respectively.

Due to the cessation of Twitter data availability after 2022, the modified indicator relies on the estimates obtained from the above statistical model, in particular on β, the estimate of the historical effect of exposure to a heatwave day on positive and negative sentiment, respectively. Using this parameter from the positive and negative sentiment statistical models, the indicator examines the prior year's estimated alteration in global sentiment due to heatwave exposure as compared to the

hindcasted sentiment alterations due to heatwave exposure from the baseline period of 1986–2005. To do so, the indicator employs ERA5 hourly raster 2m temperature data from the prior year and over the last decade (2014–2023) alongside ERA5 hourly raster 2m temperature data from each year in the baseline period (1986–2005). Daily heatwave metrics, using the approach described above, are calculated across each year in the ERA5 data, providing a measure of heatwave exposure for each grid-cell-day in the data for a particular year. This measure is then aggregated to the yearly level. A given raster cell in a particular year may record eight days of heatwave exposure while another might record 20 days and another might record zero.

Next, the parameter β is employed -- for both positive and negative sentiment -- to calculate the cell-year effect of heatwave exposure on positive and negative sentiment for the current year, each year in the prior decade, and for each year in the historical baseline. This provides an unweighted estimate at the grid-cell level of the impact of heatwave exposure on grid-cell sentiment.

However, not every grid cell has an equal number of people living in it. In order to ensure that the heatwave exposure variable measures population exposure to heatwaves, rather than simply geographic exposure, the indicator incorporates population count data from the Gridded Population of the World (GPW), version 4, from the Center for International Earth Science Information Network at Columbia University. Because the GPW data are estimated from a single source year and are advised not to be used in a time-series fashion, rasters from 2000–2020 are averaged to create a cross-sectional mean population count for each grid-cell. The raster projections and extents are harmonised between the GPW data and the ERA5 data. The grid-cell effect of heatwave exposure on positive and negative sentiment in a given cell-year is then weighted by the population proportion in that cell to create a population-weighted exposure value for each cell-year for the present year as well as each year in the historical baseline period.

The resultant grid-cell-year values are then summed for each year of interest — both the most recent year and the years in the baseline — providing an estimate of the planetary population-weighted heatwave sentiment impact for each year. The final indicator is the percentage change, relative to the baseline average, in the planetary heatwave sentiment impact for the past year for both positive and negative sentiment. The plain language interpretation of this indicator is: 'How much did extreme temperature alter global sentiment this year as compared to baseline effects?' — 95% confidence intervals were calculated in a similar fashion, employing the standard errors of the coefficient β from the statistical model in each of the above steps.

Lastly, an exploratory subgroup analysis was conducted across human development groups by stratifying the raster data according to the UN’s Human Development Index (HDI). The data were grouped into “high development” countries (operationalized as “very high” and “high” HDI countries) and “developing” country contexts (“medium” and “low” HDI countries), following the HDI-defined classifications,[144] and employed the same indicator analysis specification as above on the two subgroups — rasters cells were assigned into one of the two groups, respectively. Similar stratified subgroup analyses were conducted for each of the WHO geographic regions and *Lancet* Countdown regions.

Data

- Climate data from the European Centre for Medium-Range Weather Forecasts (ECMWF) ERA5 reanalysis.[30]
- Population count data from the Gridded Population of the World, version 4, from the Center for International Earth Science Information Network at Columbia University.
- Geolocated tweets collected via the Twitter Streaming API, 2015–2022.
-

Caveats

Countries that did not have X broadly available to the public—such as China—were underrepresented in the indicator, despite the addition of Mandarin tweets.

Geo-tagged tweets constitute approximately two percent of all tweets. However very similar effects have been consistently documented across social media platforms, including massive multi-country samples of status posts from Facebook, Chinese Weibo (Twitter-style) posts, and Twitter geo-located data.[54,56,128] There appears to be little reason to suspect that the Twitter data is substantially biased from the overall relationship between climatic variables and emotional expressions on social media. The functional relationships are nearly identical across platform and location.

Since higher income populations likely have greater access to adaptive amenities (air conditioning, etc.), the estimates produced by the identification strategy may be conservative (biased towards zero) for those disproportionately exposed to some of the hottest conditions in poorer socioeconomic contexts. However, a recent national analysis in China[5] suggests similar functional response forms across socioeconomic contexts, with very similar magnitudes observed for extreme heat-related responses, suggesting that added income may only smooth the relationship to a more moderate degree, and primarily for cold temperatures rather than warm ones.

While the prior indicators have observed remarkably stable annual estimates of the impact of weather extremes on sentiments up to this point (as evidenced by the relatively stable effects from each year in 2015–2022), it is possible that people will adapt to such extremes in the future — or be sensitised further to them. While there is no substantial evidence of this occurring yet (see the 2023 indicator), it is possible that as humanity moves further away from our historical estimates in time, how humans respond in the future may differ from the past.

Future form of the indicator

While the focus of this current version of the indicator is on sentiment responses to heatwaves, future iterations can expand to cover expressed responses to additional climate-related environmental stressors, including extreme precipitation, floods, hurricanes/cyclones/typhoons, fires, and smoke. Mirroring the approach taken with heatwaves in the current indicator, these extreme events can be registered using standard definitions, including those specified directly by the *Lancet* Countdown in future annual reports. Further, future measures should look at sentiment impacts across the full underlying distribution of temperature in addition to the hot extreme of its distribution.

Additional analysis

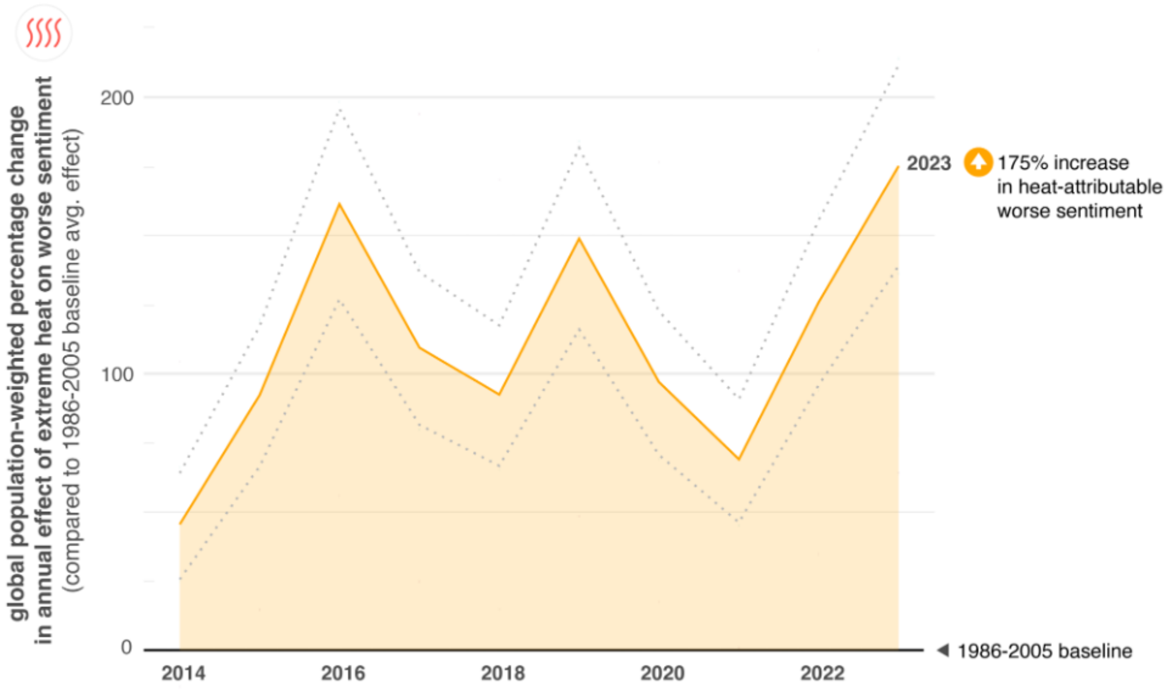

Figure 73: Global population-weighted percentage change in annual effect of extreme heat on negative sentiment, with respect to a 1986–2005 baseline

1.3: Climate suitability for infectious disease transmission

Indicator 1.3.1: dengue

Indicator Authors

Pratik Singh, Dr Henrik Sjödin, and Prof Joacim Rocklöv

Methods

The escalation in human mobility, coupled with the increasingly conducive climate conditions for arboviral disease transmission, is fostering the emergence of mosquito-borne illnesses worldwide.[145–147] Notably, Dengue fever has exhibited a concerning trend, with reported cases doubling every decade since 1990.[11] Between 2000 and 2019, WHO observed a ten-fold rise in documented cases of reported mosquito-borne diseases globally, soaring from 500,000 to 5.2 million cases. Moreover, year 2023 witnessed an unprecedented rise in Dengue cases, with reported incidents in over 80 countries/territories and five WHO regions totaling a historic high of over 5 million cases in 2023 alone.[148] This disease burden translates to over 5,000 fatalities and 1.14 million disability-adjusted life-years, underscoring its substantial impact on global health.[148] Other emerging or re-emerging mosquito borne diseases including Chikungunya,[149] Yellow fever, Zika[150] also observed similar trends and are significant burden on public health.

The transmission of these arboviruses primarily occurs through female *Aedes* mosquitoes, with *Aedes aegypti* and *Aedes albopictus* as primary vectors. These species exhibit a strong affinity for human hosts and frequently compete for similar habitats, particularly in urban and peri-urban settings. The physiological and ecological dynamics of these ectothermic organisms, are profoundly influenced by climate. Variations in climatic condition significantly impact their survival rates, feeding behaviours, developmental processes, and reproductive capacities. Consequently, understanding the interplay between climate and *Aedes* mosquito biology is crucial for predicting and mitigating the spread of above mentioned arboviruses. Building upon established methodologies outlined in previous studies,[151–161] the population dynamics of these mosquitoes are characterised by a comprehensive stage - structured mechanistic model where model parameters incorporate the effects of temperature, rainfall, and other climatic factors. The mechanistic model encapsulates three aquatic stages: Egg (E), Diapausing egg (E_{dia}), and Juvenile stage (combining Larval and Pupal stages) for *Aedes albopictus* and two aquatic stages for (Egg (E), and Juvenile stage) for *Aedes aegypti* Additionally, it incorporates three aerial stages Emerging adult (A_{em}), Blood-fed adults (A_b), and Ovipositing adults (A_o) and only one Blood-fed adults (A_b) stage for *Aedes aegypti* and *Aedes albopictus* respectively. Due to limitations in available data for parameterisation and insignificant improvements in model fitting observed with separate Larval and Pupal stages, these stages are merged into a single juvenile class. The resting and mating stage of emerging mosquitoes are also combined into the emerging adult stage. The models compute the population density of mosquitoes autonomously within each grid cell, assuming well-mixed mosquito populations within grid cells of 0.5° × 0.5° spatial resolution based on ERA - 5 land data [162,163] resampled from 0.1° × 0.1° original resolution. Gridded human population and population density data are obtained from ISIMIP3a protocol.[164] Linear interpolation or extrapolation is used to retrieve the data for population count and population density of missing years. Daily time steps are used for simulating the *Aedes albopictus* model and then the output is aggregated (population per hectare) to monthly time steps. In contrast, monthly time steps were used to solve the *Aedes aegypti* model to obtain number of mosquitoes per breeding site.

The simulated abundance *(Aedes aegypti and Aedes albopictus)* of blood-fed mosquitoes, denoted as A_b, is then utilized to estimate the Vectorial Capacity (VC) or ability of vector to transmit the virus[165] as $VC = \frac{m\, a^2(T) bc(T) \exp\left\{\frac{-\mu(T)}{PDR\,(T)}\right\}}{\mu(T)}$, where a refers to the biting rate (per mosquito) or the inverse of gonotrophic cycle duration *i.e.*, the time taken between two consequent blood meals, bc denotes the vector competence and it is the proportion of mosquito who got infectious after being infected with dengue virus (*i.e*., the product of proportion of mosquito c having virus in head and peripheral parts of vector referred as disseminated by virus and the proportion of disseminated mosquitoes having virus in saliva denoted as b)[160], μ is the adult female mosquito mortality rate, $PDR,$ the parasite (in this case, viral) development rate is the inverse of duration for the development of sporozoites (getting infectious) after the mosquito was infected, and $m = \frac{A_b}{N}$ is the approximate number of simulated mosquitoes in the vicinity of a single human.[166] VC of Dengue is computed using abundance of both *Aedes aegypti* and *Aedes albopictus* independently in overlapping region of Americas, Asia, and Australia. While for Chikungunya and Zika, only the abundace estimates of *Aedes albopictus* and *Aedes aegypti* were utilized respectively.[11]

Finally, the basic reproduction number (R_0) a key metric, which is defined as the expected number of susceptible hosts to become infected due to a single primary non-immune infected host in an entirely susceptible population is calculated from VC. R_0 is formulated as: $R_0 = \frac{VC\,.\,\beta}{r}$. Here, r represents the recovery rate of infected humans (or the duration of the infectious period), and β denotes the

probability of a susceptible host becoming infected if bitten by an infectious mosquito. The ratio $m = \frac{A_b}{N}$ is central to R_0 calculation and is estimated using the approach used in Colón-González et al.[166] Since, the model provides results in terms of number of mosquitoes per hectare (*Aedes albopictus*) and mosquitoes per breeding sites (*Aedes aegypti*), a correction factor is used to scale R_0 depending on the population of the grid cells. Finally, the scaling parameter were estimated by comparing the R_0 data available for a subset of spatiotemporal points.[167] Lastly, spatial, and temporal aggregation is performed to obtain region (WHO, HDI and country) wise annually aggregated values of R_0 using raster R package.[168]

Data

- Monthly climate data (2m air temperature and total precipitation) from the European Centre for Medium-Range Weather Forecasts (ECMWF) ERA5-Land reanalysis: global, 1950-2023, 0.1 grids.[162,163]
- Annual population data at 30arcmin from ISIMIP3a protocol [164]
-

Caveats

The proposed mechanistic model although reliable, primarily relies on climatic factors for describing disease dynamics and completely ignores the component of human mobility and social dynamics which are proven to be crucial for predicting disease transmission.[145,165] Furthermore, the indicator exclusively focus on two primary vectors *(Aedes aegypti and Aedes albopictus)* and their spatial dispersion, neglecting numerous other *Aedes* species which are proven to be important for dengue transmission. This is due to unavailability of sufficient data on interaction between climate and physiology of these species which eventually leads to absence of robust transmission suitability models. Also, the model computes $\boldsymbol{R_0}$ independently for both vectors and therefore lacks in providing a net or total estimate of transmission risk in overlapping area of abundance of these two vectors. The model predicted $\boldsymbol{R_0}$ should not be confused with actual $\boldsymbol{R_0}$ as the latter is driven by interaction between socioeconomic and climatic factor which was not captured by model prediction. Although, the predicted $\boldsymbol{R_0}$ is an indicator of potential for outbreaks.[165,169]

Future form of the indicator

Future versions of the indicator will seek to address the caveats listed above. Additionally, it is hoped to mechanistically integrate human mobility and socio dynamic interactions along with updated versions of the temperature response function of mosquito-pathogen traits in the model.

Additional analysis

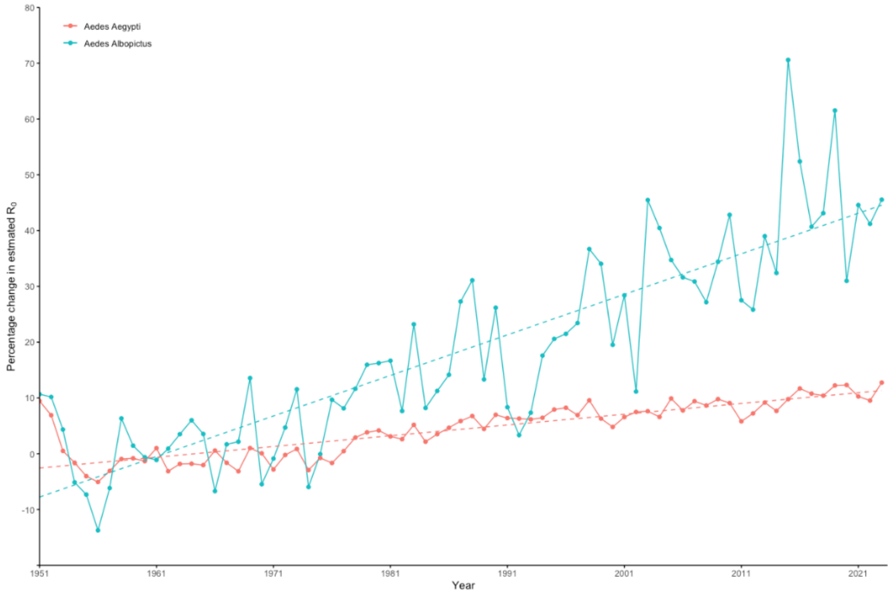

Figure 74: Percentage change in yearly average dengue estimated $\boldsymbol{R_0}$ *for Aedes Albopictus and Aedes Aegypti globally in the period 1950–2023.*

Indicator 1.3.2: malaria

Indicator Authors

Dr Alba Llabrés-Brustenga and Prof Rachel Lowe

Methods

Historical monitoring of malaria suitability (1940–2023)

The length of the transmission season, measured as the number of months suitable for malaria transmission per year from 1940 to 2023 was calculated on a grid with a resolution of 0.25° x 0.25°. Climate suitability was based on empirically derived thresholds of precipitation, temperature, and relative humidity for *P. falciparum* and *P. vivax*.

Monthly climate information between 1940 and 2023 was obtained from the ERA5 repository.[170] Relative humidity in percentage was calculated using the August-Roche-Magnus equation, which derives this value by combining dew point temperature and temperature, using the formula below.[171]

$$\boldsymbol{RH = 100 * \frac{exp\ (\frac{aT_d}{b + T_d})}{exp\ (\frac{aT}{b+T})}}$$

Where *a* and *b* are the coefficients 17.625 and 243.04, respectively, and T and T_d are temperature and dew point temperature in °C.

Elevation data were extracted from the JISAO repository, University of Washington (http://research.jisao.washington.edu/data_sets/elevation/).

Land cover classes from 2015 were extracted from the Copernicus Global Land Monitoring Service repository at 100 m resolution (https://land.copernicus.eu/pan-european/corine-land-cover) in tag image file format (.*tif*) and was assumed to be constant across the entire time series. Suitable land classes were determined according to the literature about the environmental requirements and limitations of different dominant vector species (DVS) of human malaria.[172–174] Namely, closed, and open forests, herbaceous wetlands, cultivated and managed vegetation/agriculture, and permanent water bodies, were considered as potentially suitable areas for settlement of *Anopheles* mosquito populations. In addition, urban / built up areas were also considered suitable only to analyse the increase in suitable areas for malaria transmission following a potential expansion of *Anopheles* mosquito populations to urban areas.

Suitability for a particular month was defined as the coincidence of precipitation accumulation greater than 80 mm, average temperature between 18°C and 32°C for *P. falciparum*, and 14.5°C and 33°C for *P. vivax*, and relative humidity greater than 60%.[175,176] These combined values reflected the climatic limits for potential transmission of parasites. The number of months per year with suitable conditions were then stratified by elevation using a threshold of 1500 m.a.s.l. to differentiate low from highland areas (highlands ≥1500 m.a.s.l.). Averages by country, HDI category, and WHO and *Lancet* Countdown regions were computed, weighted by the amount of suitable land cover classes. Note that in the *Lancet* Countdown region classification SIDS, highlands are only present in Papua New Guinea.

Results were visualised using time series line plots (figure 75 to figure 76), and maps and tables containing the change in the number of suitable months between the decades 1951–1960 and 2014–2023 (Table 17 to Table 19; figure 77 and figure 78). Additionally, newly suitable areas for malaria transmission in the last decade versus the baseline 1951–1960 are visualised in figure 79 and figure 80 and the increase in the length of the transmission season over the last decade considering a potential expansion of *Anopheles* mosquitoes in urban areas in figure 81 and figure 82.

Caveats

These results are based on climatic data, not malaria case data. The malaria suitability climate thresholds used are based on a consensus of the literature. In practice, the optimal and limiting conditions for transmission are dependent on the particular species of the parasite and vector.[177] Control efforts might limit the impact of these climate changes on malaria or conversely, the climate suitability may enhance or hamper control efforts.[178]

The inclusion of land suitability assumes a constant distribution of land cover classes as reported in 2015. However, dynamics in malaria transmission are highly correlated to changes in land use patterns, such as deforestation and urbanisation.[174,179] Additionally, different *Anopheles* species have adapted to different types of forests[174] and urban areas.[180] This indicator assumes a strict relationship between forest type and suitability for vector development, hence omitting disease dynamics at lower scales.

Discrepancies with previous results of the same indicator are attributable to the change in the used datasets and their spatial resolution.

Additional analysis

Historical monitoring of malaria suitability (1940–2023).

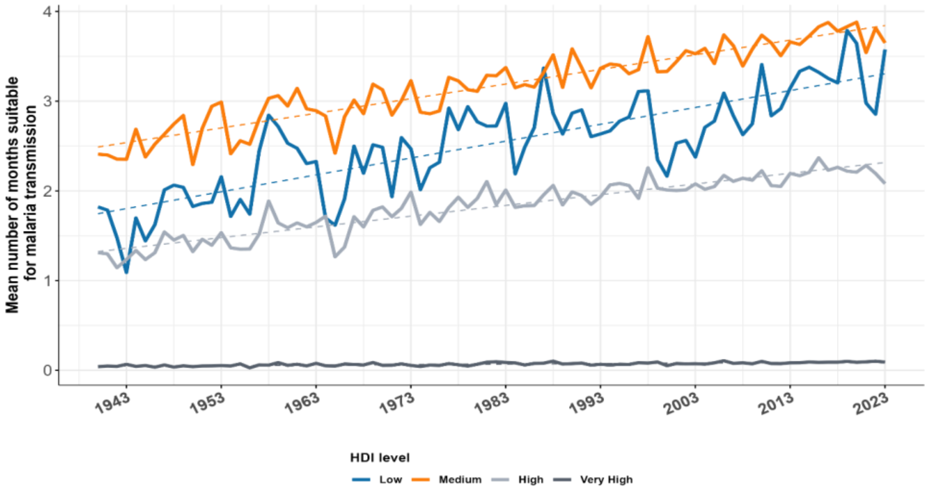

Figure 83: Mean length of malaria transmission season (months per year) for P. falciparum in highland areas (>= 1500 m.a.s.l) between 1940 to 2023, grouped by HDI categories. The length of the transmission season was calculated as the number of months per year with precipitation accumulation greater than 80 mm, average temperature between 18°C and 32°C and relative humidity greater than 60%, weighted by the amount of land cover classes suitable for Anopheles mosquitoes. Linear regression used for trend computation.

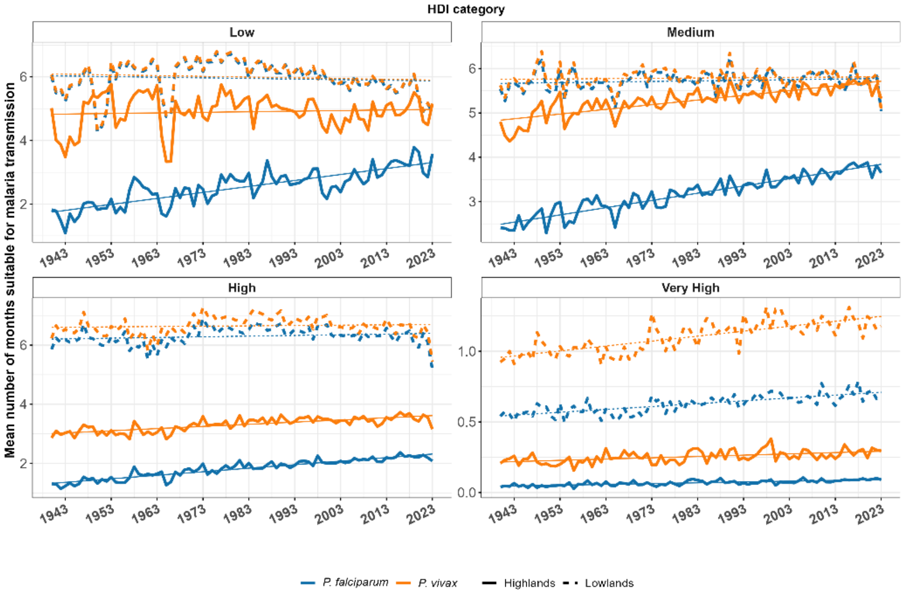

Figure 84: Mean number of months suitable for malaria transmission between 1940 and 2023, weighted by the amount of land suitability for Anopheles mosquitoes. Stratification by HDI level and elevation (highlands above or equal to 1500 masl). Linear regression was used for trend estimation.

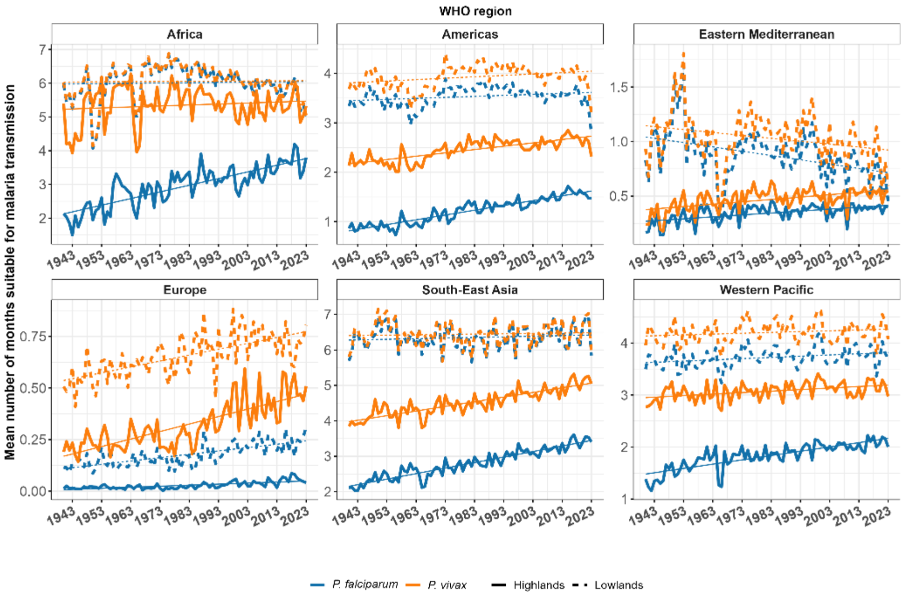

Figure 85: Mean number of months suitable for malaria transmission between 1940 and 2023, weighted by the amount of land suitability for Anopheles mosquitoes. Stratification by WHO region and elevation (highlands above or equal to 1500 masl). Linear regression was used for trend estimation.

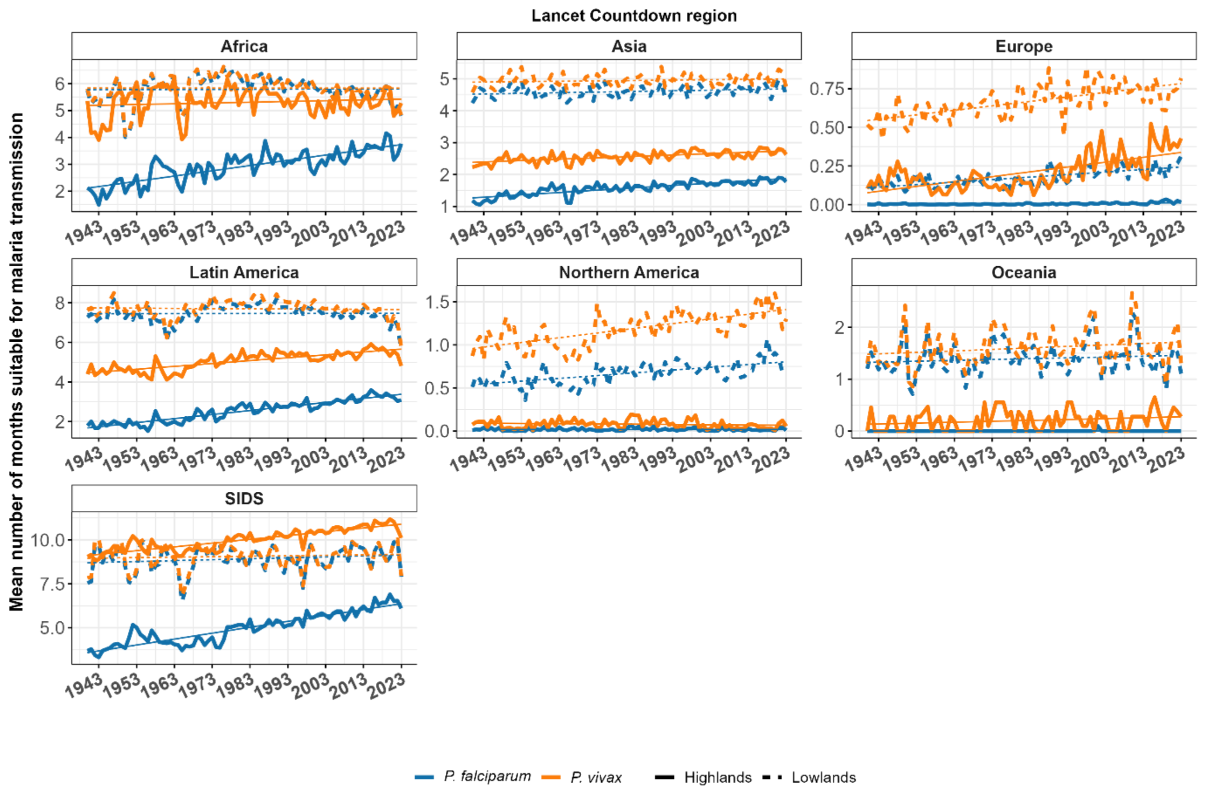

Figure 86: Mean number of months suitable for malaria transmission between 1940 and 2023, weighted by the amount of land suitability for Anopheles mosquitoes. Stratification by Lancet Countdown region and elevation (highlands above or equal to 1500 masl). Linear regression was used for trend estimation.

Table 17: Percentage change in median number of months suitable for malaria transmission between 1951–1960 and 2014–2023 stratified by Human Development Index (HDI) and altitude (highlands above or equal to 1500 masl).

**HDI level**	**P. falciparum**		**P. vivax**	
	**Highlands N months (%)**	**Lowlands N months (%)**	**Highlands N months (%)**	**Lowlands N months (%)**
Low	1.3 (63.9%)	-0.72 (-11.5%)	-0.44 (-8%)	-0.73 (-11.7%)
Medium	0.93 (32.2%)	0.13 (2.3%)	0.54 (10.6%)	0.12 (2.1%)
High	0.73 (48.7%)	0.13 (2.2%)	0.58 (19.3%)	0.08 (1.2%)
Very high	0.04 (69.2%)	0.12 (20.8%)	0.09 (42.8%)	0.19 (18.5%)

Table 18: Percentage change in median number of months suitable for malaria transmission between 1951–1960 and 2014-2023 stratified by WHO region and altitude (highlands above or equal to 1500 masl).

**WHO Regions**	**P. falciparum**		**P. vivax**	
	**Highlands N months (%)**	**Lowlands N months (%)**	**Highlands N months (%)**	**Lowlands N months (%)**
Africa	1.25 (51.1%)	-0.51 (-8.2%)	-0.5 (-8.7%)	-0.54 (-8.6%)
Americas	0.66 (72.3%)	0.14 (4.1%)	0.52 (24.1%)	0.19 (5%)
Eastern Mediterranean	0.08 (24.7%)	-0.31 (-31.2%)	0.1 (23.8%)	-0.23 (-20.2%)
Europe	0.04 (238.2%)	0.11 (88.8%)	0.27 (121.7%)	0.16 (28.4%)
South-East Asia	0.95 (38.6%)	-0.02 (-0.3%)	0.82 (19.3%)	-0.07 (-1.1%)
Western Pacific	0.43 (25.8%)	0.19 (5.3%)	0.13 (4.3%)	0.16 (3.9%)

Table 19: Percentage change in median number of months suitable for malaria transmission between 1951–1960 and 2014–2024 stratified by Lancet Countdown group and altitude (highlands above or equal to 1500 masl).

***Lancet* Countdown Regions**	**P. falciparum**		**P. vivax**	
	**Highlands N months (%)**	**Lowlands N months (%)**	**Highlands N months (%)**	**Lowlands N months (%)**
Africa	1.24 (50.9%)	-0.52 (-8.8%)	-0.50 (-8.7%)	-0.55 (-9.1%)
Asia	0.45 (32.7%)	0.16 (3.6%)	0.25 (10.0%)	0.13 (2.6%)
Europe	0.02 (1750%)	0.12 (94.1%)	0.26 (215.6%)	0.17 (29%)
Northern America	0.00 (25.0%)	0.26 (43.5%)	0.01 (40.3%)	0.37 (36.1%)
Oceania	0 (0%)	0.03 (2.5%)	0.05 (17.5%)	0.08 (5.8%)
SIDS	1.94 (43.0%)	0.57 (6.7%)	1.25 (12.9%)	0.44 (5%)
Latin America	1.40 (73.4%)	0.06 (0.9%)	1.12 (25.1%)	-0.03 (-0.4%)

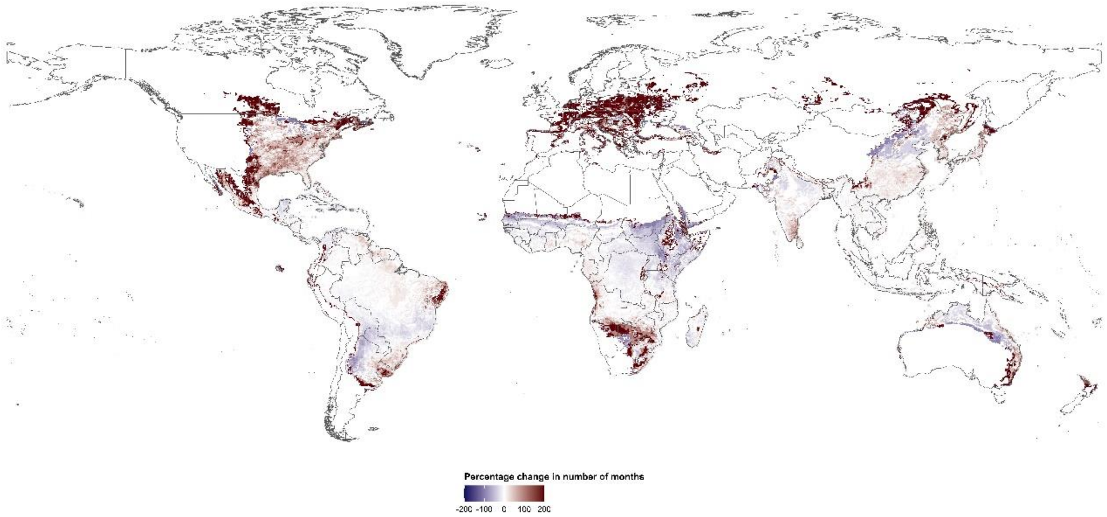

Figure 87: Change in the length of transmission season for P. falciparum from 1951–1960 to 2014–2023 in suitable land cover classes suitable for Anopheles mosquitoes.

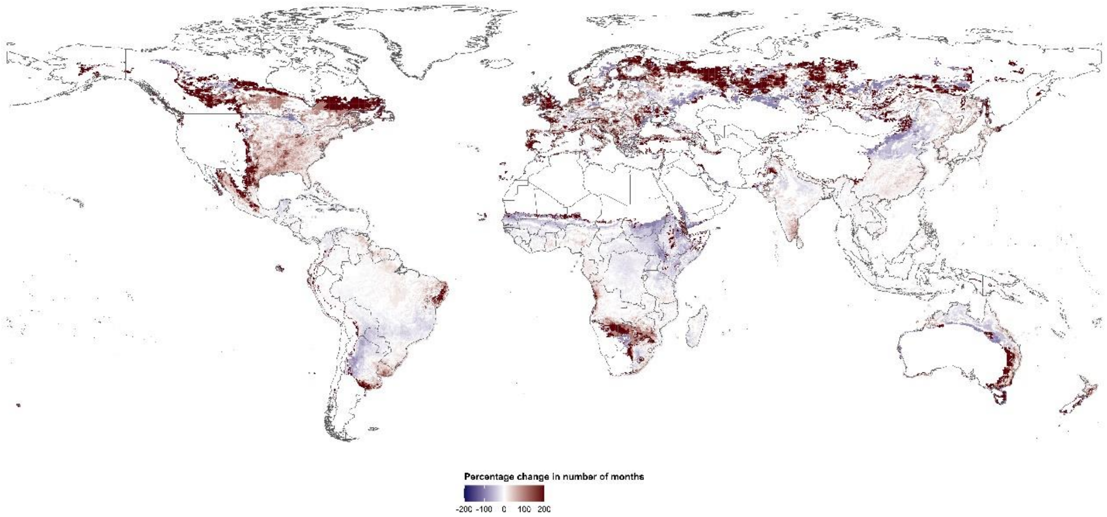

Figure 88: Change in the length of transmission season for P. vivax from 1951–1960 to 2014–2023 in suitable land cover classes suitable for Anopheles mosquitoes.

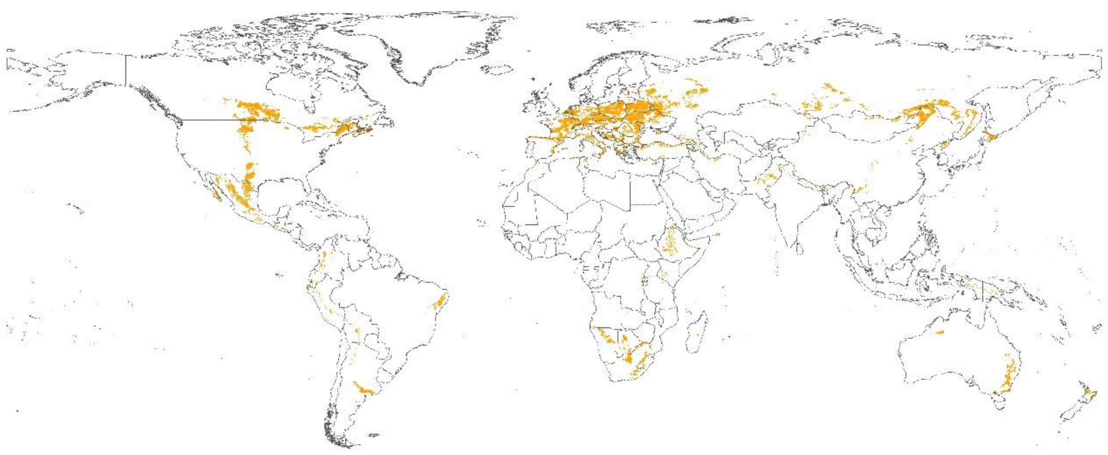

Figure 89: Newly suitable areas for transmission of P. falciparum in the period 2014–2023 (orange), compared to 1951–1960, in land cover classes suitable for Anopheles mosquitoes.

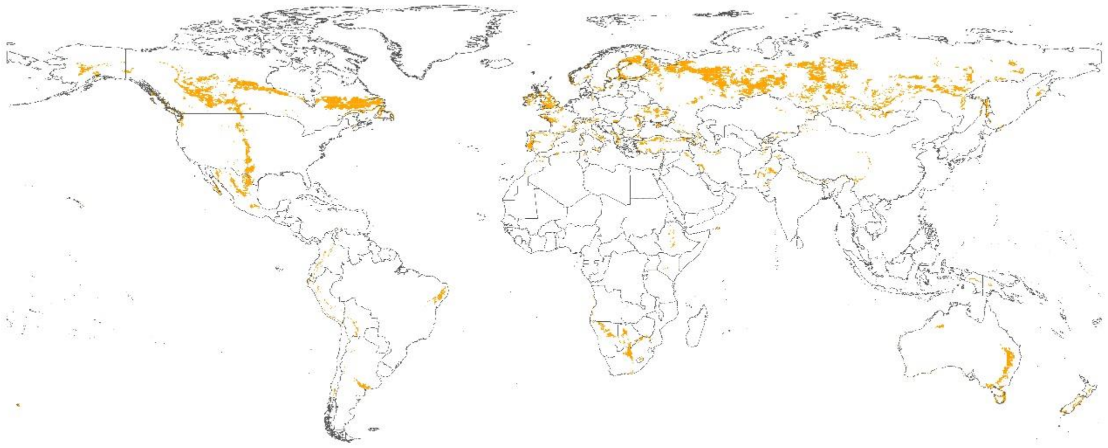

Figure 90: Newly suitable areas for transmission of P. vivax in the period 2014–2023 (orange), compared to 1951–1960, in land cover classes suitable for Anopheles mosquitoes.

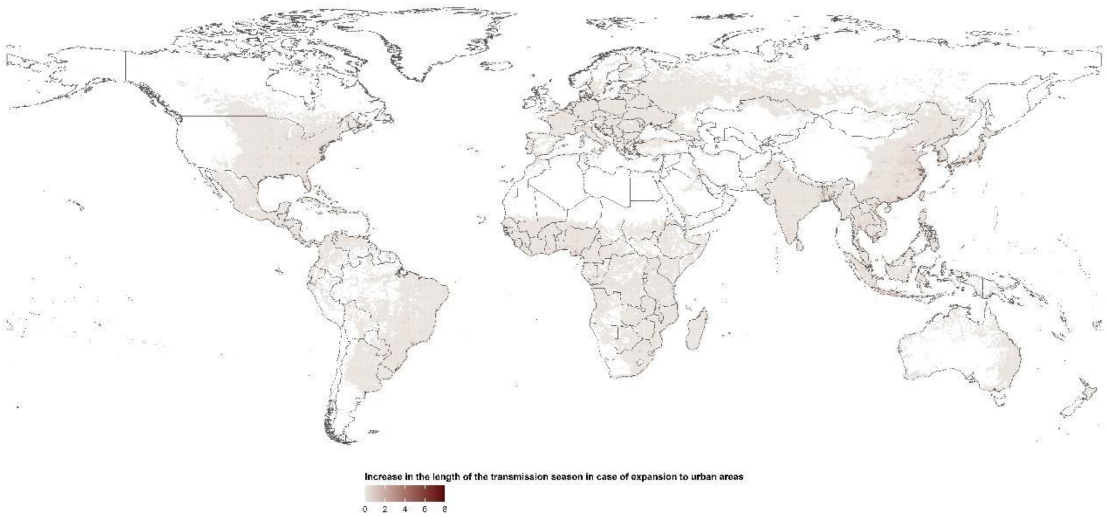

Figure 91: Increase in the length of the transmission season (number of suitable months) of P. falciparum in 2014–2023 by including urban / built up areas as suitable land cover classes for Anopheles mosquitoes.

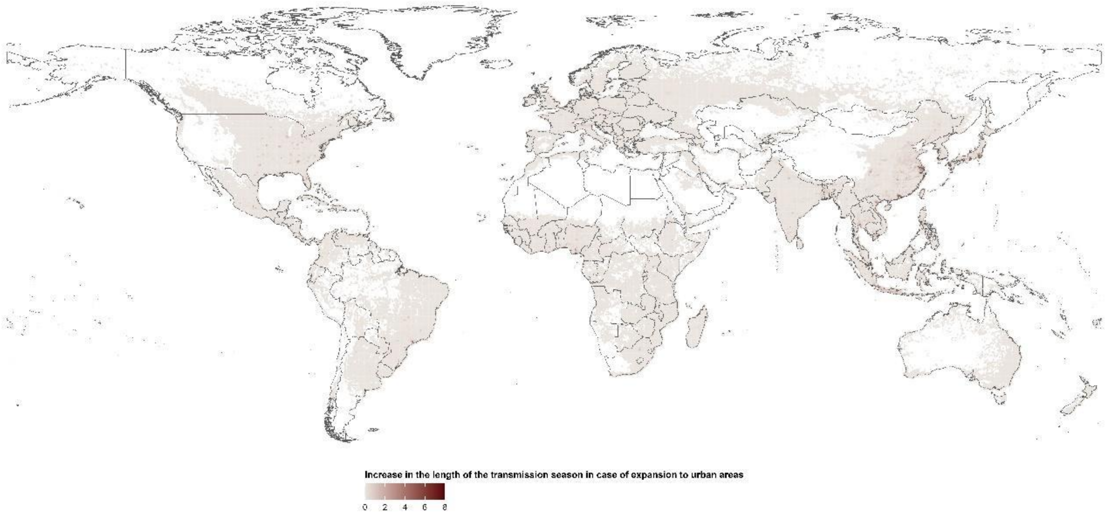

Figure 92: Increase in the length of the transmission season (number of suitable months) of P. vivax in 2014–2023 by including urban / built up areas as suitable land cover classes for Anopheles mosquitoes.

Table 20: Historical monitoring of malaria suitability (1940–2023)

Variable	Source	Frequency of update	Spatial resolution	Temporal range
Monthly 2-meter dew point temperature	ERA5	Monthly with a 3-month delay relative to present	0.25°	Jan 1940 to Dec 2023
Monthly 2-meter temperature				
Monthly total precipitation				
Altitude	JISAO	-	0.25°	-
Land cover	Copernicus Global Land Service	Annually	100 m	2015

Indicator 1.3.3: vibrio

Indicator Authors

Prof Jaime Martinez-Urtaza, Prof Jan C. Semenza, Dr Joaquin A. Trinanes

Methods

This indicator focuses on mapping environmental suitability for pathogenic *Vibrio* spp. in coastal zones globally (<10km from coast). *Vibrio* spp. are globally distributed aquatic bacteria that are ubiquitous in warm estuarine and coastal waters with low to moderate salinity. *V. parahaemolyticus*, *V. vulnificus*, and non-toxigenic *V. cholerae* (non-O1/non-O139) are pathogenic in humans. These *Vibrio* species are associated with sporadic cases of gastroenteritis, wound infections, ear infections, or septicaemia in circumscribed localities.

Vibrio ecology, abundances, distributions, and patterns of infection are often strongly mediated by environmental conditions.[181–184] On the basis of the consensus in the literature on what environments *Vibrio* infections may thrive, the indicator uses thresholds of >18°C for Sea Surface Temperature (SST) and <28 PSU for Sea Surface Salinity (SSS). The *Vibrio* suitability regions were determined based on a threshold-based approach for sea surface temperature and sea surface salinity estimates. Those areas showing temperatures above 18ºC and salinities below 28 psu were flagged as suitable for *Vibrio*. These thresholds were used considering previous studies[185,186] and match the values currently being used for the global operational *Vibrio* suitability fields. The threshold value for salinity is well below the usual ranges in most of the open ocean and takes into account the potential local decreases due to freshwater fluxes into the ocean (e.g., precipitation, runoff), making it a conservative estimate. For SST and SSS, only those cells closer than 10km to the global coastline were analysed. This band represents the areas where human exposure to *Vibrio* via direct contact with

water is maximum, and also the region where most of the aquaculture-related activities, another core source of vibriosis, take place.

In previous reports of the *Lancet* Countdown, the *Vibrio* indicator was estimated based on the two environmental factors described above — seawater temperature and salinity — missing other key elements related to exposure and transmission of *Vibrio* illness, such as socioeconomic and demographic aspects. The advent of a new generation of models, such as those participating in CMIP6 (Coupled Model Intercomparison Project 6)[187], in combination with the new Shared Socioeconomic Pathways (SSPs)[188], has provided an exceptional opportunity to introduce a wider prospect and more robust projections into the models, integrating an increasing resolution and with key socioeconomic drivers (economic growth, demography, education and technological development).

For the 2023 *Vibrio* indicator projections, CMIP6 data is used from the AWI Climate model AWI-CM-1-1. As a difference from last studies, which rely on monthly projections, these are daily projections, in order to provide a more refined analysis, better capturing the variability patterns of vibrio risk. Additionally, the Inter-Sectoral Impact Model Intercomparison Project (ISIMIP) Project 2b annual global population data was employed to compute the population at risk. The population potentially affected by exposure to Vibrio has been selected based on the ad-hoc distance of 100km between areas showing Vibrio suitability and the center of the population cell for that time period. Climate, population and socioeconomic projections were combined to generate more accurate estimates of changes in Vibrio suitability, and provide a global estimate of the population at risk of vibriosis for 2023 compared to a 1995-2014 baseline with data coverage from 1982 to present. A conservative assumption is applied of infection rate per 100,000 population of 0.3 reported for the USA (as estimated by both COVIS-CDC and FoodNet for the USA)[189,190] and took in consideration the limitations of surveillance data and underreporting in the USA, scaled up the number of infections 143 times[190] to calculate a more probable incidence of disease.

Finally, the climate, population and socioeconomic projections included into the framework of the Shared Socioeconomic Pathways (SSPs)[17] are also considered to provide accurate estimates of future changes in *Vibrio* suitability and population at risk and generate projections for a low- and high-emission scenarios (SSP1-2.6. and SSP3-7.0 respectively) by the end of the century compared to the pre-industrial period. In this study, one realisation under emission scenario SSP1-2.6. and 5 realisations under scenario SSP3-7.0 are used.

Here suitability is reported at two levels; the length in Km of coastline that experienced suitable conditions for *Vibrio* infections and the period of suitable conditions for Vibrio in days per year. These two indicators were calculated globally (for all coastal countries), and the results summarised by country.

Data

- AWI-CM-1-1 sea surface temperature (SST) and sea surface salinity (SSS) from CMIP6 (2015-2100) SSP126 and SSP370 experiments. Both variables are provided at daily time steps and 25km resolution, offering an improved coastal coverage than similar products at 0.5º resolution
- Coastline length from the World Factbook data (https://www.cia.gov/the-world-factbook/). This dataset is used to estimate the trends (km/year and per country) in the length of coastline affected by Vibrio favourable conditions

- ISIMIP2b annual global population data at 0.5° resolution for the period 2006–2100 (SSP245 & SSP585) were used to estimate the population potentially affected by Vibrio infections. Our methodology takes into account a maximum distance of 100km between the pixel showing Vibrio suitability and the center of the population cell. Our simulations for each scenario use the corresponding socio-economic population dataset
- Sea surface temperature data from the Global Ocean OSTIA Sea Surface Temperature and Sea Ice Reprocessed dataset between 1982–2023[191]
- Sea surface salinity data from the Mercator Ocean Reanalysis[192]

Caveats

The results are derived on the basis of suitable SST and SSS conditions only, and do not include other potentially important drivers (e.g., globalisation), environmental predictors of pathogenic *Vibrio* infections (e.g., cholorphyll-*a*, turbidity) or disease case data. Nevertheless, these associations have been explored and are reported in the supporting references included above.

In the global analysis, the slope of the trendlines over the time series is mostly flat for the tropical/subtropical region and the southern Hemisphere. However, the SST-only suitability shows a strong upward trend in the southern hemisphere, indicating that on average temperature conditions are also improving growth conditions for *Vibrio* in these areas, while SSS is generally limiting. However, locally suitable SSS conditions will also occur in these regions based on, for example, variation in local rainfall and river runoff, which can make these regions sporadically suitable for *Vibrio* infections.

Additional analysis

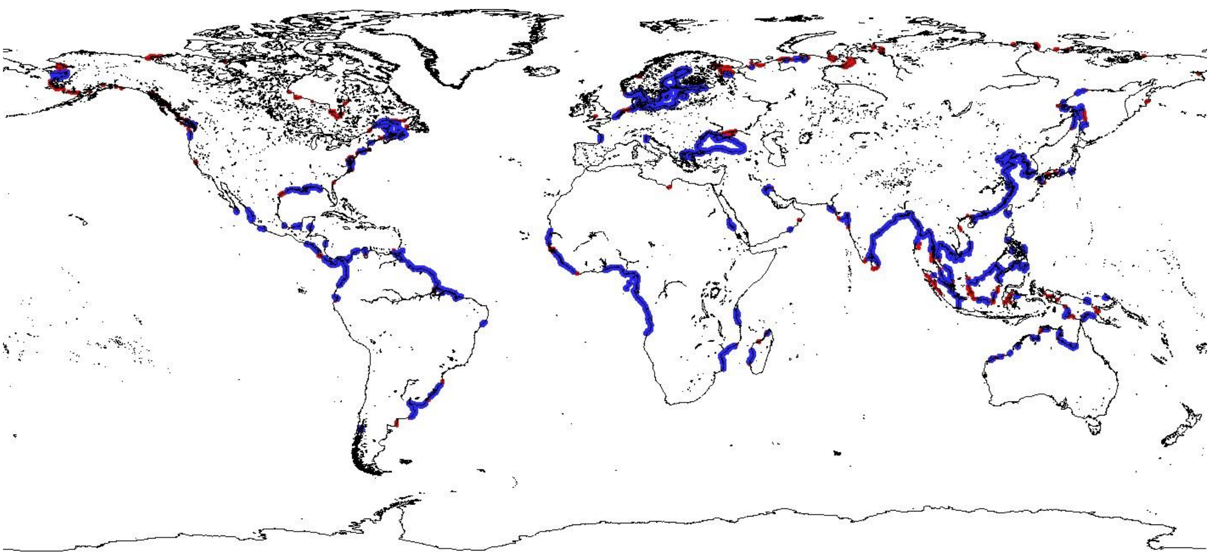

Figure 93: Areas suitable for Vibrio bacteria transmission in 1982-1991 (blue), and areas with conditions newly suitable for Vibrio transmission in 2014-2023 (red).

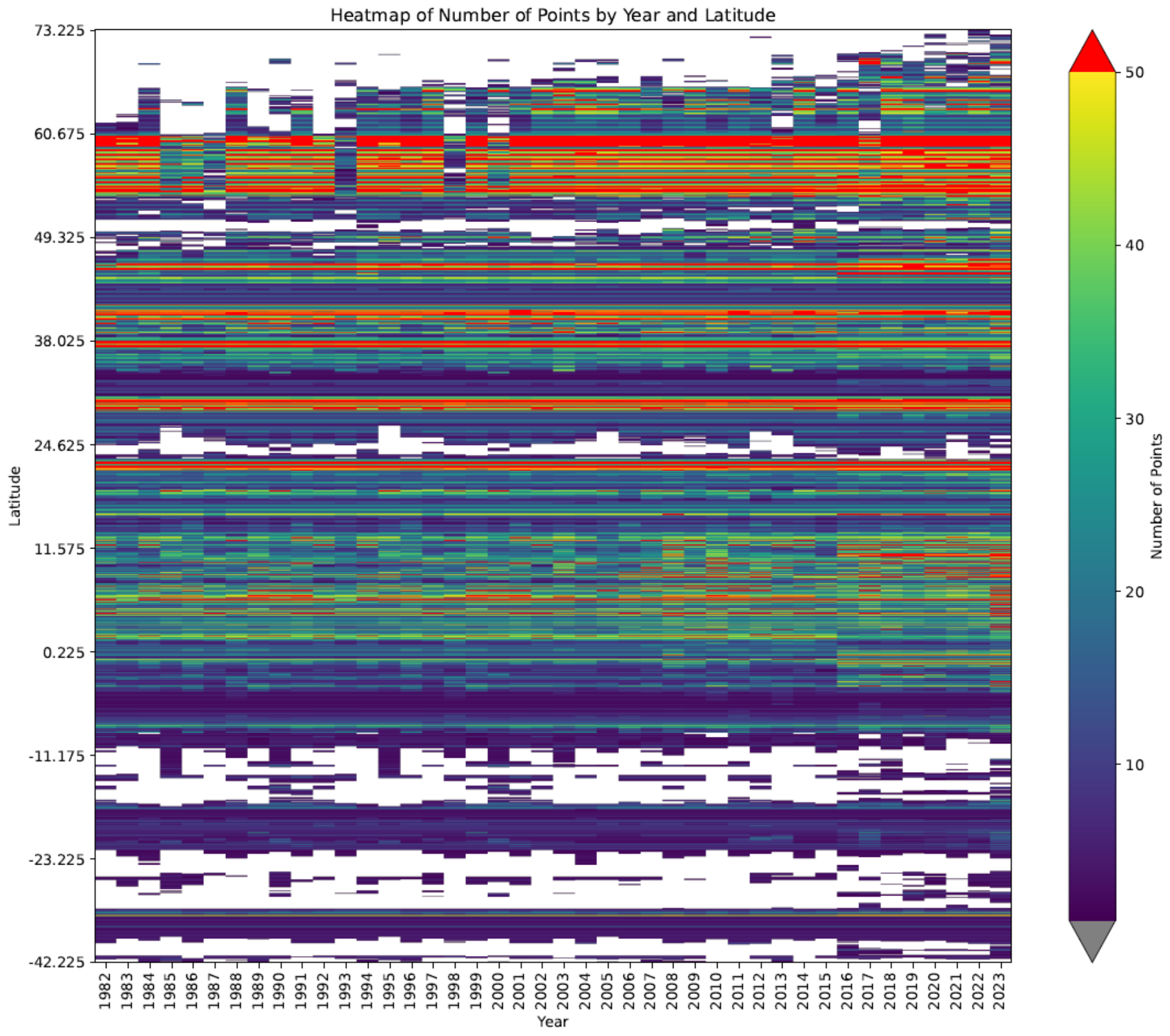

Figure 94: number of coastal grid points showing conditions suitable for vibrio transmission per year, and per latitude.

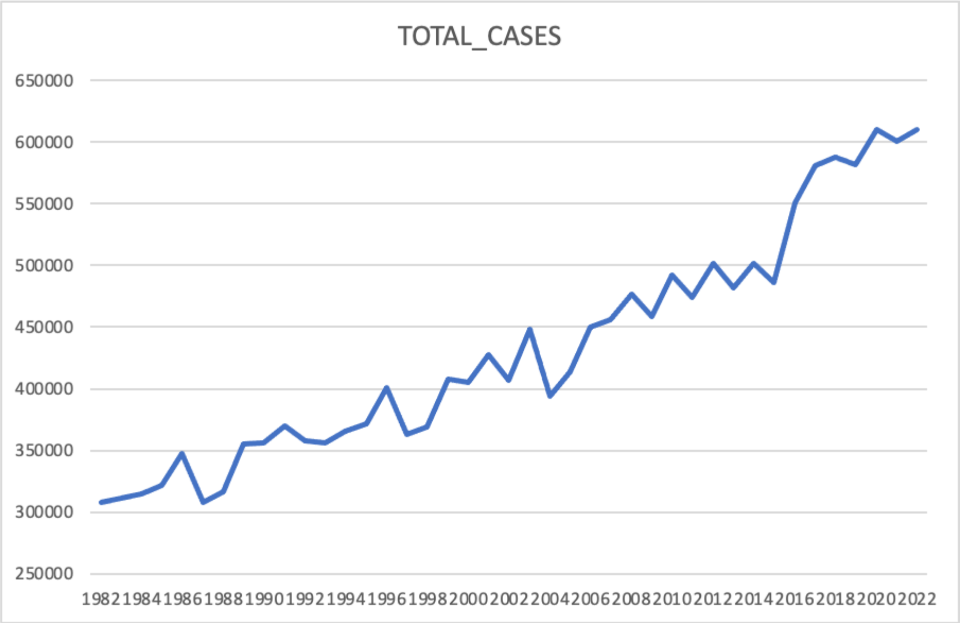

Figure 95: Number of cases per year (historical series).

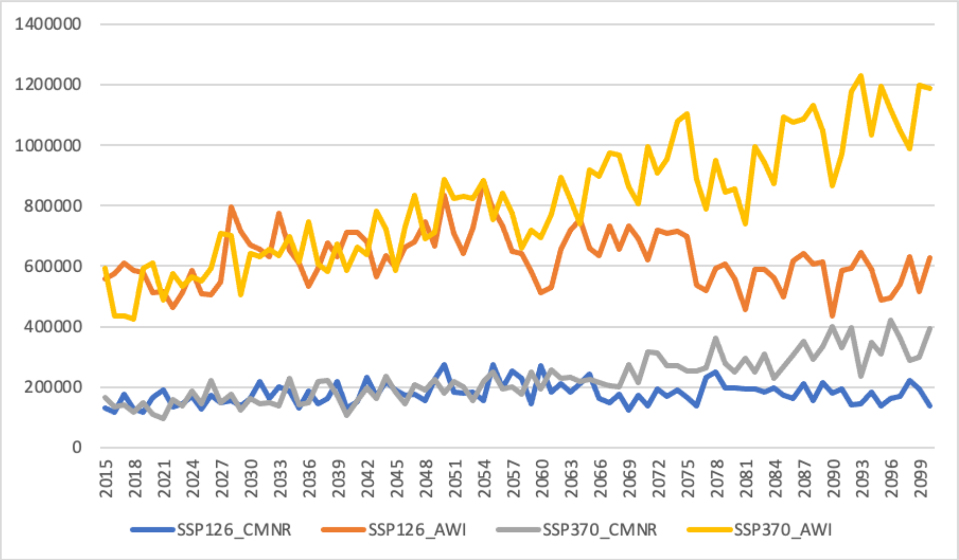

Figure 96: Time series of estimated cases per CMIP6 model and SSP. The differences in the values between models are the result of different coverage in highly populated areas, mostly in China and India.

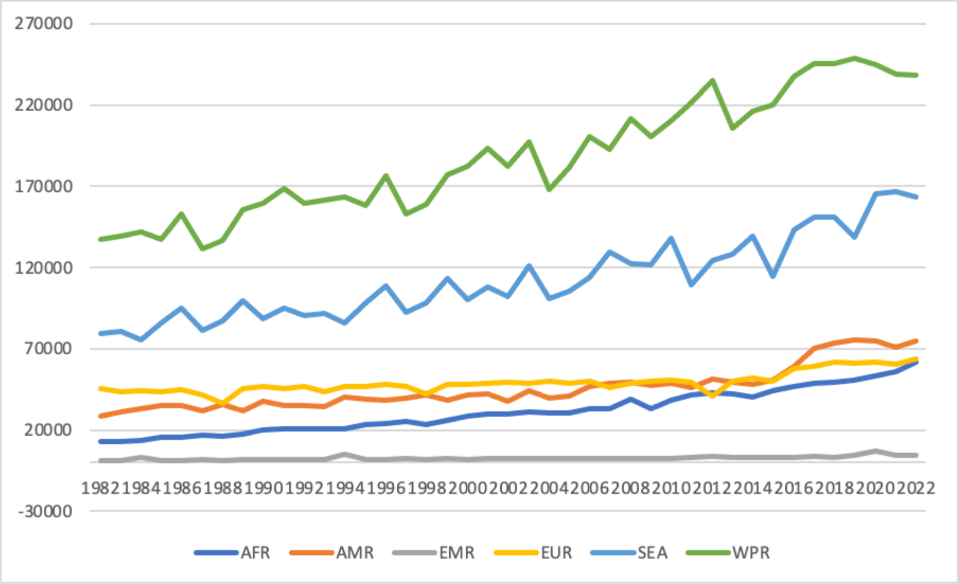

Figure 97: Temporal evolution of the number of cases per WHO region. The figure shows a positive trend over the study period with higher values in more densely populated areas in the Western Pacific Region and Southeast Asia.

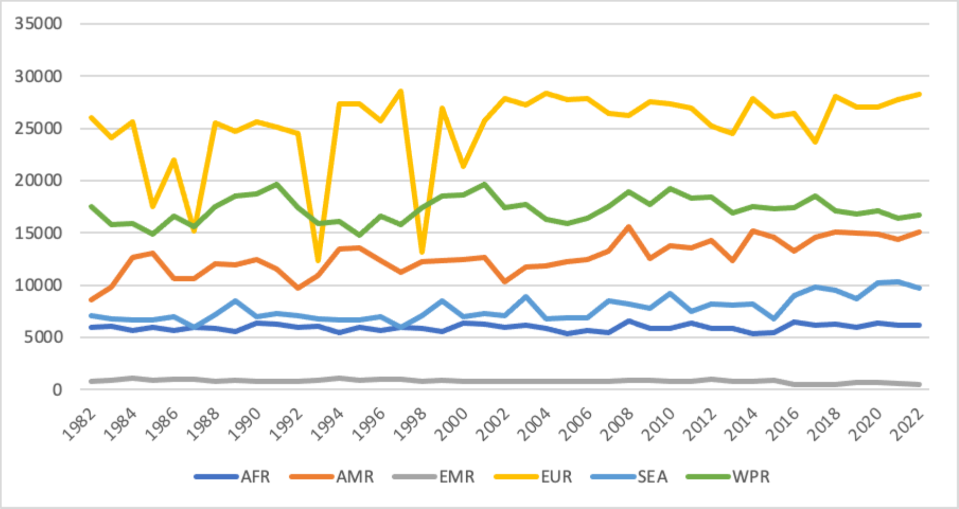

Figure 98: Length of coastline showing suitable conditions for Vibrio per WHO region. The increasing trend is larger in the European Region and the Region of the Americas

.

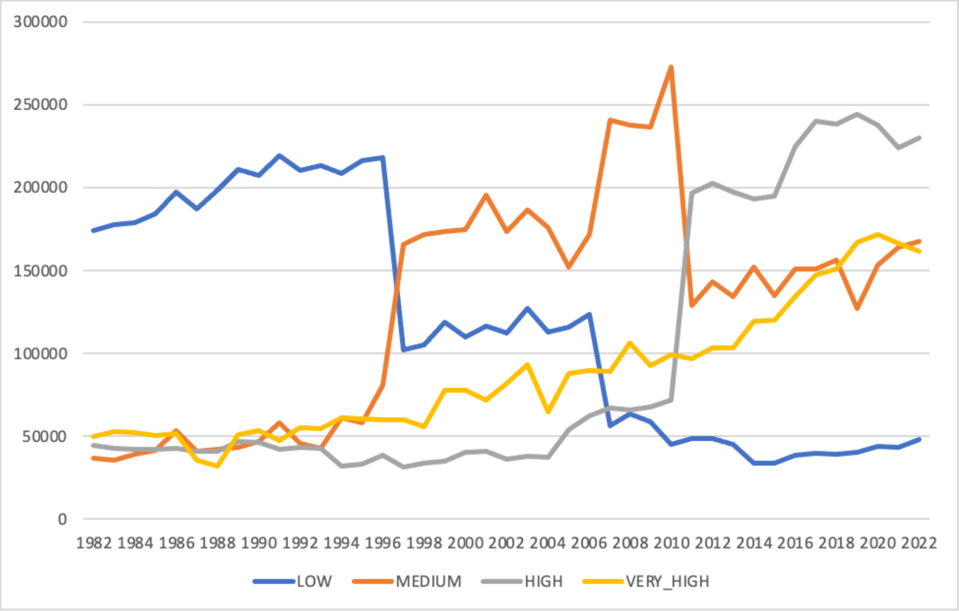

Figure 99: Temporal changes in the number of cases per HDI category. The abrupt changes in the time series correspond to when countries transition from one HDI category to another (e.g. China moving from low to medium in 1997, and from medium to high in 2011).

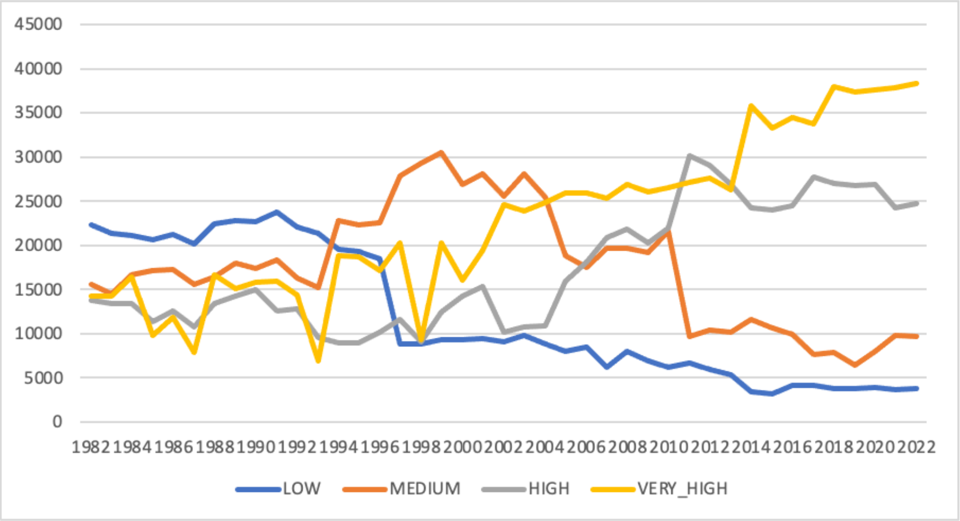

Figure 100: Length of coastline (in km) showing Vibrio suitability at any one point during the year per HDI region. The trend varies across HDI categories, and it is more pronounced for High and Very High, as more countries migrated from lower levels.

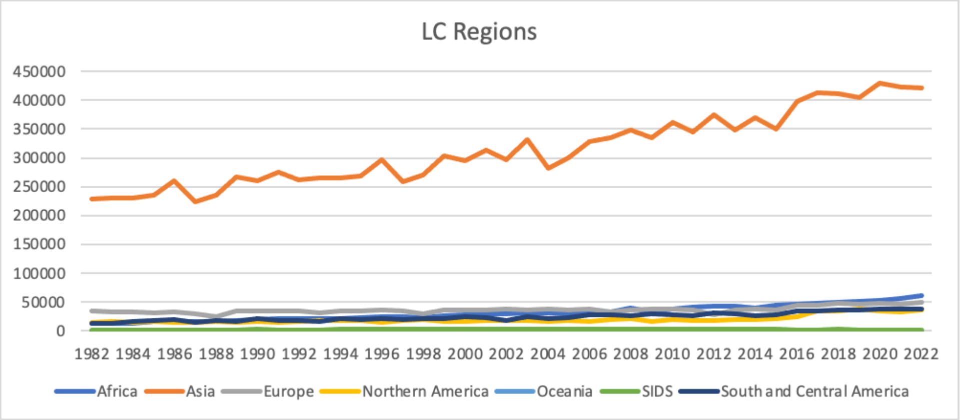

Figure 101: Time series of the number of cases per LC region. Asia emerges as the region with the highest number of estimated cases worldwide. After Asia, the regions with the highest number of cases are Europe and Africa, the latter showing more infections in recent years.

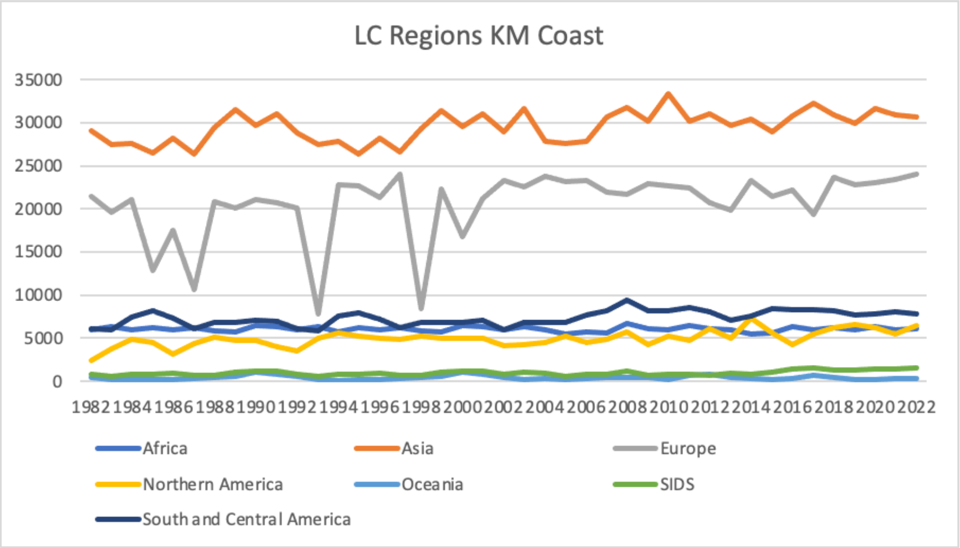

Figure 102: Length of coastline per LC region. Although the values for Asia and Europe are in the same range of magnitudes, it is important to highlight that the population are risk is very much higher in the first case.

Indicator 1.3.4: West Nile virus

Indicator Authors

Julian Heidecke, Prof Joacim Rocklöv, Dr Marina Treskova

Methods

The input data for this indicator have been extended for the 2024 report.

The West Nile virus (WNV) indicator monitors changes in the temperature suitability for WNV transmission by tracking its basic reproduction number.

WNV is a vector-borne pathogen that is maintained in a transmission cycle between mosquitoes (primarily genus *Culex*) and birds from which it can spill over into human populations. Human infection with WNV can lead to rare but severe and even life-threatening disease. Currently, there are no pharmaceutical prevention or treatment options for WNV infection in humans. Since its intrusion into the Americas in 1999, the pathogen occurs almost globally facilitated through the widespread occurrence of competent *Culex* mosquitoes. In the USA alone, more than 51,000 human clinical cases have been reported in this period, including over 2,300 deaths.[193] Europe has seen a geographic range expansion and increase in WNV transmission in recent decades. The largest yet recorded outbreak in Europe occurred in 2018 with 2,083 reported locally acquired infections in humans.[194] Climate change impacts WNV transmission in multifaceted ways.[195] In this regard, temperature is recognised as a key driver that impacts mosquito life-history traits and the ability of mosquitoes to transmit WNV.[196]
The basic reproduction number $\boldsymbol{R_0}$, i.e., the expected number of secondary infected hosts arising from a single infected host in a completely susceptible population, is a key metric in infectious disease epidemiology. Here, a trait-based model is used that approximates the temperature $(\boldsymbol{T})$ dependence of WNV $\boldsymbol{R_0}$:

$$\boldsymbol{R_0(T) = \frac{m(T)a(T)^2 b_m(T)p(T)^{n(T)} b_h}{-\ln(p(T))r_h}}$$

which includes the temperature dependent biting rate $\boldsymbol{a(T)}$, vector competence $\boldsymbol{b_m(T)}$, daily mosquito survival probability $\boldsymbol{p(T)}$ (given by $\boldsymbol{e^{-\mu_M(T)}}$, where $\boldsymbol{\mu_M(T)}$ is the mosquito mortality rate), length of the extrinsic incubation period $\boldsymbol{n(T)}$, the mosquito to host ratio $\boldsymbol{m(T) = M(T)/N}$, as well as the host infection probability $\boldsymbol{b_h}$, and host recovery rate $\boldsymbol{r_h}$. The temperature dependence of these traits is incorporated based on the work by Shocket et al. who derived temperature response functions for the above mosquito-pathogen traits and several mosquito-virus pairs based on laboratory data.[196] The indicator considers three WNV vectors, namely *Cx. pipiens*, *Cx. tarsalis*, and *Cx. quinquefasciatus*. Taken together these species inhabit a vast geographical area globally and are often considered key WNV vectors where they occur.[196]

To incorporate the impact of temperature on the mosquito to host ratio, a proxy for $\boldsymbol{M(T)}$ is derived from a mosquito population dynamic model incorporating the temperature-dependent life history traits. The population dynamic model is given by:

$$\boldsymbol{\dot{J} = \beta(T)M - \left(1 + \frac{J}{K}\right)\mu_J(T)J - \gamma(T)J}$$
$$\boldsymbol{\dot{M} = \omega\gamma(T)J - \mu_M(T)M}$$

where $\boldsymbol{J}$ represents individuals in the juvenile aquatic stage (encompassing eggs, larva, and pupa) and $\boldsymbol{M}$ represents adult female mosquitoes. The model includes the temperature-dependent oviposition rate $\boldsymbol{\beta(T)}$, the juvenile mortality rate $\boldsymbol{\mu_J(T)}$, the mosquito development rate $\boldsymbol{\gamma(T)}$, and the adult mortality rate $\boldsymbol{\mu_M(T)}$. The proportion of female mosquitoes at adult emergence $\boldsymbol{\omega}$ is assumed to be 0.5. Furthermore, the model incorporates a growth-limiting density-dependent juvenile mosquito mortality controlled by the parameter $\boldsymbol{K}$. The egg-to-adult survival probability $\boldsymbol{p_{EA}(T)}$ in Shocket et al. was used to calibrate the temperature-dependence of the juvenile mortality rate $\boldsymbol{\mu_J(T)}$.[196] The adult female demographic equilibrium given by:

$$\boldsymbol{M(T) = K\frac{\omega^2\beta(T)\gamma(T)^2}{\mu_M(T)^2\mu_J(T)}\left(1 - \frac{\mu_M(T)}{\omega\beta(T)p_{EA}(T)}\right)}$$

is used as a proxy for mosquito abundance in the $\boldsymbol{R_0}$ model whereby $\boldsymbol{M(T) = 0}$ if the population reproduction number $\boldsymbol{\omega\beta(T)p_{EA}(T)/\mu_M(T)}$ is less than or equal to one. The parameter $\boldsymbol{K}$ would depend on a multitude of factors determining the availability of suitable mosquito breeding habitat. To avoid incorporating elusive relationships into the model, $\boldsymbol{K}$ is excluded from the expression for $\boldsymbol{M(T)}$. This results in a dimensionless proxy for $\boldsymbol{M(T)}$ that isolates the impact of temperature via the mosquito life-history traits.

Similarly, the host recovery rate $\boldsymbol{r_h}$, the host infection probability $\boldsymbol{b_h}$, and the host density $\boldsymbol{N}$, are not directly temperature dependent and were therefore excluded from the formula for $\boldsymbol{R_0}$. The resulting relative $\boldsymbol{R_0}$ model was rescaled to $[0,1]$. It represents a relative measure of the temperature dependent transmission risk space with upper and lower thermal limits and a temperature optimum (obtained between 23°C and 26°C varying between species) described by the nonlinear interaction of the included species-specific and temperature-dependent mosquito-virus traits.

ERA5-Land temperature data was used to calculate relative $\boldsymbol{R_0}$ at 0.1°x0.1° spatial resolution.[197,198] The species-specific relative $\boldsymbol{R_0}$ was applied in each vectors' distribution range described by georeferenced versions of the species distribution maps in Shocket et al.[196] To obtain a combined global indicator, in areas where *Cx. pipiens* and *Cx. quinquefasciatus* overlap in the Americas, Asia, and Australia, the relative $\boldsymbol{R_0}$ temperature response of the two species was averaged. In these regions there is typically genetic introgression between the two species, and it was assumed that the resulting hybrid populations would yield a relative $\boldsymbol{R_0}$ temperature response "in between" the response of the original species. For the overlapping regions in Africa (where *Cx. pipiens* and *Cx. quinquefasciatus* do not substantially hybridise) as well as the overlapping regions with *Cx. tarsalis*, the maximum out of the relative $\boldsymbol{R_0}$'s was taken. This was based on the simplifying assumption that the species with the highest transmission suitability would dominate WNV transmission. The gridded combined monthly relative $\boldsymbol{R_0}$ were aggregated by country, WHO regions, LC groupings, and human development index (HDI).

Data

- Monthly temperature data from the European Centre for Medium-Range Weather Forecasts (ECMWF) ERA5-Land reanalysis: global, 1950–2022, 0.1° grids[197,198]
- Temperature response data: Data from Shocket et al. showing temperature response of mosquito-pathogen traits for specific WNV-mosquito species combinations[196]
- Mosquito species distribution maps shown in Shocket et al[196]
-

Caveats

The indicator is limited to three key WNV vectors and their spatial distribution. Multiple other mosquito species are known to be competent for WNV transmission but currently lack robust transmission suitability models and could therefore not be considered. Moreover, intraspecific differences in temperature suitability of mosquito populations are neglected. The indicator isolates the temperature-dependence of the basic reproduction number via mosquito-virus traits. Impacts of climate change beyond these relationships such as impacts of changing precipitation patterns or variations in WNV host populations (impacting host abundance, host species composition, and host community competence) are currently not considered. Mosquito distribution ranges are incorporated based on coarse-grained published distribution maps and do not track potential range

expansions/contractions due to climate change. The relative $\boldsymbol{R_0}$ model underlying the indicator does not allow interpretation as a threshold parameter for outbreaks or an absolute measure of secondary infections such as the classical basic reproduction number. Despite this limitation tracking changes in relative $\boldsymbol{R_0}$ is informative of whether WNV transmission temperature suitability increases or decreases. Currently the indicator utilises monthly averaged temperature data.

Future form of the indicator

Future versions of the indicator will seek to address the caveats listed above. Moreover, following iterations of the indicator will seek to incorporate updated versions of the temperature response of mosquito-virus traits.

Additional analysis

Figure 103 and figure *104* shows the yearly average relative $\boldsymbol{R_0}$ on a global scale (combined mosquito ranges) as well as the breakdown by HDI country groups. The results of aggregations by WHO regions and LC groupings are shown in figure *105* and figure 106, respectively. Increases in temperature suitability for WNV transmission have occurred in the Africa (3.1%), Americas (6.6%), Europe (20.3%), South-East Asia (3.0%), and Western Pacific (5.0%) WHO regions in 2014–2023 compared to 1951–1960, while the Eastern Mediterranean WHO region has experienced a decrease in the same period (-8.4%). The percentage changes in WNV temperature suitability observed by LC groupings in 2014–2023 compared to 1951–1960 are 1.0% in Africa, 4.4% in Asia, 43.4% in Europe, 5.4% in Latin America, 14.1% in Northern America, -2.0% in Oceania, and 3.6% in SIDS.

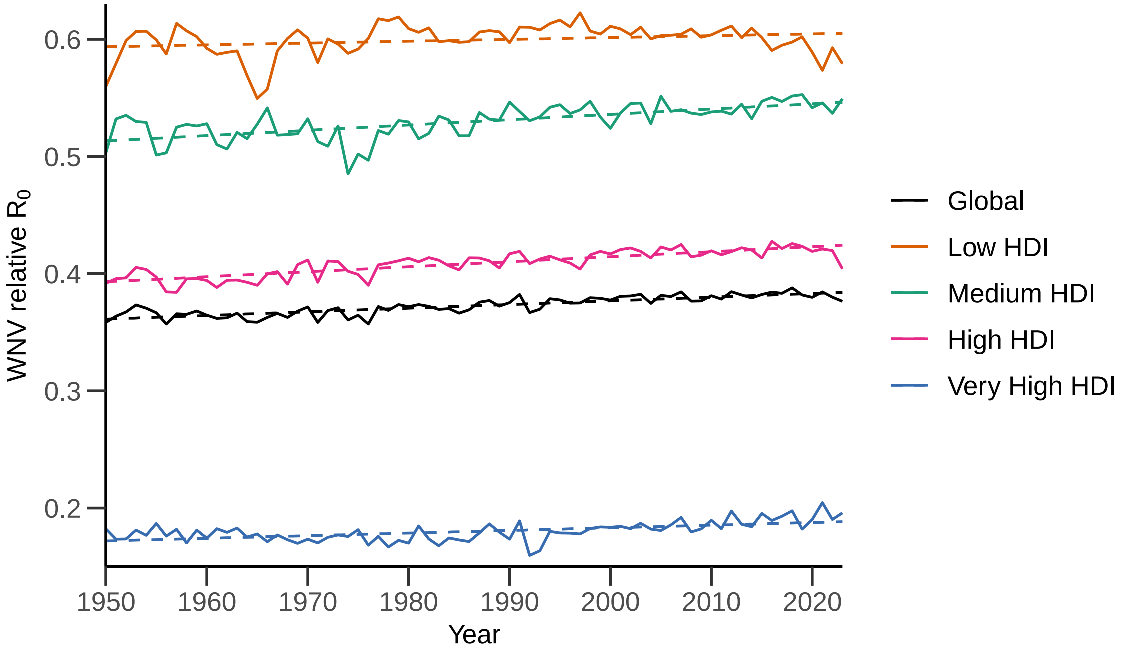

Figure 107: Yearly average WNV relative $\boldsymbol{R_0}$ *contrasting results for the global (combined mosquito ranges) indicator and by HDI country group in the period 1950–2023.*

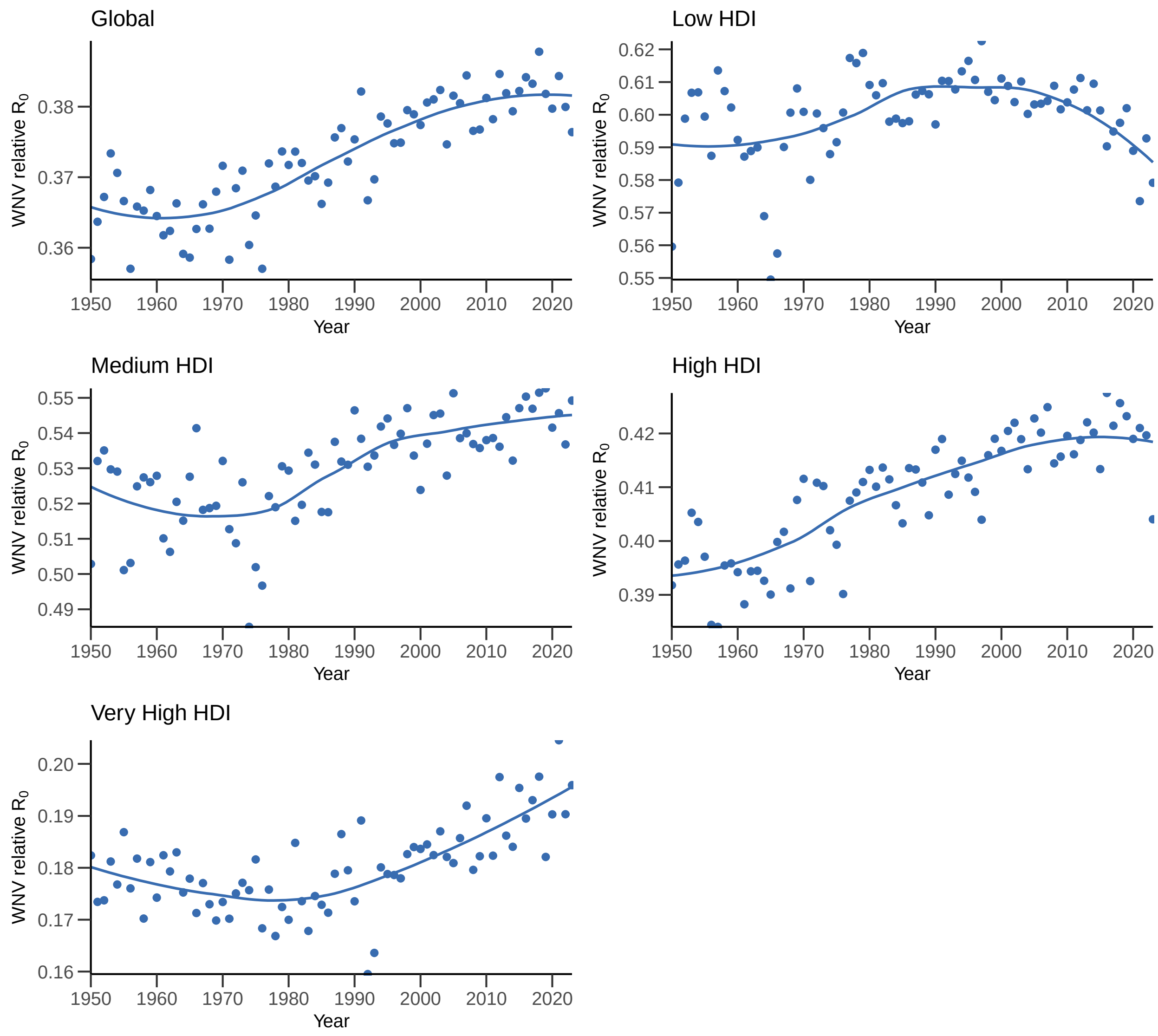

Figure 108: Yearly average WNV relative $\boldsymbol{R_0}$ *for the global (combined mosquito ranges) indicator and by HDI country group in the period 1950–2023 in individual panels.*

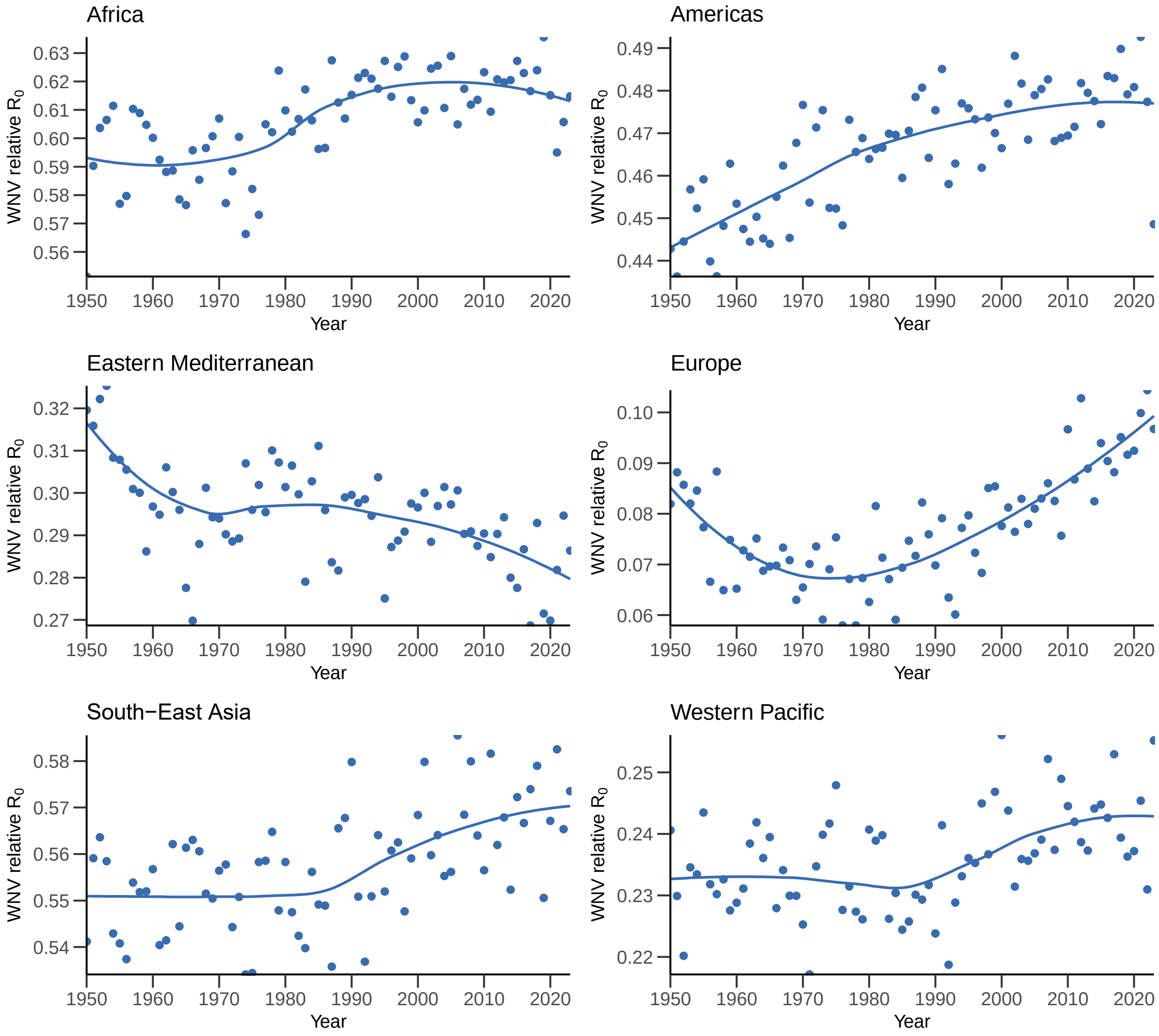

Figure 109: Yearly average WNV relative $\boldsymbol{R_0}$ by WHO region in the period 1950–2023.

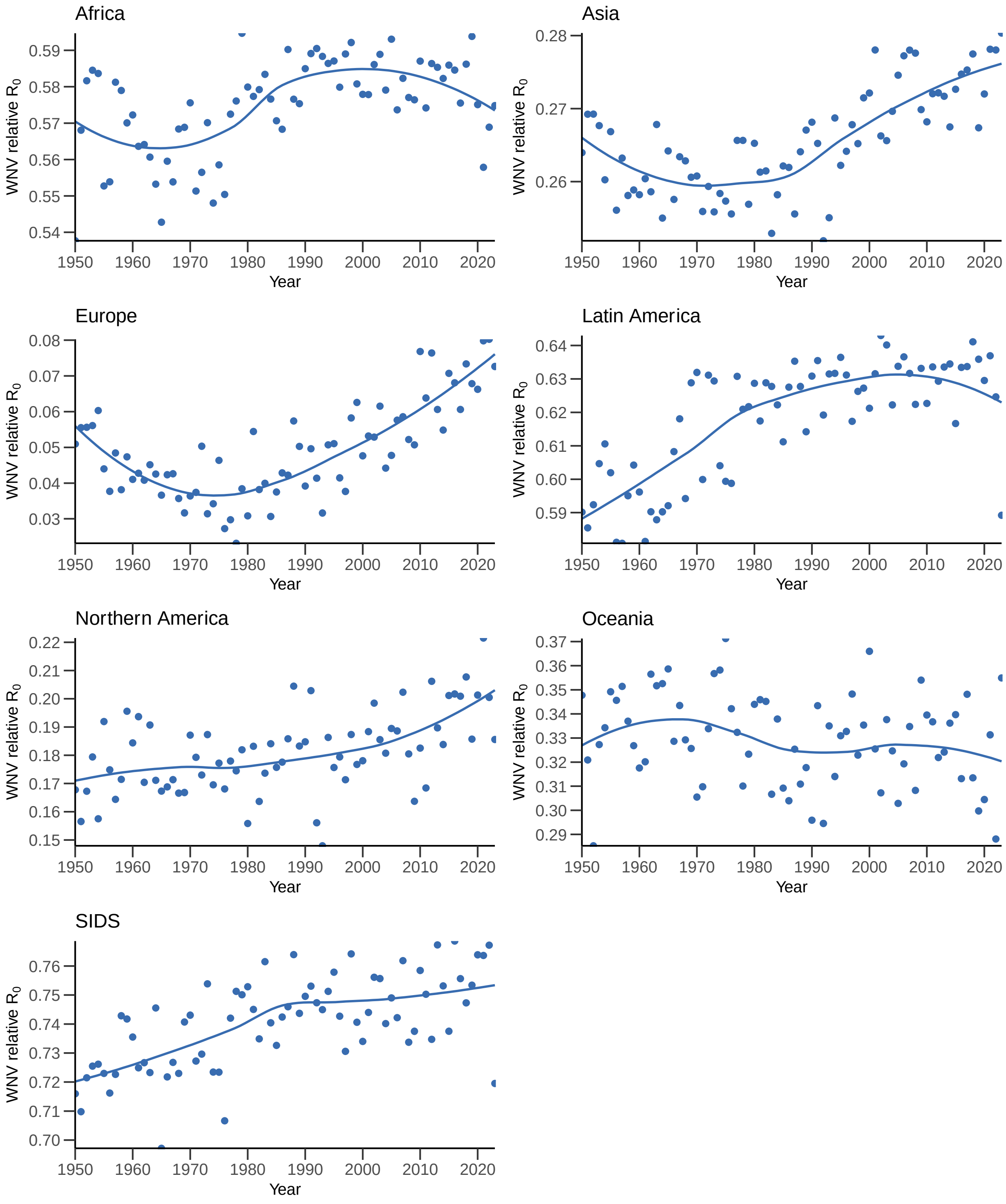

Figure 110: Yearly average WNV relative $\mathbf{R_0}$ *by LC group in the period 1950–2023.*

Indicator 1.4: Food security and undernutrition

Food Security And Undernutrition

Indicator Section Authors

Dr Shouro Dasgupta, Prof Elizabeth J.Z. Robinson

Methods

The methodology of this indictor is based on Dasgupta and Robinson.[199] To track the impact of climate change and income on the incidence of food insecurity, a panel data regression is used with coefficients that vary over time. To operationalise the concept of climate change, a focus on the number of heatwave days, and the frequency of droughts, during the four major crop growing seasons in each region is used.[200] A heatwave is defined as a period of at least two days where both the daily minimum and maximum temperatures are above the 95th percentile of the respective climatologies (Indicator 1.1.2) in each region. The gridded 95th percentile of daily minimum and maximum temperatures, taken from the ERA5-Land hourly dataset,[201] were calculated for 1986–2005. The lagged number of heatwaves and frequency of droughts (measured by SPEI-12) is used during the crop growing seasons for each year during 2014–2022.

Increase in the number of heatwave days can affect food insecurity through multiple pathways.[202] These can variously be through the impacts of heat stress and droughts on crop yields, on agricultural labour and therefore crop production and agricultural income, on non-agricultural labour and non-agricultural income, on health and the ability to earn enough to afford food, on food prices and therefore the affordability of food, and on food supply chains and therefore the variety of food (which we can summarise as income and food supply effects). Our regression also includes twelve-month Standardized Precipitation Evapotranspiration Index (SPEI) as a measure of drought. SPEI-12 was computed using precipitation data from ERA5-Land monthly averaged dataset[200] and the SPEI package in R.[203]

We consider two dependent variables: first, the probability of moderate to severe food insecurity; and second the probability of severe food insecurity from the FAO Food Insecurity Experience Scale (FIES).[204] To account for unobserved heterogeneity such as differences in food and storage policies across countries and changes in the prices of food items from year to year, our specification also includes both location and time (year) fixed-effects. The standard errors are clustered at the country-level.[205] Our panel data specification can be written as follows:

$$FIES_{it} = \beta_1(\tau_t) + V_{(it)} + \gamma'(\tau_t)X_{(it)} + \alpha_{(i)} + \mu_{(it)}$$

where $FIES_{it}$ is the probability of moderate or severe food insecurity or probability of severe food insecurity, $\boldsymbol{V}_{it}$ is a vector of change in the number of heatwave days and the frequency of drought months during the four major crop growing season, and $\boldsymbol{X}_{it}$ is a vector of relevant variables affecting food insecurity – income and a dummy to control for the COVID-19 pandemic in 2020. $\boldsymbol{\mu}_{it}$ is a random error term. All variables are recorded for different locations with index $i = 1, \ldots, N$ and over a number of years $t = 1, \ldots, T$. The time-varying coefficients allow us to examine whether the relationship between temperature anomaly and food insecurity has evolved over time.

In the second-step, we conduct a counterfactual analysis to explore the extent to which food insecurity may have been affected by climate change.[199] To do this we compute the cumulative impacts of increasing frequency of heatwaves and frequency of drought months above the historical norms over

the period 1981–2010. The counterfactual impact of climate change on food insecurity is derived by combining the coefficients from the time-varying regression with the historical norm average and each year for which we have food security data. We consider the effects of increases in the frequency of heatwaves and frequency of drought months over compared to the baseline (1981–2010) under which frequency of heatwaves increases according to its historical trend.

Data

- Temperature and SPEI: ERA5-Land[201]
- Food insecurity: FAO Food Insecurity Experience Scale[204]
- ISIMIP3b[206]

FIES "represents a significant change in approach to food insecurity measurement compared to traditional ways of assessing it indirectly through determinants such as food availability or consequences such as poor-quality diets, anthropometric failures and other signs of malnutrition." The indicator is "developed by professionals from the nutrition field," includes quantity and quality measures, making it particularly relevant for the Lancet Countdown and its focus on climate change and health. "FIES provides a tool for the nutrition and food security community to build on existing knowledge regarding relationships between the experience of food insecurity and indicators of malnutrition".

Caveats

The main caveat the food insecurity indicator is the possible recall bias in the survey data and the bias that may have been induced to interviews during the pandemic being conducted by phone instead of in-person visits.

Future form of the indicator

In the future, provide disaggregated analysis by HDI groups will be provided.

Additional analysis

Table 21: Relationship between frequency of heatwaves and droughts, and food insecurity during 2014-2022 using a time-varying regression. 95% confidence intervals in parentheses.

	**Moderate or severe food insecurity**
Low income	0·185
	(0·177, 0·193)
High income	-0·114
	(-0·110, -0·118)
COVID-19 dummy	0·163
	(0·155, 0·171)
Heatwave frequency$_{(t)}$	
2014	1·15
	(1·00, 1·30)
2015	1·52
	(1·40, 1·64)
2016	1·93
	(1·80, 2·06)
2017	2·27
	(2·16, 2·38)
2018	2·96
	(2·84, 3·08)
2019	3·32
	(3·22, 3·42)
2020	3·72
	(3·66, 3·78)
2021	4·04
	(3·94, 4·14)
2022	4.40(4.32, 4.48)
Drought frequency$_{(t)}$	
2014	0·56
	(0·41, 0·71)
2015	0·59
	(0·49, 0·69)
2016	0·74
	(0·62, 0·86)
2017	0·92
	(0·85, 0·99)
2018	1·12
	(0·99, 0·1·25)
2019	1·34
	(1·20, 1·48)
2020	1·59
	(1·43, 1·75)
2021	1·80
	(1·70, 1·90)
2022	2.00(1.92, 2.08)

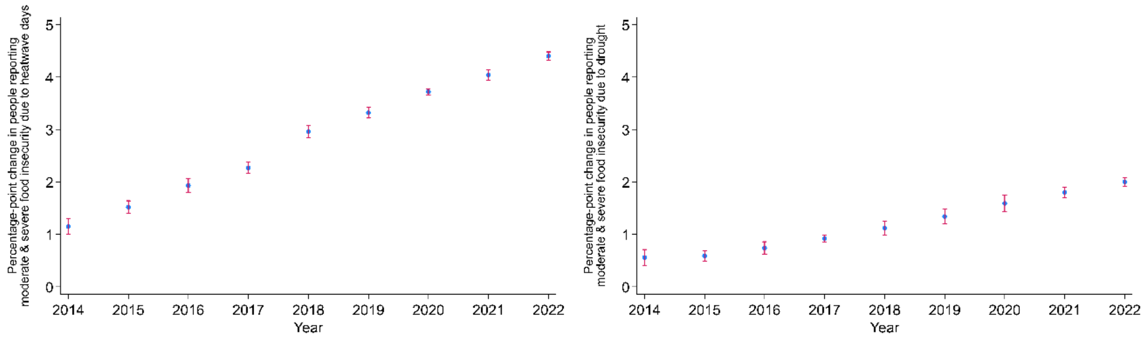

Figure 111: Change in the share of the population (percentage point change) reporting moderate or severe food insecurity (as defined by FIES) due to heatwave days (left–panel) and frequency of drought months (right–panel) occurring during four major crop (maize, rice, sorghum, and wheat) growing seasons.

Marine Food Productivity

Indicator Section Authors

Prof Maziar Moradi-Lakeh, Dr Fereidoon Owfi, Dr Mahnaz Rabbaniha, Prof Meisam Tabatabei

Methods

Sixteen major marine basins and five inland water basins (FAO Fishing Areas), which are important in terms of projected impacts and vulnerabilities associated with climate change, were selected (Table 23). The remaining three basins are located in the Antarctica.

The countries/territories (N=148) located in these basins were selected to attribute climate change impacts, more specifically, Sea Surface Temperature (SST), to the deterioration of major coral reef sites and the consequent decreased per capita consumption of capture-based fish. A total number of 789,624 data points based on position and time were used to have a more accurate estimate for SST variations.

The availability of measured SST data (obtained from ORAS5: *Ocean Reanalysis System 5*) ranged from 1980 to 2023, while the projected data were available for 2015–2100 and were presented in annual intervals for 2040–2060 (*Figure* 116).

The data considered on coral reef sites (NOAA Coral Reef Watch), i.e., annual maximum Bleaching Alert Area caused by thermal stress, were presented in five-year intervals (1985–2022).

Moreover, the data concerning capture-based and farmed-based per capita fish consumption in the investigated countries from 1980–2021 were collected and analysed.

Disability-adjusted life years (DALYs) attributed to diet low in seafood ω3 was provided compared to the burden attributable to all other risk factors (Figure 117).

Data

- ORAS5 (Ocean Reanalysis System 5)[207]
- UNEP status of coral reefs of the world, 2020[208]
- IUCN, Marine Protected Areas[209]
- Ramsar, International Wetland[210]
- UNESCO Biosphere Reserves[211]
- UNESCO World Heritage sites
- World Resources Institute, marine protected areas of the world
-

Caveats

There is a lack of information and data in the available databases such as FAO on fish species composition of the captured and farmed fish products. This could, in turn, lead to some concerns about the methodological approach used to calculate ω3 intake. More specifically, most of the approaches are based on fish intake, which usually ignores or underestimates variations in ω3 contents of different types of fishes, and especially capture-based compared with farmed-based fish. It should also be highlighted that GBD estimates for the association between this dietary risk factor and cardiovascular diseases, as the primary reference for human health impacts, are not based on type and source of seafood products either.

Fish production data were used as a surrogate for fish consumption. This is not a completely accurate assumption, but there is no comprehensive alternative source of data for all the investigated countries.

Future Form of the Indicator

Further analysis will be required to connect the different components of the causality chain, i.e., between SST and health impacts.

Additional Analysis

Although the North America and European regions still have the lowest average sea surface temperature, they are projected to experience the highest increase by the year 2060 (table 22). Despite an increase in per capita farm-based fish consumption globally, the share of capture-based in total fish consumption has continued to decrease (figure 112). The increasing sea surface temperature well supports the thermal stress-induced deterioration of coral bleaching status and the consequent decline in fish capture (figure 113 and figure 114, respectively).

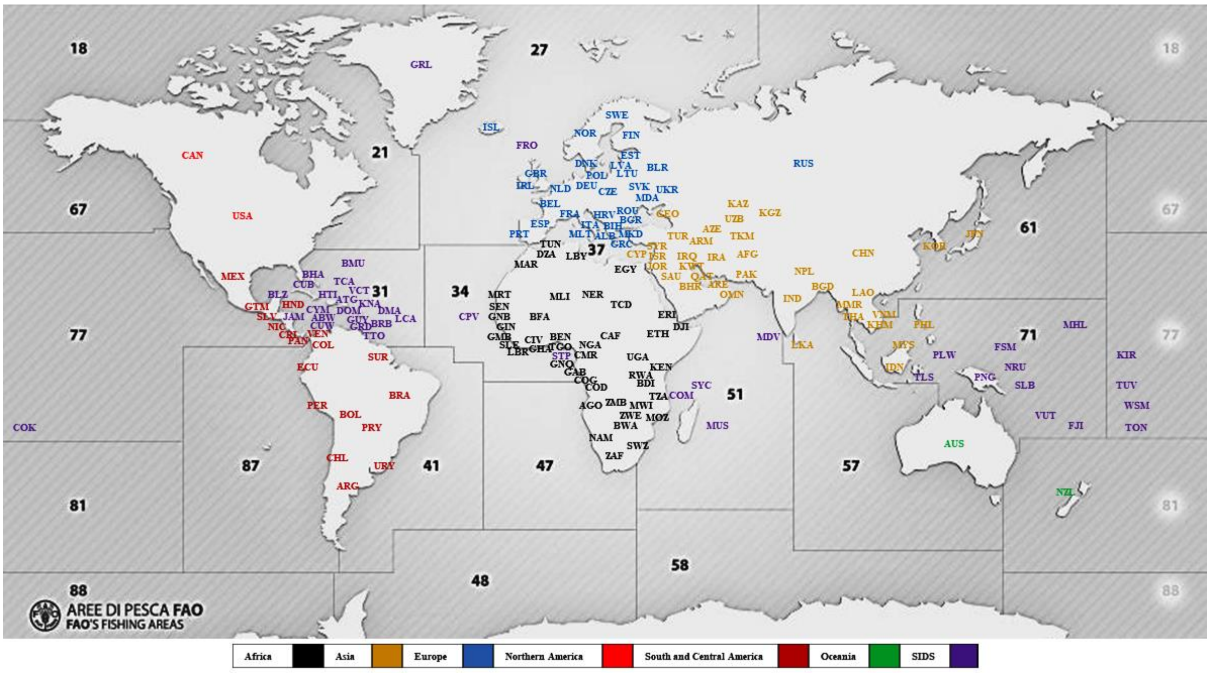

Figure 115: The geographical location of selected countries and their respective marine basins (FAO fishing areas – adapted from source of map: http://www.fao.org/tempref/fi/maps/Default.htm). Different colours represent different Lancet Countdown regions.

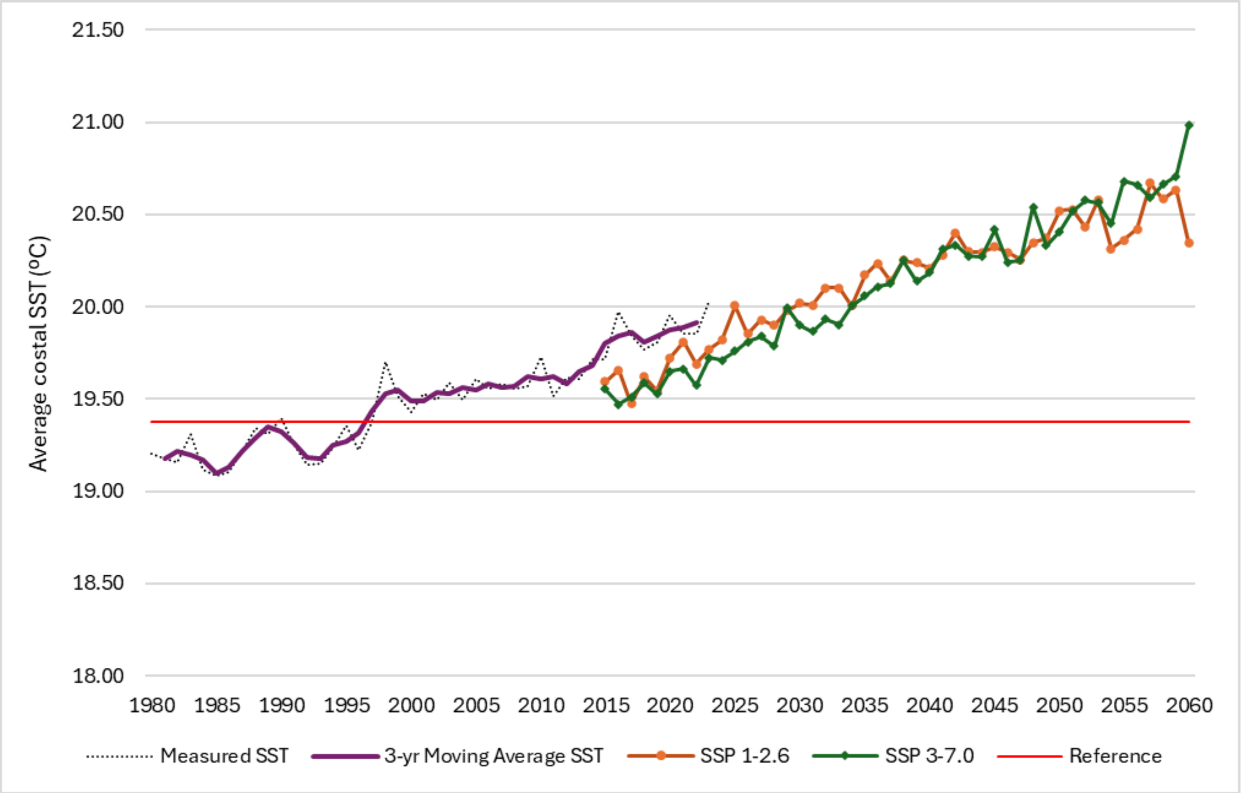

Figure 116. Trend of the measured sea surface temperature (SST) and its 3–year moving average in the global coastal areas (1980–2023), and the projected values under the SSP1–2·6 and SSP3–7.0 scenarios (Red line: the 1981–2010 baseline).

*Table 23: Scope of the investigation by country/territories, marine/inland basin, FAO fishing area, Lancet Countdown (LC) grouping, WHO country grouping, HDI country classification, and coral reef site**

No.	Country / Territory	ISO 3 country code	FAO Fishing Area	Marine / Inland Basin	LC Grouping	WHO Region	HDI Level	Coral reef location (marine basin)	Marine Protected Areas (MPAs) with Coral Reefs
1	Botswana	BWA	01	Inland Waters	Africa	Africa	Medium	-	-
2	Burkina Faso	BFA			Africa	Africa	Low	-	-
3	Burundi	BDI			Africa	Africa	Low	-	-
4	Central African Republic	CAF			Africa	Africa	Low	-	-
5	Chad	TCD			Africa	Africa	Low	-	-
6	Eswatini	SWZ			Africa	Africa	Medium	-	-
7	Ethiopia	ETH			Africa	Africa	Low	-	-
8	Malawi	MWI			Africa	Africa	Low	-	-
9	Mali	MLI			Africa	Africa	Low		
10	Niger	NER			Africa	Africa	Low	-	-
11	Rwanda	RWA			Africa	Africa	Low	-	-
12	Uganda	UGA			Africa	Africa	Low	-	-
13	Zambia	ZMB			Africa	Africa	Medium	-	-
14	Zimbabwe	ZWE			Africa	Africa	Medium	-	-
15	Canada	CAN	02	Inland Waters	Northern America	Americas	Very High	-	-
			18	Arctic Sea				-	-
			21	North-West Atlantic				-	-
			67	North-East Pacific				-	-
16	United States of America	USA	02	Inland Waters	Northern America	Americas	Very High	-	-
			21	North-West Atlantic				-	-
			31	West-Central Atlantic				-	-
			67	North-East Pacific				-	-
			77	East-Central Pacific				-	-
17	Bolivia	BOL	03	Inland Waters	South and Central America	Americas	Medium	-	-
18	Paraguay	PRY			South and Central America	Americas	High		
19	Brazil	BRA	03	Inland Waters	South and Central America	Americas	High	-	-
			41	South-West Atlantic				West Coast of Atlantic Ocean	Parque Estadual Marinho do Parcel Manoel Luis,<br>Abrolhos Bank,<br>Atol das Rocas, Parcel Manoel Luis, Fernabdo de Noronha,<br>Recife de Fora, Amazon Reef
20	Kyrgyzstan	KGZ	04	Inland Waters	Asia	Europe	Medium	-	-

No.	Country / Territory	ISO 3 country code	FAO Fishing Area	Marine / Inland Basin	LC Grouping	WHO Region	HDI Level	Coral reef location (marine basin)	Marine Protected Areas (MPAs) with Coral Reefs
21	Armenia	ARM			Asia	Europe	High	-	-
22	Afghanistan	AFG			Asia	Eastern Mediterranean	Low	-	-
23	Lao People's Democratic Republic	LAO			Asia	Western Pacific	Medium	-	-
24	Nepal	NPL			Asia	South-East Asia	Medium	-	-
25	Kazakhstan	KAZ			Asia	Europe	Very High	-	-
26	Azerbaijan	AZE	04	Inland waters, Caspian Sea,	Asia	Europe	High	-	-
27	Turkmenistan	TKM			Asia	Europe	High	-	-
28	Uzbekistan	UZB	04	Inland waters, Aral Sea,	Asia	Europe	High	-	-
29	Islamic Republic of Iran	IRA	04	Inland waters, Caspian Sea,	Asia	Eastern Mediterranean	High	-	-
			51	West Indian				Persian Gulf, Hormoz Strait, Gulf of Oman	Dayyer and Nakhilo, Nayband Bay, Chabahar Bay, Islands: Kharg, Kharko, Qeshm, Hormoz, Hengam, Larak, Sheedvar, Lavan, Farour, Bani Farour, Kish, Hendourabi, Farsi
30	Pakistan	PAK	04	Inland waters	Asia	Eastern Mediterranean	Low	-	-
			57	East Indian				Arabian Sea, Gulf of Oman	Astola (Haft Talar) Island
31	India	IND	04	Inland waters	Asia	South-East Asia	Medium	-	-
			51	West Indian				East of Arabian Sea, Laccadive Sea, Gulf of Kutch	Gulf of Kutch
			57	East Indian				Bay of Bengal, Gulf of Mannar	Great Nicobar, Gulf of Mannar, Wandur (Mahatma Gandhi)
32	China	CHN	04	Inland waters	Asia	Western Pacific	High	-	-
			61	North-West Pacific				South China Sea	Kat o Cau, Shan Hu Jiao
33	Belarus	BLR			Europe	Europe	Very High	-	-
34	Czechia	CZE			Europe	Europe	Very High	-	-
35	Republic of Moldova	MDA	05	Inland Waters	Europe	Europe	High	-	-
36	North Macedonia	MKD			Europe	Europe	High	-	-
37	Slovakia	SVK			Europe	Europe	Very High	-	-
38	Russian Federation	RUS	05	Inland Waters	Europe	Europe	Very High	-	-
			18	Arctic Sea				-	-
			27	North-East Atlantic				-	-
			37	Mediterranean Sea, Black Sea				-	-
			61	North-West Pacific				-	-
39	Greenland	GRL	21	North-West Atlantic	SIDS	Europe	NA	-	-
			27	North-East Atlantic				-	-

No.	Country / Territory	ISO 3 country code	FAO Fishing Area	Marine / Inland Basin	LC Grouping	WHO Region	HDI Level	Coral reef location (marine basin)	Marine Protected Areas (MPAs) with Coral Reefs
40	Iceland	ISL	27	North-East Atlantic	Europe	Europe	Very High	-	-
41	United Kingdom	GBR			Europe	Europe	Very High	-	-
42	Ireland	IRL			Europe	Europe	Very High	-	-
43	Finland	FIN			Europe	Europe	Very High	-	-
44	Sweden	SWE			Europe	Europe	Very High	-	-
45	Denmark	DNK			Europe	Europe	Very High	-	-
46	Norway	NOR			Europe	Europe	Very High	Norwegian Sea	Sula and Rost Reef Deep Water Coral Reefs Sites
47	Estonia	EST			Europe	Europe	Very High	-	-
48	Latvia	LVA			Europe	Europe	Very High	-	-
49	Lithuania	LTU			Europe	Europe	Very High	-	-
50	Portugal	PRT			Europe	Europe	Very High	-	-
51	Germany	DEU			Europe	Europe	Very High	-	-
52	Poland	POL			Europe	Europe	Very High	-	-
53	Belgium	BEL			Europe	Europe	Very High	-	-
54	Netherlands	NLD			Europe	Europe	Very High	-	-
55	Faroe Islands	FRO			SIDS	Europe	NA	-	-
56	France	FRA	27	North-East Atlantic	Europe	Europe	Very High	-	-

No.	Country / Territory	ISO 3 country code	FAO Fishing Area	Marine / Inland Basin	LC Grouping	WHO Region	HDI Level	Coral reef location (marine basin)	Marine Protected Areas (MPAs) with Coral Reefs
			37	Mediterranean Sea, Black Sea				-	-
57	Spain	ESP	27	North-East Atlantic	Europe	Europe	Very High	East Coast of Atlantic Ocean	Pasito Blanco reef, Gran Canarias Islands
			37	Mediterranean Sea, Black Sea				-	-
58	Cuba	CUB	31	West-Central Atlantic	SIDS	Americas	High	Caribbean Sea, Gulf of Mexico	Peninsula de Guanahacabibes, Cienaga de Zapata, Cayo Sabinal, Subarchipielago de los Canarreos, Cayo Coco, Cayo Guillermo, Cayo Romano, CuchillasdelToa, Sur Isla, Cienaga de Zapata, Subarchipielago de Jardines de la Reina, Subarchipielago de Sabana-Camaguey, Desembarcodel Granma Buenavista, , Punta Frances, De la Juventud Cayosde Ana Maria, PuntaPederales
59	Bahamas	BHA			SIDS	Americas	Very High	Caribbean Sea	Conception Island, Exuma Cays, Inagua, Abaco, Peterson Cay, Little San Salvador (Little Island), Pelican Cays, Union Creek, Lucayan, Tilloo Cay,
60	Dominican Republic	DOM			SIDS	Americas	High		Marine Mammal, Jaragua, Del Este Montecristi, Parque Submarino la Caleta, Litoral Sur (Santo Domingol)
61	Haiti	HTI			SIDS	Americas	Low		Port-au- Prices and Les Arcadins Islands, De La Gonave
62	Jamaica	JAM			SIDS	Americas	High		Bogue, Middle Morant Cay, Montego Bay, Negril, Ocho Rios, Portland Bight
63	Saint Kitts and Nevis	KNA			SIDS	Americas	High		Southeast Peninsula
64	Grenada	GRD			SIDS	Americas	High		Molinere-Beausejour, Sandy Island Oyster Bed, Woburn Bay, Grand Anse
65	Antigua and Barbuda	ATG			SIDS	Americas	High		Redona Island
66	Dominica	DMA			SIDS	Americas	High		Cabrits, Soufriere, Scott's Head

No.	Country / Territory	ISO 3 country code	FAO Fishing Area	Marine / Inland Basin	LC Grouping	WHO Region	HDI Level	Coral reef location (marine basin)	Marine Protected Areas (MPAs) with Coral Reefs
67	Trinidad and Tobago	TTO			SIDS	Americas	Very High		Buccoo Reef, Little Tobago
68	Barbados	BRB			SIDS	Americas	High		Barbados
69	Saint Lucia	LCA			SIDS	Americas	High		Rodney and Vigie Bay Artificial Reefs, Anse Cochon, Anse Mamin Reef, Grande Caille and Rachette, Anse I'Lvrogne, Malgretoute and Jalousie, Anse des Piton, Gros and Petit Piton, Anse Chastanet, Anse Galet, Anse la Verdure, Maria Islet Reef, Caesar and Mathurin, Artificial Reef at Moule a Chique
70	Saint Vincent and the Grenadines	VCT			SIDS	Americas	High		South Coast, Bequita, Isle a Quatre, Mustique, Canouan, Tobago Cays-Mayreau, Union-Palm,Island, Petit St. Vincent
71	Aruba	ABW			SIDS	Americas	NA		Het Spaans Lagoon, Mangel Halto, Boca Catalin, Santana Reef
72	Curaçao	CUW			SIDS	Americas	NA		Christofel, Weillemstad, Curacao,
73	Belize	BLZ			SIDS	Americas	Medium		Caay, Belize, Honduras, Glovers Reef, Blue Hole, Bacalar Chico, Gladden Spit, Man-o-War Cay, Half Moon Caye, Hol Chan, Sapodilla Cayes,
74	Cayman Islands	CYM			SIDS	Americas	NA		Bloody Bay, Bowse Bluff, Cayman Dive Lodge, Bats Cave Beach, Coral Isle Club, West Bay, Dick Sessions Bay, Frank Sound, Head of Barkers, Jennifer Bay, Little Sound, Mary Bay, Spot Bay, North and South Sound, Preston Bay, Radio Mast, Victoria House,
75	Bermuda	BMU			SIDS	Americas	NA		North Rock, Aquarium, Blue Hole, Mills Breaker, South West Breaker, Smugglers Notch, Bad Caves, Killa Puffa, Cathedral and Basilica, Hangover Hole, Parrot Mission, Table Top, Bad Lands, Kevin Reef, Three Sisters, Tarpon, Hole, Ben Bender, Watch Hill Park Reef, Sic-O-Big-c, Walsingham
76	Turks and Caicos Islands	TCA			SIDS	Americas	NA		Fort George Land and Sea, West Caicos Marine, French - Bush and Seal Cays, Grand Turk Cays-Land and Sea, North-Middle and East Caicos Islands
77	Guyana	GUY			SIDS	Americas	High		-
78	Bolivarian Republic of Venezuela	VEN			South and Central America	Americas	Medium		Archipelago Los Roques, Mochima, Morrocoy, San Esteban
79	Suriname	SUR			South and Central America	Americas	High		-
80	Nicaragua	NIC	31	West-Central Atlantic	South and Central America	Americas	Medium	Caribbean Sea	Cayos Miskitos
			77	East-Central Pacific				-	-
81	Honduras	HND	31	West-Central Atlantic	South and Central America	Americas	Medium	Caribbean Sea	Ragged Cay, Laguna de Guaymoreto, Jeanette Kawas, Refugio de Vida,
			77	East-Central Pacific				East Coast of Pacific Ocean	

No.	Country / Territory	ISO 3 country code	FAO Fishing Area	Marine / Inland Basin	LC Grouping	WHO Region	HDI Level	Coral reef location (marine basin)	Marine Protected Areas (MPAs) with Coral Reefs
									Silvestre Punta Izopo, Islasdel Cisne, Punta Isopo, Bahia de Chismuyo, La Alemania El Quebrachal, Guameru, Teonostal, Guapinol, Montecristo, Parque Nacional Jeanette Kawas, Cayos Cochinos, El Jicarito, Las Iguanas,
82	Mexico	MEX	31	West-Central Atlantic	South and Central America	Americas	High	Caribbean Sea, Gulf of Mexico	Costa Occidental de Isla Mujeres, Baco Chincharro, , Los Arcos, Arrecife Alacranes, Fondo Cabo San Lucas, Xcalak, Isla Mujeres Punta Cancun Punta Nizuc, Isla Cantoy, Sian Kaan Archipelago Ravillagigedo, La Sian Kaan, Arrecifes de Sian Kaan, Blanquilla, Cabo Polmo, Sistema Arrectifal Veracruzano Arrecifes de Puerto Morelos, Laguna de Chankannab, Bahia Loreto, Arrecifes de Cazumel, Costa Occidental Isla Cozumel, Islas del Golfo California
			77	East-Central Pacific				East Coast of Pacific Ocean, Gulf of California	
83	Costa Rica	CRI	31	West-Central Atlantic	South and Central America	Americas	Very High	Caribbean Sea	Cocos Island, Area de Conservation Guanacaste, Cabo Blanco, Cahuita, Gandoca-Manzallino
			77	East-Central Pacific				East Coast of Pacific Ocean	Isla del Cano, Isla del Coco, Manuel Antonio
84	Panama	PAN	31	West-Central Atlantic	South and Central America	Americas	Very High	Caribbean Sea	Comarca Kuna,Yala (San Blas), Isla Bastimentos, Portobelo,
			77	East-Central Pacific				Gulf of Panama	Punta Patino
85	Colombia	COL	31	West-Central Atlantic	South and Central America	Americas	High	Caribbean Sea	Corales del Rosario. Sierra Nevada de Santa Marta, Lagoon, Tayrona, Old Providence Mc Bean
			77	East-Central Pacific				Gulf of Panama	Ensenada de Utria
			87	South-East Pacific				East Coast of Pacific Ocean	Isla de Malpelo, Isla Gorgona
86	Mauritania	MRT	34	East-Central Atlantic	Africa	Africa	Medium	-	-
87	Senegal	SEN			Africa	Africa	Low	-	-
88	Gambia	GMB			Africa	Africa	Low	-	-
89	Guinea Bissau	GNB			Africa	Africa	Low	-	-
90	Guinea	GIN			Africa	Africa	Low	-	-
91	Sierra Leone	SLE			Africa	Africa	Low	-	-
92	Liberia	LBR			Africa	Africa	Low	-	-
93	Equatorial Guinea	GNQ			Africa	Africa	Medium	-	-
94	Côte d'Ivoire	CIV			Africa	Africa	Medium	-	-
95	Ghana	GHA			Africa	Africa	Medium	-	-
96	Togo	TGO			Africa	Africa	Low	-	-
97	Benin	BEN			Africa	Africa	Low	-	-
98	Nigeria	NGA			Africa	Africa	Low	-	-
99	Cameron	CMR			Africa	Africa	Medium	-	-
100	Congo	COG			Africa	Africa	Medium	-	-
101	Democratic Republic of Congo	COD			Africa	Africa	Low	-	-
102	Gabon	GAB			Africa	Africa	High	-	-

No.	Country / Territory	ISO 3 country code	FAO Fishing Area	Marine / Inland Basin	LC Grouping	WHO Region	HDI Level	Coral reef location (marine basin)	Marine Protected Areas (MPAs) with Coral Reefs
103	Cabo Verde	CPV			SIDS	Africa	Medium	-	-
104	Sao Tome and Principe	STP			SIDS	Africa	Medium	-	-
105	Morocco	MAR	34	East-Central Atlantic	Africa	Eastern Mediterranean	Medium	East Coast of Atlantic Ocean	Cold-water coral mounds (Moroccan Atlantic Continental Margin)
			37	Mediterranean Sea, Black Sea				-	-
106	Bulgaria	BGR	37	Mediterranean Sea, Black Sea	Europe	Europe	High	-	-
107	Romania	ROU			Europe	Europe	Very High	-	-
108	Ukraine	UKR			Europe	Europe	High	-	-
109	Italy	ITA			Europe	Europe	Very High	Mediterranean Sea, Tyrrhenian, Adriatic, and Ionian Seas	Sensitive deep-sea coral reefs
110	Albania	ALB			Europe	Europe	High	-	-
111	Bosnia and Herzegovina	BIH			Europe	Europe	High	-	-
112	Croatia	HRV			Europe	Europe	Very High	-	-
113	Greece	GRC			Europe	Europe	Very High	-	-
114	Malta	MLT			Europe	Europe	Very High	-	-
115	Turkey	TUR			Asia	Europe	Very High	-	-
116	Georgia	GEO			Asia	Europe	Very High	-	-
117	Cyprus	CYP			Asia	Europe	Very High	East of Mediterranean Sea	Sensitive deep-sea coral reefs
118	Syrian Arab Republic	SYR			Asia	Eastern Mediterranean	Medium	-	-
119	Tunisia	TUN			Africa	Eastern Mediterranean	High	-	-
120	Libya	LBY			Africa	Eastern Mediterranean	High	-	-
121	Algeria	DZA			Africa	Africa	High	-	-
122	Israel	ISR	37	Mediterranean Sea, Black Sea	Asia	Europe	Very High	-	-
			51	West Indian				Red Sea	Marine Reserve
123	Uruguay	URY	41	South-West Atlantic	South and Central America	Americas	Very High	-	-
124	Argentina	ARG			South and Central America	Americas	Very High	-	-
125	Angola	AGO	47	South-East Atlantic	Africa	Africa	Medium	-	-
126	Namibia	NAM			Africa	Africa	Medium	-	-
127	South Africa	ZAF	47	South-East Atlantic	Africa	Africa	High	South East Coast of Atlantic Ocean, West Coast of Indian Ocean	Browns Bank Corals, Port Elizabeth Corals, St. Lucia
			51	West Indian				-	-
128	Egypt	EGY	37	Mediterranean Sea, Black Sea	Africa	Eastern Mediterranean	High	-	-
			51	West Indian				Red Sea, Gulf of Aqaba	Ras Mohammed, Nabq, Abu Galum, Elba, Safaga Island, Giftun Islands and Straits of Gubal, Sharm al-Lulu, Dedalus Island, Zabareged Island, Brother Islands, Al-Qusair Reef Complex
129	Bangladesh	BGD	51	West Indian	Asia	South-East Asia	Medium	Bay of Bengal	Island of St. Martin’s

No.	Country / Territory	ISO 3 country code	FAO Fishing Area	Marine / Inland Basin	LC Grouping	WHO Region	HDI Level	Coral reef location (marine basin)	Marine Protected Areas (MPAs) with Coral Reefs
130	Iraq	IRQ			Asia	Eastern Mediterranean	Medium	-	-
131	Kuwait	KWT			Asia	Eastern Mediterranean	Very High	Persian Gulf	Kubbar, Qaro Island, Um Al-Maradem Islands
132	United Arab Emirates	ARE			Asia	Eastern Mediterranean	Very High	Persian Gulf	Rul Dibba, Dadna, Al Aqa, Al Bidiyah, Al Yasat, Marawaah
133	Bahrain	BHR			Asia	Eastern Mediterranean	Very High	Persian Gulf	The Northern 'Hayrat' and Reef Bul Thamah
134	Qatar	QAT			Asia	Eastern Mediterranean	Very High	Persian Gulf	Khor Al Oudeid, Halul Island, Fasht al Dibal
135	Saudi Arabia	SAU			Asia	Eastern Mediterranean	Very High	Persian Gulf, Red Sea	Asir, Dawat Ad-Dafl ,Dawat al- Musallamiyah, Farasan and Umm al-Qamari Islands
136	Oman	OMN			Asia	Eastern Mediterranean	Very High	Arabian Sea, Gulf of Oman	Daymaniyat Islands
137	Jordan	JOR			Asia	Eastern Mediterranean	High	Gulf of Aqaba	Aqaba Marine Park, Aqaba Marine Protected Area
138	Djibouti	DJI			Africa	Eastern Mediterranean	Low	Gulf of Aden	Maskali Sud, Musha
139	Eritrea	ERI			Africa	Africa	Low	Red Sea, Gulf of Zula	Dahlak, Desie Island, Madot Island
140	Kenya	KEN			Africa	Africa	Medium	West Coast of Indian Ocean	Diani, Kisite, Kiunga, Malindi, Malindi-Watamu, Mombasa, Mpunguti, Watamu
141	Mozambique	MOZ			Africa	Africa	Low	Mozambique Channel	Bazaruto, Ilhas de Inhaca e dos Portugueses
142	Comoros	COM			SIDS	Africa	Medium	North of Mozambique Channel	Moheli
143	Seychelles	SYC			SIDS	Africa	High	West of Indian Ocean	Seychelles Archipelago<br>(made up of 115 islands, covering 410,000 km2)
144	Mauritius	MUS			SIDS	Africa	Very High	West of Indian Ocean	Grand Port-Mahebourg, Trou d Eau, Douce, Balaclava, Black River, Flacq, Port Louis, Riviere du Rampart – Poudre d Or
145	United Republic of Tanzania	TZA			Africa	Africa	Low	West of Indian Ocean	Bongoyo, Mnazi Bay-Ruvuma Estuary, Menai Bay, Dar es Salaam, Tanga Coelacanth, Tanga, Islands: Mbudya, Mafia, Ulenge, Kirui, Fungu Yasini, Maziwe, Kwalekuni, Mwewe, Mbarakuni, Nyororo, Shungi Mbili, Pangavini,
146	Maldives	MDV			SIDS	South-East Asia	High	North of Indian Ocean, South of Arabian Sea	22 Coral Atolls, 1200 Coralline Islands, 25 Dive Sites, (6900 km^2 Surface-Level Atolls)
147	Myanmar	MMR	57	East Indian	Asia	South-East Asia	Medium	Bay of Bengal	Lampi, Moscos Island
148	Sri Lanka	LKA			Asia	South-East Asia	High	Bay of Bengal, Palk Strait, Gulf of Mannar	Bar Reef Marine, Hikkaduwa Marine
149	Thailand	THA	57<br>71	East Indian<br>West-Central Pacific	Asia	South-East Asia	Very High	Andaman Sea<br>Gulf of Thailand	Khao Sam Roi Yot, Ao Phang Mai, Hat Chao, Mu Ko Surin,<br>Hat Nopharat Thara – Mu Ko Phi Phi, Tarutao, Mu Ko Petra,<br>Mu Ko Similan, Mu Ko Chang, Mu Ko Libong, Mu Ko Lanta,<br>Mu Ko Ang Thong, Sirinath, Mai, Khao Laem Ya – Mu Ko Samet,
150	Indonesia	IDN	57<br>71	East Indian<br>West-Central Pacific	Asia	South-East Asia	High	Natuna Sea, Sunda Strait, Bali Sea, Java Sea, South China Sea,<br>Karimata Strait, Sibrut Strait<br>Tomini Bay, Bony Bay, Makasar Strait, Sawu Sea, Halmahera Sea, Triton Bay, Flores Sea, Mulluca Sea, Seram Sea, Sulawesi Sea (Celebes), Timor Sea, Banda Sea, Arafura Sea, Sulu Sea,	Penya (Turtle Island), Perhentian Bako, Chebeh, Tunka Abdul Rahman, Penang, Rusukan Besar, Tiga,Goal, Harimau, Hujung, Jahat, Kaca, Kapas, Nyireh, Payar, Rawa, Redang, Perhentian Kecil, Kuraman, Labes, Lang Tengah, Lembu, Lima, Ekor Sipadan, Pemanggil, Aur, Besar, Tinggi Segantang,Tebu, Besar, Mensirip, Mentinggi, Tioman, TokongBahara, Tulai, Rusukan Kecil, Sri Buat, Sembilang, Sepoi, Sibu, Sibu Hojung, Susu Dara, Tengah Tenggol,
151	Malaysia	MYS	57	East Indian	Asia	Western Pacific	Very High	Andaman Sea, Bali Sea, Java Sea, Karimata Strait, Strait of Malacca,	Over 17000 islands with 60 Coral Reef sites as 51 MPAs in Peninsular Malaysia and East Malaysia

No.	Country / Territory	ISO 3 country code	FAO Fishing Area	Marine / Inland Basin	LC Grouping	WHO Region	HDI Level	Coral reef location (marine basin)	Marine Protected Areas (MPAs) with Coral Reefs
			71	West-Central Pacific				South China Sea, Singapore Strait, Gulf of Thailand, Makasar Strait, Natuna Sea, Sulu Sea, Sulawesi Sea	(51020 km^2 of Reef area)
152	Australia	AUS	57	East Indian	Oceania	Western Pacific	Very High	Shark Bay	Lord Howe Island, Christmas and Solitary Islands, Cocos Islands, South West Cobourg Peninsula, Ashmore Reef, Cobourg, Coringa-Harold, Mermaid Reef, Shark Bay, Solitary Island, Rowley Shoals, Coral Sea, Lihou Reef, Yongala, Shoal Water and Corio Bays, Ningaloo, Emden, Moreton Bay, Lord Howe Island, Pulu Keeling, Elizabeth and Middleton Reefs, Great Barrier Reef
			71	West-Central Pacific				Timor Sea, Arafura Sea, Gulf of Carpentaria	
			81	South-West Pacific				Papua Gulf, Torres Strait, Coral Sea, Tasman Sea	
153	Japan	JPN	61	North-West Pacific	Asia	Western Pacific	Very High	East China Sea	Kasari Hanto Higashi Kaigan, Kirishima–Yaku, Nichinan Kaigan, Ogasawara, Okinawa Kaigan, Genkai, Iriomote, Kamae, Kametoku, Yoronto, Sakiyama-Wan, Sakurajima, Sata Misaki, Saikai, Okinawa, Setouchi, Shimobishi, Surikozaki, Tokashiki, Okinawa Senseki, Zamami, Yoshino-Kumano, Kiyanguchi, Maibishi, Nichinan,
154	Vietnam	VNM			Asia	Western Pacific	High	Gulf of Thailand, Gulf of Tonkin South China Sea,	Cat Ba, Con Dao
155	Cambodia	KHM			Asia	Western Pacific	Medium	-	-
156	Republic of Korea	KOR			Asia	Western Pacific	Very High	-	-
157	Papua New Guinea	PNG			SIDS	Western Pacific	Medium	Bismarck Sea, Coral Sea, Gulf of Papua, Arafura Sea, Torres Strait, Solomon Sea	Bagiai, Baniara Island, Crown Island, Kamiali, Horseshoe Reef, Kimbe Bay, Long Island, Maza, Nanuk Island, Ndrolowa, Pirung, Sawataetae, Simbine Coast, Sinub Island, Talele Island
158	Solomon Islands	SLB			SIDS	Western Pacific	Medium	Solomon Sea, Coral Sea	Arnavon, East Rennell
159	Timor-Leste	TLS			SIDS	South-East Asia	Medium	Flores Sea, Timor Sea	Pulau Besar
160	Philippines	PHL			Asia	Western Pacific	Medium	Sulawesi Sea, Bohol Sea, China Sea, Samar Sea, Sibuyan Sea, Sulu Sea, Philippine Sea, Panay Gulf,	Northern Sierra Madre, Turtle Islands, Puerto Galera, Tubbataha Reefs, Batanes, El Nido, Palawan, Puerto Pricesa, (60 Coral Reef sites and 50 MPAs)
161	Palau	PLW	71	West-Central Pacific	SIDS	Western Pacific	High	North-West Pacific Ocean	Ngerukewid Islands, Negerumekaol Grouper, Ngaremeduu Bay, Ngeruangel, Ngiwal State, Ngemelis Islands, Ngermach Bkulachelid
162	Vanuatu	VUT			SIDS	Western Pacific	Medium	South-Central Pacific Ocean	Aore, BucaroAore, Naomebaravu, Malo, President Coolidge and Million Dollar Point
163	Fiji	FJI			SIDS	Western Pacific	High	Koro Sea	Viti Levu, Vanua Levu, Beqa Barrier Reef, Kadavu, Yasawa
164	Nauru	NRU			SIDS	Western Pacific	NA	-	-
165	Federated States of Micronesia	FSM			SIDS	Western Pacific	Medium	-	-
166	Marshal Islands	MHL			SIDS	Western Pacific	Medium	-	-
167	Tonga	TON			SIDS	Western Pacific	High	South-Central Pacific Ocean	Ha-atafu, Hakaumama o Reef, Malinoa Island, Ha-amongaatarilithon, Monuafe Island, Nui Hopo Hoponga, Pangaimotu Reef, Eua, Fangauta, Fanga Kakau,
168	Samoa	WSM	77	East-Central Pacific	SIDS	Western Pacific	High	South-Central Pacific Ocean	Pago Pago Harbor, Shipwreck, Rose Atoll, Palolo Deep Marine, Tafua Rainforest Reserve
169	Guatemala	GTM			South and Central America	Americas	Medium	-	-

No.	Country / Territory	ISO 3 country code	FAO Fishing Area	Marine / Inland Basin	LC Grouping	WHO Region	HDI Level	Coral reef location (marine basin)	Marine Protected Areas (MPAs) with Coral Reefs
170	El Salvador	SLV			South and Central America	Americas	Medium	-	-
171	Tuvalu	TUV			SIDS	Western Pacific	Medium	South-Central Pacific Ocean	Funafuti Lagoon, Nui; Vaitupu, Funafuti, Nukulaelae
172	Kiribati	KIR			SIDS	Western Pacific	Medium	South-Central Pacific Ocean	Ra'ui, Rarotonga
173	Cook Islands	COK			SIDS	Western Pacific	NA	-	-
174	New Zealand	NZL	81	South-West Pacific,	Oceania	Western Pacific	Very High	-	-
175	Ecuador	ECU	87	South-East Pacific	South and Central America	Americas	High	-	-
176	Peru	PER			South and Central America	Americas	High	-	-
177	Chile	CHL			South and Central America	Americas	Very High	-	-

Marine Protected Areas (MPAs) including: Biological Reserve (BiR), Biosphere Reserve - National (BR-N), Bird Sanctuary (BS), Conservation Area (CA), Coral Reef Ecosystem Reserve (CRER), Dive Site (DS), Environmental Zone (EnvZ), Fauna and Flora Protection Area (FFPA), Fauna and Flora Sanctuary (FFS), Fish Nursery Reserve (FNR), Fisheries Management Area (FMA), Fishing Reserve (FiR), Game Reserve (GS), Hunting Reserve (HR), Indigenous Commarc (IndCo), Integral Reserve (IR), Littoral Conservation Area (LtCA), Marine Conservation District (MarCD), Marine National Park (NatP / NP), Marine National Reserve (MNaR), Managed Resources Protected Area (MRPA), Marine Lifr Conservation District (MLCD), Marine Park (MP), Marine Reserve / Tourist Zone (MR / TZ), Marine Resources Reserve (MRR), Marine Sanctuary (S), Municipal Marine Reserve (MuMR), Municipal Park (MuP), National Marine Sanctuary (NaMS), National Park (NP), National Wildlife Refuge (NWR), Nature Conservation Park (NCA), Nature Reserve (NR - RNat), Natural Monument (NM), Natural National Park (NatNP), Non Hunting Area (NHA), Other Area (ETC), Park (P), Preserve (Pr.), Protected Coatalline (PCo), State Park (SP), Private Reserve (PrivR), Prohibited Fishing Area (PFA), Protected Area (PA), Protected Landasecape / Seascape (PLS), Provincial Park (PP), Quasi National Park (QNP), Ramsar Site, Recreation Park (RP), Recreation Reserve (RecR), Repienisshement Zone (RpZ), Reserve (R), Resticted Area (RestA), Sanctuary (S), Spawing Area (SpnA), Special Fauna Reserve (SpFR),Special Nature Reserve (SpNR), Special Reserve (SpR), State Recreation Area (SRA), State Wildlife Sanctuary (SWS), Strict Nature Reserve (SNR), UNESCO Biosphere Reserve, Wildlife Preserve (WPres), Wildlife Refuge (WR), World Heritage Site.

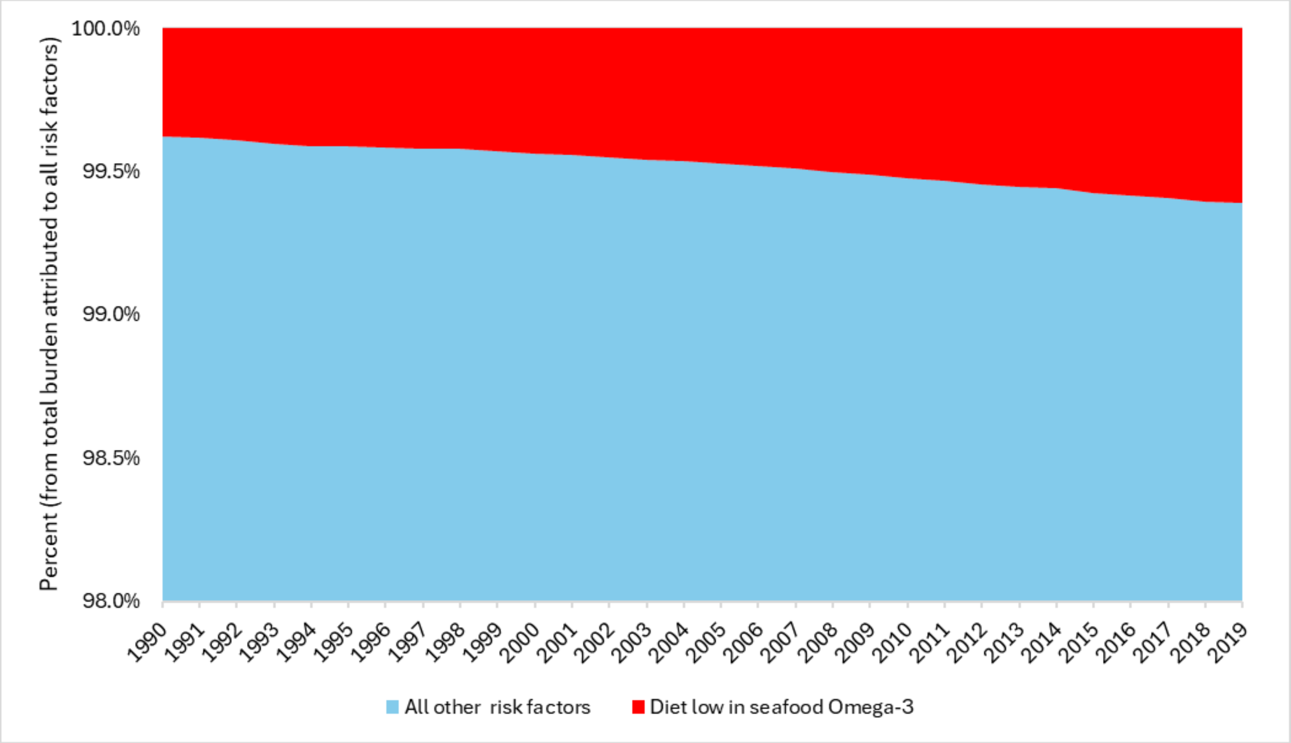

Figure 117: Percentage of burden of disease attributed to diet low in omega-3 fatty acids to the total burden attributed to all risk factors (1990–2019). Source: Global Burden of Disease 2019 study: https://vizhub.healthdata.org/gbd-results/

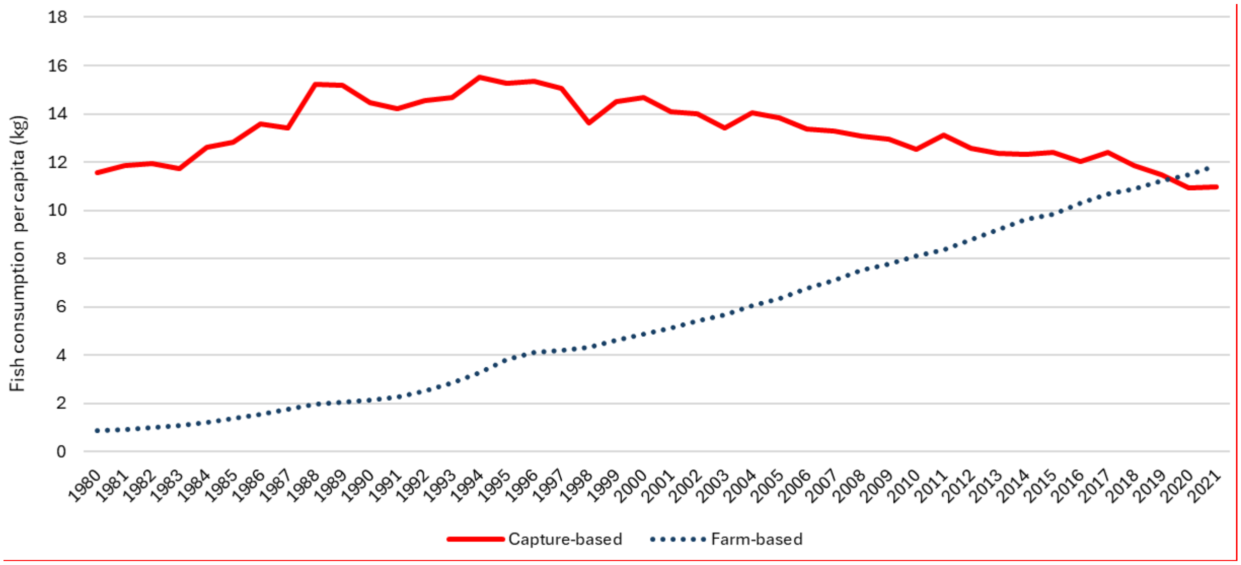

Figure 118: Population-weighted average fish consumption per capita in 177 investigated countries/territories, separated by the origin of fish (capture-based and farm-based) from 1980 to 2021.

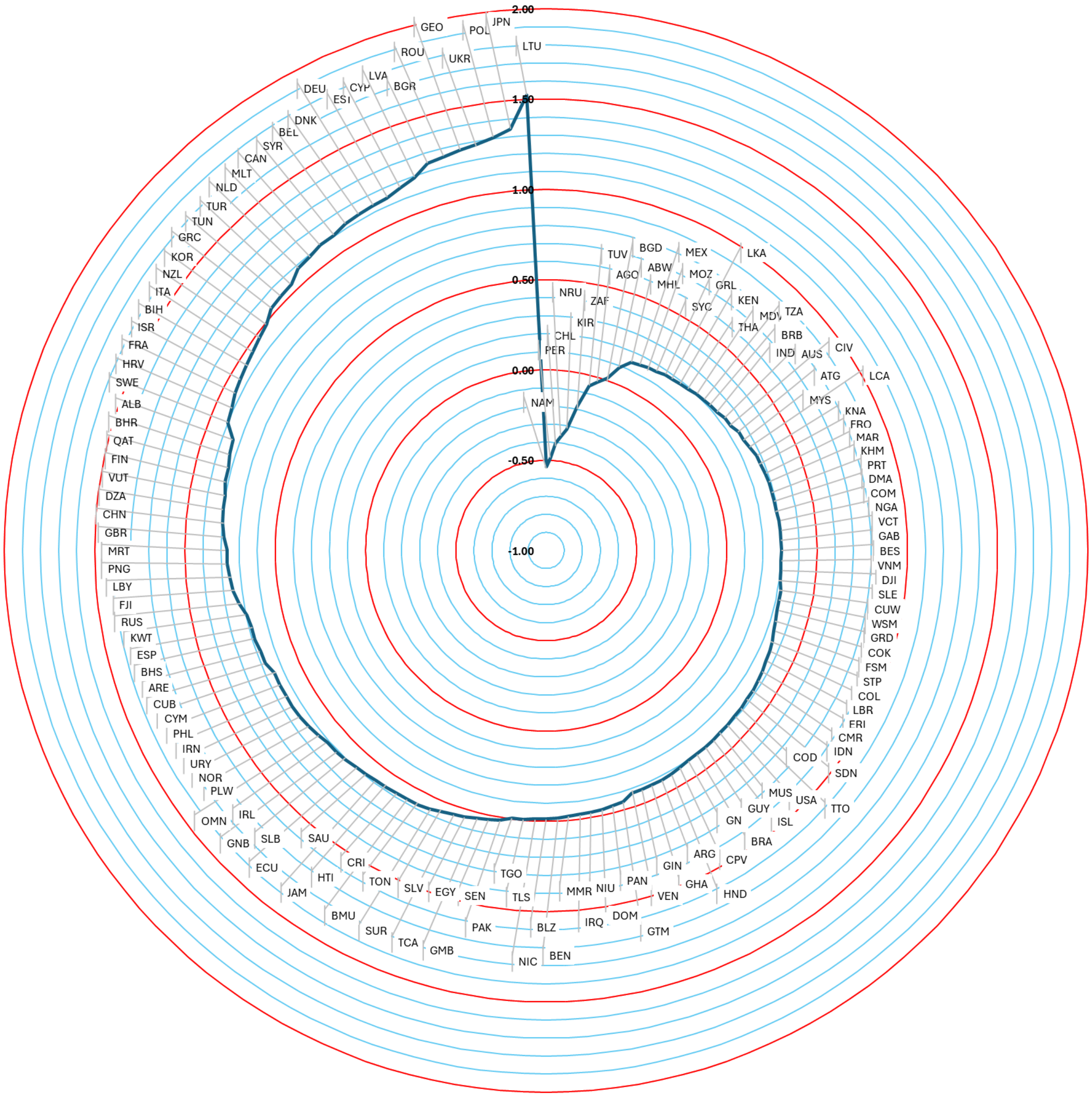

Figure 119: Changes in 3-year moving average sea surface temperature (°C) for the coastal waters of the investigated 148 countries/territories: 2021–2023 compared to the 1981-2010 baseline. Source: https://cds.climate.copernicus.eu/cdsapp#!/dataset/reanalysis-oras5?tab=overview

Table 24: Sea Surface Temperature (SST) in 1980 and 2023, projected SST based on SSP1-2.6 and SSP3-7.0 compared to the reference period (1981-2010) for the coastal waters, by the Lancet Countdown (LC) grouping, WHO regions and Human Development Index (HDI) levels.

	**Weights: Number of countries/territories (Total: 148)**						**Weights: Number of data points (Total: 789,624 for measured SSTs)**					
**Locations**	**Measured SST (◦C)**				**Mean projected SST (◦C)**		**Mean measured SST (◦C)**				**Mean projected SST (◦C)**	
	**1981-2010 (Reference)**	**1980**	**2023**	**Difference***	**2041-2060 SSP1-2.6**	**2041-2060 SSP3-7.0**	**1981-2010 (Reference)**	**1980**	**2023**	**Difference***	**2041-2060 SSP1-2.6**	**2041-2060 SSP3-7.0**
LC grouping												
Africa	25.15	24.89	25.74	0.366	26.43	26.54	23.67	23.40	24.18	0.301	24.98	25.08
Asia	24.88	24.83	25.63	0.648	25.21	25.25	25.49	25.41	26.12	0.592	26.02	26.10
Europe	11.84	11.23	12.90	0.912	13.11	12.97	8.52	8.08	9.41	0.758	10.11	10.01
Northern America	7.30	7.04	8.17	0.749	9.65	9.67	7.35	7.08	8.21	0.745	9.69	9.71
Oceania	18.80	18.63	19.34	0.605	18.79	19.13	20.75	20.64	21.08	0.414	21.03	21.33
SIDS	27.60	27.59	28.17	0.392	27.61	27.79	27.96	27.92	28.55	0.504	28.01	28.19
South and Central America	24.34	24.40	25.08	0.336	25.53	25.66	22.11	22.13	22.62	0.189	23.42	23.56
WHO regions												
Africa	25.82	25.60	26.37	0.329	27.17	27.32	24.65	24.44	25.03	0.209	26.16	26.33
Americas	25.11	25.14	25.82	0.406	25.69	25.83	17.50	17.42	18.19	0.451	18.97	19.06
Eastern Mediterranean	24.89	24.79	25.73	0.643	24.98	25.00	24.61	24.46	25.46	0.615	24.90	24.92
Europe	12.78	12.21	13.88	0.935	14.03	13.91	9.25	8.82	10.17	0.781	10.82	10.72
South-East Asia	28.55	28.63	28.77	0.269	28.80	28.95	28.76	28.75	28.98	0.331	29.13	29.32
Western Pacific	25.92	25.84	26.46	0.471	25.94	26.11	23.55	23.42	24.15	0.599	23.91	24.06
HDI classification												
Low	27.23	27.07	27.86	0.409	28.04	28.20	27.34	27.24	27.89	0.360	27.89	28.06
Medium	26.77	26.71	27.29	0.364	27.57	27.71	27.21	27.11	27.67	0.425	27.83	27.99
High	24.96	24.82	25.70	0.478	25.53	25.63	25.48	25.36	26.06	0.409	26.23	26.35
Very High	16.59	16.26	17.50	0.764	17.49	17.47	13.23	12.99	13.99	0.649	14.60	14.62
N/A	26.92	26.91	27.41	0.328	26.64	26.82	26.93	26.92	27.43	0.379	26.56	26.74
All (Global)	22.93	22.79	22.61	23.52	0.537	23.51	23.59	19.38	19.20	20.03	0.536	20.41

* The coastal SST average for 2021-23 compared to the 1981-2010 baseline.

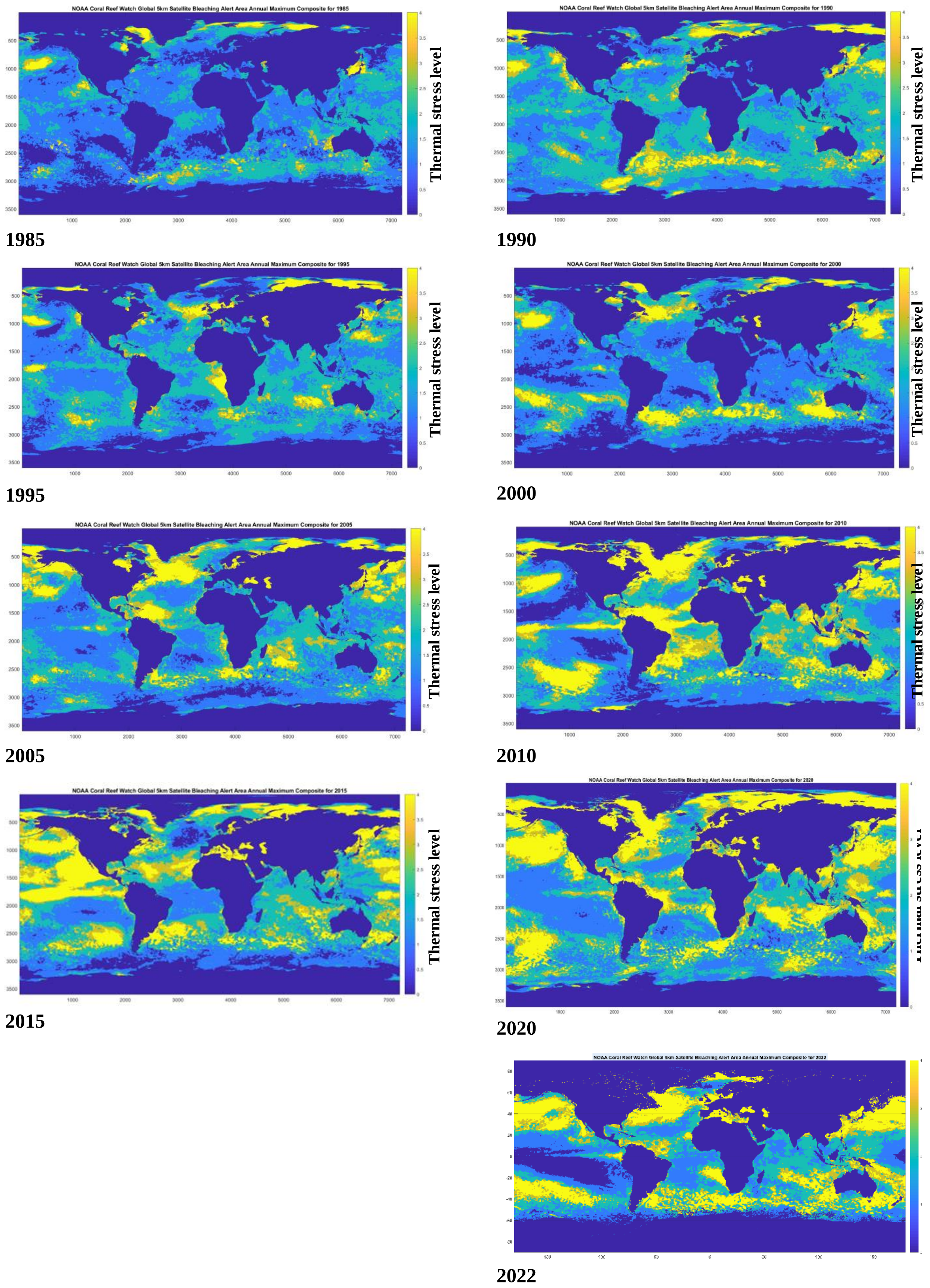

Figure 120: Comparing annual maximum Bleaching Alert Area caused by thermal stress in five-year intervals (1985–2022). Map resolution: 3600×7200 pixels, each pixel equals approx. 5-km. Source: NOAA Coral Reef Watch Global 5km Satellite Bleaching Alert Area Annual Maximum Composite Version 3.1.

Section 2: Adaptation, Planning, and Resilience for Health

Section Leads: Prof Joacim Rocklöv and Dr Marina Treskova
Research Fellow: Dr Yasna Palmeiro Silva

2.1: Assessment and planning of health adaptation

Indicator 2.1.1: national assessments of climate change impacts, vulnerability, and adaptation for health

Indicator Authors

Dr Diarmid Campbell-Lendrum

Methods

COP26 Health Programme

During the 26th United Nations Climate Change Conference (COP26), the COP26 Health Programme was promoted by the UK as the COP president to enable transformational change to protect the health of people and the planet. The initiatives under the COP26 Health Programme were not part of the formal negotiated outcome of COP26 but promoted at its margins, and include:

- Building climate resilient health systems
- Developing sustainable low carbon health systems
- Adaptation research for health
- The inclusion of health priorities in Nationally Determined Contributions
- Raising the voice of health professionals as advocates for stronger ambition on climate change

Two of the Programme's key initiatives aim to support countries in committing to strengthening climate resilient and sustainable low carbon health systems (Table 25).

Table 25: Commitments under the COP26 Health Programme

**Commitment 1: Climate resilient health systems**	**Commitment 2: Sustainable low carbon health systems**
• Commit to conduct climate change and health vulnerability and adaptation assessments (V&As) at population level and/or health care facility level by a stated target date<br>• Commit to develop a health National Adaptation Plan informed by the health V&A, which forms part of the National Adaptation Plan to be published by a stated target date	• High ambition/high emitters: Commit to set a target date by which to achieve health system net zero emissions (ideally by 2050)<br>• All countries: Commitment to deliver a baseline assessment of greenhouse gas emissions of the health system (including supply chains)<br>• All countries: Commit to develop an action plan or roadmap by a set date to develop a sustainable low carbon health system (including supply chains) which also considers human exposure to air

• Commit to use the V&A and HNAP to facilitate access to climate change funding for health (e.g., project proposals submitted to the Global Environmental Facility, Green Climate Fund, Adaptation Fund, or GCF Readiness programme).	pollution and the role the health sector can play in reducing exposure to air pollution through its activities and its actions.

Progress on the COP26 commitments is tracked through regular reporting to the WHO-led Alliance for Transformative Action on Climate Change and Health (ATACH), which also supports countries in the delivery and implementation of these commitments.[212] Further information on the ATACH community of practice can be found here: https://www.atachcommunity.com/

Eighty-two countries have committed to these initiatives and joined ATACH as of March 2024, increasing from 50 countries that had committed in November 2021 following COP26. An analysis of country self-reported data was conducted to assess the number of countries, territories, and areas 1) with commitments to build a climate resilient health system under the COP26 Health Programme, and 2) that have conducted climate change and health V&A assessments, informed under the ATACH or the 2021 WHO Health and Climate Change Global Survey.

2021 WHO Health and Climate Change Global Survey

The 2021 WHO Health and Climate Change Global Survey is a triennial and voluntary survey, which is sent to all WHO Member States and a small number of non-Member territories. The survey is completed by Ministry of Health focal points and often as part of a multistakeholder consultation. The last global survey was published in 2021. Of the 194 WHO Member States and further non-Member territories, 95 participated in the survey, providing representation from all six WHO regions. Countries, territories, and areas participating in the 2021 WHO Health and Climate Change Global Survey were: Argentina, Azerbaijan, Bahamas, Bahrain, Barbados, Belize, Benin, Bhutan, Bolivia (Plurinational Bolivia State of), Brazil, British Virgin Islands, Brunei Darussalam, Bulgaria, Cabo Verde, Cambodia, Cameroon, Canada, China, Colombia, Comoros, Costa Rica, Côte d'Ivoire, Croatia, Cuba, Cyprus, Czech Republic, Dominica, Dominican Republic, Egypt, El Salvador, Eritrea, Estonia, Ethiopia, Germany, Ghana, Grenada, Guatemala, Guinea, Guyana, Haiti, India, Iran (Islamic Republic of), Israel, Italy, Jamaica, Jordan, Kazakhstan, Kenya, Kyrgyzstan, Lebanon, Lithuania, Madagascar, Malawi, Marshall Islands, Micronesia (Federated States of), Mozambique, Netherlands, Nicaragua, Nigeria, North Macedonia, Occupied Palestinian Territory, including East Jerusalem, Oman, Palau, Papua New Guinea, Paraguay, Peru, Philippines, Poland, Portugal, Republic of Moldova, Rwanda, Saint Kitts and Nevis, Saint Lucia, San Marino, Sao Tome And Principe, Saudi Arabia, Serbia, Seychelles, Sierra Leone, Slovakia, South Africa, Sri Lanka, Suriname, Sweden, Thailand, Togo, Trinidad and Tobago, Turkmenistan, United Republic of Tanzania, United States of America, Uruguay, Vanuatu, Yemen, Zambia, Zimbabwe.[213]

The WHO survey asks countries whether they have conducted a climate change and health V&A assessment, defined as *a process and a tool that allows countries to evaluate which populations are most vulnerable to different kinds of health effects from climate change, to identify weaknesses in the systems that should protect them, and to specify interventions to respond. Assessments can also improve evidence and understanding of the linkages between climate and health within the assessment area, serve as a baseline analysis against which changes in disease risk and protective measures can be monitored, provide the opportunity for building capacity, and strengthen the case for investment in health protection.*

The 2021 global survey results provided the baseline data for the COP26 commitments with updates on the progress until 2023 coming from regional and country reporting through the ATACH. Further information on the 2021 WHO Health and Climate Change Global Survey, its methodology, and the WHO UNFCCC Health and Climate Change Country Profile Project can be found at https://www.who.int/activities/monitoring-science-and-evidence-on-climate-change-and-health

Data validation

Validation of the 2021 WHO Health and Climate Change Global Survey data was undertaken in multiple steps. First, survey responses were reviewed for missing information or inconsistencies with follow-up questions directed to survey respondents. A summary of responses was shared with WHO regional focal points and key informants for review, comments, and validation. Source documents including national health strategies and plans, and climate change and health vulnerability and adaptation assessments were collected. A desktop review of these source documents was conducted to compare with survey results with follow-up to survey respondents to seek clarification or additional documentation. Findings were also cross referenced with existing external publications. Data detailing all the ministries, institutions, and national stakeholders that provided contributions to or review of the survey responses were collected in order to provide insight into the national consultation process of each survey submission. Of the 95 submissions, 69 surveys were completed in consultation with one to six different stakeholders, ministries, or institutions. Five consulted between 10 to 12 stakeholders, ministries, or institutions. Fifteen countries and areas did not consult with other entities or health programmes. Information was not available for the remaining six countries. Finally, all respondents reviewed and acknowledged the WHO data policy statement on the use and sharing of data collected by WHO in Member States outside the context of public health emergencies.

The 2023/24 ATACH Baseline was updated through a short questionnaire sent by WHO to all 82 ATACH countries in February 2024. A total of 79 countries responded. Information was not available for the remaining three countries.

Of note, due to the COVID-19 pandemic, the standard data collection procedures were modified to reduce reporting burden on countries that wished to participate in the global survey but that were facing human resource constraints due to pandemic response. In eight cases, WHO prepared pre-filled survey questionnaires with data provided by ministries of health in the previous 2018 survey cycle or using data the countries had published in the 2020/2021 WHO UNFCCC health and climate change country profile when available. These countries were requested to review, revise, and complete the hard copy questionnaires. These hard copy questionnaires were then entered into the online platform by WHO. The same data validation steps as described above were then followed. Additionally, a number of respondents requested an extension of the reporting period.

Data analysis

Data with complete information were considered in the analysis, which means that countries with no classification under HDI were excluded (i.e., Cook Islands, Democratic People's Republic of Korea, Monaco, Nauru, Niue, Somalia) only for that specific analysis.

Data

- Alliance for Transformative Action on Climate Change and Health (ATACH).[212,214]
- 2021 WHO Health and Climate Change Global Survey.[213]

- Human Development Index (HDI) at the United Nations Development Programme, Human Development Reports[215]
-

Caveats

The global survey sample is not a representative sample of all countries as this survey was voluntary; however, the inclusion of 95 countries in this survey, despite a global pandemic, demonstrates significant global coverage.

Data for this indicator represent the total number of countries that had made a commitment to the COP26 Health Programme as of December 2023. For the most recent list of commitments please see the ATACH website at: https://www.who.int/initiatives/alliance-for-transformative-action-on-climate-and-health/country-commitments

Future form of the indicator

The WHO Health and Climate Change Global Survey is a triennial survey and will continue to be the primary source of data to track this indicator.

The future evolution of this indicator will explore the use of evidence (particularly findings from vulnerability and adaptation assessments) to inform the development of strategies/plans and progress on level of implementation of strategies/plans. With more countries initiating the national adaptation plan (NAP) process, alignment of the health component with the overall NAP will also be more closely monitored and examined.

Interim information regarding the specific content of national strategies/plans, as explored in this qualitative analysis, may be re-assessed in the future.

Additional analysis

Eighty-two out of 195 WHO Member States and further non-Member territories have committed to ATACH as of March 2024 -Table *26*, Table *27*, and Table *28* show the distribution of WHO's members who have developed or are developing V&A assessments by different groupings.

Table 29: Distribution of WHO Member States and further non-Member territories committed to ATACH and whether they have developed V&A assessments by Human Development Index (HDI).

HDI	WHO's members that have	V&A ever developed up to 2023	V&A developed since 2020	New V&A under development in 2023

	committed to ATACH up to 2023	n (% of ATACH members)	n (% of ATACH members)	n (% of ATACH members)
Low	18 out of 32: 56%	12 (67%)	6 (33%)	2 (11%)
Medium	17 out of 44: 39%	13 (76%)	6 (35%)	1 (6%)
High	19 out of 49: 39%	10 (53%)	9 (47%)	5 (26%)
Very High	27 out of 64: 42%	15 (56%)	11 (41%)	3 (11%)
N/A	1 out of 6: 2%	0 (0%)	0 (0%)	0 (0%)
**TOTAL**	82 out of 195: 42%	50 (61%)	32 (39%)	11 (14%)

Table 30: Distribution of WHO Member States and further non-Member territories committed to ATACH and whether they have developed V&A assessments by WHO region.

**WHO region**	**WHO's members that have committed to ATACH up to 2023**	**V&A ever developed up to 2023** n (% of ATACH members)	**V&A developed since 2020** n (% of ATACH members)	**New V&A under development in 2023** n (% of ATACH members)
Africa	27 out of 47: 57%	20 (74%)	10 (37%)	1 (4%)
Americas	14 out of 35: 40%	8 (57%)	7 (50%)	2 (14%)
Eastern Mediterranean	14 out of 22: 64%	5 (36%)	3 (21%)	5 (36%)
Europe	13 out of 53: 25%	8 (62%)	6 (46%)	1 (8%)
South-East Asia	7 out of 11: 64%	5 (71%)	3 (43%)	1 (14%)
Western Pacific	7 out of 27: 26%	4 (57%)	3 (43%)	1 (14%)
**TOTAL**	82 out of 195: 42%	50 (61%)	32 (39%)	11 (14%)

Table 31: Distribution of WHO Member States and further non-Member territories committed to ATACH and whether they have developed V&A assessments by Lancet Countdown (LC) region.

**LC region**	**WHO's members that have committed to**	**V&A ever developed up to 2023**	**V&A developed since 2020** n (% of ATACH members)	**New V&A under development in 2023**

	ATACH up to 2023	n (% of ATACH members)		n (% of ATACH members)
Africa	28 out of 48: 58%	17 (61%)	9 (32%)	4 (14%)
Asia	22 out of 45: 49%	13 (59%)	9 (41%)	3 (14%)
Europe	10 out of 42: 24%	7 (70%)	5 (50%)	1 (10%)
Northern America	2 out of 2: 100%	2 (100%)	2 (100%)	0 (0%)
Oceania	2 out of 2: 100%	0 (0%)	0 (0%)	1 (50%)
SIDS	10 out of 39: 26%	7 (70%)	4 (40%)	0 (0%)
South and Central America	8 out of 17: 47%	4 (50%)	3 (38%)	2 (25%)
**TOTAL**	82 out of 195: 42%	50 (61%)	32 (39%)	11 (14%)

Indicator 2.1.2: national adaptation plans for health

Indicator Authors

Dr Diarmid Campbell-Lendrum

Methods

Progress on the COP26 commitments is tracked through regular reporting to the WHO-led Alliance for Transformative Action on Climate Change and Health (ATACH), which also supports countries in the delivery and implementation of these commitments.[212] Further information on the ATACH community of practice can be found here: https://www.atachcommunity.com/ .By November 21, 2021, approximate 50 countries committed to these initiatives. As of March 24, 2024, over 80 countries have committed to these initiatives and joined the ATACH.

An analysis of country self-reported data was conducted to assess the number of countries, territories, and areas 1) with commitments to build a climate resilient health system under the COP26 Health Programme, and 2) that have a health national adaptation plan (HNAP) or a national health and climate change plan/strategy in place, informed under the ATACH or the 2021 WHO Health and Climate Change Global Survey.

2021 WHO Health and Climate Change Global Survey

The 2021 WHO Health and Climate Change Global Survey is a triennial and voluntary survey, which is sent to all WHO Member States and a small number of non-Member territories. The survey is completed by Ministry of Health focal points and often as part of a multistakeholder consultation. The last global survey was published in 2021. Of the 194 WHO Member States and further non-Member territories, 95 participated in the survey, providing representation from all six WHO regions.

Countries, territories, and areas participating in the 2021 WHO Health and Climate Change Global Survey were: Argentina, Azerbaijan, Bahamas, Bahrain, Barbados, Belize, Benin, Bhutan, Bolivia (Plurinational Bolivia State of), Brazil, British Virgin Islands, Brunei Darussalam, Bulgaria, Cabo Verde, Cambodia, Cameroon, Canada, China, Colombia, Comoros, Costa Rica, Côte d'Ivoire, Croatia, Cuba, Cyprus, Czech Republic, Dominica, Dominican Republic, Egypt, El Salvador, Eritrea, Estonia, Ethiopia, Germany, Ghana, Grenada, Guatemala, Guinea, Guyana, Haiti, India, Iran (Islamic Republic of), Israel, Italy, Jamaica, Jordan, Kazakhstan, Kenya, Kyrgyzstan, Lebanon, Lithuania, Madagascar, Malawi, Marshall Islands, Micronesia (Federated States of), Mozambique, Netherlands, Nicaragua, Nigeria, North Macedonia, Occupied Palestinian Territory, including East Jerusalem, Oman, Palau, Papua New Guinea, Paraguay, Peru, Philippines, Poland, Portugal, Republic of Moldova, Rwanda, Saint Kitts and Nevis, Saint Lucia, San Marino, Sao Tome And Principe, Saudi Arabia, Serbia, Seychelles, Sierra Leone, Slovakia, South Africa, Sri Lanka, Suriname, Sweden, Thailand, Togo, Trinidad and Tobago, Turkmenistan, United Republic of Tanzania, United States of America, Uruguay, Vanuatu, Yemen, Zambia, Zimbabwe.[213]

The WHO survey asks countries whether they have conducted a climate change and health V&A assessment, defined as *a process and a tool that allows countries to evaluate which populations are most vulnerable to different kinds of health effects from climate change, to identify weaknesses in the systems that should protect them, and to specify interventions to respond. Assessments can also improve evidence and understanding of the linkages between climate and health within the assessment area, serve as a baseline analysis against which changes in disease risk and protective measures can be monitored, provide the opportunity for building capacity, and strengthen the case for investment in health protection.*

The 2021 global survey results provided the baseline data for the COP26 commitments with updates on the progress until 2023 coming from regional and country reporting through the ATACH.

Further information on the 2021 WHO Health and Climate Change Global Survey, its methodology, and the WHO UNFCCC Health and Climate Change Country Profile Project can be found at https://www.who.int/activities/monitoring-science-and-evidence-on-climate-change-and-health.

Data validation

Validation of the 2021 WHO Health and Climate Change Global Survey data was undertaken in multiple steps. First, survey responses were reviewed for missing information or inconsistencies with follow-up questions directed to survey respondents. A summary of responses was shared with WHO regional focal points and key informants for review, comments, and validation. Source documents including national health strategies and plans, and climate change and health vulnerability and adaptation assessments were collected. A desktop review of these source documents was conducted to compare with survey results with follow-up to survey respondents to seek clarification or additional documentation. Findings were also cross referenced with existing external publications. Data detailing all the ministries, institutions and national stakeholders that provided contributions to or review of the survey responses were collected in order to provide insight into the national consultation process of each survey submission. Of the 95 submissions, 69 surveys were completed in consultation with one to six different stakeholders, ministries, or institutions. Five consulted between 10 to 12 stakeholders, ministries, or institutions. 15 countries and areas did not consult with other entities or health programmes. Information was not available for the remaining six.

The 2023/24 ATACH Baseline was updated through a short questionnaire that WHO sent to all 82 ATACH countries in February 2024. A total of 79 countries responded. Information was not available for the remaining three countries.

Finally, all respondents reviewed and acknowledged the WHO data policy statement on the use and sharing of data collected by WHO in Member States outside the context of public health emergencies. Of note, due to the COVID-19 pandemic, the standard data collection procedures were modified to reduce reporting burden on countries that wished to participate in the global survey but that were facing human resource constraints due to pandemic response. In eight cases, WHO prepared pre-filled survey questionnaires with data provided by ministries of health in the previous 2018 survey cycle or using data the countries had published in the 2020/2021 WHO UNFCCC health and climate change country profile when available. These countries were requested to review, revise, and complete the hard copy questionnaires. These hard copy questionnaires were then entered into the online platform by WHO. The same data validation steps as described above were then followed. Additionally, a number of respondents requested an extension of the reporting period.

Data analysis
Data with complete information were considered in the analysis, which means that countries with no classification under HDI were excluded (i.e., Cook Islands, Democratic People's Republic of Korea, Monaco, Nauru, Niue, Somalia) only for that specific analysis.

Data

- Alliance for Transformative Action on Climate Change and Health (ATACH).[212,214]
- 2021 WHO Health and Climate Change Global Survey.[213]
- Human Development Index (HDI) at the United Nations Development Programme, Human Development Reports[215]

Caveats

The global survey sample is not a representative sample of all countries as this survey was voluntary; however, the inclusion of 95 countries in this survey, despite a global pandemic, demonstrates significant global coverage.

Data for this indicator represent the total number of countries that had made a commitment to the COP26 Health Programme as of January 2024. For the most recent list of commitments please see the ATACH website at: https://www.who.int/initiatives/alliance-for-transformative-action-on-climate-and-health/country-commitments

Future form of the indicator

The WHO Health and Climate Change Global Survey is a triennial survey and will continue to be the primary source of data to track this indicator.

The future evolution of this indicator will explore the use of evidence (particularly findings from vulnerability and adaptation assessments) to inform the development of strategies/plans and progress on level of implementation of strategies/plans. With more countries initiating the national adaptation

plan (NAP) process, alignment of the health component with the overall NAP will also be more closely monitored and examined.

Interim information regarding the specific content of national strategies/plans, as explored in this qualitative analysis, may be re-assessed in the future.

Additional analysis

Eighty-two out of 195 WHO Member States and further non-Member territories have committed to ATACH as of March 2024. Table *32*, Table *33*, Table *34* show the distribution of WHO's members whether they have ever developed or are developing HNAPs by different groups.

Table 35: Distribution of WHO Member States and further non-Member territories committed to ATACH and whether they have developed a HNAP by Human Development Index (HDI).

**HDI**	**WHO's members that have committed to ATACH up to 2023**	**HNAP ever developed up to 2023** n (% of ATACH members)	**HNAP developed since 2020** n (% of ATACH members)	**New HNAP under development in 2023** n (% of ATACH members)
Low	18 out of 32: 56%	11 (61%)	5 (28%)	1 (6%)
Medium	17 out of 44: 39%	8 (47%)	5 (29%)	4 (24%)
High	19 out of 49: 39%	10 (55%)	4 (22%)	4 (22%)
Very High	27 out of 64: 42%	14 (52%)	9 (33%)	5 (19%)
N/A	1 out of 6: 2%	0 (0%)	0 (0%)	0 (0%)
**TOTAL**	82 out of 195: 42%	43 (52%)	23 (28%)	14 (17%)

Table 36: Distribution of WHO Member States and further non-Member territories committed to ATACH and whether they have developed a HNAP by WHO region.

**WHO region**	**WHO's members that have committed to ATACH up to 2023**	**HNAP ever developed up to 2023** n (% of ATACH members)	**HNAP developed since 2020** n (% of ATACH members)	**New HNAP under development in 2023** n (% of ATACH members)
Africa	27 out of 47: 57%	16 (59%)	7 (26%)	2 (7%)
Americas	14 out of 35: 40%	9 (64%)	6 (43%)	3 (21%)
Eastern Mediterranean	14 out of 22: 64%	3 (23%)	1 (8%)	3 (23%)
Europe	13 out of 53: 25%	7 (54%)	4 (31%)	1 (8%)
South-East Asia	7 out of 11: 64%	5 (71%)	3 (31%)	2 (29%)
Western Pacific	7 out of 27: 26%	3 (43%)	2 (29%)	3 (43%)
**TOTAL**	82 out of 195: 42%	43 (52%)	23 (28%)	14 (17%)

Table 37: Distribution of WHO Member States and further non-Member territories committed to ATACH and whether they have developed a HNAP by Lancet Countdown (LC) region.

**LC region**	**WHO's members that have committed to ATACH up to 2023**	**HNAP ever developed up to 2023** n (% of ATACH members)	**HNAP developed since 2020** n (% of ATACH members)	**New HNAP under development in 2023** n (% of ATACH members)
Africa	28 out of 48: 58%	15 (54%)	6 (21%)	3 (11%)
Asia	22 out of 45: 49%	8 (38%)	4 (19%)	5 (24%)
Europe	10 out of 42: 24%	6 (60%)	3 (30%)	1 (10%)
Northern America	2 out of 2: 100%	2 (100%)	2 (100%)	0 (0%)
Oceania	2 out of 2: 100%	0 (0%)	0 (0%)	2 (100%)
SIDS	10 out of 39: 26%	6 (60%)	5 (50%)	2 (20%)
South and Central America	8 out of 17: 47%	6 (75%)	3 (38%)	1 (13%)
**TOTAL**	82 out of 195: 42%	43 (52%)	23 (28%)	14 (17%)

Indicator 2.1.3: city-level climate change risk assessments

Indicator Authors

Prof Karyn Morrisey

1. Using data from the 2023 CDP Annual Cities Survey, Indicator 2.1.3 captures data at the city level on cities that have undertaken a climate change risk or vulnerability assessment and;
2. The perceived vulnerability city leaders of their public health assets to climate change

Methods

Cities that have undertaken a climate change risk or vulnerability assessment

Step 1. 1,019 cities respond to the CDP.

Step 2. 979 cities report on whether they have undertaken a climate change risk assessment in the risk assessment module. This becomes the initial baseline data for the Risk assessment question.

Data associated with Health Module: the perceived vulnerability city leaders of their public health assets to climate change, health impacts and associated climate hazards.

Step 3. Of the 979 that responded to the risk assessment module, 556 cities responded to the health module (57%). However, due to cities being able to tick multiple health areas, duplicates had to be removed (first duplicates removal). Further analysis uses these 556 cities as the base dataset for the health module.

Step 4. To tabulate the climate hazards impacting each city, multiple climate hazards were allowed per city and multiples of the same climate hazard were allowed per city. Deduplication is required. The 556 cities responding to the Health Module were allowed to reply to multiple hazards, but only one response to each climate hazard was kept.

Step 5. To tabulate the health impacts associated with these climate hazards, multiple health impacts were allowed per city and multiples of the same health impacts were allowed per city. Deduplication is required. The 556 cities responding to the Health Module were allowed to reply to multiple health burdens, but only one response to each health burden was kept.

Data

- 2023 CDP Annual Cities Survey

Caveats

This is a self-reported survey, non-compulsory survey as such data provided may be subjective and response rates can fluctuate, with low uptake in certain areas, particularly the Middle East.

Future form of the indicator

The CDP collect this data annually and it is foreseen that the data collection will continue to 2030. Additional analyses may be conducted using data from the CDP annual survey to monitor associations between city-level health vulnerabilities and track reporting trends over time.

Additional analysis

Table 38: Cities reporting Risk assessment question by HDI.

	Overall	HDI			
		Very High	High	Medium	Low
Yes, a climate risk and vulnerability assessment has been undertaken	765	484	219	41	21
No: but currently undertaking one and it will be complete in the next year	60	25	25	6	4
No: but intending to undertake one in the next two years	112	42	54	12	4
No: not intending to undertake due to lack of financial capacity	10	1	6	1	2
No: not intending to undertake due to lack of expertise/technical capacity	11	3	4	2	2
No: not intending to undertake due to lack of financial capacity and expertise/technical capacity	16	5	9	1	1
No: not intending to undertake due to other higher priorities	3	1	1	1	0
No: not intending to undertake due to other reasons	2	1	1	0	0

Table 39: HDI of cities responding to climate change risk assessment question.

HDI	No of Cities	Percentage
Very High	562	57.4%
High	319	32.5%
Medium	64	6.5%
Low	32	3.3%
N/A	2	0.2%
Total	979	100%

Table 40: Health module.

Number of Cities responding to health module	556	979	57%
Health **systems**	173**	556	31%
Areas **outside** the health sector	49**	556	9%
Health Outcomes	454**	556	82%
**Unable** to measure climate change related health impacts	6	556	1%
Do not know (**DNK**)	0	556	0
Climate Change does not impact Health (null responses)	423	979	43%
Denominator is the 979 cities responding to Risk Assessment Question<br>**Cities can respond to more than one health impact			

Table 41: Health module by HDI.

		**HDI**			
	**All**	**Very High**	**High**	**Medium**	**Low**
Number of Cities responding to health module	556	372	138	31	15
Health **systems**	173	100	54	12	7
Areas **outside** the health sector	49	29	17	1	2
Public Health Outcomes	454	331	91	25	7
**Unable** to measure climate change related health impacts	6	5	1	0	0
Do not know (**DNK**)	0	0	0	0	0
Climate Change does not impact Health (null responses)	423	190	181	33	19

Table 42: Health module by WHO region.

		**WHO region**					
		**Africa**	**America**	**Eastern Mediterranean**	**European**	**South-East Asia**	**Western Pacific**
Number of Cities responding to health module	556	34	270	4	142	28	78
Health **systems**	173	14	77	2	46	7	27
Areas **outside** the health sector	49	4	24	0	13	3	5
Public Health Outcomes	454	22	217	2	124	19	70
**Unable** to measure climate change related health impacts	6	0	3	0	2	0	1
Do not know (**DNK**)	0	0	0	0	0	0	0
Climate Change does not impact Health (null responses)	423	35	247	3	66	22	50

Table 43: Health module by LC region.

	All	LC region						
		**Africa**	**Asia**	**Europe**	**Northern America**	**Oceania**	**SIDS**	**South and Central America**
Number of Cities responding to health module	556	35	102	137	154	12	1	115
Health **systems**	173	14	31	46	23	5	1	53
Areas **outside** the health sector	49	4	9	13	11	1	1	12
Public Health Outcomes	454	23	84	119	146	11	1	70
**Unable** to measure climate change related health impacts	6	0	1	2	2	0	0	1
Do not know (**DNK**)	0	0	0	0	0	0	0	0
Climate Change does not impact Health (null responses)	423	36	78	51	45	11	0	202

Table 44: Climate hazards that cities perceive will impact health outcomes.

**Climate Hazard**	**Number of Cities**	**Percentage**
Oceanic events	11	2%
Other forms of climate-induced landscape change	14	2%
Other coastal events	23	4%
Loss of green space/green cover	32	6%
Soil degradation/erosion	34	6%
Biodiversity loss	44	8%
Hurricanes	48	9%
Snow and ice	48	9%
Other	49	9%
Mass movement	62	11%
Increased water demand	69	12%
Water stress	93	17%
Coastal flooding (incl. sea level rise)	94	17%
Extreme wind	104	19%
Extreme cold	113	20%
Storm	118	21%
Fire weather (risk of wildfires)	147	26%
River flooding	156	28%
Drought	177	32%
Heat stress	198	36%
Infectious disease	204	37%
Heavy precipitation	216	39%
Urban flooding	232	42%
Extreme heat	412	74%

Table 45: Health outcomes related to climate hazards identified by cities.

**Health Outcomes**	**Number of Cities**	**Percentage**
Other Health Burdens	26	5%
Emotional and/or spiritual health impacts	40	7%
Exacerbation of Other NCDs	48	9%
Food-borne infections and illnesses	74	13%
Cold-related illnesses	86	15%
Lack of climate-informed surveillance	92	17%
Damage/destruction to health infrastructure	102	18%
Disruption to health service provision	130	23%
Food and nutrition security	150	27%
Exacerbation of NCD CVD	144	26%
Disruption of health-related services	156	28%

Overwhelming of health service provision	168	30%
Water-borne infections and illnesses	209	38%
Disruption to water	217	39%
Mental health impacts	218	39%
Direct physical injuries and deaths due	229	41%
Vector-borne infections and illnesses	290	52%
Exacerbation of NCD Respiratory Disease	294	53%
Heat-related illnesses	447	80%

2.2: Enabling conditions, adaptation delivery, and implementation

Indicator 2.2.1: climate information for health

Indicator Authors

Dr Joy Shumake-Guillemot, Dr Yasna Palmeiro Silva

Methods

The number of World Meteorological Organization (WMO) national meteorological and hydrological services (NMHS) providing climate services to the health sector is calculated based on self-reported information provided by NMHS through the Country Profile Database Integrated questionnaire. The questionnaire is one of the main sources of information to the WMO Country Profile database and is open all year round for WMO members to update their profile information. Reported data reflects answers to Question number 7.6 of this questionnaire: *Please indicate which user communities/sectors your NMHS provides with climate products/information and estimate the extent to which these products are used to improve decisions*. Human Health is one of multiple sectors which can be chosen.

Data

- World Meteorological Organization Country Profile database: https://community.wmo.int/en/members/profiles.[216]
- 2021 WHO Health and Climate Change Global Survey Report. WHO: Geneva, 2021.[213]
- 2023 WHO Review of Health in Nationally Determined Contributions and Long-term Strategies: Health at the Heart of the Paris Agreement; WHO: Geneva, 2023.[217]
- 2023 State of Climate Services: Health. WMO-No. 1335. Geneva, 2023.[218]

Caveats

The current data source from WMO only considers climate services provided by NMHS. It is unclear the degree to which other providers, such as academic institutions and research projects, private sector products, products from other Ministries, or regional and global products and services are being used, in proportion to services made available by NMHS. The open questionnaire can be updated at any time by WMO members, therefore the figures reported here may change over the year. As each

country may update their profile information at different moments in time, snapshots do not reflect progress for any given year but rather information provided until a certain date. The current questionnaire does not record the number of WMO members that do not provide climate services to the health sector. The questionnaire captures information on the provision of climate services, the status of service provision to the health sector and the type of services provided (divided in 5 categories). Questions do not capture the source or quality of the service and only one of the answer options covers the utility of the climate services. They do not capture whether data originates from national meteorological observations or is resulting from regional or global products. They do not capture the potential use of all-sector forecasts or outlooks which are accessed and used by the health sector. The WMO and WHO have some differences in their individual Member States. Responses collected from WMO Member States were reclassified according to WHO Region. WMO members that are not individual WHO members were excluded from the analyses and include Macao and Hong Kong (reported as China), Curaçao, French Polynesia, and St. Maartens. The following WHO Members are not members of WMO (and therefore representative data is not available): Andorra, Equatorial Guinea, Marshall Islands, Nauru, Palau, San Marino.

Future form of the indicator

In 2019, WMO began implementation of new survey instruments to provide greater insight on the status of climate service provision for the health sector and the type of service provided. Other complementary WMO surveys capturing specific product types, user satisfaction, and application areas, may be publicly available in the future to inform future editions of this indicator.
The WHO Health and Climate Change Country Survey now contains indicators on the inclusion of meteorological information in integrated risk monitoring and early warning systems for climate-sensitive diseases. This information may be used to improve this indicator in future publications.

Indicator 2.2.2: benefits and harms of air conditioning

Indicator Authors

Prof Robert Dubrow, Dr Lingzhi Chu

Methods

This report provides annual estimates for 2000–2021 for proportion of households with air conditioning and heat-related deaths averted by air conditioning among people 65 years of age or greater for the world, by HDI level, and by WHO region. For the 2023 report, the International Energy Agency (IEA) provided data on proportion of households with air conditioning for 12 major individual countries and 14 IEA-defined regions that did not include the 12 individual countries, which taken together constituted the entire world. For the 2024 report, the IEA also provided data on the proportion of households with air conditioning for most countries (with the proviso that only aggregate data be presented in this report). For the first time, this allows for presentation of results by HDI level and WHO Region, respectively.

Proportion of households with air conditioning
To estimate proportion of households with air conditioning by HDI level or by WHO Region, the population-weighted mean of the proportion of households with air conditioning for the countries

within each HDI level or WHO Region was calculated. Global proportion of households with air conditioning was provided directly by IEA.

Number of heat-related deaths averted by air conditioning in the 65-years-and-older population
The 2024 report utilises results from Indicator 1.1.5, Heat-related mortality, which estimates heat-related deaths by calendar-year and country in the population aged 65 years and older, in conjunction with calendar-year and country-level estimates of prevented fraction due to air conditioning (based on proportion of households with air conditioning, as explained below), to estimate the number of heat-related deaths prevented by air conditioning in the 65-years-and-older population by calendar-year for the world, by HDI level, and by WHO Region.

Number of heat-related deaths averted by air conditioning in the 65-years-and-older population was estimated in the 2021 report using data on proportion of households with air conditioning for 22 individual countries and 9 IEA-defined regions that did not include the 22 individual countries. In the 2021 report, it was concluded that there were a number of limitations to the estimate of number of heat-related deaths averted by air conditioning in the 65-and-older population, such that this is considered to be a ‘ballpark’ estimate that will need considerable refinement in future years. One major limitation was the coarse spatial resolution used in the analysis. For the 2024 report, having data on proportion of households with air conditioning for most countries presented an opportunity for a major refinement, while recognising that additional important limitations still exist – discussed below in the Caveats section.

The prevented fraction is the percent reduction in an adverse health outcome due to a preventive exposure, compared with the counterfactual scenario of complete absence of the exposure.[219] The prevented fraction is determined by two factors: 1) the relative risk of the adverse health outcome in exposed persons compared with unexposed persons; and 2) the prevalence of the exposure. The prevented fraction increases as the relative risk drops further below the null and as the prevalence of exposure increases. The formula for prevented fraction is simply:

$$Pe(1 - RRe)$$

where Pe is the prevalence of the preventive exposure and RRe is the relative risk of the adverse health outcome in exposed persons compared with unexposed persons.

For air conditioning as a preventive exposure against heat-related deaths, the prevented fraction is the percent reduction in heat-related deaths due to a given proportion of the population having household air conditioning, compared with a scenario of complete absence of household air conditioning. Thus, the prevented fraction is simply:

$$Pac(1 - RRac)$$

where Pac is the proportion of the population with household air conditioning and RRac is the relative risk of heat-related death among persons who have household air conditioning compared with persons who do not have household air conditioning.

As intuitively expected, the stronger the protection against heat-related mortality conferred by household air conditioning (i.e., the lower the relative risk of heat-related mortality in persons with household air conditioning versus persons without household air conditioning [RRac]), the greater the

prevented fraction; and the higher the proportion of the population with household air conditioning (Pac), the greater the prevented fraction.

The proportion of households with air conditioning is assumed to be a reasonable estimate of the proportion of the population ≥65 years of age with household air conditioning (Pac).

RRac was estimated using the results of a meta-analysis conducted for the 2020 report. Briefly, a literature search that was conducted for non-ecologic, analytical epidemiologic studies that examined the relationship between availability of household air conditioning and heat-related mortality identified 9 eligible studies.[220–228] In a random-effects meta-analysis, RRac was calculated to be 0.24 (95% confidence interval: 0.15, 0.39), which was used to calculate the prevented fraction. Thus, the formula for prevented fraction (PF) is:

$$Pac(1 - RRac) = Pac(1 - 0.24) = Pac(0.76)$$

The prevented fraction could range from 0% for a country with no household air conditioning (i.e., Pac = 0) to 76% for a country in which every household has air conditioning (i.e., Pac = 1.0). For each calendar-year, estimation of the number of heat-related deaths averted[229] by air conditioning is based on estimates of country-specific prevented fractions and on estimates of country-specific number of heat-related deaths in persons aged 65 years and older, taken from Indicator 1.1.5. The latter estimates constitute the observed number of heat-related deaths, given the proportion of the population having household air conditioning in each country (Do).

The number of heat-related deaths that would be expected in the complete absence of household air conditioning (De) was then estimated as:

$$De = Do/(1-PF)$$

Finally, the number of heat-related deaths averted due to the presence of household air conditioning (Da) was estimated as:

$$Da = De - Do$$

To calculate the 95% confidence intervals for Da and the ratio of heat-related deaths averted by air conditioning to actual heat-related deaths (obtained from Indicator 1.1.5), the uncertainty in RRac and in Do were accounted for using the delta method.[230] Country-level Do’s and Da’s, respectively, were summed to calculate the Do and Da for the world, for each HDI level, and for each WHO Region.

Data

The IEA kindly provided data for 2000–2021 that was a revision of the 2000–2021 data used for the 2023 report. Unfortunately, data for 2022 were not available in time to be included in the 2024 report. The data provided by IEA included the proportion of households with air conditioning 1) for most countries and 2) for 14 IEA-defined regions that together with 12 major countries constituted the entire world. In addition, data on carbon dioxide emissions from air conditioning use were provided for the 14 IEA-defined regions and for the entire world.

Table 46: Data provided by IEA on the proportion of households with air conditioning for individual countries, by HDI level

HDI level	Provided	Provided with incomplete data	Not provided
Low	Afghanistan, Benin, Burkina Faso, Central African Republic, Chad, Democratic Republic of the Congo, Djibouti, Ethiopia, Gambia, Haiti, Mali, Mozambique, Niger, Nigeria, Pakistan, Rwanda, Senegal, Sudan, Togo, Uganda, United Republic of Tanzania, Yemen	Madagascar (2019–2021)	Burundi, Eritrea, Guinea, Guinea Bissau, Lesotho, Liberia, Malawi, Sierra Leone, South Sudan
Medium	Angola, Bangladesh, Belize, Bolivarian Republic of Venezuela, Bolivia, Botswana, Cabo Verde, Cambodia, Cameroon, Congo, Cote d'Ivoire, El Salvador, Equatorial Guinea, Eswatini, Ghana, Guatemala, Honduras, India, Iraq, Kenya, Kyrgyzstan, Lao People's Democratic Republic, Mauritania, Morocco, Myanmar, Namibia, Nepal, Nicaragua, Papua New Guinea, Philippines, Syrian Arab Republic, Tajikistan	Zimbabwe (2005–2021)	Bhutan, Comoros, Federated States of Micronesia, Kiribati, Marshall Islands, Sao Tome and Principe, Solomon Islands, Timor-Leste, Tuvalu, Vanuatu, Zambia
High	Albania, Algeria, Antigua and Barbuda, Armenia, Azerbaijan, Bosnia and Herzegovina, Brazil, Bulgaria, China, Colombia, Dominica, Dominican Republic, Ecuador, Egypt, Guyana, Indonesia, Islamic Republic of Iran, Jamaica, Jordan, Lebanon, Maldives, Mexico, Mongolia, North Macedonia, Palau, Paraguay, Peru, Republic of Moldova, Saint Kitts	Gabon (2019–2021)	Barbados, Cuba, Fiji, Grenada, Libya, Occupied Palestinian territory, Saint Vincent and the Grenadines, Samoa

	and Nevis, Saint Lucia, Seychelles, South Africa, Sri Lanka, Suriname, Tonga, Tunisia, Turkmenistan, Ukraine, Uzbekistan, Vietnam		
Very High	Argentina, Australia, Austria, Belarus, Belgium, Brunei Darussalam, Canada, Chile, Costa Rica, Croatia, Cyprus, Czechia, Denmark, Estonia, Finland, France, Georgia, Germany, Greece, Hungary, Iceland, Ireland, Israel, Italy, Japan, Kazakhstan, Kuwait, Latvia, Lithuania, Malaysia, Malta, Mauritius, Montenegro, Netherlands, New Zealand, Norway, Oman, Panama, Poland, Portugal, Qatar, Republic of Korea, Romania, Russian Federation, Saudi Arabia, Serbia, Singapore, Slovakia, Slovenia, Spain, Sweden, Switzerland, Thailand, Trinidad and Tobago, Turkey, United Arab Emirates, United Kingdom, United States of America, Uruguay	Bahamas (2019–2021)	Andorra, Bahrain, Liechtenstein, Luxembourg, San Marino
HDI not available	Hong Kong SAR (China), Puerto Rico	Somalia (2009–2021)	American Samoa (USA), Anguilla, Aruba, Bermuda, British Virgin Islands, Cayman Islands, Commonwealth of the Northern Mariana Islands (USA), Cook Islands, Curaçao, Democratic People's Republic of Korea, French Guiana, French Polynesia (France), Guadeloupe, Guam (USA), Macao SAR

			(China), Martinique, Monaco, Montserrat, Nauru, New Caledonia (France), Niue, Pitcairn Island (UK), Sint Maarten, Tokelau (New Zealand)Turks and Caicos, US Virgin Islands, Wallis and Futuna (France)

Table 47: The 14 IEA-defined regions, which do not include 12 major countries (Canada, Brazil, China, India. Indonesia, Japan, Mexico, Republic of Korea, Russian Federation, South Africa, United Kingdom, United States) for which country-level data were provided.

IEA-defined regions	Countries
**Caspian**	Armenia, Azerbaijan, Georgia, Kazakhstan, Kyrgyzstan, Tajikistan, Turkmenistan, Uzbekistan
**European Union A**	Italy, France, Germany
**European Union B**	Austria, Belgium, Czechia, Denmark, Estonia, Finland, Greece, Hungary, Ireland, Latvia, Lithuania, Luxembourg, Netherlands, Poland, Portugal, Slovakia, Slovenia, Spain, Sweden
**European Union C**	Bulgaria, Croatia, Cyprus, Malta, Romania
**Other Europe A**	Iceland, Israel, Norway, Switzerland, Turkey
**Other Europe B**	Albania, Belarus, Bosnia and Herzegovina, Gibraltar, Holy See, Kosovo, Montenegro, North Macedonia, Republic of Moldova, Serbia, Ukraine
**North Africa**	Algeria, Egypt, Libya, Morocco, Tunisia
**Other Africa**	Angola, Benin, Botswana, Burkina Faso, Burundi, Cabo Verde, Cameroon, Central African Republic, Chad, Comoros, Congo, Côte d'Ivoire, Democratic Republic of the Congo, Djibouti, Equatorial Guinea, Eritrea, Eswatini, Ethiopia, Gabon, Gambia, Ghana, Guinea, Guinea-Bissau, Kenya, Lesotho, Liberia, Madagascar, Malawi, Mali, Mauritania, Mauritius, Mozambique, Namibia, Niger, Nigeria, Réunion, Rwanda, Sao Tome and Principe, Senegal, Seychelles, Sierra Leone, Somalia, South Sudan, Sudan, Togo, Uganda, United Republic of Tanzania, Zambia, Zimbabwe
**Chile, Colombia, and Costa Rica**	Chile, Colombia, and Costa Rica
**Other Latin America**	Antigua and Barbuda, Argentina, Aruba, Bahamas, Barbados, Belize, Bermuda, Bolivia, Bolivarian Republic of Venezuela, British Virgin Islands, Cayman Islands, Cuba, Curaçao, Dominica, Dominican Republic, Ecuador, El Salvador, Falkland Islands (Malvinas), French Guiana, Grenada, Guadeloupe, Guatemala, Guyana, Haiti, Honduras, Jamaica, Martinique, Montserrat, Caribbean Netherlands, Nicaragua, Panama, Paraguay, Peru, Saint Kitts and Nevis, Saint Lucia, Saint Pierre and Miquelon, Saint Vincent and the Grenadines, Sint Maarten (Dutch part), Suriname, Trinidad and Tobago, Turks and Caicos Islands, Uruguay
**Middle East**	Bahrain, Iraq, Islamic Republic of Iran, Jordan, Kuwait, Lebanon, Oman, Qatar, Saudi Arabia, Syrian Arab Republic, United Arab Emirates, Yemen
**Association of Southeast Asian Nations (ASEAN) countries**	Brunei Darussalam, Cambodia, Lao People’s Democratic Republic, Malaysia, Myanmar, Philippines, Pitcairn Island, Singapore, Thailand, Vietnam
**Other Asia**	Afghanistan, Bangladesh, Bhutan, Cook Islands, Democratic People’s Republic of Korea, Fiji, French Polynesia, Kiribati, Macao SAR (China), Maldives, Mongolia, Nepal, New Caledonia, Pakistan, Palau, Papua New

	Guinea, Samoa, Solomon Islands, Sri Lanka, Taiwan, Timor-Leste, Tonga, Vanuatu
**Australia and New Zealand**	Australia, New Zealand

In the calculation of population-weighted means of the proportion of households with air conditioning for the countries within each HDI level or WHO Region or the calculation of country-level prevented fractions, the proportion of households with air conditioning for individual countries for which IEA estimates were not available was estimated by the proportion of households with air conditioning for the country's IEA region. For the countries for which IEA provided incomplete data for proportion of households with air conditioning, this proportion was estimated for the years before the year with the first available value as the minimum of the first available value and the estimate for the country's IEA region.

The following 47 countries were not included in the calculations of heat-related deaths averted by air conditioning because they were not included in the Indicator 1.1.5 calculations of heat-related mortality: American Samoa (USA); Andorra; Anguilla; Antigua and Barbuda; Aruba; Bahrain; Barbados; Bermuda; British Virgin Islands; Brunei Darussalam; Cayman Islands; Commonwealth of the Northern Mariana Islands (USA); Cook Islands; Curaçao; Dominica; Federated States of Micronesia; French Polynesia (France); Grenada; Guadeloupe; Guam (USA); Hong Kong SAR (China); Liechtenstein; Macao SAR (China); Maldives; Malta; Marshall Islands; Martinique; Monaco; Montserrat; Nauru; New Caledonia (France); Niue; Occupied Palestinian territory; Palau; Pitcairn Island (UK); Saint Kitts and Nevis; Saint Lucia; San Marino; Seychelles; Singapore; Sint Maarten; Tokelau (New Zealand); Tonga; Turks and Caicos; Tuvalu; Vanuatu; Wallis and Futuna (France).

Caveats

Estimate of number of heat-related deaths averted by air conditioning

There were a number of limitations to the estimate of number of heat-related deaths averted by air conditioning in the 65-and-older population, such that this is considered to be a ballpark estimate that will need considerable refinement in future years:

1. The prevented fraction calculation was based on a pooled RRac of 0.24 from a meta-analysis that included nine studies: four from the United States; two from France; one from Italy, one from Greece, and one from Australia. This RRac may differ in other parts of the world, but studies of the relationship between availability of household air conditioning and heat-related mortality are sparse, such that it is not currently possible to make region-specific estimates of RRac.
2. The target population for four of the nine studies included in the meta-analysis was the general population, whereas the target population for five of the studies was the elderly (persons ≥65 years of age in two studies, persons ≥75 years of age in one study, and nursing home residents in two studies). Because the target population of Indicator 1.1.5, Heat-related mortality was ≥65 years of age, restricting the meta-analysis to the five studies that focused on the elderly was considered. However, when it was found that one of the five studies contributed 73% of the weight in the restricted meta-analysis, the decision was made not to apply this restriction so as not to allow a single study to have such a high amount of influence on the estimate of RRac. For the nine studies, to build greater uncertainly into the analysis, a

random-effects meta-analysis, that assumes heterogeneity among studies and results in a wider 95% confidence interval than would a fixed-effects meta-analysis, was conducted. Nevertheless, it is possible that RRac differs between persons ≥65 years of age and younger persons.

3. Eight of the nine studies in the meta-analysis estimated the relative risk of heat-related death among persons who have household air conditioning compared with persons who do not have household air conditioning (i.e., RRac) during heatwaves (e.g., the 2003 French heatwave, the 1995 Chicago heatwave). However, in Indicator 1.1.5, Heat-related mortality, heat-related deaths constitute excess deaths attributable to temperatures over the optimal (i.e., minimum mortality) temperature. It is possible that RRac for heat-related deaths defined in this way differs from RRac during heatwaves.
4. Because the meta-analysis is based on observational studies, it is possible that the RRac estimate was distorted by confounding in some or all of the 9 studies included in the meta-analysis. That is, having household air conditioning may be associated with other characteristics that prevent heat-related mortality (e.g., good baseline health status, not living alone) for which there was no adjustment in some or all of the 9 studies.
5. However, although caution should be observed in claiming causality from observational studies, some observational associations are so strong and consistent, and are supported by toxicological, physiological, and/or other experimental studies (such as the association between smoking and lung cancer), that causality can, in fact, be claimed. In this case, it is likely that the strong negative association between air conditioning and heat-related mortality observed in the meta-analysis (RRac = 0.24) does represent a causal association. Most of Hill's classic criteria for causality are met, including strength of association, consistency across studies, temporality, and plausibility. Based on physiological considerations alone, it is highly likely that having access to a cool indoor environment, by virtue of air conditioning or other means, confers protection against heat-related mortality.
6. The proportion of households with air conditioning was used to estimate the proportion of the 65-and-older population having household air conditioning. This estimate did not take into account the size of households with air conditioning versus those without air conditioning or the vulnerability to heat stress of persons living in households with air conditioning versus those without air conditioning. In addition, the presence of air conditioning in a household does not guarantee the use of air conditioning in that household.
7. To estimate the number of heat-related deaths prevented by air conditioning, the finer the spatial resolution, the more accurate the estimates. The data available on proportion of the population having household air conditioning was at the country level which was a major improvement over the analysis in the 2021 report. Nevertheless, in this estimation, it was by necessity assumed that the proportion of the population having household air conditioning is homogeneous within each country. This assumption may not be accurate, especially for larger, heterogeneous countries.
8. The estimation of calendar-year-specific and country-specific heat-related deaths from Indicator 1.1.5, Heat-related mortality, had its own limitations, described in the Indicator 1.1.5 section of this Appendix. In particular, potential over-estimation of heat-related deaths in later calendar years would result in over-estimation of number of heat-related deaths averted by air conditioning in later calendar years.

Future form of the indicator

As new studies become available, the meta-analysis of the relationship between having household air conditioning and heat-related mortality will be updated. Another improvement would be inclusion of all age groups, not just persons ≥65 years, in the estimate of heat-related deaths averted due to air conditioning. In addition, city-level case studies to estimate number of lives saved from air conditioning versus premature deaths from exposure to $PM_{2.5}$ due to air conditioning may be performed. The indicator may be updated each year as new data become available on air conditioning use. Finally, metrics related to more efficient cooling (e.g., national building codes, minimum energy performance standards, labelling rules for air conditioners) and progress on implementing the Kigali Amendment may be tracked in the future. The future form of the indicator section for Indicator 1.1.5, heat-related mortality, in this appendix discusses how examination of heat-related mortality could be improved, which would also improve the validity of the air conditioning indicator.

Additional analysis

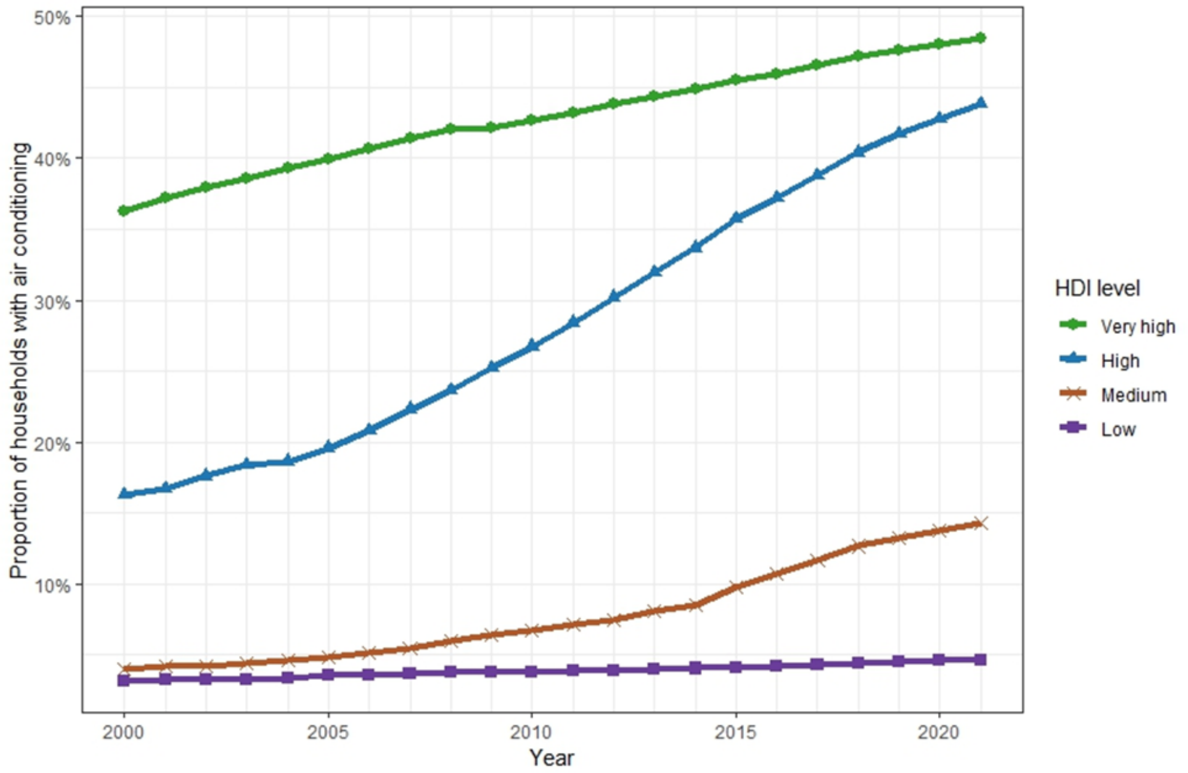

Figure 121: Proportion of households with air conditioning by HDI group

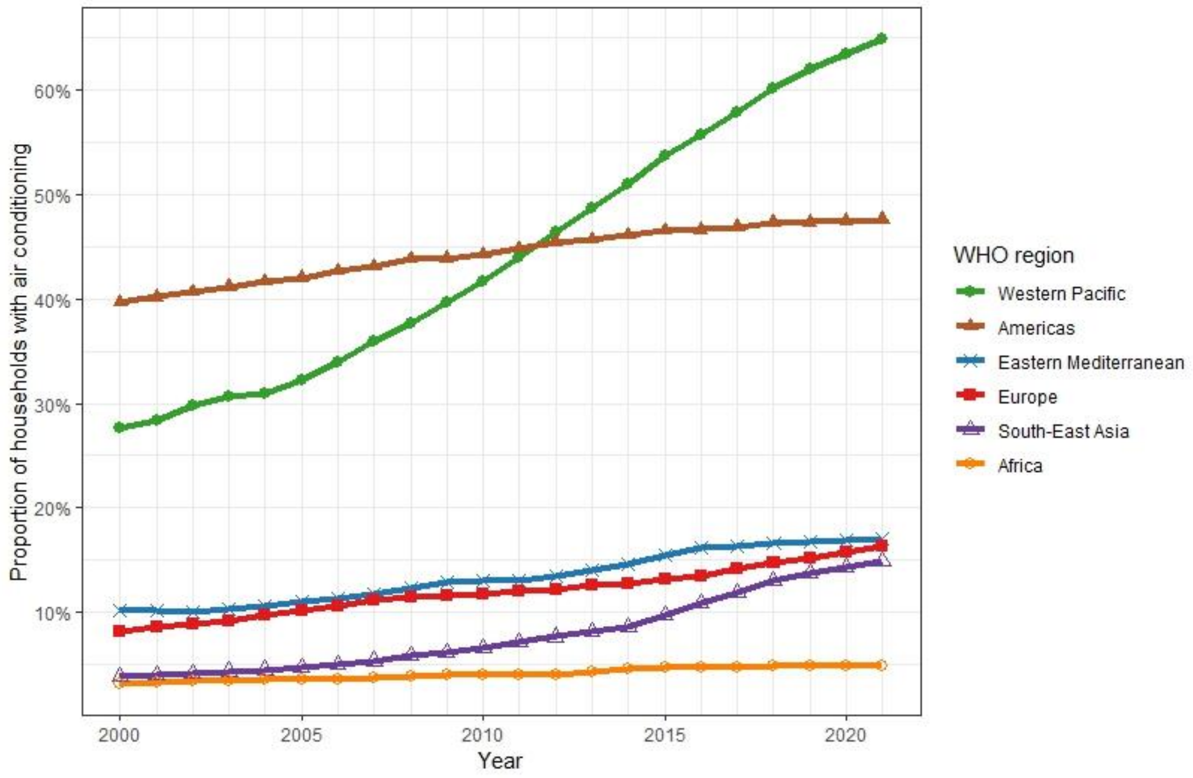

Figure 122: Proportion of households with air conditioning by WHO region

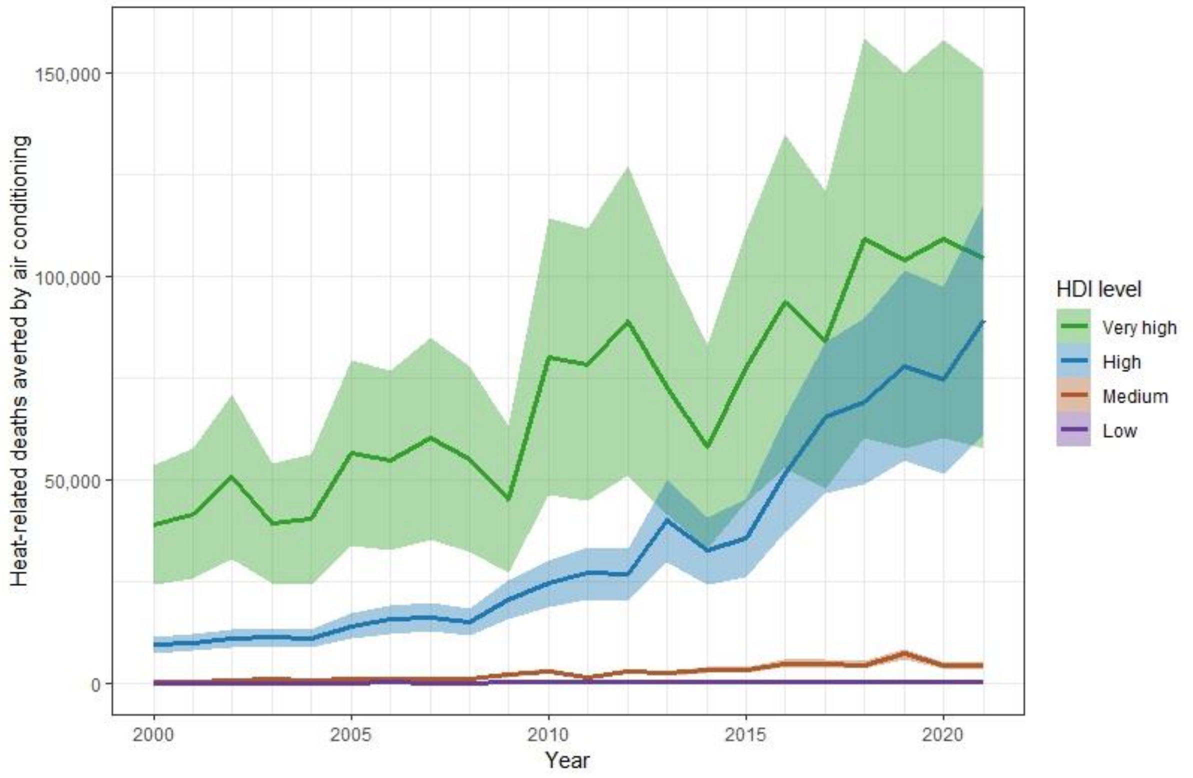

Figure 123: Heat-related deaths averted by air conditioning with 95% confidence intervals, by HDI level

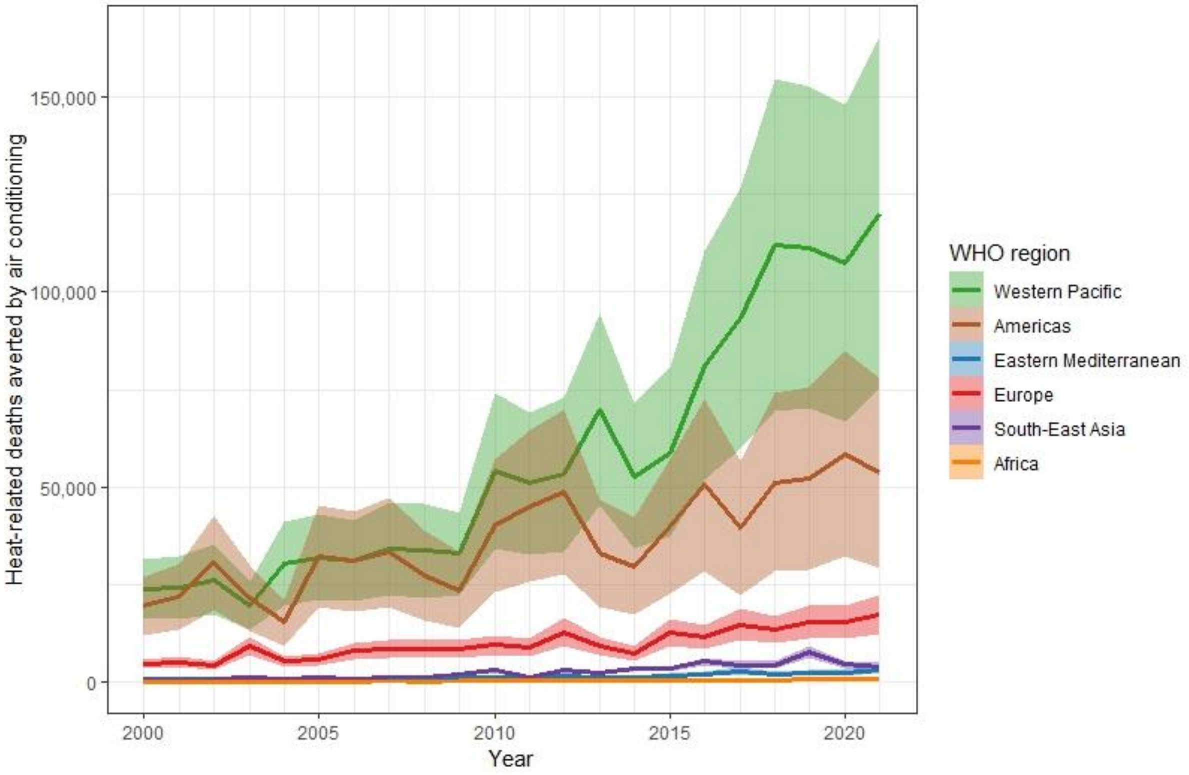

Figure 124: Heat-related deaths averted by air conditioning with 95% confidence intervals, by WHO region

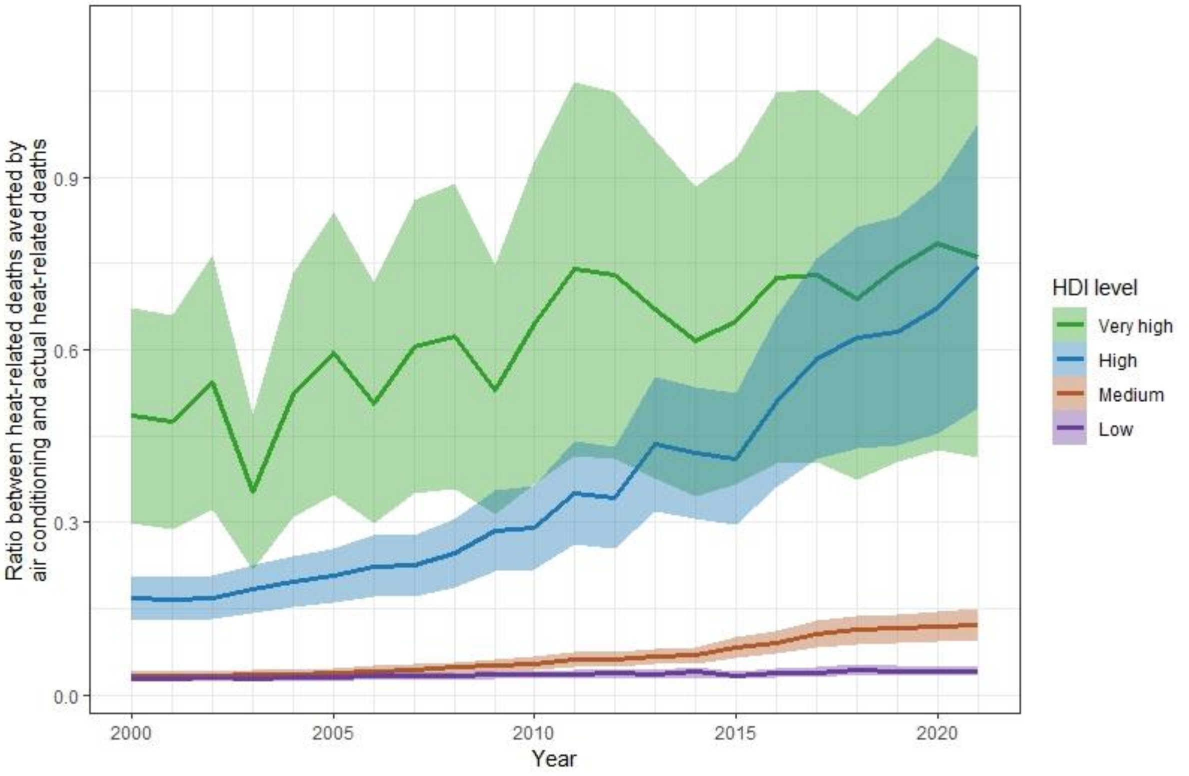

Figure 125: Ratio between heat-related deaths averted by air conditioning and actual heat-related deaths with 95% confidence intervals, by HDI level

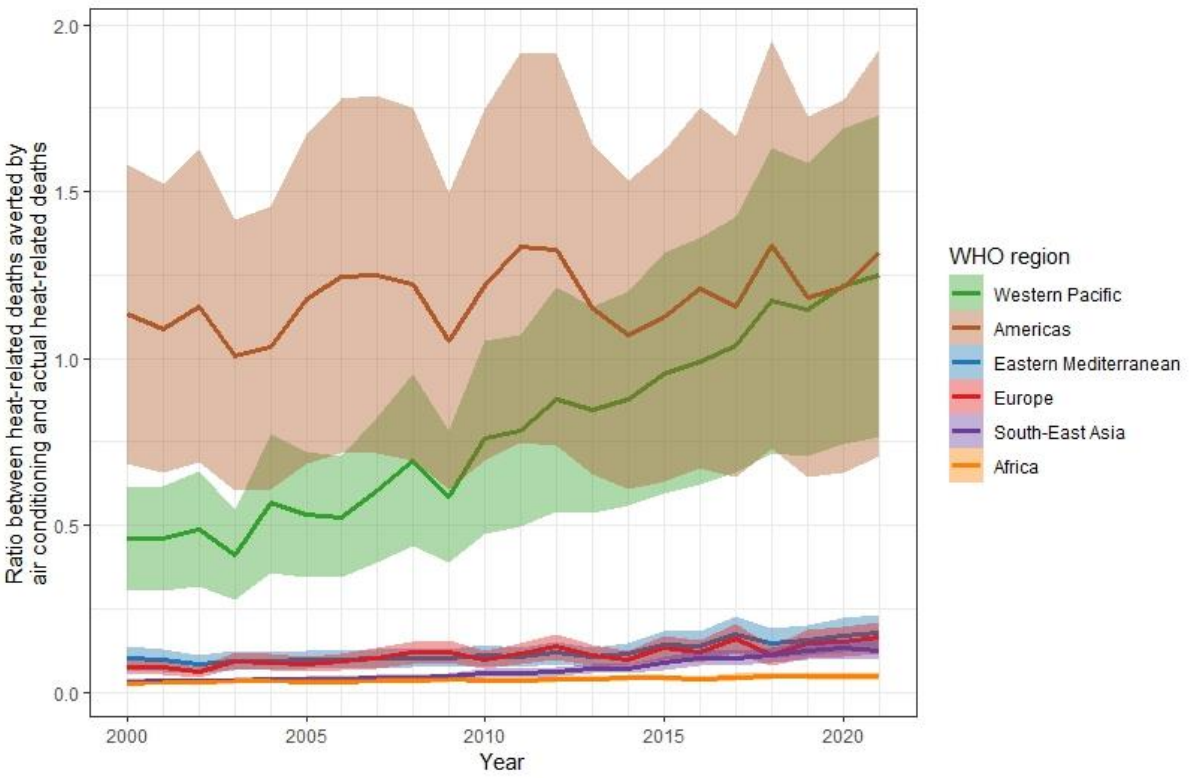

Figure 126: Ratio between heat-related deaths averted by air conditioning and actual heat-related deaths with 95% confidence intervals, by WHO region

Indicator 2.2.3: urban green space

Indicator Authors

Dr Jennifer D. Stowell, Prof Patrick Kinney

Methods

Urban centre spatial extents were defined by the Global Human Settlement (GHS) program of the European commission.[231] The GHS uses a blend of demographic and remote sensing data to define more than 10,000 urban centres worldwide. The team computed the greenness indicator for global urban centres with populations larger than 500,000. For countries that lacked urban centres meeting this threshold, the team selected the most populated city where possible, giving a final count of 1,041 urban centres across 174 countries. Due to missing data in either the GHS or the Normalized Difference Vegetation Index (NDVI) data, 22 countries (mostly small island states) were not represented in the analysis.

Data on population size for all years came from the Gridded Population of the World from the Center for International Earth Science Information Network (CIESN, Columbia University), which models the distribution of human population at 30 arc-second output resolution.[232]

Green space was estimated using the Normalized Difference Vegetation Index (NDVI). The NDVI is the most commonly used satellite-based vegetation index, it calculates the ratio of the differences between near infrared radiation and visible radiation to the sum of these two measures. NDVI values range from -1.0 to 1.0 with values less than 0 indicating bodies of water and values close to 1 indicating high levels of vegetation density or greenness.[233] For this process, publicly available data from the Landsat satellite was utilised, a joint program of the USGS and NASA.[234] Landsat images the Earth's surface at 30-meter resolution approximately every two weeks (~16 days). To account for seasonal fluctuations, NDVI for each of the following time periods was computed (with season labels based on the northern hemisphere):

- Winter–December 1 of previous year through February 28
- Spring–March 1 through May 31
- Summer–June 1 through August 31
- Fall–September 1 through November 30

This was analysed for five different years: 2015, 2020, 2021, 2022, and 2023. Landsat 8 (2015, 2020, 2021) and Landsat 9 (2022, 2023) were used to estimate values for the included years. For each year and city, a total of four exposure metrics were calculated: peak NDVI (maximum NDVI across the four seasons); annual mean NDVI based on the four-season average NDVI; population-weighted peak NDVI; and population-weighted mean NDVI. The population weighted NDVI was computed for each city by multiplying each NDVI value (peak and four-season average) by the population size of the corresponding year within the same 1´1 km raster, summing up over the weighted values within the urban extent, and dividing by the sum of the weights, as shown by the equation below:

$$\frac{\sum_{i=1}^{n}(NDVI_i * population_i)}{\sum_{i=1}^{n}(population_i)}$$

Additional analyses include subsetting the data by levels of the Human Development Index (HDI), climate regions as defined by the Köppen Climate Classification System (see Figure 9SM), *Lancet* Countdown (LC) regional country groupings, and WHO Region.[235,236]Google Earth Engine was used to generate the raw data for analysis. The R Statistical Software was used for data analysis and management and to compute the four metrics described above. The team defined 'Level of Greenness' according to Table 48.

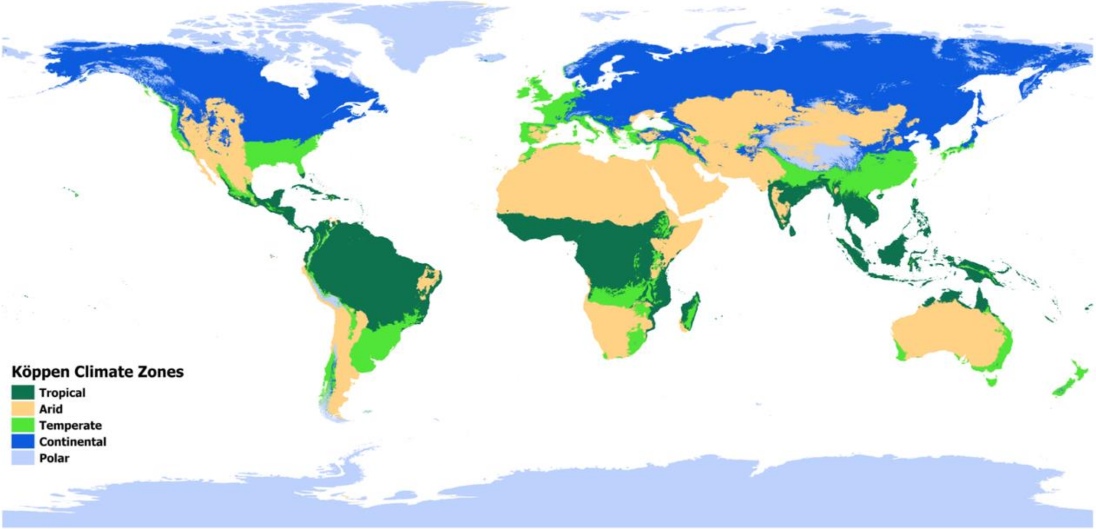

Figure 127: Köppen Climate Regions. Designated climate regions of the world using the Köppen Climate Zones system.

Table 49: Categorisation of Greenness Levels

Level of Greenness	Population-Weighted Peak NDVI
Exceptionally Low	<0.20
Very low	0.20–0.29
Low	0.30–0.39
Moderate	0.40–0.49
High	0.50–0.59
Very High	0.60–0.69
Exceptionally High	≥0.70

Data

- Global Human Settlement Programme of the European Commission (GHS) used to identify urban centres.[231]
- Population size identified from NASA GPWv4.[232]
- Satellite data were downloaded from the publicly available Landsat satellite, a joint program of the US Geological Survey and NASA.[234]
- Global climate regions from the Köppen Climate Classification System.[235]
- Human Development Index (HDI) at the United Nations Development Programme, Human Development Reports[215]

Caveats

This approach has some limitations. First, while satellite-based vegetation measures are used extensively to measure greenness, the NDVI cannot decipher the quality of greenness (e.g., curated park vs vacant lot), the type of green space (e.g., park vs. forest), the type of vegetation (e.g., shrubs vs. trees) or social characteristics (e.g., level of security). However, studies have demonstrated that NDVI performs adequately when compared with environmental psychologists' evaluations of green

spaces.[237] In addition, reviews of the literature on greenness and health have been undertaken and found consistent and strong evidence of associations of higher greenness measured by NDVI, with improvements in birthweights, physical activity, lower mortality rates, and lower levels of depression.[238,239] Second, it is important to note that missing values due to cloud cover or other factors may limit the generalisability of the findings.

Future form of indicator

Future versions of the urban greenness indicator will continue to assess changes in NDVI over time. In the coming years, the team intend to incorporate blue space as a separate indicator and combined with greenspace. The team also plan to explore the combination of urban green space with other indicators such as extreme temperature and vulnerability.

Additional analysis

The findings below represent multiple measures of urban greenness and allow for examination of trends over time.

Table 50: Global average population-weighted peak-season NDVI and global percent moderate or above (population-weighted average peak-season NDVI ≥0.40)

Year	Pop-weighted average peak-season NDVI	% > Moderate Greenness<br>% (n urban centres / total urban centres)
2015	0.34	28% (288/1,041)
2020	0.34	28% (286/1,041)
2021	0.34	27% (280/1,041)
2022	0.34	27% (281/1,041)
2023	0.34	28% (291/1,041)

Population-weighted peak-season NDVI

Table 51: Population-weighted peak -season NDVI by climate region

Climate Region	2015	2020	2021	2022	2023
**Arid**	0.24	0.25	0.24	0.24	0.24
**Continental**	0.37	0.39	0.38	0.38	0.39
**Polar**	0.14	0.13	0.13	0.14	0.11
**Temperate**	0.35	0.35	0.35	0.35	0.35
**Tropical**	0.39	0.38	0.38	0.38	0.38

Table 52: Population-weighted peak-season NDVI by HDI

HDI-level	2015	2020	2021	2022	2023
**Low**	0.31	0.30	0.29	0.29	0.30
**Medium**	0.37	0.37	0.37	0.37	0.37
**High**	0.31	0.32	0.31	0.32	0.32
**Very High**	0.36	0.36	0.35	0.35	0.35

Table 53: Population-weighted peak-season NDVI by WHO region

WHO Region	2015	2020	2021	2022	2023
**African**	0.33	0.32	0.32	0.31	0.33
**Americas**	0.34	0.34	0.33	0.34	0.31
**E Mediterranean**	0.22	0.22	0.21	0.20	0.22
**European**	0.37	0.37	0.37	0.37	0.37
**SE Asian**	0.40	0.40	0.41	0.40	0.40
**W Pacific**	0.31	0.32	0.31	0.32	0.32

Table 54: Population-weighted peak-season NDVI by Lancet Countdown (LC) region

LC Region	2015	2020	2021	2022	2023
**African**	0.31	0.30	0.29	0.29	0.30
**Asia**	0.34	0.34	0.34	0.34	0.34
**European**	0.40	0.40	0.40	0.39	0.40
**North American**	0.39	0.40	0.37	0.40	0.38
**Oceania**	0.35	0.33	0.35	0.34	0.35
**SIDS**	0.38	0.38	0.38	0.38	0.38
**South/Central America**	0.31	0.30	0.30	0.30	0.30

Percent moderate or above (population-weighted average peak-season NDVI ≥0.40)

Tables present percentage of urban centres and (number of urban centres / total urban centres).

Table 55: Climate region percent moderate or above (population-weighted average peak-season NDVI ≥0.40)

Climate Region	2015	2020	2021	2022	2023
**Arid**	4% (9/233)	5% (11/233)	6% (13/233)	5% (12/233)	4% (9/233)
**Continental**	48% (69/144)	44% (63/144)	43% (62/144)	51% (73/144)	42% (60/144)
**Polar**	0% (0/1)	0% (0/1)	0% (0/1)	0% (0/1)	0% (0/1)
**Temperate**	28% (102/368)	27% (98/368)	26% (93/368)	24% (87/368)	29% (107/368)
**Tropical**	39% (115/295)	42% (123/295)	40% (117/295)	40% (117/295)	38% (111/295)

Table 56: Percent moderate or above by HDI (population-weighted average peak-season NDVI ≥0.40)

HDI-level	2015	2020	2021	2022	2023
**Low**	18% (19/105)	18% (19/105)	15% (16/105)	13% (14/105)	17% (18/105)
**Medium**	37% (110/297)	36% (108/297)	40% (118/297)	36% (108/297)	41% (122/297)
**High**	17% (63/373)	16% (61/373)	16% (61/373)	18% (67/373)	18% (68/373)
**Very High**	37% (95/254)	38% (96/254)	33% (84/254)	36% (91/254)	33% (83/254)

Table 57: Percent moderate or above by WHO region (population-weighted average peak-season NDVI ≥0.40)

WHO Region	2015	2020	2021	2022	2023
**African**	21% (24/112)	20% (23/112)	18% (20/112)	18% (20/112)	23% (26/112)
**Americas**	28% (49/178)	28% (49/178)	25% (45/178)	25% (45/178)	25% (44/178)
**E Mediterranean**	5% (6/113)	6% (7/113)	5% (6/113)	4% (4/113)	4% (5/113)
**European**	44% (70/160)	45% (72/160)	40% (64/160)	44% (71/160)	43% (68/160)
**SE Asian**	47% (120/257)	46% (117/257)	49% (126/257)	45% (116/257)	47% (120/257)
**W Pacific**	9% (19/221)	8% (17/221)	8% (18/221)	11% (24/221)	11% (25/221)

Table 58: Percent moderate or above by Lancet Countdown region (population-weighted average peak-season NDVI ≥0.40)

LC Region	2015	2020	2021	2022	2023
**African**	17% (26/150)	16% (24/150)	15% (22/150)	14% (21/150)	18% (27/150)
**Asia**	24% (137/569)	24% (135/569)	25% (141/569)	24% (135/569)	25% (145/569)
**European**	55% (70/128)	56% (72/128)	50% (64/128)	55% (71/128)	53% (68/128)
**North American**	60% (34/57)	58% (33/57)	49% (28/57)	51% (29/57)	46% (26/57)
**Oceania**	17% (1/6)	0% (0/6)	0% (0/6)	17% (1/6)	0% (0/6)
**SIDS**	27% (6/22)	41% (9/22)	45% (10/22)	50% (11/22)	55% (12/22)
**South/Central America**	12% (13/109)	11% (12/109)	13% (14/109)	11% (12/109)	12% (13/109)

Estimates of Urban Green Space

Table 59: Estimates of Urban Green Space by Climate Region (2015, 2020, 2021, 2022, 2023)

**Climate region**	**Peak NDVI**					**Four-season NDVI**					**Pop. weighted Peak NDVI**					**Pop. weighted Four-season NDVI**				
	**2015**	**2020**	**2021**	**2022**	**2023**	**2015**	**2020**	**2021**	**2022**	**2023**	**2015**	**2020**	**2021**	**2022**	**2023**	**2015**	**2020**	**2021**	**2022**	**2023**
**Arid**	0.25	0.26	0.25	0.25	0.25	0.21	0.21	0.21	0.21	0.21	0.24	0.25	0.24	0.24	0.24	0.20	0.20	0.20	0.20	0.20
**Continental**	0.38	0.40	0.39	0.39	0.39	0.26	0.27	0.26	0.27	0.27	0.37	0.39	0.38	0.38	0.39	0.25	0.26	0.25	0.26	0.27
**Polar**	0.17	0.15	0.16	0.17	0.14	0.14	0.13	0.12	0.13	0.11	0.14	0.13	0.13	0.14	0.11	0.12	0.11	0.10	0.11	0.09
**Temperate**	0.37	0.36	0.36	0.36	0.37	0.30	0.30	0.29	0.30	0.30	0.35	0.35	0.35	0.35	0.35	0.28	0.29	0.28	0.28	0.29
**Tropical**	0.41	0.40	0.40	0.40	0.40	0.35	0.35	0.34	0.34		0.39	0.39	0.38	0.38	0.38	0.33	0.33	0.33	0.33	0.28

Table 60: Estimates of Urban Green Space by HDI (2015, 2020, 2021, 2022, 2023)

**HDI group**	**Peak NDVI**					**Four-season NDVI**					**Pop. weighted Peak NDVI**					**Pop. weighted Four-season NDVI**				
	**2015**	**2020**	**2021**	**2022**	**2023**	**2015**	**2020**	**2021**	**2022**	**2023**	**2015**	**2020**	**2021**	**2022**	**2023**	**2015**	**2020**	**2021**	**2022**	**2023**
**Low**	0.32	0.31	0.31	0.30	0.31	0.26	0.26	0.25	0.25	0.26	0.31	0.30	0.30	0.29	0.30	0.25	0.25	0.24	0.24	0.25
**Medium**	0.38	0.38	0.38	0.37	0.38	0.31	0.32	0.31	0.31	0.31	0.37	0.37	0.37	0.37	0.37	0.31	0.31	0.30	0.30	0.31
**High**	0.34	0.35	0.34	0.34	0.34	0.27	0.28	0.27	0.28	0.28	0.31	0.32	0.31	0.32	0.32	0.25	0.25	0.25	0.26	0.26
**Very High**	0.37	0.36	0.36	0.36	0.36	0.29	0.29	0.28	0.28	0.28	0.36	0.36	0.35	0.35	0.35	0.28	0.28	0.27	0.27	0.28
**Global Mean**	0.35	0.35	0.36	0.35	0.35	0.29	0.29	0.28	0.28	0.29	0.34	0.34	0.35	0.34	0.34	0.27	0.27	0.27	0.27	0.28

Table 61: Estimates of Urban Green Space by WHO region (2015, 2020, 2021, 2022, 2023)

**WHO Region**	**Peak NDVI**					**Four-season NDVI**					**Pop. weighted Peak NDVI**					**Pop. weighted Four-season NDVI**				
	**2015**	**2020**	**2021**	**2022**	**2023**	**2015**	**2020**	**2021**	**2022**	**2023**	**2015**	**2020**	**2021**	**2022**	**2023**	**2015**	**2020**	**2021**	**2022**	**2023**
**African**	0.35	0.34	0.33	0.33	0.34	0.28	0.27	0.27	0.27	0.28	0.33	0.32	0.32	0.31	0.33	0.26	0.26	0.25	0.25	0.27
**Americas**	0.36	0.36	0.36	0.36	0.34	0.31	0.30	0.30	0.30	0.29	0.34	0.34	0.33	0.34	0.31	0.29	0.29	0.28	0.28	0.27
**E Mediterranean**	0.23	0.23	0.22	0.22	0.23	0.20	0.20	0.19	0.19	0.20	0.22	0.22	0.21	0.20	0.22	0.19	0.19	0.18	0.17	0.19
**European**	0.38	0.38	0.38	0.38	0.38	0.29	0.29	0.28	0.28	0.30	0.37	0.37	0.37	0.37	0.37	0.28	0.28	0.27	0.28	0.29
**SE Asian**	0.41	0.41	0.41	0.41	0.41	0.34	0.35	0.34	0.34	0.34	0.41	0.40	0.41	0.40	0.40	0.34	0.34	0.33	0.33	0.34
**W Pacific**	0.33	0.34	0.33	0.34	0.34	0.25	0.26	0.26	0.26	0.27	0.30	0.32	0.31	0.32	0.32	0.23	0.24	0.24	0.25	0.28

Table 62: Estimates of Urban Green Space by LC region (2015, 2020, 2021, 2022, 2023)

LC Region	Peak NDVI					Four-season NDVI					Pop. weighted Peak NDVI					Pop. weighted Four-season NDVI				
	2015	2020	2021	2022	2023	2015	2020	2021	2022	2023	2015	2020	2021	2022	2023	2015	2020	2021	2022	2023
**Africa**	0.33	0.32	0.31	0.31	0.32	0.27	0.26	0.26	0.26	0.27	0.31	0.30	0.29	0.29	0.30	0.25	0.24	0.24	0.24	0.25
**Asia**	0.35	0.35	0.35	0.35	0.35	0.28	0.29	0.28	0.28	0.29	0.34	0.34	0.34	0.34	0.34	0.27	0.28	0.27	0.27	0.28
**Europe**	0.41	0.41	0.41	0.40	0.42	0.30	0.31	0.30	0.30	0.32	0.40	0.40	0.40	0.39	0.40	0.29	0.29	0.29	0.29	0.32
**Northern America**	0.40	0.40	0.38	0.29	0.38	0.32	0.31	0.31	0.31	0.26	0.39	0.40	0.38	0.39	0.37	0.32	0.31	0.30	0.31	0.25
**Oceania**	0.34	0.33	0.35	0.34	0.35	0.31	0.30	0.32	0.31	0.33	0.35	0.33	0.35	0.34	0.35	0.31	0.30	0.32	0.31	0.33
**SIDS**	0.38	0.39	0.38	0.39	0.39	0.34	0.34	0.35	0.35	0.34	0.38	0.38	0.38	0.38	0.38	0.33	0.33	0.34	0.34	0.34
**South & Central America**	0.34	0.33	0.33	0.33	0.35	0.30	0.29	0.29	0.29	0.29	0.31	0.30	0.30	0.30	0.30	0.27	0.26	0.26	0.26	0.26

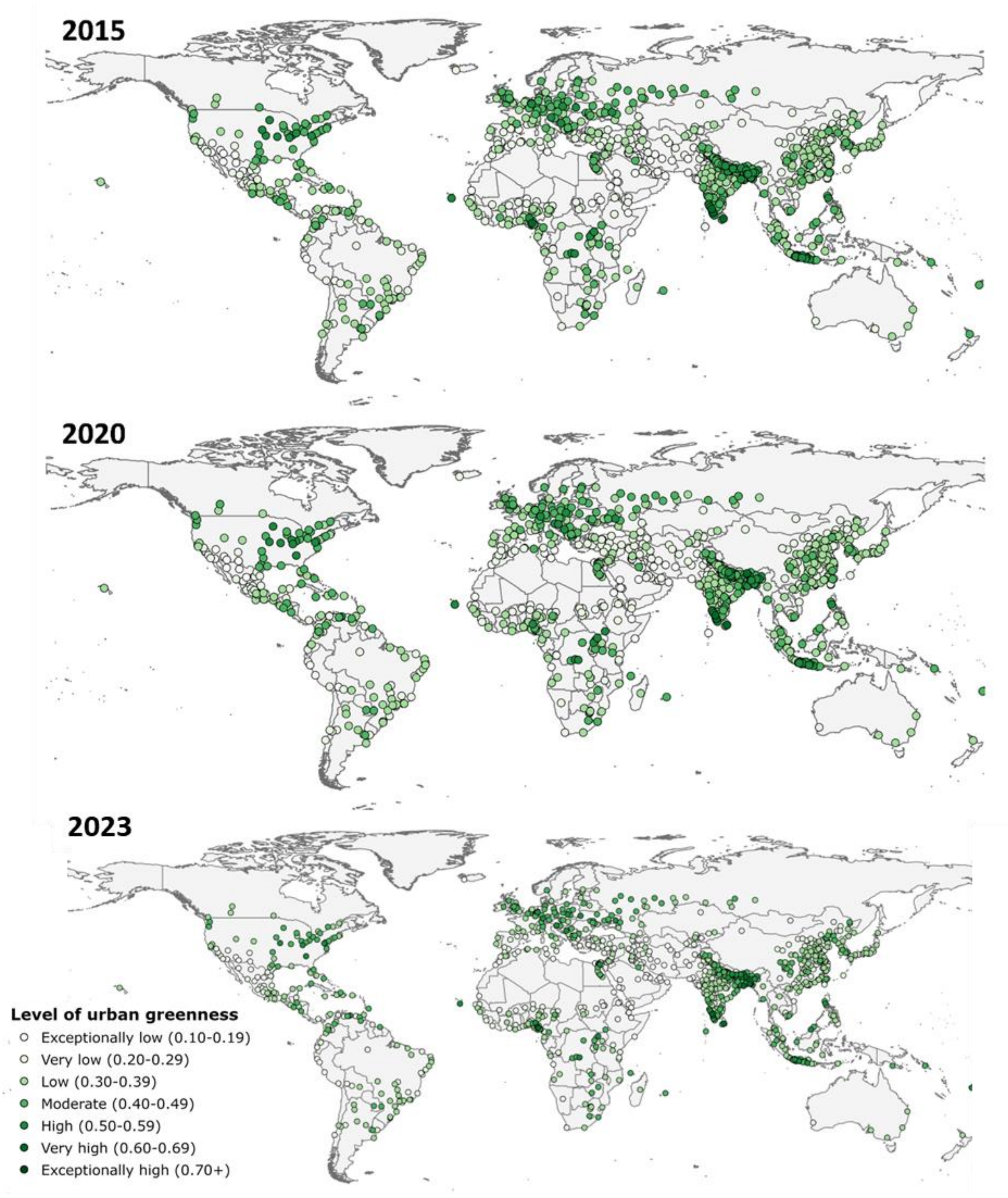

Figure 128: Temporal changes in urban greenness. Levels of urban greenness change between 2015, 2020, and 2023.

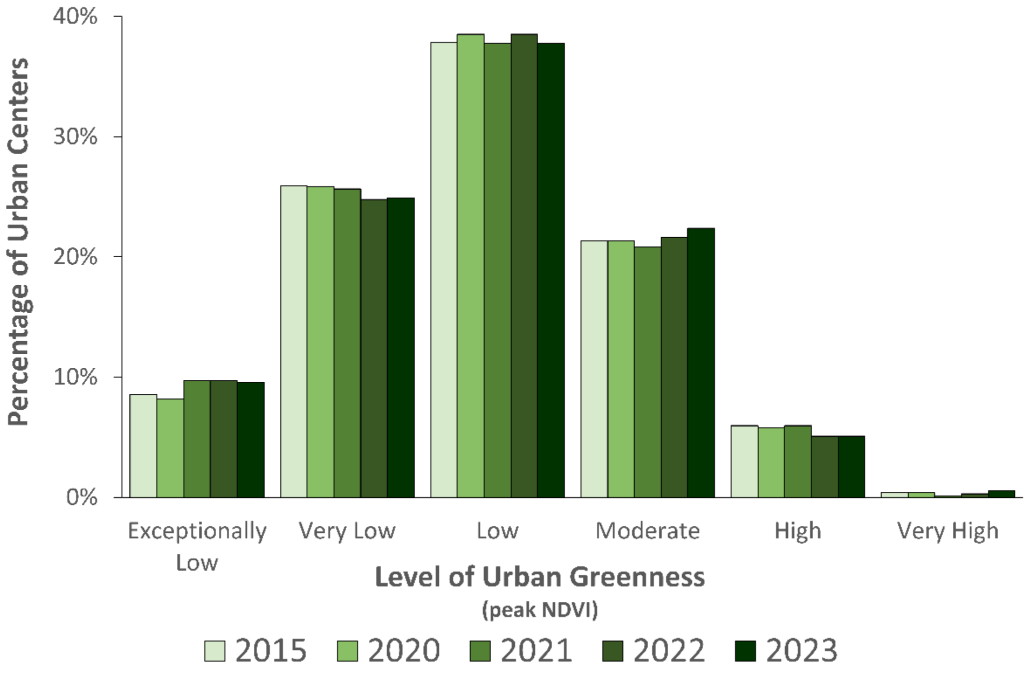

Figure 129: Percentage of urban green centres by urban greenness level over multiple years

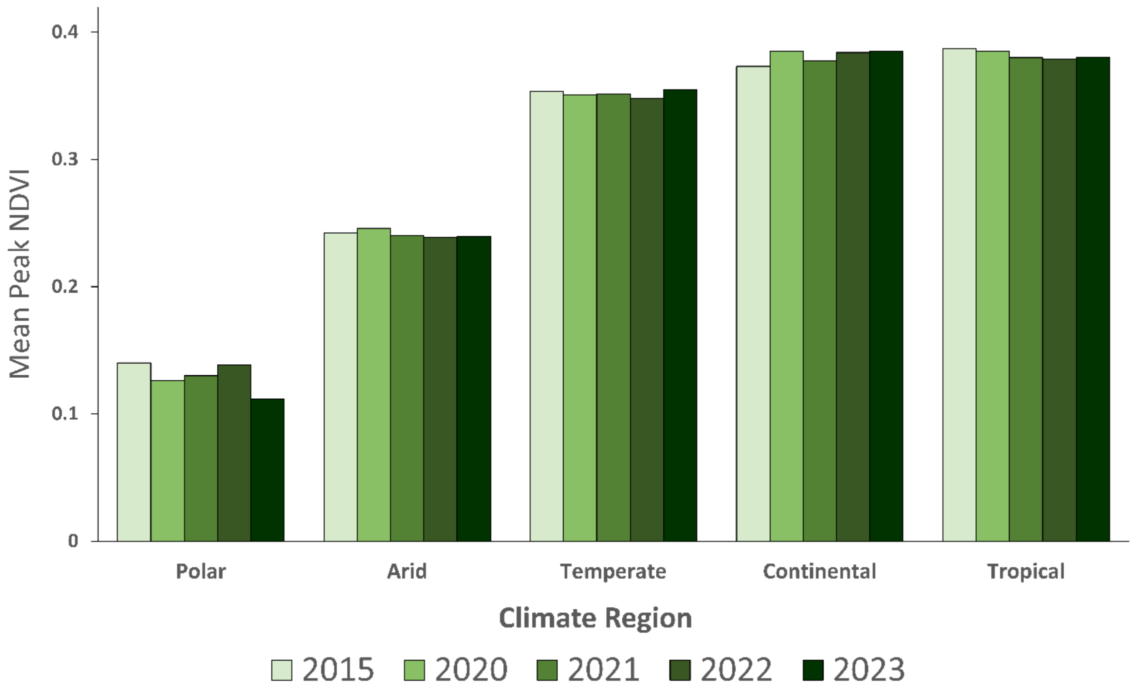

Figure 130: Mean, population-weighted peak-season NDVI by climate region and year.

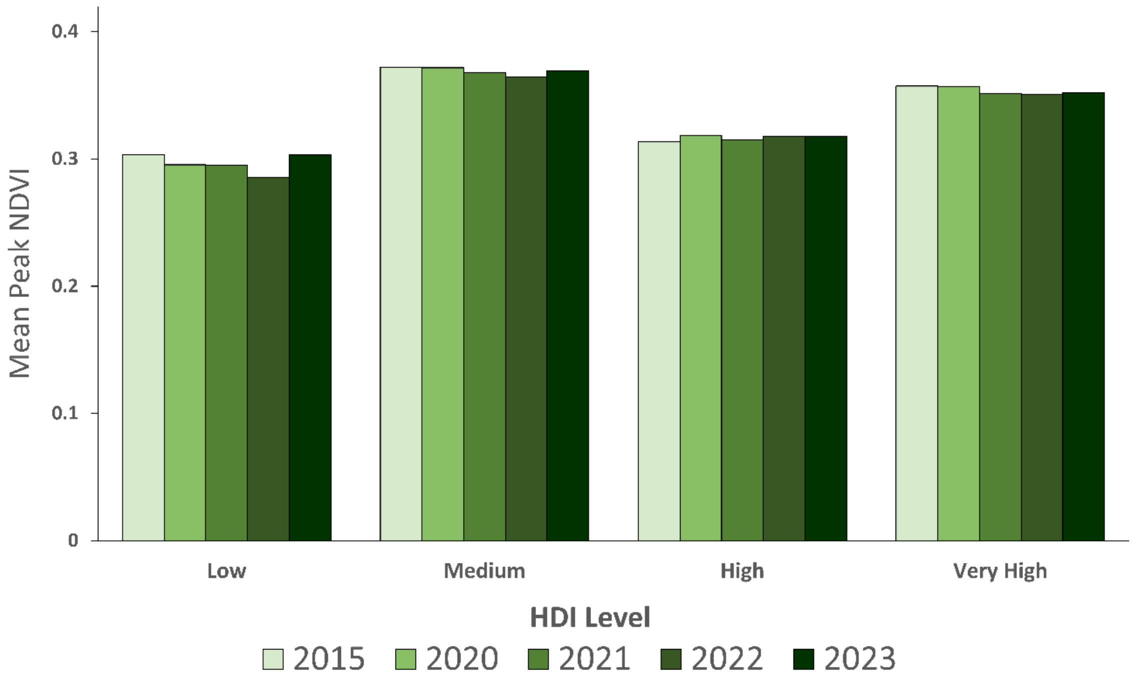

Figure 131: Mean, population-weighted peak-season NDVI by HDI and year.

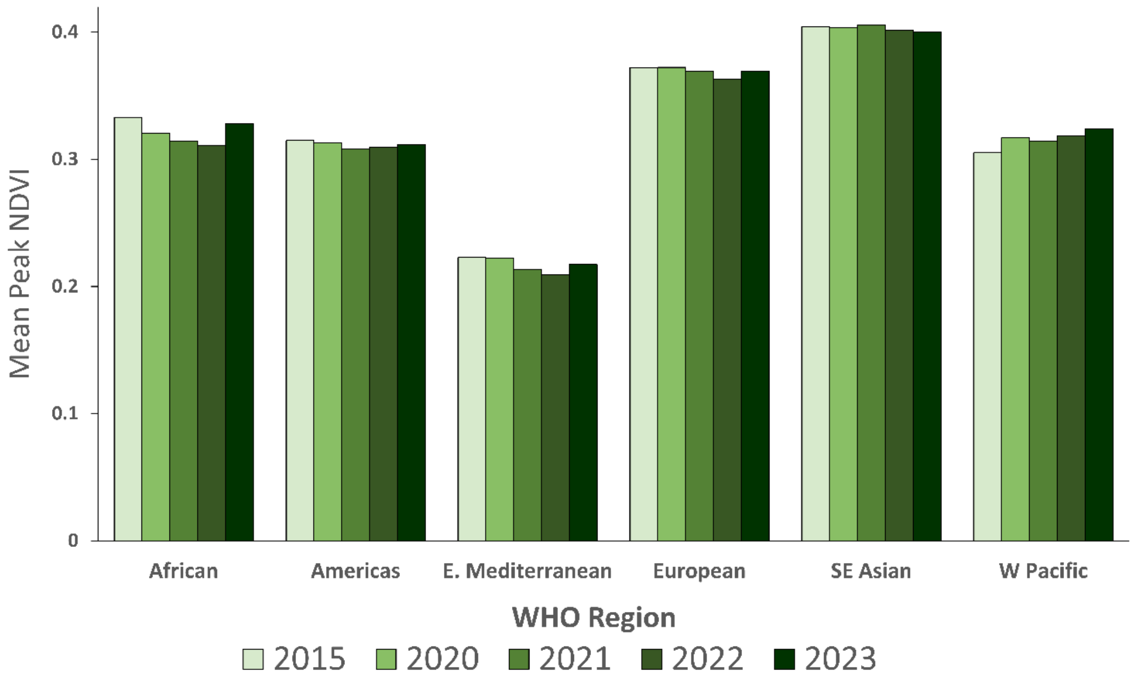

Figure 132: Mean, population-weighted peak-season NDVI by WHO region and year.

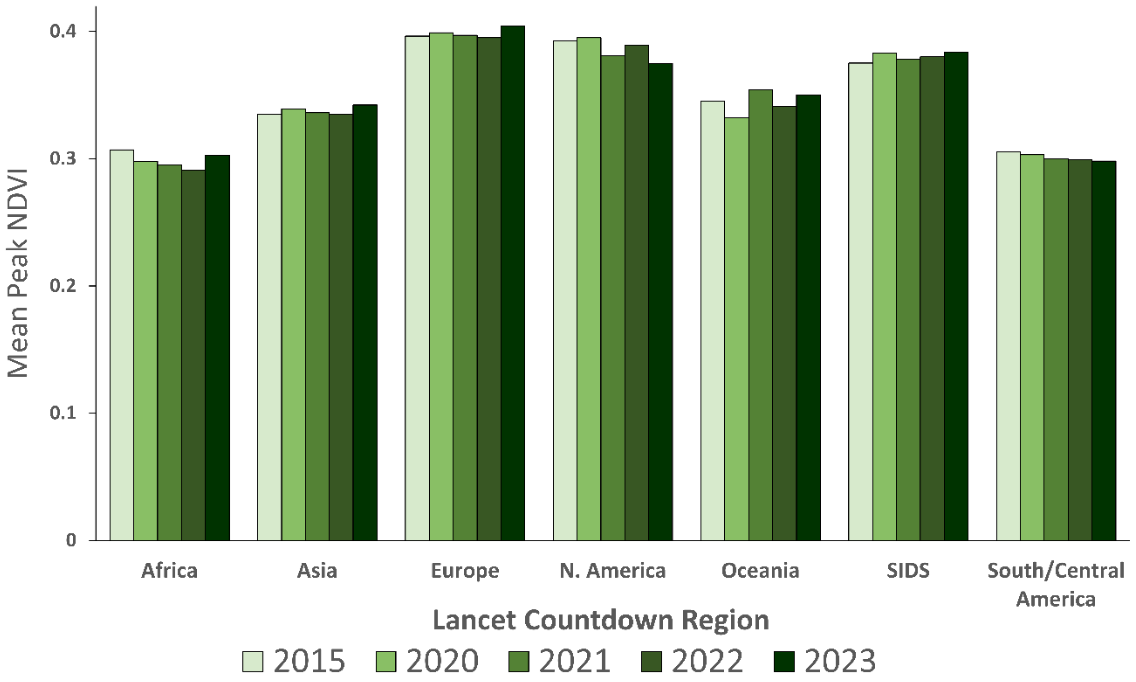

Figure 133: Mean, population-weighted peak-season NDVI by Lancet Countdown Region designation and year.

Indicator 2.2.4: global multilateral funding for health adaptation programmes

Indicator Authors

Louis Jamart, Dr Ana-Catarina Pinho-Gomes, Dr Yasna Palmeiro Silva

Methods

This indicator is based on the projects' information from the Green Climate Fund (GCF) https://www.greenclimate.fund/projects.

Data collection

Data were collected from PDF files of Approved funding proposal, accessed via the GCF Project Portfolio, and collated into a spreadsheet.

The GCF Project Portfolio is accessible online from the GCF Website following the Prompts: https://www.greenclimate.fund/ > Projects & Programmes > Lists of Projects.
The filter functionality was used to filter projects by 'Theme': [Adaptation] or [Cross-Cutting]. Lapsed projects were excluded.

PDF files of the Project Approval Documents were downloaded from each of the projects, reviewed individually, and key data points were transferred into a spreadsheet, including:

- Project Reference Number
- Project Name
- Recipient country
- Sector
- Theme: Adaptation, Cross-cutting (i.e., adaptation and mitigation components, or Mitigation
- Status: Approved, Under implementation, or Lapsed
- Date approved, under implementation, or completed
- Result area: Buildings, cities, industries, and appliances; Ecosystems and ecosystem services; Energy generation and access; Forest and Land use; Health, food, and water security; Infrastructure and built environment; Livelihoods of people and communities; Transport.
- Project Region
- Total project value
- Total GCF Financing: loan, guarantee, grant, equity, results-based payment
- Total co-financing
- Percentage of funding for Adaptation (in case of being a Cross-cutting project) (from 2021, outlined in section A.4 Result areas(s))
- Percentage of funding for "Health and wellbeing, food, and water security" (from 2021, outlined in section A.4 Result areas(s))

Once data were collected in a spreadsheet, 10% of the entries were double checked at random. No entry mistakes were found.

Data related to the reported funding for climate and health projects by ATACH members were obtained through an update of baseline data (2012 WHO Health and Climate Change Global Survey). The methodology of this update is described in Indicator 2.1.1 of this document.

Data analysis

i) Funding for adaptation elements for each of these projects was calculated as: Total GCF financing approved * Percentage of financing for adaptation elements (from 2021, outlined in section A.4 Result areas(s))
ii) Funding directed towards potential health co-benefits from adaptation elements for each of these projects was calculated as: Total GCF financing approved * Percentage of funding for Health and wellbeing, food, and water security elements of the project (from 2021, outlined in section A.4 Result areas(s))

The overall percentage of funding going towards potential health co-benefits from adaptation was calculated by dividing the sum of (ii) by the sum of (i) and then multiplying by 100.

Data

This indicator used data from the Green Climate Fund (GCF) at https://www.greenclimate.fund/

Caveats

This indicator provides in-depth analysis of funding approved by the GCF for projects with potential health benefits through adaptation measures. While this likely represents the overall trend in multilateral financing, it is possible that other funds show a different trend.

Future form of the indicator

In future years, this indicator will expand the scope in order to include other climate funds, including those committed at COP28 to provide $1 billion to health. Additionally, acknowledging the importance of the health co-benefits of mitigation, this indicator may include the contribution of mitigation funding towards health.

Indicator 2.2.5: detection, preparedness, and response to health emergencies

Indicator Authors

Dr Diarmid Campbell-Lendrum, Dr Yasna Palmeiro Silva

Methods

This indicator takes data from the International Health Regulations (IHR (2005)) State Party Self-Assessment Annual Reporting Tool (SPAR). Under the IHR (2005) all States Parties are required to have or to develop minimum core public health capacities to implement the IHR (2005) effectively. IHR

(2005) also states that all States Parties should report to the World Health Assembly annually on the implementation of IHR (2005).[240] In order to facilitate this process, WHO developed an IHR Monitoring questionnaire, interpreting the Core Capacity Requirements in Annex 1 of IHR (2005) into 35 indicators for 15 capacities. Since 2010, this self-reporting IHR monitoring questionnaire is sent annually to National IHR Focal Points (NFPs) for data collection. It contains a checklist of 35 indicators specifically developed for monitoring the development and implementation of 15 IHR capacities. The method of estimation calculates the proportion/percentage of attributes (a set of specific elements or functions which reflect the level of performance or achievement of a specific indicator) reported to be in place in a country.

The core capacities to implement the IHR (2005) have been established by a technical group of experts, as those capacities required to detect, assess, notify, and report events, and to respond to public health risks and emergencies of national and international concern. To assess the development and strengthening of core capacities, a set of components are measured for each of the core capacities, by considering a set of one to three indicators that measure the status and progress in developing and strengthening the IHR core capacities. Each indicator is assessed by using a group of specific elements referred to as 'attributes' that represents a complex set of activities or elements required to carry out this component. The annual questionnaire has been conducted since 2010 with a response rate of 72% in 2012, 66% in 2016 and 85% in 2017, with 100% of countries reporting at least once since 2010. Annual reporting results are complemented by after action reviews, exercises, and joint external evaluation (JEE).

At the beginning of 2018, in compliance with the recommendations of the IHR Review Committee on Second Extensions for Establishing National Public Health Capacities and on IHR Implementation and following formal global consultations with States Parties held in 2015, 2016, 2017, and 2018, the WHO Secretariat replaced the IHR Monitoring questionnaire by the IHR State Party Self-assessment Annual Reporting (SPAR) Tool.[241]

Between 2018 and 2020, the SPAR tool provided results for IHR core capacity 8 (C8), National Health Emergency Framework. This core capacity had 3 indicators C8.1 Planning for emergency preparedness and response mechanism, C8.2 Management of health emergency response operations, and C8.3 Emergency resource mobilization. However, adjustments were made in the SPAR tool in 2021. In 2021, core capacity 7 (C7) Health Emergency Management, reported on three indicators: C.7.1 planning for health emergencies, C.7.2 management of health emergency response, and C.7.3 emergency logistic and supply chain management. The indicator C8.3 no longer exists in this eSPAR version 2. This makes C.7 Health emergency management in version 2 incomparable with C8 version 1. Therefore, findings in the 2024 Lancet Countdown Report cannot be compared to findings in the 2021 or earlier reports.[241]
The SPAR tool scoring system has remained the same since 2018. It is summarised in the table below (Table 63 and Table 64).

Table 65: SPAR scoring system of IHR core capacities

Indicator level	Score	Score range	Colour
1	20	0-20	
2	40	21-40	
3	60	41-60	
4	80	61-80	
5	100	81-100	

Table 66: Lancet Countdown's classification of level of implementation of core capacity 7 of the IHR SPAR tool. This follows the same classification as in the 2022 report.

Indicator level	Score	Score range	Colour	Lancet Countdown's classification
1	20	0-20		Very low
2	40	21-40		Low
3	60	41-60		Medium
4	80	61-80		High
5	100	81-100		Very high

In this report, information for capacity 7 is provided comparing 2022 and 2023, and the level of implementation in 2023. Data for 2022 were updated on 12 August 2023 and downloaded on 23 March 2024 from https://extranet.who.int/e-spar. A total of 186 State Parties had reported to the Self-Assessment Annual Reporting Tool. Data for 2023 were updated on 18 March 2024 and downloaded on 23 March 2024 from https://extranet.who.int/e-spar. A total of 194 State Parties had reported to the Self-Assessment Annual Reporting Tool.
Additionally, information on capacity 3.2 — finacing for public health emergency response — under IHR is also included to evaluate its relationship with capacity 7.

Data analysis
Data with complete information were considered in the analysis, which means that countries with no classification under HDI were excluded (i.e., Cook Islands, Democratic People's Republic of Korea, Monaco, Nauru, Niue, Somalia) only for that specific analysis.

Data

- International Health Regulations (2005) Annual Reporting. Data were downloaded from the electronic IHR State Parties Self-Assessment Annual reporting Tool (e-SPAR) for 2023.[241] https://extranet.who.int/e-spar
- Human Development Index (HDI) at the United Nations Development Programme, Human Development Reports[215]

Caveats

There are some limitations to considering these capacities as proxies of health system adaptive capacity and system resilience. Most importantly, IHR monitoring questionnaire responses are self-reported. Secondly, the countries that report IHR implementation differ from year to year within these regional aggregate scores.

Thirdly, IHR Core Capacity Requirements are not specific to climate change, and hence whilst they provide a proxy baseline, they do not directly measure a country's adaptive capacity in relation to climate driven risk changes. Fourthly, these findings capture potential capacity – not action. Finally, the quality of surveillance for early detection and warning is not shown and neither is the impact of that surveillance on public health. Response systems have been inadequate in numerous public health emergencies and thus the presence of such plans is not a proxy for their effectiveness. Nevertheless, these capacities provide a useful starting point to consider the potential adaptive capacity of health systems globally.

Future form of the indicator

The World Health Assembly resolution WHA73.1 requested the WHO Director-General to initiate a process of impartial, independent, and comprehensive evaluation of the WHO-coordinated international health response to COVID-19, including the mechanisms in place under the IHR. Future forms of this indicator will need to evolve along with the outputs of this review.

Additional analysis

Table 67: Average score of C7 under IHR State Parties assessments by Human Development Index (HDI).

HDI	2022 level C7 (n=185) mean (p25; p75)	2023 level C7 (n=193) mean (p25; p75)	N (%) States that increase C7 from 2002 to 2023*	N (%) States that decrease C7 from 2002 to 2023*
Low	61.2 (51.5; 73)	57.5 (45; 73)	8 (17%)	15 (28%)
Medium	63.2 (53; 73)	61.9 (50; 73)	13 (27%)	11 (20%)
High	75 (67; 87)	74 (67; 87)	14 (29%)	14 (26%)
Very High	80.4 (73; 93)	78.5 (73; 93)	11 (23%)	12 (22%)
N/A	76 (60; 100)	65.5 (43; 87)	2 (4%)	2 (4%)
**TOTAL**	72 (60; 87)	69.8 (53; 80)	48 (100%)	54 (100%)

*Based on 185 observations

Table 68: Average score of C7 under IHR State Parties assessments by WHO region.

**WHO region**	**2022 level C7 (n=185) mean (p25; p75)**	**2023 level C7 (n=193) mean (p25; p75)**	**N (%) States that increase C7 from 2002 to 2023***	**N (%) States that decrease C7 from 2002 to 2023***
Africa	59.1 (47; 73)	57.3 (47; 73)	13 (27.1%)	20 (37%)
Americas	76.7 (67; 87)	72.9 (60; 87)	11 (22.9%)	12 (22.2%)
Eastern Mediterranean	71.4 (53; 87)	70.8 (53; 87)	7 (14.6%)	4 (7.4%)
Europe	76.7 (70; 90)	74.5 (65; 87)	9 (18.8%)	11 (20.4%)
South-East Asia	80 (73; 90)	77.5 (70; 90)	5 (10.4%)	1 (1.9%)
Western Pacific	77.9 (70; 90)	74.3 (67; 87)	3 (6.3%)	6 (11.1%)
**TOTAL**	72 (60; 87)	69.8 (53; 80)	48 (100%)	54 (100%)

*Based on 185 observations

Table 69: Average score of C7 under IHR State Parties assessments by Lancet Countdown (LC) region.

**LC region**	**2022 level C7 (n=185) mean (p25; p75)**	**2023 level C7 (n=193) mean (p25; p75)**	**N (%) States that increase C7 from 2002 to 2023***	**N (%) States that decrease C7 from 2002 to 2023***
Africa	60.9 (52; 73)	59.9 (47; 73)	17 (35.4%)	18 (33.3%)
Asia	76.7 (67; 93)	73.6 (60; 87)	8 (16.7%)	12 (22.2%)
Europe	77.2 (67; 93)	75.7 (67; 87)	8 (16.7%)	7 (12.9%)
Northern America	96.5 (95; 98)	96.5 (95; 98)	0 (0%)	0 (0%)
Oceania	83.5 (82; 85)	80 (80; 80)	0 (0%)	1 (1.9%)
SIDS	71.2 (60; 80)	70.9 (60; 80)	11 (22.9%)	7 (12.9%)
South and Central America	76.2 (67; 87)	66.7 (53; 87)	4 (8.3%)	9 (16.7%)
**TOTAL**	72 (60; 87)	69.8 (53; 80)	48 (100%)	54 (100%)

*Based on 185 observations

Table 70: Average score of C3.2 under IHR State Parties assessments by Human Development Index (HDI)

**HDI**	**2023 level C3.2 n=193 (mean p25; p75)**
Low	48 (40; 60)
Medium	57 (40; 80)
High	70 (60; 80)
Very High	82 (80; 100)
N/A	60 (30; 80)
**TOTAL**	67 (40; 80)

*Based on 185 observations

Table 71: Average score of C3.2 under IHR State Parties assessments by WHO Region.

**WHO Region**	**2023 level C3.2 n=193 (mean p25; p75)**
AFR	53 (40; 60)
AMR	63 (40; 80)
EMR	64 (20; 100)
EUR	80 (80; 100)
SEAR	75 (70; 80)
WPR	67 (45; 80)
**TOTAL**	67 (40; 80)

*Based on 185 observations

Table 72: Average score of C3.2 under IHR State Parties assessments by Lancet Countdown (LC) region.

**LC Region**	**2023 level C3.2 n=193 (mean p25; p75)**
Africa	55 (40; 60)
Asia	71 (60; 85)
Europe	82 (80; 100)
Northern America	100 (100; 100)
Oceania	80 (70; 90)
SIDS	62 (40; 80)
South and Central America	58 (40; 80)
**TOTAL**	67 (40; 80)

*Based on 185 observations

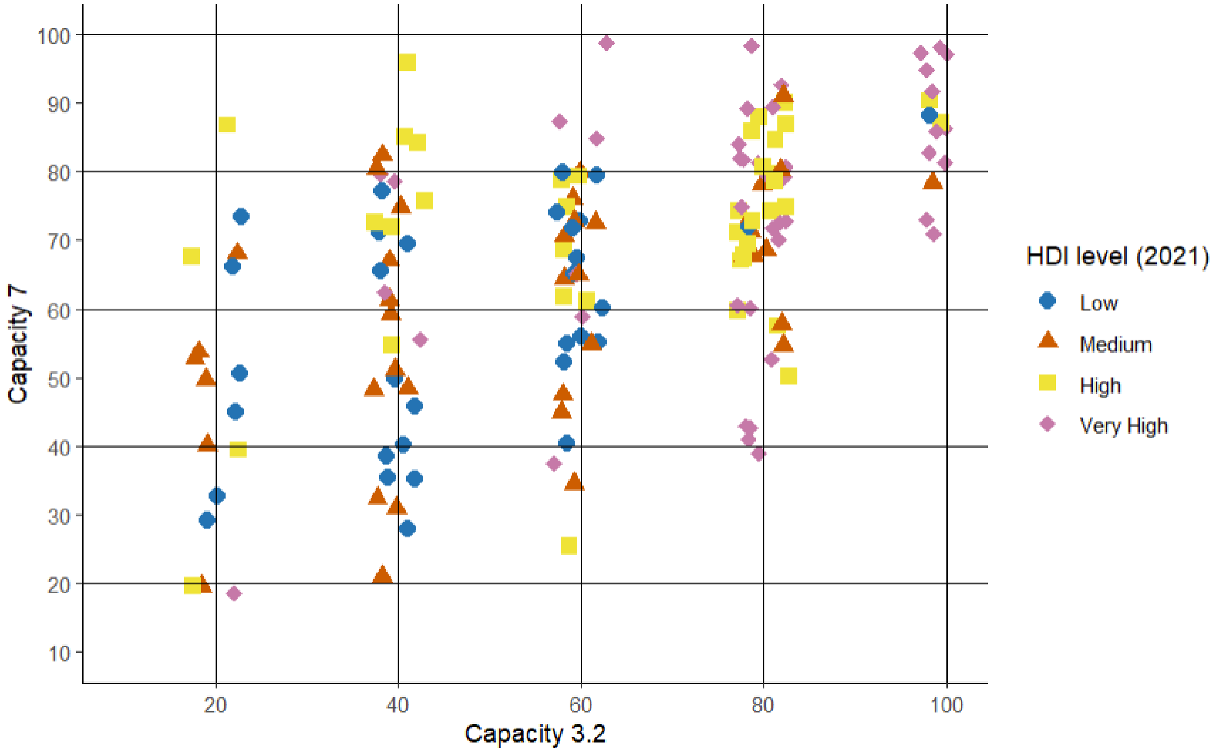

Figure 134: Scatterplot between capacity 7 and capacity 3.2 by HDI level (2021).

Indicator 2.2.6: climate and health education and training

Indicator Authors

Dr Cecilia Sorensen, Dr Ying Zhang

Methods

An online survey was conducted to quantify the percentage of degree-granting public health institutions currently providing education and training in climate and health knowledge and skills, the number of public health students trained per year for each level of degree; and the degree to which students gain capacity in climate and health knowledge and analytic skills across eight core domains. To enable global participation and dissemination of the survey instrument, the Global Consortium on Climate and Health Education (GCCHE) partnered with the Global Network for Academic Public Health, which consists of eight regional public health associations, representing over 550 public health schools from 105 countries. A letter of invitation to participate in the survey was sent to member institutions of each regional association through the associations' communication channels. Additionally, the survey was distributed to public health institution members of the GCCHE and affiliated organisations, totalling 1,251 institutions invited from 138 countries. The survey instructions indicated that when the training in climate and health was offered at the institution, the survey should be completed by faculty who design or teach climate and/or planetary health-related curriculum or faculty on curriculum committees familiar

with climate and or planetary health related content. Survey participation was entirely voluntary, however, to increase follow-up, two reminders were sent.

The survey instrument included 17 questions designed to assess current climate and health training at degree-granting institutions of public health. Seven questions pertained to demographic characteristics of the institution and the individual completing the survey; the remaining 10 questions pertained to climate and health education and training offerings at the institution. Responses to the demographic characteristics were mandatory and responses to the remaining 10 questions were optional, some of which were conditional (based on answers to previous questions).

The eight climate and health competency domains were established by performing a comparison between existing climate and health competencies for public health professionals suggested in the peer-reviewed literature and those endorsed by regional health associations.[242–246] For each of the competencies, survey participants were asked to provide ratings ranging from 0 to 10 (representing levels of Factual Knowledge, Decision Making, and Action) regarding the degree of knowledge and skills students attain in climate and health within each of the eight core domains. (Table 73).

Table 74: Competency domains for public health professionals related to the climate crisis.

**Domain**	**Competency description**
Domain A	Fundamental science behind the natural and anthropogenic changes in the environment and associated health outcomes for given exposures.
Domain B	Demographics, economic development, technology, and other drivers/ activities that create pressures on the climate and environment.
Domain C	Use of research, tracking, monitoring, and surveillance to assess future health risks from climate and environmental change and the adaptive capacity of a system to cope.
Domain D	How biological, social, economic, and structural determinants of health synergise with climate exposures to amplify health risk and vulnerability for individuals, communities, and health systems.
Domain E	Strategies for health systems to mitigate, adapt, and build resilience to climate and environmental change.
Domain F	Assessment of adaptation solutions at population level with accompanying evaluation of health co-benefits.
Domain G	Solicit and receive stakeholder and community input to inform communication strategies, taking into consideration theories of behavioural change and existing cultural and political challenges.
Domain H	Work collaboratively in transdisciplinary and interprofessional climate and health initiatives.

The survey instrument was reviewed and vetted by all regional associations involved in administering the survey in addition to Working Group 2 of the Global *Lancet* Countdown. The survey was deemed exempt by the Columbia University Institutional Review Board. The survey was translated into Spanish and Portuguese prior to dissemination.

Survey responses were collected in Qualtrics and then imported into RStudio for analysis. Responses were first filtered based on the provision of climate and health education by the institution, with non-respondents removed. Duplicate entries were checked manually, and the most complete form was used.

Institutions' responses were analysed by grouping them according to the United Nations Development Program Human Development Index, based on the physical location of the campus. Response rates were calculated based on the number of complete survey responses and the number of contacted institutions. Additionally, quantitative data provided by respondents was summarized and qualitative data was counted. Whenever possible, data were converted into percentages to make comparisons between groups more meaningful.

To evaluate the degree of knowledge and skills students attain in climate and health across the eight core domains, the mean of responses for all four of the degree programs (doctoral, masters, bachelors/undergraduate, and vocational) was calculated for each of the domain areas (A–H). The standard deviation (SD) was calculated across the same set of data using the formula for a population standard deviation. Both mean and SD were calculated using R 4.3.3. Responses indicating Not applicable were excluded from the mean and SD calculations. Additionally, a few responses had numbers greater than ten due to an initial survey error, which were adjusted to ten for the analysis and generation of Figure 16SM.

Data

- The data utilised for analysis is derived from survey responses provided by 279 degree-granting public health institutions between October 2023–March 2024.
- Human Development Index (HDI) at the United Nations Development Programme, Human Development Reports[215]

Caveats

The results from this survey provide valuable insights into the current state of climate and health education and training at public health institutions. However, there are several potential limitations inherent in the methodology. In this first-year survey, an exploratory census approach was adopted, however, the overall response rate was low, with only 22% of institutions responding from 81 countries. Among participating partner networks, fewer institutions were identified in countries with a low or medium Human Development Index (HDI) compared to those with a high or very high HDI. Moreover, institutions in countries with a low HDI exhibited lower response rates. Importantly, because the survey relies on voluntary participation, results may be biased towards responses from institutions already providing climate and health education and training, who may be more motivated to participate. Further, the indicator relies upon self-reported information from faculty or curriculum committee members. This introduces the potential for bias, as respondents may overestimate or underestimate the extent of climate and health education offered at their institution. Additionally, while efforts were made to enable global participation by partnering with regional public health associations, the limited language offerings of the survey instrument may pose barriers that hinder participation from certain regions or institutions.

Future form of the indicator

In subsequent years, the team aims to improve participation in the annual survey by increasing recruitment through our member networks and offering the survey in additional languages. Further, the team aims to undertake targeted outreach to public health training institutions in low and medium-HDI countries to ensure representative sampling. To achieve more balanced geographic representation, the intention is also to designate regional leads to undertake targeted outreach to public health training institutions. Lastly, a subsample of respondent institutions will be analysed yearly to identify trends and changes in education and training. In future years, we also plan to develop a similar survey to assess climate and health education and training in other health professions, including medicine and nursing.

Additional analysis

Overview of survey response rates and reported climate and health education and training

The survey was sent to 1251 public health degree-granting institutions across 138 countries, ultimately garnering responses from 279 institutions across 81 countries. Climate change education was reported in 70% (n=196) of responding institutions. The participation of institutions from low HDI countries was low, with only 14% (n=18) responding out of 131 invited, eight of which reported offering climate and health education. Responses from medium HDI countries accounted for 20% (n=20) of the invited institutions (n=99), with 13 offering such education. The combined response rate from countries with high and very high HDI was 24%, with 241 institutions responding out of 1021 invited (Table 75).

Table 76: Survey response rates and reported climate and health education by HDI.

HDI	Number of institutions invited	Number of countries invited	Number of responding institutions	Number of responding countries	Response rate (%)	Based on responding institutions		
						Institutions reporting climate and health education, % (n)	Institutions reporting climate and health education as part of mandatory curricula, % (n)	Institutions reporting independent Climate and Health Concentration or Certificate program
Low	131	29	18	10	14%	44% (n=8)	22% (n=4)	0% (n=0)
Medium	99	23	20	9	20%	65% (n=13)	25% (n=5)	5% (n=1)
High	568	33	77	23	14%	65% (n=50)	32% (n=25)	5% (n=4)
Very high	453	53	164	39	36%	76% (n=125)	45% (n=74)	4% (n=7)
**TOTAL**	1,251	138	279	81	22%	70% (n=196)	39% (n=108)	4% (n=12)

Curricular approaches to climate and health education across degree programs

Survey respondents were asked to report how climate and health education and training are being incorporated into the curriculum at their institution within each degree program. Among 196 responding institutions with climate and health education, 298 degree-level programs were reported, among which 57% (n=171) currently have climate and health education integrated into the core curriculum, as shown in Table 77. Additionally, 31 programs currently offer specialisation in climate and health, through a certificate or concentration within a degree program. The majority of climate and health educational offerings were found within master's degree programs.

Table 78: Degree-level public health programs offering climate and health education and type of training offered.

Degree programs	Number of programs offering C+H training	Required offerings		Elective offerings		Climate and Health Concentration or Certificate
		Standalone required course	Part of the required core curriculum	Standalone elective course	Part of elective curriculum	
**Vocational**	27	9	13	7	11	7
**Undergraduate**	94	25	52	26	31	6
**Masters**	118	22	71	36	40	12
**Doctoral**	59	8	35	23	26	6
**TOTAL**	298	64	171	92	108	31

C+H: Climate and health

Mandatory climate and health education across degree programs

Among responding institutions, a greater proportion of students receive climate and health training in countries with low HDI (74% of students) compared to countries with high and very high HDI (44% and 36%, respectively). However, when assessed by the *total number* of students trained, more were trained in very high and high HDI countries (11,860 and 23,158, respectively) as compared to low HDI countries (5,901) (Table 79).

The majority of students enrolled at responding institutions with some form of climate and health education receive mandatory climate and health training (85%). Additionally, a larger number of students receive training in undergraduate (36%) and master’s degree (24%) programs compared to other degree programs (Table 80).

Table 81: Total number of students trained via mandatory curricular offerings across various degree programs by HDI.

UNDP HDI	Number of students enrolled at responding institutions n (%)	Students receiving C+H training (n)	Students receiving mandatory C+H training (n)	Degree program with C+H training (n)			
				Vocational	Undergraduate	Master's	Doctoral
**Low**	7,975 (8%)	5,901	5,198	1,000	1,655	1,498	1,045
**Medium**	5,880 (5%)	3,946	1,775	169	391	563	652
**High**	27,156 (26%)	11,860	9,750	1,939	3,863	1,876	2,072
**Very high**	64,274 (61%)	23,158	21,566	1,207	10,057	6,907	3,395
**TOTAL**	105,285 (100%)	44,865	38,289	4,315	15,966	10,844	7,164

C+H: Climate and health

Proficiency in climate and health knowledge and analytical skills across eight core domains

Institutions were requested to provide ratings, ranging from 0 to 10, representing the degree to which students attain proficiency in climate and health within the eight core domains. Ratings were correlated with a hierarchical framework for climate and health competency progression, with lower numbers representing competency at the bottom of the pyramid and higher numbers representing competency at the top of the pyramid (figure 135). These ratings provide insights into the ability of public health institutions to comprehensively prepare students to address climate-related health challenges.

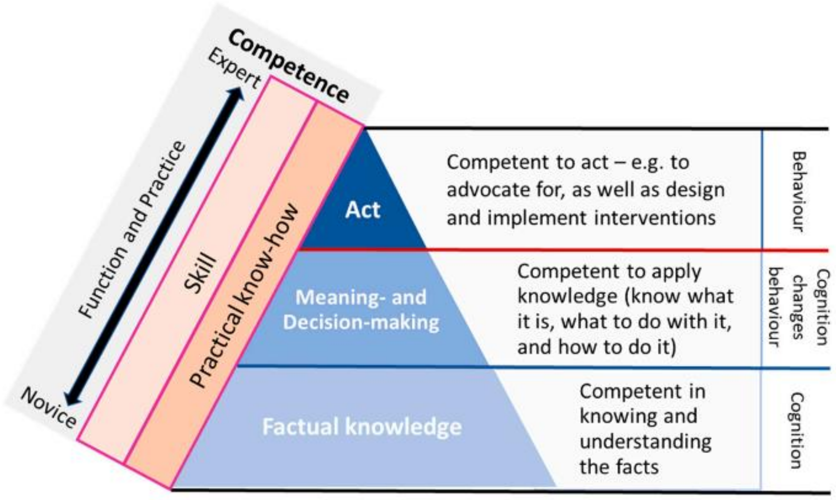

Figure 136: Hierarchical framework for climate and health competency progression.[37]

Out of the 298 degree-level programs, not all reported each of the eight domains in their curriculum. Table 64 shows the number of degree-level programs that offer the competencies across the eight domains. Overall, the mean rating across all degree-level courses indicated that decision-making level skills were generally achieved in most of the programs offering education on climate and health (Figure 89). However, it is noteworthy that the standard deviation was high, suggesting a degree of variability in the proficiency levels between institutions.

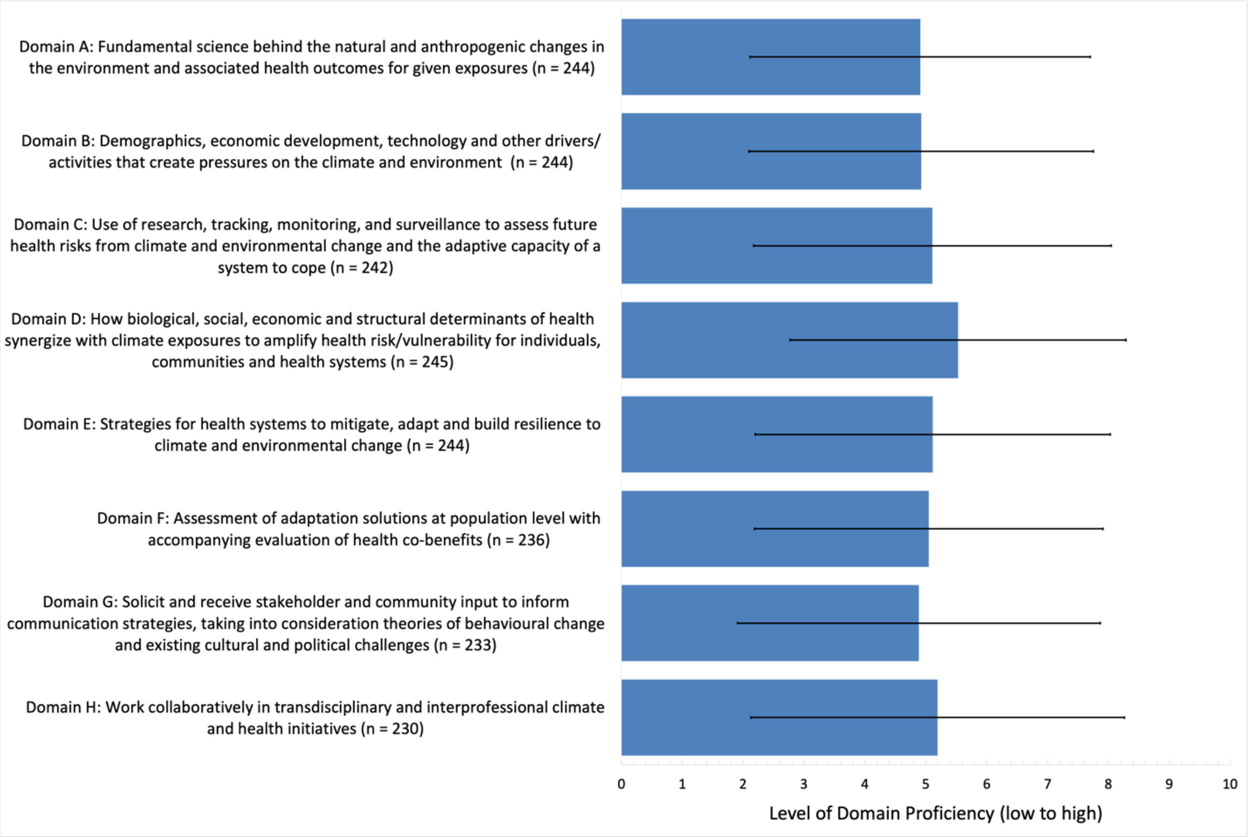

Figure 137: Assessment of climate and health education proficiency across eight core domains. Mean ratings are depicted by horizontal bars, with error bars indicating the standard deviation.

2.3: Vulnerabilities, health risk, and resilience to climate change

Indicator 2.3.1: vulnerability to severe mosquito-borne disease

Indicator authors

Prof Jan C. Semenza, Dr Yasna Palmeiro Silva

Methods

This indicator tracks the vulnerability of countries to severe adverse health outcomes from *Aedes*-borne diseases (Dengue, Chikungunya and Zika) considering urban population (UP) as a susceptibility variable and health care access and quality (HCAQ) as a coping capacity variable. The results are aggregated by Human Development Index (HDI), WHO region, and *Lancet* Countdown grouping. For the analysis, the period of consideration ranges from 1990 to 2023.
Vulnerability is computed by dividing the percentage of UP scaled from 1 to 100, by the percentage of a proxy of HCAQ scaled 1–100. HCAQ results from the subtraction of 100 - % of deaths by communicable diseases and maternal, prenatal, and nutrition conditions obtained the Global Burden of Disease Study 2019:

$$Vulnerability = \frac{sUP}{sHCAQ}$$

where sUP is scaled urban population, and $sHCAQ$ is scaled health care access and quality. The results have been scaled to display a vulnerability indicator that ranges from 0 to 100.

Data

- Global Burden of Disease Collaborative Network. Global Burden of Disease Study 2019 (GBD 2019) Results. Seattle, United States: Institute for Health Metrics and Evaluation (IHME), 2020. Available from https://vizhub.healthdata.org/gbd-results/. [Visited on 26 February 2024][247]
- World Bank, World Development Indicators. Urban population (% of total population). Available from: https://data.worldbank.org/indicator/SP.URB.TOTL.IN.ZS Urban population refers to people living in urban areas as defined by national statistical offices. The data are collected and smoothed by United Nations Population Division. [Visited on 26 February 2024][248]
- Human Development Index (HDI) at the United Nations Development Programme, Human Development Reports[215]

Caveats

HCAQ values have been updated from 2020 to 2023 by linearly extrapolating yearly estimates from 2019 data from the Global Burden of Disease Study 2019. The indicator is extrapolated to country level, no estimations at subnational level to differentiate vulnerability between rural and urban settings have been done. This extrapolation does not consider COVID-19 effect on communicable diseases.

Future form of the indicator

An improved version of this indicator will be developed in the future, incorporating other factors linked to vulnerability to dengue in the literature.

Additional analysis

Table 82: Relative change in scaled vulnerability from 1990–1999 to 2014–2023 by HDI group.

HDI	Relative change in scaled vulnerability from 1990-1999 to 2014-2023
Low	-46.02%
Medium	-32.15%
High	-2.17%
Very High	+5.36%

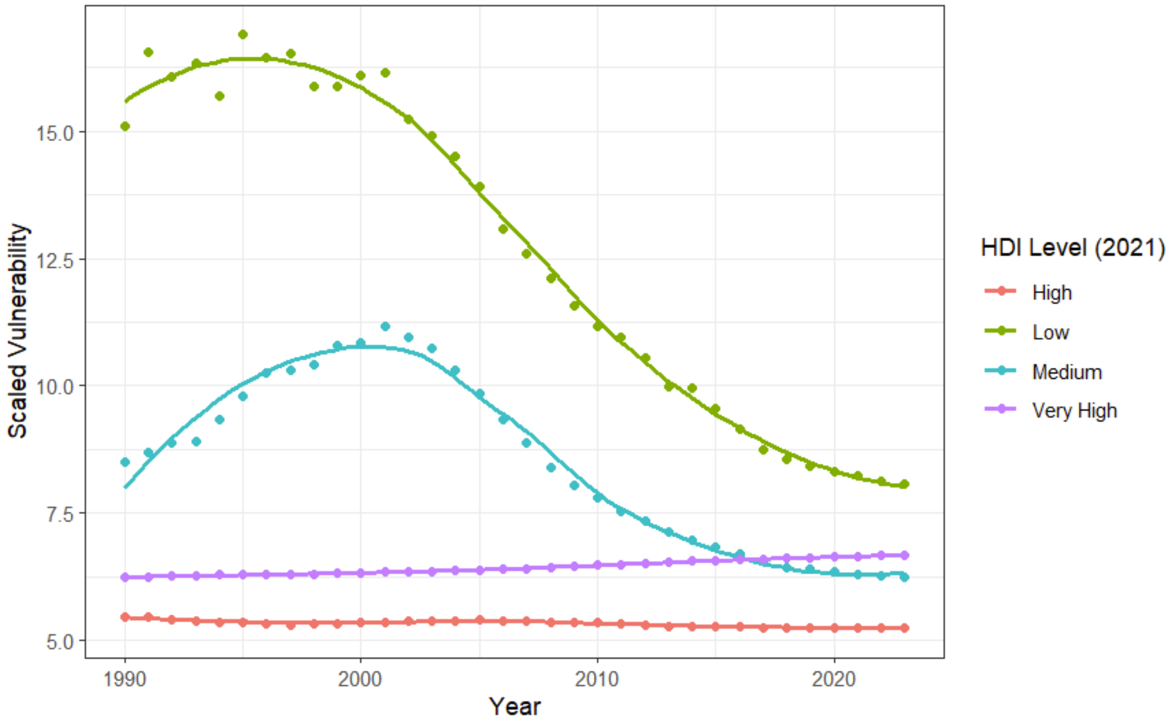

Figure 138: Global scaled vulnerability to mosquito-borne diseases, by HDI, from 1990 to 2023.

Table 83: Relative change in scaled vulnerability from 1990–1999 to 2014–2023 by WHO region.

WHO Region	Relative change in scaled vulnerability from 1990–1999 to 2014–2023
Africa	-42.01%
Americas	-5.97%
Eastern Mediterranean	-20.95%
Europe	+3.59%
South-East Asia	-12.67%
Western Pacific	+4.57%

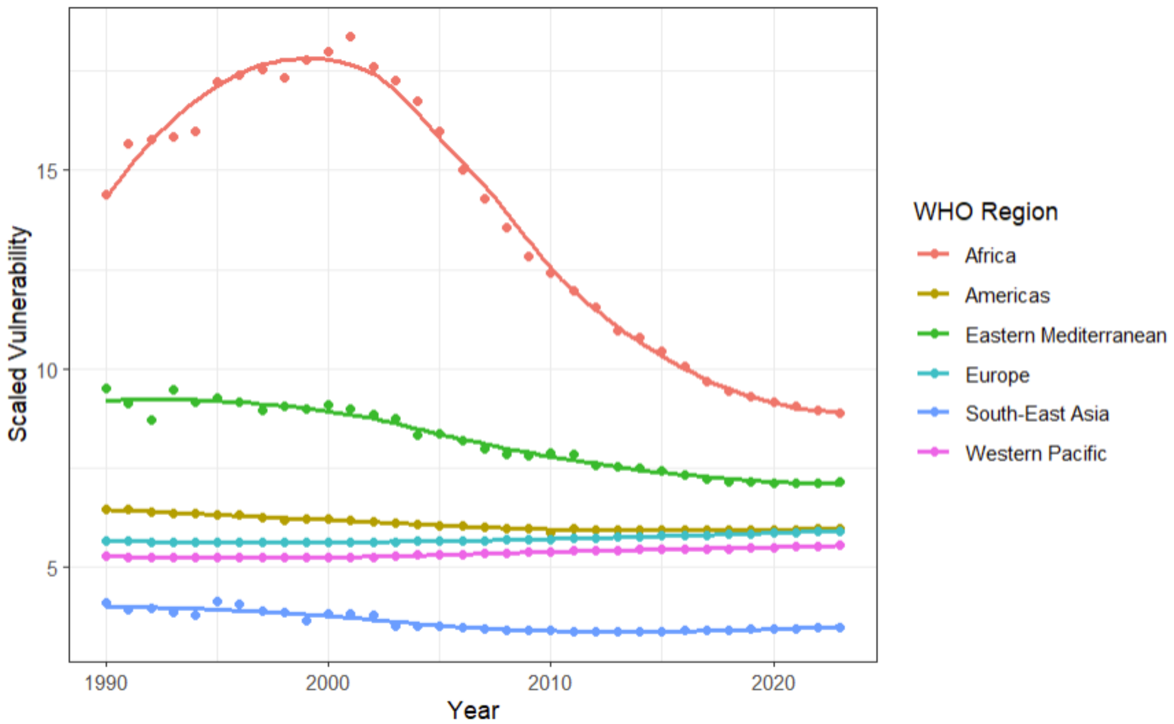

Figure 139: Global scaled vulnerability to mosquito-borne diseases, by WHO region, from 1990 to 2023.

Table 84: Relative change in scaled vulnerability from 1990–1999 to 2014–2023 by LC region.

**LC region**	**Relative change in scaled vulnerability from 1990–1999 to 2014–2023**
Africa	-42.32%
Asia	-4.12%
Europe	+6.07%
Northern America	+4.96%
Oceania	+1.08%
SIDS	-8.12%
South and Central America	-6.96%

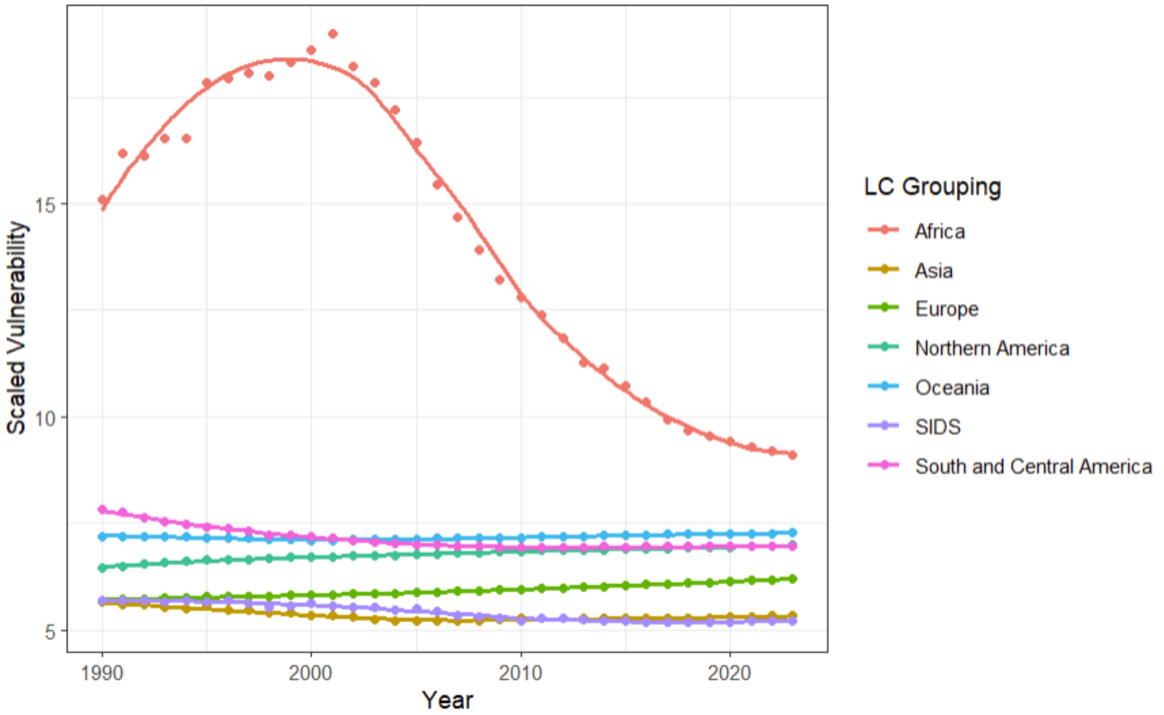

Figure 140: Global scaled vulnerability to mosquito-borne diseases, by LC region, from 1990 to 2023.

Indicator 2.3.2: lethality of extreme weather events

Indicator Authors

Prof Dominic Kniveton and Dr Yasna Palmeiro-Silva

Methods

Data from the EM-DAT database[249] were combined with those from the 2021 WHO Health and Climate Change Survey Report[213] to explore trends in mortality rates associated with disasters involving floods or storms in countries with climate-informed health early warning systems (HEWS). Analysis was restricted from 2000 following the recommendations by the EM-DAT team in September 2023 to exclude pre-2000 data from trend analyses based on their data (https://doc.emdat.be/docs/known-issues-and-limitations/specific-biases/).[250]

From EM-DAT database, deaths, as proxy of the lethality of weather-related disasters, are defined as the number of people who lost their life because the disaster happened. People affected are defined as those requiring immediate assistance during a period of emergency; requiring basic survival needs such as food, water, shelter, sanitation, and immediate medical assistance.

According to WHO, HEWS *"involves integrating multiple surveillance systems (e.g., disease surveillance and weather surveillance) to improve the use of information for detecting, investigating, and responding to public health threats. This integration of data, therefore, improves the flow of surveillance information throughout the health system".* [213] Most commonly, countries have climate-informed health surveillance systems for: vector-borne diseases, 39% of countries (30 out of 78); waterborne diseases, 32% of countries (25 out of 78); or airborne and respiratory diseases, 35% of countries (23 out of 65). Few countries have climate-informed health surveillance systems in place for

malnutrition and foodborne diseases, 11% (eight out of 70 countries); zoonoses, 21% (14 out of 66 countries); and mental and psychosocial health, 13% (six out of 47 countries).[251]

Only countries reporting on their status of implementation of HEWS for floods and storps participating in the 2021 WHO survey and with HDI classification available were included in the analysis. These included:

- Countries that reported having implemented a HEWS: Azerbaijan, Bhutan, Brunei Darussalam, Canada, China, Croatia, Cuba, Czechia, El Salvador, Ethiopia, Germany, Guatemala, Israel, Lithuania, Madagascar, Marshall Islands, Netherlands, North Macedonia, Oman, Palau, Poland, Portugal, Sao Tome and Principe, South Africa, Sri Lanka, Sweden.[251]
- Countries that reported not having implemented a HEWS: Argentina, Bahamas, Belize, Bolivia (Plurinational Bolivia State of), Brazil, Bulgaria, Cambodia, Cameroon, Cabo Verde, Costa Rica, Côte d'Ivoire, Dominican Republic, Egypt, Eritrea, Estonia, Ghana, Guyana, Haiti, Jamaica, Jordan, Kenya, Lebanon, Malawi, Mozambique, Nicaragua, Paraguay, Saint Kitts and Nevis, Saint Lucia, Serbia, Seychelles, Sierra Leone, Slovakia, Suriname, Turkmenistan, United Republic of Tanzania, United States of America, Uruguay, Vanuatu, Yemen, Zambia.[251]

Analyses of observed data were conducted, comparing mortality rate per event per year for the 2000-2009 and 2014-2023 periods by HDI country group and reported implementation or not of HEWS. Complementarily, Generalized Linear Models (GLMs) with a Poisson distribution were fitted in order to evaluate differences between HDI groups. In this case, the outcome variable was the number of deaths per event, and the independent variables included: year as a continuous variable and HDI (Very High, High, Medium, Low). To account for differences in population size in time and across countries, population size was included in the model as an offset. The Poisson GLM was specified as follows:

$$\log(Deaths_i) = \beta_0 + \beta_1 * Year_i + \beta_2 * HDI_i + \log(Population_i)$$

Where $Deaths_i$ represents the number of deaths in event i, $Population_i$ is the population size used as an offset, and β_0, β_1, β_2 are the coefficients estimated by the model.

All analyses were performed using R Statistical Software version 4.3.2. Native functions and the R package MASS were used.

Data

- EM-DAT at the Centre for Research on the Epidemiology of Disasters (CRED) at the Université Catholique de Louvain, Belgium[249]
- Human Development Index (HDI) at the United Nations Development Programme, Human Development Reports[215]
- 2021 WHO Health and Climate Change Global Survey.[213]

Caveats

The EM-DAT database contains a number of possible biases. Firstly, there is a possible bias in missing some disaster events because of under-reporting. EM-DAT classifies an event as a disaster if

10 or more people die; 100 or more people are affected; there is a declaration of a state of emergency; or a call for international assistance. Similarly, there are likely biases in how countries report both the number of deaths and people affected. Numbers of deaths for example may not include mortality from the cascading risks of natural hazards or those that occur as a result of longer causal chains from the hazard. Secondly, estimates of the numbers of people affected have different biases for different countries because of how the concept of affected people is defined. This must be considered when comparing countries.

The combination of two different datasets may not be fully compatible as the HEWS from the 2021 WHO survey does not have a clear starting implementation year.

Additional analysis

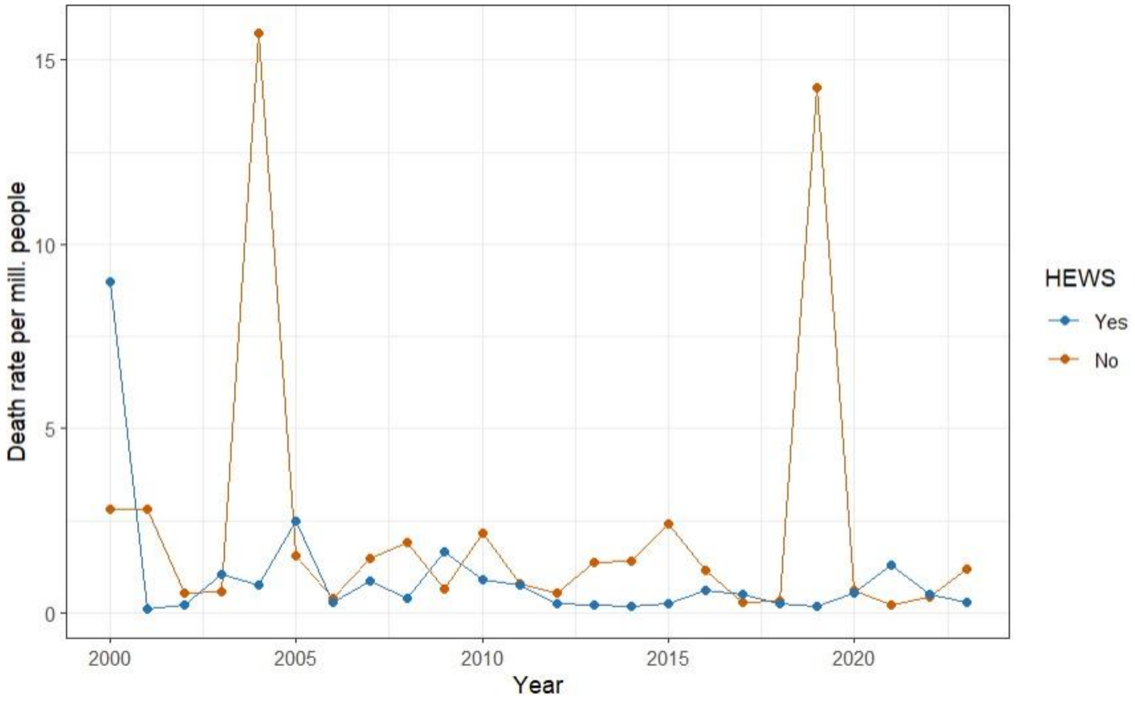

Figure 141: Death rate per flood or storm related disaster by countries with and without HEWS

.

Indicator 2.3.3: rising sea levels, migration, and displacement

Indicator Authors

Dr Sonja Ayeb-Karlsson, Dr Shouro Dasgupta, Prof Ilan Kelman, Prof Celia McMichael

Methods

The methodology for this indicator remains similar to that described in the 2023 report of the Lancet Countdown.[11] Using a bathtub model, the indicator overlays future Global Mean Sea Level Rise (GMSLR) of 1m with coastal elevation value grid-cells to delineate areas of potential inundation and current global population distribution grid-cells to delineate populations living in areas exposed to absolute GMSLR of 1m.

In the first step, the Coastal Digital Elevation Model (CoastalDEM) dataset was used to categorise inundated grid-cells under 1m of GMSLR[252] In the second step a gridded population dataset[253] was overlaid to estimate population exposure values. These grid-cells were then matched with country boundaries using the Global Administrative Areas (GADM) Dataset (version 4.0.4). Grid-cell level data were then aggregated to country level (i.e. national population numbers exposed to 1m of GMSLR).

Data

- GMSLR: Estimated global mean increases in sea-levels.[254]
- Elevation: Coastal Digital Elevation Model (CoastalDEM)[252]
- Hybrid gridded demographic data for the world.[253]
- Global Administrative Areas (GADM) version 4.0.4, http://www.gadm.org/

Caveats

Global mean sea level increased by 0.20m between 1901 and 2018 and, depending on emission scenarios and environmental responses, is projected to rise 0.28–1.88m by 2100 (relative to 1995-2014) with significant variations at local and regional scales.[252,255,256] Due to uncertainty in the Greenland and Antarctic ice sheet melt dynamics, high-end GMSLR by 2100 under very high greenhouse gas emission scenarios cannot be ruled out[255,257,258] This indicator does not consider relative sea-level change due to the additional impacts of glacial-isostatic adjustment and delta subsidence.[258]

Estimates of population exposure to GMSLR vary according to datasets, timeframes, emission and socioeconomic scenarios, and analytical method.[259] This indicator uses CoastalDEM (3-arc second; 90m), a global coastal digital elevation model that is adjusted to reduce Shuttle Radar Topography Mission (SRTM) error.[252] While SLR-related hazards could potentially displace people living in sites of coastal risk, population exposure to SLR is not a proxy indicator for population displacement. Displacement can be prevented or forestalled through coastal protection (e.g., revegetation) and accommodation (e.g., land use planning) measures; some may be unable or unwilling to leave; and people migrate into low-lying coastal sites.[260,261] When protection and accommodation are exhausted or not feasible, relocation and retreat from sites of SLR-related risk may occur albeit influenced by broader economic, social, political and demographic processes.[262] Empirical studies identify diverse health consequences of relocation and retreat from sites of coastal risk, including consequences for mental health, food security, water supply, sanitation, infectious diseases, injury, and health care access.[263]

Future form of the indicator

As new, higher spatial resolution and more precise datasets become available, this indicator will be updated to produce robust estimates of population exposure to future GMSLR.

Additional analysis

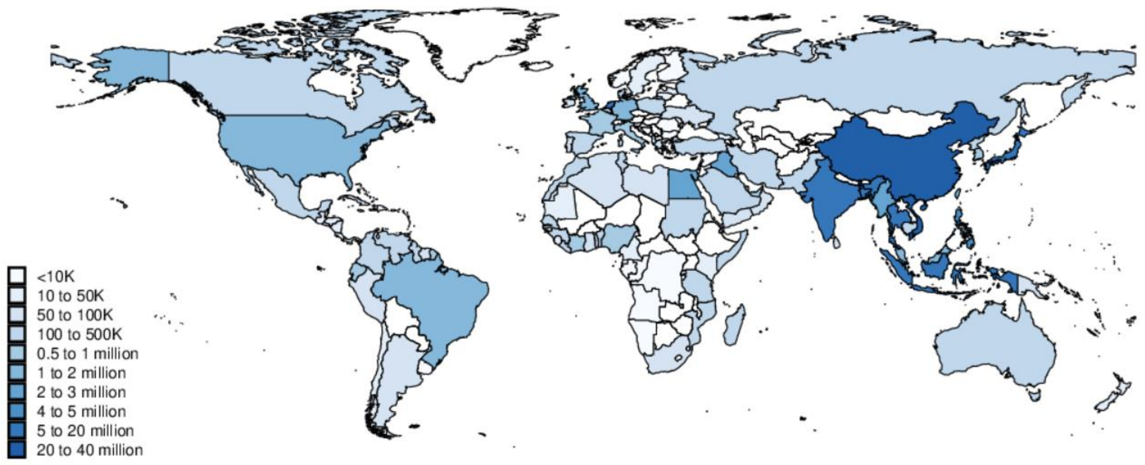

Figure 142: Population living less than 1 metre above current sea levels by country.

National Policies on Migration

Methods

The methodology for this indicator remains similar to that described in the 2023 report of the *Lancet* Countdown .[11] This component of this indicator on national policies reports:

1a. The number of currently valid national-level policies including legislation for migrants, migration, displacement, displaced people, relocation, and relocated people specifically related to climate change (not climate or disasters), including immobility (trapped populations/non-migration/non-displacement).

1b. The number of such policies mentioning health or well-being along with a qualitative analysis of how health and/or well-being are/is mentioned.

2a. The number of countries with at least one such policy.

2b. The number of such countries whose policies mention health or well-being along with a qualitative discussion of how health or well-being is mentioned.

"Country" refers sovereign state or autonomous non-sovereign territory (not just a sub-national jurisdiction). Multi-lateral, inter-governmental, and international policies are specifically excluded. Explicit mentions of "climate change" and "health" or "well-being" must be present, not implied definitions or references to wider contexts which might (or might not) encompass these points, e.g., "climate", "climate disasters", "humanitarian", and "environment".

The method for identifying national-level policies is:

1. A systematic review, using the keywords which define the indicator

2. Crowd-sourcing and expert queries.[264]

Because this search can never know what might have been missed, the numbers reported for this indicator represent minimum counts. Each policy included is also categorised by:

1. (a) Migration/mobility/displacement/relocation from a location,

   (b) migration/mobility/displacement/relocation to a location, and

   (c) immobility/trapped populations.

2. (a) Domestic migration/mobility/displacement/relocation and

(b) international migration/mobility/displacement/relocation – all immobility, by definition, is domestic

A given policy might be counted in more than one category for 1abc and for 2ab. Some policies do not have an end date, and some do, with both included. Policies which are now out-of-date are retained in a separate list as well as a list of policies considered but not included in this indicator.

Caveats

As documented in previous *Lancet* Countdown reports and supporting publications,[259,265] the main problems with using migration or displacement as a climate change and health indicator are:

1. Attributing movement or immobility to climate change or climate change impacts is not straightforward
2. Attributing health outcomes to movement or immobility is not straightforward.

These attribution relationships are debated, including whether or not: (i) there are or will be links between climate change and migration, displacement, (im)mobility, relocation; and (ii) there are or will be links between migration, displacement, (im)mobility, relocation and health/wellbeing.

This indicator assists in overcoming the attribution problem by:

1. Examining written policies, so attribution is not a concern, because the policies exist, even if attribution is inappropriate
2. Examining how policies mention health/well-being, so again actual attribution is not a concern, because the text on health or well-being either exists or does not exist, even if attribution is inappropriate

If spurious attributions are made in the policies between (i) climate change and migration/displacement/immobility or (ii) migration/displacement/immobility and health or well-being, then this indicator can analyse those attributions and why they might not be defensible, based on the scientific literature. Thus, this indicator provides what is happening at the national level and the appropriateness of these policies in terms of the scientific literature. The key to this approach and to overcoming the caveats is keeping the indicator simple and straightforward, which is why the indicator has been designed in the proposed manner.

Selecting policies, and in particular national policies, does not cover all possibilities, but it serves as an indicator. As well, it is an indicator of how national governments perceive the climate change / (im)mobility / health links, without making a statement on the actual links, which the literature explains is exceptionally difficult. This approach to the indicator also means that misattributions are easily filtered out, such as reporting migration and health links to disasters or climate, both of which are different from links to climate change. Using ‘climate change’ synonymously with ‘climate’, ‘climate-related disasters’, and/or ‘disasters’, is a common mistake in many policies reviewed as well as in the academic literature.

The main caveat is that most of the data is confined to documents in English, with a few other languages on occasion. The advantage is that policies which are not available in English have typically been discussed in English publications, including blogs and news reports, suggesting that much relevant material has been captured. Nonetheless, the numbers reported can only be taken as the

minimum, as in ‘at least so many’ policies match the criteria stated. One minor caveat is that the number of countries sometimes changes year-to-year, providing a different baseline. These changes are rarely more than one or two countries per year out of a sample of around 200. Substantial changes to the numbers of countries will be reported if this occurs.

Future form of the indicator

The indicator design helps in overcoming these caveats by reporting that the counts provided must be only minimum numbers, because we cannot know what we would have missed. Through publicity, publication, crowd sourcing, and expert connections, this limitation will be overcome because people will provide examples of what we missed. As an indicator, it is important to accept that the numbers are not comprehensive but provide only minimum numbers as a lower-bound baseline.

Section 3: Mitigation Actions and Health Co-Benefits

Section Lead: Prof Ian Hamilton
Research Fellow: Dr Shih-Che Hsu

3.1: Energy use, energy generation, and health

Indicator 3.1.1: energy systems and health

Indicator authors

Dr Harry Kennard, Dr Shih-Che Hsu

Carbon Intensity of the Energy System

Methods

This indicator contains two components:

- Carbon intensity of the energy system, both at global and regional scales, (1971–2021), in tCO_2/TJ
- Global CO_2 emissions from energy combustion by fuel, in $GtCO_2$ (1972–2020). Global emissions without fuel breakdown are also provided for 2022 and provisionally for 2023.

The technical definition is the tonnes of CO_2 emitted for each unit (TJ) of primary energy supplied. The rationale for the indicator choice is that carbon intensity of the energy system will provide information on the level of fossil fuel use, which has associated air pollution impacts. Higher intensity values indicate a more fossil dominated system, and one that is likely to have a higher coal share. As countries pursue climate mitigation goals, the carbon intensity is likely to reduce with benefits for air pollution.

The indicator is calculated based on total CO_2 emissions from fossil fuel combustion divided by Total Energy Supply (TES). TES reflects the total amount of primary energy used in a specific country, accounting for the flow of energy imports and exports.

The data is available for most countries of the world, for the period 1971–2021.

Data

- This indicator is based on based on the IEA dataset, CO_2 Emissions From Fuel Combustion: CO_2 Indicators, accessed via the UK data service,[266] and supplemented with additional data for 2023.[267]

Caveats

IEA data are generated using both direct input from national governments and modelling. As such, while they represent the best available data on national CO_2 emissions from fuel, they are subject to caveats which vary by energy commodity and country. Full details are given in the CO_2 Emissions From Fuel Combustion documentation.[268]

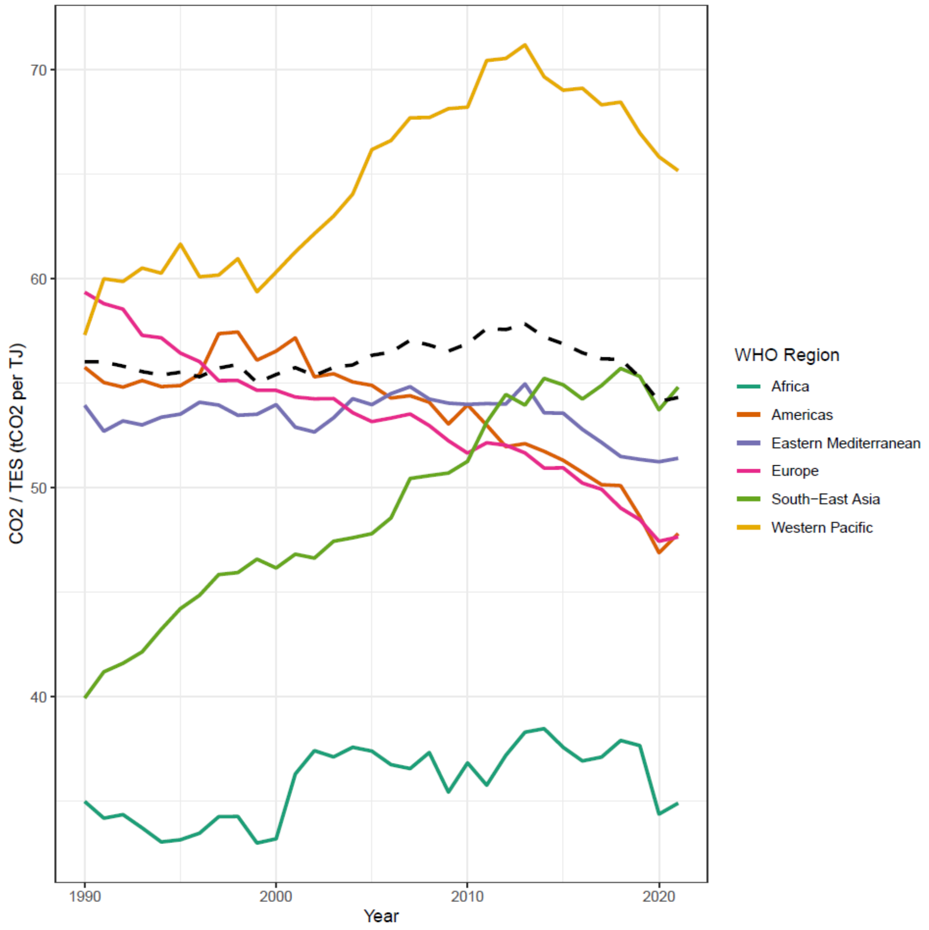

Figure 143: Carbon intensity of the energy system by WHO region, 1971–2021 (tCO_2/TJ)

Coal Phase-Out

Methods

Two indicators are used here:

1. Total primary coal supply by region / country (in exajoules, EJ);
2. Share of electricity generation from coal (% of total generation from coal) and global generation from coal (in TWh)

These indicators are important to enable tracking of changes in coal consumption at a regional and country level. Due to the level of coal used for power generation, a second indicator tracks the contribution to electricity generation from coal power plants in selected countries. As countries pursue climate mitigation goals, the use of coal is likely to reduce with resulting benefits for air pollution. The indicator on primary energy coal supply is an aggregation of all coal types used across all sectors (from the IEA energy balances). The data are available for most countries of the world, for the period 1978–2021.

The indicator on the share of electricity generation from coal is estimated based on electricity generated from coal plant as a percentage of total electricity generated. Regional data are available from 1990–2021; pre-1990 data are not used due to incomplete time series.

Countries or regions with large levels of coal use (as a share of generation, or in absolute terms), have been selected to show in the figures.

The following types of coal are added to produce the total primary coal supply: ‘Anthracite’, ‘Coking coal’, ‘Lignite’, ‘Other bituminous coal’, ‘sub-bituminous coal’

Data

1. This indicator is based on the extended energy balances from the International Energy Agency. The specific dataset is called World Extended Energy Balances (for 2023), and is sourced via the UK data service.[269]

Caveats

IEA data are generated using both direct input from national governments and modelling. As such, they are subject to caveats which vary by energy commodity and country. Full details are given in the IEA World Energy Balances documentation.[270] This documentation also covers changes to methodology in previous editions of IEA World Energy Balances. A typical example of the way data can be impacted by methodology updates by reporting countries is as follows, relating to Belgium ‘New data on consumption cause breaks in time series for primary solid biofuels between 2011 and 2012’. However, since data are aggregated, the impacts on overall trends is minimal.

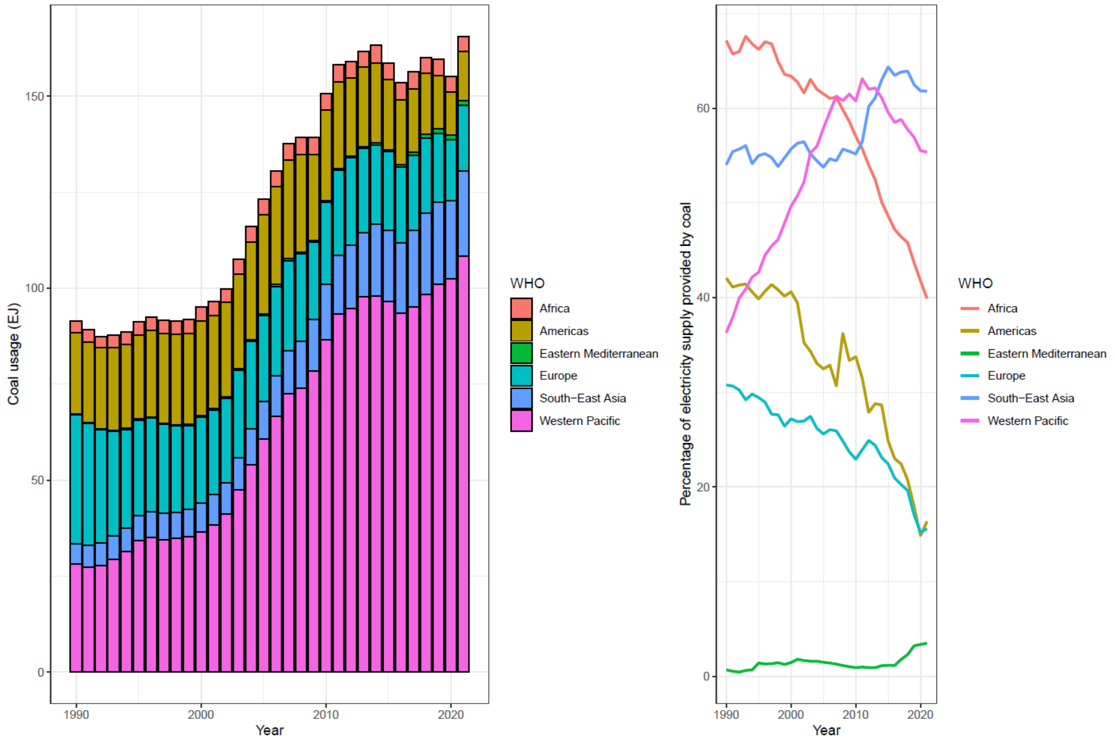

Figure 144 Left: Coal use by WHO region, 1990–2020 (EJ) and right: share of electricity supply provided by coal by WHO region, 1990–2020 (%)

Zero-Carbon Emission Electricity

Methods

Two indicators are used here, and presented in two ways:

1. Total low carbon electricity generation, in absolute terms (TWh) and as a % share of total electricity generated (to include nuclear, and all renewables); and
2. Total modern renewable generation (wind and solar), in TWh, and as a % share of total electricity generated

The increase in the use of low carbon and renewable energy for electricity generation will push other fossil fuels, such as coal, out of the mix over time, resulting in an improvement in air quality, with benefits to health.
The renewables (wind and solar) indicator has been used to allow for the tracking of rapidly emergent renewable technologies. For both indicators, generation, rather than capacity, has been chosen as a metric as the electricity generated from these technologies is what actually displaces fossil-based generation. Countries with large levels of low carbon generation (as shares, or in absolute terms), or with higher fossil dependency, have been selected.

The data are taken from the IEA extended energy balances.[269] The absolute level indicators are total gross electricity generated aggregated from the relevant technology types. The share indicators are estimated as the low carbon or renewable generation as a % of total generation.

The data are available for most countries of the world, for the period 1971–2021. Only the period from 1990 has been used, due to data gaps for selected countries prior to 1990.

The following IEA variable names are added to produce total low carbon electricity generation: ‘Nuclear, ‘Hydro’, ‘Geothermal’, ‘Solar photovoltaics’, ‘Solar thermal’, ‘Tide, wave and ocean’, ‘Wind’

The following IEA variable names are added to produce total modern renewable electricity generation:
‘Geothermal’, ‘Solar photovoltaics’, ‘Solar thermal’, ‘Tide, wave and ocean’, ‘Wind’

Data

- This indicator is based on the extended energy balances from the International Energy Agency. The specific dataset is called World Extended Energy Balances, and is sourced via the UK data service (http://stats.ukdataservice.ac.uk/).[269]

Caveats

IEA data are generated using both direct input from national governments and modelling. As such, they are subject to caveats which vary by energy commodity and country. Full details are given in the IEA World Energy Balances documentation.[270] This documentation also covers changes to methodology in previous editions of IEA World Energy Balances. A typical example of the way data can be impacted by methodology updates by reporting countries is as follows, relating to Belgium

‘New data on consumption cause breaks in time series for primary solid biofuels between 2011 and 2012’. However, since data are aggregated, the impacts on overall trends is minimal.

Additional analysis

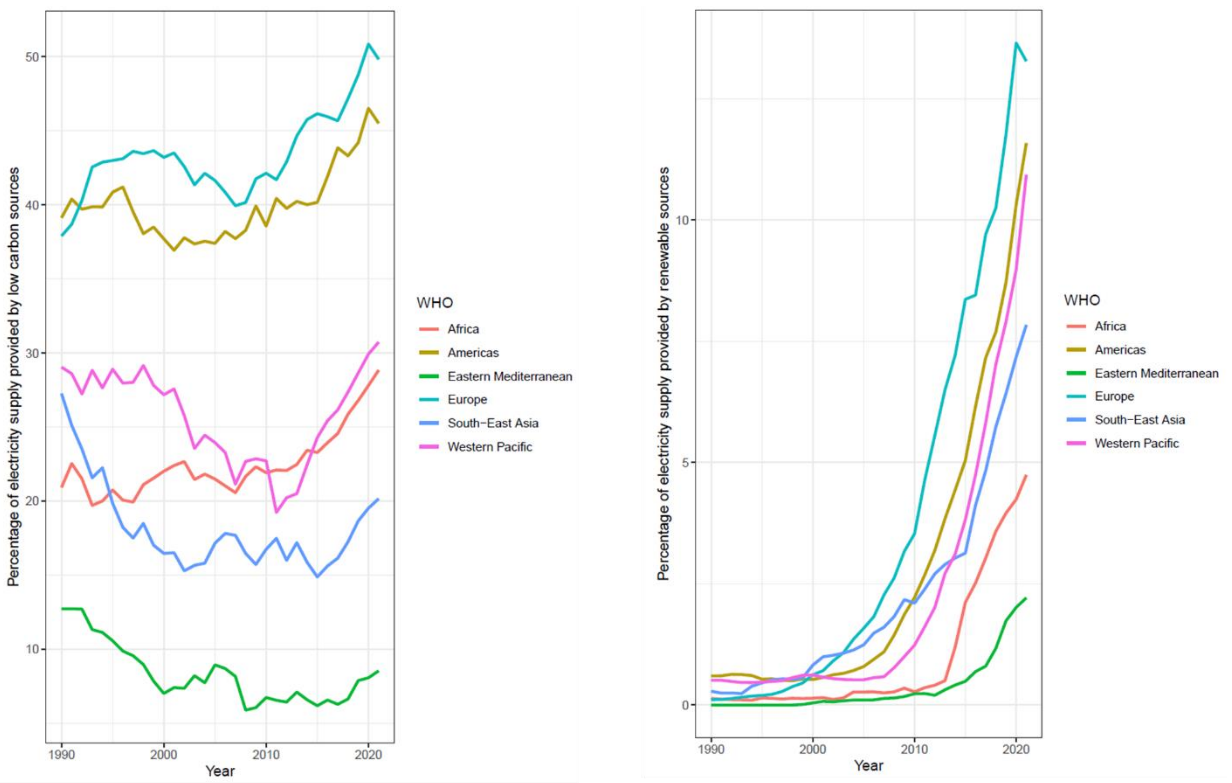

Figure 145: Left: Share of total energy supply provided by low carbon energy sources by WHO region, 1990–2020. Right: Share of electricity generation provided by modern renewables (wind, solar and geothermal) by WHO region, 1990–2020

Indicator 3.1.2: household energy use

Indicator authors

Luciana Blanco-Villafuerte, Prof Ian Hamilton, Prof Stella Hartinger, Dr Harry Kennard

Methods

Access to clean energy is defined by the IEA (2020) as:
"a household having reliable and affordable access to both clean cooking facilities and to electricity, which is enough to supply a basic bundle of energy services initially, and then an increasing level of electricity over time to reach the regional average".[271]

The use of energy in the residential sector is drawn from the IEA extended global residential modelling produced in the World Energy Outlook from the ‘World Extended Energy Balances’ 2023 edition, which covers all countries or major regions in the world.[269] The values are measured in EJ and

cover all fuels supplied for consumption within the residential sector (IEA flow code QGFLOW076) final energy demand.

The specific IEA variables were combined in the following way:

- `Solid biofuels` = Charcoal + `Primary solid biofuels`
- `Coal, coke and peat` = `Hard coal (if no detail)` + BKB + `Petroleum coke` + `Patent fuel`+`Coke oven coke`+`Brown coal (if no detail)`+Peat+`Gas coke`+`Peat products`+`Coking coal`+`Sub-bituminous coal`+`Other bituminous coal`+Lignite+Anthracite+Bitumen
- `Other biofuels` = `Other liquid biofuels` + Biogasoline + `Non-specified primary biofuels and waste`+`Biogases`+`Biodiesels`
- `Liquid fossil fuels` =`Paraffin waxes`+`Other oil products`+`Naphtha`+ `Gas/diesel oil excl. biofuels` + Lubricants + `Natural gas liquids`+ `Other kerosene`+`Liquefied petroleum gases (LPG)`+`Fuel oil`+`Motor gasoline excl. biofuels`+`Crude oil`
- `Waste & other` = `Municipal waste (non-renewable)`+`Municipal waste (renewable)`+`Industrial waste`+`Refinery gas`+ `Blast furnace gas`+`Gas works gas`+`Coke oven gas`+`Oil shale and oil sands`,
- Finally, Natural gas, Heat, Solar thermal, Geothermal and Electricity variables were provided directly from IEA flow QGFLOW076.

Data

- Healthy fuels for cooking were provided by the WHO[272,273]
- The additional energy usage and access is based on data from the IEA World Energy Balances 2023.[269]

Caveats

The data from the IEA on residential energy flows and energy access provide an indication of both the access to electricity and the proportion of the different types of energy used within the residential sector. These provide an important picture on how access and use might be interacting.
IEA data are generated using both direct input from national governments and modelling. As such, they are subject to caveats which vary by energy commodity and country. Full details are given in the IEA World Energy Balances documentation.[270] This documentation also covers changes to methodology in previous editions of IEA World Energy Balances. A typical example of the way data can be impacted by methodology updates by reporting countries is as follows, relating to Belgium 'New data on consumption cause breaks in time series for primary solid biofuels between 2011 and 2012'. However, since data are aggregated here by HDI level, the impacts on overall trends is minimal.

Future form of the indicator

The WHO are in the process of updating the household energy survey database which underpins this indicator. Future forms of the indicator may be able to be coupled more directly with the negative health outcomes related to the use of dirty fuels in the home.

Indicator 3.1.3: sustainable and healthy road transport

Indicator authors

Dr Harry Kennard, Dr Melissa Lott

Methods

Fuel use data (by fuel type) from the IEA World Extended Energy Balances are divided by corresponding population statistics from the United Nations, Department of Economic and Social Affairs, Population Division (Figure 146).

The fuel flows from the IEA are combined in the following way:

- Biofuels = Biodiesels +Biogasoline +Biogases + Other liquid biofuels
- Fossil fuels = Natural gas liquids+Natural gas+Motor gasoline excl. biofuels+Liquefied petroleum gases (LPG)+Refinery gas+White spirit & SBP+Kerosene type jet fuel excl. biofuels+Gas/diesel oil excl. biofuels+Lubricants+Naphtha+Fuel oil+Other kerosene+Other oil products+Bitumen

Electricity is given by the existing IEA total.

Totals for a given year and country are then divided by the corresponding country population, and then summed to produce the final estimate. This avoids including the population of the countries that are not covered by the IEA.

Data

- Fuel use data is from the IEA, World Extended Energy Balances 2023.[269]
- UN Population estimates, 2019 edition.[274]

Caveats

This indicator captures change in total fuel use and type of fuel use for transport, but it does not capture shifts in modes of transport used. In particular, it does not capture walking and cycling for short trips, which can yield substantial health benefits through increased physical activity.[275] Alongside the fossil fuel combustion pollutants, tyre wear accounts for an estimated 3–7% of airborne $PM_{2.5}$ particulates worldwide.[276]

Future form of the indicator

An ideal fuel use indicator would capture the direct health impacts of the use of transport fuels, with country- and urban-level specificity within the global coverage. In turn, the co-benefits of transitioning to less-polluting fuels would be quantified directly in terms of reduced exposures to air pollution and their corresponding health impact.

To capture sustainable uptake more fully a future indicator could collate information on the proportion of total distance travelled by different modes of transport based on comprehensive local survey data. Other data on sustainable travel infrastructure, for instance the presence of cycle schemes, would also be useful. The data described below in the additional analysis section provided

from smartphone data serves to expand the picture provided by IEA data alone. Further development of data of this type may be possible in future reports.

Additional analysis:

Figure 147 shows the global per capita use of fuels for road transport, as well as the percentage energy provided by biofuels and electricity with the countries with the highest adoption of these fuels for road transport.

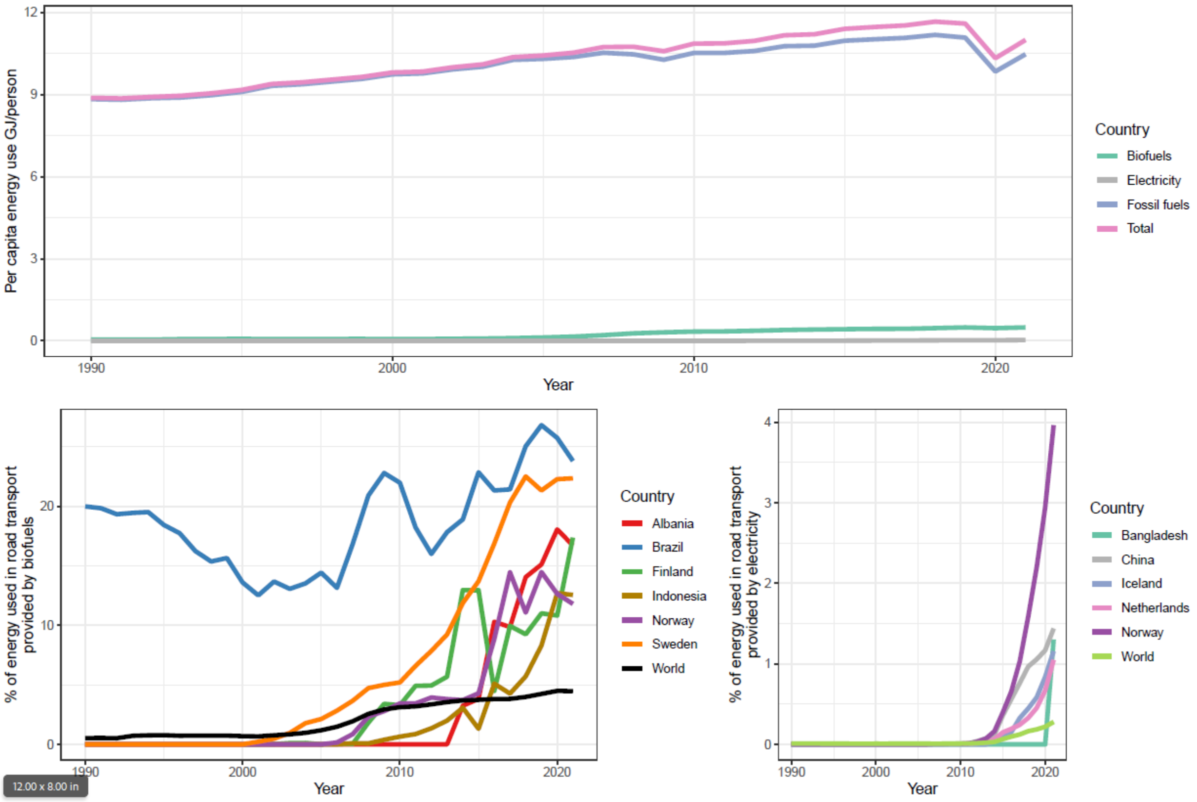

Figure 148: Top panel: Global per capita energy use on road transport by fuel (GJ/Person). Bottom panel: % of road transport energy provided by biofuels (left) and electricity (right) for select countries.

3.2: Air quality and health co-benefits

Indicator 3.2.1: mortality from ambient air pollution by sector

Indicator authors

Dr Gregor Kiesewetter, Dr Jessica Slater, Laura Warnecke

Methods

This indicator quantifies contributions of individual source sectors to ambient $PM_{2.5}$ exposure and its health impacts. Contributions from coal have been highlighted across all sectors.

Estimates of sectoral source contributions to annual mean exposure to ambient $PM_{2.5}$ were calculated using the GAINS model,[211] which combines bottom-up emission calculations with atmospheric chemistry and dispersion coefficients.

Energy statistics are taken from the IEA World Energy Statistics for 2015, from the IEA World Energy Outlook 2021[212] for 2020 and from the World Energy Outlook 2022[213] for 2021. Data on energy consumption in individual sectors are imported into GAINS, matching the sectors of the World Energy Statistics and downscaling to the 180 GAINS global regions. They are then merged with GAINS information on application of emission control technologies in each region and their emission factors to calculate emissions of $PM_{2.5}$ and its precursor gases SO_2, NOx, NH_3, and non-methane VOC.

Ambient $PM_{2.5}$ concentrations are calculated from the region and sector specific emissions by applying atmospheric transfer coefficients, which are a linear approximation of full chemistry-transport models. Atmospheric transfer coefficients in GAINS are based on full year perturbation simulations with the EMEP Chemistry Transport Model[214] at 0.1°×0.1° resolution (for low-level sources) / 0.5°×0.5° resolution (for all other sources) using meteorology of 2015. In Europe, the resolution is slightly different but the principle is the same. Calculations for Europe are described in detail by Kiesewetter et al. (2015),[215] calculations for the rest of the world are described by Amann et al.[216] Calculated ambient $PM_{2.5}$ concentrations have been validated against in-situ observations from the WHO's Urban Ambient Air Pollution Database (2018 update),[217] and other sources where available (e.g., Chinese statistical yearbook) and show in general good agreement with monitoring data up to urban background level (local variation at roadside stations is not captured by the resolution of a few kilometres).

In previous versions of the *Lancet* Countdown, deaths from total ambient $PM_{2.5}$ for regions other than Europe were calculated following the methodology of the Global Burden of Disease studies with updated exposure-response relationships consistent with those reported in the Global Burden of Disease 2019 study.[81] The MR-BRT curves were obtained from the public release site[219] and relative risks for six diseases IHD, COPD, stroke, lung cancer, ALRI, and type 2 diabetes calculated from them.

For this edition of the *Lancet* Countdown, new concentration-response functions (CRFs) were taken from updated analysis by Burnett et al. (2022),[277] which takes a meta-analysis of cohort studies and applies a fusion model to the log-linear function relating to $PM_{2.5}$ exposure with mortality. Particularly this model decreases the derivative responses at high $PM_{2.5}$ concentrations, which allows for the limiting of the attributable risk at extremely high $PM_{2.5}$ concentrations. The Fusion CRFs are given for all six mortality endpoints related to air pollution exposure indicated above (IHD, COPD, stroke, lung cancer, ALRI, and type 2 diabetes) as well as total non-communicable disease plus lower respiratory infection (NCD LRI). For the reported mortalities in this report, the NCD LRI CRFs were used as total all-cause mortality following the analysis presented in a recent paper by Lelieveld et al. (2023).[278] The mortality rate for each of the six disease endpoints was also calculated from the Fusion CRFs and these results were compared with those using the GBD 2019 exposure-response relationships. The updated Fusion CRFs resulted in a significant increase in attributable mortality beyond the numbers published in the previous editions of the *Lancet* Countdown which used the GBD 2019 exposure-response relationships. Table 86 in the additional analysis section show the differences between utilising the GBD exposure-response relationships and the Fusion-CRFs.

Disease and age specific baseline mortality rates are taken from the GBD Results database[221] and have been updated to the 2019 data. In this version of the *Lancet* Countdown, baseline mortality rates for each year from 2007 to 2021 were utilised in the calculation of attributable deaths meaning the calculations now account for changes in overall mortality rates for each specific disease endpoint. The shares of different diseases were applied to age-specific total deaths taken from UN World Population Prospects (2017 update);[222] for 2019, the statistics were interpolated linearly between 2015 and 2020. Attribution of estimated deaths from AAP to polluting sectors was done proportional to the contributions of individual sectors to population-weighted mean $PM_{2.5}$ in each country.

Data

- Energy: IEA World Energy Balances for 2015,[224] World Energy Outlook 2021 (for the year 2020),[212] World Energy Outlook 2022 (for the year 2021)[213]
- Other activities: Agricultural livestock data are based on FAO statistics and projections[225] and fertiliser use is based on data from the International Fertilizer Association
- UN World Population Prospects, 2017 update[226]
- Global Burden of Disease 2019 study,[218] MR-BRT curves obtained from the public release site[219]

Caveats

The indicator relies on model calculations which are inherently uncertain. The resolution of approximately seven to ten km is deemed appropriate for urban background levels of $PM_{2.5}$ but may underestimate exposure in case of strong local $PM_{2.5}$ increments. The meteorology year is fixed to 2015.

Uncertainty in the shape of integrated exposure-response relationships (IERs) make the quantification of health burden inherently uncertain.

Additional Analysis

To understand the impact of the updated Fusion CRFs on the mortality estimates, we calculate the results also using the CRF from the GBD 2019 study which were utilised in the previous versions of the *Lancet* Countdown. In the report, the total all-cause mortality due to air pollution exposure is presented (defined here as non-communicable disease [NCD] plus lower respiratory infection [LRI]). However, the GBD 2019 study details CRFs for the six disease endpoints only. In table 85, the total mortality estimates aggregated at the global level are compared, for all-cause mortality (defined here as the deaths from the six disease endpoints) using the updated Fusion-CRFs with the GBD 2019 CRFs used in the previous *Lancet* Countdown.

Table 86: Total mortality estimates aggregated at the global level for all-cause mortality using the updated Fusion-CRFs compared with the GBD 2019 CRFs

Million Premature Deaths	GBD 2019	Fusion 6 Diseases	Difference	Fusion NCD	Difference
2016	3.29	4.69	1.40	6.12	1.43
2021	3.42	4.92	1.50	6.41	1.50

These results show a large increase in total deaths from ambient air pollution using the Fusion CRFs compared to the GBD 2019 CRFs used in the previous versions of the *Lancet* Countdown. Utilising the Fusion CRFs increases the estimate of the total premature deaths by 1.4 million in 2016 and 1.5 million in 2021. There is also a larger estimate of attributable premature deaths from when all-cause mortality CRFs are used compared to summing the six-disease endpoints. These higher levels of premature deaths estimated for all-cause mortality are indicative of risks relating to other diseases outside of these six categories but for which air pollution exposure is a known risk factor. Premature mortality from exposure to fossil fuel based ambient air pollution calculated for this version of the Lancet countdown results in significantly lower estimates of premature mortality compared to a recent paper by Lelieveld et al. (2023) which utilises the same CRFs.[279] This is likely due to differences in how exposure to ambient $PM_{2.5}$ is calculated between this study and Lelieveld et al. (2023),[279] however as specific concentrations or exposures are not detailed within the paper, at this stage no specific comparison can be made.

Indicator 3.2.2: household air pollution

Indicator authors

Prof Michael Davies, Dr Shih-Che Hsu, Dr Nahid Mohajeri, Dr James Milner, Dr Jonathon Taylor

Methods

Existing estimates of global household air pollution attributable mortality from GBD and WHO are based on the frequency of use of different fuels in the population. These are presented relative to the outdoor air pollution estimates (e.g., the additional mortality caused by household fuels above that caused by outdoor air pollution). The new indicator complements this work via a method tailored for the *Lancet* Countdown process which can 1) link the health effects of household fuels to their role in climate change accounting for the GHG and $PM_{2.5}$ emissions, and 2) complement how outdoor air pollution mortality is estimated in the *Lancet* Countdown by using the same inputs, and 3) be updated yearly.

A Bayesian hierarchical $PM_{2.5}$ exposure model was developed using sample data of indoor concentration from an updated WHO Global HAP database,[272,280] while wood, crop residues, and dung is combined into the category of 'biomass' and LPG, Natural gas, and biogas into category of 'gas'.[273] Variables were selected from monitored data available in 282 peer-reviewed studies covering the years 1996 to 2021 to develop Bayesian models for the $PM_{2.5}$ indoor concentration (sample size, n=315). Bayesian hierarchical models were built to generate accurate $PM_{2.5}$ exposure coefficients and variance around the estimates from the sample data and apply to the countries with unknown household air pollution to predict the $PM_{2.5}$ indoor concentration globally. This model provides estimates on $PM_{2.5}$ indoor concentration levels based on average 24-hour period.
The hierarchical model incorporating the following predictors for each country:

i. fuel types (biomass, charcoal, coal, gas, electricity);
ii. traditional/improved stove;
iii. urban/rural location;
iv. population weighted heating degree days;
v. population weighted ambient PM2.5

vi. GNI index;
vii. education index;
viii. season (winter/summer/whole year)

Annual population-weighted average $PM_{2.5}$ indoor concentration were estimated for 65 countries in five WHO regions (African Region, Eastern Mediterranean Region, Region of the Americas, South-East Asian Region, Western Pacific Region). We exclude the European Region due to high uncertainty in the estimated exposure values.

Attributable premature mortality due to $PM_{2.5}$ indoor concentration is estimated at national level (per 100,000 population) using the standard comparative risk assessment (CRA) approach. This involves calculation of population attributable fractions based on the estimated $PM_{2.5}$ indoor concentration for each country (and separately for urban and rural populations).[280,281] This exposure is then converted into an estimate of excess deaths using Global Burden of Disease functions. We use three following weighted averages to quantify the mortality rates for the number of attributable deaths per 100,000 individuals for solid fuels at national level: (i) Proportion of people using each fuel type (biomass, charcoal, coal, gas, electricity) in each country and for urban and rural settings.[273] (ii) Proportion of people using each stove type (traditional, improved) in each country for urban and rural settings. (iii) Proportion of people living in urban and rural setting in each country.

The household air pollution model includes ambient $PM_{2.5}$ exposure from GAINS as an input. The mortality estimates currently include some degree of overlap with estimates of mortality due to ambient air pollution, which is also the case for the WHO estimates.[282]

Data

- Ambient $PM_{2.5}$ concentrations for 2020 from IIASA[283]
- Fuel Type: IIASA GAINS model via IEA[284]
- Stove Type[285]
- Heating Degree Days for the year 2000 (1985–2015) provided by NASA[286]
- Education index and GNI index provided by United Nations Development Programme (UNDP). Year 2019
- WHO Global HAP Database.[272]
- WHO. Proportion of population with primary reliance on fuels and technologies for cooking, by fuel type (%)[287]
- Baseline mortality data: GBD national estimates for males and females[288]
- Exposure-response functions for attributable premature mortality: GBD2019 MR-BRTs, cause, and age specific, for six diseases[289]

The indicator provides useful information as to the variation of $PM_{2.5}$ exposure for a given fuel use and stove type and urban/rural locations as well as the related health impacts. The inclusion of ambient air pollution for urban and rural locations (obtained from IIASA GAINS modelled gridded data) and the heating degree days for the same urban and rural areas are two unique predictors used here for the first time in Bayesian $PM_{2.5}$ exposure models at global scale.

Caveats

Indoor air pollution is complex and impacted by a number of different factors including housing characteristics (e.g., ventilation rate, kitchen locations, window in kitchen, roofing materials) which

are not typically captured in all the monitored data. Updating the sample data with information on these and related factors should greatly improve the future predictions as to household air pollution. Another challenge concerns the measured/monitored household air pollution data (e.g., studies included in the WHO database). More specifically, the concerns are as follows: rather limited number of households monitored in each study; each study uses different monitoring technology to collect the data; and data collected from different measurement periods as well as different analytic methods used for data processing in each study. Nevertheless, using Bayesian predictive models developed in this study allows us to explore a wide range of $PM_{2.5}$ indoor concentration depending on fuel use, stove types, and for differences urban and rural locations of countries worldwide.

3.3: Food, agriculture, and health co-benefits

Indicator 3.3.1: emissions from agricultural production and consumption

Indicator authors

Dr Carole Dalin, Dr Harry Kennard

Methods

Since the 2023 update of this indicator, GHG emissions from agricultural production and consumption incorporates numerous fruits, vegetables, nuts, pulses and legumes and other crops. While these crops tend to have much lower GHG emission intensity than animal derived products, their inclusion provides a fuller picture of the agricultural commodities used in the global food system.

The methods by which the GHG emission estimates for food products is divided into two sections, one covering livestock and the second covering crops.

Livestock products

GHG emissions intensities for the year 2000 are calculated as follows in the following manner, as in Dalin et al.[290]

The included livestock species are listed below:
Table 87: Livestock species included in analysis

Ruminant	Non-Ruminant
Cattle, dairy<br>(FAO Item Code 960)	Chicken, broilers<br>(FAO Item Code 1053)
Cattle, non-dairy<br>(FAO Item Code 961)	Chicken, layers<br>(FAO Item Code 1052)
Buffaloes<br>(FAO Item Code 946)	Swine, market<br>(FAO Item Code 1049)
Goats<br>(FAO Item Code 1016)	Swine, breeding<br>(FAO Item Code 1079)
Sheep<br>(FAO Item Code 976)	

All livestock categories also include secondary products—such as cheese in the case of milk—where data were available. Cattle products comprise beef meat and milk and buffalo meat and milk. Sheep and goat products comprise meat and milk. Poultry products comprise meat and eggs of chickens, geese, ducks, and turkeys. Swine products include pork and secondary processed commodities, such as ham and bacon.

GHG emissions from enteric fermentation and manure management are obtained from Herrero et al.[291]

For manure left on pasture, rates from the GLOBIOM model were used[292] and a linear N_2O emission model applied.[293]

This information is presented in tonne carbon dioxide equivalent (CO_2e) per tropical livestock unit (tlu), which is converted to livestock head using the table below.[294]

Table 88: Conversion table to livestock head

	**Head per tlu**
Bovine (Buffalo, Cattle (dairy), Cattle(non-dairy))	1.43
Small Ruminants (Goats, Sheep)	10
Poultry (Chicken)	100
Swine	5

The emissions per head are divided into world regions (as in the GLOBIOM model) and, for ruminants, livestock system (combination of climates from arid to humid, and practices from rangeland to feedlots, c.f. Herrero et al. 2013).[291] To convert these emissions to country values, an average is made across the region-system pairs within each country, weighted by the number of animals.

To obtain the emissions from grazing, the synthetic fertilizer applied to grassland from Chang et al.[295] is used as input to the N_2O emission model.[293]Animal products' emissions also include emissions from feed crops proportionally to the feed ingredients consumed by animals—by species, region and systems— using feeding data from Herrero et al. 2013.[291] These emissions from feed crops (see crop section below) and grazed grasslands are then added to the direct livestock emissions (from enteric fermentation, manure management, and manure left on pasture) to provide overall emissions rates for each livestock species in the year 2000.

Finally, emissions intensity values for each livestock commodity (egg, meat, milk) and country are obtained by dividing CO_2e values by the output of milk/meat/egg per head from Herrero et al. 2013.[291]

Crop Products:

The emissions from fertiliser (synthetic and manure) application, rice cultivation, and cultivation from organic soils for 172 crops for the year 2000 are obtained from Carlson et al. 2017,[296] who use IPCC methodology and a N_2O emission model. Crop types corresponding to "fodder" and "fibre" types are then excluded for this report, leaving 147 crops which are directly consumed by humans.
Crops used for livestock feed are excluded from the "crops" emissions, as they are included in the intensity of livestock production; the FAO reports this in the following way: "Cereal crops harvested for hay or harvested green for food, feed or silage or used for grazing are therefore excluded".[294]

Production values 2001–2021

Since the emission intensity of production is not constant over time, its values by commodity (for both livestock and crop products) were scaled using the FAO values as an index. The FAO produces GHG emissions intensity values by animal commodity and broad crop category (distinguishing rice, which, unlike other crops, emits large amounts of methane) for the most countries. However, these values are volatile at the country level, so regional values were used here. The annual percentage change from the year 2000's value was applied to the intensities derived from Herrero et al.,[291] Chang et al.,[295] and Carlson et al.,[296] outlined above (methodology from Dalin et al.)[290]Any missing values in scaling factor were assumed to be 1 (constant emission intensity). Any intensity values missing for a given country were given the regional average for that year and commodity, although practically this had little impact, because missing values only corresponded to countries which had very low or no production of the commodity in question.

Consumption emissions

The GHG emissions associated with agricultural commodity consumption uses FAO production and trade data to estimate the total GHG emissions footprint associated with each of the commodities considered in a given country. This method is used by Dalin et al. 2017[297] for tracing water consumption in global food networks but is adapted here to calculated GHG intensity. The basic equation the indicator follows is:

Consumption = production + imports - exports

FAO production and trade data are used in the following manner. For a given commodity the national production values in tonnes are converted into CO_2e values using the GHG emissions intensity values supplied by indicator 3.3.1 GHG production estimates (via Carlson et al. 2017)[296] associated with producing that tonnage of the commodity. Next, secondary commodities are converted in primary equivalent values by multiplying the trade tonnage by the value derived from Dalin et al. 2017.[297] For example, the primary equivalences for wheat products are as shown in
Table 89:

Table 89: Primary equivalences for wheat products

Bran, wheat	1.01
Bread	0.88
Bulgur	1.05
Cereals, breakfast	1.18
Flour, wheat	1.01
Macaroni	1.01
Pastry	0.88
Wafers	0.88
Wheat	1.00

These values are then converted into GHG emissions equivalent, based on the GHG emissions intensity. For a given year, the trade balances are corrected to take into account that a given commodity may have been produced in one country, processed in another and finally imported into a third, using an algorithm first developed by Kastner et al 2011.[298]

Data

- National annual production of animal products items (tonnes) – FAOSTAT (2023 update)[294]
- National annual trade (country-country) of animal products items (tonnes) – FAOSTAT (2023 update) [294]
- Correspondence of items across item lists with different grouping – FAOSTAT[294]
- GHG production estimates including grassland and feed crop emissions (via Herrero et al. 2013 and Dalin et al. 2019)[290,291] Definitions: Animal types: bovine cattle (beef and buffalo), sheep and goat ruminants, pigs, poultry (chicken, ducks, geese and turkeys)
- National annual production of crops (tonnes) – FAOSTAT (2023 update) [294]
- National annual trade (country-country) of crop products (tonnes) – FAOSTAT (2023 update) [294]
- GHG emissions intensity of crop products for each country– provided by Carlson et al. (2017)[296]
- Dalin et al., 2017[297]
- Kastner et al., 2011 [298]

Caveats

In the context of this indicator, *consumption* refers to the net balance of food products entering a country within a given year, i.e., national production and *net* imports together, which could also be referred to as "national supply". Here net imports refers to imports minus exports. It does not refer to the total GHG emissions attributable to food consumed by individuals. Indeed, at present, this indicator only considers the emissions associated with food production described above and does not take into account emissions associated with food transport and processing, storage and decomposition.[299]

This indicator does not account for emissions associated with land conversion to agriculture (such as deforestation) but does consider emissions form cultivation of organic soils (such as peatland). For livestock, data on stock numbers has been extracted from FAO database, however, some data is missing for some years, most notably Somalia (missing data 2000–2011) for non-dairy cattle. Data on grazing emissions from small islands is also missing, and therefore imputed using regional average values as described above.

The emission factors differ from FAO numbers:

- For livestock, this is due to calculation of emissions of enteric fermentation, manure management and manure left on pasture at GLOBIOM region (n=29) and livestock system (n=8) level whereas the FAO use subcontinental (n=9) and climatic level (n=3).[294]
- For crops, this is due to the FAO assuming slightly higher synthetic N application, greater manure N inputs, and a linear emissions factor of 1%, in contrast to a mean of 0.77% used by the non-linear model of Carlson et al. (2017).[296]

Agricultural consumption emissions estimates are derived directly from FAO trade values (re-organised as producer-consumer trade only with the algorithm), as described above. Therefore, these values differ from the production estimates, which are based on extrapolating year 2000 figures. On average across all years, the estimate of total emissions due to consumption are 2.25% above production values, and do not differ by more than 10% in any given year. The sole exception to this is

the estimates of the differences between production and consumption by WHO region shown in the figure in the main text. For this figure the production values are derived directly from FAO values.

Additional analysis

A substantial amount of CO_2e is associated with food that it is not consumed, whether that be during the food production process, transportation loses, or being wasted at the plate. The volumes of food considered here include food that is wasted or lost in transport, but not the additional emissions associated with the decomposition of food waste. The IPCC estimates that between 8–10% of total anthropogenic GHG emissions are associated with food loss and waste.[300] However, wastage is not equally distributed by country, with one analysis finding that high income nations waste six times more than low-income ones (by weight).[246]

Indicator 3.3.2: diet and health co-benefits

Indicator authors

Prof Marco Springmann

Methods

Baseline consumption data

We estimated baseline food consumption by adopting estimates of food availability from the FAO's food balance sheets, and adjusting those for the amount of food wasted at the point of consumption.[301,302] We disaggregated this proxy for food consumption by age and sex by adopting the same age and sex-specific trends as observed in dietary surveys.[303]

An alternative would have been to rely on a set of consumption estimates that has been based on a variety of data sources, including dietary surveys, household budget and expenditure surveys, and food availability data.[304,305] However, neither the exact combination of these data sources, nor the estimation model used to derive the data have been made publicly available. For some individual countries, using dietary surveys would also have been an alternative. However, underreporting is a persistent problem in dietary survey,[306,307] and regional differences in survey methods would have meant that our results would not be comparable between countries. In contrast to dietary surveys, waste-adjusted food-availability estimates indicate levels of energy intake per region that reflect differences in the prevalence of overweight and obesity across regions.[308]

Food balance sheets report on the amount of food that is available for human consumption.[302] They reflect the quantities reaching the consumer, but do not include waste from both edible and inedible parts of the food commodity occurring in the household. As such, the amount of food actually consumed may be lower than the quantity shown in the food balance sheet depending on the degree of losses of edible food in the household, e.g., during storage, in preparation and cooking, as plate-waste, or quantities fed to domestic animals and pets, or thrown away.

We followed the waste-accounting methodology developed by the FAO to account for the amount of food wasted at the household level that was not accounted for in food availability estimates.[301] Table 90 provides and overview of the parameters used in the calculation.

For each commodity and region, we estimated food consumption by multiplying food availability data with conversion factors (*cf*) that represent the amount of edible food (e.g. after peeling) and with the percentage of food wasted during consumption (*1-wp(cns)*). For roots and tubers, fruits and vegetables, and fish and seafood, we also accounted for the differences in wastage between the proportion that is utilised fresh (pct_{frsh}) and the proportion that utilised in processed form (pct_{prcd}). The equation used for each food commodity and region was:

$$Consumption = Availability \cdot \frac{pct_{frsh}}{100} \cdot cf_{frsh} \cdot \left(1 - \frac{wp(cns_{frsh})}{100}\right)$$

$$+ Availability \cdot \frac{pct_{prcd}}{100} \cdot cf_{prcd} \cdot \left(1 - \frac{wp(cns_{prcd})}{100}\right)$$

Table 91. Percentage of food wasted during consumption (cns), and percentage of processed utilisation (pctprcd). The percentage of fresh utilisation is calculated as 1-pctprcd. Conversion factors to edible portions of foods are provided below the table.

Food group	Item	Region						
		Europe	USA, Canada, Oceania	Indus-trialized Asia	Sub-Saharan Africa	North Africa, West and Central Asia	South and Southeast Asia	Latin America
cereals	wp(cns)	25	27	20	1	12	3	10
roots and tuber	pct_{prcd}	73	73	15	50	19	10	80
	wp(cns)	17	30	10	2	6	3	4
	wp(cns_{prcd})	12	12	12	1	3	5	2
oilseeds and pulses	cns	4	4	4	1	2	1	2
fruits and vegetables	pct_{prcd}	60	60	4	1	50	5	50
	wp(cns)	19	28	15	5	12	7	10
	wp(cns_{prcd})	15	10	8	1	1	1	1
milk and dairy	wp(cns)	7	15	5	0.1	2	1	4
eggs	wp(cns)	8	15	5	1	12	2	4
meat	wp(cns)	11	11	8	2	8	4	6
fish and seafood	pct_{prcd}	*40% for low-income countries, and 96% for all others.*						
	wp(cns)	11	33	8	2	4	2	4
	wp(cns_{prcd})	10	10	7	1	2	1	2

Conversion factors: maize, millet, sorghum: 0.69; wheat, rye, other grains: 0.78; rice: 1; roots: 0.74 (0.9 for industrial processing); nuts and seeds: 0.79; oils: 1; vegetables: 0.8 (0.75 for industrial processing); fruits: 0.8 (0.75 for industrial processing); beef: 0.715; lamb: 0.71; pork: 0.68; poultry: 0.71; other meat: 0.7; milk and dairy: 1; fish and seafood: 0.5; other crops: 0.78

Comparative risk assessment

We estimated the mortality and disease burden attributable to dietary and weight-related risk factors by calculating population impact fractions (PIFs) which represent the proportions of disease cases that would be avoided when the risk exposure was changed from a baseline situation to a counterfactual situation. For calculating PIFs, we used the general formula[309–311]:

$$PIF = \frac{\int RR(x)P(x)dx - \int RR(x)P'(x)dx}{\int RR(x)P(x)dx}$$

where $RR(x)$ is the relative risk of disease for risk factor level x, $P(x)$ is the number of people in the population with risk factor level x in the baseline scenario, and $P'(x)$ is the number of people in the population with risk factor level x in the counterfactual scenario. We assumed that changes in relative risks follow a dose-response relationship,[310] and that PIFs combine multiplicatively, i.e. $PIF = 1 - \prod_i (1 - PIF_i)$ where the *i*'s denote independent risk factors.[310,312]

The number of avoided deaths due to the change in risk exposure of risk *i*, $\Delta deaths_i$, was calculated by multiplying the associated PIF by disease-specific death rates, *DR*, and by the number of people alive within a population, *P*:

$$\Delta deaths_i(r,s,a,d) = PIF_i(r,s,a,d) \cdot DR(r,s,a,d) \cdot P(r,s,a)$$

where PIFs are differentiated by region *r*, sex *s*, age group *a*, and disease/cause of death *d*; the death rates are differentiated by region, sex, age group, and disease; the population groups are differentiated by region, sex, and age group; and the change in the number of deaths is differentiated by region, sex, age group, and disease.
We used publicly available data sources to parameterize the comparative risk analysis. Mortality and population data were adopted from the Global Burden of Disease project.[313] Baseline data on the weight distribution in each country were adopted from a pooled analysis of population-based measurements undertaken by the NCD Risk Factor Collaboration.[308]

The relative risk estimates that relate the risk factors to the disease endpoints were adopted from meta-analyses of prospective cohort studies for dietary and weight-related risks.[314–320] In line with the meta-analyses, we included non-linear dose-response relationships for fruits, vegetables, and nuts and seeds, and assumed linear dose-response relationships for the remaining risk factors. As our analysis was primarily focused on mortality from chronic diseases, we focused on adults aged 20 year or older, and we adjusted the relative-risk estimates for attenuation with age based on a pooled analysis of cohort studies focussed on metabolic risk factors,[321] in line with other assessments.[311,322]

Table 92 provides an overview of the relative-risk parameters used. For the counterfactual scenario, we defined minimal risk exposure levels (TMRELs) as follows: 300 g/d for fruits, 500 g/d for vegetables, 100 g/d for legumes, 20 g/d for nuts and seeds, 125 g/d for whole grains, 0 g/d for red meat, 0 g/d for processed meat, and no underweight, overweight, or obesity. The TMRELs are in line with those defined by the Nutrition and Chronic Diseases Expert Group (NutriCoDE),[322] with the exception that we used a higher value for vegetables, and we used zero as minimal risk exposure for red meat, in each case based on a more comprehensive meta-analysis.[316,317]

The selection of risk-disease associations used in the health analysis was supported by available criteria used to judge the certainty of evidence, such as the Bradford-Hill criteria used by the Nutrition and Chronic Diseases Expert Group (NutriCoDE),[322] the World-Cancer-Research-Fund criteria used by the Global Burden of Disease project,[323] as well as NutriGrade (Table 93).[324] The certainty of evidence supporting the associations of dietary risks and disease outcomes as used here were graded as moderate or high with NutriGrade,[317–319] and/or assessed as probable or convincing by the Nutrition and Chronic Diseases Expert Group,[322] and by the World Cancer Research.[325] The certainty of

evidence grading in each case relates to the general relationship between a risk factor and a health outcome, and not to a specific relative-risk value.

Table 94: Relative risk parameters (mean and low and high values of 95% confidence intervals) for dietary risks and weight-related risks.

Food group	Endpoint	Unit	RR mean	RR low	RR high	Reference
Processed meat	CHD	50 g/d	1.27	1.09	1.49	Bechthold et al (2019)
	Stroke	50 g/d	1.17	1.02	1.34	Bechthold et al (2019)
	Colorectal cancer	50 g/d	1.17	1.10	1.23	Schwingshackl et al (2018)
	Type 2 diabetes	50 g/d	1.37	1.22	1.55	Schwingshackl et al (2017)
Red meat	CHD	100 g/d	1.15	1.08	1.23	Bechthold et al (2019)
	Stroke	100 g/d	1.12	1.06	1.17	Bechthold et al (2019)
	Colorectal cancer	100 g/d	1.12	1.06	1.19	Schwingshackl et al (2018)
	Type 2 diabetes	100 g/d	1.17	1.08	1.26	Schwingshackl et al (2017)
Fruits	CHD	100 g/d	0.95	0.92	0.99	Aune et al (2017)
	Stroke	100 g/d	0.77	0.70	0.84	Aune et al (2017)
	Cancer	100 g/d	0.94	0.91	0.97	Aune et al (2017)
Vegetables	CHD	100 g/d	0.84	0.80	0.88	Aune et al (2017)
	Cancer	100 g/d	0.93	0.91	0.95	Aune et al (2017)
Legumes	CHD	57 g/d	0.86	0.78	0.94	Afshin et al (2014)
Nuts	CHD	28 g/d	0.71	0.63	0.80	Aune et al (2016)
Whole grains	CHD	30 g/d	0.87	0.85	0.90	Aune et al (2016b)
	Cancer	30 g/d	0.95	0.93	0.97	Aune et al (2016b)
	Type 2 diabetes	30 g/d	0.65	0.61	0.70	Aune et al (2016b)
Underweight	CHD	15<BMI<18.5	1.17	1.09	1.24	Global BMI Collab (2016)
	Stroke	15<BMI<18.5	1.37	1.23	1.53	Global BMI Collab (2016)
	Cancer	15<BMI<18.5	1.10	1.05	1.16	Global BMI Collab (2016)
	Respiratory disease	15<BMI<18.5	2.73	2.31	3.23	Global BMI Collab (2016)
Overweight	CHD	25<BMI<30	1.34	1.32	1.35	Global BMI Collab (2016)
	Stroke	25<BMI<30	1.11	1.09	1.14	Global BMI Collab (2016)
	Cancer	25<BMI<30	1.10	1.09	1.12	Global BMI Collab (2016)
	Respiratory disease	25<BMI<30	0.90	0.87	0.94	Global BMI Collab (2016)
	Type 2 diabetes	25<BMI<30	1.88	1.56	2.11	Prosp Studies Collab (2009)
Obesity (grade 1)	CHD	30<BMI<35	2.02	1.91	2.13	Global BMI Collab (2016)
	Stroke	30<BMI<35	1.46	1.39	1.54	Global BMI Collab (2016)
	Cancer	30<BMI<35	1.31	1.28	1.34	Global BMI Collab (2016)
	Respiratory disease	30<BMI<35	1.16	1.08	1.24	Global BMI Collab (2016)
	Type 2 diabetes	30<BMI<35	3.53	2.43	4.45	Prosp Studies Collab (2009)
Obesity (grade 2)	CHD	30<BMI<35	2.81	2.63	3.01	Global BMI Collab (2016)
	Stroke	30<BMI<35	2.11	1.93	2.30	Global BMI Collab (2016)
	Cancer	30<BMI<35	1.57	1.50	1.63	Global BMI Collab (2016)
	Respiratory disease	30<BMI<35	1.79	1.60	1.99	Global BMI Collab (2016)
	Type 2 diabetes	30<BMI<35	6.64	3.80	9.39	Prosp Studies Collab (2009)
Obesity (grade 3)	CHD	30<BMI<35	3.81	3.47	4.17	Global BMI Collab (2016)
	Stroke	30<BMI<35	2.33	2.05	2.65	Global BMI Collab (2016)
	Cancer	30<BMI<35	1.96	1.83	2.09	Global BMI Collab (2016)
	Respiratory disease	30<BMI<35	2.85	2.43	3.34	Global BMI Collab (2016)
	Type 2 diabetes	30<BMI<35	12.49	5.92	19.82	Prosp Studies Collab (2009)

We did not include all available risk-disease associations that were graded as having a moderate certainty of evidence and showed statistically significant results in the meta-analyses that included

NutriGrade assessments.[317–319] That was because for some associations, such as for milk and fish, more detailed meta-analyses (with more sensitivity analyses) were available that indicated potential confounding with other major dietary risks or health status at baseline.[326–328] Such sensitivity analyses were not presented in the meta-analyses that included NutriGrade assessments, but they are important for health assessments that evaluate changes in multiple risk factors.

Table 95: Overview of existing ratings on the certainty of evidence for a statistically significant association between a risk factor and a disease endpoint. The ratings include those of the Nutrition and Chronic Diseases Expert Group (NutriCoDE),[322] the World Cancer Research Fund,[325] and NutriGrade.[317–319] The ratings relate to the risk-disease associations in general, and not to the specific relative-risk factor used for those associations in this analysis.

Food group	Endpoint	Association	Certainty of evidence
Fruits	CHD	reduction	NutriCoDE: probable or convincing;<br>NutriGrade: moderate quality of meta-evidence
	Stroke	reduction	NutriCoDE: probable or convincing<br>NutriGrade: moderate quality of meta-evidence
	Cancer	reduction	WCRF: strong evidence (probable) for some cancers<br>NutriGrade: moderate quality of meta-evidence for colorectal cancer
Vegetables	CHD	reduction	NutriCoDE: probable or convincing<br>NutriGrade: moderate quality of meta-evidence
	Cancer	reduction	WCRF: strong evidence (probable) for non-starchy vegetables and some cancers<br>NutriGrade: moderate quality of meta-evidence for colorectal cancer
Legumes	CHD	reduction	NutriCoDE: probable or convincing<br>NutriGrade: moderate quality of meta-evidence
Nuts and seeds	CHD	reduction	NutriCoDE: probable or convincing<br>NutriGrade: moderate quality of meta-evidence
Whole grains	CHD	reduction	NutriCoDE: probable or convincing<br>NutriGrade: moderate quality of meta-evidence
	Cancer	reduction	WCRF: strong evidence (probable) for colorectal cancer<br>NutriGrade: moderate quality of meta-evidence for colorectal cancer
	Type-2 diabetes	reduction	NutriCoDE: probable or convincing<br>NutriGrade: high quality of meta-evidence
Red meat	CHD	increase	NutriGrade: moderate quality of meta-evidence
	Stroke	increase	NutriGrade: moderate quality of meta-evidence
	Cancer	increase	WCRF: strong evidence (probable) for colorectal cancer<br>NutriGrade: moderate quality of meta-evidence for colorectal cancer
	Type-2 diabetes	increase	NutriCoDE: probable or convincing<br>NutriGrade: high quality of meta-evidence
Processed meat	CHD	increase	NutriCoDE: probable or convincing<br>NutriGrade: moderate quality of meta-evidence
	Stroke	increase	NutriGrade: moderate quality of meta-evidence
	Cancer	increase	WCRF: strong evidence (convincing) for colorectal cancer<br>NutriGrade: moderate quality of meta-evidence for colorectal cancer
	Type-2 diabetes	increase	NutriGrade: high quality of meta-evidence

NutriCoDE: Nutrition and Chronic Diseases Expert Group
NutriGrade: Grading of Recommendations Assessment, Dvelopment, and Evaluation (GRADE) tailored to nutrition research
WCRF: World Cancer Research Fund

Weight-related risks are connected to imbalanced energy intake. To highlight this connection, we attributed the weight-related disease burden to consuming too much or too little of specific foods. For that purpose, we first compared the current energy intake by food group in each country to a dietary pattern that minimises both diet and weight-related risks, and then attributed the proportion of energy

intake of under and over-consumed foods to the proportion of deaths attributable to underweight on the one hand and to overweight and obesity on the other. The minimal-risk patterns were based on recommendations for optimal energy intake given the sex, age, and height structure of each country,[308,329] the TMREL values used in the dietary risk assessment,[314,315,317,322,330–332] and food-based recommendations for healthy and sustainable diets for the remaining food groups.[333] We implemented the recommendations as minimum and maximum values, which preserved a country's intake if it was within recommendations (96).

Table 97: Food-based recommendations used to construct minimal risk dietary patterns. The recommendations include minimal risk exposure levels for dietary risks (upper rows) and food-based recommendations for a healthy and sustainable diets (lower rows).

Food group	Recommended intake		Source
	Min	Max	
Fruits	300	>300	Aune et al (2017)
Vegetables	500	>500	Aune et al (2017)
Legumes	100	>100	Micha et al (2017), Afshin et al (2017)
Nuts and seeds	20	>20	Micha et al (2017), Aune et al (2016a)
Whole grains	125	225	Micha et al (2017), Aune et al (2016b)
Red meat	0	0	GBD 2019 (2020), Bechthold et al (2017)
Processed meat	0	0	GBD 2019 (2020), Bechthold et al (2017)
Oils	40	80	Willett et al (2019)
Sugar	0	31	Willett et al (2019)
Roots	0	100	Willett et al (2019)
Milk	0	250	Willett et al (2019)
Eggs	0	13	Willett et al (2019)
Poultry	0	29	Willett et al (2019)
Fish	0	28	Willett et al (2019)

For the different diet scenarios, we calculated uncertainty intervals associated with changes in mortality based on standard methods of error propagation and the confidence intervals of the relative risk parameters. For the error propagation, we approximated the error distribution of the relative risks by a normal distribution and used that side of deviations from the mean which was largest. This method leads to conservative and potentially larger uncertainty intervals as probabilistic methods, such as Monte Carlo sampling, but it has significant computational advantages, and is justified for the magnitude of errors dealt with here (<50%) (see e.g. IPCC Uncertainty Guidelines).

Data

Table 98 provides an overview of the data sources used for this indicator.

Table 99 Overview of data sources

Type	Coverage	Source
Exposure data:		
Food consumption data	Country-level	Food availability data adjusted for food waste at the household level and for age and sex-specific trends.[301,303,334] Estimates of energy intake were in line with trends in body weight across countries.[308]
Weight estimates	Country-level	Baseline data from pooled analysis of measurement studies differentiated by sex and age with global coverage.[308]
Health analysis:		
Relative risk estimates	General	Adopted from meta-analysis of prospective cohort studies.[314–320] The certainty of evidence for the risk-disease associations were rated as moderate to high by NutriGrade.[317–319]
Mortality and population data	Country-level	Adopted from the Global Burden of Disease project by country, sex, and age group.[313]

Caveats

In the comparative risk assessment, we used relative risk factors that are subject to the caveats common in nutritional epidemiology, including small effect sizes and potential measurement error of dietary exposure, such as over and underreporting and infrequent assessment.[335] For our calculations, we assumed that the risk-disease relationships describe causal associations, an assumption supported by the existence of statistically significant dose-response relationships in meta-analyses, the existence of plausible biological pathways, and supporting evidence from experiments, e.g. on intermediate risk factors.[317–320,322,330,331] However, residual confounding with unaccounted risk factors cannot be ruled out in epidemiological studies. Additional aspects rarely considered in meta-analyses are the importance of substitution between food groups that are associated with risks, and the time lag between dietary exposure and disease.

To address potential confounding, we omitted risk-disease associations that became non-significant in fully adjusted models, in particular those related milk intake,[326,327] and to fish intake.[328] The quality of evidence in meta-analyses that covered the same risk-disease associations as used here was graded with NutriGrade as moderate or high for all risk-disease pairs included in the analysis (SI Table 3).[317–319] In addition, the Nutrition and Chronic Diseases Expert Group and the World Cancer Research Fund graded the evidence for a causal association of ten of the 12 risk-disease associations included in the analysis as probable or convincing,[322,325] The relative health ranking of leading risk factors found

in our analysis was similar to existing rankings that relied on different relative-risk parameters and exposure data.[323]

As exposure data, we used a proxy of food consumption that was derived from estimates of food availability that were adjusted for the amount of food wasted at the point of consumption.[301,302] An alternative would have been to rely on a set of consumption estimates that has been based on a variety of data sources, including dietary surveys, household budget and expenditure surveys, and food availability data.[304,305] However, neither the exact combination of these data sources, nor the estimation model used to derive the data have been made publicly available. For some individual countries, using dietary surveys would also have been an alternative. However, underreporting is a persistent problem in dietary survey,[306,307] and regional differences in survey methods would have meant that our results would not be comparable between countries. In contrast to dietary surveys, waste-adjusted food-availability estimates indicate levels of energy intake per region that reflect differences in the prevalence of overweight and obesity across regions.[308]

Indicator 3.4: tree cover loss

Indicator authors

Prof. David Rojas-Rueda

Methods

The data focused on all vegetation five meters or taller and areas with at least 30% tree cover density.[336,337] Tree cover loss was defined as the disturbance of a stand or the complete removal of the tree cover canopy at the pixel scale of the satellite image. This loss can occur due to human activities, such as forestry practices like timber harvesting and deforestation, as well as natural causes like disease or storm damage.

The data area totaled 128.8Mkm2 and included all global land except Antarctica and several Arctic islands. This approach used satellite imagery analysis from 2001 to 2022 at a resolution of 30x30 meters to measure the global loss of tree cover.[336,337] The dataset used in this study included Landsat 7 Enhanced Thematic Mapper Plus (ETM+) scenes of the growing season. The dataset was pre-processed using automated Landsat pre-processing steps, which included image resampling, conversion of raw digital values (DN) to top-of-atmosphere (TOA) reflectance, cloud/shadow/water screening and quality assessment (QA), and image normalization. The training data for percent tree cover and forest loss were used to relate to the time-series metrics using a decision tree. Decision trees are hierarchical classifiers that predict class membership by recursively partitioning a data set into more homogeneous or less than 3 varying subsets, referred to as nodes. For the tree cover and change products, a bagged decision tree methodology was employed. Forest loss was disaggregated to annual time scales using a set of heuristics derived from the maximum annual decline in percent tree cover and the maximum annual decline in minimum growing season Normalized Vegetation Difference Index (NDVI). Trends in annual forest loss were derived using an ordinary least squares slope of the regression of y=annual loss versus x=year.

To identify the various types of tree cover loss, such as deforestation, forestry, wildfire, urbanisation, and shifting agriculture, a decision tree model (recursive partitioning model) was used.[337] The model used true/false conditions of data values to predict the most likely driver of tree cover loss for each

grid cell. To estimate the proportion of tree cover loss drivers, a sample-based approach and stratified random sampling were used, and confidence intervals were calculated. The model was able to differentiate between permanent conversion (deforestation) and temporary loss due to forestry or wildfire. The overall accuracy of the model was 89%, with individual class accuracies ranging from 55% (urbanization) to 94% (deforestation). The five drivers are defined as follows:

- Commodity-driven deforestation: Large-scale deforestation linked primarily to commercial agricultural expansion.
- Shifting agriculture: Temporary loss or permanent deforestation due to small- and medium-scale agriculture.
- Forestry: Temporary loss from plantation and natural forest harvesting, with some deforestation of primary forests.
- Wildfire: Temporary loss, does not include fire clearing for agriculture.
- Urbanization: Deforestation for expansion of urban centers.

The commodity-driven deforestation and urbanisation categories represent permanent deforestation, while tree cover affected by the other categories often regrows. The data set does not indicate the stability or condition of land cover after the tree cover loss occurs or distinguish between natural and anthropogenic wildfires.

Data

- Tree cover loss: Hansen/UMD/Google/USGS/NASA[338]
- Administrative boundaries: Global Administrative Areas database (GADM), version 3.6.
- Tree Cover Loss by Driver: The Sustainability Consortium, World Resources Institute, and University of Maryland.

Caveats

Exclusion of Certain Disturbances: The model does not include disturbances such as insect outbreaks, wind and ice storms, flooding, or rivers changing course. These disturbances were found to be highly localized and temporally restricted, affecting only 1% of all model validation sample cells.

Misclassification Issues: There was low model accuracy for the commodity-driven deforestation class in Africa, with much of this deforestation misclassified as shifting agriculture. In northern forests, especially in Russia, distinguishing between drivers was challenging in areas where wildfires spread through previously logged areas or where logging occurred after a fire event.

Lack of Detailed Differentiation: The study did not map changes in forest conditions over time in landscapes dominated by shifting agriculture, nor did it differentiate primary from secondary forest clearing within this land-use class. Differentiating key drivers like row crops from pasturelands in South America or tree plantations from disturbed natural forests in Southeast Asia could enhance the analysis.

Indicator 3.5: healthcare sector emissions and harms

Indicator authors

Dr Matthew Eckelman, Dr Jodi D. Sherman

Methods

This indicator is in the form of healthcare-associated GHG emissions per capita per year, including direct emissions from healthcare facilities as well as emissions from the consumption of goods and services supplied by other sectors. Results are calculated by assigning aggregate national health expenditures from WHO to final demand for 'Health and Social Work' sectors in the EE-MRIO model. Environmental satellite accounts including GHG emissions accompany each EE-MRIO model. Consumption-based GHG emissions are then calculated using the standard Leontief inverse technique.[339]

Modeling for years 2019–2022 of the *Lancet* Countdown report utilized the WIOD MRIO model; however, the most recent emissions satellite accounts for this model date to 2016 for carbon dioxide emissions or to 2013 when considering other air pollutant emissions. Starting in 2023 and continuing this year, the most recent version (v3.8.2) of the EXIOBASE MRIO model was used, which includes macroeconomic tables across multiple years, including the current model year 2021.

EXIOBASE uses euros as the currency unit. Per capital WHO expenditure data in 2021 US dollars were converted to 2021 using the average exchange rate for that year (1 USD = 0.8455 EUR) as expressed natively in the WHO health accounts for that year. Because the expenditure and model years were the same, no deflation adjustments were necessary.

EXIOBASE v3.8.3 satellite accounts include both uncharacterized emissions (physical quantities) and characterized emissions (impacts). Here, the characterized emissions intensities were used for GHG emissions (in CO_2-equivalents) per million 2021 EUR for estimation of health sector carbon footprints. For estimation of public health damages, separate EXIOBASE factors for disability-adjusted life-years (DALYs) per million 2021 EUR were used for emissions of $PM_{2.5}$ and ozone precursors.

Data

- Environmentally extended multi-region input-output tables: EXIOBASE v3.8.3 model for year 2021.
- Per capita health expenditure data is from the World Health Organization's Global Health Expenditure Database; the latest reporting year is 2021.[340]
- UN Sustainable Development Goal Indicator 3.8.1 *Coverage of Essential Health Services* from the World Health Organization's Global Health Observatory; the latest reporting year is 2021.[341]

Caveats

As only total health expenditure data are available from WHO, all expenditures are assigned to Final Demand, with no separation for investment.

MRIO models are built from aggregated top-down statistical data. Results do not reflect individual health care systems' power purchase agreements for renewable energy or any offsetting activities. Results do not include direct emissions of waste anaesthetic gases from clinical operations nor emissions from metered dose inhalers, as these are not currently reported consistently in national emissions inventories.

Section 4: Economics and Finance

Section Lead: Prof Michael Grubb
Research Fellow: Dr Daniel Scamman

4.1: The economic impact of climate change and its mitigation

Indicator 4.1.1: economic losses due to weather-related extreme events

Indicator authors

Dr Daniel Scamman

Methods

The Swiss Re Institute provided the data for this indicator.[342] The Swiss Re Institute sigma catastrophe database is an international commercial database recording both natural and man-made disasters from 1970 and has over 12,000 entries.

The term 'natural catastrophe' refers to an event caused by natural forces. Such an event generally results in a large number of individual losses involving many insurance policies. The scale of the losses resulting from a catastrophe depends not only on the severity of the natural forces concerned, but also on man-made factors, such as building design or the efficiency of disaster control in the afflicted region.

Natural catastrophes are categorised as shown in Table 100.

Table 100: Categorisation of natural catastrophes in the data provided by the Swiss Re Institute.

Category	Peril Group	Peril
	Earthquake	Earthquake
		Tsunami
		Volcano eruption
	Weather-related	Storm
		Flood
		Hail
		Cold, frost
		Drought, bush fires, heat waves
		Other natural catastrophes

For this indicator, only data for 'weather-related' events is presented.

Total (insured and uninsured) economic losses reported by Swiss Re are all the financial losses directly attributable to a major event, i.e., damage to buildings, infrastructure, vehicles etc. This also includes losses due to business interruption as a direct consequence of the property damage. Insured losses are gross of any reinsurance, be it provided by commercial or government schemes. Total loss figures do not include indirect financial losses – i.e., loss of earnings by suppliers due to disabled businesses, estimated shortfalls in GDP and non-economic losses, such as loss of reputation or impaired quality of life. Insured losses refer to all insured losses except liability. To calculate uninsured losses, insured losses are subtracted from total losses.

Data are collected from a variety of sources, both internal and external. These include professional insured claims aggregators as well as insurance associations. Among the sources are also official government data, when available. Economic loss data can be estimated on the basis of Swiss Re proprietary catastrophe risk models. Also, if insured loss data are available, economic loss data are estimated on the basis of the local insurance penetration and other event-specific information (such as damages to public infrastructure, number of buildings damaged or destroyed etc.).

Minimum thresholds apply for inclusion in the database. At least one of the following must apply, for events recorded in 2023 (with economic values changing each year following changes to US CPI):

- **Insured losses (claims):** $26.0 million (maritime disasters), $51.9 million (aviation), $64.5 million (other)
- **Economic losses**: $129 million
- **Casualties**: Dead or missing: 20; Injured: 50; Homeless: 2000

Loss values are presented in US$, or if initially expressed in local currency, converted to US$ using year-end exchange rates.

Country data are summed into the four HDI classifications (Very High, High, Medium, Low). Data is also presented according to WHO regions and LC groupings. Further information on the methodology of the sigma explorer database can be found here: https://www.sigma-explorer.com/documentation/Methodology_sigma-explorer.com.pdf. Total insured and uninsured losses are then divided by total GDP for each year and HDI group. Nominal GDP values are taken from the IMF's World Economic Outlook (October 2023 Edition) and inflated to $2023 using data from IMF's International Finance Statistics. All values reported for this indicator are in $2023.

Data

- Swiss Re Institute sigma catastrophe database[342]
- IMF World Economic Outlook (October 2023)[343]
- IMF International Finance Statistics (2024)[344]

Caveats

Only events with measurable economic losses above the threshold levels are included. Each natural catastrophe event recorded is assigned a direct economic loss, and where applicable, an insured loss. Where available, data is taken from official institutions, but where not, estimates are calculated. The process for estimation depends on what data is available. For example, if loss estimates from insurance market data is available, this data may be combined with data on insurance penetration and other event-specific information to estimate total economic losses. If only low-quality information is available, such as a description of the number of homes damaged or destroyed, assumptions on value and costs are made. Some data (including both losses and GDP values) may be revised compared to previous reports, due to updated information or detailed measurement approaches.

Additional analysis

Charts showing losses as a fraction of GDP are shown below grouped according to HDI band, WHO region and LC group (Figure 149, Figure 150, and Figure 151). The underlying data for these charts is available in the online data explorer.

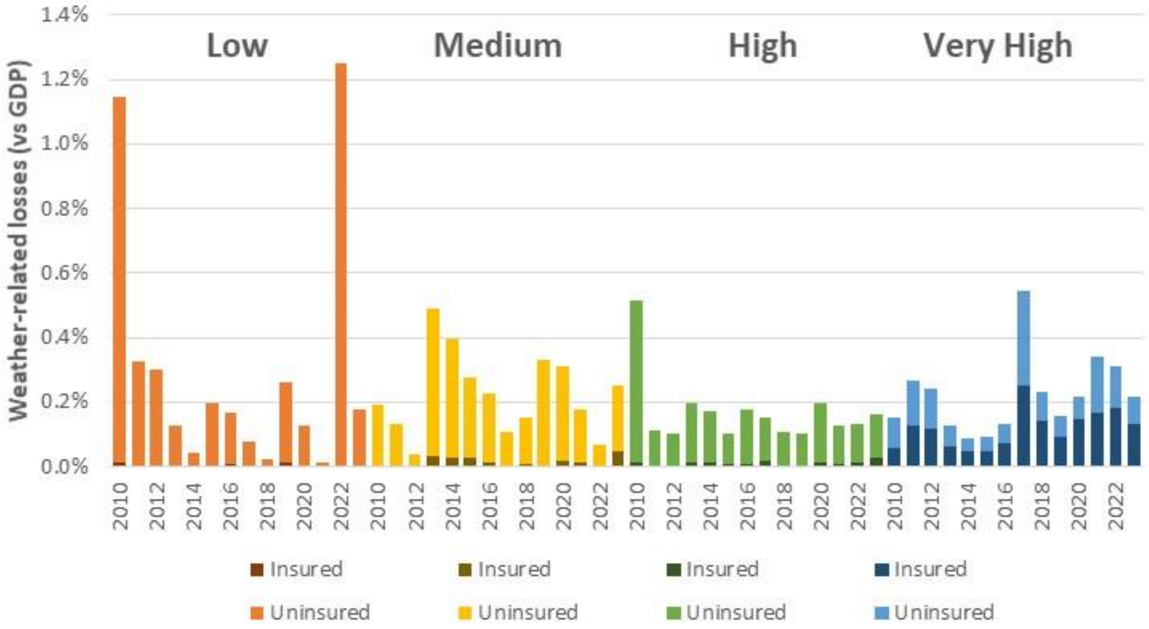

Figure 149: Insured and uninsured losses from weather-related extreme events vs GDP 2010–2023, by HDI band.

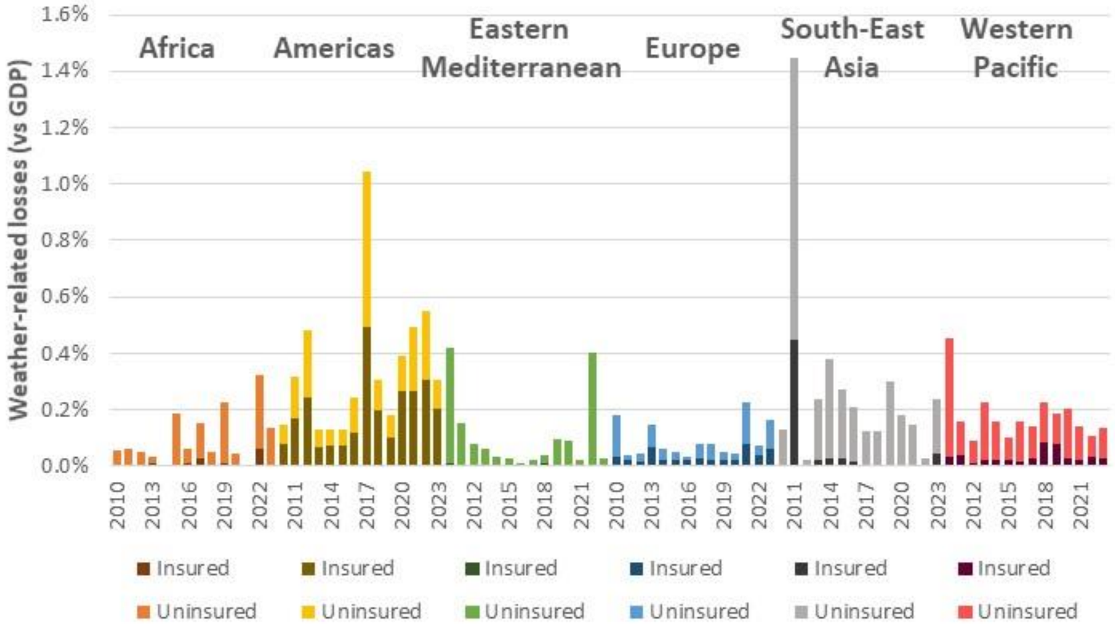

Figure 150: Insured and uninsured losses from weather-related extreme events vs GDP 2010–2023, by WHO region.

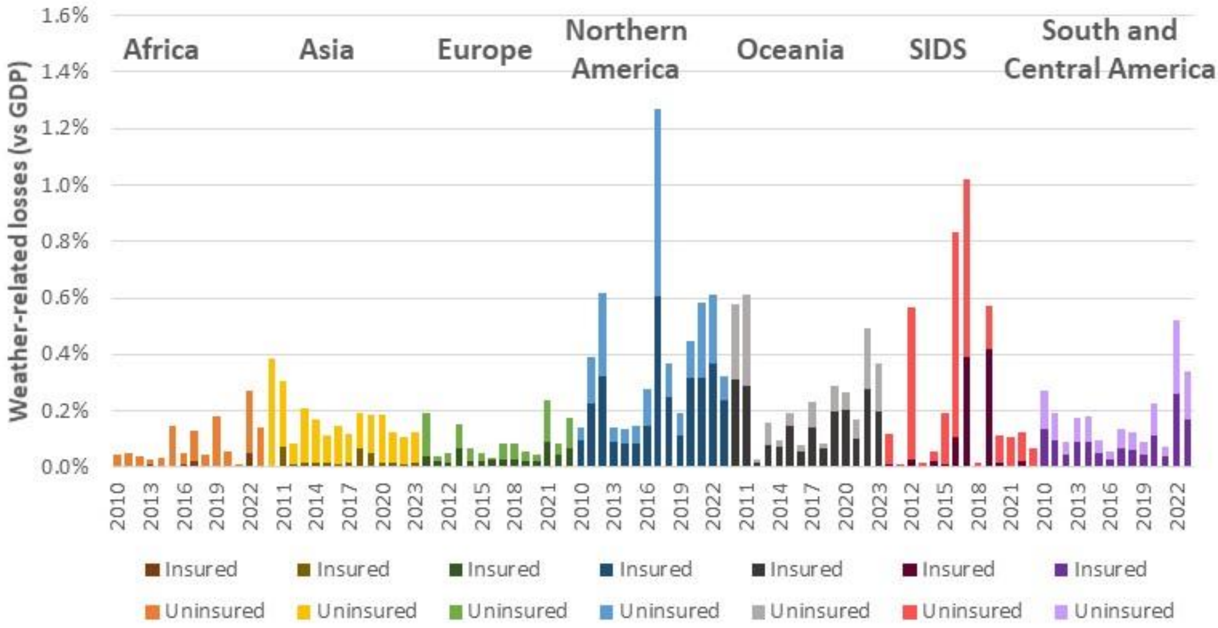

Figure 151: Insured and uninsured losses from weather-related extreme events vs GDP 2010–2023, by LC group.

Indicator 4.1.2: costs of heat-related mortality

Indicator authors

Prof Wenjia Cai, Dr Shihui Zhang

Methods

This indicator used the value of statistical life-year (VSLY) to monetise the years of life lost (YLL) caused by heat-related mortality (data for which is provided by indicator 1.1.5). Unlike using the value of a statistical life (VSL) to monetise mortality, the usage of VSLY can reflect age structure differences of heat-related mortalities across countries. VSLY measures how people value the discounted years of remaining life.[345] VSL can be interpreted as the discounted sum of VSLY of each year remaining in life, therefore, mathematically, the VSLY can be derived from the VSL and how many remaining years people are expected to live at certain age (Eq.2). As for the change of VSLY to age, some studies assumed that VSLY is constant across age span, while others assumed that VSLY will increase before mid-age and then decrease till death, which is an Inverted-U shape.[346] 169 countries spanning six World Health Organization (WHO) regions were included in the estimation. Population and GDP per capita are taken from the World Bank and OECD statistics.[347,348] The life table used to derive remaining years of life, was taken from WHO.[349]

The same ratio between VSLY and GDP-per-capita is assumed for each country for years 2000–2023, and data from OECD countries was used as the basis to derive the ratio on account of data availability and method consistency across reports in different years. The assumption is shown in Eq. (1), where *Y*

denotes the gross domestic product (GDP) per capita, *i* denotes the country in WHO regions, *t* denotes time.

$$\frac{VSLY_{it}}{Y_{it}} = \frac{VSLY_{OECD}}{Y_{OECD}} \qquad (1)$$

The relationship between VSL and VSLY can be obtained by years of remaining life at death (L) and discount rate (r), as shown in Eq.(2). The average VSL for OECD countries (VSL_{OECD}) was estimated at US$ 3.83 million ($2015) in 2015, and the average GDP per capita for OECD countries was $40,494 ($2015) in 2015. Equation 2 indicates that the total discounted value of each year's life value (VSLY) is equal to the value in the whole life span (VSL). Here it is assumed the VSLY remains constant for each remaining life year because only mortality of people aged over 65 is considered, where the fluctuations of VSLYs are very small even under the Inverted-U assumption.[346] The discount rate used here is 3%.

$$VSLY_{it} = \frac{VSL_{it} \cdot r}{1-(1+r)^{-L}} \qquad (2)$$

In order to calculate the monetised value of years of life loss (YLL) relative to per-capita GDP (*R*), Eq.(3) was applied, where YLL is multiplied by the fixed VSLY-to-GDP per capita-ratio produced by Eq.(1).

$$R_{it} = \frac{VSLY_{it} * YLL_{it}}{Y_{it}} = \frac{VSLY_{OECD}}{Y_{OECD}} * YLL_{it} \qquad (3)$$

In order to calculate monetised value of years of life loss as a proportion of GDP (V), Eq.(4) was applied, where YLL as a proportion of total population (P) is multiplied by the fixed VSLY-to-GDP per capita-ratio in OECD countries.

$$V_{it} = \frac{VSLY_{it} * YLL_{it}}{GNI_{it}} = \frac{VSLY_{it} * YLL_{it}}{Y_{it} * P_{it}} = \frac{VSLY_{OECD}}{Y_{OECD}} * \frac{YLL_{it}}{P_{it}} \qquad (4)$$

Country-level results are aggregated according both to WHO regions and HDI level. Considering data availability, some countries in WHO regions are not included: Cabo Verde, Sao Tome and Principe, Saint Vincent and the Grenadines, US Virgin Islands, Samoa, Eritrea, Andorra, Antigua and Barbuda, Bahrain, Barbados, Cook Islands, Dominica, Grenada, Kiribati, Maldives, Malta, Marshall Islands, Micronesia, Monaco, Montenegro, Nauru, Niue, Palau, Saint Kitts and Nevis, Saint Lucia, San Marino, Seychelles, Singapore, South Sudan, Tonga, Tuvalu. The population of these countries accounts for 0.3% of total population in WHO regions. In order to quantify the monetised value of economic costs, we also used the world average GDP per capita (2023 USD) to multiply the equivalent of GDP per capita. The world average GDP per capita in 2023 USD were derived from GDP per capita in 2015 USD and inflation rate.[350]

Data

- Heat-related mortality data is provided by Indicator 1.1.5 in section 1.

- Population in each country are taken from World Bank.[351]
- GDP per capita in OECD members are taken from OECD statistics.[348]
- VSL in OECD are taken from OECD report on Mortality Risk Valuation in Environment, Health
- and Transport Policies.[352]
- Years of remaining life are obtained from WHO.[349]
- World average GDP per capita in 2015 USD are taken from World Bank.[347]
- Inflation rate each year are taken from IMF.[350]

Caveats

This indicator could be improved through two perspectives. First, the inequality embedded within the economic costs of heat-related mortalities across different social groups are ignored in this indicator due to lack of data. In future, with heat-related mortality data with more detailed social groups aggregations, this indicator might explore further inequalities. Second, this indicator only considered the direct costs from mortalities of older populations, ignoring the potential costs that might be derived from it.

Future form of the indicator

In the future we will consider estimating the direct and indirect costs from heat-related mortalities of older persons.

Additional Analysis

The charts below show the indicator results, according to HDI, WHO and LC groupings (Figure 152, Figure 153, and Figure 154).

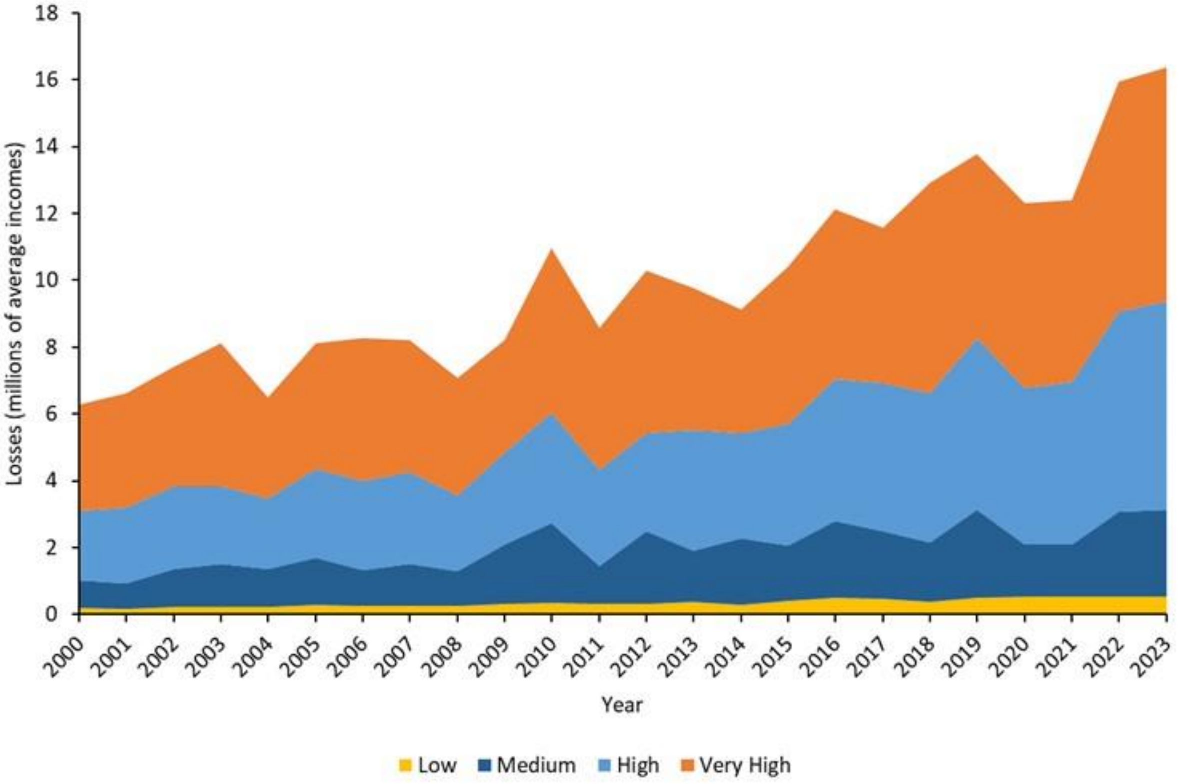

Figure 152: Monetised value of heat-related mortality losses (in numbers of average incomes expressed as GDP per capita) by HDI bands from 2000 to 2023.

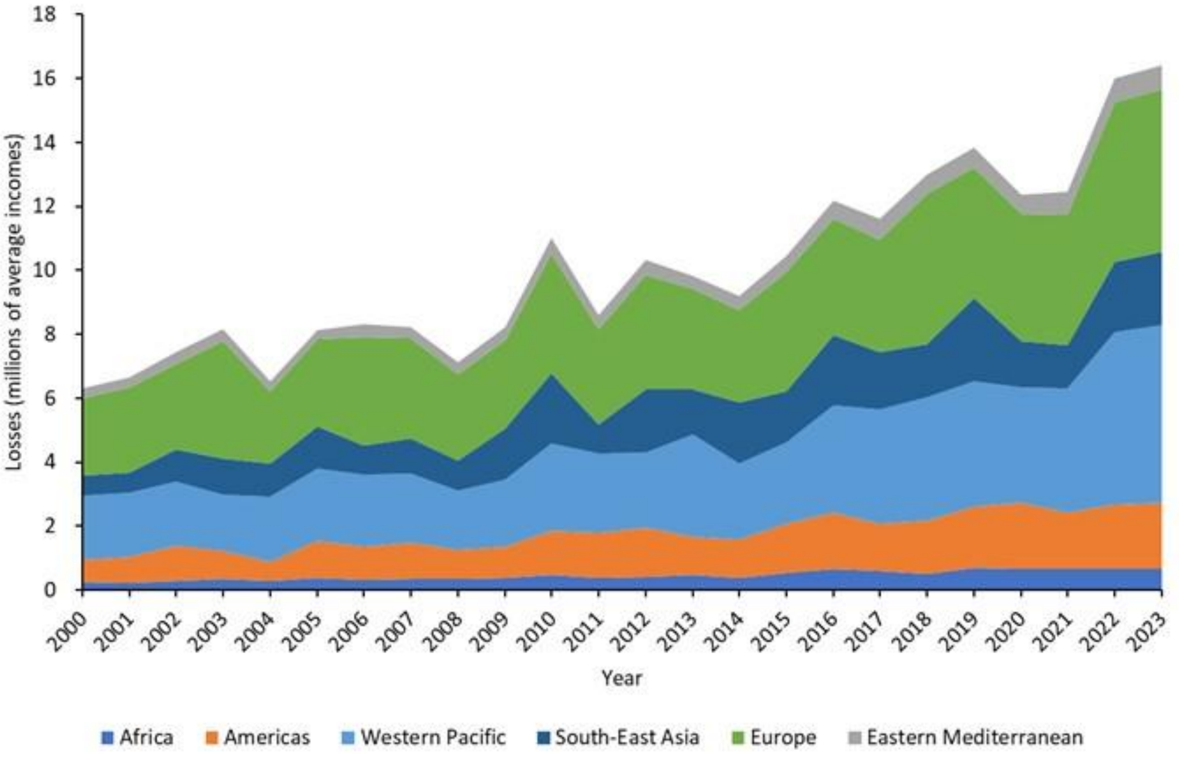

Figure 153: Monetised value of heat-related mortality losses (in numbers of average incomes expressed as GDP per capita) by WHO regions from 2000 to 2023.

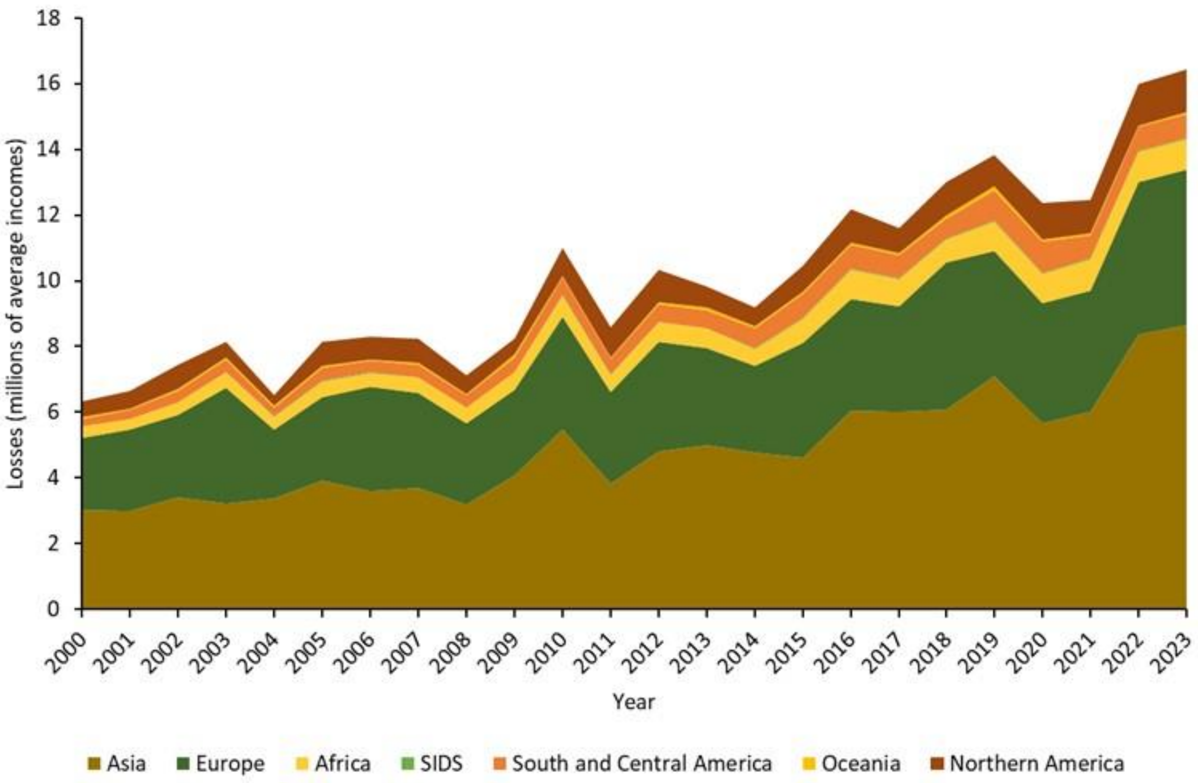

Figure 154. Monetised value of heat-related mortality losses (in numbers of average incomes expressed as GDP per capita) by Lancet Countdown groupings from 2000 to 2023.

Indicator 4.1.3: loss of earnings from heat-related labour capacity reduction

Indicator authors

Dr Daniel Scamman

Methods

Indicator 1.1.3 provides data on heat-related labour capacity loss, in terms of work hours lost (WHLs), at country scale across four sectors (services, manufacturing, construction and agriculture) for the years 1990-2022 inclusive. In order to calculate potential loss of earnings from this labour capacity loss, it was necessary to compile a dataset of average earnings per hour for each of these countries, sectors and years.

Earnings and income statistics were compiled from the ILOSTAT databases held by the ILO, within the category 'Statistics on Wages'.[353] ILOSTAT includes a number of indicators which are of potential relevance to deriving the average annual hourly wages for the required countries and years. There are variations in the coverage of these indicators, with none having an entirely comprehensive coverage of the countries, sectors and years required for this indicator. Multiple ILOSTAT indicators were therefore used to fill as many gaps as possible. The three main indicator sets used were:

- Mean nominal monthly earnings of employees by sex and economic activity: annual
- Mean nominal monthly earnings of employees by sex and occupation: annual
- Mean nominal hourly earnings of employees by sex and occupation: annual

Within each of these indicator sets, the employment activities most accurately reflecting the four required sectors were selected. In some cases, more than one such activity was available, due to different reporting conventions (for example, the set of activities under ISCO-08 being an update from ISCO-88). Full descriptions of ILO indicators and classifications are available on the ILOSTAT website.[354]

Each indicator and activity was available in US dollar and local currency units. US dollar units were preferred, however in each indicator and activity case, the number of returns in local currency units was slightly higher, so these were selected as well in case more data points could be covered by doing so.

The following tables set out for each of the four employment sectors, the ILOSTAT indicators and activity definitions that were selected in order to supply as much of used the required data as possible. In each table the indicator, activity and currency combinations are arranged in the order of preference with which they were used.

Table 101: Indicators, activity classes and currencies selected to gather data from the ILOSTAT databases on earnings in the services sector, in order of preference.

	Indicator	Activity	Currency
1	Mean nominal monthly earnings of employees by sex and economic activity: annual	Aggregate: Trade, transportation, accommodation and food, and business and administrative services	US Dollars
2		Aggregate: Trade, transportation, accommodation and food, and business and administrative services	Local currency
3	Mean nominal monthly earnings of employees by sex and occupation: Annual	ISCO-08: 5. Service and sales workers	US Dollars
4		ISCO-08: 5. Service and sales workers	Local currency
5		ISCO-88: 5. Service workers and shop and market sales workers	US Dollars
6		ISCO-88: 5. Service workers and shop and market sales workers	Local currency
7	Mean nominal hourly earnings of employees by sex and occupation: Annual	ISCO-08: 5. Service and sales workers	US Dollars
8		ISCO-08: 5. Service and sales workers	Local currency
9		ISCO-88: 5. Service workers and shop and market sales workers	US Dollars
10		ISCO-88: 5. Service workers and shop and market sales workers	Local currency
11	Mean nominal monthly earnings of employees by sex and economic activity: annual	ISIC Rev.4: N. Administrative and support service activities	US Dollars
12		ISIC Rev.4: N. Administrative and support service activities	Local currency
13		ISIC Rev. 3.1: K. Real estate, renting and business activities	US Dollars
14		ISIC Rev. 3.1: K. Real estate, renting and business activities	Local currency
15		ISIC Rev.2: 8. Financing, insurance, real estate and business services	US Dollars
16		ISIC Rev.2: 8. Financing, insurance, real estate and business services	Local currency

Table 102: Indicators, activity classes and currencies selected to gather data from the ILOSTAT databases on earnings in the manufacturing sector, in order of preference.

	**Indicator**	**Activity**	**Currency**
1	Mean nominal monthly earnings of employees by sex and economic activity: annual	Aggregate: Manufacturing	US Dollars
2		Aggregate: Manufacturing	Local currency
3		ISIC Rev.4: C. Manufacturing	US Dollars
4		ISIC Rev.4: C. Manufacturing	Local currency
5		ISIC Rev. 3.1: D. Manufacturing	US Dollars
6		ISIC Rev. 3.1: D. Manufacturing	Local currency
7		ISIC Rev.2: 3. Manufacturing	US Dollars
8		ISIC Rev.2: 3. Manufacturing	Local currency
9	Mean nominal monthly earnings of employees by sex and occupation: Annual	ISCO-08: 8. Plant and machine operators, and assemblers	US Dollars
10		ISCO-08: 8. Plant and machine operators, and assemblers	Local currency
11		ISCO-88: 8. Plant and machine operators and assemblers	US Dollars
12		ISCO-88: 8. Plant and machine operators and assemblers	Local currency
13	Mean nominal hourly earnings of employees by sex and occupation: Annual	ISCO-08: 8. Plant and machine operators, and assemblers	US Dollars
14		ISCO-08: 8. Plant and machine operators, and assemblers	Local currency
15		ISCO-88: 8. Plant and machine operators and assemblers	US Dollars
16		ISCO-88: 8. Plant and machine operators and assemblers	Local currency

Table 103: Indicators, activity classes and currencies selected to gather data from the ILOSTAT databases on earnings in the agricultural sector, in order of preference.

	**Indicator**	**Activity**	**Currency**
1	Mean nominal monthly earnings of employees by sex and economic activity: annual	Aggregate: Agriculture	US Dollars
2		Aggregate: Agriculture	Local currency
3		ISIC Rev.4: A. Agriculture; forestry and fishing	US Dollars
4		ISIC Rev.4: A. Agriculture; forestry and fishing	Local currency
5		ISIC Rev.3.1: A. Agriculture, hunting and forestry	US Dollars
6		ISIC Rev.3.1: A. Agriculture, hunting and forestry	Local currency
7		ISIC Rev.2: 1. Agriculture, hunting, forestry and fishing	US Dollars
8		ISIC Rev.2: 1. Agriculture, hunting, forestry and fishing	Local currency
9	Mean nominal monthly earnings of employees by sex and occupation: Annual	ISCO-08: 6. Skilled agricultural, forestry and fishery workers	US Dollars
10		ISCO-08: 6. Skilled agricultural, forestry and fishery workers	Local currency
11		ISCO-88: 6. Skilled agricultural and fishery workers	US Dollars
12		ISCO-88: 6. Skilled agricultural and fishery workers	Local currency
13	Mean nominal hourly earnings of employees by sex and occupation: Annual	ISCO-08: 6. Skilled agricultural, forestry and fishery workers	US Dollars
14		ISCO-08: 6. Skilled agricultural, forestry and fishery workers	Local currency
15		ISCO-88: 6. Skilled agricultural and fishery workers	US Dollars
16		ISCO-88: 6. Skilled agricultural and fishery workers	Local currency

Table 104: Indicators, activity classes and currencies selected to gather data from the ILOSTAT databases on earnings in the manufacturing sector, in order of preference.

	**Indicator**	**Activity**	**Currency**
1	Mean nominal monthly earnings of employees by sex and economic activity: annual	Aggregate: Construction	US Dollars
2		Aggregate: Construction	Local currency
3		ISIC Rev.4: F. Construction	US Dollars
4		ISIC Rev.4: F. Construction	Local currency
5		ISIC Rev. 3.1: F. Construction	US Dollars
6		ISIC Rev. 3.1: F. Construction	Local currency
7		ISIC Rev.2: 5. Construction	US Dollars
8		ISIC Rev.2: 5. Construction	Local currency
9	Mean nominal monthly earnings of employees by sex and occupation: Annual	ISCO-08: 9. Elementary occupations	US Dollars
10		ISCO-08: 9. Elementary occupations	Local currency
11		ISCO-88: 9. Elementary occupations	US Dollars
12		ISCO-88: 9. Elementary occupations	Local currency
13	Mean nominal hourly earnings of employees by sex and occupation: Annual	ISCO-08: 9. Elementary occupations	US Dollars
14		ISCO-08: 9. Elementary occupations	Local currency
15		ISCO-88: 9. Elementary occupations	US Dollars
16		ISCO-88: 9. Elementary occupations	Local currency

A spreadsheet tool was developed to select the relevant data points for all available countries in order of indicator preference – if there was no data point for a given country, year and sector in the first priority indicator, the data point was sought in the next indicator, and so on until a data point was found, or all indicators had been tried.

Monthly earnings data were converted to hourly values using a standard assumption of 40 hours per week and 4.33 weeks per month, i.e., 173.2 hours per month.[355]
Data in nominal local currency units were converted to nominal US dollars at market exchange rates using IMF International Financial Statistics. Nominal US dollar values were converted to real 2022 US dollar values using the US dollar consumer price index from the IMF World Economic Outlook database.

Even after searching 16 variations of ILO indicator, activity and reporting currency for each sector, there were still considerable gaps, with around two thirds of required data points unfilled. In addition, there was a small number of clearly erroneous data points – e.g., with hourly earnings rates orders of magnitude too high, possibly caused by incorrect recording of the currency in which the data were reported, or by episodes of rapid inflation and currency devaluation, with which the recorded market exchange rates were not keeping track.

In order to fill the gaps with no data, as well as to correct data points that were clearly erroneous, a gap filling process was undertaken, using other data points to stand in for the missing or erroneous data. This process was undertaken after all of the data had been corrected to real 2023 US dollar values, so that all of the data were already expressed in constant values. Wherever possible, gaps were filled using data from a different year but from the same sector and country. Where data were available in years before and after the gaps in the same sector and country, linear interpolation was used to fill the gaps. If no future year was available, data were filled using the nearest past year. Likewise, if no previous year was available, the nearest future year was used. If there were no data points available at all for a certain sector or country, the data were taken from the same sector of a different country that was as comparable as possible to the country with missing data. Identification of a reasonably comparable country was achieved primarily by selecting one as close as possible on the current HDI scale, within the same or similar region and current World Bank Income Group (WBIG), of a similar population, and with a reasonable number of datapoints. It should be recognised, however, that countries that currently have similar HDI or WBIG bandings could have had quite different bandings in the past.

A small number of countries with no wage data or HDI value could not be included in the analysis as no suitable substitute could be found.

This process resulted in estimates of hourly earnings for the four sectors for 182 countries for the years 1990-2023 inclusive (the period for which WHL data are available from indicator 1.1.4). These hourly earnings data were multiplied by the corresponding values for work hours lost (WHL) in each country, sector, and year, to provide a quantification of potential earnings lost. The WHLs used assumed that work in the agricultural and construction sectors took place in the sun.

These total lost earnings were expressed as a percentage of the country's GDP in each relevant year. GDP data in nominal US dollars at market exchange rates were downloaded from the IMF World Economic Outlook database, and rendered in constant 2023 US dollars using the GDP deflator index from the same source. Gaps in this GDP data for some countries and years imposed a small further restriction on the coverage of this indicator, and not all of the same countries are available for all years. The maximum country-coverage of the indicator is 183 countries, during the years 2002–2023 inclusive. Results are presented as the average value for countries in each of the HDI bandings, WHO regions and *Lancet* Countdown (LC) regions.

Data

- Data on working hours lost from indicator 1.1.3
- Data on earnings by country and sector from ILOSTAT.[353]
- Exchange rate data from IMF International Financial Statistics.[344]
- US Dollar CPI and GDP deflator index from the IMF World Economic Outlook database.[343]
- Country GDP data from the IMF World Economic Outlook database.[343]
- World Bank Income Groups.[356]

Caveats

There are several important caveats associated with the analysis:

- The ILOSTAT data do not cover all of the countries, years and sectors required, hence some gap filling was required, as described above. Whilst reasonable care has taken to identify appropriate estimates, gaps filled in the data are subject to uncertainties
- Whilst reasonable efforts have been made to correct for clearly erroneous data points, the analysis is dependent on the reliability of the ILOSTAT data, which could be subject to uncertainties in reporting, collection and processing
- The use of different combinations of ILOSTAT indicators and activity classes, rather than one single indicator and one activity class per sector, was necessary to increase data coverage as much as possible. Nonetheless this entails risks of inconsistencies, for example associated with different classifications and reporting methods
- The conversion of monthly data to hourly was carried out on the basis of a standard assumption of 4.33 weeks per month, and 40 hours per week. Real monthly working times will vary from these assumptions to a greater or lesser extent in different countries

All of these issues mean that caution should be exercised when examining results for any particular country. In addition, it must be emphasised that the results produced are the *potential* loss of earnings, rather than actual. The indicator is not based on evidence as to whether time off work was in fact taken. Further, if time was taken off work, the bearer of the costs of the lost labour could have varied between countries and sectors. In some instances, workers may have been able to claim sick pay, in which case the losses would have been borne by the employer through paying for non-productive time. In other instances, no arrangements for sick pay may have been in place, in which case it would have been the worker who would have borne the cost through a direct loss of earnings due to the inability to work.

Finally, the indicator by definition is an estimate of potential loss of earnings from formal paid sectors. In many countries informal and unpaid labour is also significant. Such activities could include domestic work and small-scale agriculture. The impacts on productivity and health of extreme heat on workers involved in so-called informal sectors, would be in addition to the monetised estimates quantified by this indicator.

Additional analysis

The following graphs present the results according to HDI, WHO, and LC groupings and also changes in results 1990-2023 according to HDI group (Figure 155, Figure 156, Figure 157, Figure 158, Figure 159, and Figure 160).

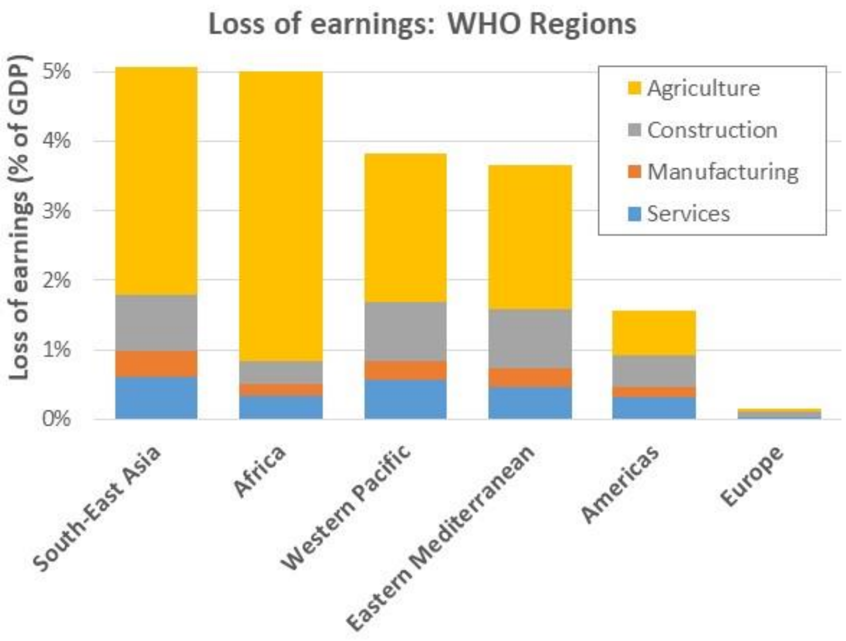

Figure 155: Average potential loss of earnings from heat-related labour capacity reduction in 2022 as a share of GDP according to WHO region and sector of employment

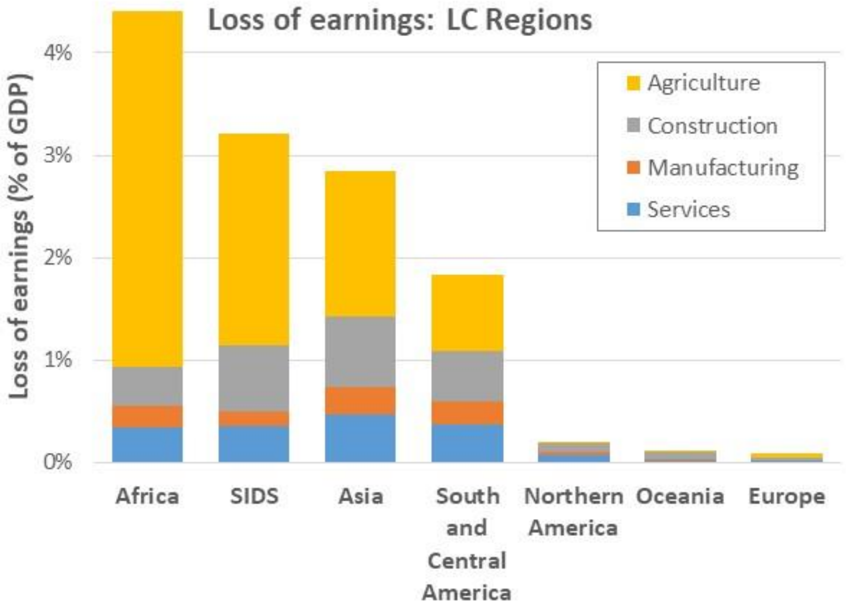

Figure 156: Average potential loss of earnings from heat-related labour capacity reduction in 2022 as a share of GDP according to LC grouping and sector of employment

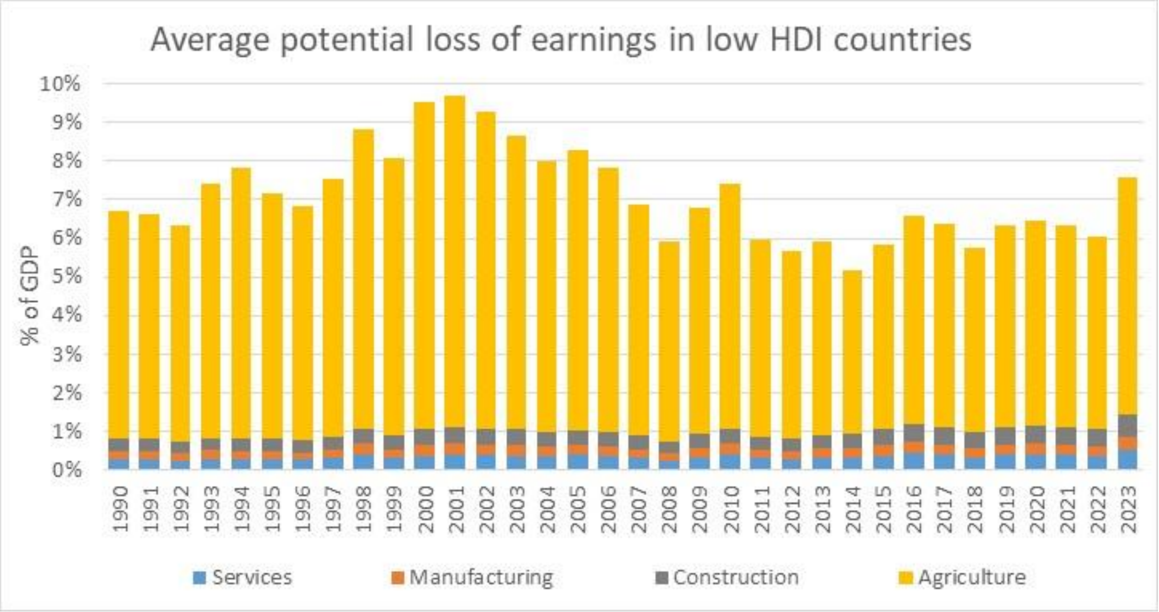

Figure 157: Average potential loss of earnings from heat-related labour capacity reduction as a share of GDP for low HDI countries, by sector of employment

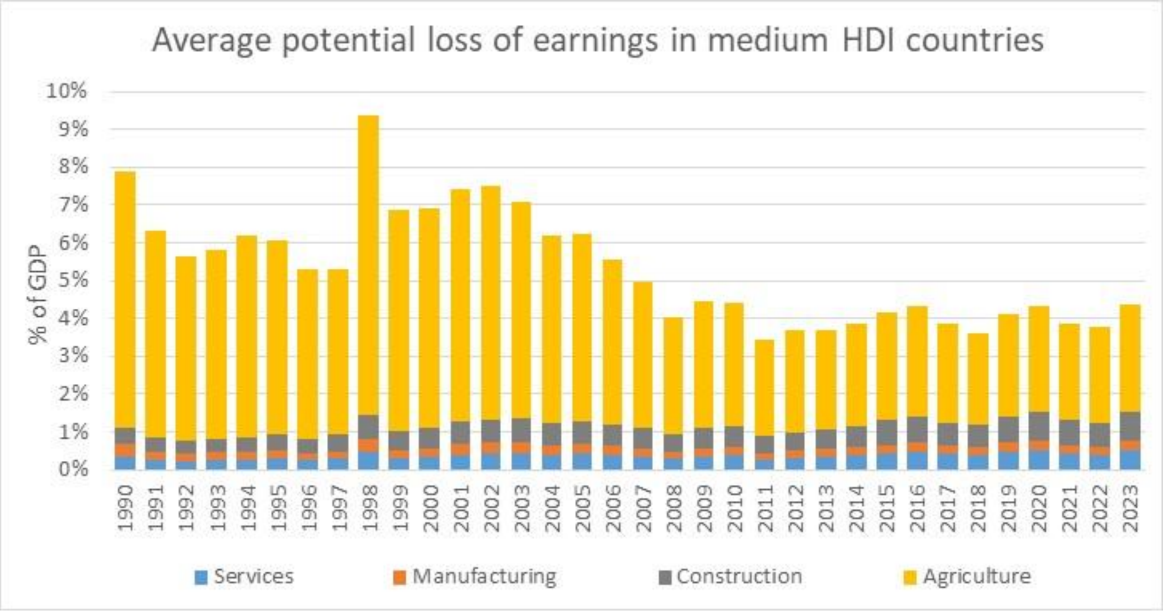

Figure 158. Average potential loss of earnings from heat-related labour capacity reduction as a share of GDP in medium HDI countries, by sector of employment.

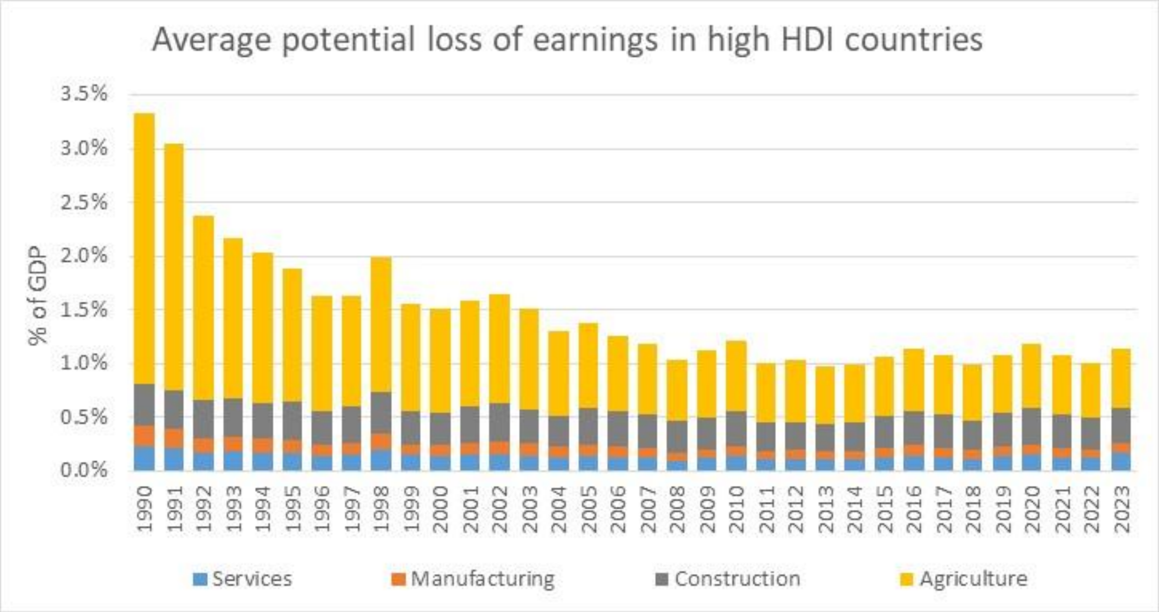

Figure 159. Average potential loss of earnings from heat-related labour capacity reduction as a share of GDP in high HDI countries, by sector of employment.

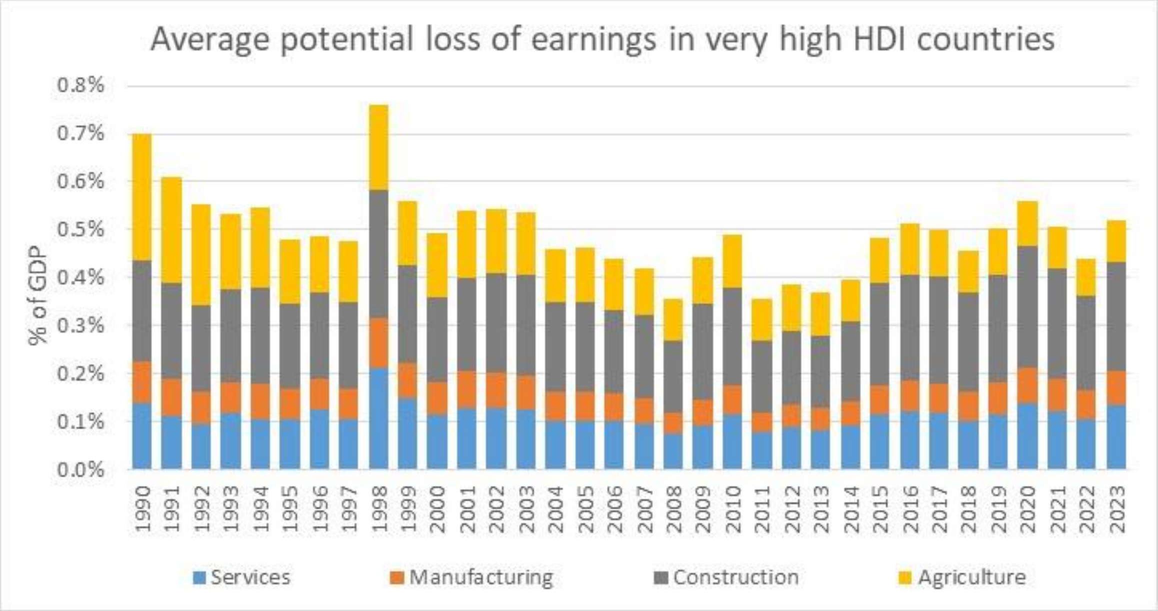

Figure 160. Average potential loss of earnings from heat-related labour capacity reduction as a share of GDP for very high HDI countries, by sector of employment.

Indicator 4.1.4: costs of the health impacts of air pollution

Indicator authors

Dr Daniel Scamman, Dr Gregor Kiesewetter

Methods

This indicator uses input data from Indicator 3.2.1, which provides data on deaths attributable to both natural and anthropogenic ambient air pollution. YLLs were calculated for 140 individual countries for all the years between 2007 and 2021. Each country was then classified according to its HDI category, WHO region and LC grouping. For the WHO region and LC grouping calculations, the YLL data for 43 additional countries in three 'rest of world' regions from the GAINS model were also included (see Table 105), though population and GDP data had to be excluded for some of these countries where data was unavailable as shown. From 2023, three countries in a fourth GAINS region (Former Soviet Union) were included as separate countries (Tajikistan, Turkmenistan and Uzbekistan). Also the US Virgin Islands have been included in the Americas WHO region, and for the LC groupings French Guiana is included in the SIDS region rather than South and Central America, and Belize in South and Central America rather than SIDS. It was not possible to include countries in the three GAINS regions in the HDI classification, due the heterogeneity of classifications of the countries that constitute each region.

The YLLs for each category and region were then summed. To determine the economic value of the YLLs for each category and region relative to per capita average annual income in each, the results were multiplied by the fixed ratio of the Value of a Statistical Life Year (VSLY) to GDP per capita derived by indicator 4.1.2. To calculate the economic value of the YLLs relative to total GDP for each year, the results of this first calculation were multiplied by average GDP per capita (calculated from the sum of GDP for each category and region, inflated to $2023 from current prices, divided by the sum of the population for each category and region), and then divided by the sum of GDP in $2023 for the category or region in question.

GDP and GDP inflator data were taken from the International Monetary Fund (IMF), and population data were taken from the United Nations (UN).[343,357] This was supplemented by GDP and population data from the World Bank for Antigua and Barbuda, Aruba, Bahrain, Barbados, Brunei Darussalam, Cayman Islands, Cuba, Curacao, Dominica, Grenada, St. Lucia, Malta, Singapore, Somalia, Syria, US Virgin Islands, and West Bank and Gaza.[351,358] The data and methods used to calculate the fixed ratio between VSLY and GDP per capita are described in indicator 4.1.2.

Table 105: Countries in GAINS model 'rest of word' region group included in the calculation of costs of air pollution for WHO and LC groupings.

GAINS Region	WHO Region	LC Group	Country
Caribbean (CARB)	Americas	SIDS	Anguilla*, Antigua and Barbuda, Aruba, Bahamas, Barbados, British Virgin Islands*, Caribbean Netherlands*, Cayman Islands, Cuba, Curaçao, Dominica, Dominican Republic, French Guiana*, Grenada, Guadeloupe*, Guyana, Haiti, Jamaica, Martinique*, Puerto Rico, Saint Lucia, Saint Vincent and the Grenadines, Suriname, Trinidad and Tobago, United States Virgin Islands
Central America (CEAM)	Americas	South and Central America	Belize, Costa Rica, Guatemala, Honduras, Nicaragua, Panama, El Salvador
Middle East (MIDE)	Eastern Mediterranean	Asia	United Arab Emirates, Bahrain, Iraq, Jordan, Kuwait, Lebanon, Oman, Occupied Palestinian Territory, Qatar, Syrian Arab Republic, Yemen

*Population and GDP excluded from the calculations due to lack of data of either one or other data point.

Data

- IMF World Economic Outlook (October 2023).[343]
- UN World Population Prospects.[357]
- World Bank GDP and Population data.[351,358]

Caveats

See Indicator 3.2.1, for caveats related to the calculation of reduced life expectancy.

Caveats regarding the calculation of VSLY are discussed under indicator 4.1.2. Countries not listed in Table 105 above have been excluded from the analysis, due to the lack of individual characterisation in the GAINS model used to calculate YLLs. Countries in Table 105 with an asterisk (*) have had their YLLs included in the GAINS Regions, but have not had their GDPs and Population included in this indicator due to a lack of data, but as mostly small countries this effect will be small. Democratic People's Republic of Korea is excluded from the analysis due to the lack of reliable GDP data. Somalia is excluded from the HDI analysis as it does not have a HDI classification. Data for previous years differs to those presented in earlier reports due to updated data.

Additional analysis

The monetised losses from premature mortality due to air pollution according to HDI group, WHO region and LC group is shown in Figure 161, Figure 162 and Figure 163 respectively.

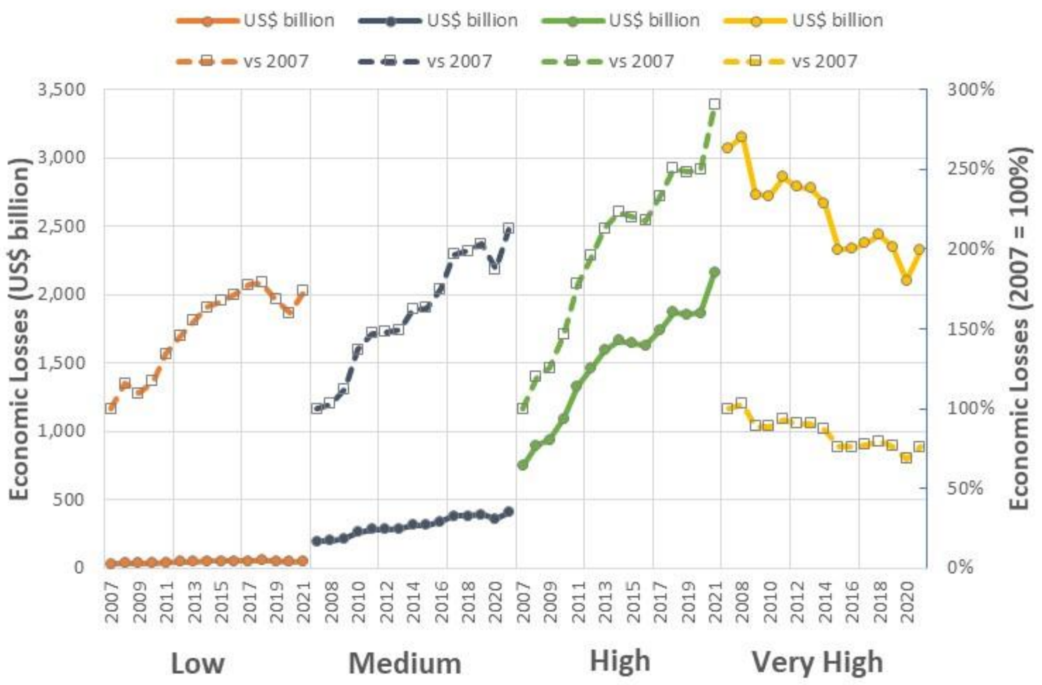

Figure 161: Monetised losses from premature mortality due to air pollution according to HDI group (2007-2021). Solid lines show absolute losses in US$ billion, dashed lines change since 2007 (2007 = 100%).

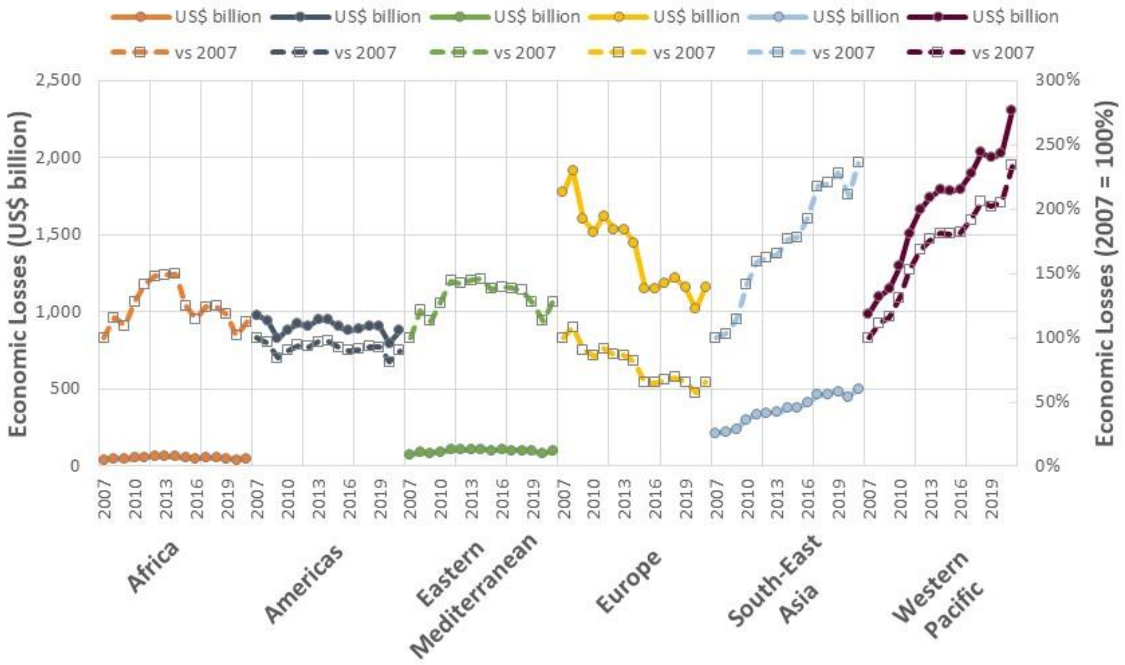

Figure 162: Monetised losses from premature mortality due to air pollution according to WHO region (2007-2021). Solid lines represent absolute losses in US$ billion, dashed lines change since 2007 (2007 = 100%).

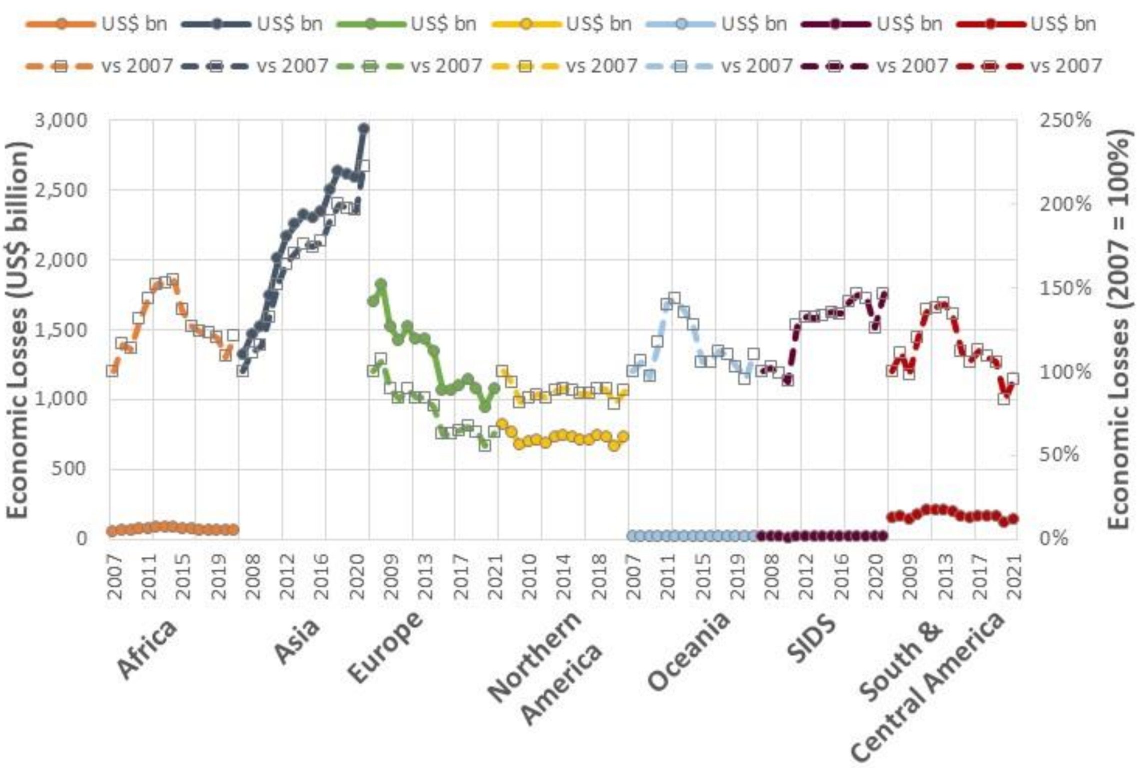

Figure 163: Monetised losses from premature mortality due to air pollution according to LC group (2007–2021). Solid lines show absolute losses in US$ billion, dashed lines change since 2007 (2007 = 100%).

4.2: The transition to net zero-carbon, health-supporting economies

Indicator 4.2.1: employment in low-carbon and high-carbon industries

Indicator authors

Dr Daniel Scamman

Methods

The data for this indicator is sourced from IRENA (renewables) and IBISWorld (fossil fuel extraction). Renewable industries included are:

- Hydropower;
- Solar heating/cooling;
- Solar photovoltaic;
- Wind energy;
- Bioenergy;
- Other technologies.

Bioenergy includes liquid biofuels, soil biomass and biogas. ‘Other technologies’ includes geothermal energy, ground-based heat pumps, concentrated solar power, municipal and industrial waste, and ocean energy. Fossil fuel extraction values include direct employment, whereas renewable energy jobs include direct and indirect employment (e.g., equipment manufacturing), except for large hydropower (direct employment only).

Data

- Data for employment in renewables from IRENA.[359]
- Data for employment in fossil fuel extraction from IBISWorld: oil and gas exploration and
- production; and coal mining.[360,361]

Caveats

Fossil fuel extraction values include direct employment, whereas renewable energy jobs include direct and indirect employment (e.g., equipment manufacturing), with the exception of hydropower. Historic IBISWorld data can change noticeably from year to year if a new analyst changes the methodology.

Additional analysis

Table 106 shows employment in renewable energy and fossil fuel extraction industries from 2012 to 2022.

Table 106: Employment in renewable energy and fossil fuel extraction industries.

Million Jobs	2012	2013	2014	2015	2016	2017	2018	2019	2020	2021	2022
Solar Photovoltaic	1.36	2.27	2.49	2.77	3.09	3.37	3.68	3.75	3.98	4.29	4.90
Bioenergy	2.4	2.5	2.99	2.88	2.74	3.05	3.18	3.58	3.52	3.44	3.58
Hydropower	1.66	2.21	2.04	2.16	2.06	1.99	2.05	1.96	2.18	2.37	2.49
Wind Energy	0.75	0.83	1.03	1.08	1.16	1.15	1.16	1.17	1.25	1.37	1.4
Solar Heating/Cooling	0.89	0.5	0.76	0.94	0.83	0.81	0.8	0.82	0.82	0.77	0.71
Other Technologies	0.22	.023	0.19	0.2	0.24	0.16	0.18	0.18	0.27	0.45	0.64
**Renewables Total**	**7.28**	**8.54**	**9.5**	**10.03**	**10.12**	**10.53**	**11.05**	**11.46**	**12.02**	**12.69**	**13.72**
**Fossil Fuel Extraction**	**12.49**	**12.67**	**12.66**	**12.75**	**13.17**	**13.06**	**12.94**	**12.56**	**11.96**	**11.78**	**11.82**

Indicator 4.2.2: compatibility of fossil fuel company strategies with the Paris Agreement

Indicator authors

Dr Daniel Scamman

Methods

Absolute emission targets

Carbon dioxide (CO_2) is responsible for around three-quarters of total greenhouse gas emissions as measured in gigatonnes of CO_2-equivalent.[362] Climate change has multiple direct impacts on human health, as identified throughout the *Lancet* Countdown. Hence reducing CO_2 emissions from fossil fuels in order to reduce climate change will bring about significantly improved health outcomes. This indicator connects CO_2 emissions to the activities of major oil and gas (O&G) companies that extract these fossil fuels and analyses the extent to which their future production plans are consistent with the need to reduce CO_2 emissions in order to avoid dangerous climate change. In particular, this indicator focuses on production projections based on actual corporate activities, which may not always reflect declared targets or aspirations. Those companies whose business strategies fall short of what is required to be compliant with climate change targets can be said to be posing a danger to public health.

The indicator tracks the gap between the projected production of oil and gas companies based on their actual activities, and production trajectories consistent with the Paris target of 1.5°C of warming. The indicator is expressed as a percentage of the projected production that each company is above or below a pathway consistent with the Paris targets. If the indicator value is positive, the company projection is above the climate-consistent plan, and therefore not consistent with the climate target. The indicator analyses both international, publicly traded oil companies (IOCs) and national oil companies (NOCs), which in many cases have larger production volumes than IOCs but are subject to less public or shareholder scrutiny.

A number of organisations analyse the activities of oil and gas companies relative to climate targets, many of them aimed at investors. The Transition Pathways Initiative (TPI) publishes an annual assessment of around sixty large publicly owned O&G companies. However their data is based on companies' own disclosures and reports, so may be more aspirational rather than based on actual production projections, and excludes some of the large state-owned NOCs.[363] The Science-Based Targets Initiative (SBTi) helps businesses set science-based emissions reduction targets, but these are based on companies' own submissions rather than objective assessments of actual activity.[364] The annual Production Gap Report (PGR) tracks the discrepancy between fossil fuel production and climate-consistent production levels, but focuses on 15 countries rather than O&G companies, and relies on government production projections from national energy outlooks and targets.[365] Climate Action 100+ produce an annual Net Zero Company Benchmark;[366] their indicators primarily focuses on ambition, governance and disclosure, but a capital allocation alignment indicator generated by Carbon Tracker Initiative (CTI) evaluates the alignment of company actions with the Paris Agreement. CTI also publish an annual Two Degrees of Separation report determining potential transition risk exposure for upstream oil and gas companies, finding that the asset stranding risk of unsanctioned (pre-FID) assets is severe.[367] In 2020 Oil Change International (OCI) published production projections for eight IOCs, but this was a one-off report restricted to a small number of companies.[368] A recent journal paper found a discrepancy between the discourse and actions of four oil majors, but focussed on historic actions only.[369]

It is best practice in assessing corporate strategies to consider both absolute and intensity emission targets.[370–372] This prevents a situation where a company scores favourably by reducing its absolute emissions, but merely because its production is decreasing while its emission intensity (kgCO_2/MJ) could actually be increasing. Or where a company improves its emission intensity but releases more emissions overall because its production is growing. However, an emissions intensity target is not considered here due to the challenges in projecting improvements in operational efficiency (e.g., reduced flaring or leakage) or transitions to lower-carbon fuels such as renewables or nuclear. Instead, this indicator uses absolute reduction targets, which are the most meaningful for reducing global total atmospheric emissions.[370]

Emission Benchmarks

An internationally-recognised standard for reporting emissions used by many companies is the GHG Protocol Corporate Standard, developed in 2004 by the World Resources Institute (WRI) and World Business Council for Sustainable Development (WBCSD).[373,374] This divides emissions into Scope 1 (direct emissions), Scope 2 (indirect energy emissions) and Scope 3 (other indirect emissions). Scope 3 emissions (and Use of Sold Products in particular) can be much higher than Scope 1 and 2 emissions (*e.g.* 86% of total emissions of European companies recently reported to the Carbon Disclosure Project, CDP), particularly for energy companies.[375,376]

Corporate benchmarks for companies operating in different sectors can be established using the Sectoral Decarbonisation Approach (SDA), developed in 2015 by the CDP, WRI, and WWF[371] and used by organisations including TPI and SBTi.[370,377] However the SDA is primarily designed for Scope 1 and 2 emissions, and most companies focus their efforts on scopes 1 and 2 emissions over which they have more direct control.[372] Also, the SDA is intended primarily to help companies in homogenous energy intensive sectors (sectors that can be described with a single physical indicator) rather than the oil and gas sector,[370] with SBTi regarding science-based emission reductions for fossil fuel companies as complex.[378] TPI have generated a sectoral decarbonisation pathway for the O&G sector, but this is an emissions intensity pathway which is not being considered for this indicator.[377] Paris-compliant least-cost pathways typically generate projections for future global oil and gas production. This oil and gas has to be produced by O&G companies, so this production data can be used to represent the pathway O&G companies must take to be Paris-compliant. In addition, this production data (as opposed to consumption) can be assumed to cover Scope 1, 2, and 3 emissions. Hence this indicator uses oil and gas production data emanating from Paris-compliant modelling to generate Paris-compliant benchmarks for the O&G industry, rather than an SDA approach that generates separate Scope 1, 2, and 3 emission targets.

O&G sector benchmarks are typically derived using climate-compliant pathways from either the IEA or the IPCC. SBTi selected 20 1.5°C scenarios from an ensemble of over 400 peer-reviewed IPCC pathways for their analysis.[379–381] Likewise the Production Gap Report is based on a grouping of 19 IPCC 1.5°C scenarios.[365] Alternatively TPI, Carbon Tracker and OCI used IEA pathways;[367,368,377] some considered both. IEA scenarios provide a greater amount of sectoral granularity,[380] and this indicator (in alignment with TPI) uses the IEA’s Net Zero Emissions by 2050 (NZE) scenario for its 1.5°C scenario from the IEA’s World Energy Outlook (WEO) report. The NZE scenario in the 2021 WEO report[382] was used as the 1.5°C benchmark for the 2022 *Lancet* Countdown report, the NZE scenario in the 2022 WEO report[383] was used for the 2023 *Lancet* Countdown report, and now the NZE scenario in the 2023 WEO report[384] was used for the 2024 *Lancet* Countdown report. The updated NZE reaches net zero emissions in 2050 and is consistent with limiting the global

temperature rise to 1.5°C without a temperature overshoot; 1.5°C refers to the median temperature rise, meaning there is a 50% probability of remaining below 1.5°C. Table 107shows a comparison between fossil fuel supply in the 2021, 2022 and 2023 versions of the NZE; the 2023 NZE shows increases in the supply of coal, oil and gas in 2030 compared to the 2022 NZE to reflect the growing challenge of reducing emissions by 2030. Fossil fuel supply then drops more rapidly than previously in the 2030s and 2040s to reach the same total supply in 2040 as in the 2022 NZE, and going on to reach a lower total in 2050. Overall fossil fuel supply is still substantially greater than zero in 2050, indicating a significant reliance on offsetting technologies in other sectors.

Table 108: Comparison of fossil fuel supply in 2021, 2022 and 2023 IEA NZE scenarios.[382–384]

		2021 NZE scenario				2022 NZE scenario				2023 NZE scenario		
		**2020**	**2030**	**2040**	**2050**	**2020**	**2030**	**2040**	**2050**	**2030**	**2040**	**2050**
Coal	EJ	154	72	32	17	157	89	27	16	95	25	15
Oil	EJ	173	137	79	42	172	143	76	40	148	79	42
Natural gas	EJ	137	129	75	61	140	113	54	40	118	53	32
**Total**	**EJ**	**464**	**338**	**186**	**120**	**469**	**345**	**157**	**96**	**362**	**157**	**88**

Emission Projections

A company's future oil and gas production (and hence emissions) can be estimated from changes in its reserves and investments as recorded in their company reports. However this is a challenging and complex process, for example with different definitions of reserves in different regions, reserves being bought and sold according to market conditions, and an observed recent sector-wide reduction in reserve holdings.[385] Hence a number of analyses base their future production projections on data from Rystad Energy,[366–368] an independent oil and gas consultancy that maintains a database of every oil and gas project in the world.[386] Historical and projected production data were downloaded for this indicator from the Rystad Energy Ucube database on 6th March 2024, and compared to historical Rystad data from March 2023 to determine the change over one year. Total oil and gas production was included (i.e., Crude Oil, Condensates, NGLs, Refinery Gains, Other Liquids and Natural Gas). Likewise production from all company types were included (Major, Integrated, Independent, NOC, INOC, Operating Company, E&P Company, Exploration Company, Industrial, Investor, Supplier and Unspecific). Data for the largest 1,000 companies were extracted from Rystad, accounting for over 99% of Rystad's projected production. Of these, it was found that the largest 114 companies accounted for over 80% of projected 2040 production, and these were included for analysis. The bulk of the remaining 20% production was classified as Unspecified (Open acreage, Other liquids, Other partner(s), Refinery Gains, Relinquished or Unknown Owner) i.e. unable to be allocated to an existing company, and hence not able to be included in the analysis. Total Rystad historical production for 2015-2021 was found to be within 10% of IEA World Energy Balance data,[387] with the difference likely to be due to refinery losses, definitions and inclusions, or some combination of the above.

Each company needs its own benchmark pathway against which its projected production is assessed. This is generated by assigning each company its own market share, based on its average market share over the historical period 2015-2023 relative to the Rystad total production for each year. The company is then allocated this fraction of the future oil and gas production trajectory contained in the NZE scenario. Typically this market share is assumed to be constant over time,[371,377] though

uncertainties over changing market shares may limit targets to, for example, 15 years ahead.[371] Fixing market shares to current levels allows the future performance of a firm to be compared; if a firm's market share increases, this indicates its non-compliance has increased relative to its peers, whereas compliance improves if its share falls. Rystad data indicated that some firms changed market share slightly over the period 2015-23, but many remained at similar levels. Rystad projections for the future indicated that projected productions for many firms relative to each other remained relatively stable, suggesting that many firms can be expected to rebound from short-term volatility in production.

The effect of uncertainties over future market shares for individual firms can be reduced by assessing firms in groups. Here, companies are grouped into publicly listed International Oil Companies (IOCs), including the widely known Oil Majors, and state-owned National Oil Companies (NOCs) that in many cases have higher production levels but lower scrutiny than IOCs. In addition to the 114 companies included to cover 80% of projected production in 2040, the pathways for the twenty largest O&G firms by projected 2040 production were also plotted; these included eleven NOCs and nine IOCs, responsible for 34% and 15% respectively of total market share for 2015-23 (48% overall).

Data

- Oil and Gas firm production projection data from Rystad Energy.[386]
- 1.5°C NZE pathway from IEA World Energy Outlook 2023.[384]
- Historical oil and gas production data from IEA World Energy Balances 2022.[387]

Caveats

There are several caveats to consider with this indicator.

The IEA NZE benchmark used in this analysis only have 50% probability of maintaining temperatures below the 1.5°C target. Although typical for this sort of analysis, it needs to be remembered that, even if O&G firms follow the Paris-compliant pathways outlined here, there is still a substantial chance that temperature targets will be exceeded.

This indicator uses projections of future production of O&G firms from the Rystad Energy database. Although a leading database in the sector, there is a significant possibility that O&G firms will follow different projection pathways to the ones projected by Rystad. These uncertainties are likely to increase over time, meaning projections in the long-term are less certain than in the shorter-term. O&G firms are assumed here to have constant market shares. This assumption is typical for this sort of analysis but can be expected to introduce errors for at least some firms that increase over time, especially smaller firms. This can be at least partly addressed by aggregating firms into groupings such as IOCs and NOCs.

Future form of the indicator

In upcoming years, this indicator will monitor the extent to which oil and gas company strategies are compliant with the goals of the Paris Agreements, as production strategies change.

Additional Analysis

Of the 114 companies included in the indicator in this year's report, responsible for over 80% of projected production in 2040, state-owned national O&G companies (NOCs) are on track to exceed their share of emissions compatible with 1.5°C by 51% in 2030, and 184% in 2050 (Figure 115). Publicly-listed international O&G companies (IOCs) are further off-track, poised to exceed their 1.5°C-compatible share by 71% in 2030 and 198% in 2040. Eight of the largest nine companies were NOCs, which together were projected to generate 30.2% of global production in 2040, exceeding their 1.5°C-compatible share by 226%; these were Saudi Aramco, Gazprom, NIOC (Iran), PetroChina, Rosneft, Kuwait Petroleum Corp (KPC), ADNOC and QatarEnergy. Cumulative production from 2020 to 2040 of the group of 114 companies is projected to exceed their 1.5°C-compatible share by 61%.

This year, an analysis was also performed of how company strategies changed since the Paris agreement came into effect by comparing projected 2040 from March 2024 with November 2016. The largest 40 O&G companies by projected 2040 production in March 2024 were included in the analysis, with both snapshots compared to the 2023 NZE. Firms that acquired other businesses or assets between 2016 and 2024 were compared against the 2016 projections of the original firm, as acquisitions were interpreted as an attempt to increase production despite climate targets. It should also be noted that firms that decreased projected production may simply have sold assets to other businesses rather than decommissioning them. The results of this analysis are shown in the main report. It was found that thirty-four of these 40 companies showed an increase in their projected 2040 excess production since 2016, and for 16 of these companies this increase was over 100%. The median excess production in 2040 for these 40 companies increased from 102% in 2016 to 197% in 2024, i.e., it nearly doubled. These results indicate that the strategies of the largest O&G companies have increasingly deviated from a pathway consistent with 1.5°C of heating since the Paris Agreement was signed, in contravention of the goals of the Agreement.

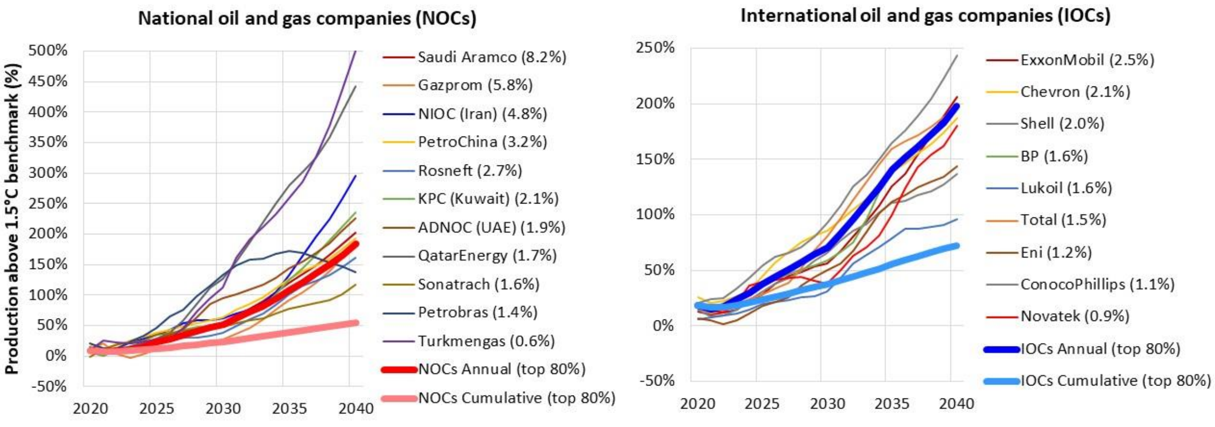

Figure 164: Compatibility of production strategies of the twenty largest oil and gas companies with the Paris 1.5°C climate target. Percentages in brackets in the legend represent average 2015-2023 global market share. Also shown are the average annual excess productions of the largest O&G companies totalling 80% of projected production in 2040 for both NOCs and IOCs (a total of 114 companies), and the cumulative production from 2020.

Indicator 4.2.3: stranded coal assets from the energy transition

Indicator authors

Prof Chi Zhang, Prof Wenjia Cai, Bo Li

Methods

Stranded assets are defined as coal-fired power generation units that are no longer able to obtain expected economic returns due to the transition to a low-carbon economy and end operation before their economic life. Stranded assets valuation methods mainly include Net Book Value (NBV),[388389390391] Net Present Value method (NPV),[392393] and Cost method,[394] etc. The Overnight Cost of Capital method from the Net Book Value (NBV) method, which can estimate stranded assets at the factory level in various countries globally with key parameters, represents a concise estimation approach. Upon comparing the advantages and disadvantages of the NPV and NBV methods, it is observed that the NPV method involves relatively complex parameter selection. Previous studies using the NPV method have only evaluated one country or region due to limitations on the availability of data. Furthermore, specific data for over 50 countries' factory-level parameters are often unavailable, such as dynamic changes in electricity prices, factory-specific discount rates, capacity factors, capital, operations, maintenance, and energy costs. The present values of these parameters are commonly undisclosed proprietary information, and forecasting their future values is uncertain. In contrast, the NBV method requires only a few key parameters to estimate stranded assets at the factory level for various countries globally; hence, it is believed that the NBV method is more appropriate for this indicator. Combined with global plant-level data availability considerations, the NBV method is adopted to design a lowest-stranded assets phase-down roadmap in order to get a lower bound of the scale of stranded assets.

Stranded assets of a coal-fired power unit are estimated by multiplying the percentage of its remaining lifespan relative to the expected lifespan with the capital expenditure of the coal-fired power plant. Stranded assets calculated by the NBV method are expressed as:

$$Stranded\ Assets = OCC * K * (L - R)/L$$

Where OCC refers to the country's overnight cost of capital (unit: US$/kW) and serves as a reference value for estimating the capital expenditure of the coal-fired power plant. K is the installed capacity scale of coal-fired units (unit: kW). L is the expected lifespan of coal-fired units (unit: years). According to the estimates from the Global Energy Monitor (GEM)[395] , the expected lifespan of coal-fired power units (L) is 40 years. R is the retirement age due to premature retirement (unit: years).

Data

- Geographic information comes from Global Energy Monitor[395] and Open Street Map.[396]
- The 1.5°C SSP1-1.9 (Shared Socioeconomic Pathways) scenario comes from the SSP Database (Version 2.0) hosted by IIASA.[397] Considering the reality, the stranded assets are assessed using the 1.5°C pathway, corresponding to the SSP1-1.9 scenario.[398,399]
- The Overnight Cost of Capital (OCC) for coal-fired power plants in 40 countries have been obtained from the International Energy Agency (IEA) and the International Renewable Energy Agency (IRENA). The Overnight Capital Cost data for the European Union, the

United States, China, Japan and India can be accessed on the Key Input Data page of the Global Electricity Cost (GEC) model from the IEA.[400] For Australia, Indonesia, South Africa, Brazil, Malaysia, Turkey, the Philippines, South Korea, and Vietnam, the Overnight Capital Cost data is available from IRENA.[401] For the remaining countries, their OCC is estimated based on the Human Development Index (HDI)[402] or GDP per capita[403] by using OCC data for 40 countries. Firstly, the current 50+ countries are ranked according to their HDI[402] or GDP per capita[403], and then compare these indicators with the remaining countries. If the remaining countries have similar HDI[402] or GDP per capita[403], their OCC will be evaluated by using a similar OCC. However, if there is a significant gap between their HDI or GDP per capita,[394] indicating no similar situation, the method of equal proportion will be used to estimate the OCC for the remaining countries. According to the latest data published by the World Bank,[404] India belongs to the lower middle-income country. In the countries of stranded assets calculated, the Madagascar and North Korea are low-income countries. India's OCC data is 1200 US$/kW, which can be used as a reference suitable for generating OCC for low-income countries. Therefore, the current OCC data for low-income countries is assumed to be 1200 US$/kW.

- The installed capacity scale of coal-fired units (K, unit: kW) comes from Global Energy Monitor.[395]
- According to the estimation by Global Energy Monitor (GEM) for the expected lifetime of coal-fired units (L, unit: years), an average expected lifetime of coal-fired units is set to be 40 years.[395]
- The GEM dataset[395] includes opening dates for each coal-fired unit. The opening dates for each plant are used to determine the remaining operational lifespan when plants are stranded.

These data are all available and are open to download for different countries The data sources of NBV indicators are shown in *Table 111* in the Additional Information Section.

Caveats

- This indicator only considered stranded assets of coal-fired power plants in the power generation sector. The analysis includes only stranded assets generated by existing coal-fired units and does not include planned units.
- To the authors' knowledge, the NBV method used does not include the Weighted Average Cost of Capital (WACC). This is because there is a lack of accurate and reliable data for the WACC for global countries or regions. The NBV method prioritizes the consideration of actual capital expenditures and the remaining plant lifetime, as these two core factors directly impact the valuation of stranded assets, and hence, the decision to omit WACC aligns with practical considerations.
- The capital cost and service life are as two key indicators to estimate stranded assets. Apart from capital cost and service life, other indicators influence the value of stranded assets, such as dynamic changes in electricity prices operations, and maintenance costs, among other factory-level parameters. The present values of these parameters are commonly undisclosed proprietary information, and it is uncertain to forecast their future values, which can't be included in the method for 50+ countries.
- Although there are many different roadmaps to phase down coal-fired power units, which would lead to different results, a lowest-stranded-assets phase-out roadmap is designed to get a lower bound of the scale of stranded assets.

Future form of the indicator

In the future, the health impact of different pathways might be evaluated with relevant available data.

Due to incomplete data collection for gas power plants, this year's calculations for the new indicators only contain coal power plants. Depending on the actual acquisition of subsequent data, gas power plants might be incorporated into the indicator in 2025’s report.

Additional Analysis

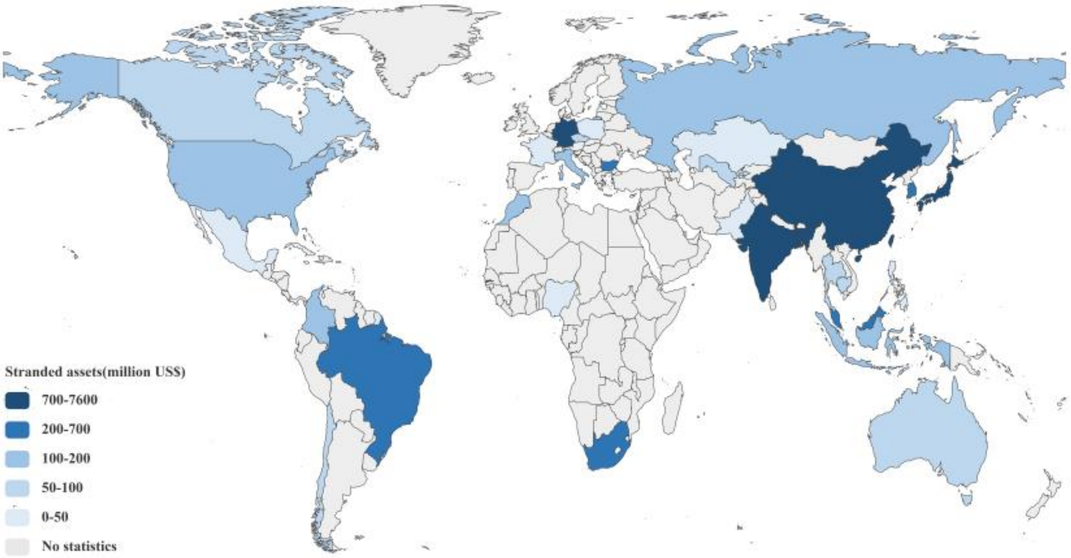

Figure 165: Stranded assets in the global coal-fired power generation sector in 2030.

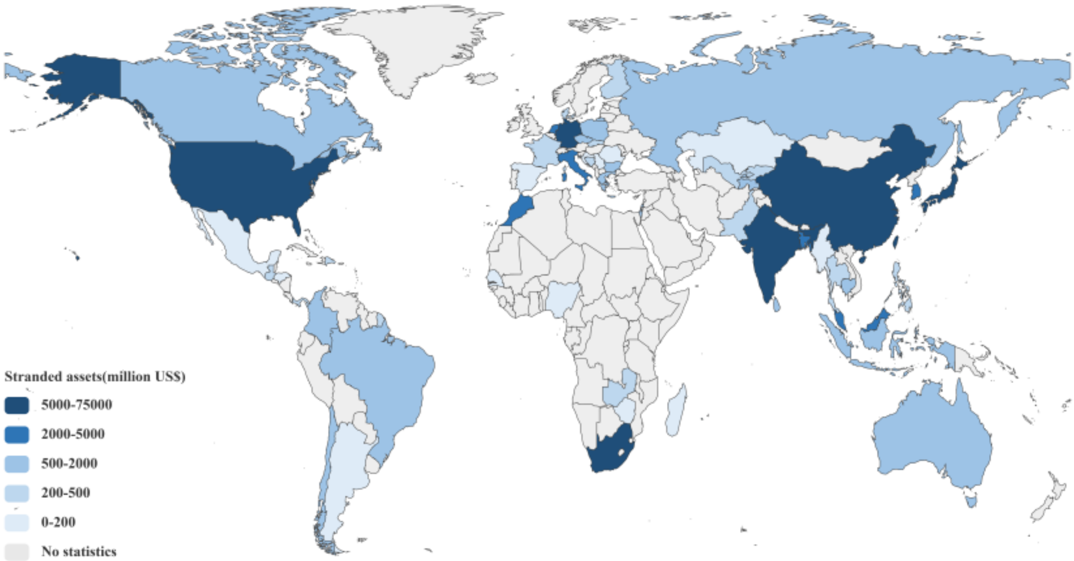

Figure 166: Cumulative stranded assets in the global coal-fired power generation sector, 2025-2034.

Table 109: Human Development Index (HDI) classification and stranded assets globally in 2030.

HDI classification	Stranded assets globally in 2030 (million US$)
Very High	4,199.1
High	9,226.5
Medium	2,181.7
Low	38.6

Table 110: Human Development Index (HDI) classification and Cumulative stranded assets globally in 2025-2034.

HDI classification	Cumulative stranded assets globally in 2025-2034 (million US$)
Very High	52,685.2
High	89,527.9
Medium	21,483.5
Low	831.5

Table 111: The data sources of NBV indicators

Indicators	Data Source	The number of applicable countries
Geographic information	Global Energy Monitor,[395] Open Street Map[396]	54
Overnight cost of capital (*OCC*)	International Energy Agency[400]	54
Installed capacity(*K*)	Global Energy Monitor[395]	54

Firstly, the 1.5°C pathway is selected, which emphasizes sustainable development, technological innovation, and a low-carbon economy. By calculating and comparing the difference between the annual potential carbon dioxide emissions and the annual carbon quota under the 1.5°C pathway, the installed capacity of coal-fired power plants is determined in a given country for a specific year. Then, the NBV method is used to estimate the stranded assets for each coal-fired power unit in the given country, assuming that its coal-fired power units were retired in the calculated year. Lastly, following a phased-out approach based on the lowest-stranded-assets phase-out roadmap, coal-fired power units are progressively retired, with the lowest-stranded assets eliminated first. The total size of the installed capacity scale for the total retired coal-fired units to be phased out in each country is determined by both the annual carbon emissions from each unit and the lowest-stranded-assets phase-out roadmap. Once the total capacity of retired coal-fired power units meets the emission reduction quotas specified for each country, further retirements cease, allowing for the estimation of the total stranded assets generated by the retired coal power stations. e.g., Esperanza G et al. (2019),[405] Pfeiffer A et al. (2018).[406]

The calculation method for potential carbon dioxide emissions related to coal-fired units：
For CO_2 calculation method, see "Estimating carbon dioxide emissions from coal plants" in GEM.[407]
Carbon allowances calculation method:

Firstly, this study is based on the global total CO_2 emissions data for 2019-2100 under the 1.5°C pathway (SSP1-1.9 scenario) in the SSP database CMIP6 Emissions.[397–399] Then the carbon allowances of global countries in 2019–2100 are calculated based on the modeling of the allocation method of fairness principles. The fairness principles employed include: (1) Historical Responsibility (Resp), (2) Capability to pay (Cap), and (3) Equal per capita convergence (Eqpc).[408–411] This approach assists in integrating various circumstances of different countries into the quota distribution, providing a more comprehensive reflection of the fairness in global climate actions. It is the most mainstream method of carbon quota allocation at the country level.

Historical Responsibility (Resp)

Countries with higher historical cumulative national emissions (E_i^{cum}) are allocated a stricter emission reduction target. Firstly, the global reduction quota is estimated and allocated to countries according to their cumulative emission share. Calculation:

$$E_{i,t} = BAU_{i,t} - \left(BAU_{glob,t} - E_{glob,t}\right) * \frac{E_i^{cum}}{E_{glob}^{cum}}$$

Where $E_{i,cum} = \sum_{t=t_0}^{t_e} E_{i,t}$. $E_{i,t}$ are country emission budgets for $i = 1,...,I$ for all I countries in year t, $BAU_{i,t}$ are business-as-usual (BAU) emissions of country i in year t, $BAU_{glob,t}$ is the global BAU emission, $E_{glob,t}$ is the emission budget in year t, and E_{glob}^{cum} is the cumulative global emissions.

The Resp scheme has two operational parameters (OPs): the starting year of historical cumulative emissions (OP1), and dynamic or static timing mechanism (OP2). The starting year of historical cumulative emissions defines when counting the historical cumulative national emissions from. Under the static timing mechanism, the amount of historical cumulative national emission is not updated over time, while under the dynamic timing mechanism, it is updated to the latest time. This study uses the dynamic timing mechanism.

Capability to pay (Cap)

GDP per capita (GPC) represents the ability to pay and set a threshold of reduction to guarantee the development needs of countries below the threshold. Countries above the income threshold (GPC_{thre}) bear a share of emission reduction responsibility proportionate to their ability to pay, while countries below the threshold maintain their BAU emissions.

If $GPC_i \leq GPC_{thre}$,

$$E_{i,t} = BAU_{i,t}$$

If $GPC_i > GPC_{thre}$,

$$E_{i,t} = BAU_{i,t} - \left(BAU_{glob,t} - E_{glob,t}\right) * \frac{GPC_{i,t}}{\sum_{GPC_i > GPC_{thre}} GPC_{i,t}}$$

The Cap scheme has two OPs: GDP conversion method (OP3) and income threshold (OP4). When compare the actual economic scale of each country, there are usually two methods of currency conversion: Purchasing Power Parity (PPP) and Market Exchange Rate (MER). This study lists the GDP conversion standard as an important operability parameter, because different conversion methods will lead to large differences in national economic rankings. The income threshold parameter includes three options: no threshold, \$6,000PPP, or \$6,400PPP.

Equal per capita convergence (Eqpc)

The principle of Per Capita Equality reflects convergence to equal per capita emissions by 2100 (beginning at today's unequal level of emissions), based on all sectors' emissions (sectors in and outside the Emission Trading System). Thus, the calculation of country emission shares in 2100 is simply:

$$s_j = \frac{p_j}{\sum_{j=1}^{j} p_j}$$

where s_j are country emissions shares for $j = 1,...,J$ for all J countries and p_j is population of country j. Finally, the three allowances (Resp, Cap, Eqpc) are weighted, each with a weight of 1/3, to get the total carbon emissions of each country. The results of the carbon allowances for the year 2030 for certain larger countries, including China, India, the USA, and Indonesia, are presented in *Table 112.*

Table 112: The results of the carbon allowances for the year 2030 in China, India, USA, and Indonesia

ISO3	Resp (Mt CO_2)	Cap (Mt CO_2)	Eqpc (Mt CO_2)	Total carbon allowances (Mt CO_2)
CHN	11,081.7	9,876.3	3,964.5	8,307,5
IND	2,659.3	1,866.5	4,069.2	2,865
USA	230.2	2,489.2	954	1,224.5
IDN	689.5	503.3	795	662.6

Considering that the SSP is updated every 5-6 years, this report will regularly reassess and update the carbon allowances based on the latest CMIP6 carbon emissions data. This ensures that they continue to meet the actual needs and changing circumstances under the 1.5°C pathway. Currently this report uses SSP Database-Version 2.0, which was updated in December 2018. Moreover, this indicator's research methodology is open and verifiable, with public resources available for reference in the initial version of kawilliges/EU-Effort-Sharing. This document can be found in a permanent public Github repository linked to Zenodo.[412]

Global Energy Monitor's data has been used by climate researchers at many well-known institutions, including the IPCC, Climate Action Tracker, Greenpeace, the *Lancet*, OECD Environment Directorate, Imperial College London, Harvard University School of Engineering and Applied Sciences, etc. For its accuracy, the dataset with yearbook and officially published data are compared to verify the GEM dataset. For example, the total capacity of coal and gas plants in China in GEM dataset is 1,177GW while the coal-fired generation capacity is 1,297GW in China Power Industry Statistics, the variation of which is less than 10%. Compared with another latest dataset, it is found that the capacity of coal-fired power plants in China is 710GW in GPED,[413] covering only 55% of all capacity. Meanwhile, it is widely recognised in the community that lots of scientific studies are based on this dataset, e.g., Cui et al. (2021),[414] Wang et al. (2021).[415]

The countries for this calculation of global stranded assets include China, Japan, India, Germany, South Africa, United States of America, Netherlands, Republic of Korea, Bangladesh, Malaysia, Morocco, Italy, Brazil, Indonesia, Russian Federation, Greece, Israel, Czechia, Australia, Cambodia, Bulgaria, Canada, Chile, Colombia, Sri Lanka, Poland, Tajikistan, Denmark, Thailand, Kyrgyzstan,

Panama, France, Pakistan, Bosnia and Herzegovina, Philippines, Dominican Republic, Slovakia, Guatemala, Finland, Uzbekistan, Zambia, Mexico, Croatia, Spain, Senegal, Nigeria, Serbia, Myanmar, Argentina, Honduras, Kazakhstan, Madagascar, Romania, Zimbabwe.

Indicator 4.2.4: country preparedness for the transition to net zero

Indicator authors

Dr Denitsa Angelova, Dr Nadia Ameli

Methods

The indicator builds upon the World Bank indicator of Transition Risks - Preparedness of Countries for a Low-Carbon Transition.[416] Factors contributing to resilience include macroeconomic stability and performance, strong institutions, developed financial market development, robust infrastructure and technological advancement, advanced human capital, access to renewable resources and the availability of green finance. Vulnerability factors include reliance on fossil fuel, both as a source of revenue and in energy generation, carbon intensity in key sectors, share of the labour force in highly affected sectors, and income disparity. Data for each variable are sourced from the respective database for all available years. The data are then compiled to investigate patterns of missing values. Observations for individual variables are recovered for as many years as possible. In cases where specific years have missing values, variables are imputed at the country level if the last available observation is reasonably recent. This process results in a cross-sectional dataset, wherein some countries have a full set of observations.

A correlation analysis is performed on the subset of countries with complete observations. Variables displaying high correlation variables are identified, and one of the variables is omitted to avoid double counting. Weights for the selected variables are derived through principal component analysis and the variable weights associated with the first component are extracted. As the resilience and vulnerability factors are designed to represent opposing influences on a country's preparedness, the signs of the principal component weights for resilience and vulnerability indicators typically diverge; for instance, resilience factors tend to have positive weights, while vulnerability factors tend to have negative weights. A second imputation of values is conducted for countries with over 65 percent of observations available, including key indicators on macroeconomic stability, infrastructure, and financial market development. Resilience and vulnerability components are treated separately. Any missing value in resilience-contributing factors is filled using the country average for the remaining resilience factors. Likewise, missing values for vulnerability factors are imputed using the country average for the remaining vulnerability factors.

Variables are normalized within countries by first subtracting the smallest observation for each specific variable j and country i $x_{j,i}$ from the dataset $x_{j,min}$. This difference is then divided by the distance between the largest observation $x_{j,max}$.and the smallest observation $x_{j,min}$ in the dataset for that variable j. The normalized values of the variables are thus obtained:

$$|x_{j,i}| = \frac{x_{j,i} - x_{j,min}}{x_{j,max} - x_{j,min}}$$

It should be noted that the normalized values $|x_{j,i}|$ are non-negative and fall within the range of zero to one. These normalized values for the variables are subsequently multiplied by the weights w_j

obtained through the principal component analysis. The resilience, vulnerability, and preparedness scores are then computed as the sum of the weighted and normalized variables. The preparedness score of country i denoted as pc_i is the sum of the resilience and vulnerability scores, calculated as:

$$pc_i = \sum_{j=1}^{J} \mathrm{w}_j * |x_{j,i}|$$

In the final step, resilience, vulnerability, and preparedness scores are rescaled between zero and one using the outlined procedure to enhance the interpretation of the scores. Before rescaling, the non-normalized resilience indicator tends to be positive due to the predominantly positive factor weights, whereas the non-normalized vulnerability indicator tends to be negative. Following rescaling, both indicators become non-negative and fall within zero and one. They should be interpreted as a normalized distance to the minimum possible resilience and maximum possible vulnerability, respectively. Similarly, a preparedness score of one would indicate the maximum possible preparedness to transition risk. A normalised resilience score of one would indicate the maximum possible resilience, while a normalized vulnerability score of one would indicate the minimum possible vulnerability. To prevent confusion, the normalized vulnerability indicator, which ranges from zero to one, is henceforth referred to as "lack of vulnerability."

Data

The new indicator encompasses twenty-five indicators from multiple data sources. The data on macroeconomic stability, infrastructure, financial market development, technology absorption, and the future orientation of the government are obtained from the World Economic Forum (WEF).[417,418] These WEF indicators themselves are composite in nature:

- macroeconomic stability captures data on inflation and debt dynamics;
- infrastructure encompasses transport and utility infrastructure,
- financial market development covers the depth and stability of the financial system,
- the technology absorption indicator summarizes mobile and internet subscriptions, and
- the future orientation of the government indicator provides insights into policy stability and the government responsiveness to changes, energy efficiency, and renewable energy regulation, and the number of environment-related treaties in force. These indicators contribute to a country's resilience.

The additional data sources include:

- The human capital data are sourced from the Human Development Reports of the United Nations Development Programme, contributing to a country's resilience[419]
- Economic complexity data originate from the Observatory of Economic Complexity and MIT Media Lab, contributing to a country's resilience[420]
- Economic performance data, adjusted net savings, net trade, monetary strength information, and fuel exports are obtained from the World Development Indicators of the World Bank. The first three indicators contribute to a country's resilience, while the latter two contribute to country's vulnerability[421]
- Institutions data originate from the Worldwide Governance Indicators Project of the World Bank, contributing to a country's resilience[422]
- The breakeven oil price is from Rystad, contributing to a country's resilience[423]

- Expected fossil fuel resource rents and the share of wealth from renewables to total wealth originate from the Changing Wealth of Nations Database of the World Bank. The first indicator contributes to a country's vulnerability, while the second contributes to a country's resilience[424]
- Availability of finance for the energy transition is sourced from Bloomberg, contributing to a country's resilience[425]
- Committed power sector emissions over power generation are computed by the World Bank based on information from Platts and the International Energy Agency, contributing to a country's vulnerability[426,427]
- The GINI coefficient originates from the World Inequality Database, contributing to a country's vulnerability[427]
- Current carbon intensity of manufacturing industries, transportation, and construction as well as data on labour employed in highly vulnerable sectors, originate from the Global Trade Analysis Project (GTAP), contributing to a country's vulnerability[428]

All databases have global coverage and provide yearly estimates, with updates occurring annually, except for Bloomberg, Rystad, and GTAP. Bloomberg undergoes daily updates, Rystad is continuously updated and GTAP receives updates every few years.

Table *113* lists the 25 indicators used to construct the overall indicator and names the data sources. It also specifies the dimension that the indicator captures and justifies its inclusion.

Table 114: The 25 indicators used to construct indicator 4.2.8, along with dimension, data source and justification for inclusion.

Number	Indicator	Dimension	Data Source	Justification
1	Macroeconomic stability	Resilience	World Economic Forum	The general economic stability with respect to inflation and debt developments contributes to the overall resilience of a country.
2	Infrastructure	Resilience	World Economic Forum	A more developed transport and utility system eases the low-carbon transition.
3	Technology absorption	Resilience	World Economic Forum	A more developed infrastructure with respect to the Internet and mobile eases the low-carbon transition.
4	Financial market development	Resilience	World Economic Forum	Like the general macroeconomic stability, the general stability of the financial system contributes to the overall resilience of a country.
5	Availability of finance, % of GDP	Resilience	Bloomberg	An abundance of capital for green transition increases the number of projects that can be implemented.
6	Future orientation of the government	Resilience	World Economic Forum	The stability of the political system and the consistency of the energy and environmental regulations provide the necessary stability for the low-carbon transition.
7	Economic performance, GNI per capita	Resilience	World Development Indicators of the World Bank	The monetary wealth of a nation contributes to the overall resilience of a country.
8	Adjusted net savings, % of GNI	Resilience	World Development Indicators of the World Bank	The non-monetary wealth of a nation also contributes to the overall resilience of a country. The adjusted net savings accounts for these aspects by correcting the net national savings and education expenditure by energy depletion, mineral depletion, net forest depletion, and carbon dioxide and particulate emissions damage.
9	Economic complexity	Resilience	Observatory of Economic Complexity and MIT Media Lab	The more complex the economy, the easier it is to adapt to generating income in an alternative way should the necessity arise.
10	Human capital	Resilience	United Nations Development Programme	The more educated the population, the easier it is to adapt to generating income in an alternative way should the necessity arise.

11	Institutions: Voice and accountability	Resilience	Worldwide Governance Indicators Project of the World Bank	Strong institutions create the conditions under which the low-carbon transition is possible. The freedom of the press and the accountability of the government to the public contribute towards the implementation of climate and environmental goals.
12	Institutions: Absence of violence	Resilience	Worldwide Governance Indicators Project of the World Bank	Strong institutions create the conditions under which the low-carbon transition is possible. The lack of terrorism and political violence contributes towards an atmosphere, where climate and environmental goals could be a priority.
13	Institutions: Governmental effectiveness	Resilience	Worldwide Governance Indicators Project of the World Bank	Strong institutions create the conditions under which the low-carbon transition is possible. The general governmental effectiveness safeguards the implementation of climate and energy policies.
14	Net trade, % of GDP	Resilience	World Development Indicators of the World Bank	The net trade in goods is a proxy for the comparative advantages of a country such as a skilled workforce or natural resources, which increases resilience.
15	Breakeven oil price	Resilience	Rystad	The lower extraction costs of fossil fuels increase the resilience of the country in comparison to a country with higher extraction costs.
16	Renewable assets as a % of total wealth	Resilience	Changing Wealth of Nations Database of the World Bank	The share of renewable assets as a fraction of total wealth increases the resilience of the country as it represents the theoretical ability to benefit from the transition.
17	Fuel exports as a % of exports	Vulnerabili ty	World Development Indicators of the World Bank	The higher the percentage of fuel exports to all merchandisable exports, the more vulnerable a country is to the international market fluctuations that come with transition risks.
18	Expected fossil fuel resource rents	Vulnerabili ty	Changing Wealth of Nations Database of the World Bank	The more fossil fuel resources are available, the larger the urge to use them.
19	Committed power sector emissions	Vulnerabili ty	World Bank calculations based on Platts and the International Energy Agency	The more developed the infrastructure for energy generation from fossil fuels, the longer it would take to adjust to alternative energy sources. The risk of stranded assets increases as well.
20	Currency weakness	Vulnerabili ty	World Development Indicators of the World Bank	A weaker currency makes the exports of a country more attractive in general, which makes it more vulnerable to the international market fluctuations that come with transition risks

21	Carbon intensity of manufacturing	Vulnerabili ty	Global Trade Analysis Project (GTAP)	A higher carbon intensity of the production directly increases the vulnerability to transition climate risks.
22	Carbon intensity of transportation	Vulnerabili ty	Global Trade Analysis Project (GTAP)	A higher carbon intensity of transportation directly increases the vulnerability to transition climate risks.
23	Carbon intensity of the production of non-metallic minerals	Vulnerabili ty	Global Trade Analysis Project (GTAP)	A higher carbon intensity of the production directly increases the vulnerability to transition climate risks.
24	Labor in highly exposed sectors, % of all labor force	Vulnerabili ty	Global Trade Analysis Project (GTAP)	A larger share of the labor force in the highly vulnerable sectors makes a country more vulnerable.
25	GINI coefficient	Vulnerabili ty	World Inequality Database	A more unequal distribution of income might indicate that a country is less prepared for a just transition.

Caveats

The World Bank estimated committed emissions from power generation using data from the Platts database and information provided by the International Energy Agency. While these databases are among the most comprehensive and consistent sources available regarding the energy production infrastructure of a country, there are still significant limitations that need to be overcome to accurately assess committed emissions. The reliability of the powerplant database is difficult to verify since crucial information such as the powerplant commission year, maximum capacity, and current capacity utilization, which would enable verification, may not be publicly accessible. Additionally, developing countries require country-specific estimates of the carbon intensity of power generation for different technologies as well as information on the technological alternatives for decarbonization across different industries.

Additionally, the indicator in its current form does not reflect opportunities arising from the low-carbon transition, particularly those emerging from access to non-renewable resources vital to the transition, such as critical minerals. It also overlooks different aspects of access to renewable energy sources such as security, reliability, and flexibility. This omission is primarily attributed to the lack of reliable data. The integration of these factors, which influence the potential benefits of the low-carbon transition, will be considered in future iterations of the indicator as more comprehensive and consistent data products become accessible.

Future form of the indicator

Better and more consistent datasets appear over time, which would allow for a more accurate estimation of the committed emissions from power generation and of the theoretical ability to benefit from the low-carbon transition in future versions of the indicator. The next version of the indicator would also recreate the scores for previous years.

Additional analysis

The lack of vulnerability, resilience, and preparedness scores are reported in Table *115*. Since the resilience, lack of vulnerability, and preparedness scores are rescaled between zero and one in the final step, the resilience and lack of vulnerability scores do not directly contribute to the preparedness score in a linear fashion.

Table 116: Lack of vulnerability, resilience, and preparedness scores. Source: Own calculation.

ISO3	Country	Lack of vulnerability	Resilience	Preparedness
AGO	Angola	0.478	0.187	0.171
ALB	Albania	0.757	0.478	0.491
ARE	United Arab Emirates	0.680	0.806	0.787
ARG	Argentina	0.728	0.469	0.478
AR	Armenia	0.836	0.478	0.503
AUS	Australia	0.772	0.824	0.819
AUT	Austria	0.958	0.861	0.885
AZE	Azerbaijan	0.714	0.493	0.497
BDI	Burundi	0.620	0.128	0.138
BEL	Belgium	0.930	0.827	0.847
BEN	Benin	0.000	0.313	0.211

BFA	Burkina Faso	0.399	0.196	0.166
BGD	Bangladesh	0.679	0.361	0.368
BGR	Bulgaria	0.785	0.623	0.632
BHR	Bahrain	0.710	0.633	0.629
BIH	Bosnia and Herzegovina	0.755	0.444	0.458
BOL	Bolivia	0.586	0.373	0.363
BRA	Brazil	0.729	0.493	0.500
BRB	Barbados	0.669	0.585	0.577
BRN	Brunei Darussalam	0.500	0.662	0.622
BW	Botswana	0.515	0.566	0.534
CAN	Canada	0.826	0.853	0.856
CHE	Switzerland	0.957	0.984	1.000
CHL	Chile	0.685	0.692	0.680
CHN	China	0.690	0.662	0.653
CIV	Cote d'Ivoire	0.611	0.329	0.326
CMR	Cameroon	0.716	0.240	0.259
COD	Democratic Republic of the Congo	0.661	0.053	0.073
COL	Colombia	0.678	0.495	0.494
CPV	Cabo Verde	0.290	0.378	0.319
CRI	Costa Rica	0.792	0.579	0.591
CYP	Cyprus	0.670	0.601	0.592
CZE	Czechia	0.910	0.777	0.797
DEU	Germany	0.882	0.902	0.911
DNK	Denmark	0.928	0.940	0.954
DO	Dominican Republic	0.744	0.506	0.515
DZA	Algeria	0.268	0.385	0.323
ECU	Ecuador	0.615	0.473	0.462
EGY	Egypt	0.715	0.381	0.392
ESP	Spain	0.839	0.784	0.793
EST	Estonia	0.872	0.773	0.787
ETH	Ethiopia	0.716	0.194	0.216
FIN	Finland	0.950	0.916	0.935
FRA	France	0.928	0.845	0.865
GAB	Gabon	0.543	0.378	0.361
GBR	United Kingdom	0.890	0.871	0.883
GEO	Georgia	0.659	0.515	0.510
GHA	Ghana	0.624	0.398	0.393
GIN	Guinea	0.581	0.203	0.202
GM	Gambia	0.707	0.307	0.321
GRC	Greece	0.633	0.598	0.583
GTM	Guatemala	0.404	0.356	0.318
HKG	Hong Kong SAR (China)	0.680	0.831	0.811
HND	Honduras	0.566	0.324	0.314
HRV	Croatia	0.895	0.653	0.678
HTI	Haiti	0.507	0.129	0.120
HUN	Hungary	0.907	0.703	0.727
IDN	Indonesia	0.568	0.530	0.509
IND	India	0.541	0.520	0.495

IRL	Ireland	0.846	0.879	0.884
IRN	Islamic Republic of Iran	0.122	0.358	0.273
ISL	Iceland	0.978	0.859	0.886
ISR	Israel	0.725	0.689	0.684
ITA	Italy	0.830	0.732	0.742
JAM	Jamaica	0.514	0.493	0.465
JOR	Jordan	0.634	0.478	0.470
JPN	Japan	0.847	0.893	0.896
KAZ	Kazakhstan	0.620	0.521	0.508
KEN	Kenya	0.645	0.350	0.351
KGZ	Kyrgyzstan	0.545	0.376	0.359
KH	Cambodia	0.759	0.333	0.354
KOR	Republic of Korea	0.836	0.891	0.892
KW	Kuwait	0.451	0.690	0.640
LAO	Lao People's Democratic Republic	0.426	0.356	0.321
LBN	Lebanon	0.492	0.387	0.362
LBR	Liberia	0.704	0.158	0.180
LKA	Sri Lanka	0.726	0.449	0.458
LSO	Lesotho	0.608	0.297	0.296
LTU	Lithuania	0.824	0.736	0.745
LUX	Luxembourg	0.813	1.000	0.992
LVA	Latvia	0.823	0.693	0.704
MA	Morocco	0.688	0.472	0.473
MD	Republic of Moldova	0.679	0.483	0.482
MD	Madagascar	0.512	0.182	0.171
MEX	Mexico	0.693	0.566	0.563
MK	North Macedonia	0.773	0.494	0.508
MLI	Mali	0.793	0.133	0.171
MLT	Malta	0.792	0.795	0.796
MNE	Montenegro	0.776	0.523	0.536
MN	Mongolia	0.393	0.386	0.344
MOZ	Mozambique	0.631	0.120	0.132
MRT	Mauritania	0.694	0.203	0.220
MUS	Mauritius	0.622	0.703	0.681
MWI	Malawi	0.641	0.218	0.226
MYS	Malaysia	0.616	0.718	0.694
NA	Namibia	0.663	0.478	0.475
NGA	Nigeria	0.681	0.221	0.235
NIC	Nicaragua	0.588	0.300	0.295
NLD	Netherlands	0.866	0.929	0.933
NOR	Norway	0.977	0.930	0.953
NPL	Nepal	0.462	0.329	0.301
NZL	New Zealand	0.857	0.750	0.764
OM	Oman	0.395	0.583	0.530
PAK	Pakistan	0.641	0.293	0.297
PAN	Panama	0.366	0.587	0.529
PER	Peru	0.710	0.484	0.488
PHL	Philippines	0.645	0.494	0.487

POL	Poland	0.844	0.693	0.707
PRT	Portugal	0.868	0.744	0.759
PRY	Paraguay	0.652	0.399	0.399
QAT	Qatar	0.587	0.838	0.803
ROU	Romania	0.862	0.640	0.660
RUS	Russian Federation	0.718	0.560	0.562
RW	Rwanda	0.626	0.376	0.373
SAU	Saudi Arabia	0.135	0.718	0.615
SEN	Senegal	0.435	0.350	0.317
SGP	Singapore	0.715	0.968	0.946
SLE	Sierra Leone	0.707	0.177	0.198
SLV	El Salvador	0.742	0.385	0.400
SRB	Serbia	0.799	0.539	0.554
SVK	Slovakia	0.997	0.713	0.751
SVN	Slovenia	0.963	0.807	0.834
SWE	Sweden	1.000	0.932	0.958
SWZ	Eswatini	0.592	0.341	0.335
SYC	Seychelles	0.776	0.559	0.570
TCD	Chad	0.601	0.070	0.080
THA	Thailand	0.618	0.583	0.567
TJK	Tajikistan	0.714	0.349	0.362
TTO	Trinidad and Tobago	0.596	0.622	0.600
TUN	Tunisia	0.585	0.481	0.465
TUR	Turkey	0.735	0.510	0.517
TZA	United Republic of Tanzania	0.415	0.293	0.260
UGA	Uganda	0.592	0.274	0.271
UKR	Ukraine	0.695	0.428	0.433
URY	Uruguay	0.907	0.652	0.679
USA	United States of America	0.746	0.865	0.854
VEN	Bolivarian Republic of Venezuela	0.683	0.213	0.229
VN	Vietnam	0.409	0.502	0.456
YEM	Yemen	0.516	0.000	0.000
ZAF	South Africa	0.366	0.561	0.504
ZMB	Zambia	0.659	0.287	0.295
ZWE	Zimbabwe	0.475	0.241	0.220

Countries with a higher Human Development Index (HDI) tend to score higher on preparedness as illustrated in figure 167. The average preparedness score of countries with a very high HDI is 0.74, while the average score of countries with a high HDI is 0.47. The average score of countries with a medium HDI is 0.35, while the average score of countries with a low HDI is 0.2. Figure 169 shows that there is more variation in the preparedness scores of countries with a very high HDI than in the scores for any of the other categories: the standard deviation of preparedness scores in the very high HDI countries is 0.14, while the standard deviations of the preparedness scores in the other categories range between 0.08 and 0.09.

Countries with a higher HDI tend to on average score higher on the resilience scale as illustrated in figure 168. The average resilience score of a country with a very high HDI is 0.74, while the average score of a country with a high HDI is 0.48. The average score of a country with a medium HDI is 0.35,

while the average resilience score of a country with a low HDI is 0.20. There is more variation in resilience scores among countries with a very high HDI than in any of the other HDI categories: the standard deviation of resilience scores is 0.14 among countries with a very high HDI, while it is 0.07 for countries with a high HDI, 0.093 for countries with a medium HDI and 0.096 for countries with a low HDI.

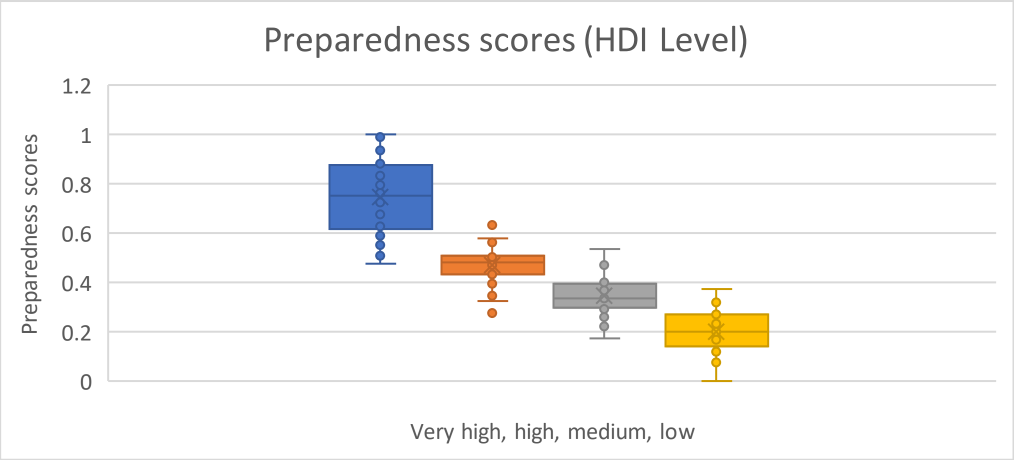

Figure 169: Distribution of preparedness scores across HDI categories: very high HDI, high HDI, medium HDI and low HDI (from left to right).

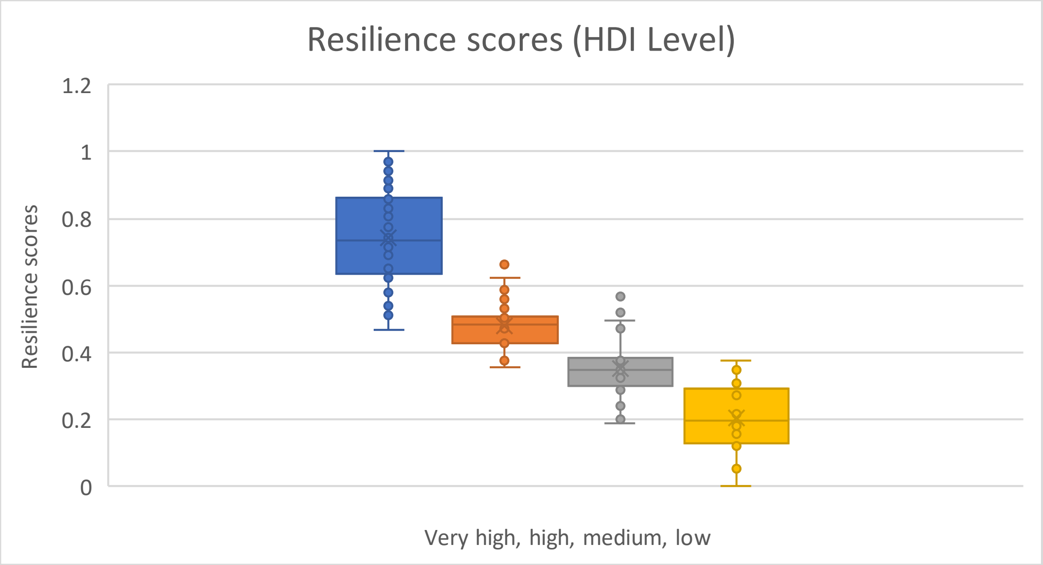

Figure 170: Distribution of resilience scores across HDI categories: very high HDI, high HDI, medium HDI, and low HDI (from left to right).

Countries with a higher HDI tend to on average score lower on vulnerability as illustrated in figure 171. The average vulnerability score among countries with a very high HDI is 0.23, while the average score among countries with a high HDI is 0.38, the average vulnerability score among countries with a medium HDI is 0.41, and the average vulnerability score of the countries with a low HDI is 0.42. The standard deviations of vulnerability scores in the different HDI categories range from 0.11 to 0.16. Three exceptionally vulnerable countries can be detected in Figure 172: Saudi Arabia in the group of countries with a very high HDI, the Islamic Republic of Iran in the group of countries with a high HDI, and Benin in the group of countries with a low HDI. These three countries exemplify the tendency of heavy oil exporters with highly carbon-intensive energy production, manufacturing, transportation, and construction, a large share of the labour force employed in these sectors, and a high social inequality to be more vulnerable to transition climate risk.

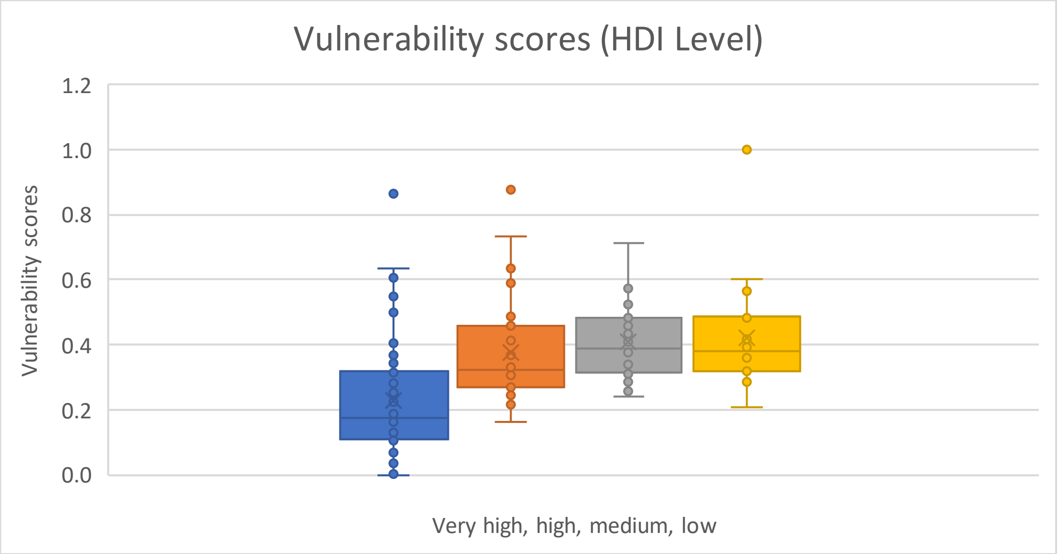

Figure 172: Distribution of vulnerability scores across HDI categories: very high HDI, high HDI, medium HDI and low HDI (from left to right).

Figure 173 displays the lack of vulnerability score of a country on the horizontal axis and the corresponding resilience score on the vertical axis. It reveals a positive correlation between resilience and lack of vulnerability scores. In other words, the more resilient countries are to transition risks, the less vulnerable they tend to be. This observation seems to be confirmed when examining the scores across different regions as they are defined by the World Health Organization (WHO). The resilience and lack of vulnerability scores for Europe are displayed in figure 174, the scores for South-East Asia and the Western Pacific are displayed in figure 175 and the scores for the Americas are displayed in figure 176. All three figures illustrate the positive relationship between resilience and lack of vulnerability. This is to some extent true for the scores displayed in figure 177, which illustrate the results for Eastern Mediterranean. It should be noted that this group of countries includes some of the most extreme observations in the dataset, namely the cases of Saudi Arabia, the Islamic Republic of Iran, and Yemen. Figure 178 displays the results for Africa and illustrates the tendency towards lower resilience in the Global South. Many African countries score only moderately high on vulnerability to transition climate risk.

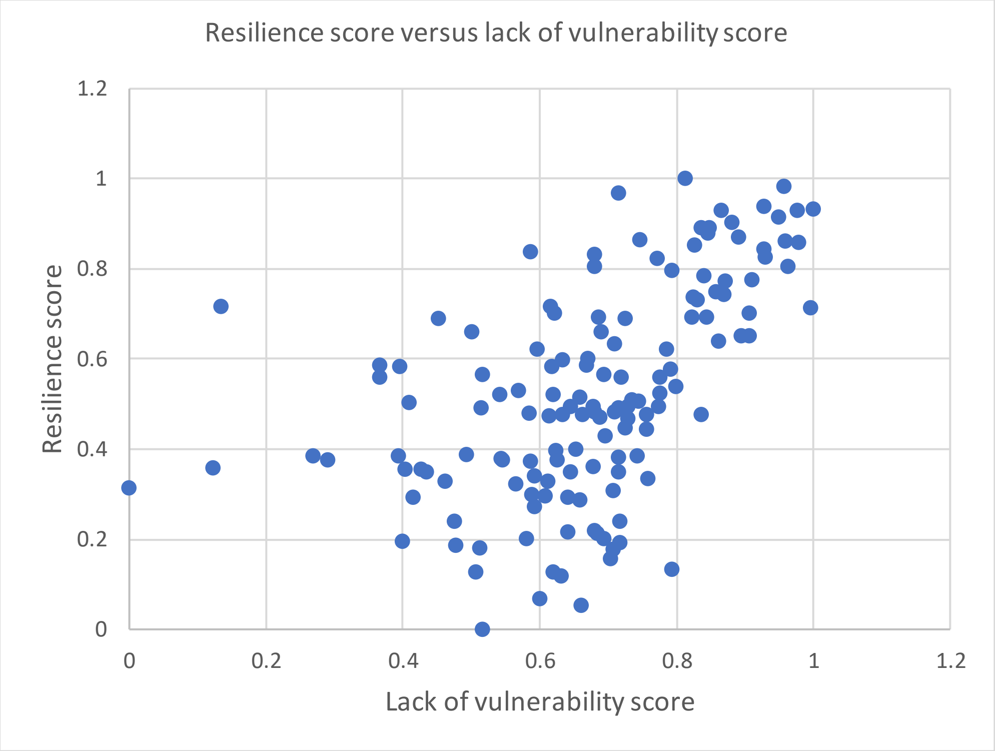

Figure 179: Resilience scores versus lack of vulnerability scores. Country names have been omitted to increase readability.

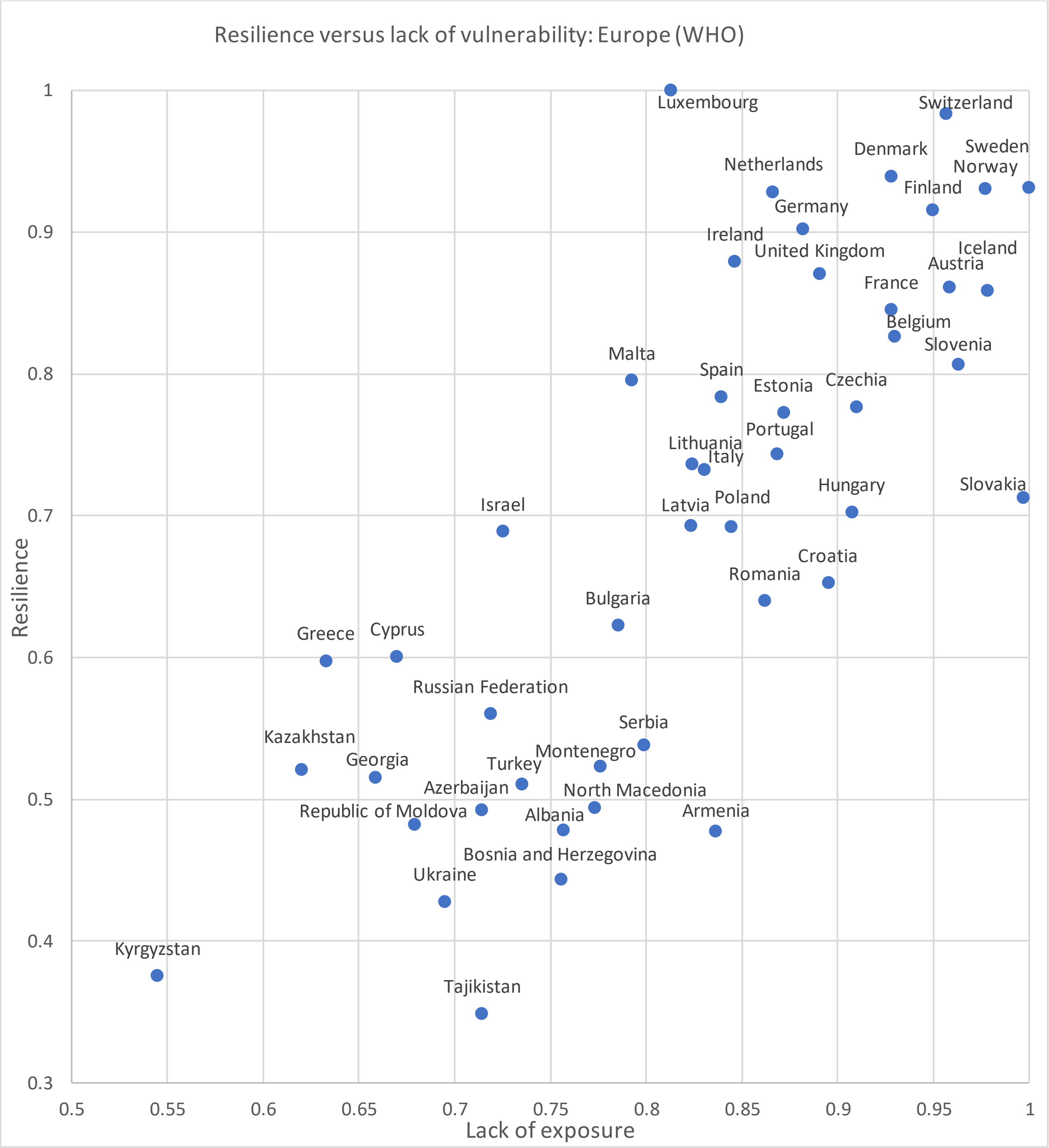

Figure 180: Resilience versus lack of vulnerability scores for Europe (WHO regional classification).

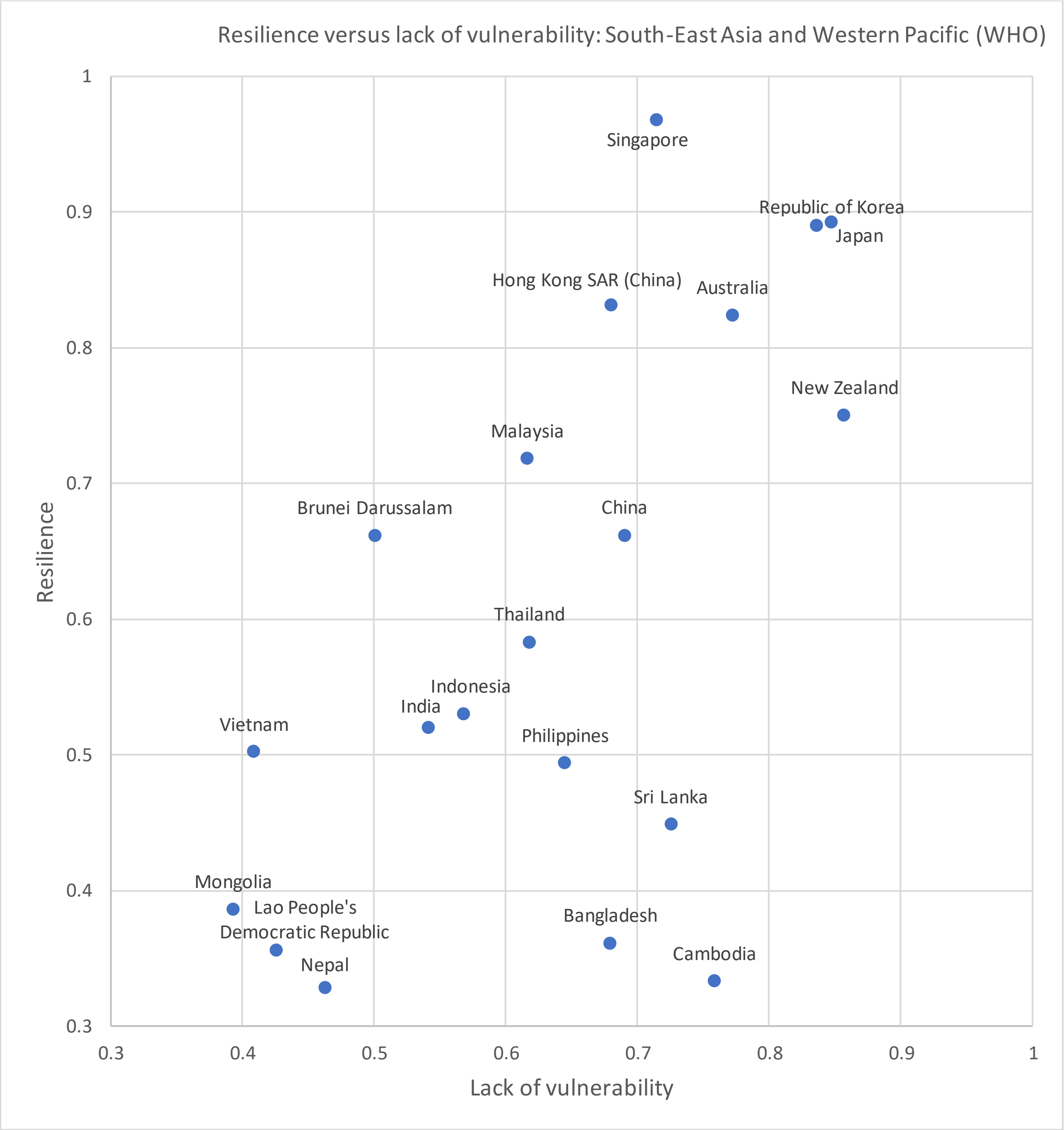

Figure 181: Resilience versus lack of vulnerability scores for South-East Asia and the Western Pacific (WHO regional classification).

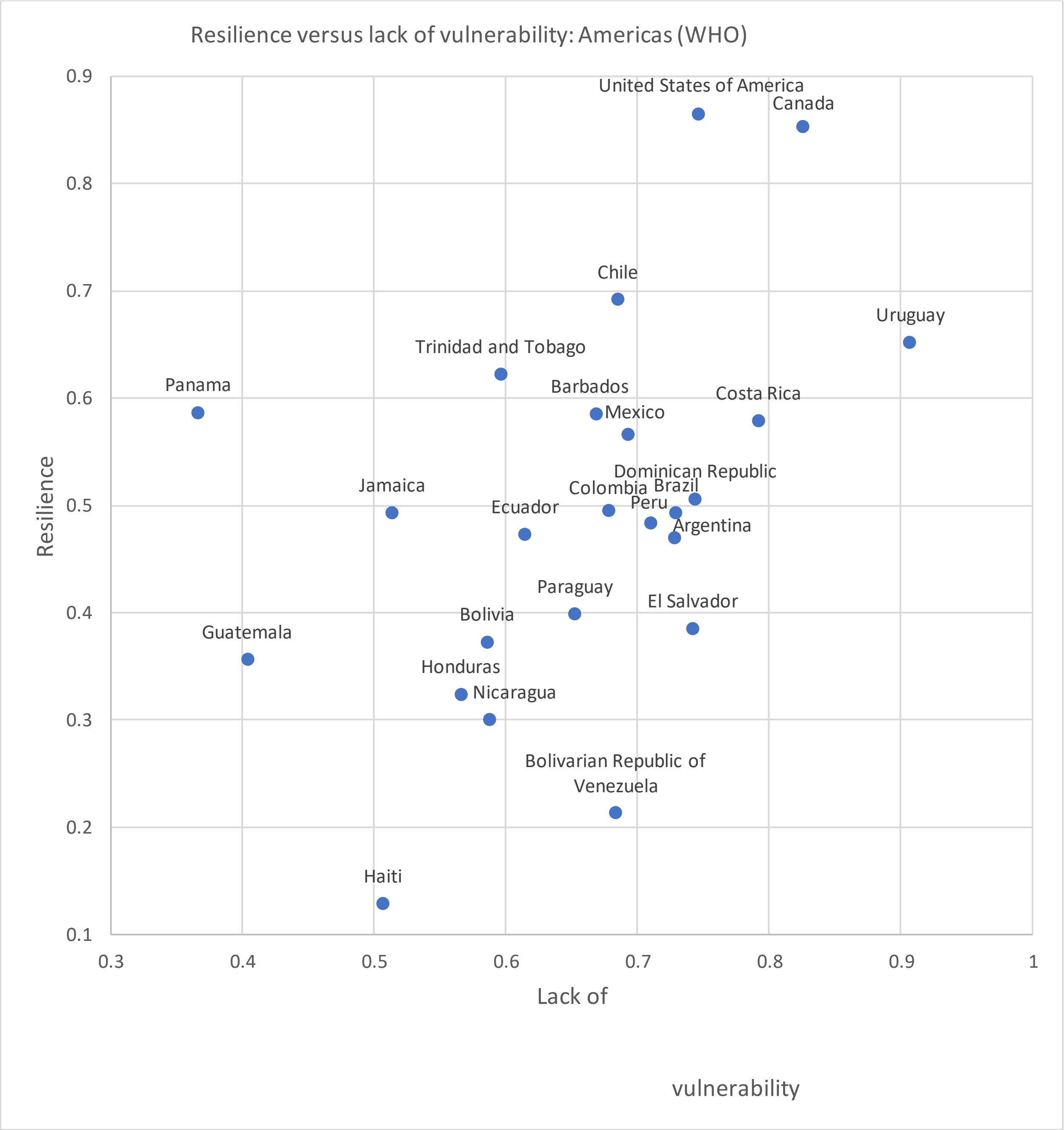

Figure 182: Resilience versus lack of vulnerability scores for the Americas (WHO regional classification).

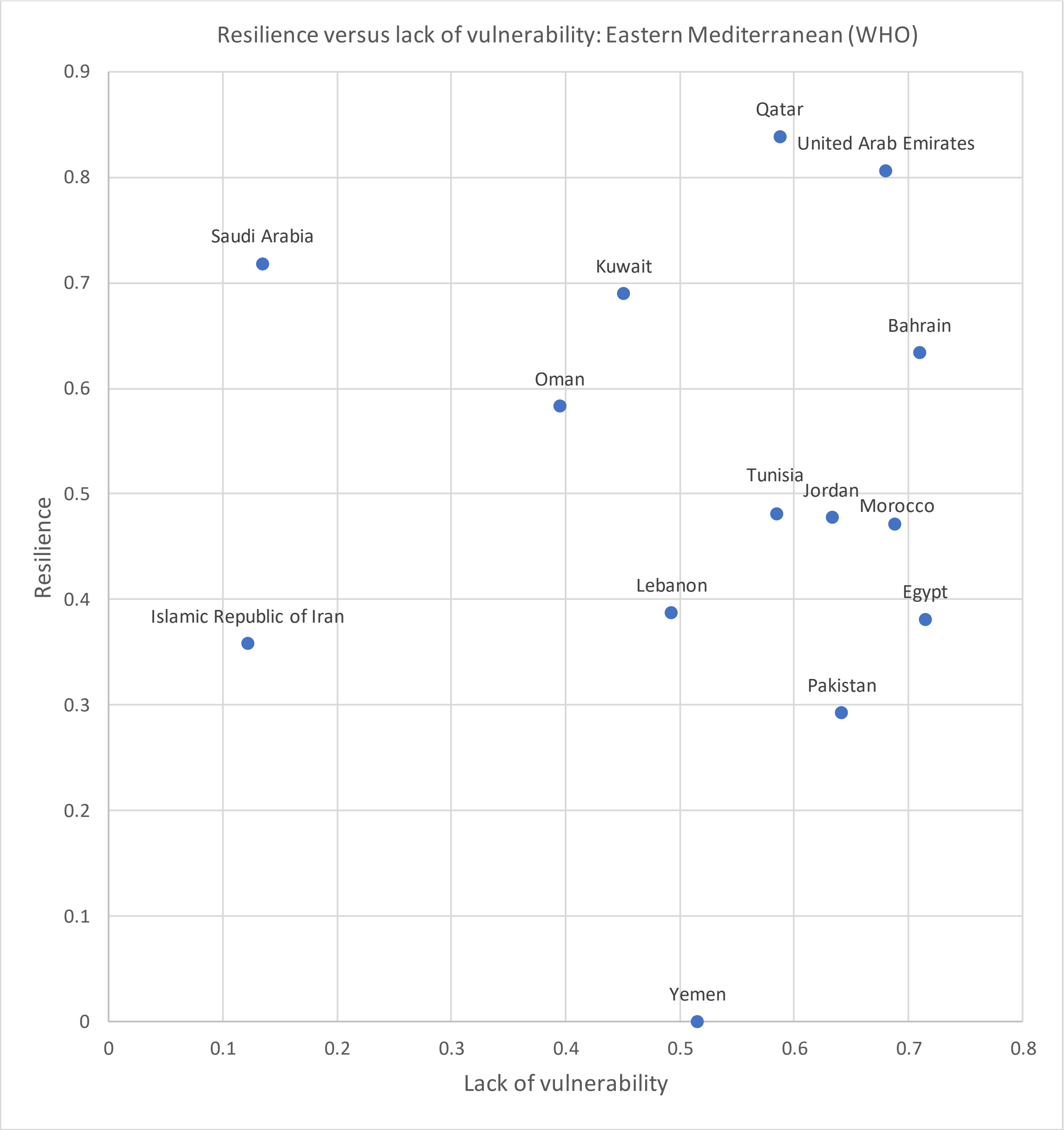

Figure 183: Resilience versus lack of vulnerability scores for the Eastern Mediterranean (WHO regional classification).

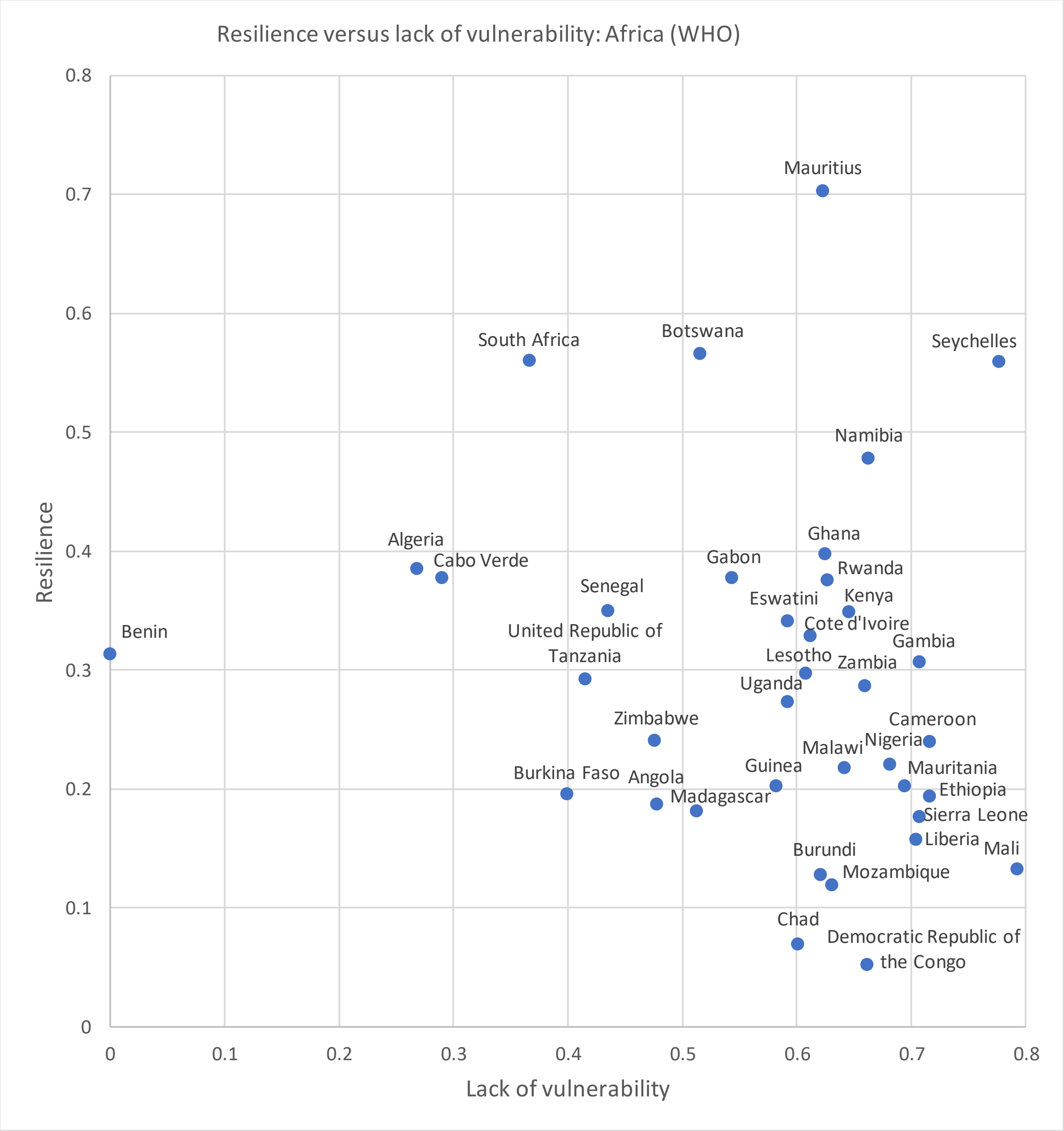

Figure 184: Resilience versus lack of vulnerability scores for Africa (WHO regional classification).

Indicator 4.2.5: production-based and consumption-based attribution of CO_2 and $PM_{2.5}$ emissions

Indicator authors

Dr Kehan He, Prof Zhifu Mi, Dr Fabian Wagner

Methods

Environmentally Extended Multi-Regional Input-Output Analysis

There are two approaches to measure emissions: production-based (sometimes referred to as territorial-based) accounting and consumption-based accounting. Production-based emissions occur within the geographical territory of a nation, while consumption-based emissions encompass the emissions from the nation's domestic final consumption, as well as those caused by the production of its imports. Since both CO_2 emissions via climate change, and air pollution directly, are detrimental to human health, an understanding of the responsibilities of emissions across borders is crucial in the globalised world. This indicator estimates $PM_{2.5}$ and CO_2 emissions embodied in international trade, and then calculates national $PM_{2.5}$ and CO_2 emissions from the consumption perspective. Thus, the responsibility of these emissions and the associated environmental and human health consequences can be distributed for international environmental policy formulation.

Environmentally extended multi-regional input-output (EEMRIO) analysis is used in the calculation of consumption-based emissions.[429] The EEMRIO analysis can reflect production and consumption structures and interdependencies between economic sectors across regions. The relationships between final use and emissions are estimated via Leontief inverse matrix, which is expressed as follows in equation (1):

$$\mathrm{C} = \mathrm{E} \cdot \mathrm{L} \cdot \mathrm{F} = \mathrm{E} \cdot (\mathrm{I} - \mathrm{A})^{-1} \cdot \mathrm{F} \qquad (1)$$

C is the total consumption-based emissions, CO_2 or $PM_{2.5}$ emissions in this case. It is mapped directly to emissions inventories. *E* is the row vector of the production-based emission intensity defined as the emissions per unit of output. *F* is the vector of final demand. and *L* is the Leontief inverse matrix calculated by $(I\text{-}A)^{-1}$, where *I* is the identity matrix, and *A* is the technical coefficient matrix describing the inter-sectoral and inter-regional flows per unit of output.

Consumption-based accounting encompasses emissions from domestic final consumption and those caused by the production of its imports, while production-based accounting measures emissions which take place within national territory. The above relationship can also be expressed as follows:

$$\mathrm{C_{CBA}} = \mathrm{C_{PBA}} - \mathrm{C_{exp}} + \mathrm{C_{imp}} \qquad (2)$$

where C_{CBA} is the consumption-based emissions, C_{imp} is the emissions embodied in imports, C_{PBA} is the production-based emissions, and C_{exp} is the emissions embodied in exports.

Emission Inventory Mapping with GAINS

To construct the production-based $PM_{2.5}$ emission inventory with the GAINS model, the workflow illustrated in figure 185 is followed. First, an intermediary aggregation level to which emissions from the GAINS source categories are aggregated is defined. In a second step these aggregated or grouped emissions are distributed among the relevant MRIO sectors according to a specific rule. This process is repeated until the emissions from all relevant GAINS source categories have been mapped to the relevant MRIO sectors.

In most cases GAINS sectors are used. However, in a few cases the relevant source categories are sector-fuel combinations in the GAINS system: for example, in the power plant sectors, coal-, oil-, gas-, and biomass-fired plants are distinguished [and combustion free generation] so as to be able to map directly to the corresponding MRIO sectors.

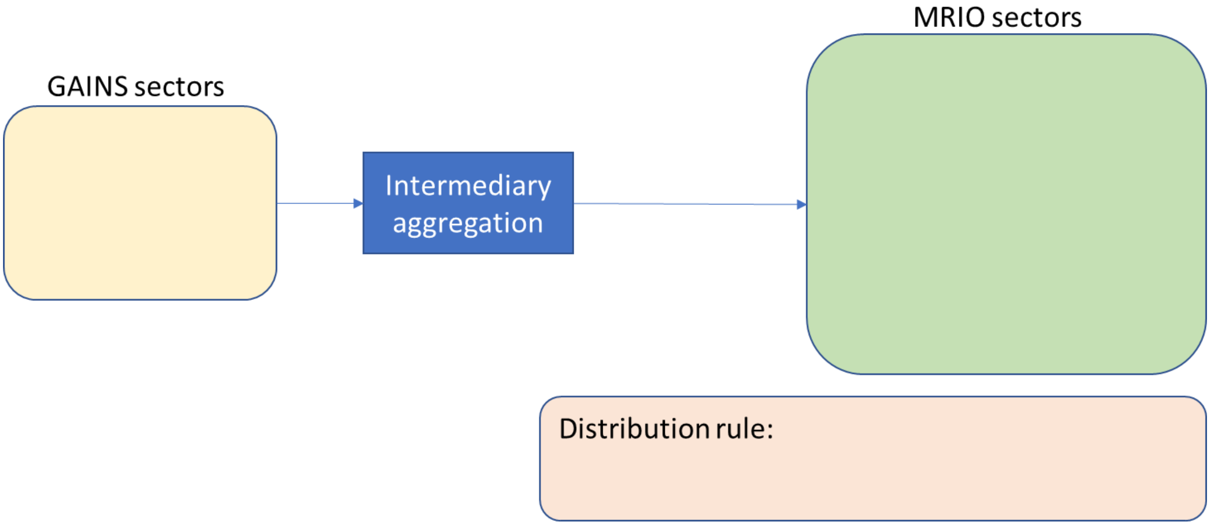

Figure 186: Generic approach for mapping the GAINS sectoral emissions to MRIO sectors.

In practice, the GAINS source categories are clustered into three groups, so that there are three rounds of mappings. These groupings correspond to energy-related emissions (except trucking, see below), process-related emissions, and trucking-related emissions. In a final step, for each MRIO sector the contributions from the three rounds of mappings are summed so that a total emission can be associated with each MRIO sector. In all calculations determining the relative energy share of an MRIO sector in the total energy, the use of electricity is ignored, since the emissions from electricity production are accounted for elsewhere.

On the GAINS side, trucking is related to the sectors TRA_RD_HDT and TRA_RD_LD4T and the fuel-related activities, such as diesel, gasoline, LPG etc, as well as km-related emissions such as abrasion, tyres, and braking. On the MRIO side, diesel consumption from road transport by MRIO sector is used to determine the share of each sector in the total. In some countries significant amounts of diesel is also used by cars, a fact that is neglected here. Figure 187 illustrates the mapping process for trucking-related emissions between GAINS and the MRIO sectors.

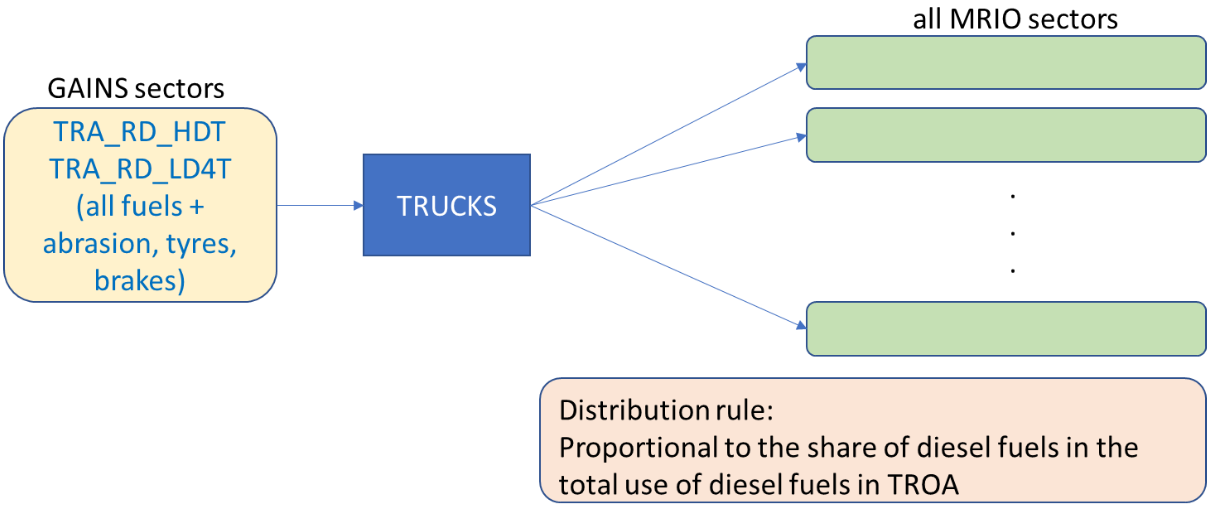

Figure 188: Mapping of trucking-related emissions

The trucking-related emissions in region *r* for MRIO sector *m* are thus calculated as:

$$\mathrm{Em}_r(m,t) = \mathrm{Em}_r(\mathrm{TRUCKS}) \cdot \mathrm{sh}_r(m,\mathrm{diesel}) \tag{3}$$

where

$$\mathrm{sh}_r(m,\mathrm{diesel}) = \frac{\mathrm{TROA}_r(m,\mathrm{diesel})}{\sum_{m'} \mathrm{TROA}_r(m',\mathrm{diesel})} \tag{4}$$

is the share of sector m in the road transport related diesel consumption in region r, and $\mathrm{Em}_r(\mathrm{TRUCKS})$ are the total trucking related emissions in region r as calculated by GAINS.

Once the trucking-related emissions and energy use has been separated out what is relevant for distributing the remaining energy (but not trucking-related emissions) is generally the total final energy consumption minus the diesel consumption in TROA. Thus, non-trucking related final energy consumption excluding electricity is referred to as the relevant final energy consumption in each MRIO sector that is used to determine the shares for distributing energy-related emissions into MRIO sectors.

In the mapping of energy-related emissions, intermediary clusters for energy-related emissions are defined as follow:

Table 117: Aggregated energy-related sectors, their description and coverage in terms of GAINS sectors as well as MRIO clusters.

Label	Description	GAINS sector coverage	MRIO clusters
ELE_COAL	Coal-fired power plants	All power plants combusting coal or solid biomass	coal_electricity
ELE_OIL	Oil-fired power plants	All power plants combusting heavy fuel oil or diesel	oil_electricity
ELE_GAS	Gas-fired power plants	All power plants combusting natural gas	gas_electricity
AGR_MACH	Agricultural machinery	TRA_OT_AGR, DOM_OTH	cultivation + livestock_farming + items_dom_oth
IND_IS	Iron and steel industry	IN_OC_ISTE	manuf_is
IND_NFME	Non-ferrous metals	IN_OC_NFME	manuf_nfme
IND_NMMI	Non-metallic minerals	IN_OC_NMMI	manuf_bricks + manuf_cem + manuf_nmmi
IND_CHEM	Chemical industries	IN_BO_CHEM, IN_OC_CHEM	manuf_chem + manuf_fert + manuf_chem_nec
IND_CON	Conversion industries, incl. refineries	IN_BO_CON, CON_COMB	ind_conversion
PPAPER	Pulp and paper	IN_BO_PAP, IN_OC_PAP	manuf_paper
OTH_IND	Other industries	All IN_XX_OTH sectors	other_industries
SERVICES	Services	DOM_COM subsectors, MSW	items_services
RAIL	Trains	TRA_OT_RAI	rail
Ships	Sea-going ships	TRA_OTS_X	ships
INW	Ships on inland waterways	TRA_OT_INW	inw
CONSTRUCTION	Construction machinery	TRA_OT_CNS, TRA_OT_LD2, TRA_OTH_LB	construction

As seen in Table 118, it seems that no specific provision for biomass was made and thus it is included with coal-fired power plants. In GAINS, energy-related emissions in non-metallic minerals (largely cement production) are all absorbed into process-related emissions.

The following approach is used for the mapping. Emissions from GAINS sectors (third column table *119*) are aggregated to an intermediary sector (first column) and then distributed among the MRIO sectors belonging to the clusters in the final column using their relative shares in the energy consumption. This is illustrated further for agricultural machinery and combustion devices in figure 189.

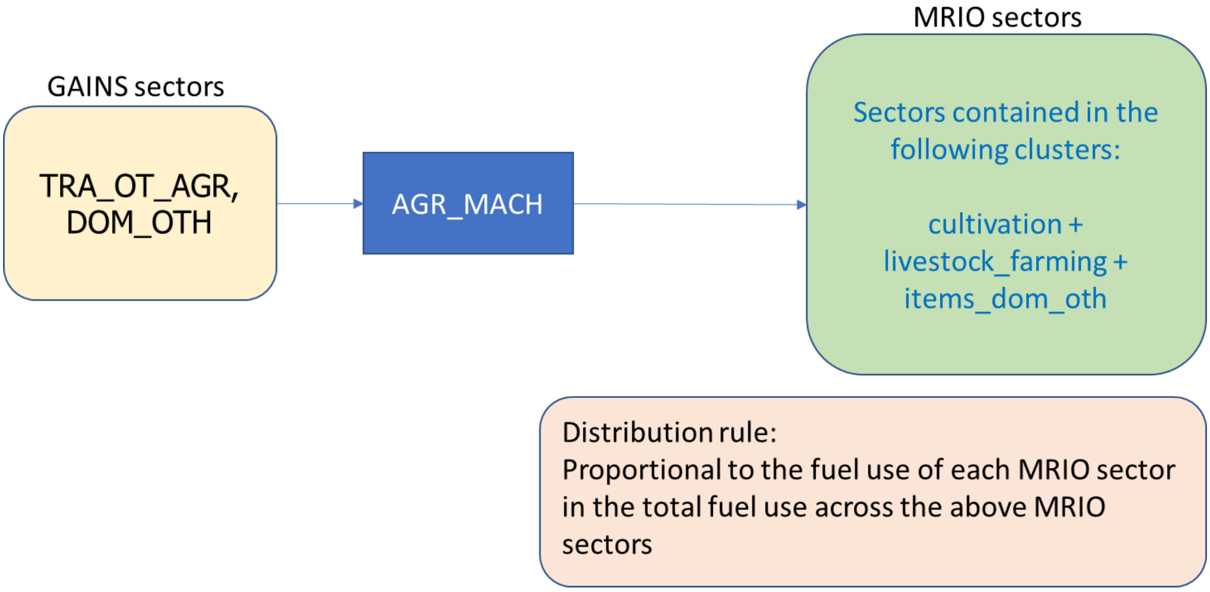

Figure 190: Approach for distributing emissions from agricultural machinery and devices (mobile and stationary) to MRIO sectors.

The energy related emissions in region *r* for MRIO sector *m* are thus:

$$\mathrm{Em}_r(m,e) = \sum_{label} \mathrm{Em}_r(\mathrm{label},\mathrm{e}) \cdot \mathrm{sh}_r(m,\mathrm{e},\mathrm{label}) \tag{5}$$

Where the sum is running over all labels given in Table 117 and the share

$$\mathrm{sh}_r(m,\mathrm{label},\mathrm{e}) = \frac{\mathrm{FE}_r^*(m, label)}{\sum_{m'} \mathrm{FE}_r^*(m', label)} \tag{6}$$

is the share of MRIO sector *m* in the final energy demand (minus trucking) in the total final energy demand (minus trucking) in cluster *label* in region *r*.

Process-related emissions are calculated in GAINS separately from energy-related emissions, i.e., there are separate source categories for these in GAINS. Again, intermediary aggregation sectors, this time relevant for the processes, are defined as follows:

Table 120: Aggregated process-related sectors, their description and coverage in terms of GAINS sectors as well as MRIO clusters.

Label	Description	GAINS sector coverage	MRIO clusters
AGR_PROC	Process emissions related to cultivation	FCON_X, AGR_ARABLE, WASTE_AGR, APPLIC_X, GRAZE_X, STH_NPK, STH_AGR	cultivation
PROC_CATTLE	Emissions related to cattle farming	AGR_COWS, AGR_BEEF	Cattle farming (single sector)
PROC_PIG	Emissions related to pig farming	AGR_PIGS	Pigs farming (single sector)
PROC_POULT	Emissions related to poultry farming	AGR_POULT	Poultry farming (single sector)
PROC_OTANI	Emissions related to farming of other animals	AGR_OTANI	Meat animals nec (single sector)
PROC_BRICK	Emissions related to brick production	PR_BRICK	manuf_bricks
PROC_CEM	Emissions related to cement production	PR_CEM, PR_LIME	manuf_cem
PROC_NMMI	Emissions related to other non-metallic minerals	PR_NMMI, PR_GLASS	manuf_nmmi
PROC_IS	Emissions related to iron and steel production	PR_EARC, PR_BAOX, PR_HEARTH, PR_CAST, PR_SINT, PR_SINT_F, PR_PIGI, PR_PIGI_F, PR_CAST_F	manuf_is
PROC_ALU	Emissions related to aluminium production	PR_ALPRIM, PR_ALSEC	manuf_alu
PROC_FERT	Emissions related to fertilizer production	PR_FERT, FERTPRO	manuf_fert
PROC_CHEM	Emissions related to other chemical processes	PR_SUAC, PR_CBLACK	manuf_chem
PROC_PULP	Emissions related to paper and pulp production	PR_PULP	manuf_paper
PROC_CONVERSION	Emissions related to energy conversion	PR_REF, PR_COKE, STH_COAL, PR_PELL	ind_conversion
PROC_COAL_MINE	Emissions related to coal mining	MINE_HC, MINE_BC, PR_BRIQ	mining_coal_io
PROC_OTHER_MINE	Emissions related to other mining	STH_FEORE, MINE_OTH, STH_OTH_IN	mining_other_io
PROC_SM_IND	Emissions related to other small industries	PR_SMIND_F, OTHER_VOC, PR_OT_NFME, PR_OTHER, OTHER_PM	other_industries
PROC_CONSTRUCT	Emissions related to construction activities	CONSTRUCT	construction

The process related emissions in region r for MRIO sector m are thus:

$$\mathrm{Em}_r(m,p) = \sum_{label} \mathrm{Em}_r(\mathrm{label},\mathrm{p}) \cdot \mathrm{sh}_r(m,\mathrm{e},\mathrm{label}) \quad (7)$$

Where the sum is running over all labels given in Table 120: and the share

$$\mathrm{sh}_r(m,\mathrm{label},\mathrm{e}) = \frac{\mathrm{FE}_r^*(m, label)}{\sum_{m'} \mathrm{FE}_r^*(m', label)} \quad (8)$$

is the share of MRIO sector m in the final energy demand (minus trucking) in the total final energy demand (minus trucking) in cluster *label* in region r. The main difference to the energy related emissions is that the clusters are different, and thus the shares for each sector within a cluster may be different.

As noted above it is a simplification to distribute the process emissions proportional to the energy use in the MRIO sector within its corresponding cluster, and refinements could be made on the basis of information as to which of the MRIO sectors within a cluster are mostly related to the process emissions and in what proportion.

The total emissions associated with MRIO sector m is then simply the sum of the above energy-related, process-related, and trucking-related emissions of $PM_{2.5}$:

$$\mathrm{Em}_r(m) = \mathrm{Em}_r(m,e) + \mathrm{Em}_r(m,p) + \mathrm{Em}_r(m,t) \quad (9)$$

Data

EXIOBASE is used for the global MRIO table and CO_2 emission inventory for the year 2022.[430] In EXIOBASE, 44 territories and five rest of the world regions are covered in the resolution of 163 industrial sectors. The associated CO_2 emission inventory is mapped on a one-to-one sectorial resolution. Hence, consumption-based CO_2 can be easily obtained using equation (1).

To present the results in HDI country groups, the 44 territories are aggregated in accordance with HDI classification developed by UNDP. In the case of the five rest of the world regions, disaggregation of both consumption-based and production-based CO_2 inventories has been conducted in proportion to the national total 2022 production-based CO_2 emissions provided by the Global Carbon Project 2023.[431] Since the 2022 MRIO table and CO_2 emission inventory in EXIOBASE is an extrapolation from historical data, adjustments are made accordingly to both the world MRIO table and CO_2 emission inventory in EXIOBASE. Specifically, change ratio of countries' GDPs[432] from 2021 to 2022 are used to adjust for the domestic intermediate and final consumptions for all countries in the global MRIO table. Change ratio of countries' exports[433] from 2021 to 2022 are used to adjust for the intermediate and final exported consumptions for all countries in the global MRIO table. 2022 global CO_2 emission data from the Global Carbon Project 2023[431] is used to adjust the total CO_2 emissions of countries. Similarly, upon the derivation of production-based $PM_{2.5}$ emission inventory using GAINS model, consumption-based $PM_{2.5}$ emission inventory can be easily obtained using equation (1). As for the five rest of the world regions, production-based emissions are disaggregated in proportion to 2015 $PM_{2.5}$ emission inventory of EDGAR database.[434] Consumption-based emission ratio of the five rest of the world regions is estimated based on CO_2 emission inventories. Having consumption-based and production-based inventories for both CO_2 and $PM_{2.5}$ emissions ready, countries are grouped according to HDI levels for results analysis.

World Bank population data is used to calculate the per capita CO_2 and PM2.5 emissions of different HDI development groups, in accordance with previous calculations. By simply dividing emissions with populations, Figure 191 is produced to show the difference in per capita emissions.

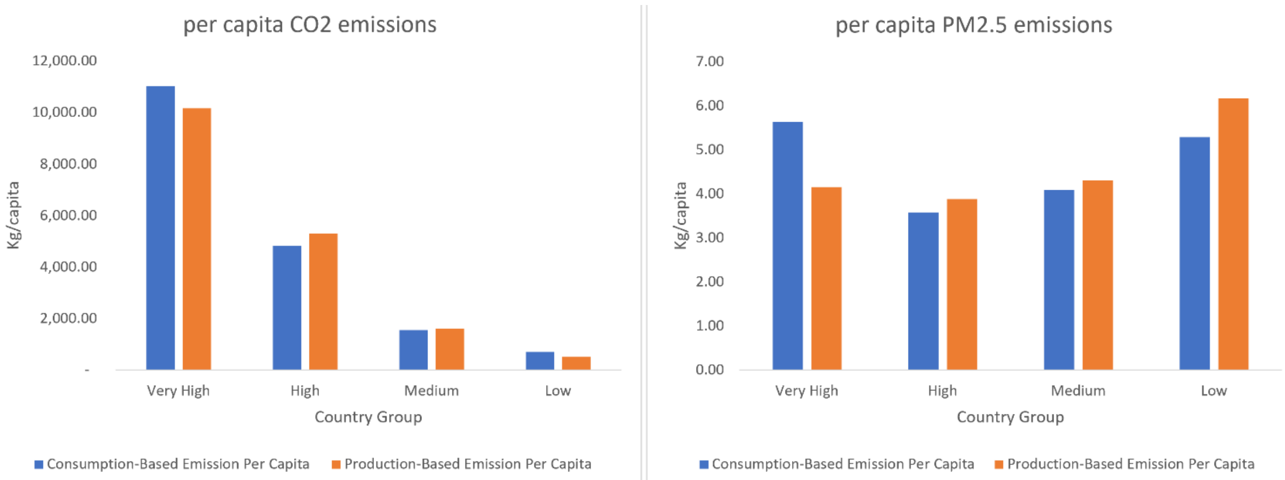

Figure 191: Per capita CO_2 and PM2.5 emissions of different HDI country groups.

Caveats

The GAINS model separating $PM_{2.5}$ emissions into three groupings appears necessary for the following reasons. First, a simplification here is done just on the basis of the total fuel use, rather than on the basis of fuel specific data, though this could be further refined in the next version of this mapping tool. Second, process-related emissions are typically related to specific sectors and thus distributing the emissions among the same cluster as the energy-related emissions seems to introduce a smearing out that is not justified. Thus, process emissions from GAINS are distributed not across all MRIO sectors, but only across those that can be clearly identified with a particular process, and those for which a process emission cannot be further resolved. Finally, trucking-related emissions are distributed among all sectors on the basis of their diesel consumption. It is assumed that the relative share of diesel consumption for road transport in each MRIO sector is generally a good proxy for the relative share in the trucking-related emissions.

In the stage of emission inventory disaggregation, simplifications and assumptions may bring uncertainties into the results. When disaggregating the five rest of the world regions, unavailable data are either filled by emissions from previous years or estimated based on the structure of embodied emissions of other pollutants. The analysis can be updated when more accurate emission inventory becomes available in the future.

Additional analysis

One of the main contributions of this work is a mapping between GAINS sectors and MRIO tables via the EXIOBASE energy extension. This is a powerful tool that maps production-based accounts of primary $PM_{2.5}$ to MRIO tables and therefore easily to consumption-based accounting schemata (figure 192). So far, the analysis has focused on historical data, but the GAINS framework offers also prominently future perspectives in the form of scenarios. Thus, in conjunction with methods to project

MRIO tables, the present methodology could be used to combine future emissions scenarios with future MRIO tables to assess future consumption patterns.

A number of simplifications have been made that could be refined in the next version of the mapping tool to increase the accuracy of the mapping. The mapping in this exercise is a viable tool to relate process-based calculations to consumption-based accounting frameworks. However, it is understood that the linking of frameworks that were built with different purposes (MRIO as an inventory relating economic inputs to economic outputs; GAINS as an integrated tool for air quality policy decision support based on forward looking scenarios) may result in conceptual anomalies. Furthermore, while numerical results are provided at high sectoral and regional resolution, it is important to keep in mind that at this level the results are more uncertain than at an aggregated level. Further to the mapping process, assumptions and estimations made due to unavailable data points in the inventories will exacerbate uncertainties. In the future, the present methodology will be refined to reflect additional insights that will arise through the application of the method to different circumstances or updated inventories.

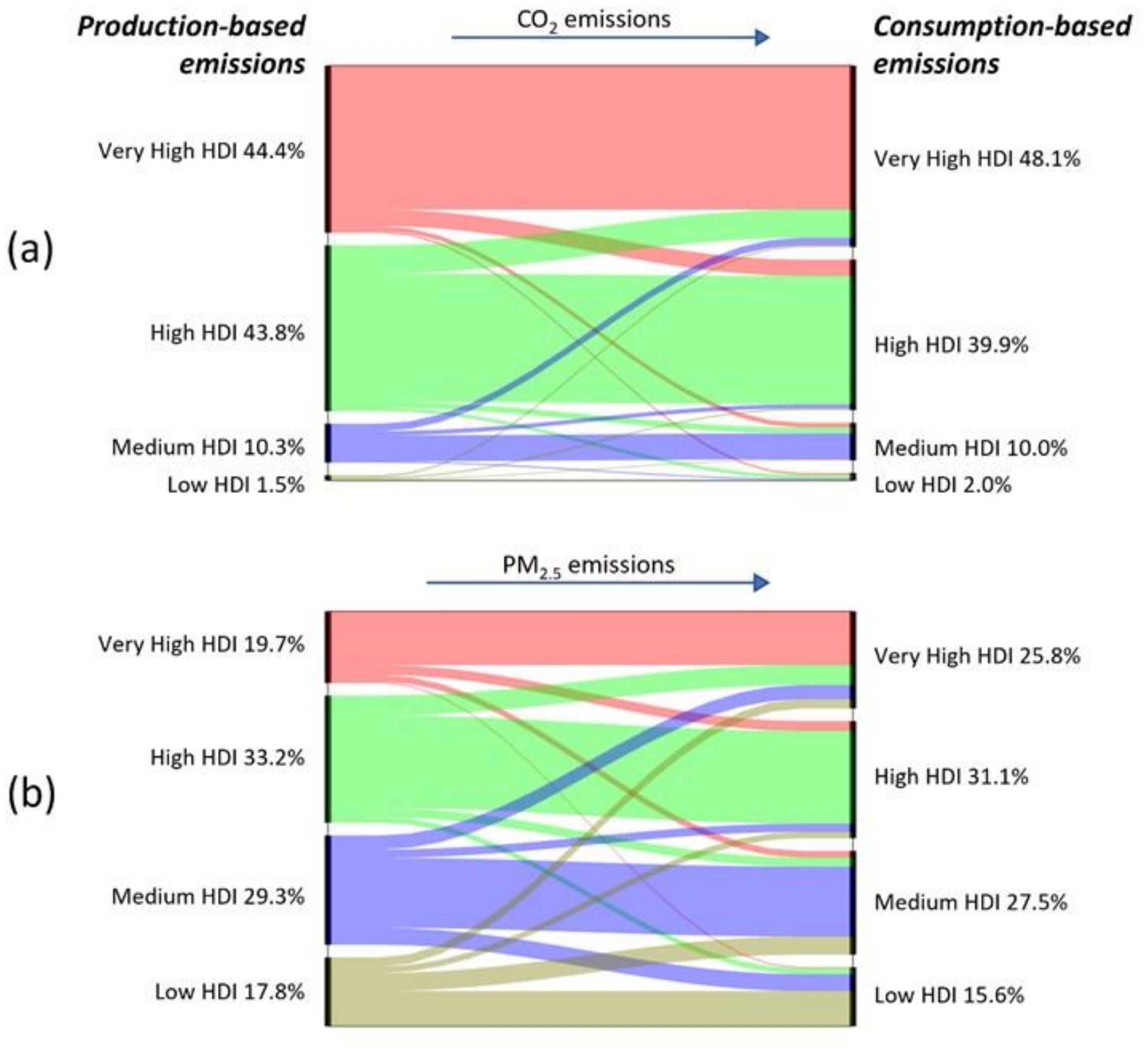

Figure 193: The flows of CO_2 and $PM_{2.5}$ emissions among countries grouped according to Human Development Index (HDI), 2022.

4.3: Financial transitions for a healthy future

Indicator 4.3.1: clean energy investment

Indicator authors

Dr Daniel Scamman

Methods

The data for this indicator is sourced from the 2024 IEA World Energy Investment (WEI) publication.[435]

Investment category definitions

Key categories of investment are defined for the 2024 report as follows:

Fossil fuels – investment in coal, oil and gas electricity generation capacity and fuel supply without CCUS (carbon capture utilisation and storage), but not transitional fossil fuels.

Clean energy – investment in renewable power, nuclear, electricity networks, storage, clean fuels, transitional fossil fuels, CCUS, and end-use in the power and fuels sectors.

Clean fuels – the 2024 WEI report does not contain a clear definition of clean fuels.[436] The 2023 WEI report says that clean fuels includes bioenergy, hydrogen and CCUS;[437] this definition is assumed in this report. Alternatively, if clean fuels are taken to be the same as low-emission fuels, the IEA's World Energy Outlook 2023 report defines low-emission fuels as including modern bioenergy, low-emissions hydrogen and low-emissions hydrogen-based fuels.[438]

End-use – includes energy efficiency, other end-use and other end-use renewables in the buildings, industry and transport sectors (*Table 121*), and includes a range of technologies including heat pumps and electric vehicles. In *Figure 194*, Other end-use includes both other end-use, and other end-use renewables.

Energy efficiency – An energy efficiency investment is defined as the incremental spending on new energy-efficient equipment or the full cost of refurbishments that reduce energy use.

For most sectors, 'investment' is defined as ongoing capital spending on assets. For some sectors, such as power generation, this investment is spread out evenly from the year in which a new plant or upgrade of an existing one begins its construction to the year in which it becomes operational. For other sources, such as upstream oil and gas and liquefied natural gas (LNG) projects, investment reflects the capital spending incurred over time as production from a new source ramps up or to maintain output from an existing asset. This definition differs from the definition previously employed by the IEA before 2019, in which investment was defined as overnight capital expenditure.

	Buildings	Industry	Transport
**Energy efficiency**	• Building materials (envelope and interior)<br>• Appliances and lighting	• Industrial energy management systems<br>• Fuel efficiency	• Road vehicles (passenger light duty vehicles, light<br>• commercial vehicles, heavy-freight traffic vehicles,

	• HVAC (heating, ventilation, and air conditioning)<br>• Smart meters	• Electrical efficiency<br>• Heat pumps	• medium-freight traffic vehicles and other road vehicles) and rail transport
**Other end-use**	• Heat pumps	• Industry CCUS	• Road electric vehicles<br>• Private EV chargers<br>• Rail transport
**Other end-use renewables**	• Bioenergy<br>• Geothermal<br>• Solar home systems<br>• Other renewables	• Bioenergy<br>• Geothermal<br>• Thermal solar	

Table 121: End-use categories (adapted from IEA WEI 2024 report methodology annex)[435]

Regional analysis

New for the 2024 report, this indicator also includes an analysis of regional investment. The WEI report does not supply country-level data, but groups countries according to the regions in Table 122. It is not possible to regroup this data according to the standard Lancet Countdown groupings, so the WEI groupings are retained. For this report, the data for China is subtracted from the Asia Pacific data to allow China's investment behaviour to be analysed separately from the rest of the Asia Pacific region.

Table 122: Country inclusion in IEA WEI report regional groupings

**Advanced economies**	OECD regional grouping and Bulgaria, Croatia, Cyprus, Malta and Romania
**Emerging market and developing economies**	All other countries not included in the advanced economies regional grouping, China is also excluded
**China**	The (People's Republic of) China and Hong Kong, China
**North America**	Canada, Mexico and United States
**Central and South America**	Argentina, Plurinational State of Bolivia (Bolivia), Brazil, Chile, Colombia, Costa Rica, Cuba, Curaçao, Dominican Republic, Ecuador, El Salvador, Guatemala, Haiti, Honduras, Jamaica, Nicaragua, Panama, Paraguay, Peru, Suriname, Trinidad and Tobago, Uruguay, Bolivarian Republic of Venezuela (Venezuela), and other Central and South American countries and territories
**Europe**	European Union and Albania, Belarus, Bosnia and Herzegovina, North Macedonia, Gibraltar, Iceland, Israel, Kosovo, Montenegro, Norway, Serbia, Switzerland, Republic of Moldova, Turkey, Ukraine and United Kingdom
**Africa**	Algeria, Angola, Benin, Botswana, Cameroon, Congo, Democratic Republic of Congo, Côte d'Ivoire, Egypt, Eritrea, Ethiopia, Gabon, Ghana, Kenya, Libya, Mauritius, Morocco, Mozambique, Namibia, Niger, Nigeria, Senegal, South Africa, South Sudan, Sudan, United Republic of Tanzania, Togo, Tunisia, Zambia, Zimbabwe and Other Africa

**Middle East**	Bahrain, Islamic Republic of Iran (Iran), Iraq, Jordan, Kuwait, Lebanon, Oman, Qatar, Saudi Arabia, Syrian Arab Republic (Syria), United Arab Emirates and Yemen
**Eurasia**	Armenia, Azerbaijan, Georgia, Kazakhstan, Kyrgyzstan, Russia, Tajikistan, Turkmenistan and Uzbekistan
**Asia Pacific**	Australia, Bangladesh, Brunei Darussalam, Cambodia, China, DPR of Korea, India, Indonesia, Japan, Korea, Laos, Malaysia, Mongolia, Myanmar, Nepal, New Zealand, Pakistan, Philippines, Singapore, Sri Lanka, Chinese Taipei, Thailand Viet Nam and Other Asia, Afghanistan, Bhutan, Cook Islands, East Timor, Fiji, French Polynesia, Kiribati, Macau, Maldives, New Caledonia, Papua New Guinea, Samoa, Solomon Islands, Tonga and Vanuatu

Data

1. IEA World Energy Investment 2024.[435]

Caveats

Other areas of expenditure, including operation and maintenance, research and development, financing costs, mergers and acquisitions or public markets transactions, are not included. Investment estimates are derived from IEA data for energy demand, supply and trade, and estimates of unit capacity costs, For more information, see IEA World Energy Investment 2024.

Additional analysis

The variation in global clean energy and fossil fuel investment for 2015-2023 is shown in Figure 194 below. Similar and stable levels of clean energy and fossil fuel investment were recorded for 2016-2020, but a sharp rise in clean energy investment occurred since 2020 with double-digit growth occurring in each of the last three years. Fossil fuel investment dipped in 2020 due to Covid, but has recovered each year since then though investment has not yet regained 2020 levels. This has resulted in a clear divergence in clean energy investment compared to fossil fuels since 2020, with clean energy investment now substantially exceeding fossil fuel spending and with the gap appearing to be growing (the average 3-year growth rate of clean energy investment for 2020-2023 of 14.6% was over twice the growth rate for fossil fuels of 6.7%). Although recent higher interest rates have increased borrowing costs, this has been partially offset by easing supply chain pressures and falling prices.

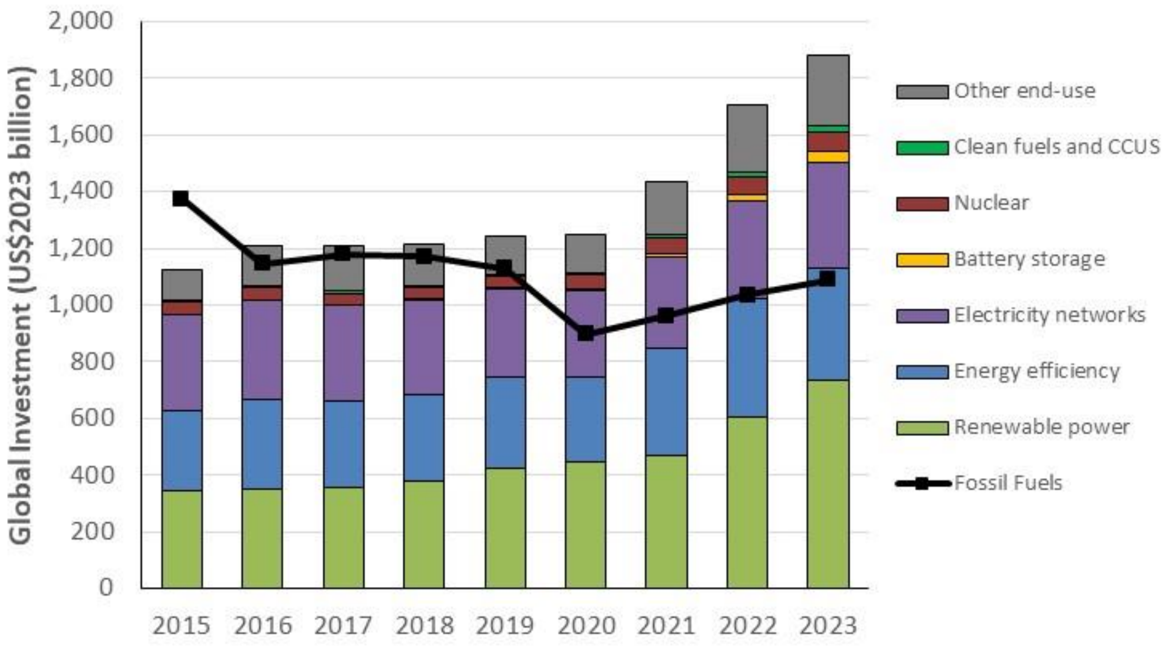

Figure 194: Global energy investment in clean energy (columns) and fossil fuels (solid line).

The variation in 2023 investment in fossil fuels and clean energy for the different WEI regions is shown in Figure 195. China was the leading investor in clean energy in 2023 (46.2% of the global total), but also the second leading investor in fossil fuels. Europe was the second leading investor in clean energy, spurred by cuts in gas deliveries following Russia's invasion of Ukraine, and invested over 5 times more in clean energy than in fossil fuels in 2023, though this partly reflects Europe's reliance on imported fuels. North America was the third leading investor in clean energy in 2023, supported by measures such as the US Inflation Reduction Act, but still invested nearly the same amount in fossil fuels with the US currently the world's largest oil and gas producer. Clean energy investment in Africa has increased by over 60% since 2020, but debt repayments, low debt ratings and high costs of capital are hampering the access of finance for capital-intensive clean energy projects. Fossil fuel investment exceeded clean energy investment by a factor of over 5 in the Middle East despite the region's potential for clean energy (particularly solar), reflecting the ongoing demand for the region's fossil fuels. Africa, Middle East and Eurasia combined invested 28.2% of global fossil fuel spending, but only 4.4% of clean energy investment, demonstrating the size of the challenge in shifting these regions to clean energy.

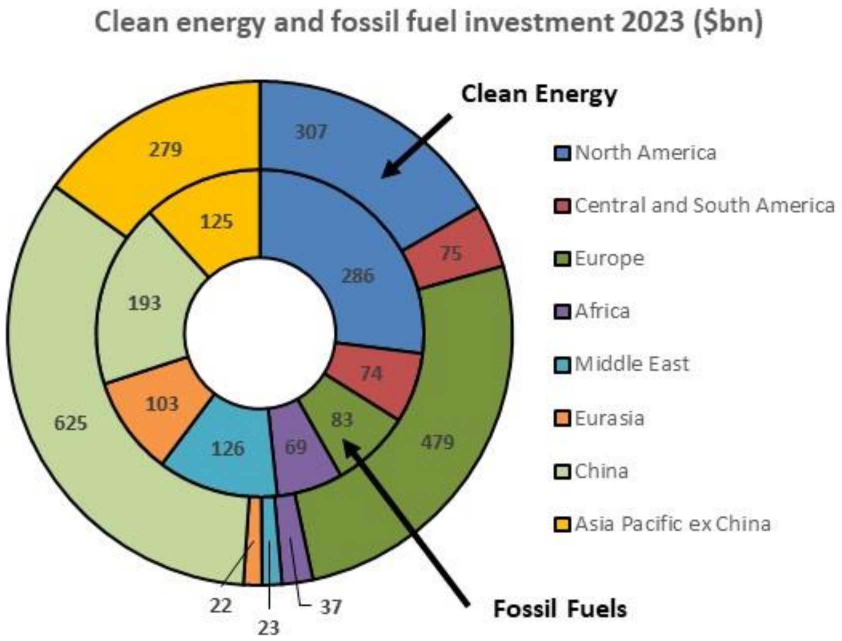

Figure 195: Variation in 2023 clean energy (outer ring) and fossil fuel investment (inner ring) according to WEI regions ($2023 bn).

The historical variation in the ratio of clean energy and fossil fuel spending is shown in Figure 196. Globally, clean energy investment as a fraction of fossil fuel spending has more than doubled from 83% in 2015 to 173% in 2023, demonstrating the global shift to clean energy investment. Europe has the highest ratio of clean energy spending, followed by China and Asia Pacific ex China, with all three regions notably increasing their spending ratio since 2015. Africa has also more than doubled their spending ratio since 2015, though it still remains well below parity. Other regions have flat-lined; Central and South America is at the same level it was at in 2016, and North America at the same level it was in 2020. Others have regressed; the Middle East's ratio of clean energy spending has dropped from 23% in 2017 to 19% in 2023.

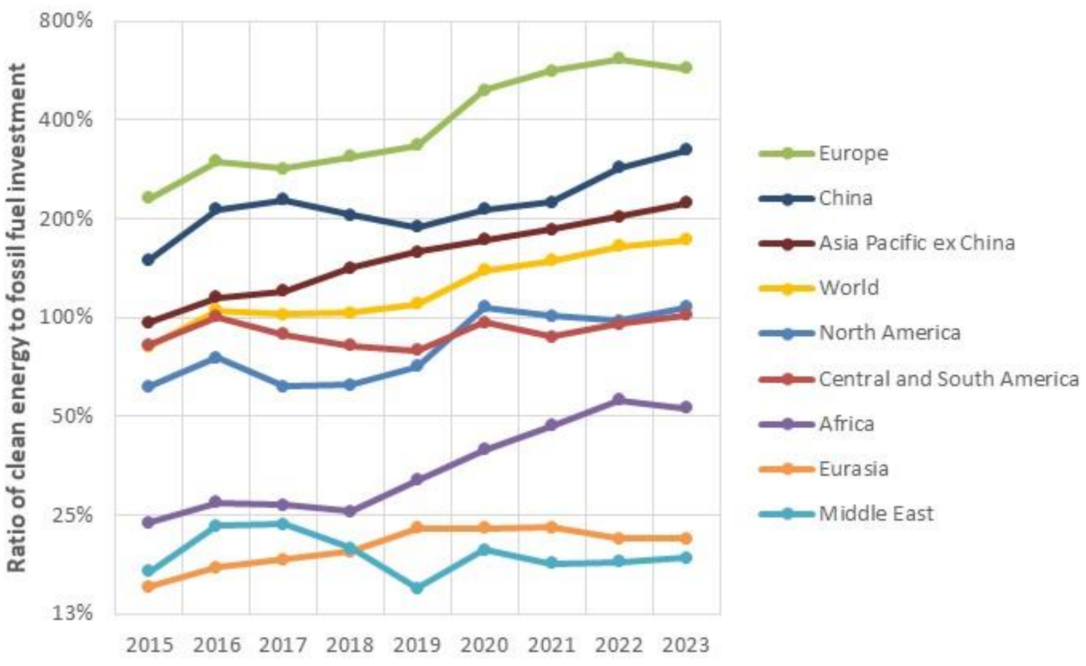

Figure 196: Ratio of clean energy to fossil fuel investment according to WEI regions.

Indicator 4.3.2: funds divested from fossil fuels

Indicator authors

Dr Daniel Scamman

Methods

The data for this indicator is collected and provided by stand.earth. Prior to this report, they represented the total assets (or assets under management, AUM) for institutions that have publicly committed to divest (for which data is available), with non-US$ values converted using the market exchange rate when the commitment was made, and thus did not directly represent the actual sums divested from fossil fuel companies. For the data used in this report, AUM data has been updated to 2023 levels. A company is committed to 'divestment' if it falls into any of the following five categories:

- **'Fossil Free' -** An institution or corporation that does not have any investments (direct ownership, shares, commingled mutual funds containing shares, corporate bonds) in fossil fuel companies (coal, oil, natural gas) and committed to avoid any fossil fuel investments in the future
- **'Full' - A**n institution or corporation that made a binding commitment to divest (direct ownership, shares, commingled mutual funds containing shares, corporate bonds) from any fossil fuel company (coal, oil, natural gas).
- **'Partial' -** An institution or corporation that made a binding commitment to divest across asset classes from some fossil fuel companies (coal, oil, natural gas), or to divest from all fossil fuel companies (coal, oil, natural gas), but only in specific asset classes (e.g., direct investments, domestic equity).

- **‘Coal and Tar Sands’** - An institution or corporation that made a binding commitment to divest (direct ownership, shares, commingled mutual funds containing shares, corporate bonds) from any coal and tar sands companies.
- **‘Coal only’** - An institution or corporation that made a binding commitment to divest (direct ownership, shares, commingled mutual funds containing shares, corporate bonds) from any coal companies.

Eight organisations that were originally recorded as non-healthcare institutions have been considered as such for the purpose of this indicator (London School of Hygiene and Tropical Medicine, The Royal College of General Practitioners, New Zealand Nurses Organisation, HESTA, HCF, Berliner Ärzteversorgung, Doctors for the Environment Australia, and the Royal College of Emergency Medicine). Divestment commitments by the American Medical Association, which divested in 2018, was not included in the data provided by 350.org, and was added separately.

Data

- Global Fossil Fuel Divestment Commitments Database from Stand.earth.[439]

Caveats

Data on the number of institutions that have divested, and the value of their assets is dependent on institutions reporting this information to Stand.earth.

Additional analysis

The cumulative value of divestment (both global total and for healthcare institutions) is presented below (Table *123*). Organisations that have divested but for which no date of divestment (a total of $1.78 billion) are recorded in a separate column, with the total assumed to begin in 2008 in the absence of more detailed information.

Table 124: Cumulative fossil fuel divestment.

	Global	Global (including data with no divestment date)	Healthcare Institutions
2008	$16	$1,780,396	$-
2009	$17	$1,780,396	$-
2010	$17	$1,780,396	$-
2011	$84	$1,780,464	$-
2012	$3,773	$1,784,152	$-
2013	$9,194	$1,789,574	$-
2014	$441,604	$2,221,983	$37,809
2015	$2,567,887	$4,348,267	$38,103
2016	$3,586,778	$5,367,157	$41,010
2017	$5,636,405	$7,416,784	$53,191
2018	$8,289,621	$10,070,001	$54,107
2019	$11,844,100	$13,624,479	$54,120
2020	$28,010,969	$29,791,349	$54,187
2021	$38,644,823	$40,425,202	$54,187
2022	$38,732,285	$40,512,665	$54,187
2023	$38,886,060	$40,666,440	$54,307

* US$ million (2023 data)

Due to confidentiality issues, the full dataset is not available for publication. However, interested readers may visit the www.divestmentdatabase.org for further information.

Additionally, Figure 197 illustrates the cumulative divestment globally from 2013 to 2023, with a specific focus on healthcare institutions.

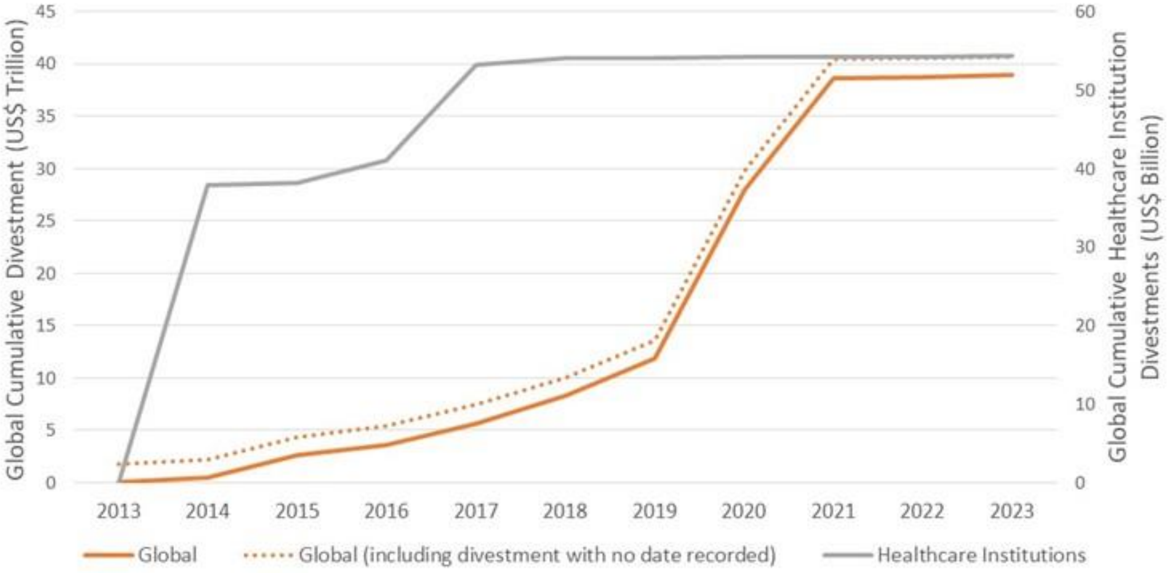

Figure 198: Cumulative divestment – global total and in healthcare institutions

Indicator 4.3.3: net value of fossil fuel subsidies and carbon prices

Indicator authors

Dr Daniel Scamman

Methods

Fossil Fuel Subsidies

Data for fossil fuel subsidies were taken from two sources. The IEA provides data on fossil fuel consumption subsidies for 48 countries, calculated using its 'price gap' approach – the difference between the end-user prices paid for fossil fuels in the country, and reference prices that account for the full cost of supply.[440,441] However, the countries provided in this list are mainly non-OECD. The OECD itself provides estimates of fossil fuel subsidies within the 38 OECD countries, plus Argentina, Armenia, Azerbaijan, Brazil, China, Georgia, India, Indonesia, Moldova, and South Africa — a total of 48 countries.[442] OECD's estimates are derived from a bottom-up inventory of subsidy mechanisms within each country, and include production and consumption support, infrastructure investments, incentives and R&D. It divides the type of support into three broad categories: Consumer Support Estimate (CSE), Producer Support Estimate (PSE) and General Services Support Estimate (GSE). Combining the IEA and OECD datasets allows a coverage of 80 countries, after accounting for overlaps and the omission of countries not covered by the *Lancet* Countdown. The OECD describes an approach for combining these two datasets, and reconciling different estimates for the countries covered by both.[443] This involves selecting line items in the OECD inventory that correspond to the price-gap definition of subsidies that is the basis of the IEA data – i.e., measures that bring about reduced consumer prices: '*conceptually, an OECD estimate derived from individual measures that capture transfers to consumers from producers and taxpayers should match the IEA price-gap estimates'* (p.22-3).[443]

The description of this approach suggests that in the few cases of countries whose subsidies have been calculated by both OECD and IEA, the OECD estimate would be expected to be the larger of the two.[443] However, analysis of overlapping countries suggests that it is in fact more often the IEA estimate that is larger. This analysis is described in more detail in the appendix of the 2020 *Lancet* Countdown report. The conclusion drawn from this is that attempting to separate some line items from the OECD estimates that seem more directed at consumers is not a reliable way of reconciling the two estimates – on the contrary, in several cases it makes the gap between the two larger by making the OECD estimate smaller. Consequently, in considering countries that overlap between the two datasets as part of preparing this indicator, a comparison was made simply between the total OECD estimate and the total IEA estimate.

Following a simple rule of thumb proposed by OECD, in order to decide which estimate to use in overlapping cases, the source that produces the larger cumulative total for a given country over the years being considered, was the one chosen as the source for that country for this indicator.[443]

Carbon prices and revenues

Information on carbon prices and carbon pricing revenues was sourced from the World Bank Carbon Pricing Dashboard.[444] Revenues from each recorded instrument were allocated to the nation state within which the instrument operated. Shares of the EU ETS revenues were allocated to each of the

participants in the EU ETS – that is the 28 members of the EU (which included the UK until the end of 2020), plus Iceland and Norway. The UK was allocated a share of ETS until the end of 2020 as, although the UK left the EU on 31 January 2020, the UK remained subject to EU rules until 31 December 2020 with the UK's own ETS replacing its participation in the EU ETS from 1 January 2021. Liechtenstein is also an EU ETS member but could not be included in this analysis due to lack of CO_2 emissions data. The allocation of EU ETS revenues was made to participating states on the basis of their share of the emissions of all EU ETS states, calculated using IEA CO_2 emissions data.[445] This was considered an acceptable simplification given that for the period 2013–2020, 88% of allowances were allocated for auction to participating states in proportion to their emissions.[446] Carbon pricing revenue data was included for El Salvador in 2010, but none has been reported since.

Countries were included in the analysis if data were available for CO_2 emissions, and either fossil fuel subsidies or carbon pricing instruments. This yielded a list of 87 countries in 2020 accounting for 93% of global CO_2 emissions.[445]

Net carbon price and revenue calculations
In reality at present, both carbon prices and fossil subsidies are typically applied to individual sectors or fuels, and do not cover the entire economy. Within different particular jurisdictions the sectors covered by subsidies and carbon prices are often not identical. As such, the only way of producing a consistent indicator across multiple countries is to average out both subsidies and prices across the CO_2 emissions of the whole economy, resulting in net average economy-wide carbon prices and revenues. Each country's total fossil fuel subsidies were subtracted from its total carbon price revenues to produce a net carbon revenue. These figures were divided by the relevant total country CO_2 emissions for each year, using data from the IEA,[445] resulting in the net carbon price. The net carbon revenue was expressed as a proportion of national expenditure on health, using current annual (i.e., not including capital) health expenditure data from the WHO's Global Health Expenditure Database.[447]

Estimating missing 2022 data
The indicator data included in the 2024 report of the Lancet Countdown includes full data for 2021, and also full net carbon revenue data for 2022. CO_2 emissions data was not available for all countries for 2022, so to allow carbon prices to be estimated for the remaining countries, CO_2 emissions were estimated for the missing countries based on the trend in their emissions for 2016–2021 (excluding 2020, where CO_2 emissions were noticeably lower due to COVID). Likewise, health expenditure data was not available for all countries for 2022, so data for the missing countries was estimated based on the trend in their health expenditure for 2015–2019 (excluding 2020 and 2021, where health expenditure was noticeably higher for some countries due to COVID, but lower for some others). Hence the net carbon price and net carbon revenue vs. health expenditure data reported for this indicator for 2022 are estimated for this report, with values to be confirmed in next year's report.

Currency standardisation
All money values are expressed in real 2023 US$. Both the OECD Inventory and the IEA fossil fuel subsidy database provide data in real 2022 US$. These units were corrected to real 2023 values, using the GDP deflator for the US dollar, from the IMF.[343] The World Bank carbon pricing revenue data and the WHO health expenditure data are given in nominal US dollars, so again the US GDP deflator from IMF was applied to correct to real 2023 values.

Data

- Fossil fuel subsidies data from the IEA and OECD[440,442]
- Carbon pricing data from the World Bank Carbon Pricing Dashboard[444]
- CO_2 emissions from fuel combustion from IEA[445]
- Health expenditure data from WHO[447]
- US Dollar GDP deflator index from the IMF World Economic Outlook database[343]

Caveats

The principal caveat is that the indicator is strongly dependent on the reliability of the main datasets from the IEA, OECD, and World Bank. It is possible that data on individual countries may not be fully comprehensive due to reporting errors, lack of information or other issues, as indeed is acknowledged by OECD.[443] The indicator should be considered as a way of illustrating global trends, and caution should be exercised in attempting to draw out specific conclusions relating to individual countries covered by the indicator.

The nature of indicators that draw on multiple datasets is that the most recent year on which they can report is defined by the most recent year that is common to all datasets used. In this case that year was 2020, which was due to this being the most recent complete year for both CO_2 emissions from fuel combustion and health expenditure.

The economy-wide net carbon price was derived by dividing fossil fuel subsidies and carbon pricing revenues by total CO_2 emissions. This fits well with the subsidies, as these are for fossil fuels, the principal source of CO_2. However, some of the carbon pricing instruments from which the revenue was assessed are not only for fossil fuel combustion but apply to other sectors and non-CO_2 gases. There is therefore a slight inconsistency between the sectoral coverage of the subsidies and the carbon pricing instruments.

Additional analysis

Progress since Paris Agreement

Progress since the Paris Agreement is shown in figure 199. Twenty-two countries showed an increase in net carbon revenues between 2016 (the year before the Paris Agreement came into force) and 2022, indicating an increase in the disincentivising of fossil fuels. However, 65 countries showed a decrease in net annual carbon revenues between 2016 and 2022, indicating an increased incentivising of fossil fuels. Similar numbers were observed for the net carbon price and net revenues vs health metrics. The median decrease in net carbon revenues was US$ 2.5 billion, 53 $/tCO2 for the net carbon price, and 7.1% for net revenue as a fraction of the health budget; these comparatively small numbers indicate that a considerable number of nations have shown relatively little change one way or the other since the Paris Agreement was signed. However, the distributions were lopsided at the extremes; the biggest increase in in net carbon revenues since 2016 was only US $8.1 billion, while the largest decrease was US$ 122 billion; 15 nations had decreases in net carbon revenues greater than US$ 25 billion. Overall, carbon price revenues increased 221% from US$ 28 billion in 2016 to US$ 89 billion in 2022, but net fossil fuel subsidies increased from US$ 445 billion in 2016 to US$ 1,431 billion in 2022, an increase of US$ 986 billion (222%), a substantial backward step. Twenty-six nations saw decreases in net carbon prices of over $100 /tCO2 since 2016, and 15 nations saw decreases in net carbon revenues as a fraction of health expenditure of more than 100%. The observed regression was worsened by the Ukraine

invasion, but this itself demonstrates the ongoing vulnerability of the existing global energy system based on fossil fuels to geopolitical crises.

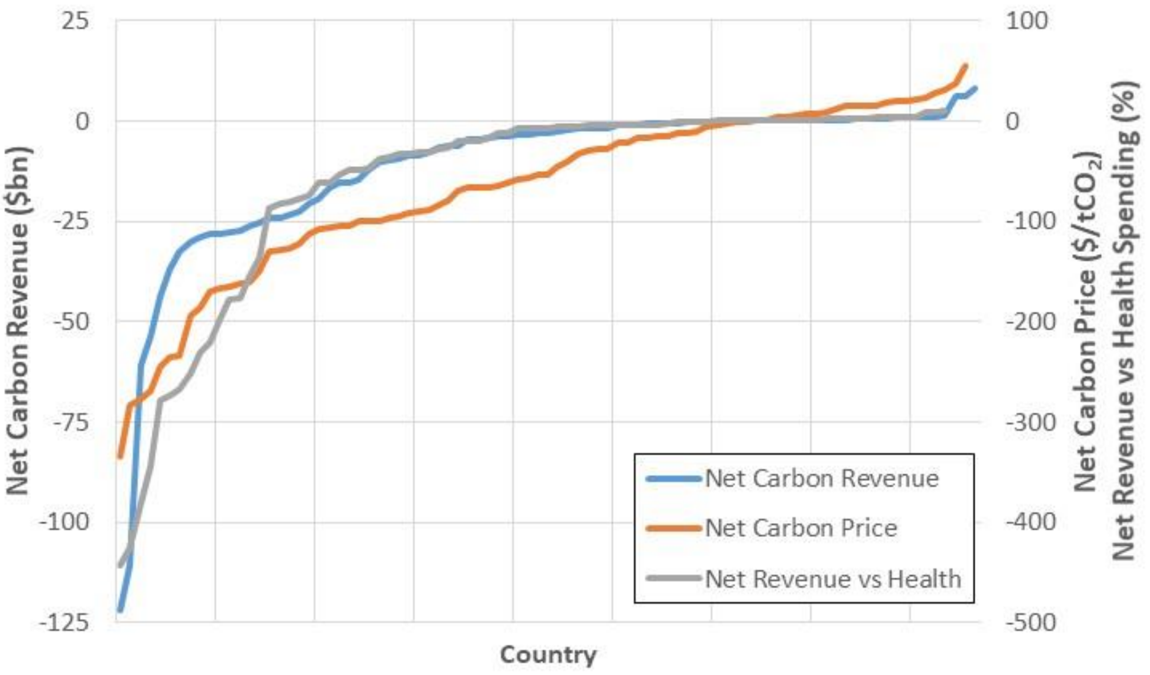

Figure 200: Increases in net carbon revenues, net carbon prices, and net carbon revenue as a share of current national health expenditure from 2016 to 2022.

Country Inclusion

The relevant section in the main report shows net carbon prices, net carbon revenues, and net carbon revenues as a proportion of health spending, by HDI grouping, for the year 2022 (*Table 125*). The following graphs show results for the same three indicators for WHO and LC groupings (*Table 126* and *Table 127*). Due to the finite number of countries included in the indicator, some classifications have fewer inclusions than others, as shown in the tables below for 2022 data. Note the number of countries included has grown since 2010 as additional countries have begun reporting data (except for El Salvador, which reported data in 2010 but not since). Also shown are charts for the same three indicators with all countries grouped together for the years 2010–2022 inclusive (Figure 201, Figure 202, and Figure 203).

Table 125: Inclusions in HDI Groupings for Indicator 4.2.4 for 2020 data

HDI Band	Number	Countries
Low	2	Nigeria, Pakistan
Medium	7	Angola, Bangladesh, Bolivarian Republic of Venezuela, Bolivia, Ghana, India, Iraq
High	21	Algeria, Armenia, Azerbaijan, Brazil, Bulgaria, China, Colombia, Ecuador, Egypt, Gabon, Indonesia, Islamic Republic of Iran, Libya, Mexico, Republic of Moldova, South Africa, Sri Lanka, Turkmenistan, Ukraine, Uzbekistan, Vietnam
Very High	57	Argentina, Australia, Austria, Bahrain, Belgium, Brunei Darussalam, Canada, Chile, Costa Rica, Croatia, Cyprus, Czechia, Denmark, Estonia, Finland, France, Georgia, Germany, Greece, Hungary, Iceland, Ireland, Israel, Italy, Japan, Kazakhstan, Kuwait, Latvia, Liechtenstein, Lithuania, Luxembourg, Malaysia, Malta, Netherlands, New Zealand, Norway, Oman, Poland, Portugal, Qatar, Republic of Korea, Romania, Russian Federation, Saudi Arabia, Singapore, Slovakia, Slovenia, Spain, Sweden, Switzerland, Thailand, Trinidad and Tobago, Turkey, United Arab Emirates, United Kingdom, United States of America, Uruguay
**Total**	**87**	

Table 126: Inclusions in WHO Groupings for Indicator 4.2.4 for 2020 data

WHO Region	Number	Countries
Africa	6	Algeria, Angola, Gabon, Ghana, Nigeria, South Africa
Americas	13	Argentina, Bolivarian Republic of Venezuela, Bolivia, Brazil, Canada, Chile, Colombia, Costa Rica, Ecuador, Mexico, Trinidad and Tobago, United States of America, Uruguay
Eastern Mediterranean	11	Bahrain, Egypt, Iraq, Islamic Republic of Iran, Kuwait, Libya, Oman, Pakistan, Qatar, Saudi Arabia, United Arab Emirates
Europe	43	Armenia, Austria, Azerbaijan, Belgium, Bulgaria, Croatia, Cyprus, Czechia, Denmark, Estonia, Finland, France, Georgia, Germany, Greece, Hungary, Iceland, Ireland, Israel, Italy, Kazakhstan, Latvia, Liechtenstein, Lithuania, Luxembourg, Malta, Netherlands, Norway, Poland, Portugal, Republic of Moldova, Romania, Russian Federation, Slovakia, Slovenia, Spain, Sweden, Switzerland, Turkey, Turkmenistan, Ukraine, United Kingdom, Uzbekistan
South-East Asia	5	Bangladesh, India, Indonesia, Sri Lanka, Thailand
Western Pacific	9	Australia, Brunei Darussalam, China, Japan, Malaysia, New Zealand, Republic of Korea, Singapore, Vietnam
**Total**	**87**	

Table 127: Inclusions in LC Groupings for Indicator 4.2.4 for 2020 data

LC Group	Number	Countries
Africa	8	Algeria, Angola, Egypt, Gabon, Ghana, Libya, Nigeria, South Africa
Asia	29	Armenia, Azerbaijan, Bahrain, Bangladesh, Brunei Darussalam, China, Cyprus, Georgia, India, Indonesia, Iraq, Islamic Republic of Iran, Israel, Japan, Kazakhstan, Kuwait, Malaysia, Oman, Pakistan, Qatar, Republic of Korea, Saudi Arabia, Sri Lanka, Thailand, Turkey, Turkmenistan, United Arab Emirates, Uzbekistan, Vietnam
Europe	34	Austria, Belgium, Bulgaria, Croatia, Czechia, Denmark, Estonia, Finland, France, Germany, Greece, Hungary, Iceland, Ireland, Italy, Latvia, Liechtenstein, Lithuania, Luxembourg, Malta, Netherlands, Norway, Poland, Portugal, Republic of Moldova, Romania, Russian Federation, Slovakia, Slovenia, Spain, Sweden, Switzerland, Ukraine, United Kingdom
Northern America	2	Canada, United States of America,
Oceania	2	Australia, New Zealand,
SIDS	2	Singapore, Trinidad and Tobago
South and Central America	10	Argentina, Bolivarian Republic of Venezuela, Bolivia, Brazil, Chile, Colombia, Costa Rica, Ecuador, Mexico, Uruguay
**Total**	**87**	

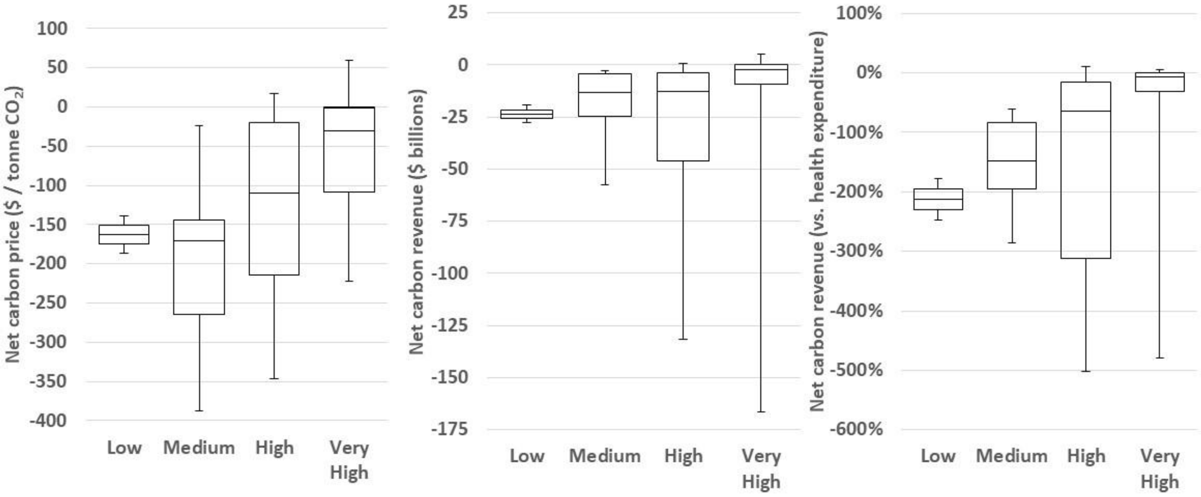

Figure 201: Net carbon prices (left), net carbon revenues (centre), and net carbon revenue as a share of current national health expenditure (right), across 87 countries in 2022, arranged by HDI country group: low (n=2), medium (n=7), high (n=21) and very high (n=57). Boxes show the interquartile range (IQR), horizontal lines inside the boxes show the medians, and the brackets represent the full range from minimum to maximum.

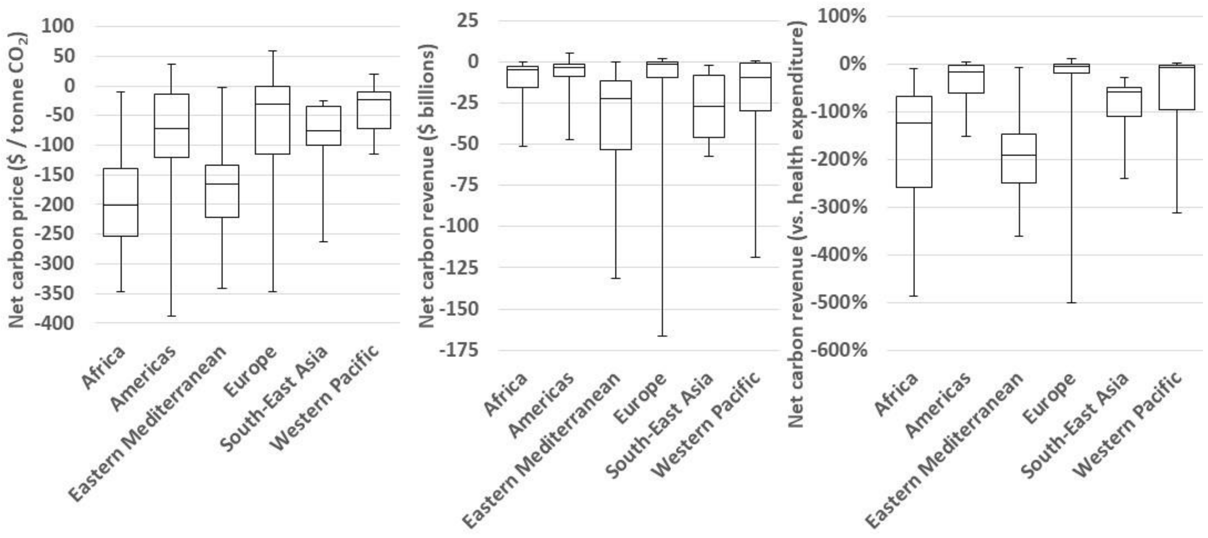

Figure 202: Net carbon prices, net carbon revenues, and net carbon revenue as a share of current national health expenditure, across 87 countries in 2022, grouped by WHO region. Boxes show the interquartile range (IQR), horizontal lines inside the boxes show the medians, and the brackets represent the full range from minimum to maximum.

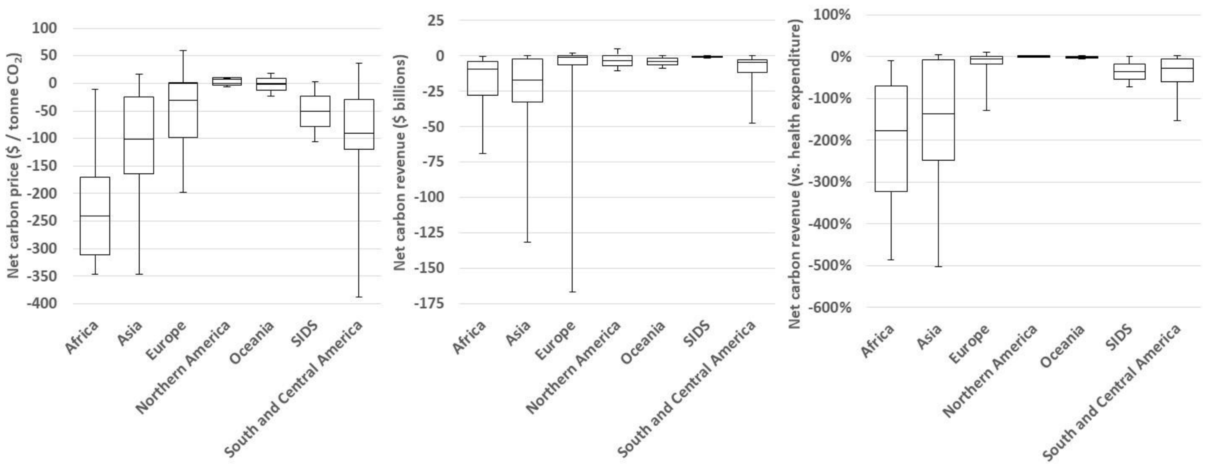

Figure 203: Net carbon prices, net carbon revenues, and net carbon revenue as a share of current national health expenditure, across 87 countries in 2022, grouped by LC grouping.

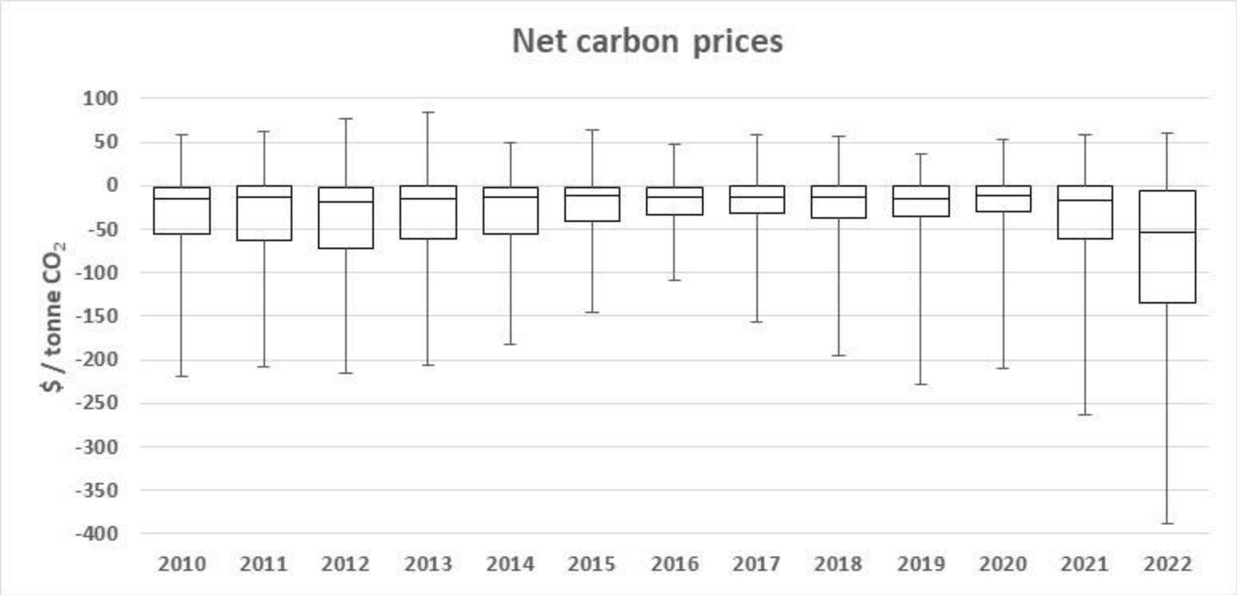

Figure 204. Net carbon prices for all countries included in the analysis, 2010–2022 inclusive.

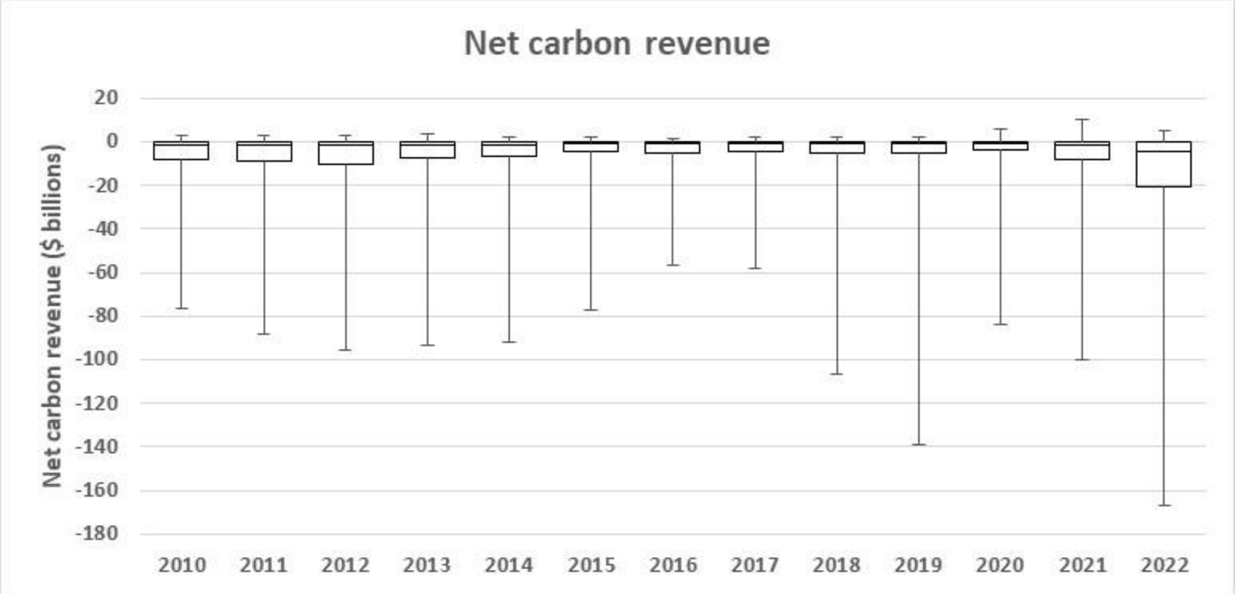

Figure 205. Net carbon revenue for all countries included in the analysis, 2010–2022 inclusive.

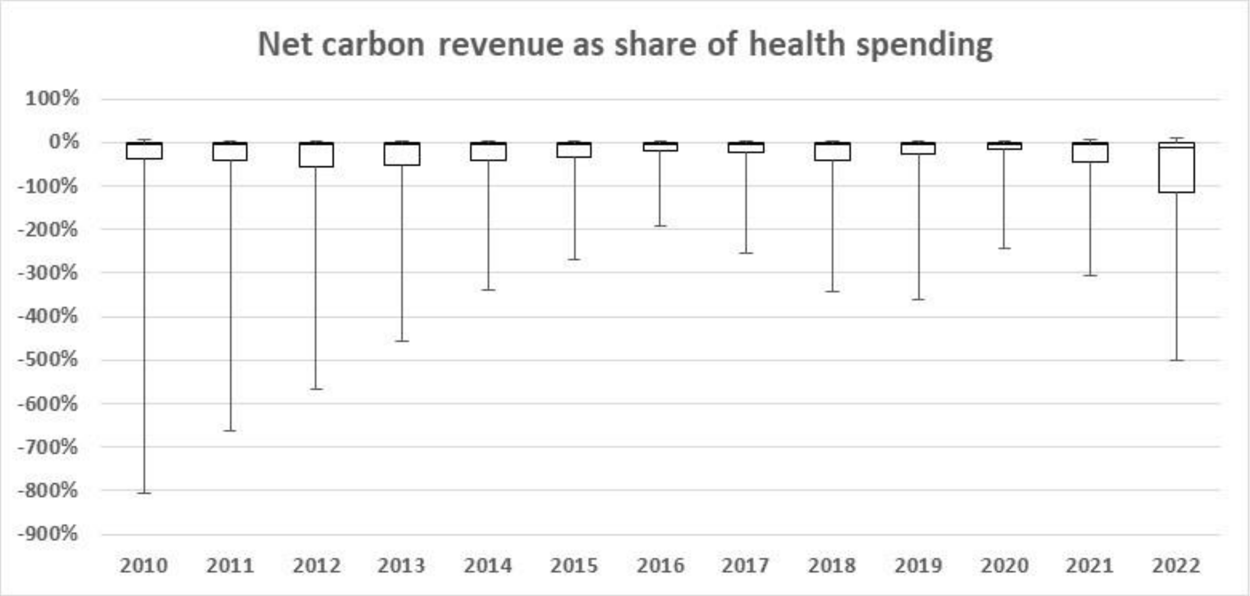

Figure 206. Net carbon revenue expressed as the equivalent share of current (i.e., not capital) annual health spending, for all countries included in the analysis, 2010–2022 inclusive.

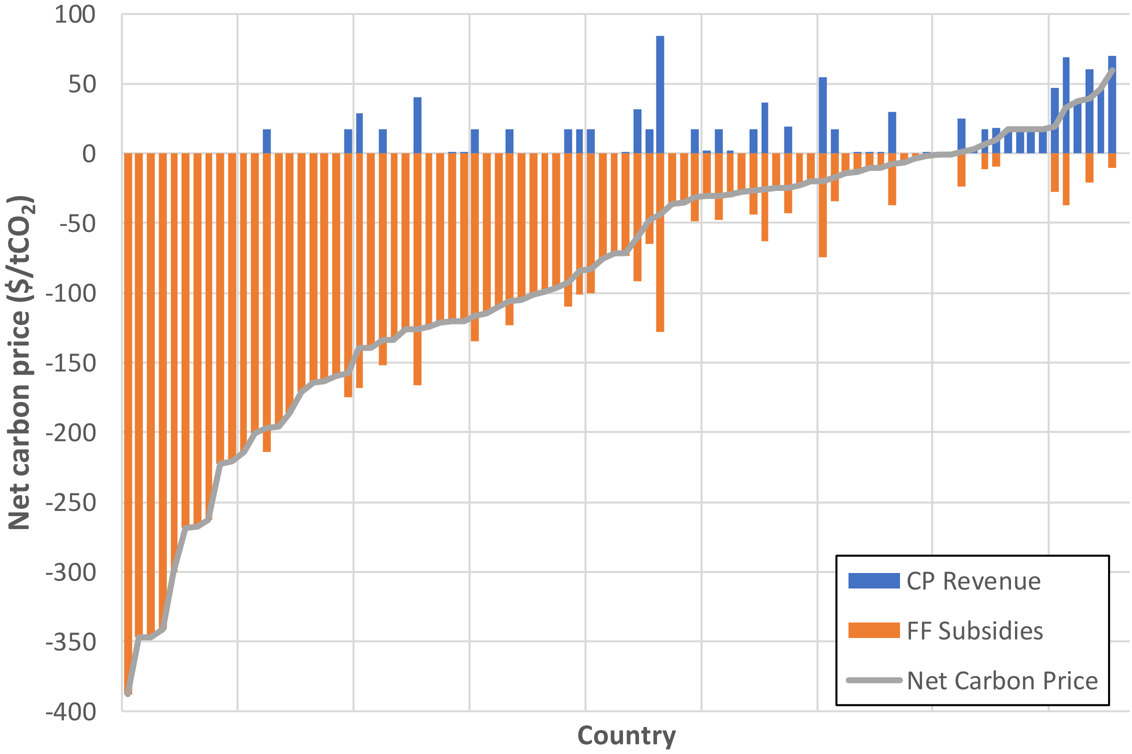

Figure 207: Net carbon prices in 2022 and their breakdown into carbon price revenue (CP) and fossil fuel subsidy (FF) components.

Indicator 4.3.4: fossil fuel and green sector bank lending

Indicator authors

Dr Jamie Rickman, Dr Nadia Ameli

Methods

Data for global lending to the fossil fuel and green sectors was taken from a proprietary Bloomberg dataset covering the global debt market. Fossil fuel lending is defined as being directed towards exploration, production, operation and marketing activities in oil and gas. Green Bonds and loans are self-identified by the issuer, who must declare use-of-proceeds are 100% dedicated to funding a project or activity with an environmental or sustainability-oriented goal i.e., renewables and energy efficiency, green building and infrastructure, agriculture, and forestry (reforestations, land-use), other sustainability (clean water, waste management). However, Bloomberg does not require additional reporting on e.g., project selection or the management of proceeds for the bond or loan to be labelled green. Bloomberg observes specific exclusions for green use of proceeds including but not limited to those involving coal and nuclear.

Data is provided as total loans provided and bonds underwritten per bank per year in USD by 920 banks from 2010 to 2022. The data was augmented by identifying each bank's ownership status (public or private) through Google search and verified through the Bloomberg terminal where necessary. Public banks, defined as banks that are majority owned by one or multiple government entities, were excluded from the dataset (over the reported period public banks provided 10% and 6% of finance to the fossil fuel and green sectors respectively). Private banks are defined as those with over 50% non-governmental ownership; they may or may not be publicly listed companies.

Bank-level financing

The yearly contribution of a bank to fossil fuel and green sector lending was calculated as the sum of bonds underwritten and loans provided. Both direct financing (loans) and facilitation of finance (bonds) is accounted for, as both forms of finance reflect the exposure of a bank to the given sector. Loans can be issued at corporate level or as project finance.

Bank-level analysis shows that the fossil fuel lending data is dominated by North American banks, while European banks are the biggest group in the green sector. This highlights that international finance flows of fossil fuel and green debt lead to investment decisions being taken in regions different to where the impact of the investment will be felt.

The top seven banks in the fossil fuel sector ranked by cumulative lending between 2010 and 2022 are (ordered from highest to lowest): JP Morgan, Wells Fargo, Citi, Bank of America, RBC Capital Markets, Mitsubishi UFJ and BNP Paribas. The top seven lending banks in the green sector ranked by cumulative investment between 2010 and 2022 are (ordered from highest to lowest): Credit Agricole, BNP Paribas, Bank of America, JP Morgan, HSBC, Citi, Deutsche Bank.

Data

- Bloomberg League Tables: (i) fossil fuel bonds, (ii) fossil fuel loans, (iii) green bonds and (iv) green use-of-proceed loans, accessed Feb 2024).
- NZBA signatories as of February 2024.[448]

Caveats

The main caveat of the data is that it represents only a subset of investments provided by the financial sector, namely debt provided by banks. Equity investments are not covered by the data, nor are contributions from other financial actors such as institutional investors.

In addition, the labelling of loans and bonds as 'green' is reliant on the classification by the issuer, which makes it susceptible to green washing. There is no independent verification of this classification.

Additional analysis

The share of loans provided, and bonds underwritten per bank and globally was also monitored, to show changes in banks' financing activities (figure 208 and figure 209). On average between 2010 and 2022, 71% of green sector debt is raised in the form of bonds compared to 39% of fossil fuel debt. Growth in the green debt sector is largely due to an exponential increase in the green bond market driven by investor appetite for sustainable investment. Compared to green loans, green bonds are typically larger in size and can be publicly traded, making them the preferred instrument for a wide range of investors including major institutional investors and asset managers. Conversely, the majority of fossil fuel debt is provided in the form of loans, which are preferred by fossil fuel companies as they have the advantage of speed of transaction and lower information disclosure over bonds.

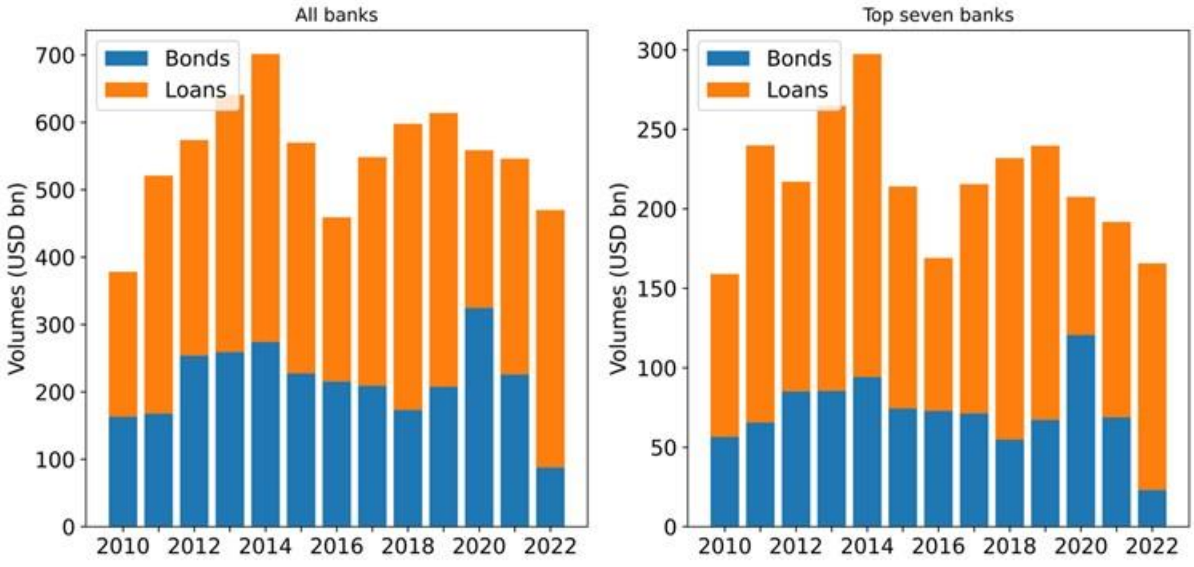

Figure 210: Financing activity of banks in the fossil fuel sector. Bar charts show volumes of bonds underwritten and loans provided by all banks (left) and the top seven banks (right).

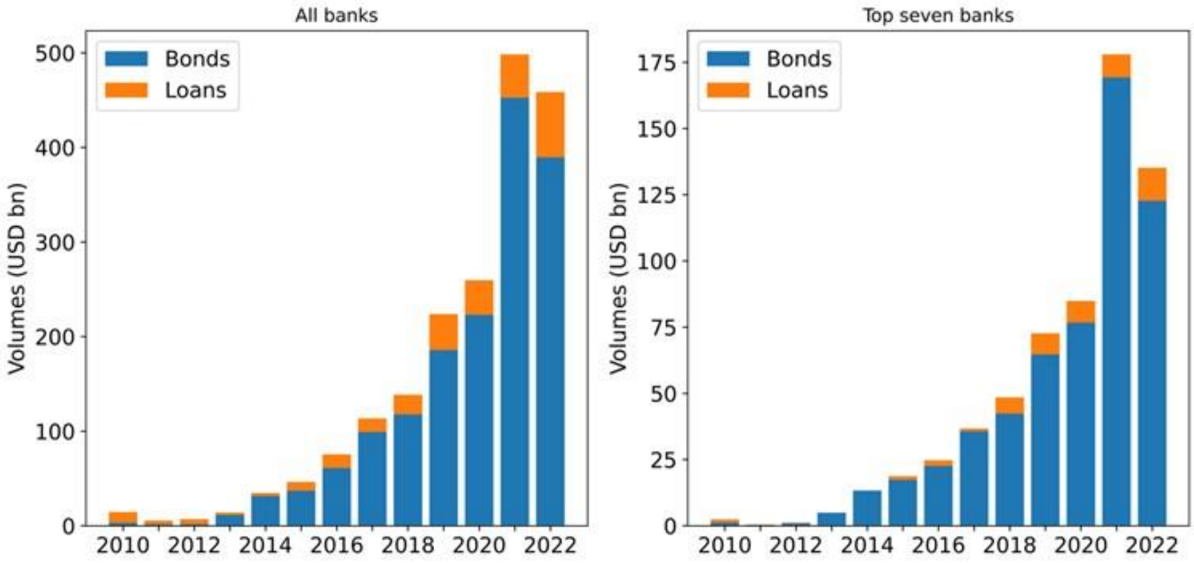

Figure 211: Financing activity of banks in the green sector. Bar charts show volumes of bonds underwritten and loans provided by all banks (left) and the top seven banks (right).

Section 5: Public and Political Engagement

Section Lead: Dr Niheer Dasandi

Indicator 5.1: Media Engagement with Health and Climate Change

Global coverage of health and climate change

Indicator authors

Dr Lucy McAllister, Dr Pete Lampard, Dr Olivia Pearman

Methods

Intersecting trends in coverage of climate change and health were identified in 65 newspaper sources from January 2007 through December 2023. The 65 sources are located across 35 countries, in four languages, and spanning the six World Health Organization (WHO) regions: African Region, Region of the Americas, South-East Asia Region, European Region, Eastern Mediterranean Region, and Western Pacific Region. These sources were monitored through Nexis Uni, Proquest and Factiva databases accessed via the University of Colorado and University of York libraries.

The 2024 report of the *Lancet* Countdown adopts the search strategy developed for the 2020 and 2021 *Lancet* Countdown reports within these three databases. The search strategy was revised for the 2020 report to increase the precision of the indicator; that is, to reduce the number of 'false positives', while retaining the maximum number of 'true positives'. This was done by retaining those terms that a) produced relevant data, and b) had a low degree of polysemy (i.e., words that have fewer meanings or words used in fewer disciplines/domains). Testing for interaction between terms also enabled fewer terms to be used (for example, it was found that the term 'morbidity' would usually pull in the term 'mortality', when related to humans).

The terms were translated once the strategy had been finalised with certain terms presenting difficulties in translation. The English terms 'hay-fever' and 'West Nile', for example, correlated with more than one term in Spanish and Portuguese and the decision was made to include all relevant terms in the respective search strategies.

For the final strategy, search functions were compared across databases to ensure consistency, as different databases utilise different search filter operators. The searches were conducted with the following key words in English, Spanish, Portuguese and German respectively:

- **English**: (climate change OR global warming) AND (health OR illness OR epidemiolog* OR malnutrition OR morbidity OR fatalit* OR diarrh* OR malaria OR chikungunya OR west nile OR dengue OR hay-fever OR zika)
- **German**: (Klimawandel OR Globale Erwärmung) AND (Gesundheit OR Krankheit OR Epidemiolog* OR Mangelernährung OR Morbidität OR Sterblich* OR Durchfall* OR Malaria OR Chikungunya OR West-Nil-Virus OR Dengue-Fieber OR Heuschnupfen OR Zika)
- **Portuguese**: (mudanças climáticas OR aquecimento global) AND (saúde OR doença OR epidemiologi* OR desnutrição OR morbilidade OR fatalidade* OR diarr* OR malária OR chikungunya OR nilo do oeste OR vírus do nilo OR dengue OR febre dos fenos OR rinite alérgica OR zika)

- **Spanish**: (cambio climático OR calentamiento global) AND (salud OR enfermedad* OR epidemiología OR epidemiólog* OR desnutrición OR malnutrición OR morbosidad OR muert* OR diarrea* OR malaria OR paludismo OR chikungunya OR nilo del oeste OR nilo occidental OR virus del nilo OR dengue OR fiebre del heno OR rinitis alérgica OR zika)

The signal of the search strategies above was found to be strong enough (over 80% relevance in a systematically randomised sample of 500) to allow a more parsimonious approach to this indicator, requiring no screening of articles during the extraction of the data.

A separate search was undertaken with the inclusion of fossil fuel terms (fossil fuel* OR gas* OR oil), in order to locate where health keywords, climate change keywords, and fossil fuel keywords were included. This was undertaken in all sources, in both 2016 (given its proximity to the Paris Agreement) and 2023.

Results were obtained from the databases by entering the relevant search strategy along with the relevant date. Counting occurred month by month and the number of returns for each source was recorded on a Microsoft Excel spreadsheet. Primary counting took place for each source along with a secondary independent count of a systematically randomised 20% sample by another researcher. Tertiary counts were undertaken where any mismatch occurred between primary and secondary counts. All counts were agreed by the whole research team.

Using the Excel spreadsheet constructed through the phases of counting, the data was organised in numerous ways for a better understanding of the patterns in coverage. These included by WHO region, by the most recent (2020) Human Development Index categories, by individual source, and now by new *Lancet* Countdown groupings. The average scores for each month (and aggregated into annual averages) were used as an adjustment for the number of sources selected per region or index category.

Data

- Three databases were used for the searches: Nexis Uni; Proquest; and Factiva databases accessed via the University of Colorado libraries.
- The 65 newspaper sources are located across 35 countries, in four languages, and spanning the six World Health Organization (WHO) regions.

Caveats

In developing the search strategy for the 2020 and 2021 *Lancet* Countdown reports it was found that a significant portion of articles may mention both climate change and health but do not engage with them as integrated issues. Including this coverage remains important as it brings both sets of issues – health and climate change – onto the public agenda and into public awareness.

Future form of the indicator

The 2025 report will look to diversify its sources to integrate more from countries in the low and medium HDI groups.

Additional analysis

As demonstrated in figure 212 absolute coverage of climate change in the media sources searched dropped by 6% from 2022 to 2023 (from 57279 to 53867 articles mentioning climate change) continuing a trend from its highest point in 2021 (68381). Co-coverage of climate change and health also declined for a second successive year, from 14134 in 2022 to 12658 in 2023: a drop of 10%. Despite the decline in both climate change and health and climate change articles from 2022 to 2023, a large increase can be observed from 2016 to 2023. The number of articles mentioning climate change increased by 71% from 2016 (31457) to 2023 (53867). The number of articles mentioning both health and climate change increased by 132% from 2016 (5447) to 2023 (12658).

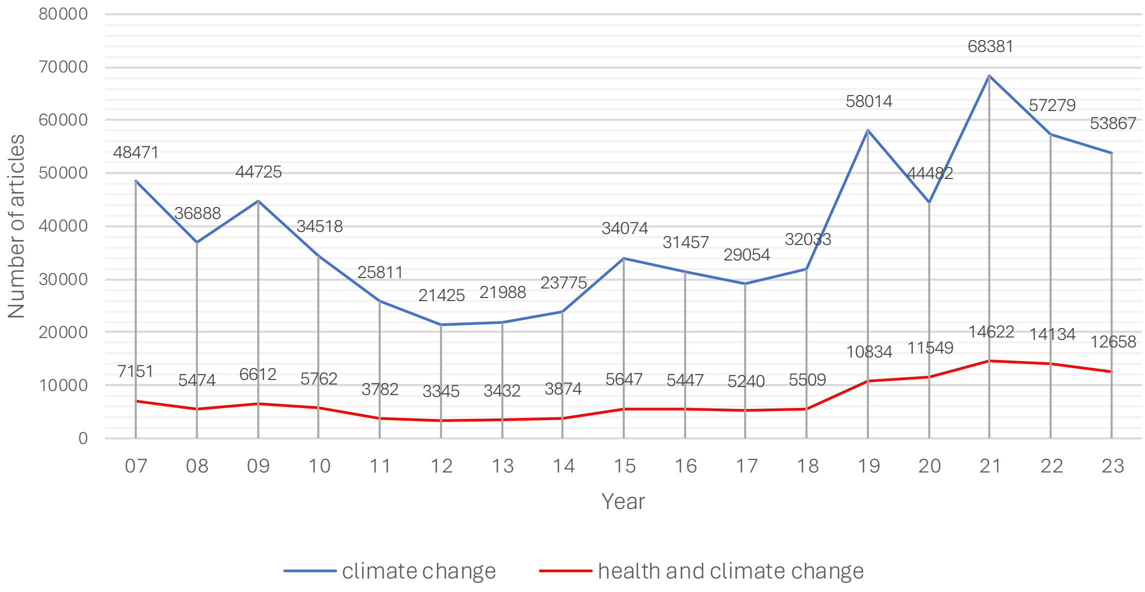

Figure 213: Number of articles mentioning climate change keywords and number mentioning both health and climate change keywords, from 2007 to 2023.

The proportion of climate change-related articles also mentioning health from 2022 to 2023 decreased from 25% to 23%. From the 2016 benchmark to 2023, the proportion increases from 17% to 23%.

Lancet Countdown, HDI Classifications, and WHO Region

When organised by *Lancet* Countdown groupings, as demonstrated in figure 214, the order of average contribution level (average across number of sources per grouping) remains the same from 2022 to 2023, with the SIDS contributing most, followed by Northern America, Europe, Oceania, Asia, South and Central America and then Africa. From 2022 to 2023, only two groupings observe increases in average coverage of health and climate change: the South and Central America (119 to 131 articles on average; 10%) and Asia (135 to 140; 4%). The other groupings all have decreases in coverage, with the Oceania grouping (273 to 176; -36%) demonstrating the largest average drop.

All groupings see large percentage increases in average numbers of articles mentioning health and climate change from 2016 to 2023. Though with smaller absolute numbers, by far the largest percentage increase is in the Africa grouping, with an increase of 651% (from 83 articles on average in 2016 to 596 in 2023). This is followed by Oceania (+198%, 92 to 176), Northern America (+195%, 180 to 434), and South and Central America (+178%, 43 to 131). Africa demonstrate the smallest percentage increase over this period (+97%, 24 to 34).

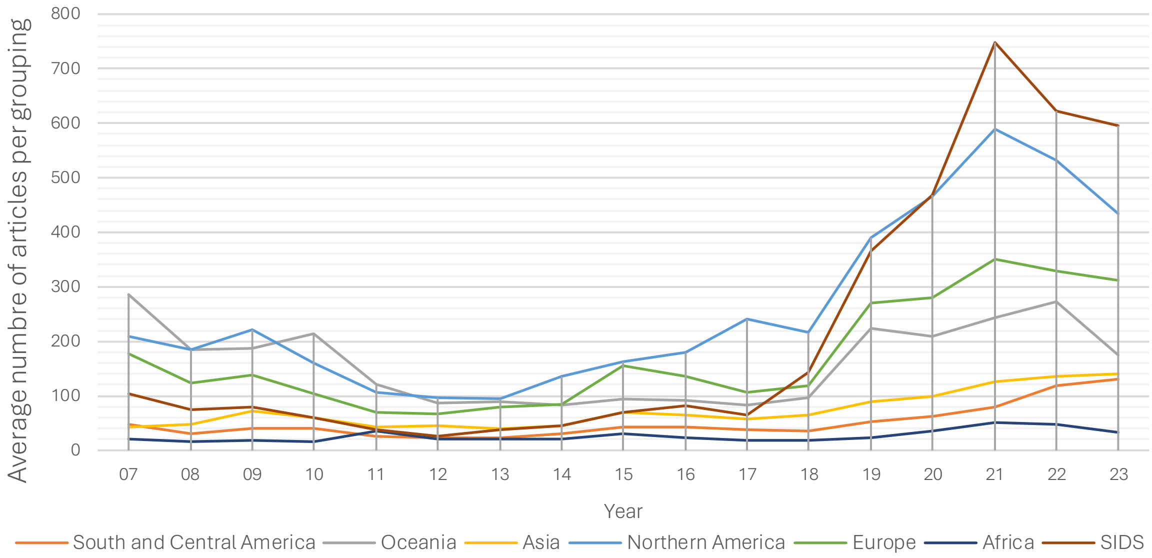

Figure 215: Average number of articles mentioning both health and climate change keywords by Lancet Countdown grouping, from 2007 to 2023.

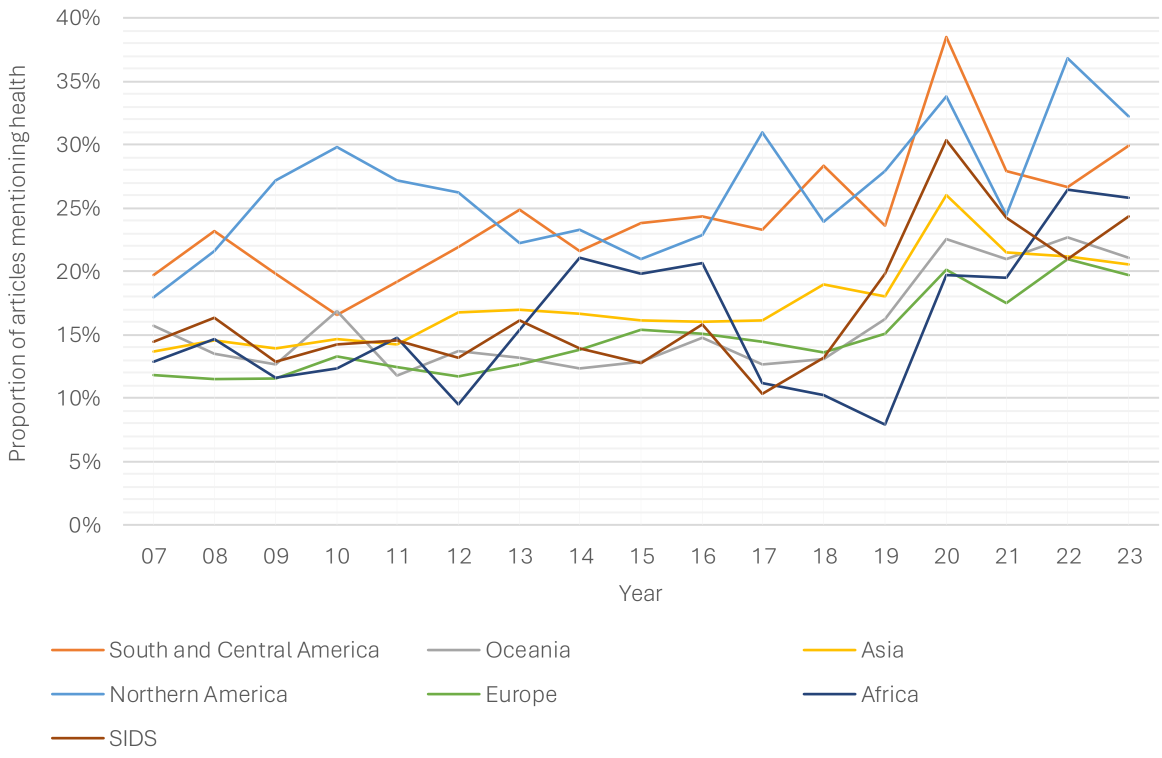

Figure 216: Proportion of climate change-related articles mentioning health by Lancet Countdown grouping, from 2007 to 2023.

Figure 217 shows the proportion of climate change articles each year within *Lancet* Countdown groupings that also mention health. The South and Central America grouping tends to have a higher proportion of articles also mentioning health, though in 2023 it is the Northern America grouping that

has the highest proportion (32%, or 3040 of 9440). The South and Central America grouping sits very slightly below (30%, or 1568 of 5236).

When organised by HDI classification, as demonstrated in Figure 218, the order of average contribution level changes, with countries in the medium HDI range moving beyond countries in the low HDI range. Though down compared to 2022 (293 average number of articles across sources), the average co-coverage of health and climate change in the very high HDI range remains higher than other HDI classifications in 2023 (249, -15%). Countries in the low HDI range also drop in co-coverage of health and climate change, from 209 in 2022 to 180 in 2023 (-14%). Increases in average coverage, however, can be seen in the medium (+5%, 166 to 180 articles) and high (+16%, 90 to 104) HDI ranges from 2022 to 2023.

All classifications see large percentage increases in average numbers of articles mentioning health and climate change from 2016 to 2023. The largest percentage increase is in the high HDI range, with an increase of 148% (from 38 articles on average in 2016 to 104 in 2023). This is followed by countries in the very high HDI range (+139%, 102 to 249), and the low HDI range (+134%, 77 to 180). Countries in the medium HDI range demonstrate the smallest percentage increase over this period (+95%, 97 to 190).

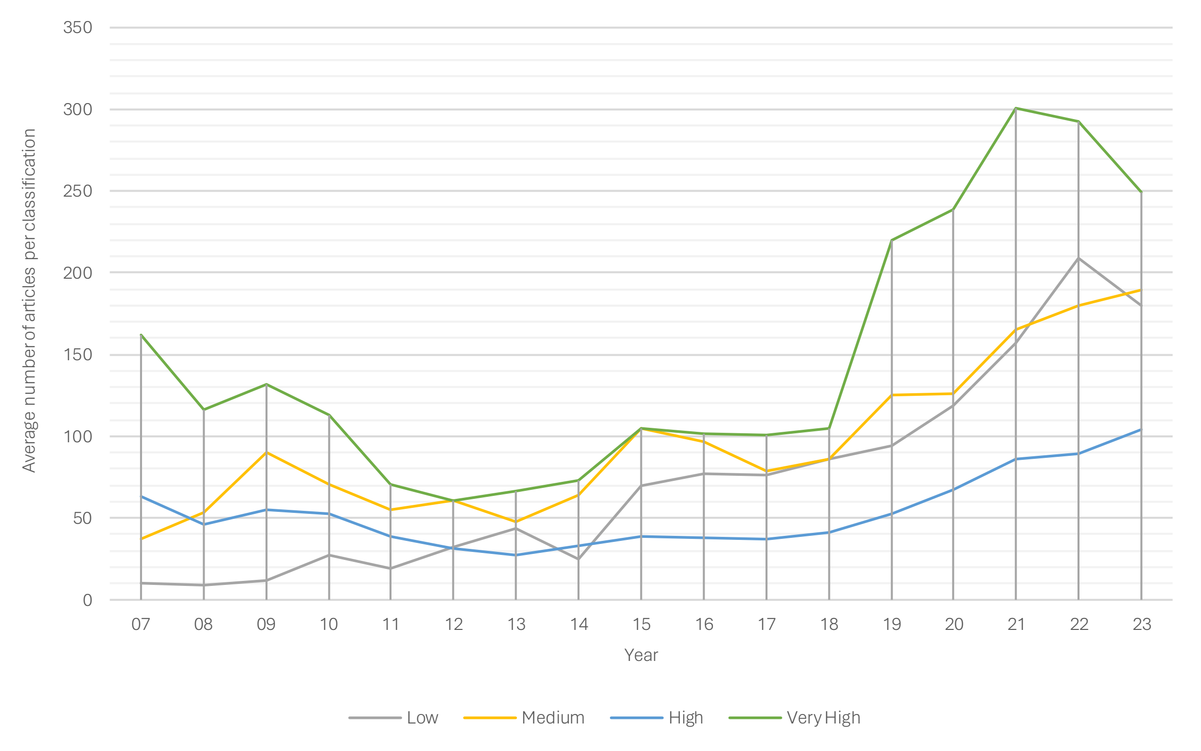

Figure 219: Average number of articles mentioning both health and climate change keywords by HDI classification, from 2007 to 2023.

Figure 220 shows the proportion of climate change articles each year within HDI classifications that also mention health. In 2023, countries within the high (26%, 104 of 407) and the very high (24%, 249 of 1041) HDI range had the highest proportion of articles also mentioning health. However, countries within the low and medium HDI classification range followed closely behind on 22% (180 of 801) and 20% (190 of 932), respectively.

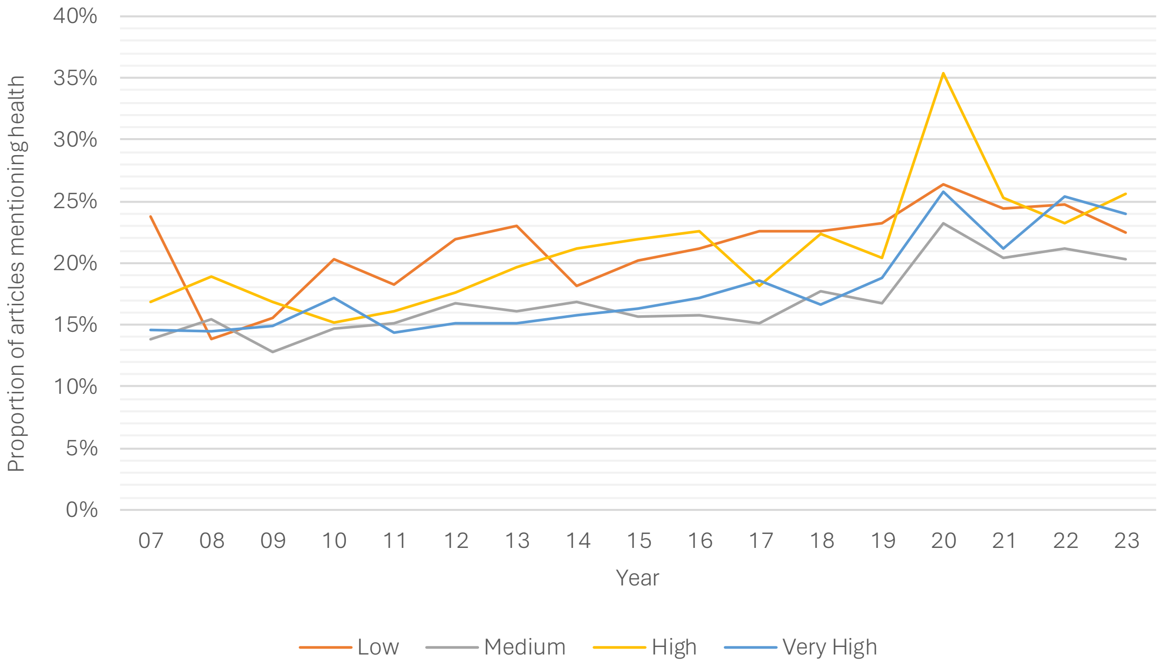

Figure 221: Proportion of climate change-related articles also mentioning health by HDI classification, from 2007 to 2023.

When organised by WHO region, as demonstrated in figure 222, the order of average contribution level changes, with coverage in the South-East Asia region moving beyond that of the Western Pacific. The South-East Asia region is the only region to demonstrate increasing coverage from 2022 to 2023, with a percentage change of 16% (from 172 articles per source in 2022 to 199 in 2023). All others see negative percentage changes, with Africa (-29%, 47 to 34) and the Western Pacific (24%, 232 to 176) with the biggest drop in average coverage across sources.

All regions see percentage increases in average numbers of articles mentioning health and climate change from 2016 to 2023. The largest percentage increase is in the region of the Americas, with an increase of 159% (from 93 articles on average per source 2016 to 243 in 2023). This is followed by countries in the South-East Asia region (+130%, 86 to 199), the Western Pacific (+128%, 77 to 176), and the European region (+115%, 127 to 295). Countries in the African region (+40%, 24 to 34) and the Eastern Mediterranean region (+85%, 37 to 102) demonstrate the smallest percentage increase over this period.

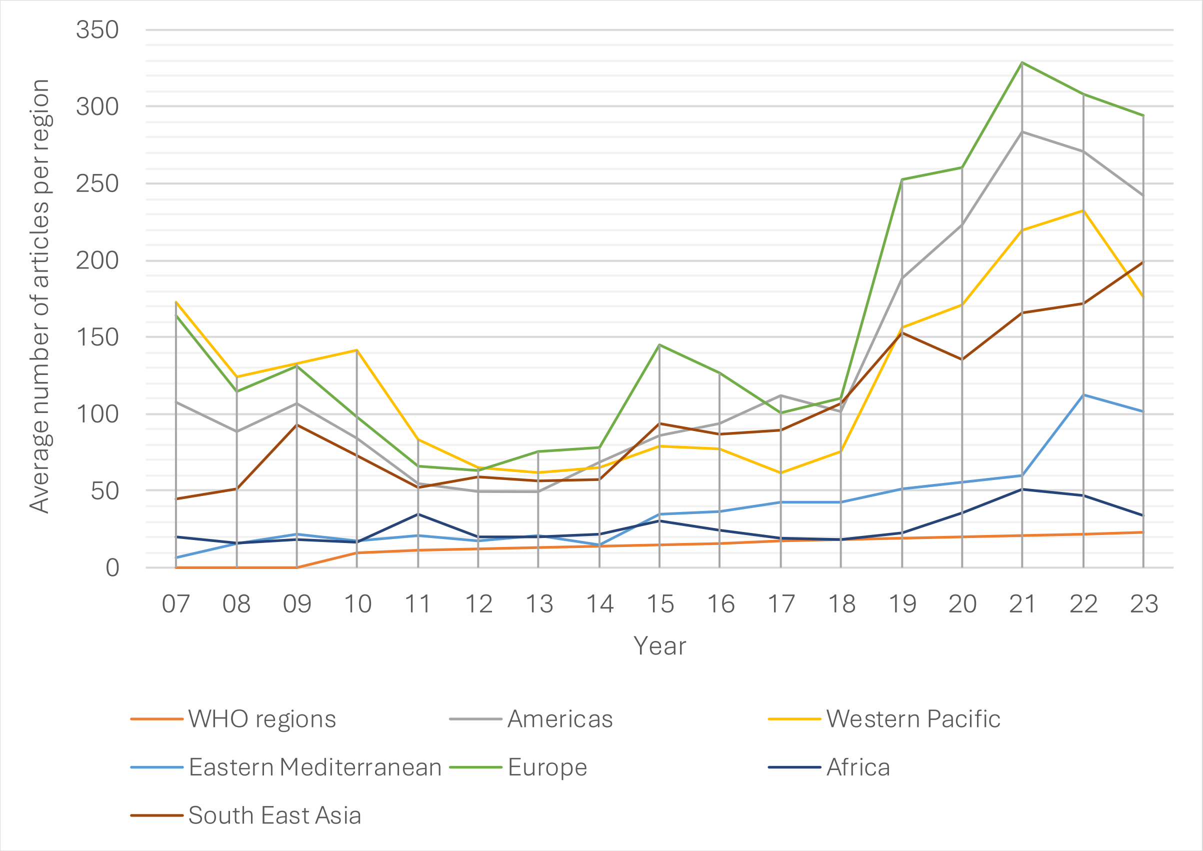

Figure 223: Average number of articles mentioning both health and climate change keywords by WHO region, from 2007 to 2023.

Health, climate change, and fossil fuels

The analysis also explored co-coverage of health, climate change, and fossil fuel terms across two different years – 2016 and 2023. Figure 224 shows the monthly differences between the two years and demonstrates a clear increase from 2016 to 2023. In overall absolute terms, the total number of articles in 2023 (3859) shows a percentage increase of 113% from 2016 (1814).

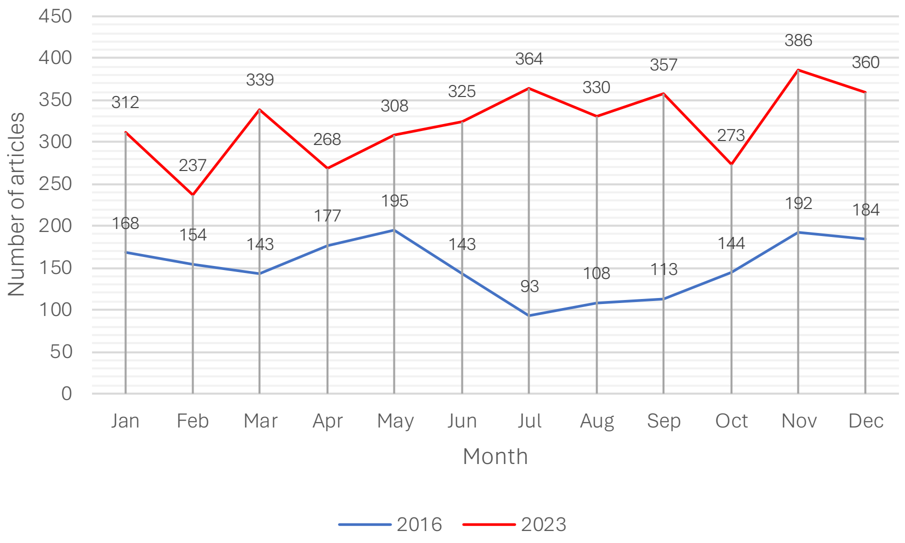

Figure 225: Total number of articles mentioning health, climate change, and fossil fuel keywords across both 2016 and 2023.

Media coverage of health and climate change in China's People's Daily

Indicator authors

Prof Wenjia Cai

Method

In the 2024 *Lancet* Countdown Report, the methodology is first trawling all articles and then searching the keywords in the text with the filtration process by score and keywords ratio as filtration criteria. The detailed steps of the method used in 2024 are shown as below:

Step 1 Crawling all the articles in 2023
All articles published in "People's Daily" in 2023 were crawled (http://paper.people.com.cn/rmrb/html/2024-02/21/nbs.D110000renmrb_01.htm)

Step 2 Searching for "Climate Change" topic articles
Articles were searched that contain the keywords in the topic of "Climate Change". The keywords are presented in the first column of table *128.*

Step 3 Identifying articles that have both climate change and health keywords (first-round search)
In this step, the first-round filtration aims to identify articles that have both climate change and health keywords. The results are the basis for the second-round search in step 4.

Step 4 Machine filtration on the results from step 3 by score and ratio (second-round search)
The articles obtained from step 3 were first scored based on the times of appearance of the keywords shown in the articles. For example, if the keywords of climate change and health have appeared 12 times in one article, then the score for this article is 12. If the keyword found is one of the "mis-hit words" (a "mis-hit words" is defined as the phrase that contains a keyword but with different meaning), the appearance will not be counted as one score.

At the same time, the ratio of times of appearance of the keywords to the total number of characters in the article (short for "the ratio" thereafter) was also calculated. When the score and the ratio of one article are both higher than the manually set thresholds, the article will be considered as relevant articles for health and climate change.

The threshold of score for each article is set to be 10, meaning the times of appearance of the keywords from both climate change and health in one article should be no less than 10. The threshold of ratio for each article is set to be no less than 1%, meaning in every 100 characters in the article, there should be no less than 1 keyword.

If the two thresholds are set too low, it would increase the workload of manual screening and increase the "false rate" of machine filtration. And if the two thresholds are set too high, it would possibly exclude the "true" articles. After several trial tests, the thresholds for score and the ratio are better set as no less than 10 and 1% respectively.

Table 129: Chinese keywords for the search in People's Daily

气候变化关键词	气候变化二级关键词	健康关键词	剔除词
气候变化	霾	疟疾	口蹄疫
全球变暖	空气污染	腹泻	黑烂病
温室	大气污染	感染	珊瑚死亡
极端天气		肺炎	沙虫死亡
全球环境变化		流行病	高温加热
低碳		公共卫生	低碳水
可再生能源		卫生	健康发展
碳排放		发病	生态健康
二氧化碳排放		营养	河流健康
气候污染		精神障碍	生态环境健康
气候		发育	
全球升温		传染	
再生能源		疾患	
CO2排放		症	
污染		瘟疫	
极端气候		流感	
高温		流行感冒	
变暖		治疗	
排放		保健	
环境变化		健康	
升温		死亡	
全球温升		精神疾病	
热浪		精神病	
暴雨		登革热	
气温		饥饿	

洪水		粮食	
洪灾		有害	
气候反常		皮肤病	
野火		风湿	
山火		呼吸系统疾病	
雪灾		人类健康	
低温		人体健康	
年代际		身体健康	
冰雪		心脏病	
可持续发展		糖尿病	
海洋酸化		疾病	
静稳		热死	
温室气体		口罩	
寒潮		防护	
强降雪			
暴雪			
台风			
干旱			
水灾			
极端降雨			
冻害			

Table 130: English translation of the Chinese keywords

**Keywords of "Climate Change"**	**Sub- level keywords of "Climate Change"**	**Keywords of "Health"**	**Removal words**
Climate change	Haze	Malaria	Aftosa
Global worming	Air pollution	Diarrhea	Black shank
Greenhouse	Atmospheric Pollution	Infected	Coral death
Extreme weather		Pneumonia	Sandworm death
Global environment change		Epidemic	Heating to higher temperature
Low carbon		Public health	Low carbohydrate
Carbon dioxide emissions		Hygiene	Healthy development
Renewable energy		Disease outbreak	Ecological health
Carbon Production		Nutrition	River health
Air pollution		Mental disorders	Eco-environmental health
Climate		Growth	
Global worming		Infection	
Renewable energy		Affection	

CO2 emissions		Symptom	
Extreme weather		Flu	
High temperature		Influenza	
Warming		Treatment	
Emission		Health care	
Environmental change		Health	
Warming		Death	
Global warming		Mental disease	
Heat wave		Mental illness	
Rainstorm		Dengue	
Temperature		Hunger	
Flood		Food	
Flood		Harmful	
Abnormal weather		Skin disease	
Wildfire		Rheumatism	
Mountain fire		Respiratory diseases	
Snowstorm		Human health	
Low temperature		Body health	
Interdecadal		Heart disease	
Ice and snow		Diabetes	
Sustainable development		Illnesses	
Ocean acidification		Heat death	
Stagnant		Mask	
Greenhouse gas		Protection	
Cold wave		Survive	
Heavy snowfall			
Blizzard			
Typhoon			
Drought			
Flood			
Extreme rainfall			
Frost damage			

Step 5: Manual screening of the results after machine filtration.
The fifth step was manually screening the filtered articles. If the manual screening confirmed that the topic is Health and Climate Change, it is retained. Titles of the 15 positive articles were presented in the additional information as table 131.

The articles obtained from step 4 were also manually screened to select the articles that contain the keywords in the topic of "fossil fuels", like oil, gas, and coal. The additional search terms based on 'fossil fuels' reveal the public concern about the health harms of fossil fuels in 2008–2023. Titles of fossil fuels coverage in health and climate change articles were presented in the additional information as table 132.

Table 133: Title of the health and climate change articles in People's Daily

文章名字	Titles of the article
智利全力抗击森林火灾	Chile makes full efforts to combat forest fires
龙卷风袭击美国多州	Tornadoes hit multiple states in the United States
采取紧急气候行动确保可持续的未来（国际视点）	Taking emergency climate action to ensure a sustainable future (International Perspective)
以更有力行动应对全球气候变化（国际视点）	Strengthening action to address global climate change more effectively (International Perspective)
非洲多国努力应对暴雨灾害	Several African countries struggle to cope with flood disasters
拉美多国紧急应对登革热	Latin American countries urgently address Dengue Fever
加拿大山火持续蔓延	Continued spread of wildfires in Canada
欧洲多国加紧应对极端高温	European countries intensify efforts to combat extreme heat
全球多地遭遇高温热浪天气（国际视点）	Global regions experience heatwaves (International Perspective)
提升气象预警能力更好服务生产生活（生态论苑）	Enhancing meteorological warning capabilities to better serve production and life (Ecological Forum)
东南亚国家加紧应对登革热	Southeast Asian countries step up efforts to combat Dengue Fever
多一分体谅生一分清凉（暖闻热评）	Showing understanding and providing relief (Warm Stories and Hot Comments）
夏威夷野火遇难人数升至80人	Hawaii wildfire death toll rises to 80 people
从自身做起，积极应对气候变化导致的过敏（名医讲堂）	Taking action from ourselves: actively addressing allergies caused by climate change (Medical Lecture Hall)

Table 134: Title of fossil fuels coverage in health and climate change articles

年份	文章名字	Titles of the article
2008	经济大国能源安全和气候变化领导人会议在日本举行	Conference of Economic Powers on Energy Security and Climate Change Held in Japan
	中国应对气候变化的政策与行动	China's Policies and Actions in Addressing Climate Change
2009	以人为本保护大气	Putting People First to Protect the Atmosphere
	积极应对全球气候变暖	Actively Addressing Global Climate Warming
	全球目光投向哥本哈根（国际视点）	Global Attention Shifts to Copenhagen (International Perspective)
2010	“十一五”能耗降20%可望实现（热点解读）	Expectations for Achieving a 20% Reduction in Energy Consumption during the Eleventh Five-Year Plan Period (Hot Topic Analysis)
	联合国气候变化坎昆会议（第一现场）	United Nations Climate Change Cancun Conference (Firsthand Account)
2015	维护气候安全保障生态文明	Safeguarding Climate Security to Ensure Ecological Civilization
2016	非洲空气污染呈加重态势	Worsening Trend of Air Pollution in Africa
2018	应对气候变暖还需持续攻坚（绿色焦点）	Continuous Efforts Needed to Combat Climate Warming (Green Focus)
2021	加强国际合作，共同应对气候变化（国际视点）	Strengthening International Cooperation to Address Climate Change (International Perspective)
	积极采取行动应对气候变化（国际视点）	Taking Active Measures to Address Climate Change (International Perspective)
	各国采取气候行动确保疫情后绿色复苏	Countries Taking Climate Actions to Ensure Green Recovery Post-Pandemic
2023	采取紧急气候行动确保可持续的未来（国际视点）	Taking Emergency Climate Actions to Ensure a Sustainable Future (International Perspective)

Data

- All the articles from 2008 to the present published on People’s Daily from the official website of People’s Daily: http://paper.people.com.cn/rmrb/html/2024-02/21/nbs.D110000renmrb_01.htm.

Additional analysis

In figure 226 the number of health and climate change coverage articles in 2023 both increased before and after manual screening. Before manually screening, there were 95 articles. 15 articles (16%) were truly related to the topic identified by manual screening, which experienced a dramatic increase. The results indicated that there were a lot false positive articles appeared during the machine filtration process in 2023. After manually checking the false positive articles, it was found that although climate related articles increased under the carbon neutrality goal, few of them focused on the topic of health.

And there are lots of articles reporting latest weather condition, causing the misleading during the filtration process.

The orange line in figure 227 shows the number of articles related to fossil fuels in the topic of climate and health. In figure 228, the number of fossil fuels coverage articles in 2023 increased compared to 2022. There were 1 out of 15 articles (6.7%) considering the harm of non-renewable energy.

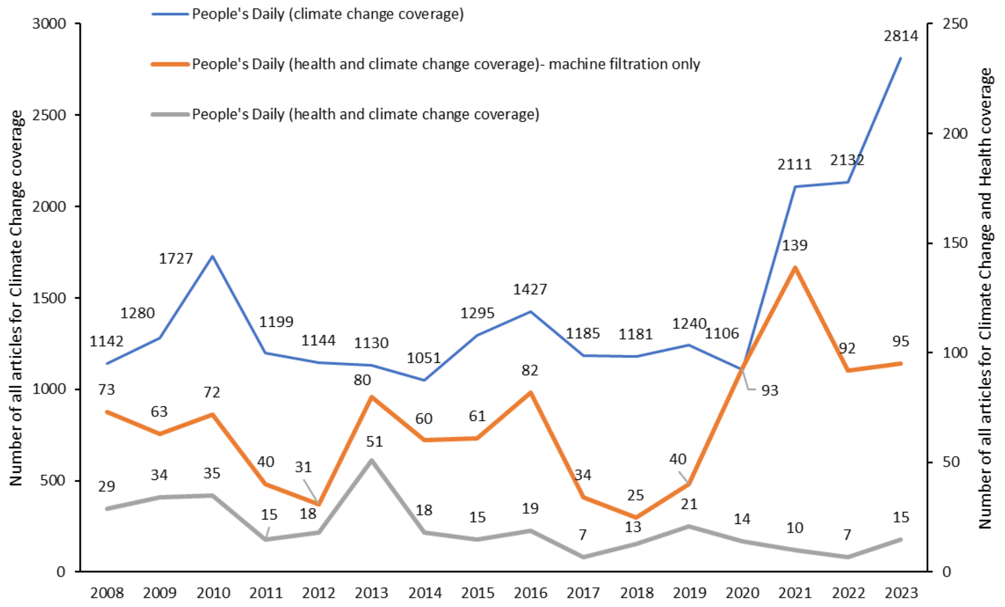

Figure 229: Numbers of all articles for climate change only (blue line), for both health and climate change after machine filtration only (orange line), and for both health and climate change after machine filtration and manual screening (grey line)

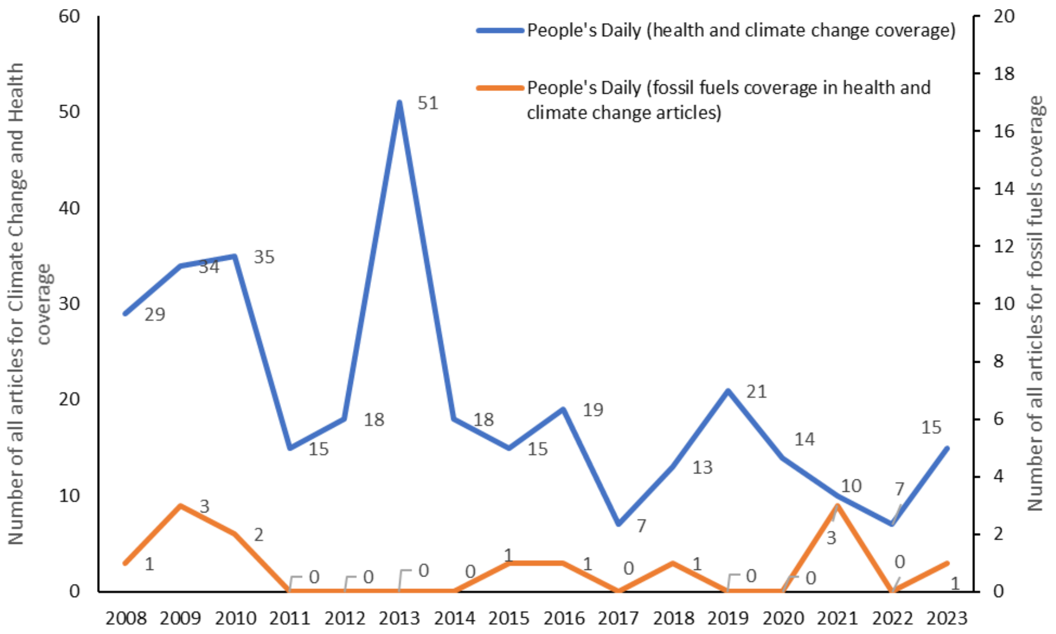

Figure 230: Numbers of articles for climate change and health (blue line), for fossil fuels in the topic of health and climate change (orange line)

Indicator 5.2: individual engagement with health and climate change

Indicator authors

Prof Simon Munzert

Method

This indicator provides an individual-level indicator of public engagement. It tracks engagement with climate change and health through people's usage of the online encyclopedia Wikipedia. Over the years, Wikipedia has grown to be a major and trusted source of information that has outpaced traditional encyclopedias in terms of reach, coverage, and comprehensiveness.[449] It is regularly listed among the ten most-visited websites worldwide.[450] The English edition covers more than 6.7 million articles and over 40,000 active editors. People around the world use it to engage in topics they are interested in. Fortunately, the traffic that goes to Wikipedia – and even that which goes to individual articles of the encyclopedia – can be analysed over time because the Wikimedia foundation makes these statistics available to everyone for free. This makes it a global indicator of what people pay attention to on a daily basis. What is more – and of particular relevance in the context of this report, the platform's health content makes it one of the most frequently used resources for information on health on the internet.[451]

The indicator

To investigate to what extent people do not only pay attention to climate change and human health in isolation, but also to the connection between both, this indicator draws on *clickstream statistics* from the English Wikipedia.

Clickstream refers to a dataset provided by the Wikimedia foundation.[452] It reports "streams of clicks", or in other words: how people get to a Wikipedia article and what links they click on. This is reported

on a monthly basis and in pairs of resources; the first being where the visit came from, the second which page was visited. This gives an indicator of monthly-level global attention towards one issue (if both articles are representative of the same issue) or two issues (if articles come from different domains, such as climate change and health). By looking at climate change–health article pairs, an indicator of attention towards climate change consequences for human health over time can be generated.

Measurement strategy

Our approach to using clickstream data as an indicator of public engagement in climate change and health is based on the following premises:

- The Wikipedia platform is a globally used source for information on a multitude of topics. (See https://stats.wikimedia.org/wikimedia/squids/SquidReportPageViewsPerCountryOverview.htm for an overview of Wikipedia usage by country and languages)
- Citizens use the platform to inform themselves about topics they are interested in
- By tracking engagement with Wikipedia articles that are related to climate change as well as with articles on health, it is possible to identify public engagement with the relationship between both topics

The following behavioural patterns are relevant for the validity of the measure as a proxy for public engagement with climate change and health:

- A person is generally interested in the nexus between climate change and public health and informs her/himself about the topic online by, e.g., reading the Wikipedia article on Effects of climate change on human health (https://en.wikipedia.org/wiki/Effects_of_climate_change_on_human_health)
- A person is interested in climate change and the consumption of information about the topic, this then sparks interest in its consequences for human health. For instance, the person reads the article on Climate change (https://en.wikipedia.org/wiki/Climate_change) and then turns to the article on Malnutrition (https://en.wikipedia.org/wiki/Malnutrition)
- A person is interested in a certain aspect of human health or consequences of climate change with an immediate impact on human health, and then turns attention to climate change issues. For instance, the person reads the article on Malaria (https://en.wikipedia.org/wiki/Malaria) and then turns to the article on climate change (https://en.wikipedia.org/wiki/Climate_change).

Indicator construction

In order to use the Wikipedia viewership statistics as a proxy for public engagement with climate change and health, it is key to select articles that are representative of these topics. To generate the populations of articles related to climate change on the one hand and health on the other, a semi-automated approach is implemented. Based on an initial set of keywords, a search was undertaken for related articles using the internal Wikipedia search.

Keywords

For climate change articles, the keywords were:

carbon dioxide, carbon emission, carbon neutral, carbon neutrality, carbon-dioxide, carbon-neutral, changing climate, climat, climate action, climate change, climate crisis, climate decay, climate emergency, climate neutrality, climate pollutant, climate variability, co2, co2 emission, decarbonisation, decarbonization, extreme temperature, extreme weather, ghge, ghges, glacial, global environmental

change, global warming, green house, green new, greenhouse, greenhouse-gas, ipcc, low carbon, net zero, net-zero, ozone, renewable energy, sea ice, sea level, sphere, temperature record.

For health articles, the seed keywords were:

air pollution, asthma, cancer, communicable disease, diagnosis, diarrhoea, disease, diseases, disorder, epidemic, epidemics, epidemiolog, epidemiology, epidemy, fever, health, health care, healthcare, hunger, icide, illness, illnesses, infection, infectious, itis, malaria, malnourishment, malnutrition, measles, mental disorder, mental disorders, morbidity, mortality, ncd, ncds, non-communicable disease, noncommunicable disease, nutrition, nutrition, osis, pandemic, pandemics, pediatric, pneumonia, psychiatric, public health, sars, stunting, syndrome.

Article processing

For each search using one of the keywords, the first 100 results were extracted and identified that led to an article with a minimum word count of 300, ensuring that the articles that were chosen as seed articles had been given a certain degree of attention by Wikipedia editors, therefore being more likely to link to other relevant articles.

Next, the articles collected via the Wikipedia search for categories were screened, which are used on the Wikipedia to categorise pages in a meaningful way (e.g., using categories such as Climate change or Effects of climate_change). Those categories were then themselves screened for relevant articles. All additional articles were once more filtered such that those with a title matching one of the initial keywords was chosen. For the health-related articles, several articles were excluded manually after they turned out to be irrelevant for the purposes of this indicator. Health topics are covered extensively on Wikipedia, but a decision was made to prioritise articles and topics that, in principle, can be related to climate change. In addition, the fact that the Wikipedia page on the effects of climate change on human health offers a variety of links to further health-related articles was exploited. This is seen as a curated list of relevant health articles and added those links to the list. The complete list of articles is recorded under Additional Information.

For the clickstream analysis, the set of articles was extended by also taking “second-level pages” into account, that is, pages that are linked to in the initially identified set of climate change or health articles and that are also somewhat related to climate change or health. Sometimes, people might not directly jump from one of the major articles on climate change to another one on health, but travel through an intermediary page (e.g., a possible individual stream of clicks could be: Climate change > Human impact on the environment > Respiratory disease). The clickstream data only allows the identification of click volume for pairs of articles, but by extending the network, it is possible to also capture clickstreams involving relevant pages that are linked in the original set of articles.

Technically, the fact that the population of health articles is far larger than the population of climate change articles does not invalidate the measurement strategy. It seems plausible that there are much more articles on health-related than on climate change-related topics because the health field is so much broader (which is one reason why the health articles cluster in the network plot is not that dense – some health topics are really far apart from each other, although both could be covering health issues that are affected by climate change). But this should not directly affect the metrics. Even if there are many more health than climate change articles, it could still be that health topics are mentioned (and clicked on) much more often in climate change articles than the other way around.

What is key in the analysis is not that one or the other topic is more extensively covered on the platform, but the co-visit patterns.

Data

The indicator draws on publicly available data from the Wikimedia foundation. It considers data from all platforms, i.e., accesses to the Wikipedia via desktop machines, mobile browsers, and mobile apps.

The clickstream data were downloaded from the Wikimedia Dumps (https://dumps.wikimedia.org/other/clickstream/). Spider traffic (i.e., traffic generated by automated bots crawling the platform) is excluded. Referrer-resource pairs (i.e., the pairs of the article of origin and the target article) that had less than 10 clicks were removed in the original dataset, so there is an expectation that the indicator will slightly underreport the actual clickstream traffic. However, it is not expected that this will add any systematic bias to the indicators in particular since the interest lies mainly in changes of engagement over time.

Clickstream data are available from November 2017 onwards. In this report, data is used from 2018 to 2023. The analyses are limited to the English Wikipedia.

The benefits of the Wikipedia usage metadata for the purpose of tracking public engagement in climate change and health are that these data (a) are globally available, (b) cover the time period of interest, (c) are collectible at virtually no cost, and, most importantly, (d) have high face validity to measure engagement in this very specific topic. Reading articles on Wikipedia is motivated by attention towards a particular issue. Individuals invest time to inform themselves about a topic, which is one manifestation of engagement. Aggregate reading behaviour can therefore be seen as an *a priori* valid approximation of public issue engagement.

Caveats

All clickstream information is only available at the aggregate level. It is not possible to link the data to information about individuals who visited the platform. Also, the data are not geo-referenced, so it is not possible to infer where page visits came from. Although the English Wikipedia is predominantly used in English-speaking countries (according to the Wikimedia Traffic Analysis Report, about 40% of the traffic on the English Wikipedia comes from the United States), it is a globally popular resource. It makes up for 50% of the global traffic to all Wikipedia language editions. Therefore, it can be seen as a global indicator of public attention that is somewhat biased towards attention from countries such as the United States, United Kingdom, India, Canada, and Australia. Extending the analyses to other language editions will help to remedy this bias and uncover potential geographic engagement heterogeneity in the future.

More generally, the measure represents an online proxy for an offline phenomenon. In addition, it is sensitive towards the selection of articles used to capture engagement. The global popularity of the platform, which consistently ranks among the ten most visited websites worldwide, speaks in favour of its usefulness for this application. However, more direct indicators of public engagement, such as survey-based measures, might provide a useful supplement and source for validation in the future.

While the data are available for free, access to future data depends on the Wikimedia API. There is no indication of Wikimedia restricting access in the future. Instead, Wikimedia has invested in data quality and making access more robust and convenient.

Additional analysis

List of English Wikipedia articles used to track public engagement in climate change

2 degree climate target, 2001 United Nations Climate Change Conference, 2014 People's Climate March, 2016 United Nations Climate Change Conference, 2017 People's Climate March, 2017 United Nations Climate Change Conference, 2019 in climate change, 2020 in climate change, 2021 in climate change, 2021 Leaders Summit on Climate, 2021 United Nations Climate Change Conference, 2022 in climate change, 2022 United Nations Climate Change Conference, 2023 Berlin climate neutrality referendum, 2023 in climate change, 2023 United Nations Climate Change Conference, 4 Degrees and Beyond International Climate Conference, A Green New Deal, Abrupt climate change, Action for Climate Empowerment, Advisory Group on Greenhouse Gases, Agency for New and Renewable Energy Research and Technology, American Association of State Climatologists, American College & University Presidents' Climate Commitment, Amundsen-Nobile Climate Change Tower, Antarctic sea ice, APEC Climate Center, Arctic Climate Impact Assessment, Arctic sea ice decline, Asia-Pacific Partnership on Clean Development and Climate, Asilomar International Conference on Climate Intervention Technologies, Association for Renewable Energy and Clean Technology, Atmosphere of Earth, Attorney General of Virginia's climate science investigation, Attribution of recent climate change, Australian Greenhouse Office, Australian Renewable Energy Agency, Aventine Renewable Energy, Aviation and climate change, Avoiding Dangerous Climate Change (2005 conference), Bali Declaration by Climate Scientists, Bangladesh Climate Change Resilience Fund, Bangladesh Climate Change Trust, Bay Area Climate Collaborative, Biber-Danube interglacial, Bioclimatology, Bjerknes Centre for Climate Research, Boston Green New Deal, Boulder Climate Action Plan, Bristol Youth Strike 4 Climate, Business action on climate change, C40 Cities Climate Leadership Group, California Climate Action Registry, California Climate Credit, California Climate Executive Orders, Camp for Climate Action, Campaign against Climate Change, Canadian Youth Climate Coalition, Carbon dioxide, Carbon dioxide (data page), Carbon dioxide angiography, Carbon dioxide clathrate, Carbon dioxide cleaning, Carbon dioxide generator, Carbon dioxide in Earth's atmosphere, Carbon Dioxide Information Analysis Center, Carbon dioxide recorder, Carbon dioxide reforming, Carbon dioxide removal, Carbon dioxide scrubber, Carbon dioxide sensor, Carbon emission label, Carbon Emission Reduction Target, Carbon emission trading, Carbon Neutrality Coalition, Carbon neutrality in the United States, Carbon-dioxide laser, Carbon-neutral fuel, Center for Climate and Energy Solutions, Center for Climate and Life, Center for the Study of Carbon Dioxide and Global Change, Centre for Climate Change Economics and Policy, Centre for International Climate and Environmental Research, Centre for Renewable Energy, Centre for Renewable Energy Systems Technology, Chemosphere (journal), Chesapeake Climate Action Network, Chicago Climate Action Plan, Chicago Climate Exchange, Cities for Climate Protection program, Citizens Convention for Climate, Citizens' Climate Lobby, Civil Society Coalition on Climate Change, Climate, Climate 200, Climate action, Climate Action Network, Climate Action Network Latin America, Climate Action Tracker, Climate Alliance, Climate and Clean Air Coalition to Reduce Short-Lived Climate Pollutants, Climate and Development, Climate and Development Knowledge Network, Climate and Ecological Emergency Bill, Climate and energy, Climate and vegetation interactions in the Arctic, Climate apocalypse, Climate appraisal, Climate as complex networks, Climate Audit, Climate Capitalism, Climate Case Ireland, Climate categories in viticulture, Climate Central, Climate change, Climate Change (Scotland) Act 2009, Climate Change Accountability Act (Bill C-224), Climate change acronyms, Climate Change Act 2008, Climate change adaptation, Climate change adaptation in the Philippines, Climate change adaptation strategies on the German coast, Climate Change Agreement (UK), Climate change and agriculture in the United States, Climate change and birds, Climate change and children, Climate change and cities, Climate change and civilizational collapse, Climate Change and Emissions Management Amendment

Act, Climate Change and Energy Transition Act, Climate change and fisheries, Climate change and food security in Africa, Climate change and gender, Climate change and Indigenous peoples, Climate change and infectious diseases, Climate change and invasive species, Climate change and poverty, Climate change and society, Climate Change and Sustainable Energy Act 2006, Climate change and wildfires, Climate change art, Climate Change Authority, Climate Change Capital, Climate Change Commission, Climate Change Committee, Climate change denial, Climate Change Denial Disorder, Climate change education, Climate change ethics, Climate change in Afghanistan, Climate change in Africa, Climate change in Alabama, Climate change in Alaska, Climate change in Algeria, Climate change in American Samoa, Climate change in Antarctica, Climate change in Argentina, Climate change in Arizona, Climate change in Arkansas, Climate change in Asia, Climate change in Australia, Climate change in Austria, Climate change in Azerbaijan, Climate change in Bangladesh, Climate change in Belgium, Climate change in Bosnia and Herzegovina, Climate change in Botswana, Climate change in Brazil, Climate change in California, Climate change in Cambodia, Climate change in Canada, Climate change in China, Climate change in Colorado, Climate change in Connecticut, Climate change in Cyprus, Climate change in Delaware, Climate change in Egypt, Climate change in Eswatini, Climate change in Ethiopia, Climate change in Europe, Climate change in Fiji, Climate change in Finland, Climate change in Florida, Climate change in France, Climate change in Georgia (U.S. state), Climate change in Germany, Climate change in Ghana, Climate change in Greece, Climate change in Greenland, Climate change in Grenada, Climate change in Guam, Climate change in Guatemala, Climate change in Honduras, Climate change in Idaho, Climate change in Illinois, Climate change in India, Climate change in Indiana, Climate change in Indonesia, Climate change in Iowa, Climate change in Iran, Climate change in Iraq, Climate change in Israel, Climate change in Italy, Climate change in Japan, Climate change in Jordan, Climate change in Kansas, Climate change in Kentucky, Climate change in Kenya, Climate change in Kyrgyzstan, Climate change in Liberia, Climate change in Louisiana, Climate change in Luxembourg, Climate change in Madagascar, Climate change in Maine, Climate change in Malaysia, Climate change in Mali, Climate change in Maryland, Climate change in Massachusetts, Climate change in Mexico, Climate change in Michigan, Climate change in Minnesota, Climate change in Mississippi, Climate change in Missouri, Climate change in Mongolia, Climate change in Montana, Climate change in Morocco, Climate change in Mozambique, Climate change in music, Climate change in Myanmar, Climate change in Namibia, Climate change in Nebraska, Climate change in Nepal, Climate change in Nevada, Climate change in New Hampshire, Climate change in New Jersey, Climate change in New Mexico, Climate change in New York (state), Climate change in New York City, Climate change in New Zealand, Climate change in Nicaragua, Climate change in Nigeria, Climate change in North Carolina, Climate change in North Dakota, Climate change in North Korea, Climate change in Norway, Climate change in Ohio, Climate change in Oklahoma, Climate change in Oregon, Climate change in Pakistan, Climate change in Pennsylvania, Climate change in Poland, Climate change in popular culture, Climate change in Puerto Rico, Climate change in Rhode Island, Climate change in Russia, Climate change in Saskatchewan, Climate change in Scotland, Climate change in Senegal, Climate change in Somalia, Climate change in South Africa, Climate change in South Asia, Climate change in South Carolina, Climate change in South Dakota, Climate change in South Korea, Climate change in South Sudan, Climate change in Spain, Climate change in Sri Lanka, Climate change in Sudan, Climate change in Suriname, Climate change in Sweden, Climate change in Taiwan, Climate change in Tanzania, Climate change in Tennessee, Climate change in Thailand, Climate change in the Arctic, Climate change in the Caribbean, Climate change in the Democratic Republic of the Congo, Climate change in the Gambia, Climate change in the Maldives, Climate change in the Marshall Islands, Climate change in the Middle East and North Africa, Climate change in the Netherlands, Climate change in the Pacific Islands, Climate change in the Philippines, Climate change in the Republic of Ireland, Climate change in the United Kingdom, Climate change in

the United States, Climate change in Turkey, Climate change in Tuvalu, Climate change in Uganda, Climate change in Uruguay, Climate change in Utah, Climate change in Vermont, Climate change in Vietnam, Climate change in Virginia, Climate change in Washington, Climate change in Washington, D.C., Climate change in West Virginia, Climate change in Wisconsin, Climate change in Wyoming, Climate change in Zambia, Climate change in Zimbabwe, Climate Change Levy, Climate change litigation, Climate change mitigation, Climate change mitigation framework, Climate Change Performance Index, Climate change policy of California, Climate change policy of the George W. Bush administration, Climate change policy of the United States, Climate change protests in Australia, Climate Change Response (Emissions Trading) Amendment Act 2008, Climate Change Response (Zero Carbon) Amendment Act, Climate Change Response Act 2002, Climate change scenario, Climate Change Science Program, Climate Change TV, Climate change vulnerability, Climate change, industry and society, Climate Change: Global Risks, Challenges and Decisions, Climate classification, Climate Commission, Climate commitment, Climate communication, Climate Council, Climate crisis, Climate Crisis Advisory Group, Climate Data Analysis Tool, Climate Data Operators, Climate debt, Climate Denial Crock of the Week, Climate Disclosure Standards Board, Climate Doctrine of the Russian Federation, Climate Dynamics, Climate emergency declaration, Climate emergency declarations in Australia, Climate emergency declarations in New Zealand, Climate emergency declarations in the United Kingdom, Climate Emergency Fund, Climate engineering, Climate ensemble, Climate fiction, Climate gentrification, Climate governance, Climate Hawks Vote, Climate Hustle, Climate inertia, Climate Investment Funds, Climate justice, Climate Justice Action, Climate Justice Alliance, Climate Justice Now!, Climate Law and Governance Initiative, Climate Leadership and Community Protection Act, Climate Leadership Council, Climate migration, Climate Mirror, Climate model, Climate Monitoring and Diagnostics Laboratory, Climate movement, Climate Nexus, Climate of Agra, Climate of Albania, Climate of ancient Rome, Climate of Argentina, Climate of Armenia, Climate of Australia, Climate of Azerbaijan, Climate of Bangladesh, Climate of Barcelona, Climate of Bihar, Climate of Bilbao, Climate of Brazil, Climate of Budapest, Climate of Buenos Aires, Climate of Cameroon, Climate of Cebu, Climate of Chile, Climate of China, Climate of Colombia, Climate of Cyprus, Climate of Delhi, Climate of Dubai, Climate of East Anglia, Climate of Ecuador, Climate of Egypt, Climate of Estonia, Climate of Ethiopia, Climate of Finland, Climate of Ghana, Climate of Gibraltar, Climate of Greece, Climate of Gujarat, Climate of Himachal Pradesh, Climate of Hungary, Climate of Iceland, Climate of India, Climate of Indonesia, Climate of Ireland, Climate of Istanbul, Climate of Italy, Climate of Kaziranga National Park, Climate of Kolkata, Climate of Kosovo, Climate of Lisbon, Climate of Madrid, Climate of Malta, Climate of Manitoba, Climate of Mexico, Climate of Moscow, Climate of Mumbai, Climate of Myanmar, Climate of New England, Climate of New Zealand, Climate of Nigeria, Climate of Norway, Climate of Nova Scotia, Climate of Pakistan, Climate of Paraguay, Climate of Paris, Climate of Peru, Climate of Poland, Climate of Porto, Climate of Puerto Rico, Climate of Rajasthan, Climate of Romania, Climate of Rome, Climate of Russia, Climate of Saudi Arabia, Climate of Seoul, Climate of Serbia, Climate of Sochi, Climate of South Africa, Climate of South Brazil, Climate of Southeast Brazil, Climate of Spain, Climate of Svalbard, Climate of Sweden, Climate of Tamil Nadu, Climate of Tasmania, Climate of Thailand, Climate of the British Isles, Climate of the Falkland Islands, Climate of the Past, Climate of the Philippines, Climate of the United Kingdom, Climate of the United States, Climate of Turkey, Climate of Uruguay, Climate of Uttar Pradesh, Climate of Valencia, Climate of Venezuela, Climate of Vietnam, Climate of Wales, Climate of West Bengal, Climate of Zambia, Climate One, Climate Policy (journal), Climate Policy Initiative, Climate prediction, Climate Prediction Center, Climate psychology, Climate reparations, Climate Research (journal), Climate resilience, Climate restoration, Climate risk, Climate risk insurance, Climate risk management, Climate Savers Computing Initiative, Climate Science Legal Defense Fund, Climate Science Rapid Response Team, Climate security, Climate sensitivity, Climate Solutions Caucus,

Climate Solutions Road Tour, Climate spiral, Climate Stewardship Acts, Climate target, Climate TRACE, Climate variability and change, Climate Vulnerability Monitor, Climate Vulnerable Forum, Climate Watch, Climate Week NYC, Climate-Alliance Germany, Climate-friendly gardening, Climate-friendly school, Climate-smart agriculture, Climate: Long range Investigation, Mapping, and Prediction, Climatic Change (journal), Climatic geomorphology, Climatic regions of Argentina, Climatic Research Unit documents, Climatic Research Unit email controversy, Climatological normal, Climatology, Cloud formation and climate change, Co-benefits of climate change mitigation, CO2 (opera), CO2 Coalition, CO2 fertilization effect, CO2 is Green, CO2balance, Committee on Climate Change Science and Technology Integration, Compressed carbon dioxide energy storage, Cool It: The Skeptical Environmentalist's Guide to Global Warming, Cooperative Institute for Climate Applications and Research, Cooperative Institute for Climate Science, Copenhagen Climate Challenge, Copper in renewable energy, Criticism of the IPCC Fourth Assessment Report, Cultural heritage at risk from climate change, Danube-Gunz interglacial, Decarbonisation measures in proposed UK electricity market reform, Decarbonization of shipping, Deep Decarbonization Pathways Project, Deforestation and climate change, Dendroclimatology, Department for Energy Security and Net Zero, Department of the Environment, Climate and Communications, Description of the Medieval Warm Period and Little Ice Age in IPCC reports, Desert climate, Direct deep-sea carbon dioxide injection, Disability and climate change, Drawdown (climate), Earth rainfall climatology, East Asia Climate Partnership, Economic analysis of climate change, Economics of climate change mitigation, Economists' Statement on Climate Change, Ecosphere (social enterprise), Ed Hawkins (climatologist), Effects of climate change, Effects of climate change on agriculture, Effects of climate change on human health, Effects of climate change on livestock, Effects of climate change on mental health, Effects of climate change on oceans, Effects of climate change on plant biodiversity, Effects of climate change on small island countries, Effects of climate change on the water cycle, Effects of global warming, Effects of global warming on human health, Effects of global warming on humans, Effects of global warming on the United Arab Emirates, Electrochemical reduction of carbon dioxide, Euro-Mediterranean Center on Climate Change, European Assembly for Climate Justice, European Climate Change Programme, European Climate Exchange, European Climate Forum, European Climate Foundation, European Climate, Infrastructure and Environment Executive Agency, European Renewable Energy Council, European Union climate and energy package, Evangelical Climate Initiative, Extinction risk from climate change, Extreme weather, Extreme weather post-traumatic stress disorder, ExxonMobil climate change denial, Federal Ministry for Economic Affairs and Climate Action, Fennec (climate program), Forward on Climate, Fourth National Climate Assessment, Freedom of Information requests to the Climatic Research Unit, G8 Climate Change Roundtable, Gallery Climate Coalition, Garnaut Climate Change Review, Generation Climate Europe, Geologic temperature record, German Climate Action Plan 2050, German Climate Consortium, German Renewable Energy Sources Act, Germany National Renewable Energy Action Plan, Ghana Climate Innovation Centre, Glasgow Climate Pact, Global Atmosphere Watch, Global Climate Action (portal), Global Climate Action Summit, Global Climate and Energy Project, Global Climate and Health Alliance, Global Climate Coalition, Global Climate March, Global Climate Network, Global climate regime, Global Covenant of Mayors for Climate & Energy, Global Day of Climate Action 2020, Global Environmental Change, Global Historical Climatology Network, Global Paleoclimate Indicators, Global Roundtable on Climate Change, Global temperature record, Global warming hiatus, Global Warming Pollution Reduction Act of 2007, Global warming potential, Global Warming Solutions Act of 2006, Global Warming: The Signs and The Science, Global Warming: What You Need to Know, Glossary of climate change, Gorgon Carbon Dioxide Injection Project, Grande-Synthe climate case, Great March for Climate Action, Green New Deal, Greenhouse and icehouse Earth, Greenhouse debt, Greenhouse Development Rights, Greenhouse effect, Greenhouse gas, Greenhouse gas emissions, Greenhouse gas emissions by Australia, Greenhouse

gas emissions by China, Greenhouse gas emissions by Russia, Greenhouse gas emissions by the United Kingdom, Greenhouse gas emissions by the United States, Greenhouse gas emissions by Turkey, Greenhouse gas emissions from agriculture, Greenhouse gas emissions from wetlands, Greenhouse gas emissions in Kentucky, Greenhouse gas inventory, Greenhouse gas monitoring, Greenhouse Gas Pollution Pricing Act, Greenhouse Gases Observing Satellite, Greenhouse Gases Observing Satellite-2, Greenhouse Mafia, Greenhouse Solutions with Sustainable Energy, Ground-level ozone, Gussing Renewable Energy, High Council on Climate, High Level Advisory Group on Climate Financing, High Plains Regional Climate Center, Highest temperature recorded on Earth, Highland temperate climate, Historical climatology, History of climate change policy and politics, History of climate change science, Holocene climatic optimum, Homogenization (climate), How Global Warming Works, How to Prepare for Climate Change, Human rights and climate change, Humid temperate climate, Ice cap climate, Idealized greenhouse model, Illustrative model of greenhouse effect on climate change, Impact of the Music of the Spheres World Tour, Index of climate change articles, India Climate Collaborative, Indian Network on Climate Change Assessment, Indian Youth Climate Network, Indigenous Peoples Climate Change Assessment Initiative, Individual action on climate change, Inside Climate News, Instrumental temperature record, Intergovernmental Panel on Climate Change, Interim Climate Change Committee, International Climate Change Partnership, International Comprehensive Ocean-Atmosphere Data Set, International Conference on Climate Change, International Day for the Preservation of the Ozone Layer, International Geosphere-Biosphere Programme, International Indigenous Peoples Forum on Climate Change, International Journal of Climatology, International Journal of Greenhouse Gas Control, International Renewable Energy Agency, International Satellite Cloud Climatology Project, IPCC Fifth Assessment Report, IPCC First Assessment Report, IPCC Fourth Assessment Report, IPCC Second Assessment Report, IPCC Sixth Assessment Report, IPCC Summary for Policymakers, IPCC supplementary report, 1992, IPCC Third Assessment Report, Jacchia Reference Atmosphere, Johannesburg Renewable Energy Coalition, Joint Institute for the Study of the Atmosphere and Ocean, Journal for Geoclimatic Studies, Journal of Applied Meteorology and Climatology, Journal of Climate, Laboratoire des sciences du climat et de l'environnement, Land surface effects on climate, Land surface models (climate), Life-cycle greenhouse gas emissions of energy sources, Liquid carbon dioxide, List of abbreviations relating to climate change, List of books about renewable energy, List of climate activists, List of climate change books, List of climate change initiatives, List of climate engineering topics, List of climate research satellites, List of climate scientists, List of countries by carbon dioxide emissions, List of countries by carbon dioxide emissions per capita, List of countries by greenhouse gas emissions, List of countries by greenhouse gas emissions per capita, List of extreme temperatures in Australia, List of extreme temperatures in Canada, List of extreme temperatures in Denmark, List of extreme temperatures in Finland, List of extreme temperatures in France, List of extreme temperatures in Germany, List of extreme temperatures in Greece, List of extreme temperatures in Italy, List of extreme temperatures in Japan, List of extreme temperatures in Portugal, List of extreme temperatures in Spain, List of extreme temperatures in Sweden, List of extreme temperatures in Vatican City, List of extreme weather records in Pakistan, List of films about renewable energy, List of ministers of climate change, List of periods and events in climate history, List of planned renewable energy projects, List of renewable energy organizations, List of renewable energy topics by country and territory, List of school climate strikes, List of U.S. states and territories by carbon dioxide emissions, List of women climate scientists and activists, Lists of renewable energy topics, London Climate Change Agency, Low Carbon Communities, Low Carbon Vehicle Event, Lowest temperature recorded on Earth, Major Economies Forum on Energy and Climate Change, Maldivian Youth Climate Network, Mandatory renewable energy target, Mayors National Climate Action Agenda, Media coverage of climate change, Mercator Research Institute on Global Commons and Climate Change, Microclimate, Mid-24th century BCE climate anomaly, Middle Eocene Climatic Optimum, Midwestern Greenhouse Gas Reduction Accord,

Millennium Alliance for Humanity and the Biosphere, Minister for Climate Change (New Zealand), Minister for Climate Change and Energy, Minister of State for Energy Security and Net Zero, Ministry of Climate Change (Pakistan), Ministry of Climate, Energy and Utilities (Denmark), Ministry of Economic Affairs and Climate Policy, Ministry of Electricity and Renewable Energy (Egypt), Ministry of Electricity, Water and Renewable Energy (Kuwait), Ministry of Environment and Climate Change (Qatar), Ministry of Environment, Forest and Climate Change, Ministry of New and Renewable Energy, Monsoon continental climate, Monthly Climatic Data for the World, Morphoclimatic zones, Mumbai Climate Action Plan, Music of the Spheres World Tour, Muslim Seven Year Action Plan on Climate Change, National Action Plan for Climate Change, National Climate Assessment, National Climate Change Secretariat, National Climatic Data Center, National Council on Climate Change, National Initiative on Climate Resilient Agriculture, National Oceanic and Atmospheric Administration Climate and Societal Interactions Program, National Renewable Energy Action Plan, National Solar Conference and World Renewable Energy Forum 2012, Nature Climate Change, Net zero emissions, New and Renewable Energy Authority, New England Governors and Eastern Canadian Premiers Climate Change Action Plan 2001, New South Wales Greenhouse Gas Abatement Scheme, New York City Panel on Climate Change, Nigeria Renewable Energy Master Plan, Noordwijk Climate Conference, North African climate cycles, Nuclear power proposed as renewable energy, NZ Climate Party, Office of Energy Efficiency and Renewable Energy, OneClimate, Ozone, Ozone depletion, Ozone depletion and climate change, Ozone depletion potential, Ozone layer, Ozone Mapping and Profiler Suite, Ozone monitoring instrument, Pacific Climate Warriors, Palaeogeography, Palaeoclimatology, Palaeoecology, Pan African Climate Justice Alliance, Pan-African Media Alliance on Climate Change, Pan-Canadian Framework on Clean Growth and Climate Change, Pastoral Greenhouse Gas Research Consortium, People's Climate Movement, Photochemical reduction of carbon dioxide, Photoelectrochemical reduction of carbon dioxide, Poland National Renewable Energy Action Plan, Politics of climate change, Portal:Climate change, Portal:Renewable energy, Potsdam Institute for Climate Impact Research, Premier's Climate Change Council, Presbyterian Church (U.S.A.) Carbon Neutral Resolution, Presidential Climate Action Plan, Program for Climate Model Diagnosis and Intercomparison, Program on Energy Efficiency in Artisanal Brick Kilns in Latin America to Mitigate Climate Change, Proxy (climate), Psychological impact of climate change, Psychology of climate change denial, Public opinion on climate change, Punjab Renewable Energy Systems Pvt. Ltd., R20 Regions of Climate Action, Rapid Climate Change-Meridional Overturning Circulation and Heatflux Array, Recognizing the duty of the Federal Government to create a Green New Deal, Reflective surfaces (climate engineering), Regional climate levels in viticulture, Regional Greenhouse Gas Initiative, Regulation of greenhouse gases under the Clean Air Act, Renewable energy, Renewable Energy (journal), Renewable energy and mining, Renewable Energy Certificate (United States), Renewable Energy Certificate System, Renewable Energy Certificates Registry, Renewable energy commercialization, Renewable energy cooperative, Renewable energy debate, Renewable Energy Directive 2018, Renewable energy in Brunei, Renewable energy in developing countries, Renewable energy in Luxembourg, Renewable energy industry, Renewable energy law, Renewable Energy Payments, Renewable energy sculpture, Renewable Energy Sources and Climate Change Mitigation, Runaway greenhouse effect, San Diego Climate Action Plan, San Francisco Climate Action Plan, Save the Climate, School Strike for Climate, Scientific Assessment of Ozone Depletion, Scientific consensus on climate change, Scorcher: The Dirty Politics of Climate Change, Sea ice emissivity modelling, Sea ice growth processes, Sea ice thickness, Sea level rise, Sea level rise in New Zealand, Seawater greenhouse, September 2019 climate strikes, Singularity (climate), Soil-plant-atmosphere continuum, Solar activity and climate, Solar Renewable Energy Certificate, South Pacific Sea Level and Climate Monitoring Project, Space mirror (climate engineering), Space-based measurements of carbon dioxide, Special Report on Climate Change and Land, Special Report on the Ocean and Cryosphere in a Changing Climate, State of the Climate, Stratospheric Processes And

their Role in Climate, Subhumid temperate climate, Supercritical carbon dioxide, Supercritical carbon dioxide blend, Surface Ocean Lower Atmosphere Study, Sweden National Renewable Energy Action Plan, Table of historic and prehistoric climate indicators, Talk:Sea level rise, Tarawa Climate Change Conference, Temperature record of the last 2,000 years, Template:Climate change in Canada, Template:Climate-change-book-stub, Template:Climate-change-stub, Template:Climate-journal-stub, Template:Climate-stub, Territorial Approach to Climate Change, The Climate Group, The Climate Mobilization, The Climate Reality Project, The Climate Registry, The Discovery of Global Warming, The Doubt Machine: Inside the Koch Brothers' War on Climate Science, The Global Warming Policy Foundation, The Great Derangement: Climate Change and the Unthinkable, The Great Global Warming Swindle, The Greenhouse Conspiracy, The Islamic Declaration on Global Climate Change, The New Climate War, Theoretical and Applied Climatology, Thornthwaite climate classification, Tianjin Climate Exchange, Timeline of international climate politics, Tipping points in the climate system, Tornado climatology, Toronto Conference on the Changing Atmosphere, Total Ozone Mapping Spectrometer, Transatlantic Climate Bridge, Transient climate response to cumulative carbon emissions, Transient climate simulation, Transportation and Climate Initiative, Trewartha climate classification, Tropical cyclones and climate change, Tropospheric ozone depletion events, Tundra climate, U.S. Climate Action Partnership, U.S. Climate Change Technology Program, U.S. Special Presidential Envoy for Climate, UK Climate Assembly, UK Health Alliance on Climate Change, United Kingdom Climate Change Programme, United Kingdom National Renewable Energy Action Plan, United Nations Special Envoy on Climate Change, United States Climate Alliance, United States federal register of greenhouse gas emissions, United States House Select Committee on Energy Independence and Global Warming, United States House Select Committee on the Climate Crisis, United States rainfall climatology, United States Senate Environment Subcommittee on Clean Air, Climate and Nuclear Safety, Urban climate, Urban climatology, Urbanization and Global Environmental Change Project, VAMOS Ocean-Cloud-Atmosphere-Land Study, Vatican Climate Forest, Vienna Convention for the Protection of the Ozone Layer, Wadebridge Renewable Energy Network, Walloon platform for the IPCC, Weather, Climate, and Society, Western Climate Initiative, Western Hemisphere Warm Pool, Weyburn-Midale Carbon Dioxide Project, White House Office of Domestic Climate Policy, White House Office of Energy and Climate Change Policy, Whole Atmosphere Community Climate Model, Women in climate change, World Climate Change Conference, Moscow, World Climate Conference, World Climate Programme, World Council for Renewable Energy, World Mayors Council on Climate Change, World People's Conference on Climate Change, World Renewable Energy Network, World Wide Views on Global Warming, Wuppertal Institute for Climate, Environment and Energy, XCO2, Yale Program on Climate Change Communication, Youth Climate Movement

List of English Wikipedia articles used to track public engagement in health

1510 influenza pandemic, 1557 influenza pandemic, 1626 influenza pandemic, 1793 Philadelphia yellow fever epidemic, 1837 Great Plains smallpox epidemic, 1847 North American typhus epidemic, 1856 Guam smallpox epidemic, 1862 Pacific Northwest smallpox epidemic, 1870 Barcelona yellow fever epidemic, 1896 Gloucester smallpox epidemic, 1906 malaria outbreak in Ceylon, 1915 typhus and relapsing fever epidemic in Serbia, 1918 flu pandemic in India, 1974 smallpox epidemic in India, 1983 West Bank fainting epidemic, 1985 World Health Organization AIDS surveillance case definition, 1994 expanded World Health Organization AIDS case definition, 1998 Winter Olympics flu epidemic, 2009 swine flu pandemic, 2009 swine flu pandemic actions concerning pigs, 2009 swine flu pandemic by country, 2009 swine flu pandemic in India, 2009 swine flu pandemic tables, 2009 swine flu pandemic timeline, 2009 swine flu pandemic timeline summary, 2009 swine flu pandemic vaccine, 2013 Swansea measles epidemic, 2018 Madagascar measles outbreak, 2019 Kuala Koh measles outbreak, 2019

measles outbreak in the Philippines, 2019 New York measles outbreak, 2019 Pacific Northwest measles outbreak, 2019 Samoa measles outbreak, 2019 Tonga measles outbreak, 2021 South Sudan disease outbreak, 2023 Chinese pneumonia outbreak, 2023 Ohio pneumonia outbreak, 412 BC epidemic, Abrazo Community Health Network, Academy of Nutrition and Dietetics, Access Health CT, Accredited Social Health Activist, Action for Global Health, Action on Smoking and Health, Acute eosinophilic pneumonia, Addison's disease, Adenovirus infection, Adult-onset Still's disease, Affordable Medicines Facility-malaria, Africa Centres for Disease Control and Prevention, Africa Fighting Malaria, African Health Economics and Policy Association, African Health Sciences, African Journal for Physical, Health Education, Recreation and Dance, African Malaria Network Trust, African Nutrition Leadership Programme, Against Malaria Foundation, Aging-associated diseases, Air pollution, Air pollution and traffic congestion in Tehran, Air pollution forecasting, Air pollution in Hong Kong, Air pollution in Karachi, Air pollution in Lahore, Air pollution in Macau, Air pollution measurement, Air Quality Health Index (Canada), Airport malaria, Akureyri disease, Alberta Health Insurance Act (1935), Alcohol and health, Alexander disease, Alliance for Health Policy and Systems Research, Alliance for Healthy Cities, AllianceHealth Durant, Alzheimer's disease biomarkers, Alzheimer's Disease Cooperative Study, Alzheimer's disease in African Americans, Alzheimer's disease in the media, Alzheimer's Disease Neuroimaging Initiative, Amazon Malaria Initiative, American Association for the Study and Prevention of Infant Mortality, American Association of Public Health Dentistry, American Association of Public Health Physicians, American College of Epidemiology, American Journal of Epidemiology, American Journal of Health Behavior, American Journal of Public Health, American Journal of Public Health and the Nation's Health, American Public Health Association, American School Health Association, American Sexual Health Association, American Society for Nutrition, Anaerobic infection, Andersen healthcare utilization model, Animal nutrition, AnMed Health Women's & Children's Hospital, Annals of Epidemiology, Annual Review of Public Health, Anti-AQP4 disease, Anti-IgLON5 disease, Antimalarial medication, Apparent infection rate, Applications of sensitivity analysis in epidemiology, Asia Pacific Leaders Malaria Alliance, Asia-Pacific Journal of Public Health, Aspiration pneumonia, Association for Community Health Improvement, Association of Medical Microbiology and Infectious Disease Canada, Association of Public Health Laboratories, Atrium Health, Atrium Health Cabarrus, Atrium Health Mercy, Atrium Health Pineville, Atrium Health Union, Atrium Health University City, Atrium Health Wake Forest Baptist, Atypical pneumonia, Australian Journal of Primary Health, Australian Longitudinal Study on Women's Health, Australian Measles Control Campaign, Autoimmune disease, Autoimmune disease in women, Autoimmune inner ear disease, Autoimmune skin diseases in dogs, Autoinflammatory diseases, Autosomal dominant polycystic kidney disease, Autosomal recessive polycystic kidney disease, Avalere Health, Bacterial pneumonia, Balwadi Nutrition Programme, Bamrasnaradura Infectious Diseases Institute, Bandim Health Project, Bangladesh Institute of Child and Mother Health, Bangladesh Institute of Child Health, Bangladesh National Nutrition Council, Baptist Health System, Batten disease, Behavior change (public health), Belgian Health Care Knowledge Centre, BENTA disease, Bill of health, Bills of mortality, Binswanger's disease, Biochemistry of Alzheimer's disease, Biologically based mental illness, Biotin-thiamine-responsive basal ganglia disease, Biphasic disease, Black Maternal Health Caucus, Black Women's Health Study, Blackheart (plant disease), Blood-borne disease, Bloodstream infections, Blount's disease, Bluetongue disease, BMC Health Services Research, BMC Public Health, BMJ Global Health, Bombay plague epidemic, Bone health, Brazilian Health Regulatory Agency, British Dental Health Foundation, British Nutrition Foundation, Bronchopneumonia, Brookwood Baptist Health, Bulletin of the World Health Organization, Busselton Health Study, C3 Collaborating for Health, Caerphilly Heart Disease Study, Calcium pyrophosphate dihydrate crystal deposition disease, California Center for Public Health Advocacy, Campaign for Better Health Care, Canadian Journal of Public Health, Canadian Public Health Association, Canadian

Society for Epidemiology and Biostatistics, Canavan disease, Cancer Epidemiology (journal), Cancer Epidemiology, Biomarkers & Prevention, Capitation (healthcare), Cardiovascular disease, Cardiovascular disease in women, Caribbean Public Health Agency, Carlos III Health Institute, Carolinas College of Health Sciences, Carondelet Health Network, Carrion's disease, Case management (mental health), Castleman disease, Cat-scratch disease, Catheter-associated urinary tract infection, Causes of mental disorders, Center for Global Infectious Disease Research, Center for Infectious Disease Research and Policy, Centre for Health and International Relations, Centre for Health Protection, Cerebrovascular disease, Chagas disease, CHAI disease, Chicago 1885 cholera epidemic myth, Child Health and Nutrition Research Initiative, Child health in Uganda, Child Health International, Child mortality, Childhood chronic illness, Children's Healthcare of Atlanta, Children's Healthcare of Atlanta at Egleston, Children's Healthcare of Atlanta at Hughes Spalding, Children's Healthcare of Atlanta at Scottish Rite, Children's right to adequate nutrition in New Zealand, Chinese Center for Disease Control and Prevention, Chinese Classification of Mental Disorders, Chlamydia pneumoniae, Chloroquine and hydroxychloroquine during the COVID-19 pandemic, Choosing Healthplans All Together, Chronic kidney disease, Chronic Lyme disease, Chronic obstructive pulmonary disease, Cinematography in healthcare, Circle of Health International, Classification of mental disorders, Classification of pneumonia, Climate change and infectious diseases, Clinical epidemiology, Clinical Epidemiology (journal), Clinical nutrition, Clinton health care plan of 1993, Clostridioides difficile infection, CNS demyelinating autoimmune diseases, Coalition for Epidemic Preparedness Innovations, Cognitive epidemiology, Cohorts for Heart and Aging Research in Genomic Epidemiology, Coinfection, Cold agglutinin disease, Colorado Department of Health Care Policy and Financing, Commission on Health Research for Development, Common disease-common variant, CommonSpirit Health, Community Dentistry and Oral Epidemiology, Community health, Community health agent, Community Health Clubs in Africa, Community Health Systems, Community-acquired pneumonia, Comorbidity, Comparison of the healthcare systems in Canada and the United States, Compartmental models in epidemiology, Compression of morbidity, Computational epidemiology, Cone Health, Cone Health Behavioral Health Hospital, Cone Health Women's Hospital, Conflict epidemiology, Congenital cytomegalovirus infection, Congenital malaria, Connecting Organizations for Regional Disease Surveillance, Consortium of Universities for Global Health, Contagious bovine pleuropneumonia, Contagious disease, Convention on Long-Range Transboundary Air Pollution, Corn stunt disease, Coronary artery disease, Coronavirus diseases, Coughs and sneezes spread diseases, Council on Education for Public Health, COVID-19 pandemic, COVID-19 pandemic cases in April 2020, COVID-19 pandemic cases in April 2021, COVID-19 pandemic cases in August 2020, COVID-19 pandemic cases in August 2021, COVID-19 pandemic cases in December 2020, COVID-19 pandemic cases in February 2020, COVID-19 pandemic cases in February 2021, COVID-19 pandemic cases in January 2020, COVID-19 pandemic cases in January 2021, COVID-19 pandemic cases in July 2020, COVID-19 pandemic cases in July 2021, COVID-19 pandemic cases in June 2020, COVID-19 pandemic cases in June 2021, COVID-19 pandemic cases in March 2020, COVID-19 pandemic cases in March 2021, COVID-19 pandemic cases in May 2020, COVID-19 pandemic cases in May 2021, COVID-19 pandemic cases in November 2020, COVID-19 pandemic cases in October 2020, COVID-19 pandemic cases in September 2020, Creativity and mental health, Creutzfeldt-Jakob Disease Surveillance System, Creutzfeldt–Jakob disease, Critical illness insurance, Critical Public Health, Crohn's disease, Cryptogenic organizing pneumonia, Cure Rare Disease, Cytomegaloviral disease, Cytomegalovirus infection, Dance and health, Darier's disease, Deen Dayal Mobile Health Clinic, Degenerative disease, Dementia and Alzheimer's disease in Australia, Dengue pandemic in Sri Lanka, Dent's disease, Depression of Alzheimer disease, Desquamative interstitial pneumonia, Diabetic foot infection, Diagnosis of malaria, Diagnostic and Statistical Manual of Mental Disorders, Diarrhoea, Diarrhoeal disease, Dignity Health, Dignity Health St. Joseph's Hospital and Medical Center, Director-

General of the World Health Organization, Directorate of Health, Disability and women's health, Discovery of disease-causing pathogens, Disease, Disease burden, Disease cluster, Disease Control Priorities Project, Disease diffusion mapping, Disease ecology, Disease management (health), Disease of despair, Disease outbreak, Disease resistance, Disease surveillance, Disease vector, Disease X, Disease-modifying osteoarthritis drug, Diseases, Diseases of abnormal polymerization, Diseases of poverty, Disneyland measles outbreak, Disseminated disease, Doctor of Public Health, Dole Nutrition Institute, Drug, Healthcare and Patient Safety, Drugs for Neglected Diseases Initiative, Dukes' disease, Dunedin Multidisciplinary Health and Development Study, Dust pneumonia, E-epidemiology, Early-onset Alzheimer's disease, Eastern Mediterranean Health Journal, Ecological health, Economic epidemiology, Ecosystem health, Effects of climate change on human health, Ehrlichiosis ewingii infection, EMBRACE Healthcare Reform Plan, Emerging infectious disease, Emerging Themes in Epidemiology, Endemic (epidemiology), Endogenous infection, Engineering World Health, Environmental disease, Environmental epidemiology, Environmental health, Environmental health ethics, Environmental health officer, Environmental health policy, Eosinophilic pneumonia, Epidemic, Epidemic curve, Epidemic Intelligence Service, Epidemic models on lattices, Epidemic polyarthritis, Epidemic typhus, Epidemics (journal), Epidemiology, Epidemiology (journal), Epidemiology and Infection, Epidemiology and Psychiatric Sciences, Epidemiology data for low-linear energy transfer radiation, Epidemiology in Country Practice, Epidemiology in Relation to Air Travel, Epidemiology of asthma, Epidemiology of attention deficit hyperactive disorder, Epidemiology of bed bugs, Epidemiology of binge drinking, Epidemiology of breast cancer, Epidemiology of cancer, Epidemiology of chikungunya, Epidemiology of child psychiatric disorders, Epidemiology of childhood obesity, Epidemiology of depression, Epidemiology of diabetes, Epidemiology of malnutrition, Epidemiology of measles, Epidemiology of metabolic syndrome, Epidemiology of pneumonia, Epidemiology of schizophrenia, Epidemiology of suicide, Epidemiology of syphilis, Epidemiology of tuberculosis, Eradication of infectious diseases, Escape Fire: The Fight to Rescue American Healthcare, Essence (Electronic Surveillance System for the Early Notification of Community-based Epidemics), Establishment of the World Health Organization, European Centre for Disease Prevention and Control, European Health Examination Survey, European Health Forum Gastein, European Journal of Epidemiology, European Journal of Public Health, European Observatory on Health Systems and Policies, European Organisation for Rare Diseases, European Parliament Committee on the Environment, Public Health and Food Safety, European Programme for Intervention Epidemiology Training, European Prospective Investigation into Cancer and Nutrition, European Public Health Alliance, European Public Health Association, European Society for Paediatric Infectious Diseases, European Society of Clinical Microbiology and Infectious Diseases, European Working Group for Legionella Infections, Evaluation & the Health Professions, Evolution of Infectious Disease, Extramammary Paget's disease, Eye Health UK, Fabry disease, Face masks during the COVID-19 pandemic, Faculty of Public Health, Fair Share Health Care Act, Farber disease, Febrile infection-related epilepsy syndrome, Federal Service for Surveillance in Healthcare, Federation of European Nutrition Societies, Federation of Health and Social Services, Fifth disease, Finnish Institute for Health and Welfare, Fire breather's pneumonia, First Nations nutrition experiments, First plague pandemic, Focus of infection, Foodborne illness, Foot-and-mouth disease, Free-market healthcare, Fungal infection, Fungal pneumonia, Gamaleya Research Institute of Epidemiology and Microbiology, Gastropod-borne parasitic disease, Gaucher's disease, Gender disparities in health, General Health Questionnaire, Genetic epidemiology, Genetic Epidemiology (journal), George Institute for Global Health, Germ theory of disease, GIS and public health, Global Acute Malnutrition, Global Alliance for Improved Nutrition, Global Alliance on Health and Pollution, Global Burden of Disease Study, Global Climate and Health Alliance, Global Forum for Health Research, Global health, Global Health Action, Global Health Council, Global Health Delivery Project, Global Health Initiatives, Global Health

Innovative Technology Fund, Global Health Observatory, Global Health Security Agenda, Global Health Security Index, Global Health Security Initiative, Global Health Share Initiative, Global Infectious Disease Epidemiology Network, Global Initiative for Chronic Obstructive Lung Disease, Global Malaria Action Plan, Global mental health, Global Network for Neglected Tropical Diseases, Global Public Health (journal), Global Public Health Intelligence Network, Global Research Collaboration for Infectious Disease Preparedness, Global Strategy for Women's and Children's Health, Globalization and disease, Globalization and Health, Glossary of the COVID-19 pandemic, Goal-oriented health care, Gold Coast Influenza Epidemic, Graduate School of Health Economics and Management, Grand Challenges in Global Health, Graves' disease, Groningen epidemic, Grossman model of health demand, Group B streptococcal infection, Handbook of Religion and Health, HCA Healthcare, Health & Place, Health & Social Care in the Community, Health 21, Health Action International, Health administration, Health advocacy, Health Advocate, Health Alliance International, Health and Human Rights, Health and Social Protection Federation, Health and wealth, Health belief model, Health Books International, Health campaign, Health Canada, Health care, Health Care Compact, Health care efficiency measures, Health care finance in the United States, Health Care for America NOW!, Health Care for Women International, Health care prices in the United States, Health care ratings, Health care rationing, Health care reform, Health care reforms proposed during the Obama administration, Health care time and motion study, Health Check, Health communication, Health Communication (journal), Health Communication Network, Health consequences of the Deepwater Horizon oil spill, Health crisis, Health Data Insight, Health departments in the United States, Health Development Agency, Health disaster, Health Disparities Center, Health Dynamics Inventory, Health ecology, Health economics, Health economics (Germany), Health Economics, Policy and Law, Health education, Health Education & Behavior, Health Education Exhibition and Resource Centre, Health Education Journal, Health Education Research, Health effect, Health Effects Institute, Health effects of caffeine, Health effects of coffee, Health effects of green tea, Health effects of salt, Health effects of sugar, Health effects of sugary drinks, Health effects of tea, Health effects of wine, Health effects of wood smoke, Health Emergencies Programme (WHO), Health equity, Health eResearch Centre, Health fair, Health For All, Health geography, Health human resources, Health humanities, Health impact assessment, Health in All Policies, Health Information National Trends Survey, Health information on Wikipedia, Health insurance, Health insurance cooperative, Health insurance coverage in the United States, Health Insurance Innovations, Health insurance mandate, Health insurance marketplace, Health law, Health literacy, Health marketing, Health measures during the construction of the Panama Canal, Health Metrics Network, Health of Hillary Clinton, Health of Towns Association, Health policy, Health policy and management, Health policy in Bangladesh, Health politics, Health promotion, Health Promotion Board, Health promotion in higher education, Health Promotion International, Health Promotion Practice, Health Protection Surveillance Centre, Health risk assessment, Health Sciences Online, Health security, Health Security Express, Health services research, Health Services Workers' Union, Health spending as percent of gross domestic product (GDP) by country, Health surveillance, Health system, Health systems strengthening, Health Threat Unit, Health Utilities Index, Health-related embarrassment, Health, Risk & Society, Healthcare Information For All, Healthcare policies of candidates in the 2008 United States presidential election, Healthcare rationing in the United States, Healthcare reform debate in the United States, Healthcare reform in the United States, Healthcare transport, HealthCare Volunteer, HealthCare.gov, Healthiest State in the Nation Campaign, Healthlink Worldwide, HealthMap, HealthOne, HealthRight International, Healthy city, Healthy community design, Healthy development measurement tool, Healthy Life Years, Healthy People program, HealthyWomen, Heat illness, High-deductible health plan, High-dependency unit (mental health), Hispanic Health Council, History of health care reform in the United States, History of malaria, History of mental disorders, Holozoic nutrition, Hookworm infection, Hospital-acquired infection, Hospital-

acquired pneumonia, Household air pollution, How to Have Sex in an Epidemic, How to Prevent the Next Pandemic, Human genetic resistance to malaria, Human Heredity and Health in Africa, Human nutrition, Human papillomavirus infection, Human Resources for Health, Huntington's disease, Hypertensive disease of pregnancy, Idiopathic disease, Idiopathic interstitial pneumonia, Idiopathic multicentric Castleman disease, Idiopathic pneumonia syndrome, IgG4-related disease, Illinois Health Benefits Exchange, Illness as Metaphor, Imagine No Malaria, Impact of the COVID-19 pandemic on children, Independent Panel for Pandemic Preparedness and Response, Inequality in disease, Infant mortality, Infection, Infection Control Society of Pakistan, Infection prevention and control, Infection rate, Infections associated with diseases, Infectious Disease (Notification) Act 1889, Infectious Disease Pharmacokinetics Laboratory, Infectious diseases, Infectious diseases (medical specialty), Infectious Diseases Institute, Infectious Diseases Society of America, Inflammatory bowel disease, Inflammatory demyelinating diseases of the central nervous system, Influenza pandemic, Influx of disease in the Caribbean, Inquiry (health journal), Institute for Health Metrics and Evaluation, Institute of Nutrition of Central America and Panama, Institute of Public Health (Bangladesh), Integrated Disease Surveillance Programme, Intentional contagion of infection, Interdisciplinary Association for Population Health Science, Intermountain Health, International Association of National Public Health Institutes, International Centre for Migration and Health, International Classification of Diseases, International Classification of Functioning, Disability and Health, International Conference on Emerging Infectious Diseases, International health, International Health (journal), International Health Exhibition, International Health Regulations, International Journal of Environmental Research and Public Health, International Journal of Epidemiology, International Journal of Men's Health, International Journal of Public Health, International Journal of Qualitative Studies on Health and Well-being, International Journal of Technology Assessment in Health Care, International Men's Health Week, International Network of Health Promoting Hospitals and Health Services, International Partnership on Avian and Pandemic Influenza, International Society for Environmental Epidemiology, International Society for Infectious Diseases in Obstetrics and Gynaecology, International Society for Pharmacoepidemiology, International Union of Air Pollution Prevention and Environmental Protection Associations, International Volcanic Health Hazard Network, Intestinal infectious diseases, Intradialytic parenteral nutrition, Iranian Journal of Public Health, Irish Nutrition and Dietetic Institute, Iron Triangle of Health Care, Isolation (health care), ITU-WHO Focus Group on Artificial Intelligence for Health, James C. Robinson (health economist), James Thornton (health economist), Jembrana disease, Joondalup Family Health Study, Journal for Healthcare Quality, Journal of American College Health, Journal of Clinical Epidemiology, Journal of Community Health, Journal of Developmental Origins of Health and Disease, Journal of Epidemiology, Journal of Epidemiology and Biostatistics, Journal of Epidemiology and Community Health, Journal of Exposure Science and Environmental Epidemiology, Journal of Health and Social Behavior, Journal of Health Care for the Poor and Underserved, Journal of Health Communication, Journal of Health Economics, Journal of Health Management, Journal of Immigrant and Minority Health, Journal of Public Health, Journal of Public Health Management & Practice, Journal of Public Health Policy, Journal of Urban Health, Kawasaki disease, Kids for World Health, Kigali Declaration on Neglected Tropical Diseases, Kivu Ebola epidemic, Korea Disease Control and Prevention Agency, Koro (disease), Krabbe disease, Kyasanur Forest disease, Laboratory-acquired infection, Lafora disease, Landscape epidemiology, Latent period (epidemiology), Legacy Health, Legionnaires' disease, Lenox Health Greenwich Village, Let's Just Play Go Healthy Challenge, Leveraging Agriculture for Improving Nutrition and Health, Lifestyle disease, Lipid pneumonia, List of autoimmune diseases, List of countries by air pollution, List of countries by total health expenditure per capita, List of countries with universal health care, List of diseases eliminated from the United States, List of epidemics and pandemics, List of foodborne illness outbreaks, List of foodborne illness outbreaks by death toll, List of foodborne illness outbreaks in the United States, List of infections of the

central nervous system, List of infectious diseases, List of infectious diseases causing flu-like syndrome, List of insect-borne diseases, List of Legionnaires' disease outbreaks, List of mental disorders, List of national public health agencies, List of pneumonia deaths, List of rare disease organisations, List of sexually transmitted infections by prevalence, List of species named after the COVID-19 pandemic, List of types of malnutrition, Liverpool Neurological Infectious Diseases Course, Lobar pneumonia, Localized disease, London Declaration on Neglected Tropical Diseases, Lower Mississippi Valley yellow fever epidemic of 1878, Lower respiratory tract infection, Lutheran Health Network, Lyme disease, Lyme Disease Awareness Month, Lyme disease microbiology, Lymphocytic interstitial pneumonia, Lysosomal storage disease, Madras motor neuron disease, Malaria, Malaria antigen detection tests, Malaria Atlas Project, Malaria Consortium, Malaria Control Project, Malaria culture, Malaria Day in the Americas, Malaria Eradication Scientific Alliance, Malaria in Benin, Malaria in Madagascar, Malaria in the Caribbean, Malaria in the River Thames, Malaria Journal, Malaria No More, Malaria No More UK, Malaria Policy Advisory Committee, Malaria prophylaxis, Malaria therapy, Malaria vaccine, Malarial nephropathy, Malnutrition, Malnutrition in India, Malnutrition in Kerala, Malnutrition in Peru, Malnutrition in South Africa, Malnutrition in Zimbabwe, Management of Crohn's disease, Managerial epidemiology, Marburg virus disease, Mass psychogenic illness, Massachusetts smallpox epidemic, Maternal and Child Health Handbook, Maternal health, Maternal health in Angola, Maternal mortality ratio, Maternal oral health, Mayaro virus disease, Measles, Measles & Rubella Initiative, Measles hemagglutinin, Measles morbillivirus, Measles resurgence in the United States, Measles vaccine, Measles: A Dangerous Illness, Medical and Health Workers' Union of Nigeria, Medicines for Malaria Venture, Mekong Basin Disease Surveillance, Melanie's Marvelous Measles, Men's health, Men's health in Australia, Meningococcal disease, Mental disorder, Mental disorders and gender, Mental health, Mental health consumer, Mental health first aid, Mental health literacy, Mental illness, Mental illness denial, Mental illness in ancient Greece, Mental illness in ancient Rome, Milk borne diseases, Mineral Nutrition of Plants: Principles and Perspectives, Ministry of Health and Welfare (South Korea), Ministry of Health Promotion and Sport (Ontario), Miscarriage and mental disorders, Mission Health System, Mississippi Health Project, Mitochondrial disease, Mobile source air pollution, Molecular epidemiology, Morbidity and Mortality Weekly Report, Mosquito-borne disease, Mosquito-malaria theory, Motor neuron diseases, Motor Neurone Disease Association, Mount Sinai Health System, MRC Human Nutrition Research, MTNL Perfect Health Mela, Muesli belt malnutrition, Multifactorial disease, Multimorbidity, Music therapy for Alzheimer's disease, Mycobacterium avium-intracellulare infection, Mycoplasma hominis infection, Mycoplasma ovipneumoniae, Mycoplasma pneumonia, Mycoplasma pneumoniae infection, National Aboriginal Health Organization, National Advisory Committee on SARS and Public Health, National Air Pollution Symposium, National Association for Public Health Policy, National Association of County and City Health Officials, National Bone Health Campaign, National Centre for Disease Control, National Centre for Infectious Diseases, National Children's Center for Rural and Agricultural Health and Safety, National Commission for Health Education Credentialing, National Comorbidity Survey, National Emerging Infectious Diseases Laboratories, National Foundation for Infectious Diseases, National Health Fund, National health insurance, National Health Insurance Fund, National Health Interview Survey, National Health Mission, National Health Policy, National Institute for Communicable Diseases, National Institute for Health and Care Excellence, National Institute for Health and Disability Insurance, National Institute for Public Health and the Environment, National Institute of Health, Islamabad, National Institute of Malaria Research, National Institute of Nutrition, Hyderabad, National Institute of Public Health of Japan, National Malaria Eradication Program, National Perinatal Epidemiology Unit, National Prostate Health Month, National public health institute, National Public Health Organization (Greece), National School of Public Health (Spain), Native American disease and epidemics, Native American Women's Health Education Resource Center, Navicent Health Baldwin,

Necrotizing pneumonia, Neglected tropical diseases, Neglected tropical diseases in India, Neonatal infection, Network for Capacity Development in Nutrition, Neuroepidemiology (journal), Nevada Health Link, New Mexico Health Insurance Exchange, NHS Health Scotland, Nigeria Centre for Disease Control, NINCDS-ADRDA Alzheimer's Criteria, Nipah virus infection, Noma (disease), Non-communicable disease, Non-communicable diseases, Non-specific interstitial pneumonia, Northwell Health, Northwest Area Health Education Center, Norwegian Association of Health and Social Care Personnel, Norwegian Institute of Public Health, Notifiable disease, Notifiable diseases in Switzerland, Notifiable diseases in the United Kingdom, Novant Health, Novant Health Forsyth Medical Center, Nurses' Health Study, Nutrition, Nutrition analysis, Nutrition and cognition, Nutrition and Education International, Nutrition education, Nutrition Foundation of the Philippines, Nutritional epidemiology, Nutritional epigenetics, Nutritional genomics, Nutritional neuroscience, Nutritional physiologist, Nutritional rating systems, Nutritional science, Nutritional value, NutritionDay, Nutritionist, Obstacles to receiving mental health services among African American youth, Occult pneumonia, Occupational safety and health, Office for Health Improvement and Disparities, One Health, Opportunistic infection, Oral Health Foundation, Oregon Medicaid health experiment, OSF HealthCare, Osong Public Health and Research Perspectives, Ottawa Charter for Health Promotion, Outline of air pollution dispersion, Outline of public health, Outline of the COVID-19 pandemic, Overwhelming post-splenectomy infection, Oxford Brookes Centre for Nutrition and Health, Pacific Open Learning Health Net, Paediatric and Perinatal Epidemiology, Paget's disease of bone, Paget's disease of the breast, Pan American Journal of Public Health, Pandemic, Pandemic fatigue, Pandemic predictions and preparations prior to the COVID-19 pandemic, Pandemic Preparedness and Response Act, Pandemic prevention, Pandemic Severity Assessment Framework, Pandemic severity index, Papaya Bunchy Top Disease, Parasitic disease, Parasitic pneumonia, Parenteral nutrition, Parkinson's disease, Pathogens and Global Health, Patriotic Health Campaign, Pay for performance (healthcare), Pelvic inflammatory disease, Perspectives in Public Health, Pervasive developmental disorder, Pervasive developmental disorder not otherwise specified, Peyronie's disease, Philosophy of healthcare, Pinta (disease), Pinworm infection, Plague (disease), Plague City: SARS in Toronto, Plague epidemics in Malta, Plant nutrition, Pneumococcal infection, Pneumococcal pneumonia, Pneumocystis pneumonia, Pneumonia, Pneumonia (non-human), Pneumonia jacket, Pneumonia severity index, Pogosta disease, Population health, Population Health Management, Population Health Metrics, Population health policies and interventions, Population, health, and the environment, Portal:Pandemics, Post-acute infection syndrome, Postorgasmic illness syndrome, Pott's disease, Prebiotic (nutrition), Pregnancy-associated malaria, Prenatal nutrition, President's Malaria Initiative, Prevalence of mental disorders, Preventing Chronic Disease, Prevention of mental disorders, Preventive nutrition, Price-Pottenger Nutrition Foundation, Primary Health Centre (India), Prime Healthcare Services, Priority-setting in global health, Prison healthcare, Program for Jewish Genetic Health, Progress in Community Health Partnerships, Progressive disease, Providence Health & Services, Psychiatric epidemiology, Psychogenic disease, Public health, Public Health (journal), Public Health Agency of Canada, Public Health Agency of Sweden, Public health emergency of international concern, Public Health England, Public Health Ethics, Public health insurance option, Public health journal, Public health laboratory, Public Health Nutrition, Public health observatory, Public health policy, Public Health Reports, Public Health Scotland, Public health system in India, Public Health Wales, Publicly funded health care, Pullorum disease, Qapqal disease, Qualitative Health Research, Quantum suicide and immortality, Race and health, RAND Health Insurance Experiment, Rare disease, Rare Diseases Clinical Research Network, Real-time outbreak and disease surveillance, Regional Forum on Environment and Health in Southeast and East Asian Countries, Reproductive health care for incarcerated women in the United States, Reproductive system disease, Respiratory disease, Respiratory tract infection, Responses to the COVID-19 pandemic in April 2020, Responses to the COVID-19 pandemic in April 2022, Responses to

the COVID-19 pandemic in August 2020, Responses to the COVID-19 pandemic in August 2021, Responses to the COVID-19 pandemic in August 2022, Responses to the COVID-19 pandemic in December 2020, Responses to the COVID-19 pandemic in December 2021, Responses to the COVID-19 pandemic in February 2020, Responses to the COVID-19 pandemic in February 2021, Responses to the COVID-19 pandemic in February 2022, Responses to the COVID-19 pandemic in January 2020, Responses to the COVID-19 pandemic in January 2021, Responses to the COVID-19 pandemic in January 2022, Responses to the COVID-19 pandemic in July 2020, Responses to the COVID-19 pandemic in July 2021, Responses to the COVID-19 pandemic in July 2022, Responses to the COVID-19 pandemic in June 2020, Responses to the COVID-19 pandemic in June 2021, Responses to the COVID-19 pandemic in June 2022, Responses to the COVID-19 pandemic in March 2020, Responses to the COVID-19 pandemic in March 2021, Responses to the COVID-19 pandemic in March 2022, Responses to the COVID-19 pandemic in May 2020, Responses to the COVID-19 pandemic in May 2021, Responses to the COVID-19 pandemic in May 2022, Responses to the COVID-19 pandemic in November 2020, Responses to the COVID-19 pandemic in November 2021, Responses to the COVID-19 pandemic in October 2020, Responses to the COVID-19 pandemic in October 2021, Responses to the COVID-19 pandemic in October 2022, Responses to the COVID-19 pandemic in September 2020, Responses to the COVID-19 pandemic in September 2021, Responses to the COVID-19 pandemic in September 2022, Rethink Mental Illness, Rheumatoid disease of the spine, Right to health, Rwandan reproductive health, Salt and cardiovascular disease, Samaritan Health Services, Saprotrophic nutrition, SARS, SARS conspiracy theory, SARS-CoV-1, SARS-CoV-2, Scandinavian Journal of Work, Environment & Health, School-based health centers, Science diplomacy and pandemics, Second plague pandemic, Self-rated health, Sentara Healthcare, Serious mental illness, Services for mental disorders, Seventh cholera pandemic, Sexual and reproductive health, Sexual and Reproductive Health Matters, Sexual health clinic, Sexually transmitted infection, ShorePoint Health Venice, Single-payer healthcare, Sissel v. United States Department of Health & Human Services, Skin and skin structure infection, Skin infection, Smallpox epidemic, Smell as evidence of disease, Social Psychiatry and Psychiatric Epidemiology, Social Work in Public Health, Society and Mental Health, Society for Family Health Nigeria, Society for Public Health Education, Society of Infectious Diseases Pharmacists, Socioeconomic status and mental health, South African Malaria Initiative, South Texas Center for Emerging Infectious Diseases, Southern tick-associated rash illness, Spanish National Health System, Spatial and Spatio-temporal Epidemiology, Spatial epidemiology, Specific replant disease, St. Patrick Hospital and Health Sciences Center, St. Vincent's Health System, Stateville Penitentiary Malaria Study, STOP Foodborne Illness, Strengthening the reporting of observational studies in epidemiology, Streptococcus pneumoniae, Study of Health in Pomerania, Suicide epidemic, Superinfection, Susceptibility and severity of infections in pregnancy, Sustainable Healthcare, Sutter Health, Sweating sickness epidemics, Swedish Association of Health Professionals, Systemic disease, Taiwan Centers for Disease Control, Tanganyika laughter epidemic, Target Malaria, Tay–Sachs disease, Template:Ascension Health, Template:Epidemic-stub, Template:Eradication of infectious disease, Template:Infectious-disease-stub, Template:Malaria, Template:Mental disorders, Template:Plant nutrition, Template:Tick-borne diseases and infestations, Template:Vertically transmitted infection, Template:Women's health, Tenet Healthcare, The Dancing Mania, an epidemic of the Middle Ages, The European Journal of Health Economics, The Global Fund to Fight AIDS, Tuberculosis and Malaria, The Journal of Mental Health Policy and Economics, The Lancet Global Health, The Medical Center, Navicent Health, The Nation's Health, The Office of Health Economics, The Oxcap MH measure of health, Theiler's disease, Third plague pandemic, Tick-borne disease, Tick-Borne Disease Alliance, Time to Change (mental health campaign), Timeline of global health, Timeline of peptic ulcer disease and Helicobacter pylori, Timeline of the COVID-19 pandemic in 2023, Timeline of the COVID-19 pandemic in April 2020, Timeline of the COVID-19 pandemic in April 2021, Timeline of the COVID-

19 pandemic in April 2022, Timeline of the COVID-19 pandemic in August 2020, Timeline of the COVID-19 pandemic in August 2021, Timeline of the COVID-19 pandemic in August 2022, Timeline of the COVID-19 pandemic in December 2020, Timeline of the COVID-19 pandemic in December 2021, Timeline of the COVID-19 pandemic in December 2022, Timeline of the COVID-19 pandemic in February 2020, Timeline of the COVID-19 pandemic in February 2021, Timeline of the COVID-19 pandemic in February 2022, Timeline of the COVID-19 pandemic in January 2020, Timeline of the COVID-19 pandemic in January 2021, Timeline of the COVID-19 pandemic in January 2022, Timeline of the COVID-19 pandemic in July 2020, Timeline of the COVID-19 pandemic in July 2021, Timeline of the COVID-19 pandemic in July 2022, Timeline of the COVID-19 pandemic in June 2020, Timeline of the COVID-19 pandemic in June 2021, Timeline of the COVID-19 pandemic in June 2022, Timeline of the COVID-19 pandemic in March 2020, Timeline of the COVID-19 pandemic in March 2021, Timeline of the COVID-19 pandemic in March 2022, Timeline of the COVID-19 pandemic in May 2020, Timeline of the COVID-19 pandemic in May 2021, Timeline of the COVID-19 pandemic in May 2022, Timeline of the COVID-19 pandemic in November 2020, Timeline of the COVID-19 pandemic in November 2021, Timeline of the COVID-19 pandemic in November 2022, Timeline of the COVID-19 pandemic in October 2020, Timeline of the COVID-19 pandemic in October 2021, Timeline of the COVID-19 pandemic in October 2022, Timeline of the COVID-19 pandemic in September 2020, Timeline of the COVID-19 pandemic in September 2021, Timeline of the COVID-19 pandemic in September 2022, Top dying disease, Trauma model of mental disorders, Treatment of mental disorders, Tropical disease, Typhus epidemic in Goose Village, Montreal, UCLA Health, UCLA Health Training Center, UCSC Malaria Genome Browser, UK Health Alliance on Climate Change, UK Health Security Agency, Undernutrition, Undernutrition in children, Undiagnosed Diseases Network, Uni Health, Unicentric Castleman disease, Union of Healthcare, UnityPoint Health, UnityPoint Health - Allen Hospital, Universal Declaration on the Eradication of Hunger and Malnutrition, Universal health care, Use of technology in treatment of mental disorders, Usual interstitial pneumonia, Vaccine-preventable disease, Value-based health care, Vanguard Health Systems, Vector-borne disease, Vegan nutrition, Vegetarian nutrition, Venereal Disease Research Laboratory test, Ventilator-associated pneumonia, Vermont health care reform, Vertically transmitted infection, Victorian Health Promotion Foundation, Viral disease testing, Viral pneumonia, Virgin soil epidemic, Waterborne disease and climate change, Waterborne diseases, Weather and climate effects on Lyme disease exposure, Western African Ebola virus epidemic, WHO Hub for Pandemic and Epidemic Intelligence, Whole Health Action Management, Wilt disease, Women's health, Women's health movement in the United States, Women's reproductive health in Russia, Workplace health promotion, World Chagas Disease Day, World Health Assembly, World Health Organization, World Health Organization Composite International Diagnostic Interview, World Malaria Day, World Oral Health Day, World Pneumonia Day, Your Health Idaho, Zardip's Search for Healthy Wellness, Zoonoses and Public Health

Complementing the analysis presented in the 2024 *Lancet* Countdown report, the figures below provide additional evidence on dynamics in pageviews and co-click networks (Figures **231** to **232**).

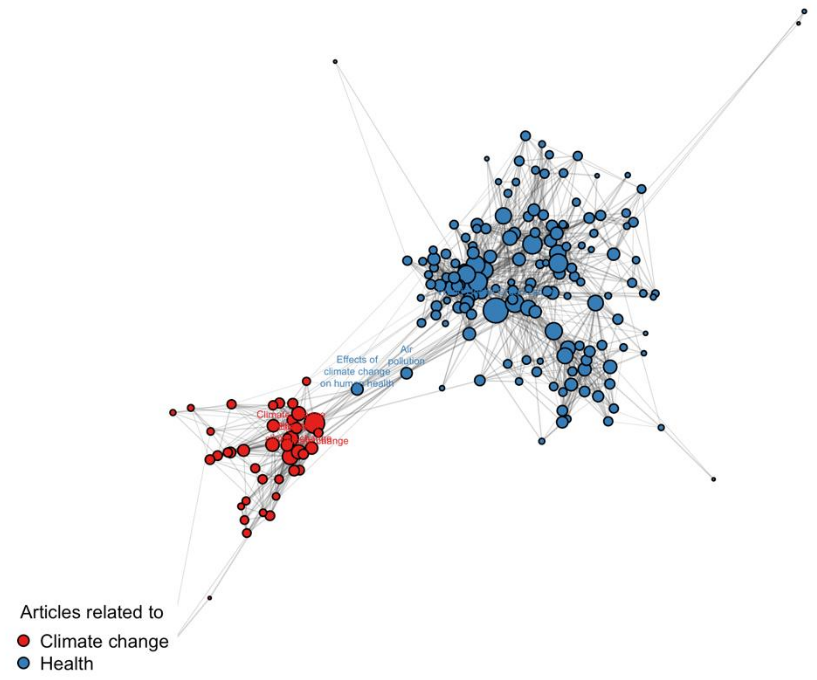

Figure 233: Connectivity graph of Wikipedia articles on climate change (red) and health (blue). Popularity of articles displayed by node size. Edges represent co-visits in the 2023 clickstream data.

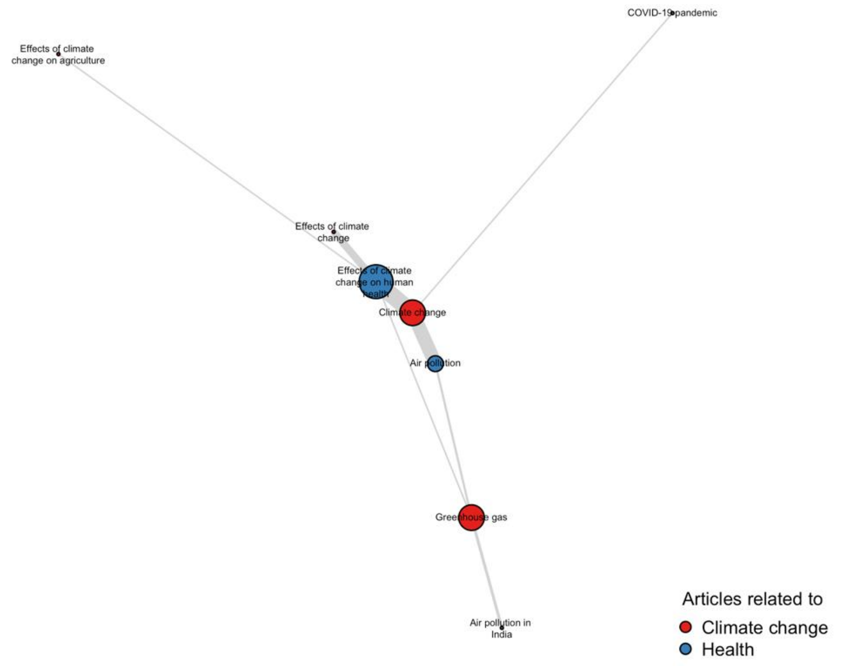

Figure 234: Connectivity graph of Wikipedia articles on climate change (red) and health (blue), filtered to co-click activity between the two domains. Popularity of articles displayed by node size. Edges represent co-visits in the 2023 clickstream data.

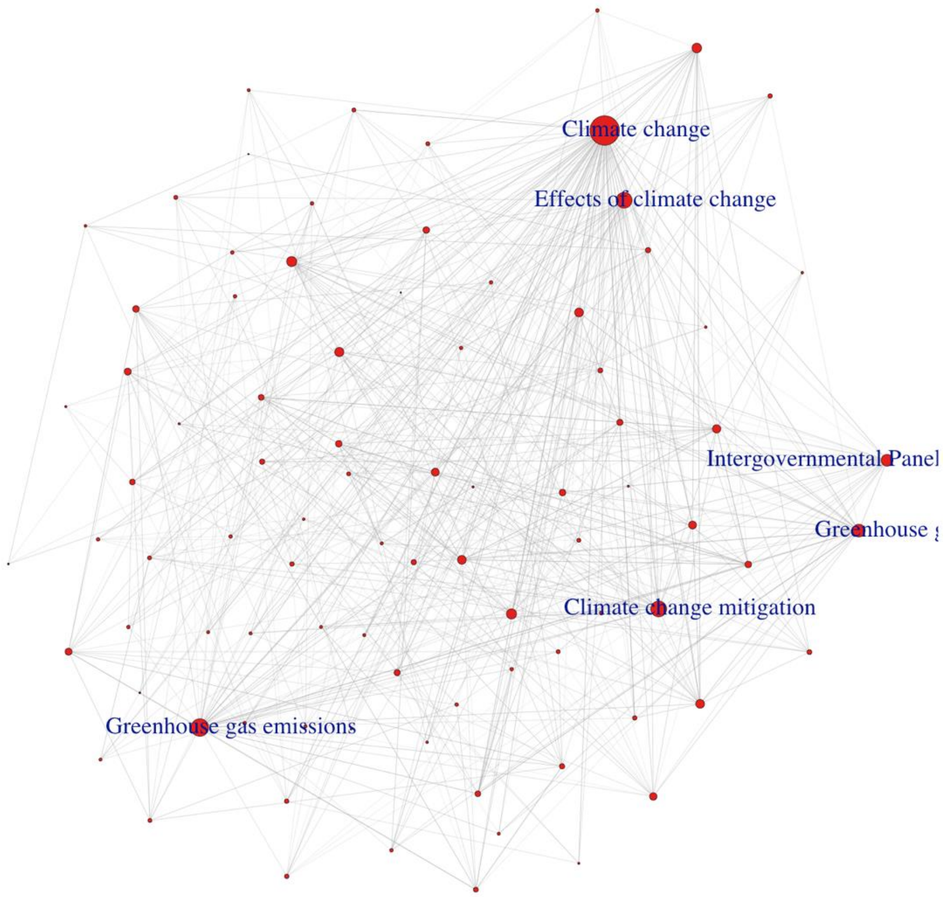

Figure 235: Connectivity graph of Wikipedia articles on climate change. Popularity of articles displayed by node size. Edges represent co-visits in the 2023 clickstream data.

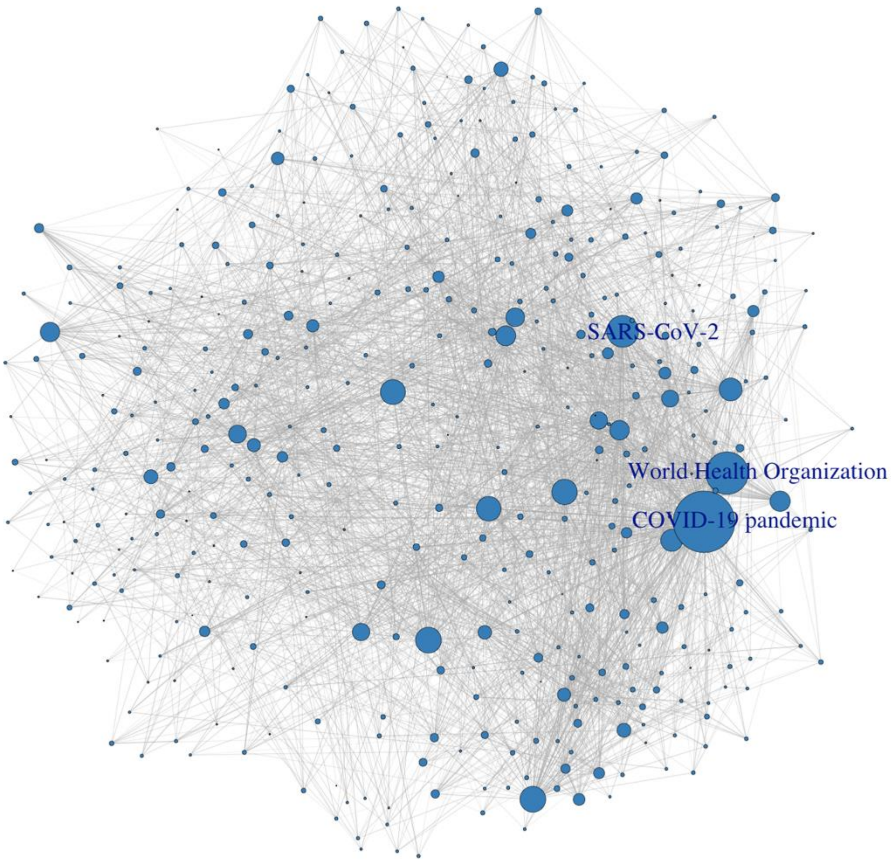

Figure 236: Connectivity graph of Wikipedia articles on health. Popularity of articles displayed by node size. Edges represent co-visits in the 2023 clickstream data.

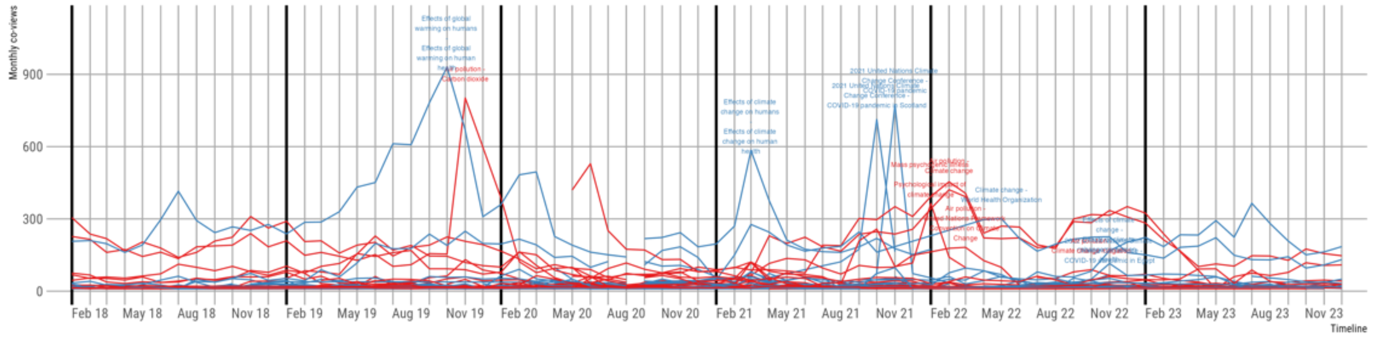

Figure 237: Co-views of climate change-health article pairs over time, 2018-2023. Dominant pairs labelled.

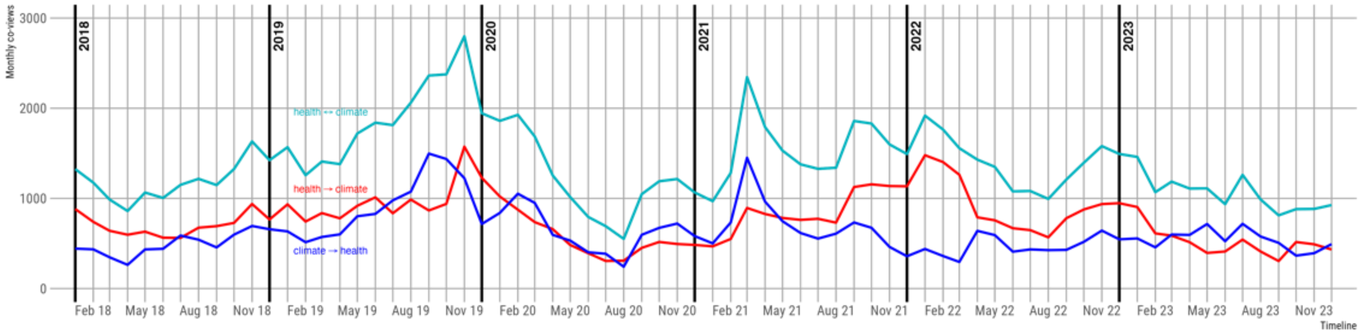

Figure 238: Aggregate monthly co-views of articles related to human health and climate change, 2018–2023 (excluding COVID-19 related articles).

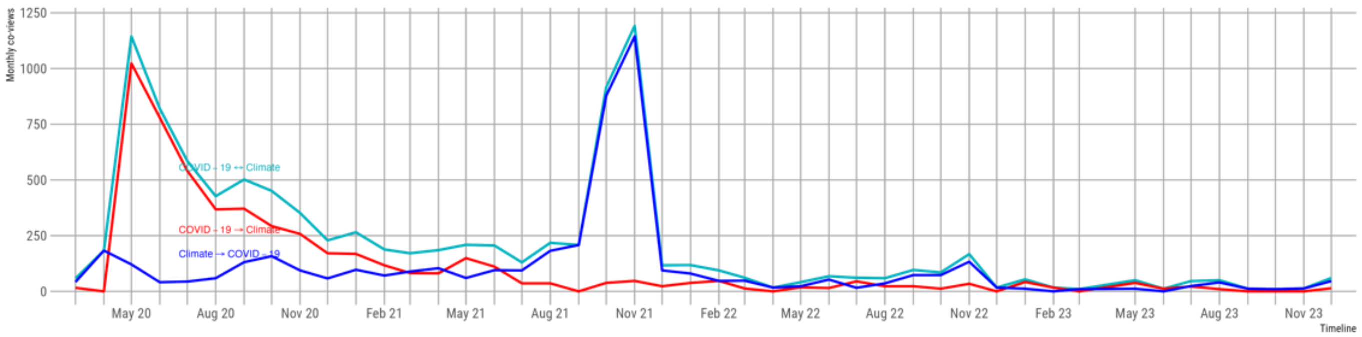

Figure 239: Aggregate monthly co-views of articles related to COVID-19 and climate change, 2020–2023.

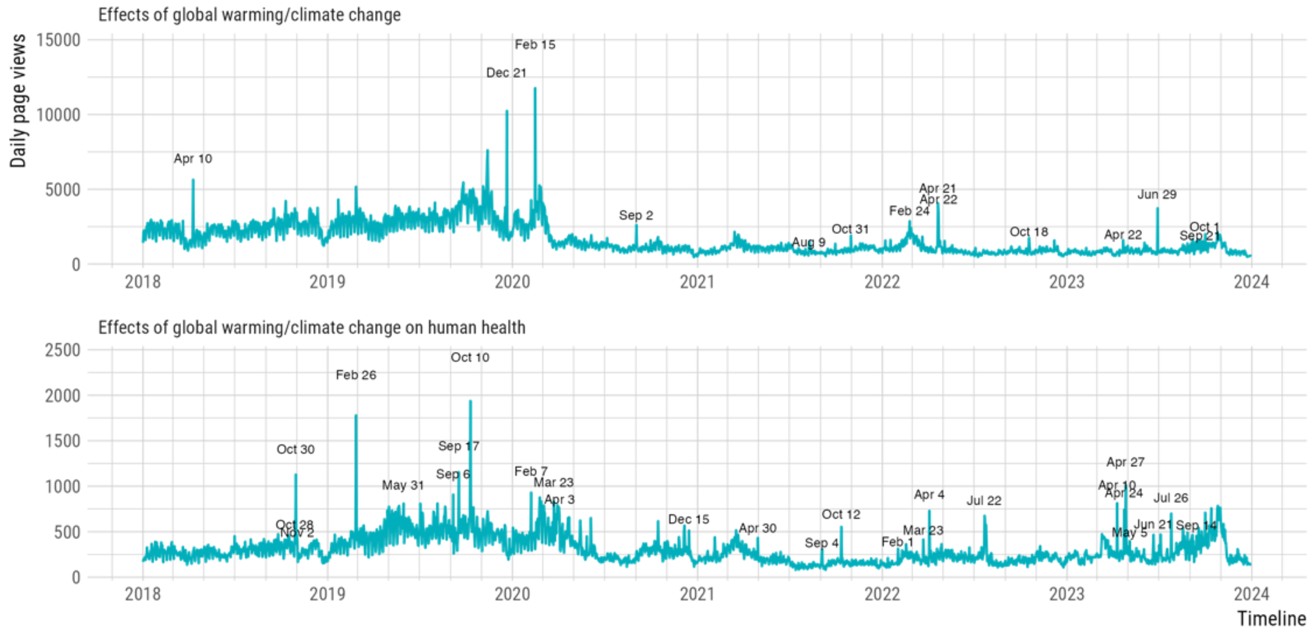

Figure 240: Daily page views 2018-2023 for Wikipedia articles directly related to the effects of climate change in general and on human health.

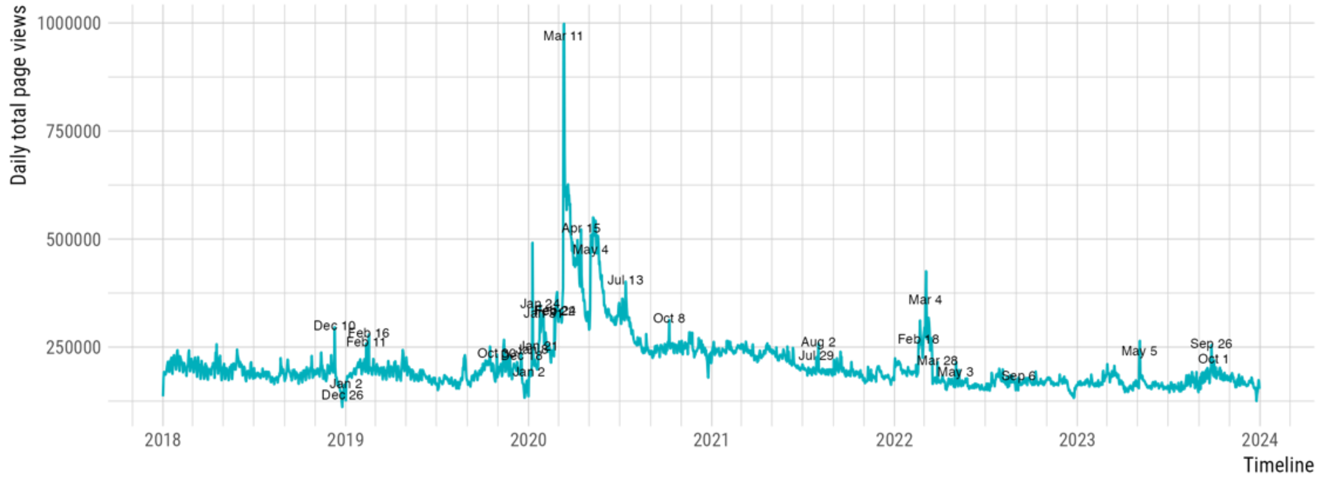

Figure 241: Aggregate daily page views 2018 to 2023 for all 1,276 selected articles on the English Wikipedia related to health.

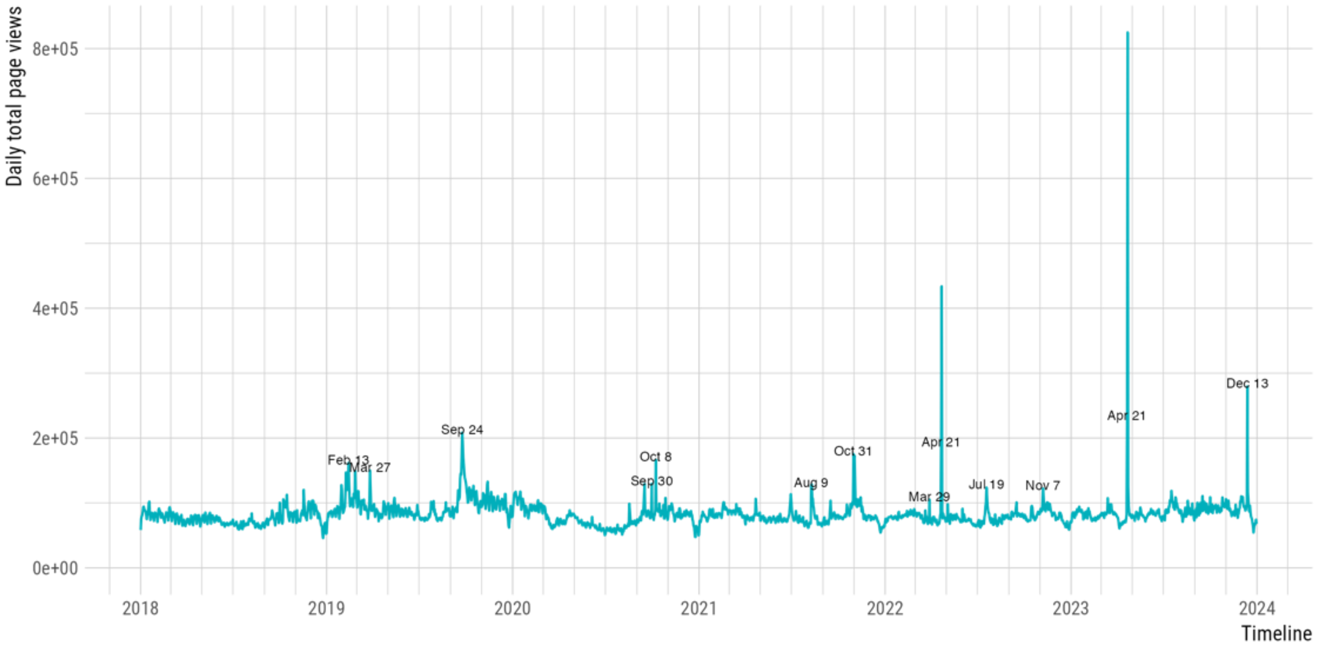

Figure 242: Aggregate daily page views 2018 to 2023 for all 942 selected articles on the English Wikipedia related to climate change.

5.3: Scientific engagement with health and climate change

Indicator 5.3.1: scientific articles on health and climate change 1990–2022

Indicator authors

Dr Max Callaghan, Prof Jan C. Minx

Scientific engagement in health and climate change is central to the *Lancet* Countdown mission: this is to facilitate, support and track progress on health and climate change. Scientific evidence is the major resource on which such progress rests; it also informs engagement in the key domains of global action, including the public, governmental and corporate domains. Engagement is quantified in the topic of climate change and health by tracking the number of publications over time. Using a machine-learning assisted approach,[453] relevant literature was identified and classified according to its subject.

Methods

The analysis pipeline of a recent study of the literature on climate and health was replicated.[453] First the search query is repeated, reproduced below. In a change to the previous indicator which used the databases Scopus, Medline, and the Web of Science Core Collection, the open source database OpenAlex was searched. The aforementioned study screened 3,730 documents by hand at the title and abstract level, labelling whether relevant documents were related to:

- The impacts of climate change on health;
- The effect on health of actions to mitigate climate change; or
- The effect on health of actions to adapt to climate change

Relevant documents were those which were indexed in English; provided a clear link to actual, projected, or perceived impacts of climate change, responses to reduce the impacts of climate change (adaptation), or the mitigation of greenhouse gas emissions; and included substantial focus on a perceived, experienced, or observed eligible health-related outcome or health system; and presented empirically driven research or a review of such research.

A support vector machine (SVM) classifier[454] was trained using document abstracts to reproduce the inclusion/exclusion decisions as well as the impacts/mitigation/adaptation labels. Classifier performance was evaluated using 10-fold cross-validation. The inclusion/exclusion classifier achieved an accuracy of 87.1% with 80% recall and 76% precision.

Here the same machine learning model was applied to classify new studies which were not available when the original paper was produced. Those studies predicted to be irrelevant were discarded. Then the multilabel impacts/mitigation/adaptation classifier was applied to those documents which were included. Documents can be classified as belonging to one or more of the noted categories.

Finally, a neural network based "geoparser",[455] was applied to titles and abstracts of the texts to extract the geographical entities mentioned in all texts. In this version of the indicator, an updated version of the geoparser was used compared to previous editions. These locations were allocated to countries, and then to WHO regions. Country names were also extracted from the institutional affiliations recorded by the bibliographic databases.

Data

This indicator uses data from bibliographic records in the online scientific database OpenAlex.

Future form of Indicator

In future editions of the indicator, it is planned to update the classifier after annotating additional documents (currently in progress). It is also proposed to train a new classifier, by fine-tuning a BERT-based model,[456] which has shown to achieve higher accuracy when classifying scientific literature.[457]

Additional Analysis

There is a large scientific literature on climate and health, comprising approximately 33,000 publications. In 2023, the number of publications increased compared to the previous year by 1.4%, reaching a figure of 3,678 papers, which is 0.03% lower than the maximum recorded in 2021 (Figure 243).

GHG emissions from fossil fuel combustion are the central driver behind observed and projected global warming and its devastating consequences for the health of people and planet. But only 4% of the scientific literature on climate change and health mention fossil fuels explicitly in the title or abstract (Figure 244). This low figure — which has remained steady over time — is because most scientific studies leave out fossil energy sources and start later in the causal chain with emissions, concentrations or warming to understand climate impacts and their consequences for human health. Hence, less than 1% of the studies on health impacts of climate change and health links to adaptation options mention fossil fuels. In contrast, 28% of studies on mitigating climate change directly mention fossil fuels, as harms to human health are often linked to co-pollutants of combustion processes (Figure 245).

The fossil fuels most commonly mentioned in the climate and health literature are coal and diesel. There is a large literature which looks at local air pollution in conjunction with these fossil fuels, as well on death and mortality, infectious diseases, child health, and asthma (Figure 246).

In terms of regional coverage, the largest number of papers on fossil fuels mention locations in Asia, followed by North America and Europe. Coal — one of the dirtiest fossil fuels — was the most commonly mentioned fossil fuel across all regions, but accounts for a larger proportion of research in Asia than in other regions (Figure 247).

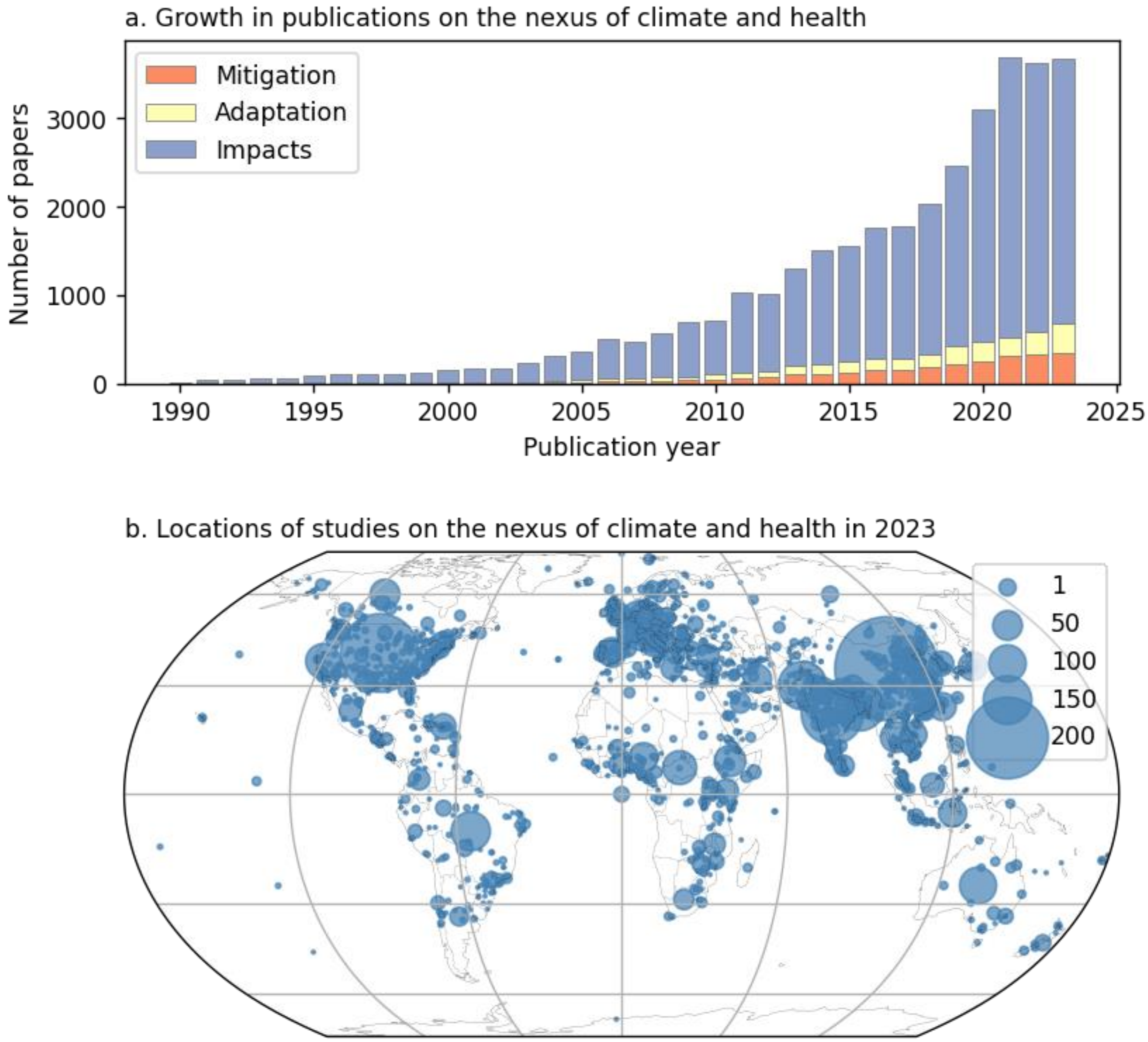

Figure 248: The number of scientific papers by year (a). Papers are allocated to the category which contains the highest prediction. The number of studies in each location (b).

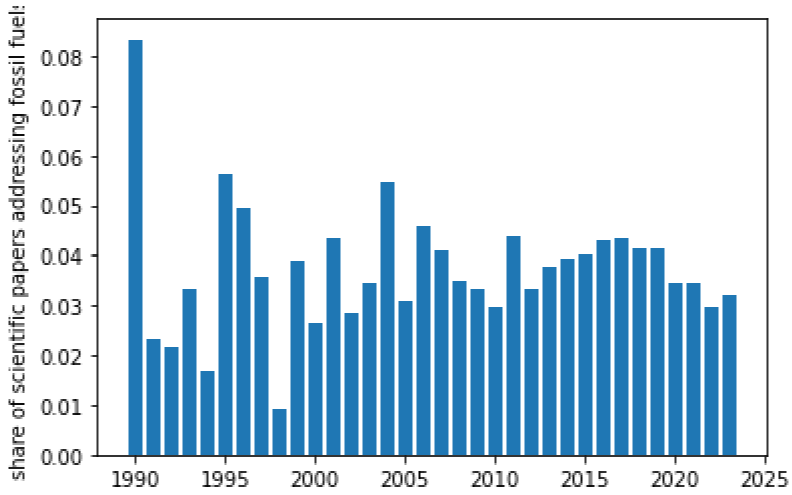

Figure 249: The proportion of literature mentioning fossil fuels in the title or abstract.

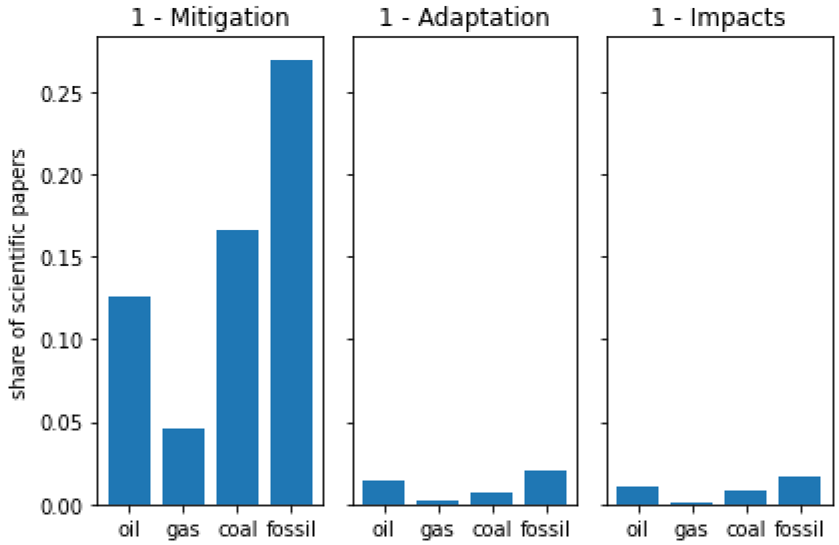

Figure 250: The proportion of literature from each category that mentions each fuel in the title or abstract.

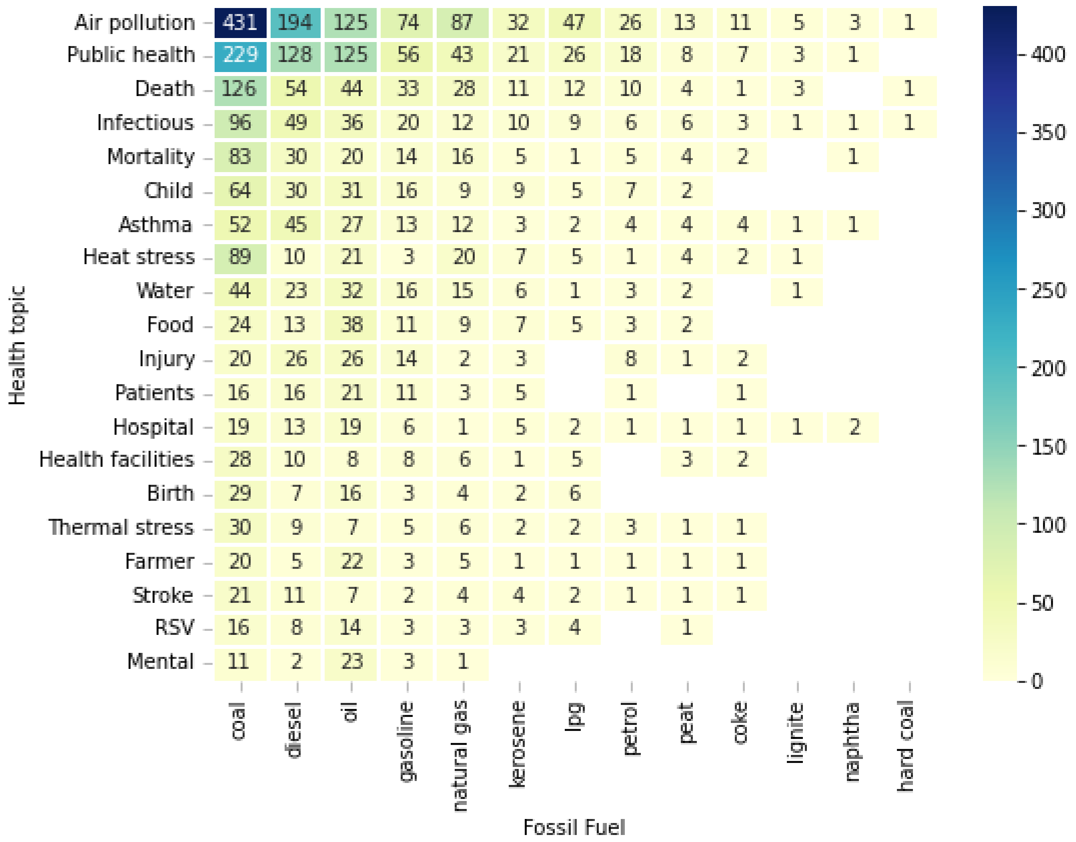

Figure 251: The number of papers mentioning each fuel in each health impact topic

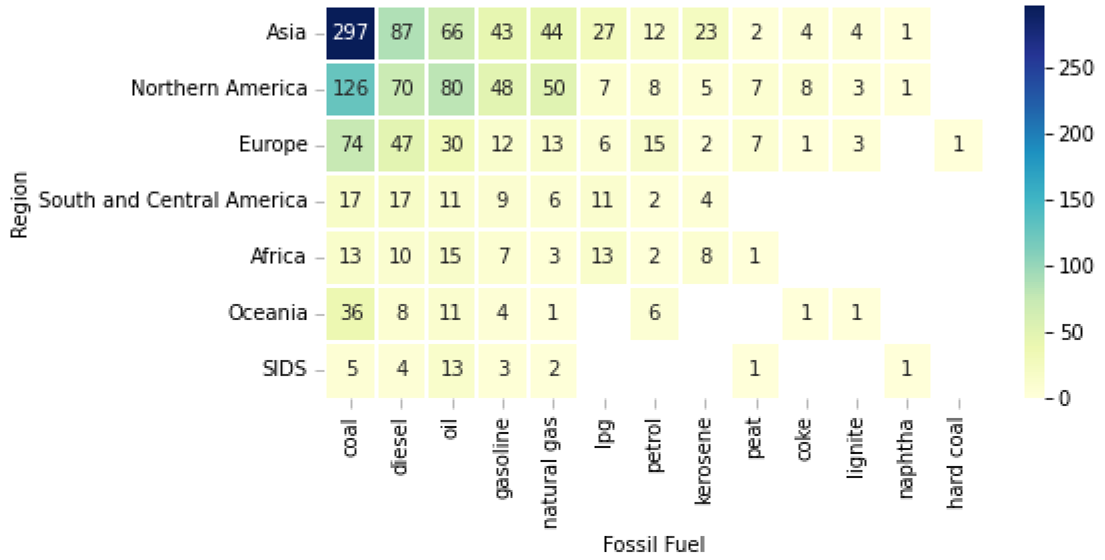

Figure 252: The number of papers mentioning each fuel in each region

Indicator 5.3.2: scientific engagement on the health impacts of climate change

Indicator authors

Dr Max Callaghan, Prof Jan C. Minx

Methods

There are multiple pathways in which climate and health outcomes are interlinked. A large and growing literature maps out the ways climate can impact human health, without necessarily isolating the role of human influence on the climate in driving the impacts. This indicator identifies this literature, classifying studies according to the climate drivers and health impacts studied, and locating where such impacts are studied. Using observational data and climate models, it identifies the subset of the literature which finds health impacts driven by climate variables, that are located in areas where changes in those variables can be attributed to human influence on the climate. These studies are referred to here as partially attributable impact studies.

Scientific studies form the basis of our understanding of how climate change and health are linked. Indicator 5.3 already identifies and tracks studies which provide evidence on climate and health. However, for those studies which address the impacts of climate on health, which form the majority of the evidence base, the studies themselves do not necessarily address the full chain of links between anthropogenic climate change, changes in a climate variable, and health impacts. Many focus only on the links between the latter two concepts. A novel approach is followed here,[457] to link studies on climate impacts with data from climate models and the observational record, in order to shed light on attribution across the whole chain from human influence on the climate, to the health impacts of climate change. The result is a new cross-working-group indicator that characterizes the available evidence from scientific studies on attributable climate impacts on human health.

The indicator starts from data from indicator 5.3, which identifies the relevant scientific literature on climate and health. Machine-learning classifiers from natural language processing were trained and applied to identify and classify a subset of documents with regard to the climate driver, the specific type of health impact, and the type of evidence provided as well as the time and location of the impact. By combining this geo-referenced set of documented climate-related health impacts with grid-cell-level human-attributable changes in temperature and precipitation, it is possible to provide a comprehensive evidence base on attributable health impacts, which is the basis for the indicator calculations.

The open access database Open Alex was searched for documents related to climate and health using a query developed in Berrang-Ford et al.[453] Two thousand examples were screened, including where documents mentioned a climate variable or extreme event AND a health impact or exposure, as defined in the *Lancet* Countdown's model of climate and health linkages. Reading the abstracts, the climate variable, the extreme event, the health impact, the exposure, and the attribution type were coded. Each document was double coded, with all inconsistencies resolved manually in discussion with a third coder if necessary.

A machine learning classifier was subsequently trained to reproduce the screening and coding decisions from the initial training sample. For each task, hyperparameters were optimised using tree-structured Parzen estimator[458] and nested cross-validation, testing three different transformer-based models. The results of the optimisation process are reported for each model and each task below.

	Category	bert-tiny	climatebert	distilroberta-base	scincl
**0**	Relevance	0.44 (0.29)	**0.85 (0.0082)**	0.82 (0.017)	0.84 (0.016)
**1**	Climate driver	0.54 (0.0048)	0.44 (0.23)	0.55 (0.0098)	**0.61 (0.062)**
**2**	Health impact	0.21 (0.11)	0.59 (0.044)	0.58 (0.013)	**0.68 (0.058)**
**3**	Attribution type	0.13 (0.027)	0.58 (0.047)	0.5 (0.047)	**0.6 (0.018)**
**4**	Extreme event	0.012 (0.017)	**0.73 (0.071)**	0.71 (0.09)	0.66 (0.014)
**5**	Exposure	0.0019 (0.0027)	0.36 (0.11)	0.36 (0.11)	**0.51 (0.14)**

Table 135: F1 scores (the mean of precision and recall) for each classifier and task

Trends in temperature and precipitation in grid cells are attributed following the procedure from Knutson et al.[459] and Knutson and Zeng.[460] As in Callaghan et al.,[457] this indicator shows where impact studies coincide geographically with attributable trends in the climate driver investigated. Study locations were extracted using the Mordecai 3 geoparser.[461] In the previous version of the indicator, an older version of the geoparser was used, making the figures not directly comparable.[455]

Caveats

It is important to acknowledge that although English is the *lingua franca* in scientific literature, searching for studies in English may result in additional gaps in coverage, which it is hoped can be addressed with multilingual searches in future versions of the indicator.

The method here cannot fully attribute the health outcomes identified in each study to human influence on the climate. Rather, it shows where the health outcomes of changes in climate variables coincide geographically with changes in those variables that can be attributed to human influence.

Additional Information

There is a large literature that investigates the impacts of climate on health, although the role of human-induced climate change is not always addressed in each study. 31,254 publications on the impacts of climate on health were identified, of which 21,509 investigate how either temperature or precipitation have influenced health outcomes (68.8%). By comparing observed trends in temperature and precipitation with model-based counterfactuals exploring a world without anthropogenic forcing and with climate model runs that model the effects of anthropogenic forcing,[459,460] the indicator demonstrates where trends in temperature and precipitation can be attributed to human influence on the climate. 16,708 of the studies showing impacts driven by changes in temperature or precipitation cover geographies where at least 50% of the area has been exposed to trends in the respective climate variable which can be attributed to human influence (Figure 253).

There is a large geographic variability in the number of studies, which is not proportional to the number of people exposed to trends in climate variables that can be attributed to human influence (Figure 254). For instance, there are 22 million people in Oceania living in 2.5 degree grid cells where trends in either temperature or precipitation can be attributed to human influence on the climate, and 1,139 climate and health studies focussing on a location in which the climate variable driving the impact can be attributed to human influence for over 50% of the area. That is a ratio of 49.8 studies per million exposed people.

In North America, Europe, and South America the ratios of studies per million people stand at 13.4, 6.6, and 4.0 respectively. Asia has 7,481 studies for its 4 billion inhabitants exposed to attributable trends in temperature or precipitation, a ratio of 1.8 per million, while Africa displays a ratio of 2.0 per million.

The disparity highlights the unequal access to resources across the world and shows that there are many areas exposed to climate change and vulnerable to its effects, of which there is little knowledge in the scientific literature.

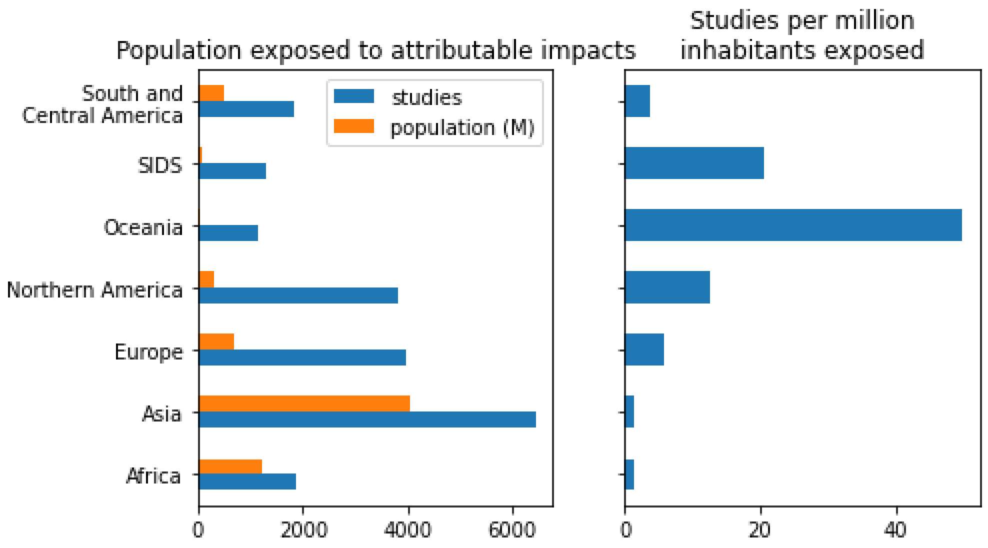

Figure 255: The number of people in each Lancet Countdown region exposed to trends in temperature or precipitation that can be attributed to human influence on the climate, compared to the number of studies exploring health impacts of climate drivers where those drivers display attributable trends.

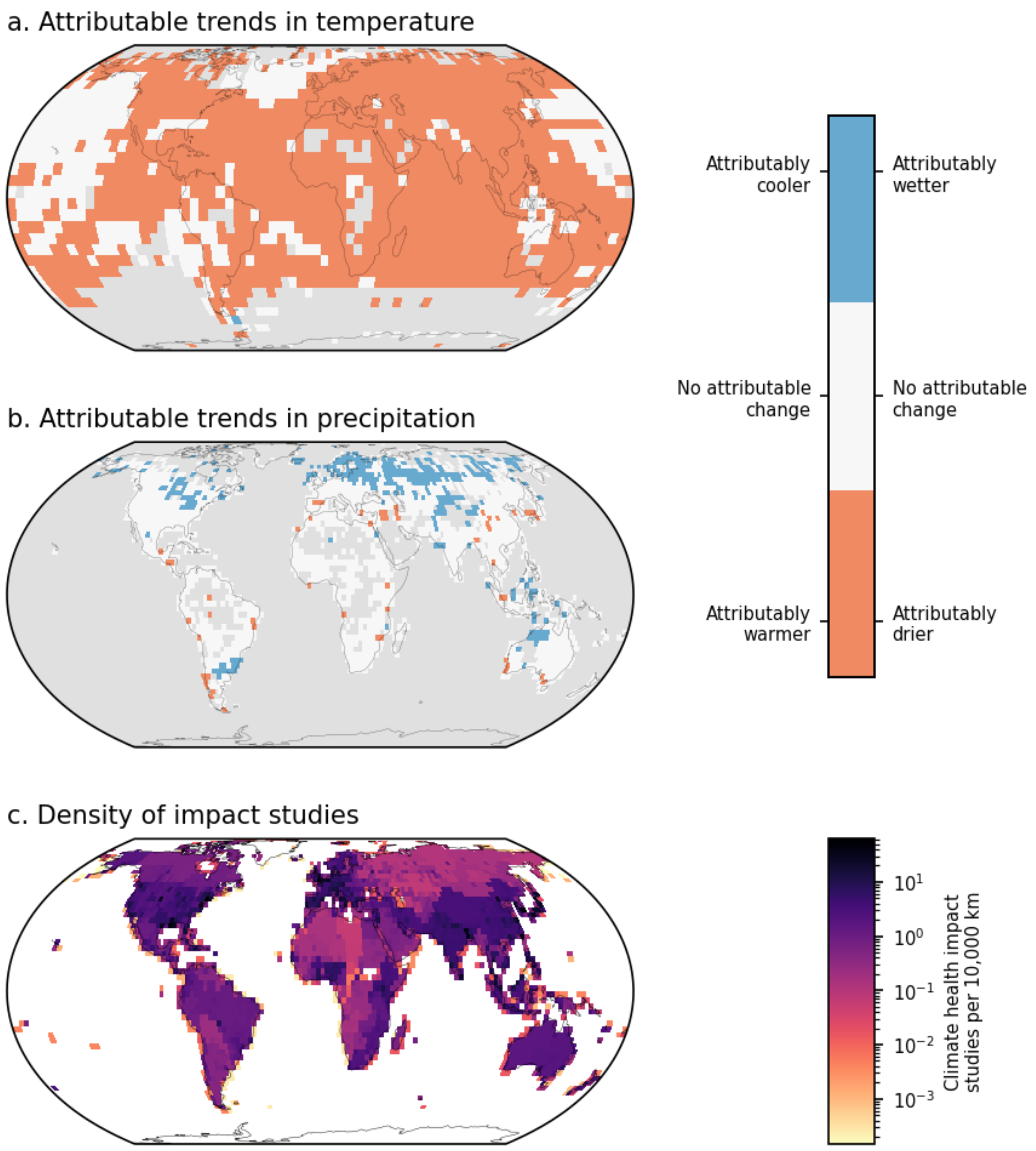

Figure 256: Attributable trends in temperature (top), precipitation (middle), and the number of climate health impact studies weighted per cell (bottom)

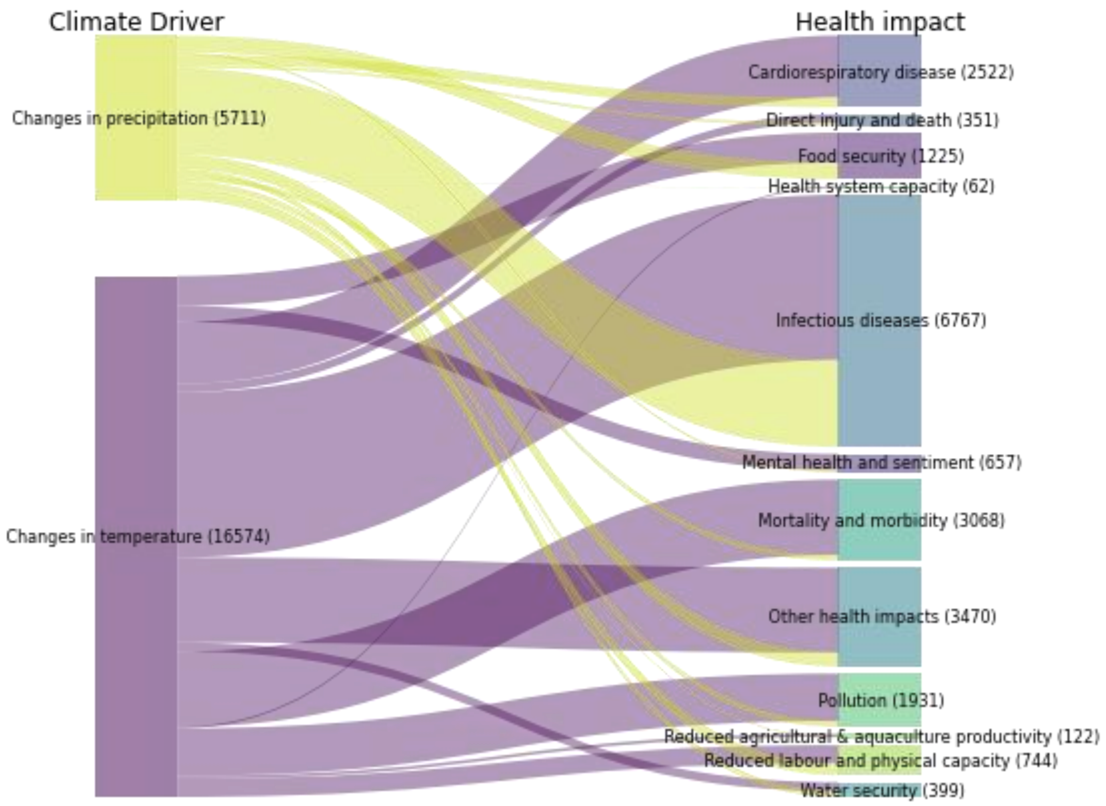

Figure 257: The number of papers exploring the links between temperature and precipitation and health outcomes, where the study focuses on area where trends in the relevant variable can be attributed to human influence on the climate. Note that single studies may investigate multiple drivers or health impacts.

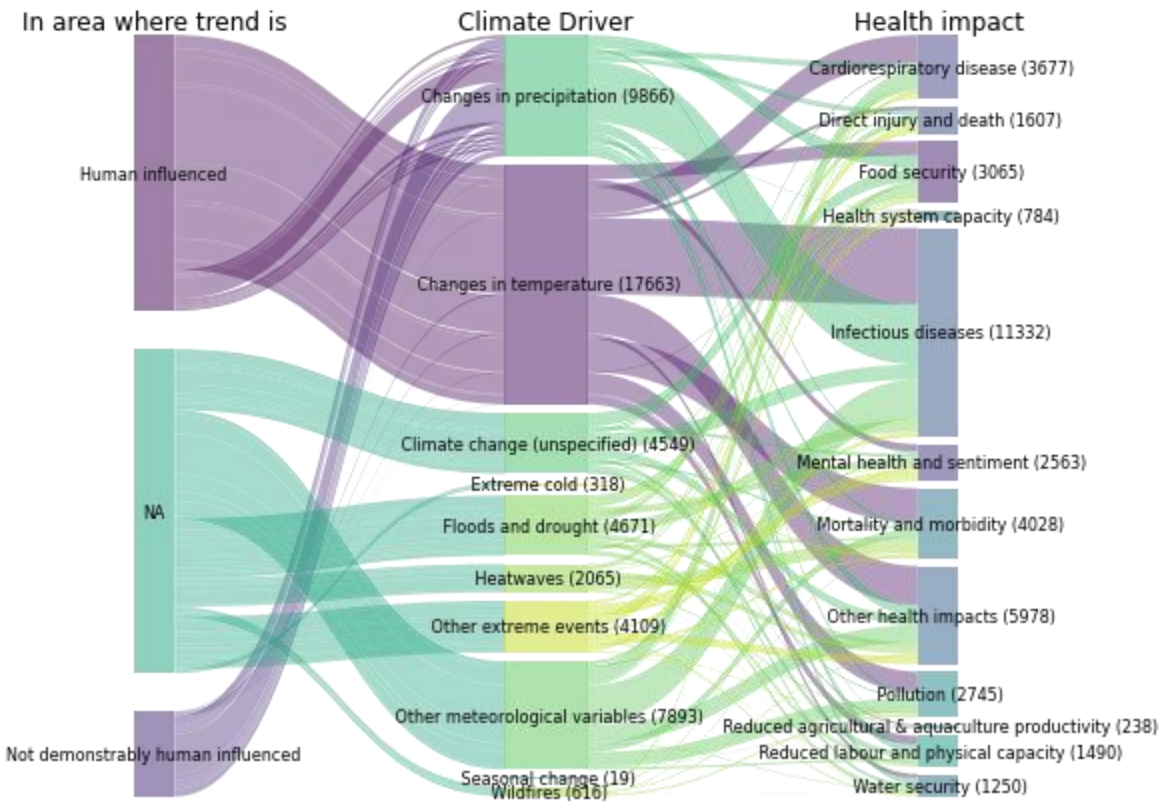

Figure 258: *The number of papers addressing links between climate drivers and health impacts.*

5.4: Political engagement with health and climate change

Indicator 5.4.1: government engagement

Indicator authors

Dr Niheer Dasandi, Prof Slava Jankin, Dr Pete Lampard

Methods

To produce the measure of high-level political engagement with climate change and health in the UN General Assembly, a new dataset of UN General Debate statements was used, which is discussed below. The approach to using UNGD statements to produce the indicators here is based on the application of natural language processing to the corpus of UNGD statements. References to key search terms linked to (a) health, and (b) climate change are identified:

- **Health:** malaria, diarrhoea, infection, disease, diseases, sars, measles, pneumonia, epidemic, epidemics, pandemic, pandemics, epidemiology, healthcare, health, mortality, morbidity, nutrition, illness, illnesses, ncd, ncds, air pollution, nutrition, malnutrition, malnourishment, mental disorder, mental disorders, stunting.
- **Climate change:** climate change, changing climate, climate emergency, climate action, climate crisis, climate decay, global warming , green house, temperature, extreme weather, global environmental change, climate variability, greenhouse, greenhouse-gas, low carbon, ghge, ghges, renewable energy, carbon emission,

carbon emissions, carbon dioxide, carbon-dioxide , co2 emission, co2 emissions, climate pollutant, climate pollutants, decarbonization, decarbonisation, carbon neutral, carbon-neutral, carbon neutrality, climate neutrality, net-zero, net zero.

These key terms have been updated to reflect the changing terminology used to discuss climate change. In order to produce an indicator of engagement with the intersection of climate change and health, this approach focused on whether any of the climate change related terms appeared immediately before or after any health terms in the GD statements. This was based on a search of the 25 words before and after a reference to a health-related term. The choice of 25-word window context corresponds to approximately half a paragraph of text. Given that UNGD statements are highly structured and methodically developed by governments over prolonged periods of time, it is assumed that half a paragraph of text around public health terms captures a sufficiently narrow context. The number of climate change term references were counted in these contexts to produce the measure of engagement with the link between health and climate change. A robustness analysis – varying the size of the context (5, 10, and 50 words) – was also undertaken. This substantively produced the same trends over time. A sample of the references produced by the search were also examined as an additional check to ensure that the references identified reflect engagement with the health impacts of climate change.

Data

This indicator draws on a new and updated dataset of GD statements: *the United Nations General Debate corpus*, in which the annual GD statements have been pre-processed and prepared for the application of natural language processing to the official English versions of the statements.[462] The dataset contains all the country speeches made in the UN General Debate between 1970 and 2023. Table *136* presents summary of the data by year:

Table 137: Summary information for UN General Debate Corpus

Year	General Debate statements	Total sentences	Total words
**1970**	70	11854	303771
**1971**	116	19901	508495
**1972**	125	21201	540958
**1973**	120	21450	536411
**1974**	129	22041	568610
**1975**	126	21365	534339
**1976**	134	23799	599926
**1977**	140	24799	605742
**1978**	141	25236	625310
**1979**	144	26462	651961
**1980**	149	27191	657546
**1981**	145	26063	633521
**1982**	147	23435	637957
**1983**	149	26803	641492
**1984**	150	27928	660382
**1985**	137	19259	592596

**1986**	149	19030	577503
**1987**	152	18336	563066
**1988**	154	18595	569486
**1989**	153	19440	574358
**1990**	156	17885	522192
**1991**	162	18552	538349
**1992**	167	18597	543138
**1993**	175	20165	587437
**1994**	178	19944	580525
**1995**	172	17870	536740
**1996**	181	18046	522695
**1997**	176	17701	514492
**1998**	181	18883	514836
**1999**	181	18529	531291
**2000**	178	16259	464312
**2001**	189	14748	414681
**2002**	188	13977	380481
**2003**	189	14716	399396
**2004**	192	14899	405290
**2005**	185	13012	353065
**2006**	193	14646	390476
**2007**	191	14586	387883
**2008**	192	14294	384880
**2009**	193	16029	423395
**2010**	189	14439	391946
**2011**	194	16293	429974
**2012**	195	16837	444516
**2013**	193	16400	440893
**2014**	194	15859	421945
**2015**	193	16129	436361
**2016**	194	15990	420148
**2017**	196	16806	439621
**2018**	196	16980	455195
**2019**	195	17526	466108
**2020**	193	15165	396529
**2021**	194	16679	442378
**2022**	193	17240	448085
**2023**	192	18270	438187

The data was pre-processed for analysis by removing punctuation, symbols, numbers, stop words, and URLs. In addition, all tokens were normalised (lower-cased). All pre-processing and analysis was carried out in R using the "quanteda" package.[463]

Caveats

The search for climate change terms in the context of public health references is a proxy for the semantic linkage between the two sets of terms in GD statements. This approach produces a scalable and reproducible measure with a high degree of reliability that does not involve human judgement or subjective biases. However, there may be examples of governments referring to climate change and health but not the direct linkages between the two, which are included in the count; and there may be examples of governments discussing the health impacts of climate change in their UNGD statements, which are not included in the measure because the distance between the mention of the climate change term and the health term exceeds 25 words. Based on an analysing a sample of the speeches and references, such cases are relatively rare and do not have a significant bearing on the indicator or the trends uncovered.

It is also worth noting that the analysis here is based on a narrow range of search terms, which excludes reference to many of indirect links between climate change and health. A number of GD statements in this time period refer to such indirect connections, such as the effects of climate change on water and agriculture – however, these are not included here. Therefore, the results present a somewhat conservative estimate of high-level political engagement with the intersection of climate change and health. Future work in this area will consider engagement with these indirect links.

Future Form of Indicator

In the future, it is planned that this indicator will look more closely at the references to indirect links between climate change and health. For example, this would question the main ways in which governments view climate change impacting on health and whether this changes over time based on awareness of the multiple ways in which climate change and health are connected. Some of the references to the indirect links between climate change and health made in UNGD statements are highlighted in the main report.

Additional Analysis

Figure 259 shows the total number of references to health, climate change, and the intersection of the two between 1970 and 2023. Figure 260 shows the proportion of countries referencing health, climate change, and the intersection of the two in their addresses during the UN General Debate between 1970 and 2023. Figure 261 presents the total number of references to the intersection in UNGD statements between 1970 and 2023. Figure 262 shows the proportion of countries that engage with the intersection of climate change and health between 1970 and 2023. The figures show the substantial increase in engagement with the health dimensions of climate change that occurred in 2020 and 2021, with a slight decline in 2022 and 2023. In 2023, there were 68 individual references to the climate change-health intersection.

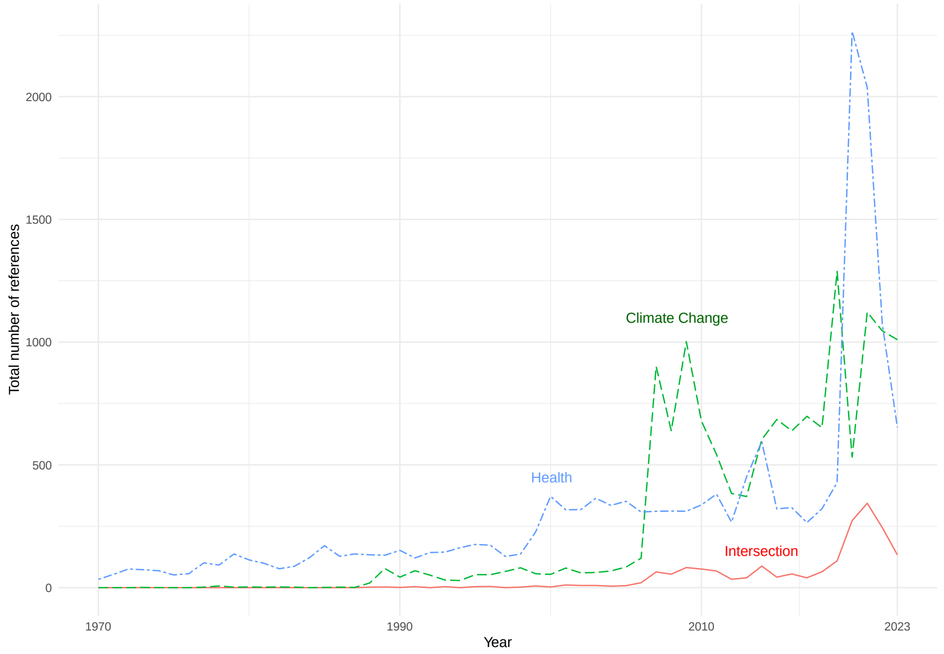

Figure 263: Total number of references to health, climate change, and intersection, 1970–2023

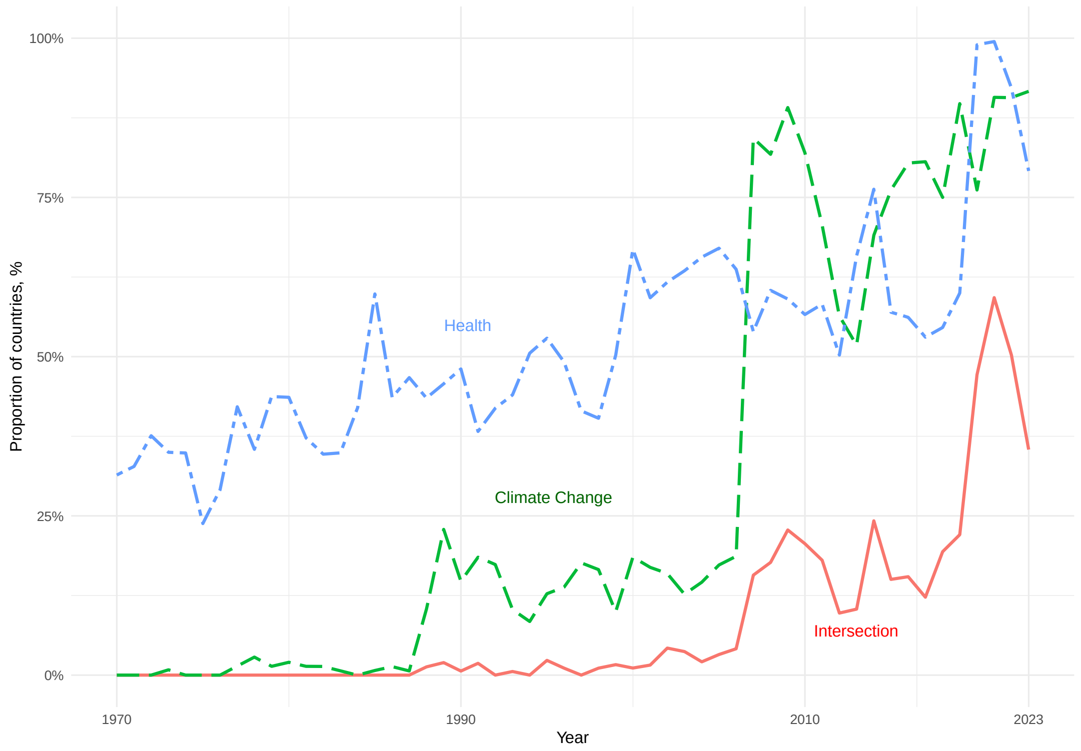

Figure 264: Proportion of countries referring to health, climate change, and the intersection of the two in UN General Debate addresses, 1970-2023.

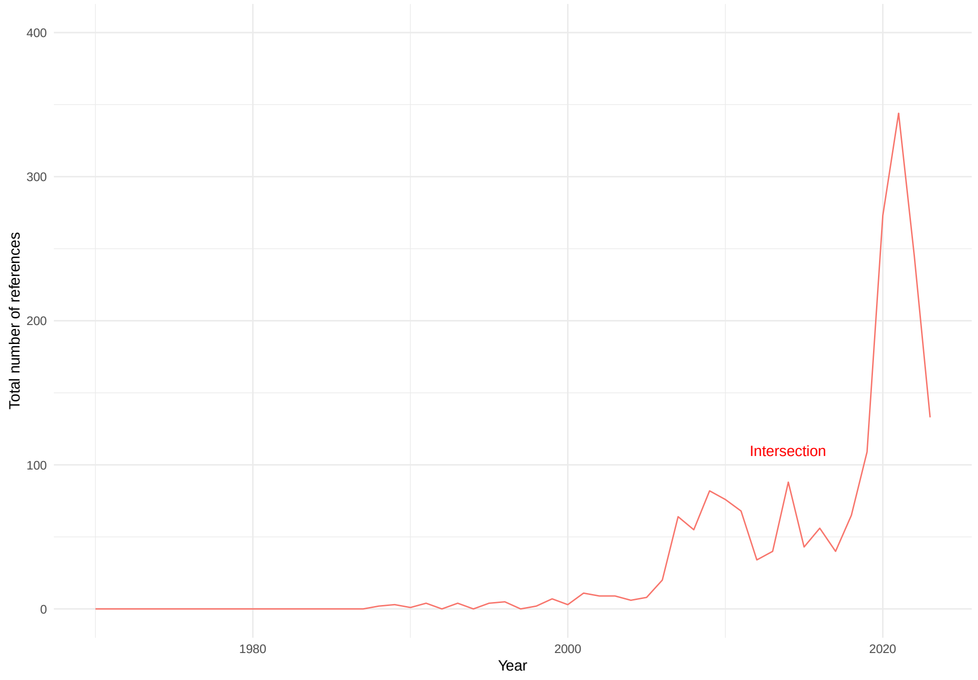

Figure 265: Total number of references to intersection, 1970–2023

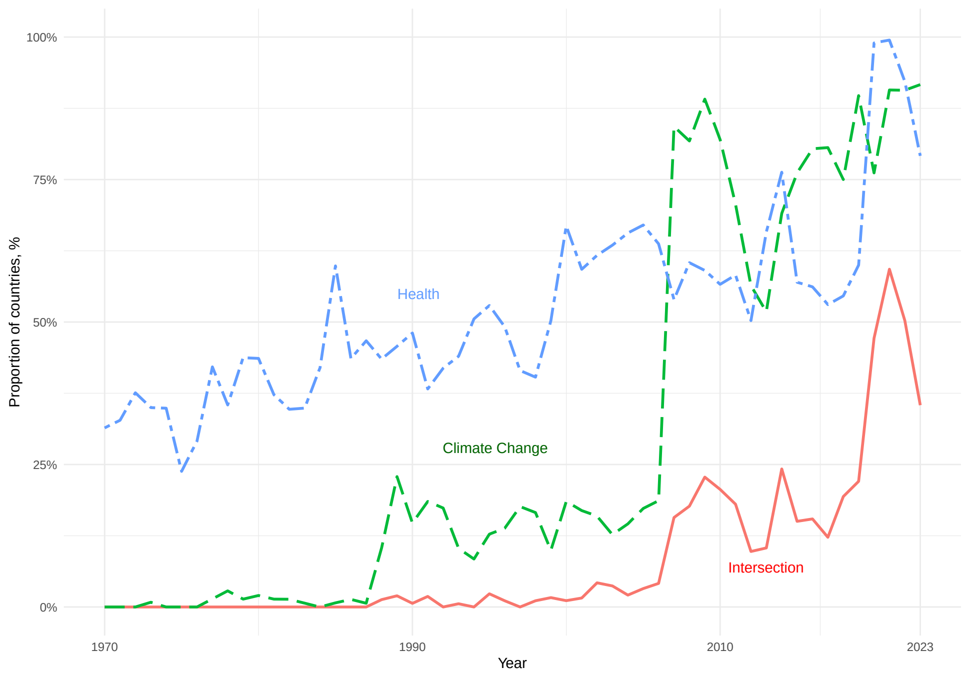

Figure 266: Proportion of countries referring to health, climate change, and the intersection of health and climate, 1970–2023

There is growing awareness of the gendered impacts of climate change and health. The extent to which references to the health dimensions of climate change in countries UN General Debate statements engage with gender issues are considered by further examining the references to the intersection of

climate change and health. Once all the references are identified to this intersection in UNGD statements for 1970–2023, additional search terms are used related to gender to identify which of the intersection references also engaged with gender issues. The gender-related search terms used were as follows: *women, women's, maternal, inequality, inequalities, gender, empowerment, sex, sexual, violence, violent, girls, reproduction, reproductive*. Hence, the analysis considers whether the 25 words of text identified in the primary search (for climate change and health terms) includes a reference to at least one of these gender-related keywords. Figure 267 shows that in 2023 6% of all references to the intersection of climate change and health also include a mention of gender. The figure shows that this is lower than in previous years, with the 2014 seeing 26% of all climate change-health references including a gender mention.

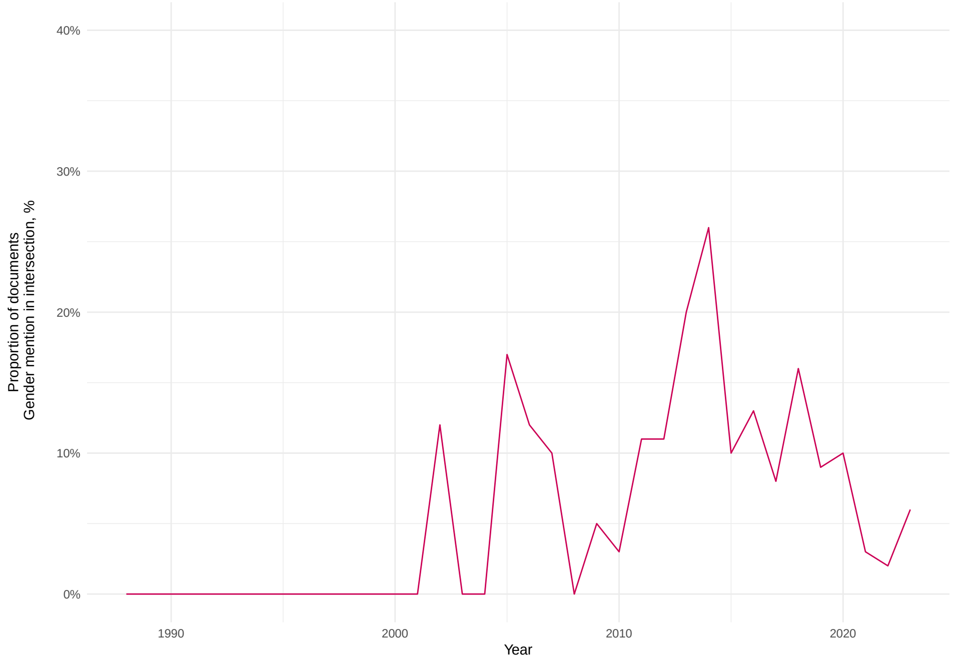

Figure 268: Proportion of references to the intersection of health and climate change that include a reference to gender, 1970–2023.

Figure 269 below presents the proportion of countries that engage with the intersection of climate change and health by WHO region. There has been a significant increase in engagement with health and climate change across all regions in the past three years. In 2023, there is a slight decline across most regions compared to 2022, though at least 15% of countries in all of the regions refer to the health dimensions of climate change in their 2023 UNGD statements. As in previous years there is especially high engagement from countries in the Western Pacific region, with 60% of countries referring to the intersection of climate change and health. There is also an increase in the proportion of South-East Asian countries referring to health and climate change with 40% of countries referring to the intersection. It is worth noting that the relatively higher level of political engagement by countries in the Western Pacific is especially driven by the small island development states (SIDS) in this region. The lowest engagement is by countries in the European region with 15% of countries in this region referring to the intersection of climate change and health.

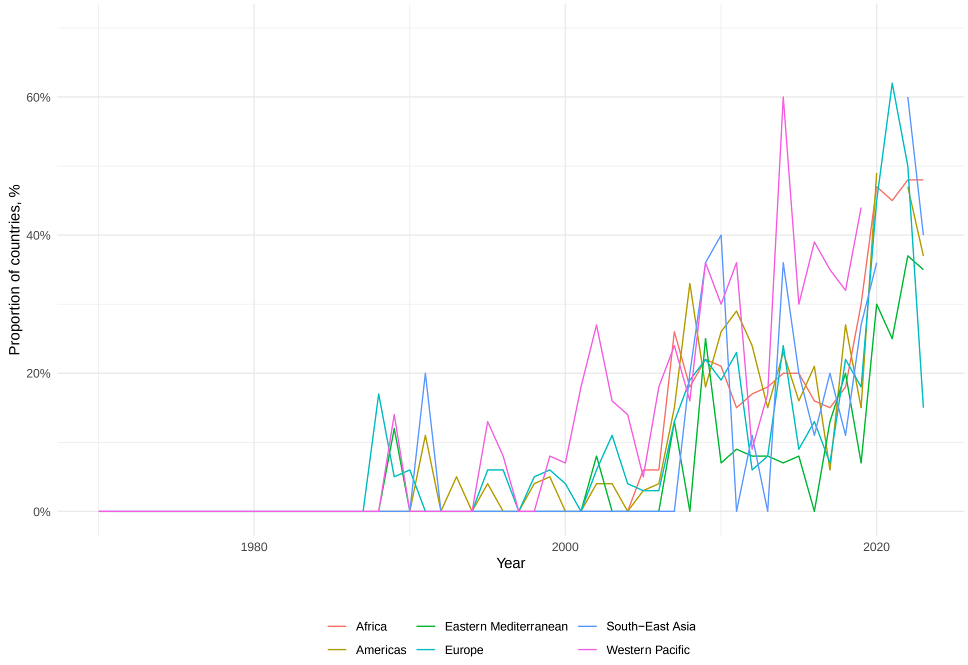

Figure 270: Proportion of countries referring to intersection of health and climate change by region, 1970–2023.

Figure 271, below, presents the total number of references to the climate change-health link between 1970 and 2023 by WHO region. The figure shows that the highest number of references to the intersection of climate change and health come from four regions: Africa, Latin America and the Caribbean, and the Western Pacific. In general, the figure suggests that there is lower engagement among countries in the Eastern Mediterranean, North America, South-East Asia, and Europe.

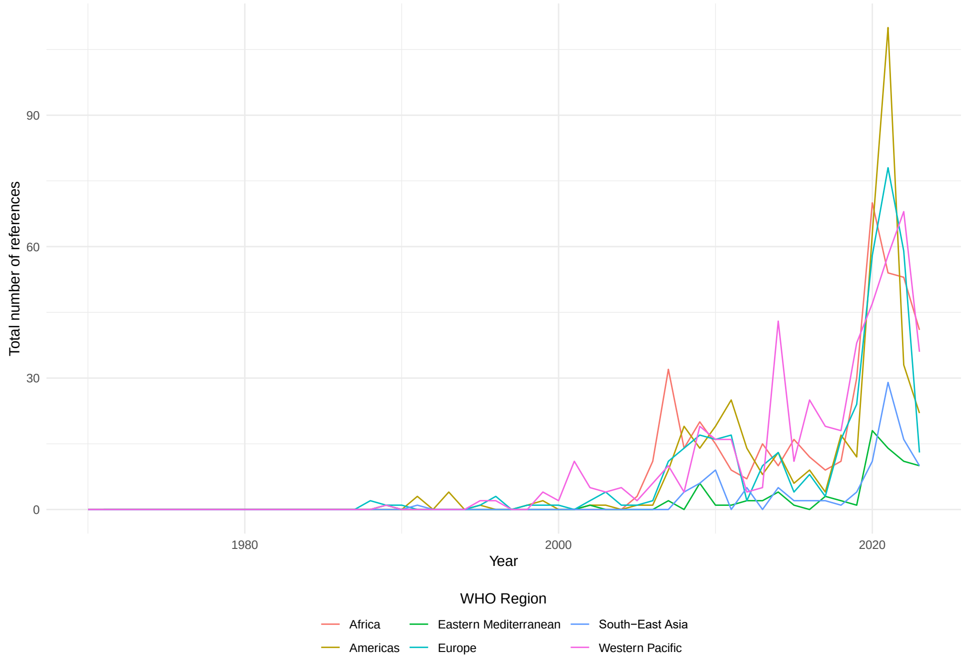

Figure 272: Total number of references to intersection by region, 1970–2023.

In addition to grouping countries by WHO region, countries were also considered by continental regions (Figure 273 and Figure 274). As noted in previous years' reports, the SIDS have driven much of the engagement with the health impacts of climate change, as well as climate change more generally, in the UN General Assembly. As such, a separate SIDS grouping is included.

Figure 275 shows the proportion of countries that engage with the intersection of climate change and health based on these country groupings. Figure 180 shows the total number of references to the climate change-health intersection according to these groupings. Both figures demonstrate the high level of engagement with the climate change-health linkages by SIDS. It is worth noting that some of the regions (e.g., Northern America and Oceania) contain very few countries, and hence they deviate in engagement between 0 and 100% in different years.

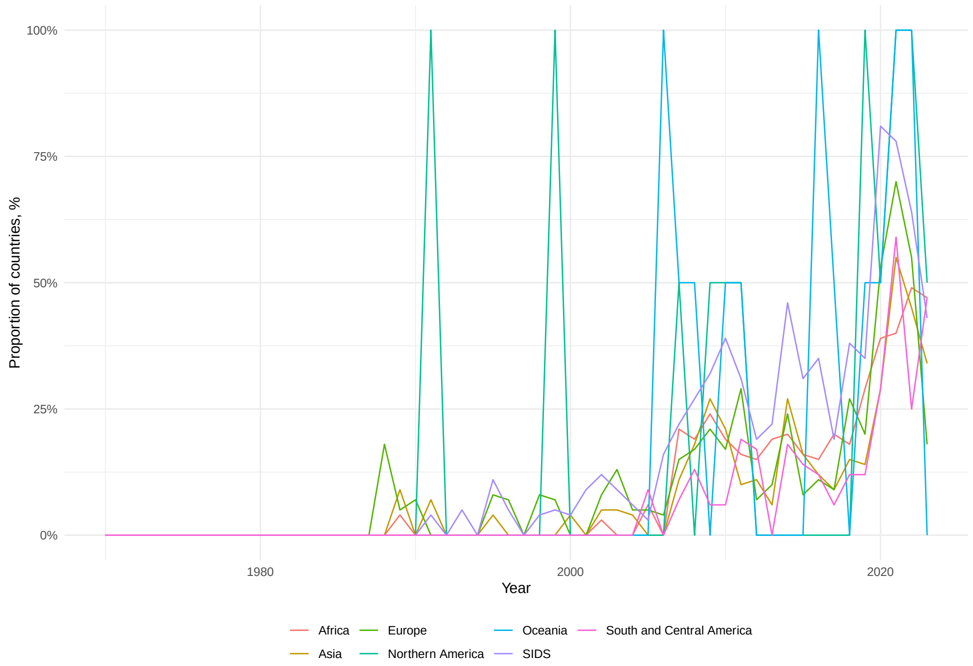

Figure 276: Proportion of countries referring to intersection of health and climate change by country grouping, 1970–2023.

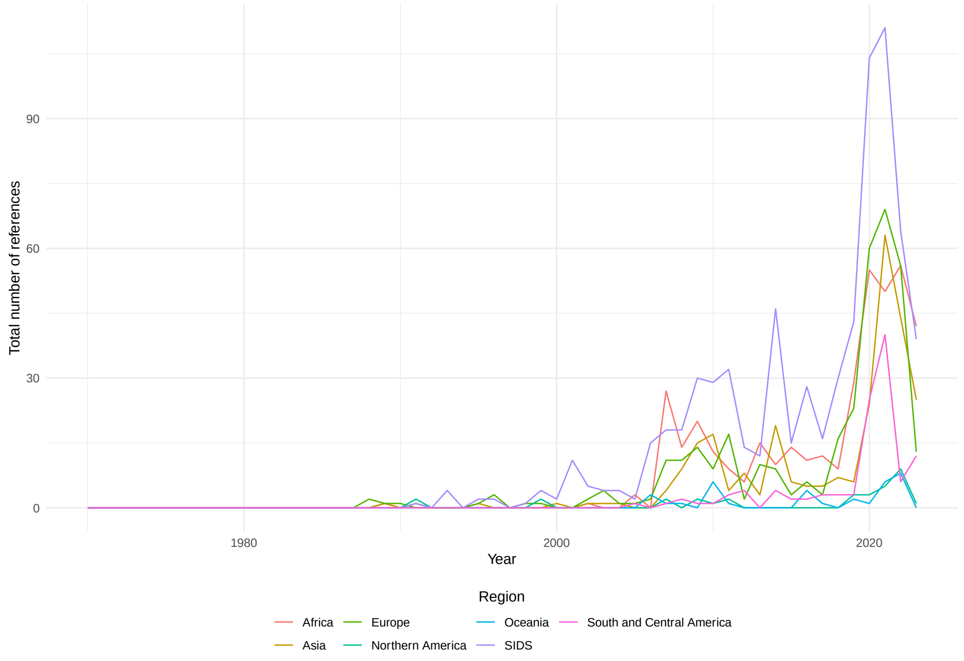

Figure 277: Total number of references to intersection by country grouping, 1970–2023.

Government engagement with the health dimensions of climate according to countries' Human Development Index (HDI) categories was also considered. Figure 278 shows the proportion of countries engaging with the intersection of climate change and health by HDI category, and Figure 279 shows the total number of references by countries' HDI categories. Both figures show that following a significant increase in engagement between 2019 and 2021, 2023 saw a second consecutive year of decline in engagement. The Medium HDI category has the highest engagement with the intersection of health and climate change in 2023.

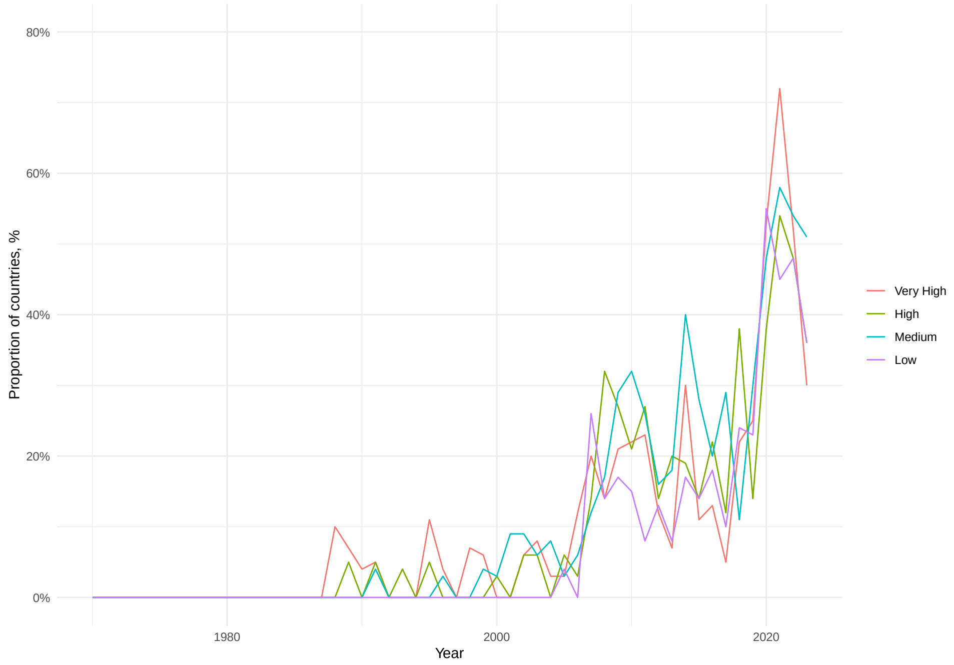

Figure 280: Proportion of countries referring to intersection of health and climate change by HDI categories, 1970–2023.

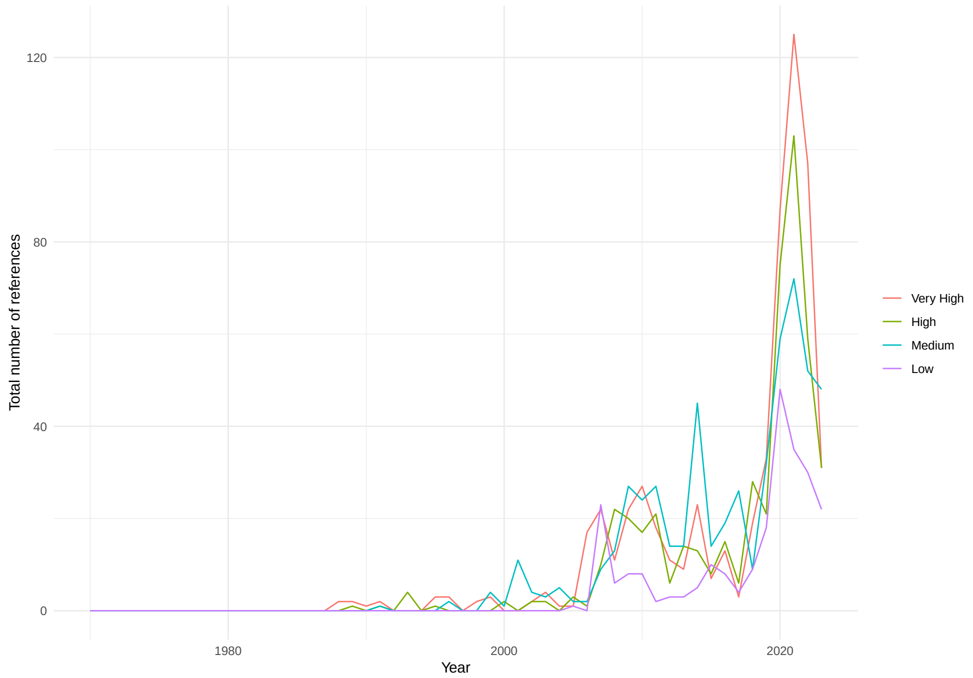

Figure 281: Total number of references to intersection by HDI categories, 1970–2023.

Figure 282 below presents a world map, which shows the countries that refer to the intersection of climate change and health in their 2023 UNGD statements, and the number of individual references they make. The map shows that half of all countries mentioned the intersection of health and climate change in their 2023 address. The map also shows that despite the higher engagement, it is evident a divide still exists between high-income countries on the one side, and low- and middle income countries on the other side – though this divide is much smaller than in previous years. The latter (low- and middle-income countries) tend to engage more with climate change and health, particularly when the

SIDS are included. Due to their size, the SIDS do not show up on the map. As preiously noted, the SIDS tend to be highly represented among nations engaging with the health-climate change links.

Figure 283 and Figure 284 present world maps, which show the countries that refer to public health and climate change respectively in their 2023 UNGD statements, as well as indicating the number of references made by each country. The figures demonstrate that there is considerable engagement with the issues of climate change and health separately. In 2023, 95% of countries referred to climate change and 82% of countries mentioned health in their UNGD statements, as can be seen in Figure 285and Figure 286. Figure 287 and Figure 288 show that as well as a much larger share of countries around the world discussing climate change and health in their GD statements compared to those discussing the intersection, there is also much deeper engagement with these two areas individually, in that countries tend to make a number of references to climate change and health in their GD statements.

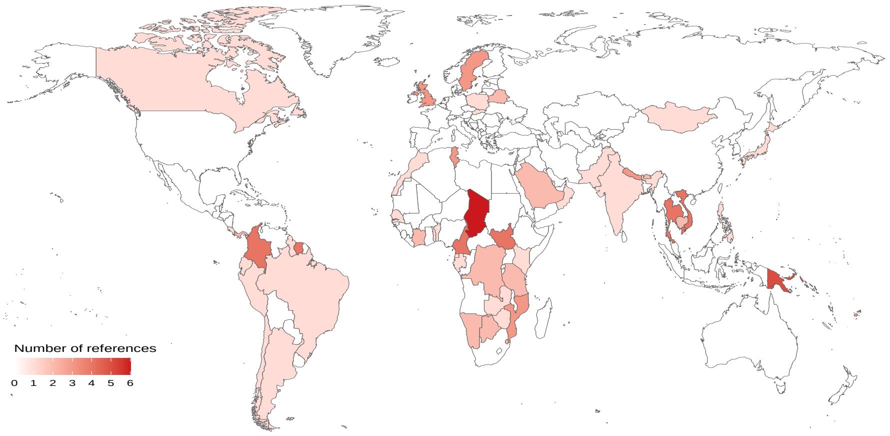

Figure 289: World map showing references to intersection of climate change and health, 2023.

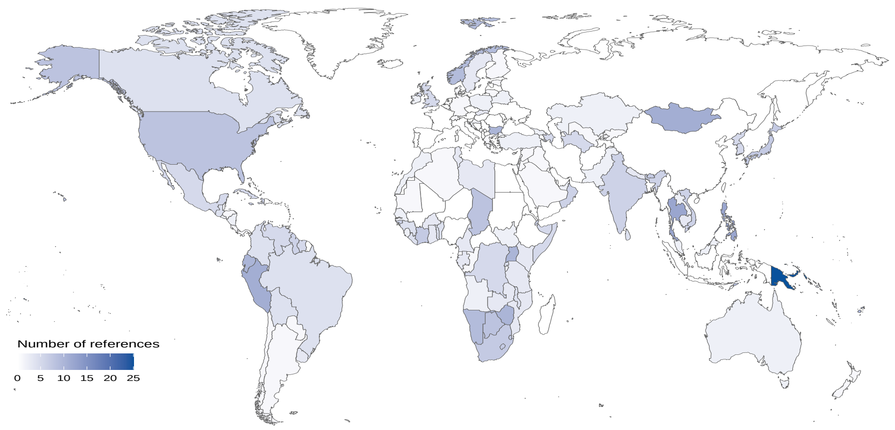

Figure 290: World map showing references to public health, 2023.

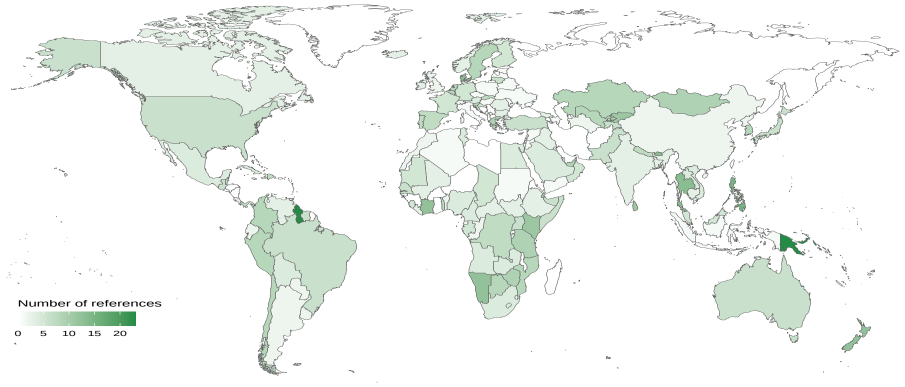

Figure 291: World map showing references to climate change, 2023.

Engagement in health and climate change in the Nationally Determined Contributions

Indicator Authors

Dr Pete Lampard

Methods

Under the Paris Agreement, Nationally Determined Contributions (NDCs) for each Party to the Agreement are communicated through the NDC registry. As a measure of engagement across climate change and health, and in particular of governments' appreciation of the health risks of climate change, all available first, second, third, and fourth NDCs (as of March 01, 2024) were analysed with respect to their inclusion of health-related terms.

Any updated or new NDC was regarded as a separate item. Most first NDCs were Intended Nationally Determined Contributions (iNDCs) ratified by the Paris Agreement into NDCs, though a small number were produced later. Analysis of first and second, and for some third and fourth, NDCs allows some indication of changes in climate-related health concerns over time.

All NDCs were downloaded from the NDC registry. These were organised by iteration as per the UNFCCC registry spreadsheet (https://unfccc.int/NDCREG). Importantly, the European Union NDC is standardised and is therefore used to represent multiple UN member states.

NDCs were uploaded to R using the Tabulizer package.[464] The Tesseract package[465] was also used for its OCR capabilities where NDCs were scanned PDFs. Where necessary, NDCs were also translated using Google Translate.

Health-related terms were developed in an iterative fashion, between climate-related health terms used in previous literature and the data.[466,467] Text referring to health directly, denoting either its presence (e.g., health, well-being) or its absence (e.g., death, illness, disease), was identified in the NDCs using text-based packages in R. The health terms can be seen in Table 138 below.

Appending country information to the results allowed aggregate data to be organised by iteration, year, *Lancet* Countdown grouping, HDI classification, and WHO region.

A sweep for false positives was taken on a number of NDCs in each iteration to ensure that search terms were not capturing irrelevant terms and that terms were not being double counted for any reason.

Table 139: Health keywords

category	keywords
health	health, illness
death	fatal*, mortal*, loss_of_life, death*
wellbeing	wellbeing
disease	infectious_disease*, disease*, morbid*, syndrome*
nutrition	malnutrition, starvation, undernutrition, nutrition
infectious disease	malaria, chikungunya, dengue, fever*, ebola, zika, leishmaniasis, leptospirosis, epidemic*, typhoid, vector*, aedes, mosquito*, pandemic*
psychological	emotion*, psychology*, mental_health
medical	medic*, hospital, hospitali*ation, patients, emergency_department, A&E, diagnos*, clinical
heat	heat_stress, heat_disorder*
injury	injur*
covid	covid, corona*, sars_cov_II, sars_cov_2

Data

Table 140 shows the number of NDCs available by year, while Table 141 shows the number available by iteration. Given that member states are expected to produce NDCs every five years, numbers are understandably higher in certain years (for example, many of the 2015 iNDCs were ratified in 2016, meaning higher numbers in 2020 and 2021).

Table 142: Number of NDCs available per year

Year	Number of NDCs
2015	185
2016	9
2017	4
2018	4
2019	3
2020	67
2021	91
2022	30
2023	34

Table 143 Number of NDCs available per iteration

Iteration	Number of NDCs
1	192
2	172
3	58
4	5

Caveats

This analysis treats updates to NDCs and completely new NDCs as iterations without distinction. While contents of updates or progress reporting may be different to completely new NDCs, this does not diminish the importance of health as a priority for reporting within either. Future analyses might make such a distinction, however.

Future form of indicator

This indicator uses the data from all available NDCs held on the UNFCCC registry (https://www4.unfccc.int/sites/NDCStaging/Pages/All.aspx) as of March 1st, 2024. Future reports will report on NDCs added after this date.

Future research could also provide a full document analysis looking at the role of health in the NDCs, but this would require further resourcing.

Additional analysis

Prevalence across iterations

Table 145 below shows the prevalence of different health keywords across the iterations of NDCs. It demonstrates that from the first to the second iteration of NDCs, there is an increase from 70% (135 of 192) to 94% (162 of 172) of NDCs using a health-related keyword. Following this, however, of the 58 third iteration NDCs, less than half (47%, 27 of 58) mention a health-related term. Four of the five (80%) fourth iterations counted in this analysis mention a health-related term.

The table also shows that the main driver of health terms is the word "health" itself. Keywords relating to deaths (often reporting numbers of fatalities or levels of mortality, for example), to disease (in a general sense), to specific infectious diseases, to nutrition, or to health or medical infrastructure all increased significantly in the second iterations. These dropped once again, however, in the third iterations. It is important to note that the kind of update will likely impact on inclusion, as some updates are about specific issues or updates on progress. That said, updates about health as a specific issue or about progress on climate-related health outcomes would be picked up in this analysis.

While Table 144 contains keyword relating to COVID19, these are not included in the overall health keyword total. COVID19 is frequently used from 2020 iterations onwards not just as a health concern, but as a contextual feature that member states have to take into consideration. For example, the Federated States of Micronesia second NDC outlines "[l]ike many countries, the FSM has been adversely affected by the Covid-19 pandemic". Similarly, the third NDC of Japan states: "*In light of the recognition that we are living in a major turning point of the era, what is required is not to simply return to the world before the COVID-19 pandemic, but to realize a transformation to a sustainable and resilient social system*".

Table 145: Health related terms and their distribution across NDC iterations. Note: COVID-19-related terms are not included in the 'all health' category.

	**1 (192)**	**2 (172)**	**3 (58)**	**4 (5)**
all health terms	70%	94%	47%	80%
health	67%	92%	43%	60%
illness	3%	10%	9%	20%
death	15%	35%	19%	20%
disease	39%	57%	22%	20%
wellbeing	19%	40%	24%	60%
nutrition	14%	38%	9%	0%
Infectious diseases	18%	31%	17%	0%
psychological	1%	5%	7%	20%
infrastructure	16%	40%	21%	20%
heat	2%	12%	9%	20%
injury	4%	8%	9%	40%
COVID19	1%	69%	83%	20%

Organising the NDCs by year of submission to the UNFCCC – see Figure 292– demonstrates that later submissions are more likely to refer to a health keyword. Where 2015 and 2016 have only 71% and 78%, respectively, of submissions referring to health, 2020, 2021, and 2022 have between 92% and 93% referring to health. 2023, largely because of the European Union submission with no reference to health, has only 24% of its 34 submissions making such reference.

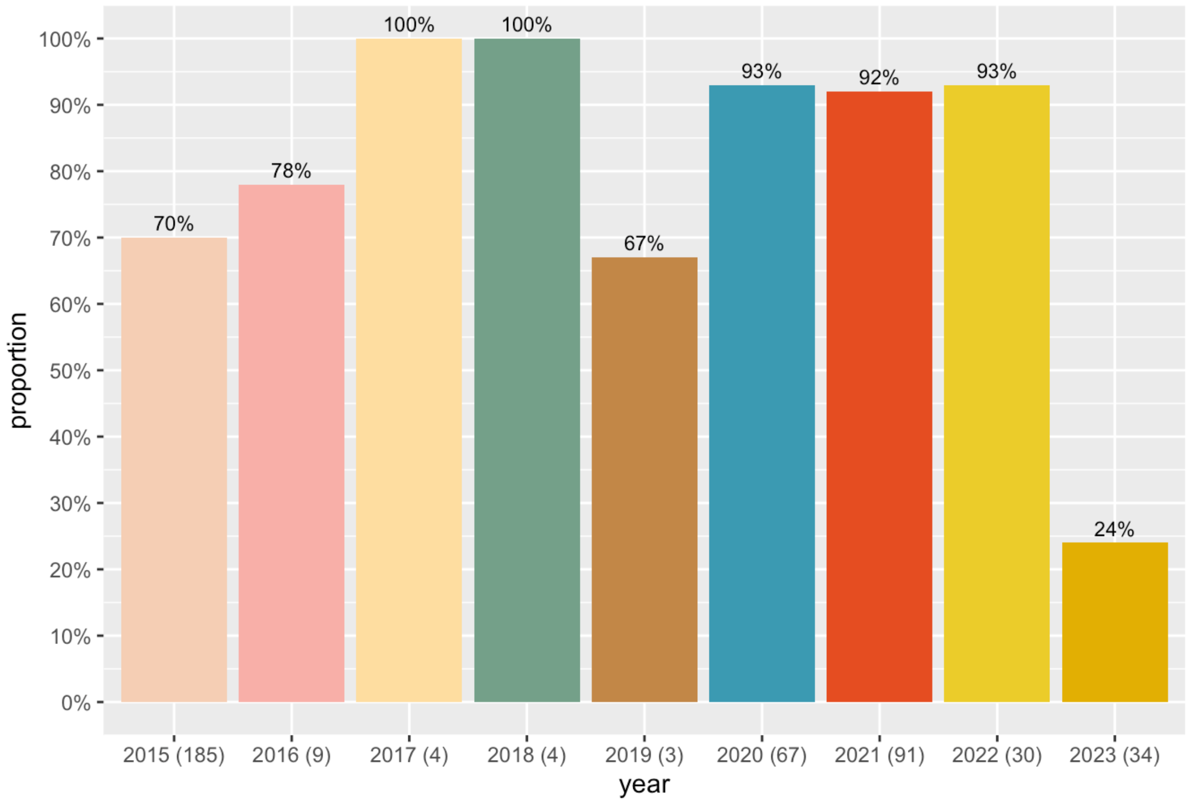

Figure 293: Proportions of NDCs per year mentioning any health keyword.

***Lancet* Countdown groupings**

Figure 294 shows the proportion of NDCs mentioning a health term by *Lancet* Countdown grouping. Member states in the African grouping fairly consistently refer to health across iterations (94% of member states in the first, 98% in the second, and 100% in the third). The South and Central American region is similar, with 88% in the first, 93% in the second, and 100% in the third. The two Northern American member states (USA and Canada) fail to refer to health in the first iteration but in each subsequent iteration they mention a health keyword. Neither of the two included Oceanic member states (New Zealand and Australia) included a health keyword in their first two NDCs, though Australia did in its third iteration. Finally, the European region, largely dominated by the European Union's NDC, made mention of health in only 16% of their first and 10% of their third NDC. With the European Union's second NDC containing reference to a health term far higher at 92%.

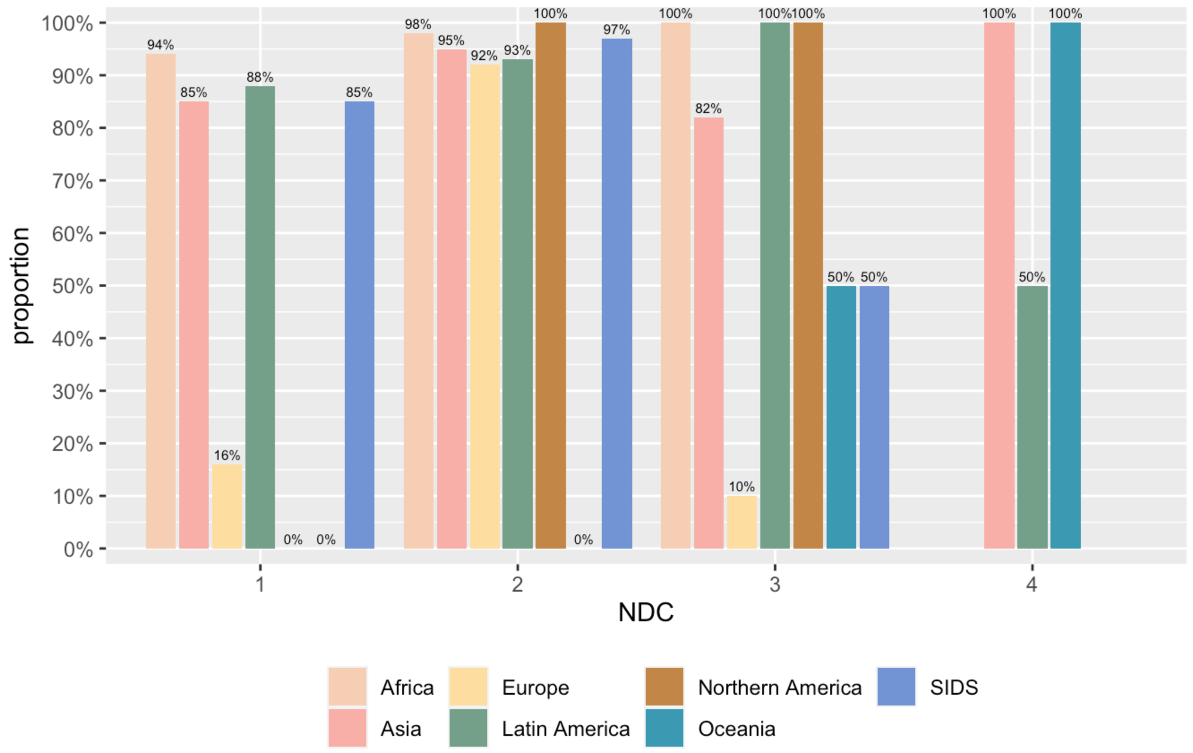

Figure 295: Proportion of NDCs per iteration mentioning any health keyword, by Lancet Countdown grouping

HDI Classification

When organised by HDI classification (see Figure 296), it is member states in the low and medium HDI range that mention health keywords most. Across those in the low HDI classification range, 94% mention a health keyword in the first and 97% in the second NDC, followed by all countries in that range for the third NDC iteration. Across those in the medium HDI range, a similarly high proportion mention health (93% in the first NDC, 97% in the second, falling to 86% in the third). It is those in the Very High HDC classification range that appear to be less likely to mention health.

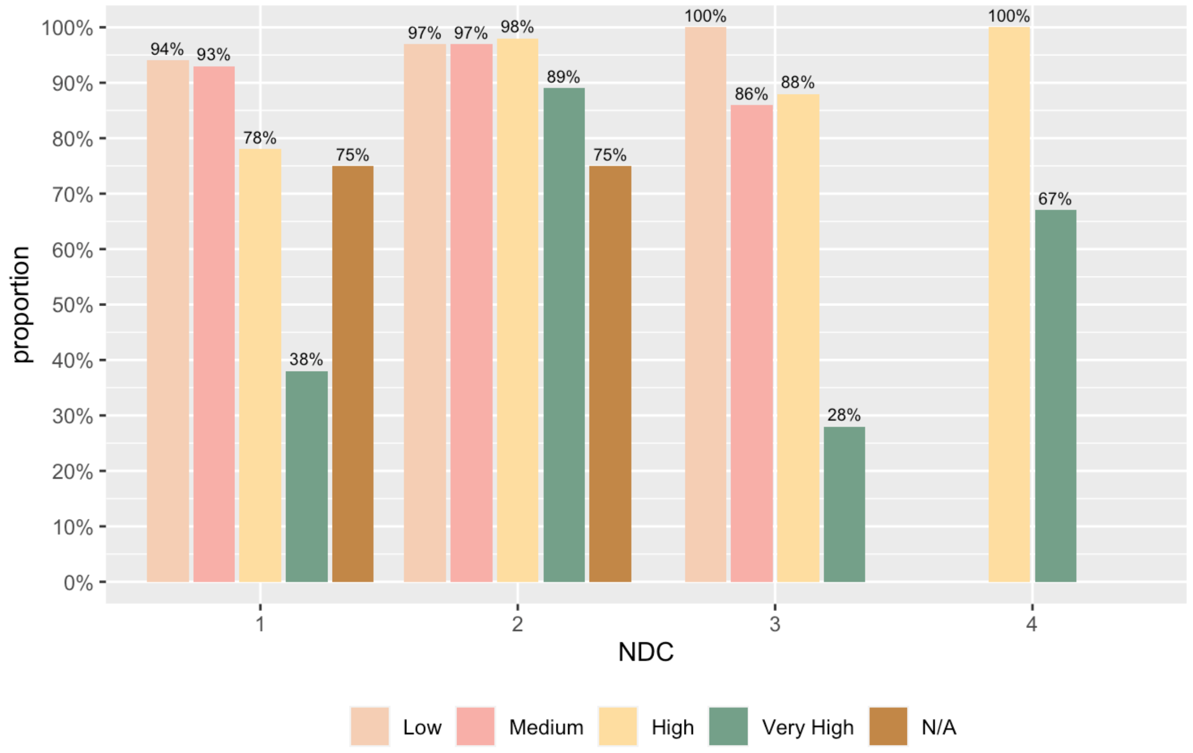

Figure 297: Proportion of NDCs per iteration mentioning any health keyword, by HDI classification

WHO region

Figure 298 shows the proportion of NDCs mentioning a health term by WHO region. This shows similar patterns to those based on *Lancet* Countdown groupings, with the European region showing a much lower proportion of countries mentioning health in the first and third iterations (again, though, this is largely dominated by the European Union NDC), with a higher proportion in the second NDC iteration (94%). Similarly, the Western Pacific region has a lower proportion of member states mentioning health for the first (76%) and third (50%) NDC, while it demonstrates a larger proportion in the second (92%).

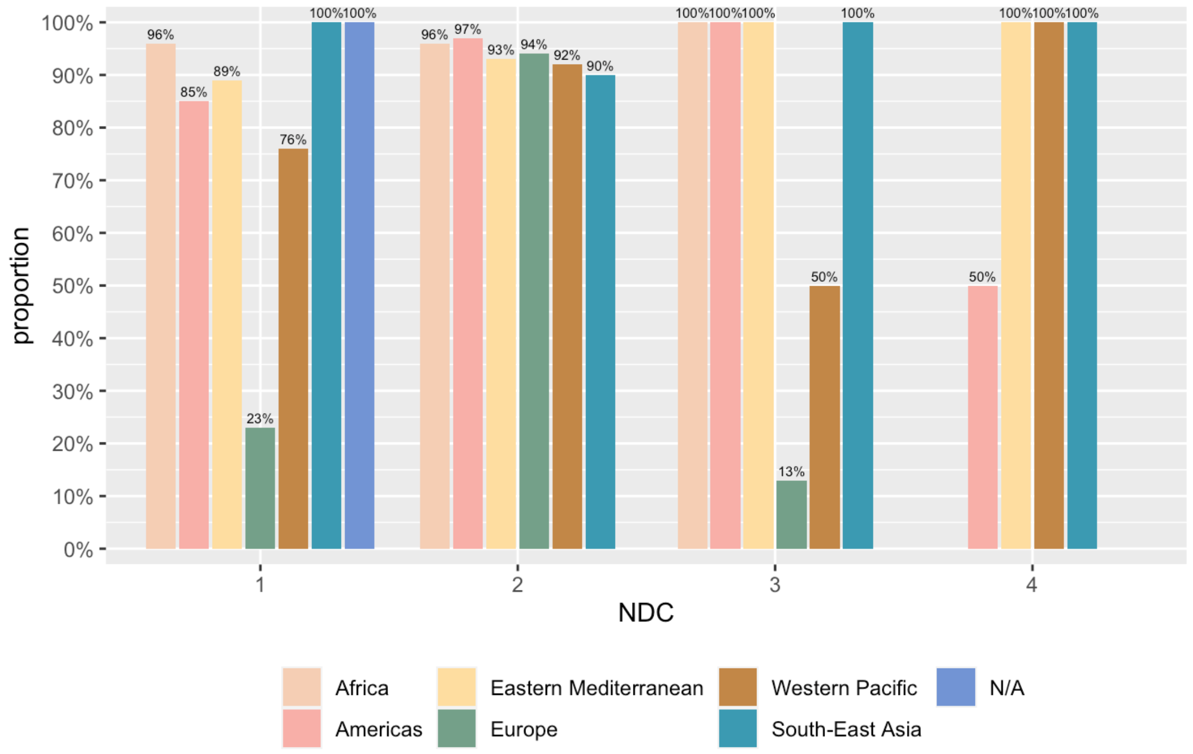

Figure 299: Proportion of NDCs per iteration mentioning any health keyword, by WHO region.

Indicator 5.4.2: engagement by international organisations

Indicator authors

Dr Olga Gasparyan, Prof Slava Jankin, Prof Cathryn Tonne

Methods

While the direct health benefits of climate change mitigation are often longer-term and diffuse, health co-benefits have been shown to provide strong, tangible local impacts, especially for developing countries, in relatively short time frames. They therefore can provide compelling arguments for increasing mitigation ambition. In many instances, the health co-benefits have been shown to outweigh the mitigation costs. Nonetheless, a gap remains between the potential and the actual role of health co-benefits in the development of national climate change mitigation policy and engagement with the concept by major international organisations. Possible explanations include the lack of political traction of health co-benefits, a dominant focus on cost minimisation (as opposed to cost-benefit analysis)[468] and barriers in research translation.[469] Natural language processing (a subfield of machine learning) is used here to track the uptake and engagement of health co-benefits in policy discourse on social media of major international organisations (IOs) involved in climate change adaptation and mitigation work.

With the dataset of IO's tweets a search through the text of each tweet was performed to identify if they discuss co-benefits. First, this included the standard list of keywords used in WG5 analysis. This produced limited results as the terms are both not specific enough to co-benefits and also do not match well to the mode of communication on social media platforms. Next, a list of keywords was developed corresponding to seven exposure pathways linking mitigation action and health (e.g., air pollution, plant-based diets)[470], and terms directly relating to the concept of health co-benefits (e.g., ancillary benefits), and specific mitigation interventions expected to have health co-benefits (e.g., transition to

renewable electricity generation). This approach was selected to encompass the overall concept of health co-benefits of mitigation as well as the specific ways in which they might be achieved. A full list of search terms is provided below.

An indicator of engagement intensity was developed as a monthly proportion of tweets containing at least one term from the search term list in relation to the total number of tweets by that IO. An originally written computational algorithm was developed to identify the lists of tweets that contain each of the keywords from a given list of keywords for each international organisation in each month of the 2010–2023 period. All lists were combined and a list of unique tweets was identified by excluding any duplicate tweets that were the results of several keywords being mentioned in the same tweet. The number of tweets in these unique tweets list was the total number of keyword-mentioned tweets, used as the numerator. The total number of tweets written by each IO in a given month was calculated and the total sum as a denominator. The resulting indicator is simply a proportion calculated by dividing keywords mentions by the total sum.

To identify the intensity for individual pathways the exact same calculations were performed but only using the keywords form a given pathway. Due to the presence of multiple keywords in the same tweet the sum of the number of tweets with each of the pathway keywords is not equal to the total number of tweets with all the keywords.

Keywords:

Direct co-benefits terms: health benefit, win-win, double dividend, benefit, cobenefit, co-benefit, secondary benefit, ancillary benefit, side benefit, collateral benefit, associated benefit, ancillary effect, knock on effect, ancillary impact, side effect, co-control, carbon benefit, reduction benefit, synergy, side effect, spillover, trade-off, distributional aspect, distributional effect, mortality impact.
Policy related terms: Paris Agreement, 2 degrees, 2C, 2°C, 1.5°C, 1.5C, Climate pledge, Climate goals, Energy pledge, Net-zero, NetZero, Zero emission, Decarbonisation, Decarbonization, Mitigate, Mitigation, Carbon neutral, Carbon neutrality, Low carbon.
Intervention, Energy: Renewables, Solar, Photovoltaics, PV, Batteries, Wind, Coal, Clean energy, Energy demand, Energy use, Energy efficiency, Heat pumps, Building retrofit, Smart thermostat, Insulation, Net-zero buildings, Green roof, Cool roof, Electric vehicles, Clean cooking, LED lighting, Geothermal power, Fuel poverty, Energy poverty, Nuclear, Electricity, Hydrogen, Fossil fuel, Energy crisis, Energy investment, Affordable energy, Natural gas
Intervention, Land use: Forest restoration, Tree plantation
Pathway, Air Pollution: Air quality, Air pollutants, Air toxin, PM2.5 , PM25, particles, particulates, ozone, Smog, Soot, black carbon , short-lived climate pollutants, SLCP , SLCPs
Pathway, Road transport noise: Traffic noise, Aircraft noise
Pathway, Temperature: Urban heat island, Heat, Overheat, Carbon sequestration, Cooling, Humidity, Mold
Pathway: Diet: Dietary, Nutrition, Meat, Dairy, Vegetarian, Vegan , Plant-based, Plant-rich,
Pathway, Physical activity: Exercise, Active travel, Walking, Walkable, Cycling , Bicycle,
Pathway, Sustainable mobility: Public transport, Rail, Trains
Pathway, Nature exposure: Green, greenspace, green space, Cooling, Trees, Forest , Nature based solution, Nature

Data

Forty-one international organisations were selected based on IOs representing various sectors: economic, financial, environment, regional development, etc. The sample of IOs is based on a recent study on policy discourse in climate change adaptation IOs[471] and extended to cover mitigation IOs. Twitter is used as the social media platform to collect official communication by IOs. All the tweets written by official Twitter accounts of the selected IOs were extracted for the period of 2010–2022. Since social media platforms have become one of the most common sources of information for journalists and the general public, policy actors utilise the platforms as the key mode of media and public engagement. Additionally, using the same platform and data structure allows a comparison of engagement across international organisations controlling for institutional variability in formats and modes.

The final dataset contains 1,392,892 tweets written by 41 international organizations through the period of 2010-2022, from which 1,354,924 are English language tweets. These are all the tweets published by official twitter accounts of these organizations, including retweets and quotations. Since the majority of tweets are written in English and all keywords are in English, the further analysis below only works with the English language tweets and excludes all the tweets that do not have "lang=en" in the tweets language meta-data identifier.

Table 146: List of International Organisations with Sectors and Field Classification

Organization	Acronym	Twitter handle	Field	Sector Classification
African Union	AU	_AfricanUnion	Regional Cooperation	adaptation
Asian Development Bank	ADB	ADB_HQ	Global Development Banking	both
African Development Bank	AFDB	AfDB_Group	Global Development Banking	adaptation
The Africa Rice Center, formerly known as the West Africa Rice Development Association	WARDA	AfricaRice	Food and Agriculture	adaptation
Asia-Pacific Economic Cooperation	APEC	APEC	Regional Cooperation	both
Association of Southeast Asian Nations	ASEAN	ASEAN	Regional Cooperation	both
European Bank for Reconstruction and Development	EBRD	EBRD	Global Development Banking	adaptation
Economic Community of West African States	ECOWAS	ecowas_cedeao	Trade and Economy	adaptation
European Investment Bank	EIB	EIB	Global Development Banking	both

European Union	EU	EU_Commission	Regional Cooperation	both
UN Food and Agriculture Organisation	FAO	FAO	Food and Agriculture	adaptation
Pacific Islands Forum	PIF	ForumSEC	Regional Cooperation	adaptation
International Energy Agency	IEA	IEA	Energy Policy	mitigation
International Fund for Agricultural Development	IFAD	IFAD	Food and Agriculture	adaptation
International Finance Corporation	IFC	IFC_org	Global Development Banking	both
International Monetary Fund	IMF	IMFNews	Global Development Banking	both
International Renewable Energy Agency	IRENA	IRENA	Energy Policy	mitigation
North Atlantic Treaty Organization	NATO	NATO	Peace and Security	adaptation
Organization of American States	OAS	OAS_official	Regional Cooperation	both
Organization for Economic Co-operation and Development	OECD	OECD	Development	both
Organization for Security and Co-operation in Europe	OSCE	OSCE	Peace and Security	adaptation
United Nations High Commissioner for Refugees	UNHCR	Refugees	Migration	adaptation
South Asian Association for Regional Cooperation	SAARC	SaarcSec	Regional Cooperation	adaptation
Southern African Development Community	SADC	SADC_News	Development	adaptation
Inter-American Development Bank	IADB	the_IDB	Global Development Banking	adaptation
UN Security Council	UNSC	UN	Peace and Security	adaptation
UN Development Programme	UNDP	UNDP	Development	adaptation
United Nations Office	UNDRR	UNDRR	Disaster Risk	adaptation

for Disaster Risk Reduction			Management	
United Nations Economic Commission for Europe	UNECE	UNECE	Development	both
United Nations Environment Programme	UNEP	UNEP	Environment Policy	mitigation
United Nations Framework Convention on Climate Change	UNFCC	UNFCCC	Environment Policy	mitigation
United Nations Population Fund	UNFPA	UNFPA	Health	adaptation
United Nations Children's Fund	UNICEF	UNICEF	Development	adaptation
International Organisation for Migration	IOM	UNmigration	Migration	adaptation
United Nations Office for the Coordination of Humanitarian Affairs	OCHA	UNOCHA	Disaster Risk Management	adaptation
World Economic Forum	WEF	wef	Trade and Economy	both
World Food Programme	WFP	WFP	Food and Agriculture	adaptation
World Health Organization	WHO	WHO	Health	both
World Bank	WB	WorldBank	Global Development Banking	both
World Trade Organisation	WTO	wto	Trade and Economy	both
East African Community	EAC	jumuiya	Regional Cooperation	adaptation

Caveats

There are several limitations of the current analysis to be improved in the next iterations of this study. First, working with a limited predefined set of international organisations: the plan is to expand the set for the near universal set of international organisations with the active Twitter accounts. Second, the set of search terms will be further fine-tuned to provide a richer picture of co-benefits discussions.

Additional Analysis

IOs intensify their engagement with health co-benefits in their public discourse over time. In comparison with the data from last year’s report where it was noted that by November 2022, 22% of

English language tweets mention co-benefits, in the new 2023 data it can be seen that this value increases even more, approximating 25% (1244 tweets with key words/4889 total number of tweets).

Figure **300** shows the dynamic of the intensity of engagement, which is the proportion of an IO's Twitter posts mentioning co-benefits with respect to the total number of tweets by that IO. The structure of the measure suggests that the observed trend is not simply reflecting IOs' increasing use of this specific social media platform, but rather a genuine increase in co-benefit topic engagement.

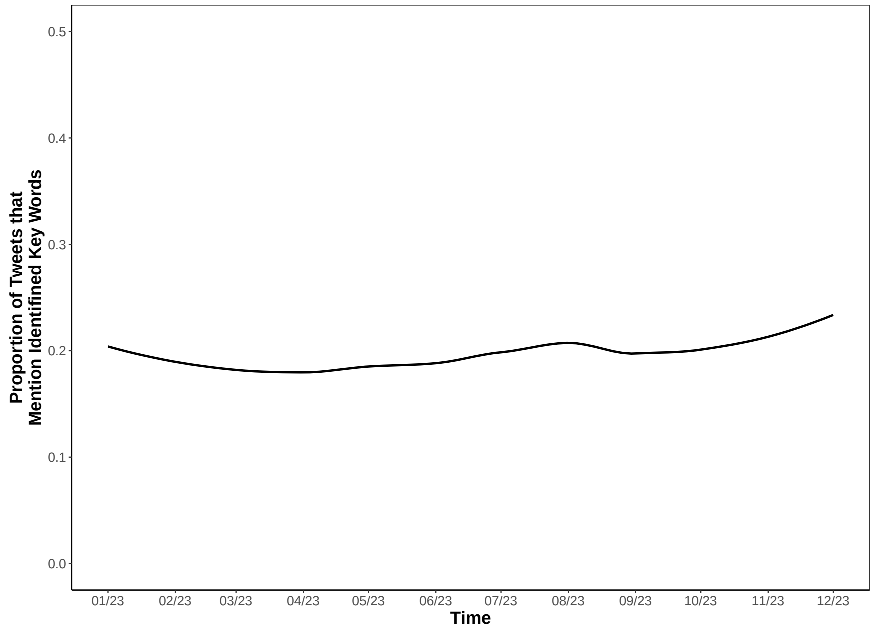

Figure 301: Engagement intensity of IOs with co-benefits on Twitter (Note: Trend line is a fitted loess line with the shaded area representing a 95% confidence interval)

IOs in the sample are diverse both in terms of their sectoral focus and in terms of key operational focus on mitigation, adaptation or a mix of adaptation and mitigation work. Figure **302** shows the results of the analysis across the mitigation/adaptation/both categorisations. Mitigation IOs have a higher co-benefits engagement intensity compared to adaptation IOs (or IOs working both on mitigation and adaptation). Increasing time trends for all three groups of IOs can be observed, with the most evident growth after early 2016 likely linked to the Paris Agreement. It is observed that mitigation IOs have a higher co-benefits engagement intensity compared to adaptation IOs (or IOs working both on mitigation and adaptation). Also observed are increasing time trends for all three groups of IOs starting from the middle of the year and picking up in July and November–December of 2023.

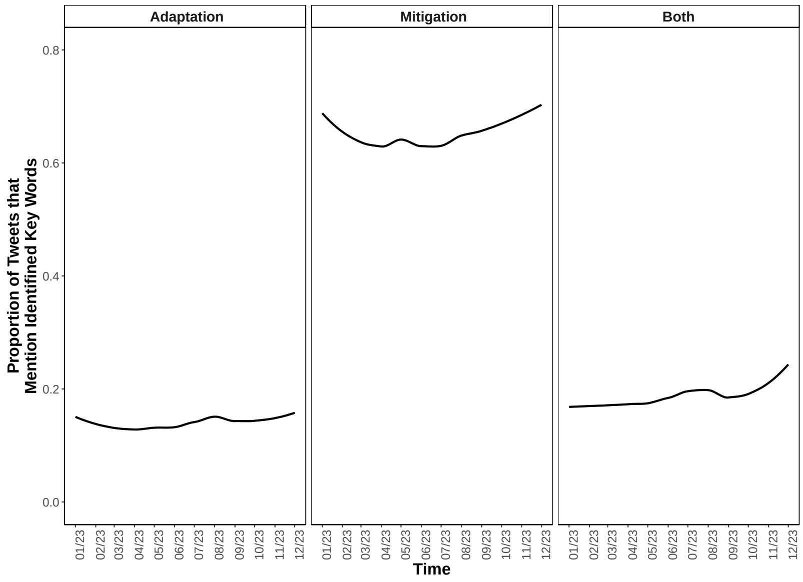

Figure 303: Engagement intensity of IOs with co-benefits on Twitter across adaptation, mitigation or both categorisation.

Sectoral analysis results are provided in Figure **304** showing clear sectoral differences in engagement intensity with co-benefits. The sectoral classification is adopted from Kural et al.[471] The most intensive co-benefits engagement is from IOs in the Energy, Environment, Food and Agriculture, and Global Development Banking sectors.

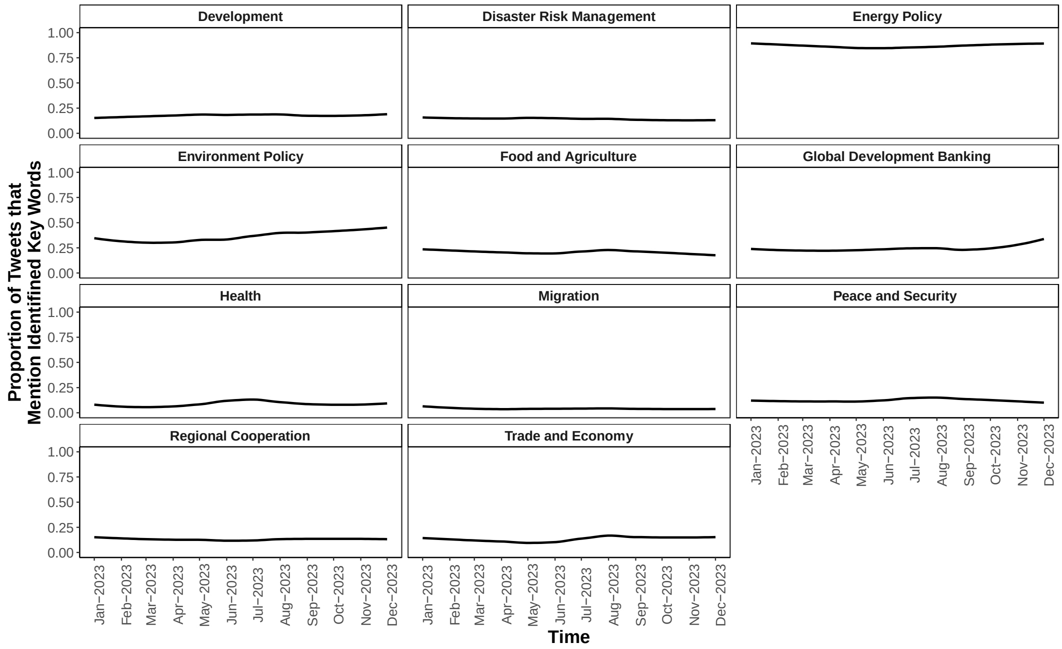

Figure 305: Sectoral analysis of engagement intensity of IOs.

The results for individual IOs are presented in X. IRENA and IEA (both from the energy sector) are the most prominent in their engagement.

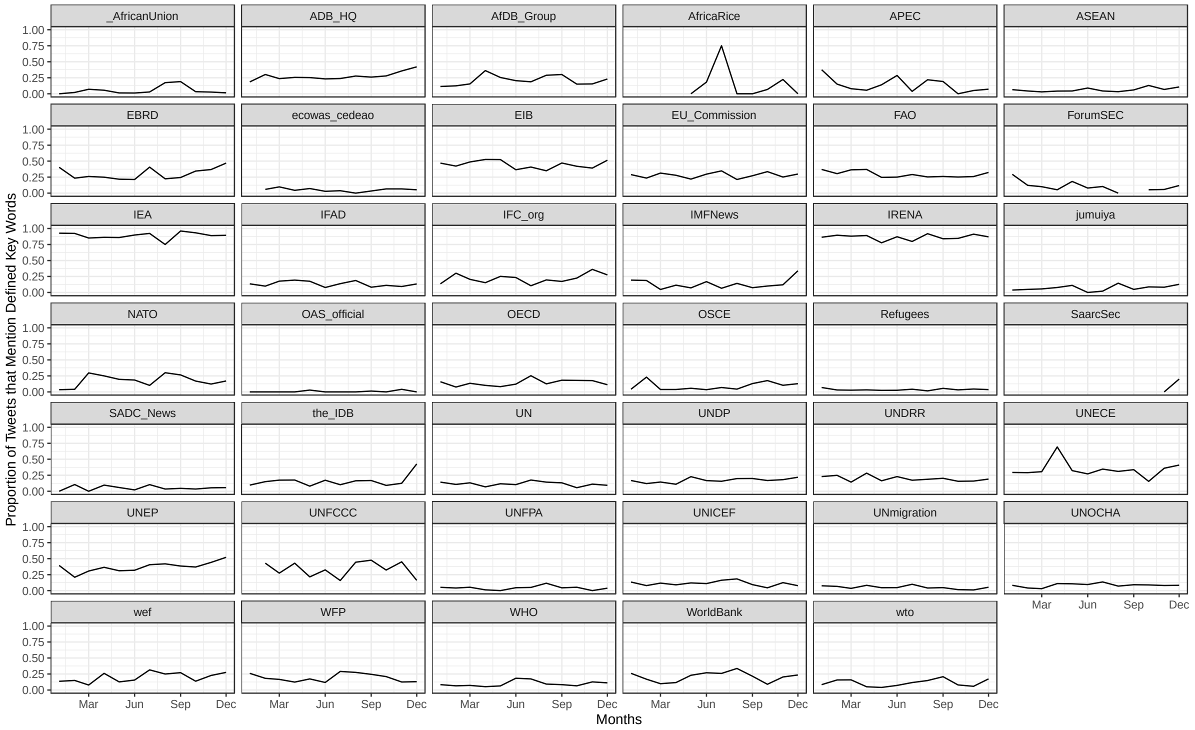

Figure 306: Engagement intensity for individual IOs (Note: Names of the IOs listed according to the official acronyms, which are listed in Table 146).

Topic modelling

Figure 308307 shows that the highest engagement intensity is around energy, nature exposure, temperature, policy specific, and co-benefits specific terms. As a validation exercise, the indicator also looked at the content of the tweets that contain the search terms. To do this at scale, probabilistic topic models are used[472] — a group of unsupervised machine learning techniques applied to the collected text corpus (tweets containing the keywords). However, for validation, in addition to identifying topic clusters in tweets, the indicator looked to understand if the topics identified are related to a set of covariates that capture specific characteristics of IOs and time-series dynamics. Structural topic models (STM)[473,474] are utilised for this task. Overall, there is evidence that, indeed, the content of the tweets picked up through the search terms is related structural IO and pathway characteristics.

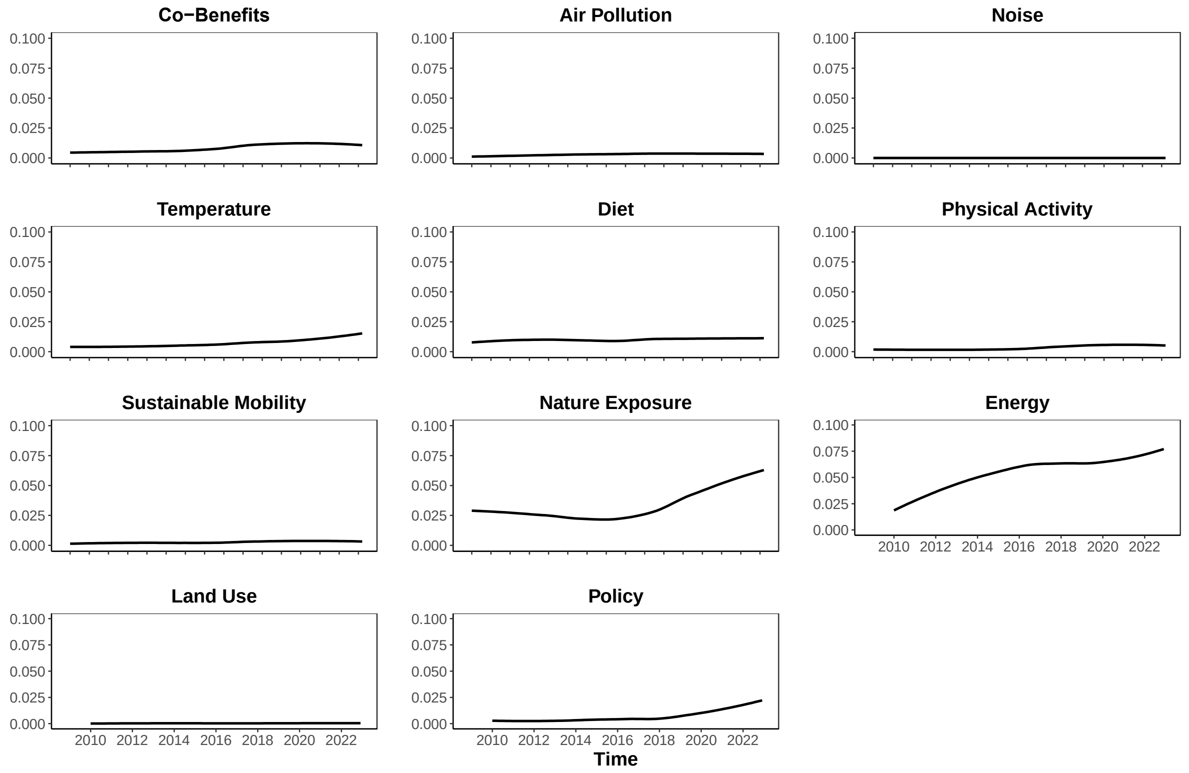

Figure 308: Exposure intensity by search term category.

Indicator 5.5: corporate sector engagement with health and climate change

Indicator authors

Prof Slava Jankin and Dr Ran Zhang

Methods

To produce the measure of engagement with climate change and health in companies' UN Global Compact Communication of Progress (GCCOP) reports, the publicly available GCCOP reports are used. Our approach to using the GCCOP reports to produce the indicators is based on identifying references to key search terms linked to (a) health, (b) climate change, (c) gendered impacts, (d) covid impacts and (d) inequality impacts:

Table 147: Key search terms

Health terms	Climate change terms	Gender terms	Covid terms	Inequality terms
malaria<br>diarrhoea<br>infection<br>disease<br>diseases<br>sars<br>measles<br>pneumonia	climate<br>change<br>changing<br>climate<br>climate<br>emergency<br>climate action<br>climate crisis	gender<br>male<br>female<br>man<br>men<br>woman<br>women<br>sex	covid-19<br>covid19<br>covid 19<br>corona sars-<br>cov-2<br>covid<br>corona virus	inequality<br>inequity<br>injustice<br>justice<br>equity<br>equality

epidemic<br>epidemics<br>pandemic<br>pandemics<br>epidemiology<br>healthcare<br>health<br>mortality<br>morbidity<br>nutrition<br>illness<br>illnesses<br>ncd<br>ncds<br>air pollution<br>nutrition<br>malnutrition<br>malnourishme<br>nt<br>mental<br>disorder<br>mental<br>disorders<br>stunting	climate decay<br>global<br>warming<br>green house<br>temperature<br>extreme<br>weather<br>global<br>environmenta<br>l change<br>climate<br>variability<br>greenhouse<br>greenhouse-<br>gas<br>low carbon<br>ghge<br>ghges<br>renewable<br>energy<br>carbon<br>emission<br>carbon<br>emissions<br>carbon<br>dioxide<br>carbon-<br>dioxide<br>co2 emission<br>co2 emissions<br>climate<br>pollutant<br>climate<br>pollutants<br>decarbonizati<br>on<br>decarbonisati<br>on<br>carbon neutral<br>carbon-<br>neutral<br>carbon<br>neutrality<br>climate<br>neutrality<br>net-zero<br>net zero			

In order to produce an indicator of engagement with the intersection of climate change and health, a focus was made on whether any of the climate change related terms appeared immediately before or after any public health terms in the GCCOP reports. This was based on a search of the 25 words before and after a reference to a public health related term. To further produce indicators involving the COVID-19 pandemic, gender and inequality, additional search terms related to gender, COVID-19 and inequality to identify which of the intersection references also engaged with these issues were used.

Data

To produce this indicator, data is drawn from the publicly available UN Global Compact COP reports. A total of 50,016 reports were downloaded from GCCOP and converted into plain text format for analysis. The reports are available for companies based in 145 countries. Reports were submitted in 40 languages with the majority reports of English language (50%). All reports of other languages were translated into English using the open-source pretrained neural machine translation model Opus-MT[475] and m2m100_418M[476] under the Huggingface[477] pipeline to implement the translation task. The distribution of available GCCOP reports over time is presented in Table 148:

Table 149: Number of SCCOP reports by year

Year	Number of reports
2011	2057
2012	3015
2013	3231
2014	3186
2015	3472
2016	3573
2017	3724
2018	3751
2019	4060
2020	3554
2021	5737
2022	6089
2023	4567

There are only single GCCOP report submissions before 2011, thus the sample of GCCOP reports is limited to the period 2011–2023. The documents were translated, pre-processed and prepared for the application of natural language processing by converting the reports to plain text format; removing stopwords and regularising (lowercasing). Pre-processing and analysis was primarily carried out in Python using NLTK package.[478]

Caveats

This analysis here is based on a narrow range of search terms based on keywords, which excludes reference to many of indirect links between climate change and health, and between the intersection and other terms such as gender, covid, and inequality. Reports may also discuss indirect connections, such as the effect of climate change on agriculture, however, these are not included here. Therefore, the

results present a somewhat conservative estimate of high corporate engagement with the intersection of climate change and health. Future work in this area will consider engagement with these indirect links, as well as providing additional forms of analysis.

Future Form of Indicator

In the future, the aim is to include search terms based on indirect links between climate change and health (e.g., agriculture) to capture references to indirect links.

Additional Information

Figure 309 presents the total number of references to climate change, health, and the intersection of climate change and health across for the GCCOP reports. Despite the increase in the proportion of companies engaging with the intersection of climate change and health, the overall number of references remains fairly low and consistent, relative to the individual references to health and climate change – though, there has been an increase in references to the intersection of climate change and health since 2020.

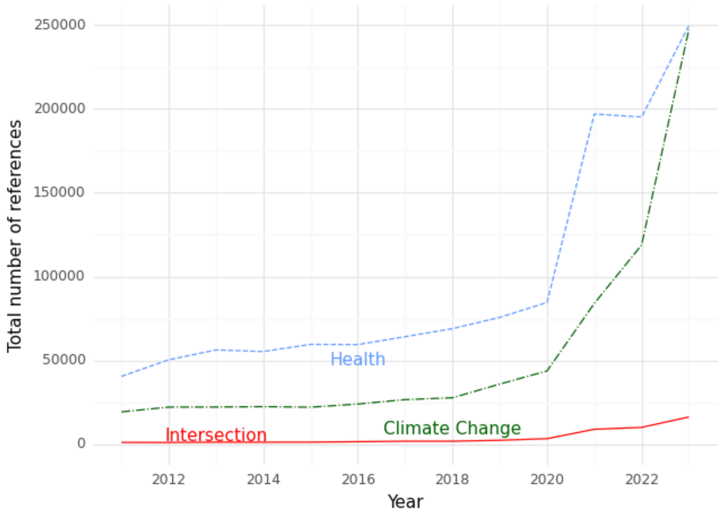

Figure 310: Total references to climate change, health, and the intersection of climate and health, 2011–2023.

In Figure 311, below, the total references with the intersection of climate change and health to better show trends occurring in the engagement are presented. The figure shows that since 2018 – and particularly since 2020 – there has been a sharp rise in the number of references, especially in 2021 and 2023.

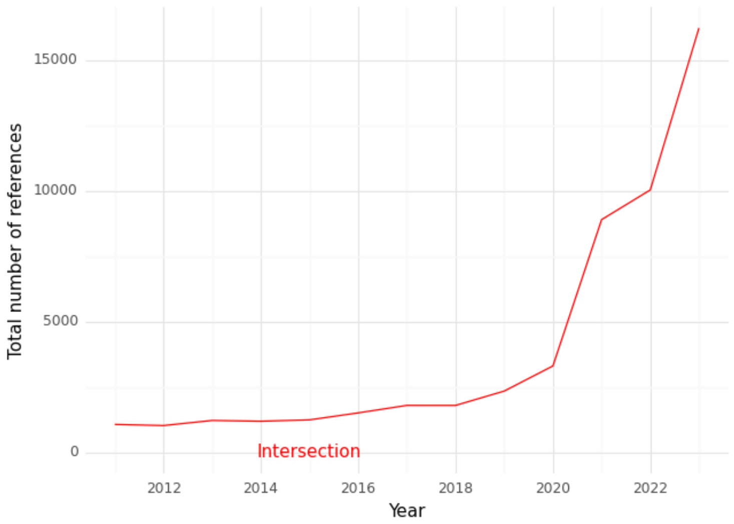

Figure 312: Total references to the intersection of climate change and health, 2011–2023.

Figure 313 shows the average number of references to climate change, health, and the intersection in GCCOP reports. The figure again demonstrates the relatively low level of intersectional engagement in GCCOP reports, compared to the separate references to health and climate change. Worth noting is the observed sharp increase in separate references to health and climate change in 2023, especially the references to climate change which achieves a similar level to that of health.

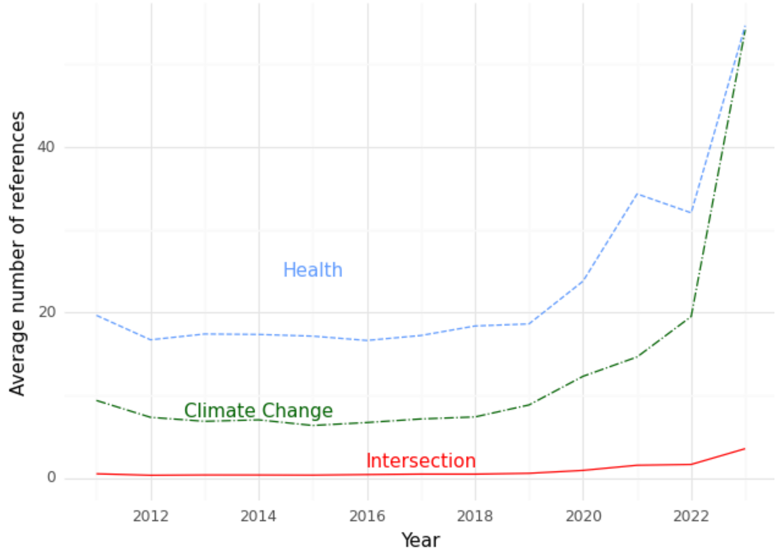

Figure 314: Average references to climate change, health, and the intersection of climate change and health in GCCOP reports, 2011–2023.

There is growing awareness of the gendered impacts of climate change and health. The extent to which references to the intersection of health and climate change in companies' GCCOP reports engage with gender issues was considered. Following identification of all the references to the intersection in GCCOP reports for 2011–2023, additional search terms related to gender were used to identify which of the intersection references also engaged with gender issues. The gender-related search terms used were as follows: *gender, male, female, man, men, woman, women, sex*. Hence, the analysis considers whether

the 25 words of text identified in the primary search (for climate change and health terms) includes a reference to at least one of these gender-related keywords.

Figure 315 presents annual references to the intersection of health and climate change with a gender focus in companies' annual GCCOP reports between 2011 and 2023. The figure shows a steady increase in engagement between 2015 and 2018. In 2019, there was a sharp rise, with 11% of documents (i.e., companies) referring to the intersection of climate change and health including a mention of one of the gender keywords, followed by another sharp rise with 16% of all companies in 2023.

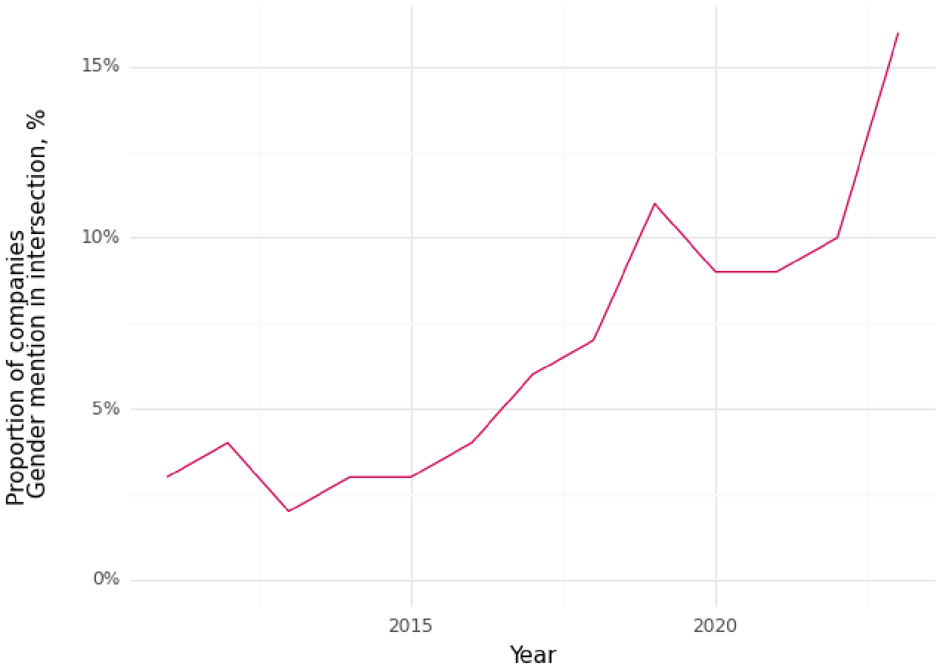

Figure 316: Proportion of documents to the intersection of health and climate change in GCCOP reports that include a reference to gender, 2011–2023.

Additionally, an examination of the impacts of inequality to the climate change-health intersection using these inequality-related keywords has been carried out. Figure 317 presents annual references to the inequality dimensions of climate change and health intersection between 2011 and 2023. The figure shows a steady increase in engagement between 2016 and 2023 with a decrease between 2014 and 2015. Overall, a notable increase in inequality-related impacts from below 6% of documents (i.e., companies) in 2011 to 25% of companies in 2023 was observed.

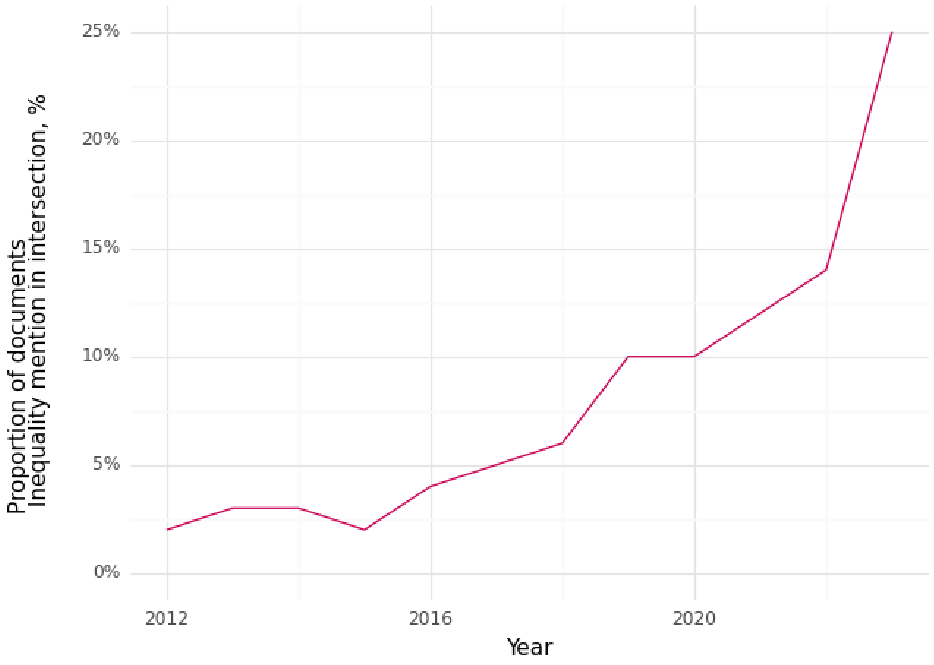

Figure 318: Proportion of documents to the intersection of health and climate-change that include a reference to inequality in GCCOP reports 2011–2023.

Also considered was engagement with climate change and health in the UN Global Compact COP reports by WHO region. Figure 319 shows the total number of references to the climate change-health intersection based on which of the WHO regions a company is based on, and Figure 320 shows the proportion of companies based in the different WHO regions that refer to the health impacts of climate change in their annual GCCOP report.

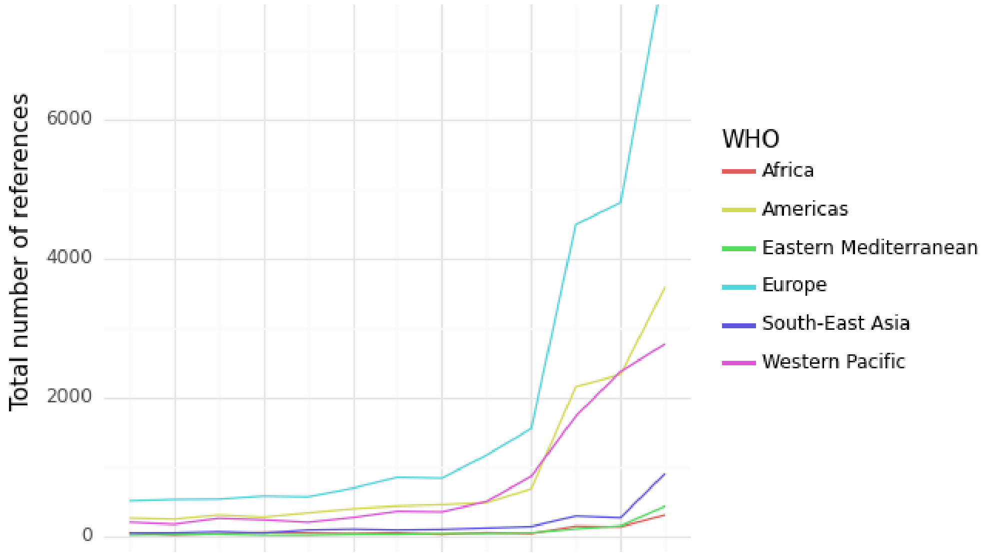

Figure 321: Total references with the intersection of climate change and health by WHO region, 2011–2023.

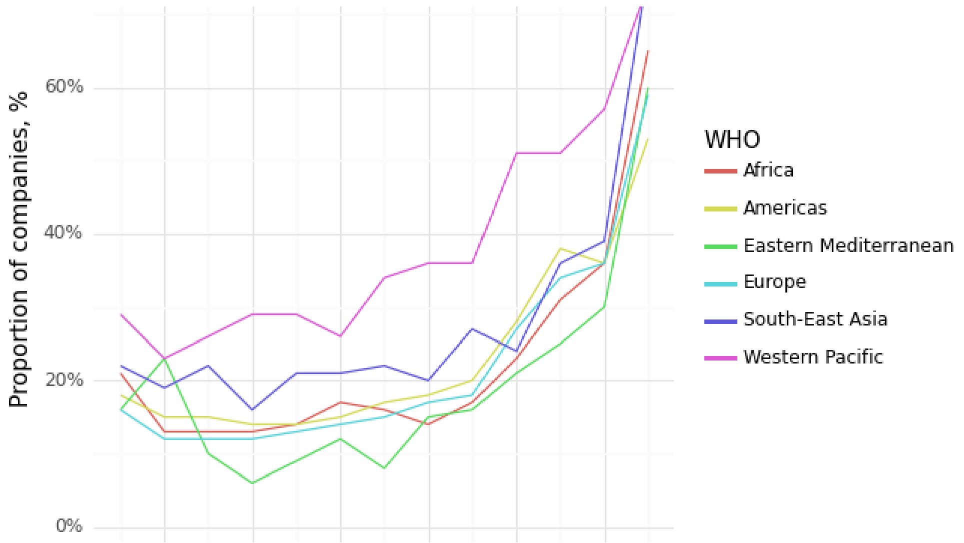

Figure 322: Proportion of companies referring to the intersection of health and climate change by WHO region, 2011–2023.

Figure 323 and Figure 324**Error! Reference source not found.** show that the highest proportion of GCCOP reports engaging with the climate change-health intersection in recent years came from corporations based in the Western Pacific and the second highest in the South-East Asia. The lowest engagement comes from corporations based in the Eastern Mediterranean region. There has been rising engagement with health and climate change across all the regions.

Also considered is engagement across different sectors. Table 150 shows the total number of references to climate change, health, and the intersection across the different sectors in 2023. Figure 202 presents the proportion of corporations engaging with the climate change-health relations in each sector in 2023.

Table 151: Total number of references to health, climate change, and to the intersection of climate change and health by sector in 2023.

Sector	intersection	climate	health
aerospace & defense	414	4886	9036
alternative energy	708	10716	9980
automobiles & parts	1056	19447	25377
banks	1358	29106	31890
basic resources	0	5	17
beverages	938	12150	22223
chemicals	2626	26351	48356
construction & materials	2305	31938	63896
diversified	3837	55300	79537
electricity	1833	38074	41602
electronic & electrical equ...	1115	20273	28125
finance and credit services	901	17248	12266
financial services	1909	33976	41000
food producers	2831	24660	71921

gas, water & multiutilities	1132	19179	26079
general industrials	2850	38698	70806
health care	0	7	50
household goods & home cons...	626	10643	17688
industrial engineering	949	12745	21448
industrial goods & services	2	50	644
industrial materials	586	10344	12508
industrial metals & mining	895	12060	28383
industrial metals and mining	1121	13744	24000
industrial support services	2501	37217	74915
industrial transportation	1178	17808	27385
investment banking and brokerage services	76	1579	1101
leisure goods	114	2824	4548
life insurance	791	6677	15503
media	307	6354	12402
medical equipment and services; health care providers	1609	6969	57517
nonlife insurance	586	7178	14929
not applicable	234	4136	16615
oil, gas, & coal	2729	38127	55399
open end and miscellaneous investment vehicles	3	56	29
other	0	26	146
personal goods	950	10758	20303
pharmaceuticals & biotechno...	3200	13638	88040
real estate investment & se...	1059	17461	22881
real estate investment trusts	438	4892	4729
retailers	1435	24138	41984
software & computer services	1632	26874	35057
technology	0	8	51
technology hardware & equip...	1072	18520	26670
telecommunications equipment; telecommunications service providers	1033	16258	27427
travel & leisure	759	13127	22100

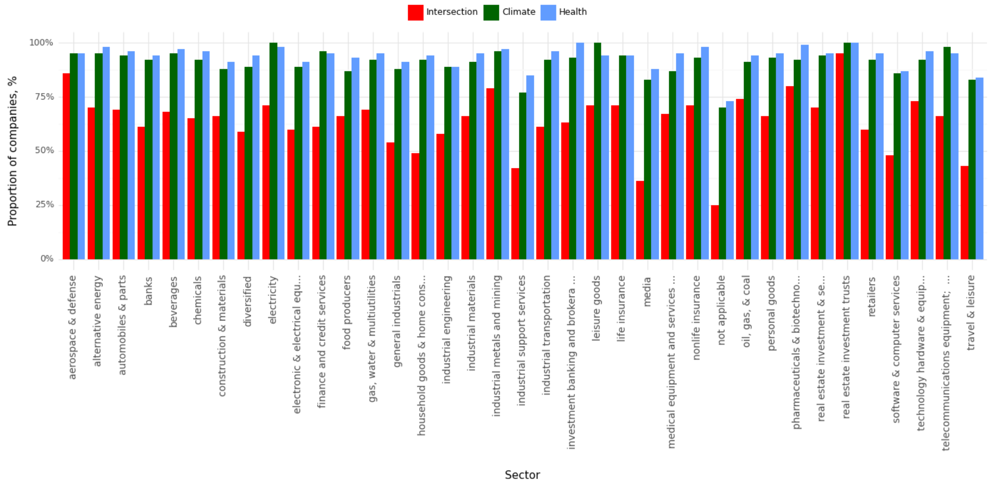

Figure 325: Proportion of corporations referring to climate change, health, and the intersection of climate change and health by sector in 2023.

As shown in Figure 325, the highest level of engagement with the intersection of climate change and health in 2023 can be seen in Pharmaceuticals & Biotechnology (81%, 83 of 103 companies), Industrial metals and mining (78%, 83 of 106 companies), and Oil, gas, & coal (74%, 78 of 105 companies). Health-related sector medical equipment and services/health care providers see lower levels of engagement, where 67% of 86 companies refer to the intersection of health and climate change in their 2023 GCCOP reports. This does, however, represent a significant increase in companies in the health care equipment and services sector engaging with health and climate change in the GCCOP reports compared to 2022 (22% in 2022).

In addition to looking at companies by WHO region, companies from different types of countries in terms of their IPCC groupings were also considered. As noted in previous years' reports, the SIDS have driven much of the engagement with the health impacts of climate change, as well as climate change more generally, in the international fora. As such, included is a SIDS grouping (Figure 326 and 327). The figures show the highest proportion of engagement from companies in North America, the SIDS, Oceania, and Asia.

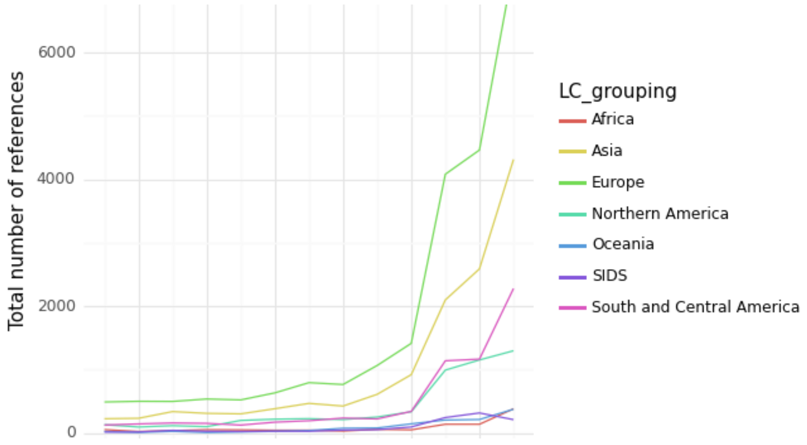

Figure 328: Total references to the climate change-health intersection by country groupings, 2011–2023.

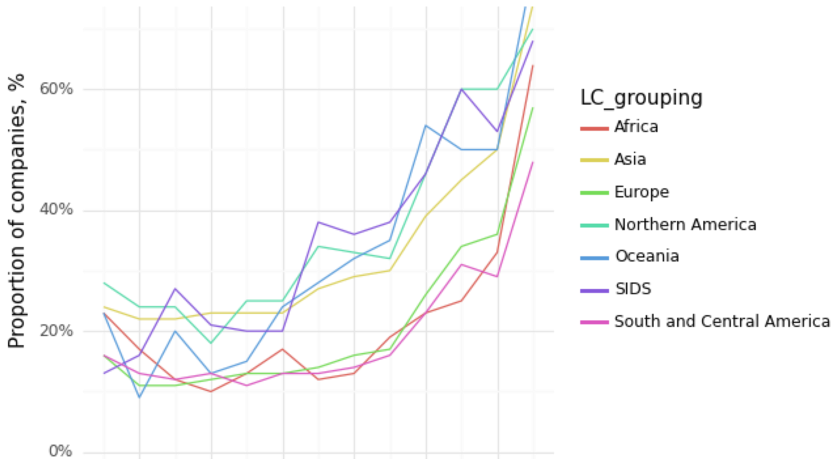

Figure 329: Proportion of corporations referring to the climate change-health intersection by country groupings, 2011–2023.

Corporate engagement with the health dimensions of climate according to the Human Development Index (HDI) categories of the countries in which companies are based is also considered. Figure 330 shows the total references to the intersection of climate change and health in companies' GCCOP reports based on the country HDI category (in 2021) and Figure 331 shows the proportion of companies engaging with climate change and health in their GCCOP report by HDI category. Figure 332 shows significantly higher references to climate change and health made by countries based in countries that have very high human development compared to companies based in countries with other levels of human development. However, this reflects the fact that the majority of companies included in the analysis are based in countries with very high human development levels. It is worth noting that when the proportion of companies that engage with climate change and health is considered (Figure 333), companies based in countries with very high human development have the highest engagement, followed by a close match with medium and high HDI. Overall, companies based in countries with low human development levels have lower engagement with climate change and health.

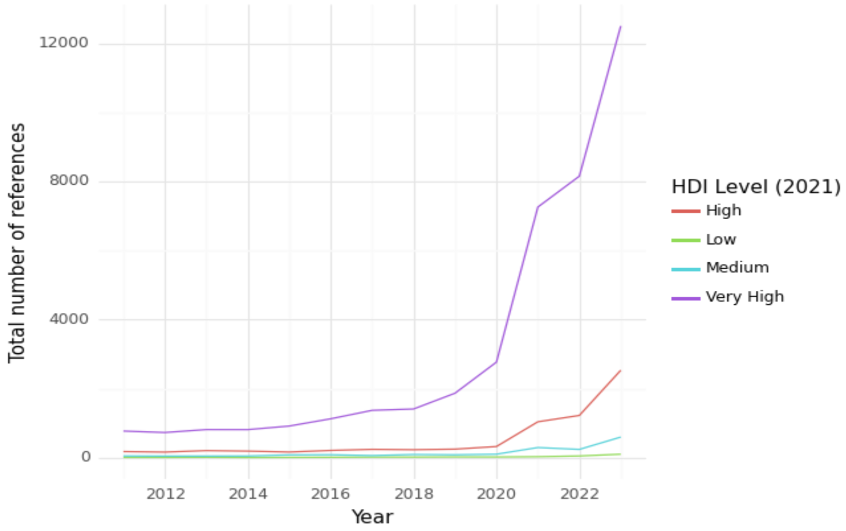

Figure 334: Total references to the climate change-health intersection by country HDI groupings, 2011–2023.

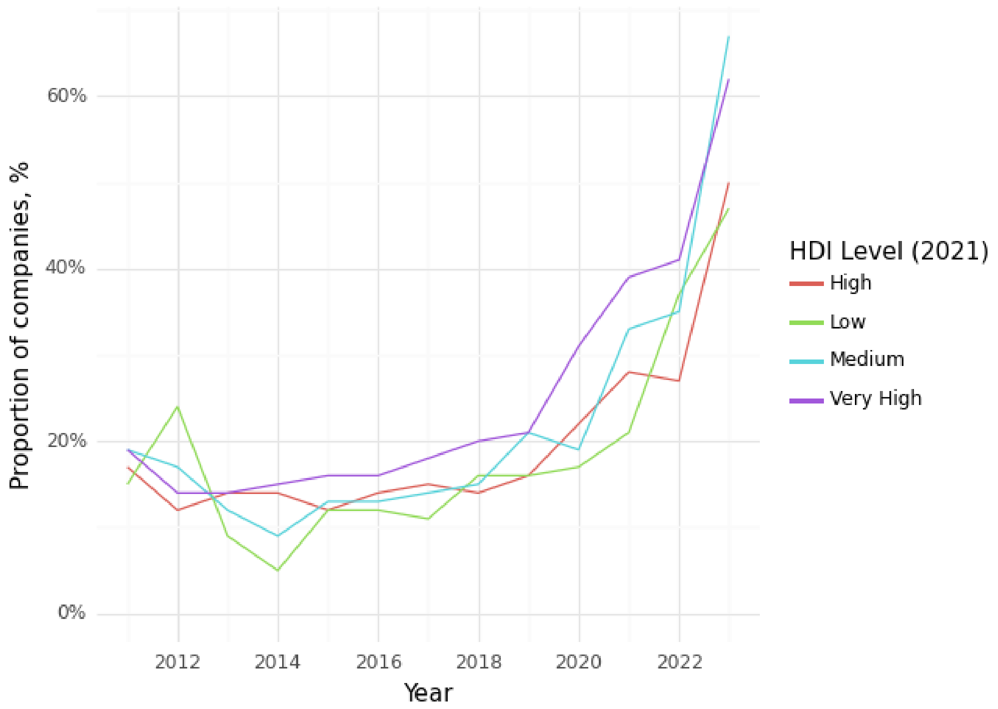

Figure 335: Proportion of companies referring to the climate change-health intersection by country HDI categories, 2011–2023.

References

1. United Nations Development Programme. Human Development Report 2020 - The Next Frontier: Human Development and the Anthropocene [Internet]. New York; 2020. Available from: https://hdr.undp.org/system/files/documents/hdr2020pdf.pdf

2. United Nations Development Programme. Human Development Report 2020: The Next Frontier: Human Development and the Anthropocene [Internet]. Human Development Reports. New York; 2020 Dec [cited 2024 Jan 15]. Available from: https://hdr.undp.org/content/human-development-report-2020

3. United Nations Development Programme. Human Development Index (HDI) [Internet]. United Nations Development Programme. 2021 [cited 2024 Jan 15]. Available from: https://hdr.undp.org/data-center/human-development-index#/indicies/HDI

4. Stalhandske Zélie, Chambers Jonathan. zeliest/lancet_countdown_heatwave_2024: Lancet Countdown indicator 1.1.2 2024. 2024.

5. De Perez EC, Van Aalst M, Bischiniotis K, Mason S, Nissan H, Pappenberger F, et al. Global predictability of temperature extremes. Environmental Research Letters [Internet]. 2018 May 2 [cited 2024 Mar 8];13(5):054017. Available from: https://iopscience.iop.org/article/10.1088/1748-9326/aab94a

6. Di Napoli C, Pappenberger F, Cloke HL. Verification of Heat Stress Thresholds for a Health-Based Heat-Wave Definition. J Appl Meteorol Climatol [Internet]. 2019 Jun 1 [cited 2024 Mar 8];58(6):1177–94. Available from: https://journals.ametsoc.org/view/journals/apme/58/6/jamc-d-18-0246.1.xml

7. Xu Z, Sheffield PE, Su H, Wang X, Bi Y, Tong S. The impact of heat waves on children's health: a systematic review. Int J Biometeorol [Internet]. 2014 Mar [cited 2024 Mar 8];58(2):239–47. Available from: https://pubmed.ncbi.nlm.nih.gov/23525899/

8. Chambers J. Global and cross-country analysis of exposure of vulnerable populations to heatwaves from 1980 to 2018. Clim Change [Internet]. 2020 Nov 1 [cited 2024 Mar 8];163(1):539–58. Available from: https://link.springer.com/article/10.1007/s10584-020-02884-2

9. School of Geography and Environmental Science U of SD of G and GU of LD de GU de N, and Center for International Earth Science Information Network (CIESIN) CU. WorldPop [Internet]. 2018. Available from: https://hub.worldpop.org/doi/10.5258/SOTON/WP00654

10. Hybrid gridded demographic data for the world, 1950-2020 0.25° resolution. [cited 2024 Mar 8]; Available from: https://zenodo.org/records/6011021

11. Romanello M, Di Napoli C, Green C, Kennard H, Lampard P, Scamman D, et al. The 2023 report of the Lancet Countdown on health and climate change: the imperative for a health-centred response in a world facing irreversible harms. Lancet. 2023;

12. Jay O, Broderick C, Smallcombe J. Extreme Heat Policy [Internet]. 2021. Available from: https://sma.org.au/wp-content/uploads/2021/02/SMA-Extreme-Heat-Policy-2021-Final.pdf

13. Center For International Earth Science Information Network-CIESIN-Columbia University. Gridded Population of the World, Version 4 (GPWv4): Population Count Adjusted to Match 2015 Revision of UN WPP Country Totals, Revision 11 [Internet]. [object Object]; Available from: https://sedac.ciesin.columbia.edu/data/set/gpw-v4-population-count-adjusted-to-2015-unwpp-country-totals-rev11

14. Hersbach H, Bell B, Berrisford P, Biavati G, Horányi A, Muñoz Sabater J, et al. ERA5 monthly averaged data on single levels from 1979 to present [Internet]. Copernicus Climate Change Service; 2019. Available from: https://doi.org/10.24381/cds.f17050d7

15. Wee J, Xiang R, Tan SH, Gunther M, Ihsan M, Khee S, et al. Effects of Medications on Heat Loss Capacity in Chronic Disease Patients: Health Implications Amidst Global Warming. 2023; Available from: https://www.cdc.gov/

16. Vanos JK, Baldwin JW, Jay O, Ebi KL. Simplicity lacks robustness when projecting heat-health outcomes in a changing climate. Vol. 11, Nature Communications. Nature Research; 2020.

17. Kjellstrom T, Freyberg C, Lemke B, Otto M, Briggs D. Estimating population heat exposure and impacts on working people in conjunction with climate change. Int J Biometeorol. 2018;62:291–306.

18. Liljegren JC, Carhart RA, Lawday P, Tschopp S, Sharp R. Modeling the Wet Bulb Globe Temperature Using Standard Meteorological Measurements. J Occup Environ Hyg. 2008;5(10):645–55.

19. Hersbach H, Bell B, Berrisford P, Hirahara S, Horanyi A, Munoz-Sabater J. The ERA5 global reanalysis. Quarterly Journal Royal Meteorological Society. 2020;146(730):1999–2049.

20. (SEDAC) NSD and AC. Gridded Population of the World (GPWv4) [Internet]. 2021. Available from: beta.sedac.ciesin.columbia.edu/data/collection/gpw-v4.

21. Organisation IL. ILOSTAT database [Internet]. 2021. Available from: ilostat.ilo.org/data

22. Project TISIMI. ISIMIP3b Bias Adjustment [Internet]. 2022. Available from: www.isimip.org/gettingstarted/isimip3b-bias-adjustment/

23. Yilmaz M. Accuracy assessment of temperature trends from ERA5 and ERA5-Land. Science of the Total Environment. 2023;856(159182):1–14.

24. Kjellstrom T, Maître N, Saget C, Otto M, Karimova T, Luu T, et al. Working on a warmer planet: The impact of heat stress on labour productivity and decent work [Internet]. Geneva, Switzerland: International Labour Office; 2019. Available from: https://www.preventionweb.net/publication/working-warmer-planet-impact-heat-stress-labour-productivity-and-decent-work

25. World Economic Forum. Extreme heat is forcing Spain's outside workers to shift their hours. 2023.

26. International Organization for Standardization Technical Committee. ISO 7243:2017 Ergonomics of the thermal environment Assessment of heat stress using the WBGT (wet bulb globe temperature) index. 2017.

27. NIOSH. Criteria for a Recommended Standard: Occupational Exposure to Heat and Hot Environments.

28. Lind AR. A physiological criterion for setting thermal environmental limits for everyday work. J Appl Physiol. 1963;

29. Kjellstrom T, Freyberg C, Lemke B, Otto M, Briggs D. Estimating population heat exposure and impacts on working people in conjunction with climate change. Int J Biometeorol [Internet]. 2018;62(3):291–306. Available from: https://doi.org/10.1007/s00484-017-1407-0

30. Romello M et al. The 2019 Report of The Lancet Countdown on Health and Climate Change. 2019.

31. World Health Organization, International Labour Organization. WHO/ILO Joint Estimates of the Work-related Burden of Disease and Injury, 2000-2016: Technical Report. Geneva; 2021.

32. World Health Organization, International Labour Organization. WHO/ILO joint estimates of the work-related burden of disease and injury, 2000-2016: global monitoring report. Geneva, Switzerland; 2021.

33. Pega F, Hamzaoui H, Náfrádi B, Momen NC. Global, regional and national burden of disease attributable to 19 selected occupational risk factors for 183 countries, 2000–2016: A systematic analysis from the WHO/ILO Joint Estimates of the Work-related Burden of Disease and Injury. Scand J Work Environ Health. 2022;48(2):158–68.

34. Pega F, Momen NC, Streicher KN, Leon-Roux M, Neupane S, Schubauer-Berigan MK, et al. Global, regional and national burdens of non-melanoma skin cancer attributable to occupational exposure to solar ultraviolet radiation for 183 countries, 2000–2019: A systematic analysis from the WHO/ILO Joint Estimates of the Work-related Burden of Disease and Injury. Environ Int. 2023;181.

35. World Health Organization. Technical Advisory Group on Occupational Burden of Disease Estimation (TAG-OBoDE) [Internet]. 2023. Available from: https://www.who.int/groups/technical-advisory-group-on-occupational-burden-of-disease-estimation

36. Descatha A, Evanoff BA, Leclerc A. Job-Exposure Matrices: Design, Validation, and Limitations. In: Wahrendorf M, Chandola T, Descatha A, editors. Handbook of Life Course Occupational Health. Springer Cham; 2023. p. 77–94.

37. International Labour Organization. ISCO–08: International Standard Classification of Occupations. Geneva; 2012.

38. International Labour Organization. International Labour Organization. Geneva; 1987.

39. Goldstein H. Multilevel Statistical Models. 4th ed. UK: WIley; 2010.

40. Bonjour S, Adair-Rohani H, Wolf J, Bruce NG, Mehta S, Prüss-Ustün A, et al. Solid fuel use for household cooking: Country and regional estimates for 1980-2010. Environ Health Perspect. 2013;121(7):784–90.

41. Stoner O, Lewis J, Martínez IL, Gumy S, Economou T, Adair-Rohani H. Household cooking fuel estimates at global and country level for 1990 to 2030. Nat Commun. 2021;12(1).

42. Wolf J, Bonjour S, Prüss-Üstün A. An exploration of multilevel modeling for estimating access to drinking water and sanitation. J Water Health. 2013;11(1):64–77.

43. Pega F, Náfrádi B, Momen NC, Ujita Y, Streicher KN, Prüss-Üstün AM, et al. Global, regional, and national burdens of ischemic heart disease and stroke attributable to exposure to long working hours for 194 countries, 2000–2016: A systematic analysis from the WHO/ILO Joint Estimates of the Work-related Burden of Disease and Injury. Environ Int. 2021;154.

44. United Nations. SDG indicator metadata. Indicator 3.9.1 Mortality rate attributed to household and ambient air pollution [Internet]. 2022. Available from: https://unstats.un.org/sdgs/metadata/files/Metadata-03-09-01.pdf

45. United Nations. World Population Prospects 2022, Online Edition. [Internet]. Geneva: United Nations; 2022. Available from: https://population.un.org/wpp/

46. Hirshkowitz M, Whiton K, Albert SM, Alessi C, Bruni O, DonCarlos L, et al. National Sleep Foundation’s sleep time duration recommendations: methodology and results summary. Sleep Health [Internet]. 2015;1(1):40–3. Available from: http://dx.doi.org/10.1016/j.sleh.2014.12.010

47. Krause AJ, Simon E Ben, Mander BA, Greer SM, Saletin JM, Goldstein-Piekarski AN, et al. The sleep-deprived human brain. Nat Rev Neurosci [Internet]. 2017;18(7):404–18. Available from: http://dx.doi.org/10.1038/nrn.2017.55

48. Irwin MR. Why sleep is important for health: a psychoneuroimmunology perspective. Annu Rev Psychol [Internet]. 2015;66:143–72. Available from: http://dx.doi.org/10.1146/annurev-psych-010213-115205

49. Cappuccio FP, Cooper D, D’Elia L, Strazzullo P, Miller MA. Sleep duration predicts cardiovascular outcomes: a systematic review and meta-analysis of prospective studies. Eur Heart J [Internet]. 2011;32(12):1484–92. Available from: http://dx.doi.org/10.1093/eurheartj/ehr007

50. Cappuccio FP, D’Elia L, Strazzullo P, Miller MA. Sleep duration and all-cause mortality: a systematic review and meta-analysis of prospective studies. Sleep [Internet]. 2010;33(5):585–92. Available from: http://dx.doi.org/10.1093/sleep/33.5.585

51. Goldstein AN, Walker MP. The role of sleep in emotional brain function. Annu Rev Clin Psychol [Internet]. 2014;10:679–708. Available from: http://dx.doi.org/10.1146/annurev-clinpsy-032813-153716

52. Bernert RA, Kim JS, Iwata NG, Perlis ML. Sleep disturbances as an evidence-based suicide risk factor. Curr Psychiatry Rep [Internet]. 2015;17(3):554. Available from: http://dx.doi.org/10.1007/s11920-015-0554-4

53. Obradovich N, Migliorini R, Paulus MP, Rahwan I. Empirical evidence of mental health risks posed by climate change. Proc Natl Acad Sci U S A [Internet]. 2018;115(43):10953–8. Available from: http://dx.doi.org/10.1073/pnas.1801528115

54. Baylis P, Obradovich N, Kryvasheyeu Y, Chen H, Coviello L, Moro E, et al. Weather impacts expressed sentiment. PLoS One. 2018;13(4):e0195750.

55. Obradovich N, Fowler JH. Climate change may alter human physical activity patterns. Nat Hum Behav. 2017;1:0097.

56. Wang J, Obradovich N, Zheng S. A 43-million-person investigation into weather and expressed sentiment in a changing climate. One Earth [Internet]. 2020; Available from: https://www.sciencedirect.com/science/article/pii/S2590332220302529

57. Romanello M, McGushin A, Di Napoli C, Drummond P, Hughes N, Jamart L, et al. The 2021 report of the Lancet Countdown on health and climate change: code red for a healthy future. Lancet [Internet]. 2021;398(10311):1619–62. Available from: https://doi.org/10.1016/S0140-6736(21)01787-6

58. Obradovich N, Minor K. Identifying and Preparing for the Mental Health Burden of Climate Change. JAMA Psychiatry [Internet]. 2022;79(4):285–6. Available from: http://dx.doi.org/10.1001/jamapsychiatry.2021.4280

59. Belova A, Gould CA, Munson K, Howell M, Trevisan C, Obradovich N, et al. Projecting the Suicide Burden of Climate Change in the United States. Geohealth [Internet]. 2022;6(5):e2021GH000580. Available from: http://dx.doi.org/10.1029/2021GH000580

60. Mullins JT, White C. Temperature and mental health: Evidence from the spectrum of mental health outcomes. J Health Econ [Internet]. 2019;68:102240. Available from: http://dx.doi.org/10.1016/j.jhealeco.2019.102240

61. Burke M, González F, Baylis P, Heft-Neal S, Baysan C, Basu S, et al. Higher temperatures increase suicide rates in the United States and Mexico. Nat Clim Chang [Internet]. 2018; Available from: https://doi.org/10.1038/s41558-018-0222-x

62. Minor K, Bjerre-Nielsen A, Jonasdottir SS, Lehmann S, Obradovich N. Rising temperatures erode human sleep globally. One Earth [Internet]. 2022;5(5):534–49. Available from: https://www.cell.com/one-earth/pdf/S2590-3322(22)00209-3.pdf

63. Obradovich N, Migliorini R, Mednick SC, Fowler JH. Nighttime temperature and human sleep loss in a changing climate. Sci Adv. 2017;3(5):e1601555.

64. Rifkin DI, Long MW, Perry MJ. Climate change and sleep: A systematic review of the literature and conceptual framework. Sleep Med Rev [Internet]. 2018;42:3–9. Available from: http://dx.doi.org/10.1016/j.smrv.2018.07.007

65. Obradovich N, Migliorini R. Sleep and the human impacts of climate change. Sleep Med Rev [Internet]. 2018;42:1–2. Available from: http://dx.doi.org/10.1016/j.smrv.2018.09.002

66. Chevance G, Minor K, Vielma C, Campi E, O'Callaghan-Gordo C, Basagaña X, et al. A systematic review of ambient heat and sleep in a warming climate. Sleep Med Rev [Internet]. 2024;101915. Available from: https://www.sciencedirect.com/science/article/pii/S1087079224000194

67. Hsiang S. Climate Econometrics. Annu Rev Resour Economics. 2016;8(1):43–75.

68. Chevance G, Minor K, Vielma C, Campi E, O'Callaghan-Gordo C, Basagaña X, et al. A systematic review of ambient heat and sleep in a warming climate. Sleep Med Rev [Internet]. 2024 Jun 1 [cited 2024 May 8];75. Available from: https://pubmed.ncbi.nlm.nih.gov/38598988/

69. Obradovich N, Migliorini R, Mednick SC, Fowler JH. Nighttime temperature and human sleep loss in a changing climate. Sci Adv [Internet]. 2017 May 1 [cited 2024 Aug 1];3(5). Available from: https://pubmed.ncbi.nlm.nih.gov/28560320/

70. Minor K, Bjerre-Nielsen A, Jonasdottir SS, Lehmann S, Obradovich N. Rising temperatures erode human sleep globally. One Earth. 2022 May 20;5(5):534–49.

71. Hersbach H, Bell B, Berrisford P, Hirahara S, Horányi A, Muñoz-Sabater J, et al. The ERA5 global reanalysis. Quart J Roy Meteor Soc [Internet]. 2020;146(730):1999–2049. Available from: https://onlinelibrary.wiley.com/doi/abs/10.1002/qj.3803

72. Doxsey-Whitfield E, Macmanus K, Adamo SB, Pistolesi L, Squires J, Borkovska O, et al. Taking Advantage of the Improved Availability of Census Data: A First Look at the Gridded Population of the World, Version 4 (GPWv4). 2014.

73. Minor K, Bjerre-Nielsen A, Jonasdottir SS, Lehmann S, Obradovich N. Rising temperatures erode human sleep globally. One Earth. 2022 May 20;5(5):534–49.

74. Mérel P, Gammans M. Climate Econometrics: Can the Panel Approach Account for Long-Run Adaptation? Am J Agric Econ [Internet]. 2021;103(4):1207–38. Available from: https://onlinelibrary.wiley.com/doi/abs/10.1111/ajae.12200

75. Gilford DM, Pershing AJ, Giguere J, Otto FEL. Human Fingerprints on Daily Temperatures in 2022. Bull Am Meteorol Soc. 2024;accepted (minor rev).

76. Gilford DM, Pershing AJ, Strauss BH, Haustein K, Otto FEL. A multi-method framework for global real-time climate attribution. Adv Stat Climatol Meteorol Oceanogr. 2022;8:135–54.

77. Lange S. Trend-preserving bias adjustment and statistical downscaling with ISIMIP3BASD (v1. 0). Geosci Model Dev. 2019;12(7):3055–70.

78. Muñoz-Sabater J, Dutra E, Agustí-Panareda A, Albergel C, Arduini G, Balsamo G, et al. ERA5-Land: a state-of-the-art global reanalysis dataset for land applications. Earth Syst Sci Data [Internet]. 2021;13(9):4349–83. Available from: https://essd.copernicus.org/articles/13/4349/2021/

79. NASA Socioeconomic Data and Applications Center (SEDAC) Gridded Population of the World (GPWv4). Available at https://beta.sedac.ciesin.columbia.edu/data/collection/gpw-v4. 2021;

80. Gasparrini A, Guo Y, Hashizume M. Mortalité attribuable au froid et à la chaleur : Analyse multi-pays. Environnement, Risques et Sante. 2015 Nov 1;14(6):464–5.

81. Global Burden of Disease Collaborative Network. Global Burden of Disease Study 2019 (GBD 2019) Particulate Matter Risk Curves [Internet]. Seattle, United States of America: Institute for Health Metrics and Evaluation (IHME). 2021. Available from: https://ghdx.healthdata.org/record/ihme-data/global-burden-disease-study-2019-gbd-2019-particulate-matter-risk-curves

82. Honda Y, Kondo M, McGregor G, Kim H, Guo YL, Hijioka Y, et al. Heat-related mortality risk model for climate change impact projection. Environ Health Prev Med. 2014 Jan;19(1):56–63.

83. Luedeling E. Interpolating hourly temperatures for computing agroclimatic metrics. Int J Biometeorol. 2018;62:1799–807.

84. Hersbach H, Bell B, Berrisford P, Hirahara S, Horányi A, Muñoz-Sabater J, et al. The ERA5 global reanalysis. Quarterly Journal of the Royal Meteorological Society. 2020;146(730):1999–2049.

85. ISIMIP. The Inter-Sectoral Impact Model Intercomparison Project (ISIMP). Input data set: Historical, gridded population. Available at https://www.isimip.org/gettingstarted/input-data-bias-correction/details/31/. 2021;

86. University C for IESINCC. Gridded Population of the World, Version 4 (GPWv4): Population Count Adjusted to Match 2015 Revision of UN WPP Country Totals, Revision 11 [Internet]. 2018. Available from: https://doi.org/10.7927/H4PN93PB

87. Fire danger indices historical data from the Copernicus Emergency Management Service. Copernicus Climate Change Service (C3S) Climate Data Store (CDS). 2019.

88. CIESIN. Documentation for Gridded Population of the World, Version 4 (GPWv4). Palisades NY: NASA Socioeconomic Data and Applications Center (SEDAC); 2016.

89. F.I.R.M.S. Fire Information for Resource Management System (FIRMS). Fire Information for Resource Management System (FIRMS [Internet]. 2024 [cited 2024 Mar 1]. Available from: https://www.earthdata.nasa.gov/learn/find-data/near-real-time/firms

90. EarthEnv. Global, remote-sensing supported environmental layers for assessing status and trends in biodiversity, ecosystems, and climate [Internet]. Available from: https://www.earthenv.org/cloud

91. Wilson Adam M. AND Jetz W. Remotely Sensed High-Resolution Global Cloud Dynamics for Predicting Ecosystem and Biodiversity Distributions. PLoS Biol [Internet]. 2016 Apr;14(3):1–20. Available from: https://doi.org/10.1371/journal.pbio.1002415

92. Hänninen R, Sofiev M, Uppstu A, Kouznetsov R. Daily surface concentration of fire related PM2.5 for 2003-2023, modelled by SILAM CTM when using the MODIS satellite data for the fire radiative power [Internet]. Finnish Meteorological Institute; 2024. Available from: https://fmi.b2share.csc.fi/records/d1cac971b3224d438d5304e945e9f16c

93. Sofiev M, Vankevich R, Lotjonen M, Prank M, Petukhov V, Ermakova T, et al. An operational system for the assimilation of the satellite information on wild-land fires for the needs of air quality modelling and forecasting. Atmos Chem Phys [Internet]. 2009;9(18):6833–47. Available from: https://acp.copernicus.org/articles/9/6833/2009/

94. Sofiev M, Vankevich R, Ermakova T, Hakkarainen J. Global mapping of maximum emission heights and resulting vertical profiles of wildfire emissions. Atmos Chem Phys [Internet]. 2013;13(14):7039–52. Available from: https://acp.copernicus.org/articles/13/7039/2013/

95. Sofiev M, Ermakova T, Vankevich R. Evaluation of the smoke-injection height from wild-land fires using remote-sensing data. Atmos Chem Phys [Internet]. 2012;12(4):1995–2006. Available from: https://acp.copernicus.org/articles/12/1995/2012/

96. Soares J, Sofiev M, Hakkarainen J. Uncertainties of wild-land fires emission in AQMEII phase 2 case study. Atmos Environ [Internet]. 2015;115:361–70. Available from: https://www.sciencedirect.com/science/article/pii/S1352231015000916

97. Sofiev M, Vira J, Kouznetsov R, Prank M, Soares J, Genikhovich E. Construction of the SILAM Eulerian atmospheric dispersion model based on the advection algorithm of Michael Galperin. Geosci Model Dev [Internet]. 2015;8(11):3497–522. Available from: https://gmd.copernicus.org/articles/8/3497/2015/

98. Kollanus V, Prank M, Gens A, Soares J, Vira J, Kukkonen J, et al. Mortality due to Vegetation Fire-Originated PM2.5 Exposure in Europe-Assessment for the Years 2005 and 2008. Environmental Health Rerspectives [Internet]. 2017;125(1):30–7. Available from: https://ehp.niehs.nih.gov/doi/10.1289/EHP194

99. Wooster MJ, Zhukov B, Oertel D. Fire radiative energy for quantitative study of biomass burning: derivation from the BIRD experimental satellite and comparison to MODIS fire products. Remote Sens Environ [Internet]. 2003;86(1):83–107. Available from: https://www.sciencedirect.com/science/article/pii/S0034425703000701

100. Wooster MJ, Roberts G, Perry GLW, Kaufman YJ. Retrieval of biomass combustion rates and totals from fire radiative power observations: FRP derivation and calibration relationships between biomass consumption and fire radiative energy release. Journal of Geophysical Research: Atmospheres [Internet]. 2005;110(D24311). Available from: https://agupubs.onlinelibrary.wiley.com/doi/abs/10.1029/2005JD006318

101. Global, remote-sensing supported environmental layers for assessing status and trends in biodiversity, ecosystems, and climate [Internet]. 2023. Available from: https://www.earthenv.org/cloud

102. Champeaux JL, Masson V, Chauvin F. ECOCLIMAP: a global database of land surface parameters at 1 km resolution. Meteorological Applications [Internet]. 2005;12(1):29–32. Available from: https://rmets.onlinelibrary.wiley.com/doi/abs/10.1017/S1350482705001519

103. Hänninen R, Sofiev M, Uppstu A, Kouznetsov R. Daily surface concentration of fire related PM2.5 for 2003-2021, modelled by SILAM CTM when using the MODIS satellite data for the fire radiative power [Internet]. Finnish Meteorological Institute.; 2022. Available from: https://fmi.b2share.csc.fi/records/a006840cce9340e8bf11e562bb8d396e

104. Beguería S, Vicente-Serrano SM, Reig F, Latorre B. Standardized precipitation evapotranspiration index (SPEI) revisited: parameter fitting, evapotranspiration models, tools, datasets and drought monitoring. Int J Climatol [Internet]. 2014 Aug 1 [cited 2024 Mar 20];34(10):3001–23. Available from: https://onlinelibrary.wiley.com/doi/full/10.1002/joc.3887

105. Fan Y, van den Dool H. A global monthly land surface air temperature analysis for 1948–present. J Geophys Res [Internet]. 2008 Jan 16 [cited 2024 Mar 20];113(D1):1103. Available from: https://onlinelibrary.wiley.com/doi/full/10.1029/2007JD008470

106. Schamm K, Ziese M, Becker A, Finger P, Meyer-Christoffer A, Schneider U, et al. Global gridded precipitation over land: A description of the new GPCC First Guess Daily product. Earth Syst Sci Data. 2014 Jan 27;6(1):49–60.

107. MeteoSwiss. Drought Indices [Internet]. [cited 2024 Mar 20]. Available from: https://www.meteoswiss.admin.ch/climate/climate-change/heatwaves-droughts-cold-and-snowfall/climate-indices/drought-indices.html

108. Consejo Superior de Investigaciones Cientificas. Global SPEI Database [Internet]. [cited 2024 Mar 20]. Available from: https://spei.csic.es/database.html

109. Romanello M, di Napoli C, Green C, Et al. The 2023 report of the Lancet Countdown on health and climate change: the imperative for a health-centred response in a world facing irreversible harms. The Lancet. 2023;

110. Muñoz-Sabater J, Dutra E, Agustí-Panareda A, Albergel C, Arduini G, Balsamo G, et al. ERA5-Land: A state-of-the-art global reanalysis dataset for land applications. Earth Syst Sci Data. 2021;13(9):4349–83.

111. Ebi KL, Vanos J, Baldwin JW, Bell JE, Hondula DM, Errett NA, et al. Extreme Weather and Climate Change: Population Health and Health System Implications. Annu Rev Public Health. 20210106th ed. 2021;42:293–315.

112. He C, Kim H, Hashizume M, Lee W, Honda Y, Kim SE, et al. The overlooked health impacts of extreme rainfall exposure in 30 East Asian cities. Nat Sustain [Internet]. 2024 Apr 19;1–9. Available from: https://www.nature.com/articles/s41893-024-01294-x

113. Kreienkamp F, Philip SY, Tradowsky JS, Kew SF, Lorenz P, Arrighi J, et al. Rapid attribution of heavy rainfall events leading to the severe flooding in Western Europe during July 2021. World Weather Attribution [Internet]. 2021; Available from: https://floodresilience.net/resources/item/rapid-attribution-of-heavy-rainfall-events-leading-to-the-severe-flooding-in-western-europe-during-july-2021/

114. IPCC. Climate Change 2022: Mitigation of Climate Change. Contribution of Working Group III to the Sixth Assessment Report of the Intergovernmental Panel on Climate Change. Shukla PR, Skea J, Slade R, Khourdajie A Al, Diemen R van, McCollum D, et al., editors. Cambridge, UK and New York, NY, USA: Cambridge University Press; 2022.

115. Hersbach H, Bell B, Berrisford P, Hirahara S, Horanyi A, Munoz-Sabater J, et al. The ERA5 global reanalysis. Quarterly Journal of the Royal Meteorological Society. 2020;146(730):1999–2049.

116. Lavers DA, Simmons A, Vamborg F, Rodwell MJ. An evaluation of ERA5 precipitation for climate monitoring. Quarterly Journal of the Royal Meteorological Society [Internet]. 2022 Jan 12;148(748):3152–65. Available from: https://onlinelibrary.wiley.com/doi/abs/10.1002/qj.4351

117. Myhre G, Alterskjær K, Stjern CW, Hodnebrog Ø, Marelle L, Samset BH, et al. Frequency of extreme precipitation increases extensively with event rareness under global warming. Sci Rep. 2019;9(1):1–10.

118. Dunn RJH, Alexander L V, Donat MG, Zhang X, Bador M, Herold N, et al. Development of an Updated Global Land In Situ-Based Data Set of Temperature and Precipitation Extremes: HadEX3. Journal of Geophysical Research: Atmospheres [Internet]. 2020;125(16):e2019JD032263. Available from: https://agupubs.onlinelibrary.wiley.com/doi/abs/10.1029/2019JD032263

119. Contractor S, Donat MG, Alexander L V. Changes in observed daily precipitation over global land areas since 1950. J Clim. 2021;34(1):3–19.

120. Alexander L V. Global observed long-term changes in temperature and precipitation extremes: A review of progress and limitations in IPCC assessments and beyond. Weather Clim Extrem [Internet]. 2016 Jan 12;11:4–16. Available from: https://www.sciencedirect.com/science/article/pii/S2212094715300414

121. Romanello M, Napoli C di, Green C, Kennard H, Lampard P, Scamman D, et al. The 2023 report of the Lancet Countdown on health and climate change: the imperative for a health-centred response in a world facing irreversible harms. The Lancet [Internet]. 2023 Dec 16 [cited 2024 Mar 13];402(10419):2346–94. Available from: http://www.thelancet.com/article/S0140673623018597/fulltext

122. Bandhauer M, Isotta F, Lakatos M, Lussana C, Båserud L, Izsák B, et al. Evaluation of daily precipitation analyses in E-OBS (v19.0e) and ERA5 by comparison to regional high-resolution datasets in European regions. International Journal of Climatology [Internet]. 2022 Jan 12;42(2):727–47. Available from: https://onlinelibrary.wiley.com/doi/abs/10.1002/joc.7269

123. Beck HE, Pan M, Roy T, Weedon GP, Pappenberger F, van Dijk AIJM, et al. Daily evaluation of 26 precipitation datasets using Stage-IV gauge-radar data for the CONUS. Hydrol Earth Syst Sci [Internet]. 2019 Jan 12;23(1):207–24. Available from: https://hess.copernicus.org/articles/23/207/2019/

124. Jiang Q, Li W, Fan Z, He X, Sun W, Chen S, et al. Evaluation of the ERA5 reanalysis precipitation dataset over Chinese Mainland. J Hydrol (Amst) [Internet]. 2021 Jan 12;595:125660. Available from: https://www.sciencedirect.com/science/article/pii/S0022169420311215

125. Romanello M, Di Napoli C, Drummond P, Green C, Kennard H, Lampard P, et al. The 2022 report of the Lancet Countdown on health and climate change: health at the mercy of fossil fuels. Lancet [Internet]. 2022;400(10363):1619–54. Available from: http://dx.doi.org/10.1016/S0140-6736(22)01540-9

126. Baylis P. Temperature and temperament: Evidence from Twitter. J Public Econ [Internet]. 2020;184:104161. Available from: https://www.sciencedirect.com/science/article/pii/S0047272720300256

127. Burke M, González F, Baylis P, Heft-Neal S, Baysan C, Basu S, et al. Higher temperatures increase suicide rates in the United States and Mexico. Nat Clim Chang [Internet]. 2018; Available from: https://doi.org/10.1038/s41558-018-0222-x

128. Moore FC, Obradovich N, Lehner F, Baylis P. Rapidly declining remarkability of temperature anomalies may obscure public perception of climate change. Proc Natl Acad Sci U S A [Internet]. 2019;116(11):4905–10. Available from: http://dx.doi.org/10.1073/pnas.1816541116

129. Carleton TA, Hsiang SM. Social and economic impacts of climate. Science (1979) [Internet]. 2016;353(6304). Available from: http://dx.doi.org/10.1126/science.aad9837

130. Diener E, Larsen RJ, Levine S, Emmons RA. Intensity and frequency: dimensions underlying positive and negative affect. J Pers Soc Psychol. 1985;48(5):1253.

131. Pennebaker JW, Boyd RL, Jordan K, Blackburn K. The development and psychometric properties of LIWC2015. 2015; Available from: https://repositories.lib.utexas.edu/handle/2152/31333

132. Tausczik YR, Pennebaker JW. The psychological meaning of words: LIWC and computerized text analysis methods. J Lang Soc Psychol. 2010;29(1):24–54.

133. Coppersmith G, Dredze M, Harman C. Quantifying Mental Health Signals in Twitter. In: Resnik P, Resnik R, Mitchell M, editors. Proceedings of the Workshop on Computational Linguistics and Clinical Psychology: From Linguistic Signal to Clinical Reality [Internet]. Baltimore, Maryland, USA: Association for Computational Linguistics; 2014. p. 51–60. Available from: https://aclanthology.org/W14-3207

134. Kahn JH, Tobin RM, Massey AE, Anderson JA. Measuring emotional expression with the Linguistic Inquiry and Word Count. Am J Psychol [Internet]. 2007;120(2):263–86. Available from: https://www.ncbi.nlm.nih.gov/pubmed/17650921

135. Beasley A, Mason W. Emotional States vs. Emotional Words in Social Media. In: Proceedings of the ACM Web Science Conference [Internet]. New York, NY, USA: Association for Computing Machinery; 2015. (WebSci '15). Available from: https://doi.org/10.1145/2786451.2786473

136. Settanni M, Marengo D. Sharing feelings online: studying emotional well-being via automated text analysis of Facebook posts. Front Psychol. 2015;6.

137. Myhre G, Alterskjær K, Stjern CW, Hodnebrog Ø, Marelle L, Samset BH, et al. Frequency of extreme precipitation increases extensively with event rareness under global warming. Sci Rep [Internet]. 2019;9(1):16063. Available from: http://dx.doi.org/10.1038/s41598-019-52277-4

138. Dunn RJH, Alexander L V, Donat MG, Zhang X, Bador M, Herold N, et al. Development of an updated global land in situ-based data set of temperature and precipitation extremes: HadEX3. J Geophys Res [Internet]. 2020;125(16):e2019JD032263. Available from: https://agupubs.onlinelibrary.wiley.com/doi/10.1029/2019JD032263

139. Contractor S, Donat MG, Alexander L V. Changes in Observed Daily Precipitation over Global Land Areas since 1950. J Clim [Internet]. 2021;34(1):3–19. Available from: https://journals.ametsoc.org/view/journals/clim/34/1/jcliD190965.xml?tab_body=fulltext-display

140. Alexander L V. Global observed long-term changes in temperature and precipitation extremes: A review of progress and limitations in IPCC assessments and beyond. Weather Clim Extrem [Internet]. 2016;11:4–16. Available from: https://www.sciencedirect.com/science/article/pii/S2212094715300414

141. Obradovich N, Tingley D, Rahwan I. Effects of environmental stressors on daily governance. Proc Natl Acad Sci U S A [Internet]. 2018;115(35):8710–5. Available from: http://dx.doi.org/10.1073/pnas.1803765115

142. Obradovich N, Rahwan I. Risk of a feedback loop between climatic warming and human mobility. J R Soc Interface [Internet]. 2019;16(158):20190058. Available from: http://dx.doi.org/10.1098/rsif.2019.0058

143. Minor K, Moro E, Obradovich N. Adverse weather amplifies social media activity [Internet]. 2023 [cited 2023 Mar 2]. Available from: http://arxiv.org/abs/2302.08456

144. Programme UND. Human Development Report 2021-22: Uncertain Times, Unsettled Lives: Shaping our Future in a Transforming World. UNDP; 2022.

145. Ramadona AL, Tozan Y, Lazuardi L, Rocklöv J. A combination of incidence data and mobility proxies from social media predicts the intra-urban spread of dengue in Yogyakarta, Indonesia. PLoS Negl Trop Dis [Internet]. 2019 Apr 1 [cited 2024 Apr 9];13(4):e0007298. Available from: https://journals.plos.org/plosntds/article?id=10.1371/journal.pntd.0007298

146. Rocklöv J, Tozan Y, Ramadona A, Sewe MO, Sudre B, Garrido J, et al. Using Big Data to Monitor the Introduction and Spread of Chikungunya, Europe, 2017 - Volume 25, Number 6—June 2019 - Emerging Infectious Diseases journal - CDC. Emerg Infect Dis [Internet]. 2019 Jun 1 [cited 2024 Apr 9];25(6):1041–9. Available from: https://wwwnc.cdc.gov/eid/article/25/6/18-0138_article

147. Semenza JC, Sudre B, Miniota J, Rossi M, Hu W, Kossowsky D, et al. International Dispersal of Dengue through Air Travel: Importation Risk for Europe. PLoS Negl Trop Dis [Internet]. 2014 Dec 4 [cited 2024 Apr 9];8(12):e3278. Available from: https://journals.plos.org/plosntds/article?id=10.1371/journal.pntd.0003278

148. Dengue- Global situation [Internet]. [cited 2024 Mar 21]. Available from: https://www.who.int/emergencies/disease-outbreak-news/item/2023-DON498

149. Chikungunya [Internet]. [cited 2024 Apr 9]. Available from: https://www.who.int/health-topics/chikungunya#tab=tab_1

150. Zika virus [Internet]. [cited 2024 Apr 9]. Available from: https://www.who.int/news-room/fact-sheets/detail/zika-virus

151. Metelmann S, Liu X, Lu L, Caminade C, Liu K, Cao L, et al. Assessing the suitability for Aedes albopictus and dengue transmission risk in China with a delay differential equation model. 15(3):e0009153. Available from: https://journals.plos.org/plosntds/article?id=10.1371/journal.pntd.0009153

152. DiSera L, Sjödin H, Rocklöv J, Tozan Y, Súdre B, Zeller H, et al. The Mosquito, the Virus, the Climate: An Unforeseen Réunion in 2018. 4(8):e2020GH000253. Available from: https://onlinelibrary.wiley.com/doi/abs/10.1029/2020GH000253

153. Liu-Helmersson J, Rocklöv J, Sewe M, Brännström Å. Climate change may enable Aedes aegypti infestation in major European cities by 2100. 172:693–9. Available from: https://www.sciencedirect.com/science/article/pii/S0013935119301069

154. Cailly P, Tran A, Balenghien T, L'Ambert G, Toty C, Ezanno P. A climate-driven abundance model to assess mosquito control strategies. 227:7–17. Available from: https://www.sciencedirect.com/science/article/pii/S0304380011005229

155. Erguler K, Smith-Unna SE, Waldock J, Proestos Y, Christophides GK, Lelieveld J, et al. Large-Scale Modelling of the Environmentally-Driven Population Dynamics of Temperate Aedes albopictus (Skuse). 11(2):e0149282. Available from: https://journals.plos.org/plosone/article?id=10.1371/journal.pone.0149282

156. Erguler K, Smith-Unna SE, Waldock J, Proestos Y, Christophides GK, Lelieveld J, et al. Large-Scale Modelling of the Environmentally-Driven Population Dynamics of Temperate Aedes albopictus (Skuse). 11(2):e0149282. Available from: https://journals.plos.org/plosone/article?id=10.1371/journal.pone.0149282

157. Erickson RA, Presley SM, Allen LJS, Long KR, Cox SB. A stage-structured, Aedes albopictus population model. 221(9):1273–82. Available from: https://www.sciencedirect.com/science/article/pii/S0304380010000578

158. Jia P, Lu L, Chen X, Chen J, Guo L, Yu X, et al. A climate-driven mechanistic population model of Aedes albopictus with diapause. 9(1):175. Available from: https://doi.org/10.1186/s13071-016-1448-y

159. Tran A, L'Ambert G, Lacour G, Benoît R, Demarchi M, Cros M, et al. A Rainfall- and Temperature-Driven Abundance Model for Aedes albopictus Populations. 10(5):1698–719. Available from: https://www.mdpi.com/1660-4601/10/5/1698

160. Mordecai EA, Cohen JM, Evans M V, Gudapati P, Johnson LR, Lippi CA, et al. Detecting the impact of temperature on transmission of Zika, dengue, and chikungunya using mechanistic models. 11(4):e0005568. Available from: https://journals.plos.org/plosntds/article?id=10.1371/journal.pntd.0005568

161. Metelmann S, Caminade C, Jones AE, Medlock JM, Baylis M, Morse AP. The UK's suitability for Aedes albopictus in current and future climates. 16(152):20180761. Available from: https://royalsocietypublishing.org/doi/10.1098/rsif.2018.0761

162. Muñoz-Sabater J, Dutra E, Agustí-Panareda A, Albergel C, Arduini G, Balsamo G, et al. ERA5-Land: a state-of-the-art global reanalysis dataset for land applications. Earth Syst Sci Data. 2021 Sep 7;13(9):4349–83.

163. Muñoz Sabater J. ERA5-Land monthly averaged data from 1950 to present [Internet]. Copernicus Climate Change Service (C3S) Climate Data Store (CDS). 2019 [cited 2024 Mar 4]. Available from: https://cds.climate.copernicus.eu/cdsapp#!/dataset/reanalysis-era5-land-monthly-means?tab=overview

164. ISIMIP3a population input data [Internet]. ISIMIP Repository; 2022. Available from: https://doi.org/10.48364/ISIMIP.822480.2

165. Rocklöv J, Tozan Y. Climate change and the rising infectiousness of dengue. Emerg Top Life Sci [Internet]. 2019 May 5 [cited 2024 Apr 19];3(2):133. Available from: /pmc/articles/PMC7288996/

166. Colón-González FJ, Sewe MO, Tompkins AM, Sjödin H, Casallas A, Rocklöv J, et al. Projecting the risk of mosquito-borne diseases in a warmer and more populated world: a multi-model, multi-scenario intercomparison modelling study. Lancet Planet Health [Internet]. 2021 Jul 1 [cited 2024 Mar 21];5(7):e404–14. Available from: http://www.thelancet.com/article/S2542519621001327/fulltext

167. Liu Y, Lillepold K, Semenza JC, Tozan Y, Quam MBM, Rocklöv J. Reviewing estimates of the basic reproduction number for dengue, Zika and chikungunya across global climate zones. Vol. 182, Environmental Research. Academic Press Inc.; 2020.

168. The raster package — R Spatial [Internet]. Available from: https://rspatial.org/raster/pkg/index.html#google_vignette

169. Rocklöv J, Quam MB, Sudre B, German M, Kraemer MUG, Brady O, et al. Assessing Seasonal Risks for the Introduction and Mosquito-borne Spread of Zika Virus in Europe. EBioMedicine. 2016 Jul 1;9:250–6.

170. Hersbach H, Bell B, Berrisford P, Biavati G, Horányi A, Sabater JM, et al. ERA5 hourly data on single levels from 1979 to present. Copernicus Climate Change Service (C3S). 2018.

171. Alduchov OA, Meteorology REEJ of A, 1996 U. Improved Magnus form approximation of saturation vapor pressure. journals.ametsoc.org [Internet]. 1996; Available from: https://journals.ametsoc.org/view/journals/apme/35/4/1520-0450_1996_035_0601_imfaos_2_0_co_2.xml

172. Laporta GZ, Linton YM, Wilkerson RC, Bergo ES, Nagaki SS, Sant'Ana DC, et al. Malaria vectors in South America: Current and future scenarios. Parasit Vectors [Internet]. 2015 Aug;8(1):1–13. Available from: https://parasitesandvectors.biomedcentral.com/articles/10.1186/s13071-015-1038-4

173. Sinka ME, Rubio-Palis Y, Manguin S, Patil AP, Temperley WH, Gething PW, et al. The dominant Anopheles vectors of human malaria in the Americas: occurrence data, distribution maps and bionomic précis. Parasites & Vectors 2010 3:1 [Internet]. 2010 Aug;3(1):1–26. Available from: https://parasitesandvectors.biomedcentral.com/articles/10.1186/1756-3305-3-72

174. Guerra CA, Snow RW, Hay SI. A global assessment of closed forests, deforestation and malaria risk. Ann Trop Med Parasitol. 2006 Apr;100(3):189–204.

175. Grover-Kopec EK, Blumenthal MB, Ceccato P, Dinku T, Omumbo JA, Connor SJ. Web-based climate information resources for malaria control in Africa. Malar J [Internet]. 2006 May;5. Available from: https://pubmed.ncbi.nlm.nih.gov/16689992/

176. Patz JA, Graczyk TK, Geller N, Vittor AY. Effects of environmental change on emerging parasitic diseases. Int J Parasitol [Internet]. 2000;30(12):1395–405. Available from: https://www.sciencedirect.com/science/article/pii/S0020751900001417

177. Gething PW, Patil AP, Smith DL, Guerra CA, Elyazar IRF, Johnston GL, et al. A new world malaria map: Plasmodium falciparum endemicity in 2010. Malar J [Internet]. 2011 Dec;10(1):1–

16. Available from: https://malariajournal.biomedcentral.com/articles/10.1186/1475-2875-10-378

178. Snow RW, Sartorius B, Kyalo D, Maina J, Amratia P, Mundia CW, et al. The prevalence of Plasmodium falciparum in sub-Saharan Africa since 1900. Nature [Internet]. 2017 Oct;550(7677):515–8. Available from: https://pubmed.ncbi.nlm.nih.gov/29019978/

179. Asenso-okyere K, Asante F, Tarekegn J, Andam KS. The linkages between agriculture and malaria: Issues for policy, research, and capacity strengthening. In 2009. Available from: https://api.semanticscholar.org/CorpusID:15159555

180. Allan R, Weetman D, Sauskojus H, Budge S, Hawail T Bin, Baheshm Y. Confirmation of the presence of Anopheles stephensi among internally displaced people's camps and host communities in Aden city, Yemen. Malar J. 2023 Apr;22(1):1.

181. Baker-Austin C, Trinanes JA, Taylor NGH, Hartnell R, Siitonen A, Martinez-Urtaza J. Emerging Vibrio risk at high latitudes in response to ocean warming. Nat Clim Chang [Internet]. 2013;3(1):73–7. Available from: https://doi.org/10.1038/nclimate1628

182. Jaime MU, Ronny van A, Michel A, Julie H, A MR, Joaquin T, et al. Genomic Variation and Evolution of Vibrio parahaemolyticus ST36 over the Course of a Transcontinental Epidemic Expansion. mBio [Internet]. 2017 Nov 14;8(6):10.1128/mbio.01425-17. Available from: https://doi.org/10.1128/mbio.01425-17

183. Parveen S, Hettiarachchi KA, Bowers JC, Jones JL, Tamplin ML, McKay R, et al. Seasonal distribution of total and pathogenic Vibrio parahaemolyticus in Chesapeake Bay oysters and waters. Int J Food Microbiol [Internet]. 2008;128(2):354–61. Available from: https://www.sciencedirect.com/science/article/pii/S0168160508005230

184. Baker-Austin C, Oliver JD, Alam M, Ali A, Waldor MK, Qadri F, et al. Vibrio spp. infections. Nat Rev Dis Primers [Internet]. 2018;4(1):1–19. Available from: https://doi.org/10.1038/s41572-018-0005-8

185. Semenza J, Triñanes J, Lohr W, Sudre B, Löfdahl M, Martinez-Urtaza J, et al. Environmental Suitability of Vibrio Infections in a Warming Climate: An Early Warning System. Environ Health Perspect. 2017 Oct 10;125.

186. Trinanes J, Martinez-Urtaza J. Future scenarios of risk of Vibrio infections in a warming planet: a global mapping study. Lancet Planet Health [Internet]. 2021;5(7):e426–35. Available from: https://www.sciencedirect.com/science/article/pii/S2542519621001698

187. Eyring V, Bony S, Meehl G, Senior C, Stevens B, Ronald S, et al. Overview of the Coupled Model Intercomparison Project Phase 6 (CMIP6) experimental design and organisation. Geoscientific Model Development Discussions. 2015 Dec 14;8:10539–83.

188. Warszawski L, Frieler K, Huber V, Piontek F, Serdeczny O, Schewe J. The Inter-Sectoral Impact Model Intercomparison Project (ISI–MIP): Project framework. Proceedings of the

National Academy of Sciences [Internet]. 2014 Mar 4;111(9):3228–32. Available from: https://doi.org/10.1073/pnas.1312330110

189. Newton A, Kendall M, Vugia D, Henao O, Mahon B. Increasing Rates of Vibriosis in the United States, 1996-2010: Review of Surveillance Data From 2 Systems. Clin Infect Dis. 2012 Jun 1;54 Suppl 5:S391-5.

190. Ralston EP, Kite-Powell H, Beet A. An estimate of the cost of acute health effects from food- and water-borne marine pathogens and toxins in the USA. J Water Health [Internet]. 2011 Apr 26;9(4):680–94. Available from: https://doi.org/10.2166/wh.2011.157

191. Good S, Fiedler E, Mao C, Martin MJ, Maycock A, Reid R, et al. The Current Configuration of the OSTIA System for Operational Production of Foundation Sea Surface Temperature and Ice Concentration Analyses. Remote Sens (Basel) [Internet]. 2020;12(4). Available from: https://www.mdpi.com/2072-4292/12/4/720

192. Copernicus Marine Service. Copernicus Marine Service. 2024.

193. Ronca SE, Ruff JC, Murray KO. A 20-year historical review of West Nile virus since its initial emergence in North America: Has West Nile virus become a neglected tropical disease? PLoS Negl Trop Dis. 2021 May 6;15(5):e0009190.

194. Epidemiological update: West Nile virus transmission season in Europe, 2018 [Internet]. [cited 2024 Mar 4]. Available from: https://www.ecdc.europa.eu/en/news-events/epidemiological-update-west-nile-virus-transmission-season-europe-2018

195. Heidecke J, Lavarello Schettini A, Rocklöv J. West Nile virus eco-epidemiology and climate change. PLOS Climate. 2023 May 1;2(5):e0000129.

196. Shocket MS, Verwillow AB, Numazu MG, Slamani H, Cohen JM, Moustaid F El, et al. Transmission of West Nile and five other temperate mosquito-borne viruses peaks at temperatures between 23°C and 26°C. Elife. 2020 Apr;9.

197. Sabater JM. ERA5-Land monthly averaged data from 1950 to present [Internet]. Copernicus Climate Change Service (C3S) Climate Data Store (CDS). 2019. Available from: https://cds.climate.copernicus.eu/cdsapp#!/dataset/reanalysis-era5-land-monthly-means?tab=overview

198. Muñoz-Sabater J, Dutra E, Agustí-Panareda A, Albergel C, Arduini G, Balsamo G, et al. ERA5-Land: a state-of-the-art global reanalysis dataset for land applications. Earth Syst Sci Data. 2021 Apr;13(9):4349–83.

199. Dasgupta S, Robinson EJZ. Attributing changes in food insecurity to a changing climate. Sci Rep. 2022;12(1):4709.

200. Monfreda C, Ramankutty N, Foley JA. Farming the planet: 2. Geographic distribution of crop areas, yields, physiological types, and net primary production in the year 2000. Global Biogeochem Cycles. 2008;22(1).

201. Muñoz-Sabater J, Dutra E, Agustí-Panareda A, Albergel C, Arduini G, Balsamo G, et al. ERA5-Land: A state-of-the-art global reanalysis dataset for land applications. Earth Syst Sci Data. 2021 Sep 7;13(9):4349–83.

202. Dasgupta S, Robinson EJZ. Improving Food Policies for a Climate Insecure World: Evidence from Ethiopia. Natl Inst Econ Rev. 2021;258:66–82.

203. Vicente-Serrano SM, Beguería S, López-Moreno JI. A Multiscalar Drought Index Sensitive to Global Warming: The Standardized Precipitation Evapotranspiration Index. J Climate. 2010;23(7):1696–718.

204. Cafiero C, Viviani S, Nord M. Food security measurement in a global context: The food insecurity experience scale. Measurement. 2018;116:146–52.

205. Dasgupta S, Maanen van N, Gosling SN, Piontek F, Otto C, Schleussner CF. Effects of climate change on combined labour productivity and supply: an empirical, multi-model study. Lancet Planet Health. 2021;5(7):e455–65.

206. Inter-Sectoral Impact Model Intercomparison Project. ISIMIP3 Protocol [Internet]. [cited 2024 Apr 23]. Available from: https://www.isimip.org/protocol/3/

207. Copernicus Climate Change Service (C3S). ORAS5 global ocean reanalysis monthly data from 1958 to present. Available at https://doi.org/10.24381/cds.67e8eeb7. 2023;

208. Souter D, Planes S, Wicquart J, Logan M, Obura D, Staub F. Status of Coral Reefs of the World: 2020 Summary for Policymakers Summary for Policymakers-Status of Coral Reefs of the World: 2020 Value of coral reefs.

209. International Union for Conservation of Nature. IUCN Oceans and coasts. 2024.

210. Ramsar. Ramsar; The convention on Wetlands, Country Profiles. 2024.

211. UNESCO. Technical Guidelines for Biosphere Reserves [Internet]. Available from: https://en.unesco.org/mab

212. World Health Organization. ATACH Community of Practice [Internet]. 2023 [cited 2024 Mar 14]. Available from: https://www.atachcommunity.com/

213. World Health Organization. 2021 WHO Health and Climate Change Survey Report. Geneva: WHO; 2021.

214. World Health Organization. COP26 Health Programme [Internet]. 2022 [cited 2024 Mar 14]. Available from: https://www.who.int/initiatives/alliance-for-transformative-action-on-climate-and-health/cop26-health-programme

215. UNDP. Human Development Index (HDI) [Internet]. 2021 [cited 2024 Apr 2]. Available from: https://hdr.undp.org/data-center/human-development-index#/indicies/HDI

216. World Meteorological Organization. World Meteorological Organization Country Profile database. WMO. 2022.

217. World Health Organization. 2023 WHO review of health in Nationally Determined Contributions and long-term strategies: health at the heart of the Paris Agreement. 2023.

218. World Meteorological Organization. 2023 State of Climate Services for Health [Internet]. Geneva; 2023. Available from: https://library.wmo.int/idurl/4/68500

219. Miettinen OS. Proportion of disease caused or prevented by a given exposure, trait or intervention. Am J Epidemiol [Internet]. 1974 May 1 [cited 2024 Mar 22];99(5):325–32. Available from: https://dx.doi.org/10.1093/oxfordjournals.aje.a121617

220. Vandentorren S, Bretin P, Zeghnoun A, Mandereau-Bruno L, Croisier A, Cochet C, et al. August 2003 Heat Wave in France: Risk Factors for Death of Elderly People Living at Home. Eur J Public Health. 2006 Dec 1;16(6):583–91.

221. Theocharis G, Tansarli GS, Mavros MN, Spiropoulos T, Barbas SG, Falagas ME. Association between use of air-conditioning or fan and survival of elderly febrile patients: a prospective study. European Journal of Clinical Microbiology & Infectious Diseases. 2013 Sep 27;32(9):1143–7.

222. Zhang Y, Nitschke M, Krackowizer A, Dear K, Pisaniello D, Weinstein P, et al. Risk factors for deaths during the 2009 heat wave in Adelaide, Australia: a matched case-control study. Int J Biometeorol. 2017 Jan 24;61(1):35–47.

223. Semenza JC, Rubin CH, Falter KH, Selanikio JD, Flanders WD, Howe HL, et al. Heat-Related Deaths during the July 1995 Heat Wave in Chicago. New England Journal of Medicine. 1996 Jul 11;335(2):84–90.

224. Naughton M. Heat-related mortality during a 1999 heat wave in Chicago. Am J Prev Med. 2002 May;22(4):221–7.

225. Marmor M. Heat wave mortality in nursing homes. Environ Res. 1978 Aug;17(1):102–15.

226. Kaiser R, Rubin CH, Henderson AK, Wolfe MI, Kieszak S, Parrott CL, et al. Heat-Related Death and Mental Illness During the 1999 Cincinnati Heat Wave. Am J Forensic Med Pathol. 2001 Sep;22(3):303–7.

227. Salines G, Lorente C, Serazin C, Adonias G, Gourvellec G, Lauzeille D, et al. Etude des facteurs de décès des personnes âgées résidant en établissement durant la vague de chaleur d'août 2003. 2005.

228. Ciancio BC, Di Renzi M, Binkin N, Perra A, Prato R, Bella A, et al. [Risk factors for mortality during a heat-wave in Bari (Italy), summer 2005.]. Ig Sanita Pubbl. 2007;63(2):113–25.

229. Strain T, Brage S, Sharp SJ, Richards J, Tainio M, Ding D, et al. Use of the prevented fraction for the population to determine deaths averted by existing prevalence of physical activity: a descriptive study. Lancet Glob Health [Internet]. 2020 Jul 1 [cited 2024 Mar 22];8(7):e920–30. Available from: http://www.thelancet.com/article/S2214109X20302114/fulltext

230. Bishop Y, Fienberg S, Holland P. Discrete Multivariate Analysis: Theory and Practice. Springer Science & Business Media; 2007.

231. Florczyk A, Melchiorri M, Corban C, Schiavina M, Maffenini L, Pesaresi M, et al. Description of the GHS Urban Centre Database 2015. Luxembourg: Publications Office of the European Union; 2019.

232. Center for International Earth Science Information Network - CIESIN - Columbia University. Gridded Population of the World, Version 4 (GPWv4): Population Density Adjusted to Match 2015 Revision UN WPP Country Totals, Revision 11. 2018.

233. Kriegler F, Malila W, Nalepka R, Richardson W. Preprocessing Transformations and Their Effects on Multispectral Recognition. Proceedings of the Sixth International Symposium on Remote Sensing of Environment. 1969;97.

234. Landsat Missions. Landsat Data Access [Internet]. [cited 2024 Mar 18]. Available from: https://www.usgs.gov/landsat-missions/landsat-data-access.

235. Beck HE, Zimmermann NE, McVicar TR, Vergopolan N, Berg A, Wood EF. Present and future Köppen-Geiger climate classification maps at 1-km resolution. Sci Data. 2018 Oct 30;5(1):180214.

236. UNDP. Human Development Index | Human Development Reports [Internet]. 2021 [cited 2024 Mar 18]. Available from: https://hdr.undp.org/data-center/human-development-index#/indicies/HDI

237. Rugel EJ, Henderson SB, Carpiano RM, Brauer M. Beyond the Normalized Difference Vegetation Index (NDVI): Developing a Natural Space Index for population-level health research. Environ Res. 2017 Nov;159:474–83.

238. Fong KC, Hart JE, James P. A Review of Epidemiologic Studies on Greenness and Health: Updated Literature Through 2017. Curr Environ Health Rep. 2018 Mar 1;5(1):77–87.

239. Iungman T, Cirach M, Marando F, Pereira Barboza E, Khomenko S, Masselot P, et al. Cooling cities through urban green infrastructure: a health impact assessment of European cities. The Lancet. 2023 Feb;401(10376):577–89.

240. World Health Organization. International Health Regulations. WHO; 2005.

241. World Health Organization. States Party Self-assessment Annual Reporting Tool [Internet]. e-SPAR. 2024 [cited 2024 Apr 2]. Available from: https://extranet.who.int/e-spar/

242. ASPHER. ASPHER Climate and Health Competencies for Public Health Professionals in Europe [Internet]. 2021 [cited 2024 Mar 14]. Available from: https://www.aspher.org/download/882/25-10-2021-final_aspher-climate-and-health-competencies-for-public-health-professionals-in-europe.pdf

243. ASPPH. Responding to the Climate Crisis: A Framework for AcademiPublic Health Association of Schools and Programs of Public Health [Internet]. 2022 [cited 2024 Mar 14]. Available from: https://aspph-prod-web-assets.s3.amazonaws.com/CCH-Framework-Summary.pdf

244. ASPPH/GCCHE. ASPPH/GCCHE Climate Change and Health for Public Health Education Toolkit - Association of Schools and Programs of Public Health (ASPPH) [Internet]. 2022 [cited 2024 Mar 14]. Available from: : https://aspph.org/aspph-gcche-climate-change-and-health-for-public-health-education-toolkit/

245. The Global Climate and Health Alliance. A call for strengthening climate change education for all health professionals [Internet]. 2022 [cited 2024 Mar 14]. Available from: https://climateandhealthalliance.org/initiatives/who-cs-wg-call-to-strengthen-climate-change-education/

246. Lee H ryeon, Pagano I, Borth A, Campbell E, Hubbert B, Kotcher J, et al. Health professional's willingness to advocate for strengthening global commitments to the Paris climate agreement: Findings from a multi-nation survey. The Journal of Climate Change and Health. 2021 May;2:100016.

247. Global Burden of Disease Collaborative Network. Global Burden of Disease Study 2019 (GBD 2019) Results [Internet]. 2020 [cited 2024 Feb 26]. Available from: https://vizhub.healthdata.org/gbd-results/

248. World Bank. World Development Indicators: Urban population (% of total population) [Internet]. 2023 [cited 2024 Feb 26]. Available from: https://data.worldbank.org/indicator/SP.URB.TOTL.IN.ZS

249. EM-DAT. The International Disaster Database. Inventorying hazards & disasters worldwide since 1988 [Internet]. 2024 [cited 2024 Jan 26]. Available from: https://www.emdat.be/

250. EM-DAT. Specific Biases [Internet]. 2024 [cited 2024 Jan 26]. Available from: https://doc.emdat.be/docs/known-issues-and-limitations/specific-biases/

251. World Health Organization. 2021 WHO Health and Climate Change Survey Report. Geneva: WHO; 2021.

252. Kulp SA, Strauss BH. New elevation data triple estimates of global vulnerability to sea-level rise and coastal flooding. Nat Commun. 2019 Oct 29;10(1):4844.

253. Chambers J. Hybrid gridded demographic data for the world, 1950-2020 0.25° resolution [Internet]. Zenodo. 2022 [cited 2024 Apr 3]. Available from: https://zenodo.org/records/6011021

254. Kopp RE, DeConto RM, Bader DA, Hay CC, Horton RM, Kulp S, et al. Evolving Understanding of Antarctic Ice-Sheet Physics and Ambiguity in Probabilistic Sea-Level Projections. Earths Future. 2017 Dec 14;5(12):1217–33.

255. IPCC. Climate Change 2023: Synthesis Report. Contribution of Working Groups I, II and III to the Sixth Assessment Report of the Intergovernmental Panel on Climate Change. Core Writing Team, H. Lee, J. Romero, editors. Geneva, Switzerland: IPCC; 2023.

256. Oppenheimer M, B.C. Glavovic, J. Hinkel, R. van de Wal, A.K. Magnan, A. Abd-Elgawad, et al. Sea Level Rise and Implications for Low-Lying Islands, Coasts and Communities. In: H.-O. Pörtner, D.C. Roberts, V. Masson-Delmotte, P. Zhai, M. Tignor, E. Poloczanska, et al., editors. IPCC Special Report on the Ocean and Cryosphere in a Changing Climate. Cambridge, UK and New York, NY, USA: Cambridge University Press; 2019. p. 321–446.

257. Cazenave A, Moreira L. Contemporary sea-level changes from global to local scales: a review. Proceedings of the Royal Society A: Mathematical, Physical and Engineering Sciences. 2022 May 18;478(2261).

258. Lincke D, Hinkel J. Coastal Migration due to 21st Century Sea-Level Rise. Earths Future. 2021 May 29;9(5).

259. McMichael C, Dasgupta S, Ayeb-Karlsson S, Kelman I. A review of estimating population exposure to sea-level rise and the relevance for migration. Environmental Research Letters. 2020 Dec 1;15(12):123005.

260. Ayeb-Karlsson S, Baldwin AW, Kniveton D. Who is the climate-induced trapped figure? WIREs Climate Change. 2022 Nov 23;13(6).

261. Robins D, Saddington L, Boyd-Macmillan E, Stojanovic T, Hudson B, Lafortune L. Staying put in an era of climate change: The geographies, legalities, and public health implications of immobility. WIREs Climate Change. 2024 Feb 6;

262. Cissé G, R. McLeman, H. Adams, P. Aldunce, K. Bowen, D. Campbell-Lendrum, et al. Health, Wellbeing and the Changing Structure of Communities. In: H.-O. Pörtner, D.C. Roberts, M. Tignor, E.S. Poloczanska, K. Mintenbeck, A. Alegría, et al., editors. : Climate Change 2022: Impacts, Adaptation and Vulnerability Contribution of Working Group II to the Sixth Assessment Report of the Intergovernmental Panel on Climate Change. Cambridge, UK and New York, NY, USA: Cambridge University Press; 2022.

263. McMichael C. Climatic and Environmental Change, Migration, and Health. Annu Rev Public Health. 2023 Apr 3;44(1):171–91.

264. Liu Y, Zhu J, Shao X, Adusumilli NC, Wang F. Diffusion patterns in disaster-induced internet public opinion: based on a Sina Weibo online discussion about the ‘Liangshan fire’ in China. Environmental Hazards. 2021 Mar 15;20(2):163–87.

265. Kelman I. Imaginary Numbers of Climate Change Migrants? Soc Sci. 2019 Apr 27;8(5):131.

266. International Energy Agency. CO2 Emissions from Fuel Combustion (2023 edition). International Energy Agency. 2024.

267. IEA. CO2 Emissions in 2023 – Analysis - IEA [Internet]. 2024 [cited 2024 Apr 22]. Available from: https://www.iea.org/reports/co2-emissions-in-2023

268. CO 2 EMISSIONS FROM FUEL COMBUSTION 2020 EDITION DATABASE DOCUMENTATION. [cited 2024 Apr 22]; Available from: http://wds.iea.org/wds/pdf/Worldco2_Documentation.pdf.

269. International Energy Agency. World Extended Energy Balances (2023 edition). International Energy Agency. 2024.

270. International Energy Agency. World Energy Balances Data Documentation [Internet]. 2024 [cited 2024 Apr 23]. Available from: https://www.iea.org/subscribe-to-data-services/world-

271. Defining energy access: 2020 methodology – Analysis - IEA [Internet]. [cited 2024 Apr 22]. Available from: https://www.iea.org/articles/defining-energy-access-2020-methodology

272. Global database of household air pollution measurements [Internet]. [cited 2024 Apr 22]. Available from: https://www.who.int/data/gho/data/themes/air-pollution/hap-measurement-db

273. Stoner O, Gavin G, Economou T, Gumy S, Lewis J, Lucio I, et al. Global Household Energy Model: A Multivariate Hierarchical Approach to Estimating Trends in The Use of Polluting and Clean Fuels for Cooking. J R Stat Soc Ser C Appl Stat [Internet]. 2020 Aug 1 [cited 2024 Apr 22];69(4):815–39. Available from: https://dx.doi.org/10.1111/rssc.12428

274. Nations U, of Economic D, Affairs S, Division P. World Population Prospects 2019 Highlights.

275. Woodcock J, Givoni M, Morgan AS. Health Impact Modelling of Active Travel Visions for England and Wales Using an Integrated Transport and Health Impact Modelling Tool (ITHIM). PLoS One [Internet]. 2013 Jan 16 [cited 2024 Apr 22];8(1):e51462. Available from: https://journals.plos.org/plosone/article?id=10.1371/journal.pone.0051462

276. Jan Kole P, Löhr AJ, Van Belleghem FGAJ, Ragas AMJ. Wear and Tear of Tyres: A Stealthy Source of Microplastics in the Environment. International Journal of Environmental Research and Public Health 2017, Vol 14, Page 1265 [Internet]. 2017 Oct 20 [cited 2024 Apr 22];14(10):1265. Available from: https://www.mdpi.com/1660-4601/14/10/1265/htm

277. Burnett RT, Spadaro J V., Garcia GR, Pope CA. Designing health impact functions to assess marginal changes in outdoor fine particulate matter. Environ Res. 2022 Mar 1;204.

278. Lelieveld J, Haines A, Burnett R, Tonne C, Klingmüller K, Münzel T, et al. Air pollution deaths attributable to fossil fuels: Observational and modelling study. BMJ. 2023;

279. Lelieveld J, Haines A, Burnett R, Tonne C, Klingmüller K, Münzel T, et al. Air pollution deaths attributable to fossil fuels: observational and modelling study. BMJ [Internet]. 2023 Nov 29 [cited 2024 Apr 30];383. Available from: https://www.bmj.com/content/383/bmj-2023-077784

280. Shupler M, Godwin W, Frostad J, Gustafson P, Arku RE, Brauer M. Global estimation of exposure to fine particulate matter (PM2.5) from household air pollution. Environ Int. 2018 Nov 1;120:354–63.

281. Smith KR, Bruce N, Balakrishnan K, Adair-Rohani H, Balmes J, Chafe Z, et al. Millions dead: How do we know and what does it mean? Methods used in the comparative risk assessment of household air pollution. Annu Rev Public Health [Internet]. 2014 Mar 18 [cited 2024 Apr 22];35(Volume 35, 2014):185–206. Available from: https://www.annualreviews.org/content/journals/10.1146/annurev-publhealth-032013-182356

282. Household air pollution [Internet]. [cited 2024 Apr 22]. Available from: https://www.who.int/news-room/fact-sheets/detail/household-air-pollution-and-health

283. Amann M, Kiesewetter G, Schöpp W, Klimont Z, Winiwarter W, Cofala J, et al. Reducing global air pollution: the scope for further policy interventions. Philosophical Transactions of the Royal Society A [Internet]. 2020 Oct 30 [cited 2024 Apr 22];378(2183). Available from: https://royalsocietypublishing.org/doi/10.1098/rsta.2019.0331

284. World Energy Outlook 2021 – Analysis - IEA [Internet]. [cited 2024 Apr 22]. Available from: https://www.iea.org/reports/world-energy-outlook-2021

285. Klimont Z, Kupiainen K, Heyes C, Purohit P, Cofala J, Rafaj P, et al. Global anthropogenic emissions of particulate matter including black carbon. Atmos Chem Phys. 2017 Jul 17;17(14):8681–723.

286. NASA Earth Exchange Global Daily Downscaled Projections (NEX-GDDP-CMIP6) | NASA Center for Climate Simulation [Internet]. [cited 2024 Apr 22]. Available from: https://www.nccs.nasa.gov/services/data-collections/land-based-products/nex-gddp-cmip6

287. Proportion of population with primary reliance on clean fuels and technologies for cooking (%) [Internet]. [cited 2024 Apr 22]. Available from: https://www.who.int/data/gho/indicator-metadata-registry/imr-details/4673

288. Abbafati C, Abbas KM, Abbasi-Kangevari M, Abd-Allah F, Abdelalim A, Abdollahi M, et al. Global burden of 87 risk factors in 204 countries and territories, 1990–2019: a systematic analysis for the Global Burden of Disease Study 2019. The Lancet [Internet]. 2020 Oct 17 [cited 2024 Apr 22];396(10258):1223–49. Available from: http://www.thelancet.com/article/S0140673620307522/fulltext

289. GBD Results | Institute for Health Metrics and Evaluation [Internet]. [cited 2024 Apr 22]. Available from: https://www.healthdata.org/data-tools-practices/interactive-visuals/gbd-results

290. Dalin C, Tuninetti M, Carlson K, Chang J, Gerber J, Herrero M, et al. Variability, drivers and interactions of key environmental stressors from food production worldwide.8). Geophysical Research Abstracts. 2019;21(1):2019–5574.

291. Herrero M, Havlík P, Valin H, Notenbaert A, Rufino MC, Thornton PK, et al. Biomass use, production, feed efficiencies, and greenhouse gas emissions from global livestock systems. Proc Natl Acad Sci U S A [Internet]. 2013 Dec 24 [cited 2024 Apr 22];110(52):20888–93. Available from: https://www.pnas.org/doi/abs/10.1073/pnas.1308149110

292. Havlík P, Valin H, Herrero M, Obersteiner M, Schmid E, Rufino MC, et al. Climate change mitigation through livestock system transitions. Proc Natl Acad Sci U S A [Internet]. 2014 Mar 11 [cited 2024 Apr 22];111(10):3709–14. Available from: https://www.pnas.org/doi/abs/10.1073/pnas.1308044111

293. De Klein C, Novoa RSA, Ogle S, Smith KA, Rochette P, Wirth TC, et al. IPCC Guidelines for National Greenhouse Gas Inventories: Agriculture, Forestry and Other Land Use 11. Vol. 4. 2006.

294. FAO Statistical Yearbook 2023 - World Food and Agriculture - World | ReliefWeb [Internet]. [cited 2024 Apr 22]. Available from: https://reliefweb.int/report/world/fao-statistical-yearbook-2023-world-food-and-agriculture

295. Chang J, Ciais P, Herrero M, Havlik P, Campioli M, Zhang X, et al. Combining livestock production information in a process-based vegetation model to reconstruct the history of grassland management. Biogeosciences. 2016 Jun 29;13(12):3757–76.

296. Carlson KM, Gerber JS, Mueller ND, Herrero M, MacDonald GK, Brauman KA, et al. Greenhouse gas emissions intensity of global croplands. Nature Climate Change 2016 7:1 [Internet]. 2016 Nov 21 [cited 2024 Apr 22];7(1):63–8. Available from: https://www.nature.com/articles/nclimate3158

297. Dalin C, Wada Y, Kastner T, Puma MJ. Groundwater depletion embedded in international food trade. Nature 2017 543:7647 [Internet]. 2017 Mar 30 [cited 2024 Apr 22];543(7647):700–4. Available from: https://www.nature.com/articles/nature21403

298. Kastner T, Kastner M, Nonhebel S. Tracing distant environmental impacts of agricultural products from a consumer perspective. Ecological Economics. 2011 Apr 15;70(6):1032–40.

299. Poore J, Nemecek T. Reducing food's environmental impacts through producers and consumers. Science (1979) [Internet]. 2018 Jun 1 [cited 2024 Apr 22];360(6392):987–92. Available from: https://www.science.org/doi/10.1126/science.aaq0216

300. Chapter 5 : Food Security — Special Report on Climate Change and Land [Internet]. [cited 2024 Apr 22]. Available from: https://www.ipcc.ch/srccl/chapter/chapter-5/

301. Global Food Losses and Food Waste – Extent, Causes and Prevention [Internet]. [cited 2024 Apr 22]. Available from: https://www.fao.org/sustainable-food-value-chains/library/details/en/c/266053/

302. FOOD BALANCE SHEETS - A Handbook [Internet]. [cited 2024 Apr 22]. Available from: https://www.fao.org/documents/card/en?details=7d49abaa-eccf-5412-878a-8c190c46748a

303. Miller V, Singh GM, Onopa J, Reedy J, Shi P, Zhang J, et al. Global Dietary Database 2017: data availability and gaps on 54 major foods, beverages and nutrients among 5.6 million children and adults from 1220 surveys worldwide. BMJ Glob Health [Internet]. 2021 Feb 1 [cited 2024 Apr 22];6(2):e003585. Available from: https://gh.bmj.com/content/6/2/e003585

304. Del Gobbo LC, Khatibzadeh S, Imamura F, Micha R, Shi P, Smith M, et al. Assessing global dietary habits: a comparison of national estimatesfrom the FAO and the Global Dietary Database. Am J Clin Nutr. 2015 May 1;101(5):1038–46.

305. Micha R, Khatibzadeh S, Shi P, Andrews KG, Engell RE, Mozaffarian D. Global, regional and national consumption of major food groups in 1990 and 2010: a systematic analysis including 266 country-specific nutrition surveys worldwide. BMJ Open [Internet]. 2015 Sep 1 [cited 2024 Apr 22];5(9):e008705. Available from: https://bmjopen.bmj.com/content/5/9/e008705

306. Freedman LS, Commins JM, Moler JE, Arab L, Baer DJ, Kipnis V, et al. Pooled Results From 5 Validation Studies of Dietary Self-Report Instruments Using Recovery Biomarkers for Energy and Protein Intake. Am J Epidemiol [Internet]. 2014 Jul 15 [cited 2024 Apr 22];180(2):172–88. Available from: https://dx.doi.org/10.1093/aje/kwu116

307. Rennie KL, Coward A, Jebb SA. Estimating under-reporting of energy intake in dietary surveys using an individualised method. British Journal of Nutrition [Internet]. 2007 Jun [cited 2024 Apr 22];97(6):1169–76. Available from: https://www.cambridge.org/core/journals/british-journal-of-nutrition/article/estimating-underreporting-of-energy-intake-in-dietary-surveys-using-an-individualised-method/6E86E733F12B402673D6BD242FD9C9EC

308. Di Cesare M, Bentham J, Stevens GA, Zhou B, Danaei G, Lu Y, et al. Trends in adult body-mass index in 200 countries from 1975 to 2014: A pooled analysis of 1698 population-based measurement studies with 19.2 million participants. The Lancet [Internet]. 2016 Apr 2 [cited 2024 Apr 22];387(10026):1377–96. Available from: http://www.thelancet.com/article/S014067361630054X/fulltext

309. Murray CJL, Ezzati M, Lopez AD, Rodgers A, Vander Hoorn S. Comparative quantification of health risks: Conceptual framework and methodological issues. Popul Health Metr [Internet]. 2003 Apr 14 [cited 2024 Apr 22];1(1):1–20. Available from: https://pophealthmetrics.biomedcentral.com/articles/10.1186/1478-7954-1-1

310. Lim SS, Vos T, Flaxman AD, Danaei G, Shibuya K, Adair-Rohani H, et al. A comparative risk assessment of burden of disease and injury attributable to 67 risk factors and risk factor clusters in 21 regions, 1990–2010: a systematic analysis for the Global Burden of Disease Study 2010. The Lancet [Internet]. 2012 Dec 15 [cited 2024 Apr 22];380(9859):2224–60. Available from: http://www.thelancet.com/article/S0140673612617668/fulltext

311. Forouzanfar MH, Alexander L, Bachman VF, Biryukov S, Brauer M, Casey D, et al. Global, regional, and national comparative risk assessment of 79 behavioural, environmental and occupational, and metabolic risks or clusters of risks in 188 countries, 1990-2013: A systematic analysis for the Global Burden of Disease Study 2013. The Lancet [Internet]. 2015 Dec 5 [cited 2024 Apr 22];386(10010):2287–323. Available from: http://www.thelancet.com/article/S0140673615001282/fulltext

312. Murray CJL, Ezzati M, Flaxman AD, Lim S, Lozano R, Michaud C, et al. GBD 2010: Design, definitions, and metrics. The Lancet [Internet]. 2012 Dec 15 [cited 2024 Apr 22];380(9859):2063–6. Available from: http://www.thelancet.com/article/S0140673612618996/fulltext

313. Abbafati C, Machado DB, Cislaghi B, Salman OM, Karanikolos M, McKee M, et al. Global age-sex-specific fertility, mortality, healthy life expectancy (HALE), and population estimates in 204 countries and territories, 1950–2019: a comprehensive demographic analysis for the Global Burden of Disease Study 2019. The Lancet [Internet]. 2020 Oct 17 [cited 2024 Apr 22];396(10258):1160–203. Available from: http://www.thelancet.com/article/S0140673620309776/fulltext

314. Afshin A, Micha R, Khatibzadeh S, Mozaffarian D. Consumption of nuts and legumes and risk of incident ischemic heart disease, stroke, and diabetes: a systematic review and meta-analysis. Am J Clin Nutr. 2014 Jul 1;100(1):278–88.

315. Aune D, Keum NN, Giovannucci E, Fadnes LT, Boffetta P, Greenwood DC, et al. Nut consumption and risk of cardiovascular disease, total cancer, all-cause and cause-specific mortality: A systematic review and dose-response meta-analysis of prospective studies. BMC Med [Internet]. 2016 Dec 5 [cited 2024 Apr 22];14(1):1–14. Available from: https://bmcmedicine.biomedcentral.com/articles/10.1186/s12916-016-0730-3

316. Aune D, Giovannucci E, Boffetta P, Fadnes LT, Keum NN, Norat T, et al. Fruit and vegetable intake and the risk of cardiovascular disease, total cancer and all-cause mortality—a systematic review and dose-response meta-analysis of prospective studies. Int J Epidemiol [Internet]. 2017 Jun 1 [cited 2024 Apr 22];46(3):1029–56. Available from: https://dx.doi.org/10.1093/ije/dyw319

317. Bechthold A, Boeing H, Schwedhelm C, Hoffmann G, Knüppel S, Iqbal K, et al. Food groups and risk of coronary heart disease, stroke and heart failure: A systematic review and dose-response meta-analysis of prospective studies. Crit Rev Food Sci Nutr [Internet]. 2019 Apr 12 [cited 2024 Apr 22];59(7):1071–90. Available from: https://www.tandfonline.com/doi/abs/10.1080/10408398.2017.1392288

318. Schwingshackl L, Hoffmann G, Lampousi AM, Knüppel S, Iqbal K, Schwedhelm C, et al. Food groups and risk of type 2 diabetes mellitus: a systematic review and meta-analysis of prospective studies. Eur J Epidemiol [Internet]. 2017 May 1 [cited 2024 Apr 22];32(5):363–75. Available from: https://link.springer.com/article/10.1007/s10654-017-0246-y

319. Schwingshackl L, Schwedhelm C, Hoffmann G, Knüppel S, Laure Preterre A, Iqbal K, et al. Food groups and risk of colorectal cancer. Int J Cancer [Internet]. 2018 May 1 [cited 2024 Apr 22];142(9):1748–58. Available from: https://onlinelibrary.wiley.com/doi/full/10.1002/ijc.31198

320. Di Angelantonio E, Bhupathiraju SN, Wormser D, Gao P, Kaptoge S, de Gonzalez AB, et al. Body-mass index and all-cause mortality: individual-participant-data meta-analysis of 239 prospective studies in four continents. The Lancet [Internet]. 2016 Aug 20 [cited 2024 Apr 22];388(10046):776–86. Available from: http://www.thelancet.com/article/S0140673616301751/fulltext

321. Singh GM, Danaei G, Farzadfar F, Stevens GA, Woodward M, Wormser D, et al. The Age-Specific Quantitative Effects of Metabolic Risk Factors on Cardiovascular Diseases and Diabetes: A Pooled Analysis. PLoS One [Internet]. 2013 Jul 30 [cited 2024 Apr 22];8(7):e65174. Available from: https://journals.plos.org/plosone/article?id=10.1371/journal.pone.0065174

322. Micha R, Shulkin ML, Peñalvo JL, Khatibzadeh S, Singh GM, Rao M, et al. Etiologic effects and optimal intakes of foods and nutrients for risk of cardiovascular diseases and diabetes: Systematic reviews and meta-analyses from the Nutrition and Chronic Diseases Expert Group (NutriCoDE). PLoS One [Internet]. 2017 Apr 1 [cited 2024 Apr 22];12(4):e0175149. Available from: https://journals.plos.org/plosone/article?id=10.1371/journal.pone.0175149

323. Afshin A, Sur PJ, Fay KA, Cornaby L, Ferrara G, Salama JS, et al. Health effects of dietary risks in 195 countries, 1990–2017: a systematic analysis for the Global Burden of Disease Study 2017. The Lancet [Internet]. 2019 May 11 [cited 2024 Apr 22];393(10184):1958–72. Available from: http://www.thelancet.com/article/S0140673619300418/fulltext

324. Schwingshackl L, Knüppel S, Schwedhelm C, Hoffmann G, Missbach B, Stelmach-Mardas M, et al. Perspective: NutriGrade: A Scoring System to Assess and Judge the Meta-Evidence of Randomized Controlled Trials and Cohort Studies in Nutrition Research. Advances in Nutrition. 2016 Nov 1;7(6):994–1004.

325. Aicr, WCRF. Diet, Nutrition, Physical Activity and Cancer: a Global Perspective A summary of the Third Expert Report. [cited 2024 Apr 22]; Available from: http://gco.iarc.fr/today

326. Aune D, Norat T, Romundstad P, Vatten LJ. Dairy products and the risk of type 2 diabetes: a systematic review and dose-response meta-analysis of cohort studies. Am J Clin Nutr. 2013 Oct 1;98(4):1066–83.

327. Aune D, Lau R, Chan DSM, Vieira R, Greenwood DC, Kampman E, et al. Dairy products and colorectal cancer risk: A systematic review and meta-analysis of cohort studies. Annals of Oncology [Internet]. 2012 Jan 1 [cited 2024 Apr 22];23(1):37–45. Available from: http://www.annalsofoncology.org/article/S0923753419390611/fulltext

328. Mohan D, Mente A, Dehghan M, Rangarajan S, O'Donnell M, Hu W, et al. Associations of Fish Consumption With Risk of Cardiovascular Disease and Mortality Among Individuals With or Without Vascular Disease From 58 Countries. JAMA Intern Med [Internet]. 2021 May 1 [cited 2024 Apr 22];181(5):631–49. Available from: https://jamanetwork.com/journals/jamainternalmedicine/fullarticle/2777338

329. Trumbo P, Schlicker S, Yates AA, Poos M. Dietary reference intakes for energy, carbohydrate, fiber, fat, fatty acids, cholesterol, protein and amino acids. J Am Diet Assoc [Internet]. 2002 Nov 1 [cited 2024 Apr 22];102(11):1621–30. Available from: http://www.jandonline.org/article/S0002822302903469/fulltext

330. Aune D, Giovannucci E, Boffetta P, Fadnes LT, Keum NN, Norat T, et al. Fruit and vegetable intake and the risk of cardiovascular disease, total cancer and all-cause mortality—a systematic

review and dose-response meta-analysis of prospective studies. Int J Epidemiol [Internet]. 2017 Jun 1 [cited 2024 Apr 22];46(3):1029–56. Available from: https://dx.doi.org/10.1093/ije/dyw319

331. Aune D, Keum N, Giovannucci E, Fadnes LT, Boffetta P, Greenwood DC, et al. Whole grain consumption and risk of cardiovascular disease, cancer, and all cause and cause specific mortality: systematic review and dose-response meta-analysis of prospective studies. BMJ [Internet]. 2016 Jun 14 [cited 2024 Apr 22];353. Available from: https://www.bmj.com/content/353/bmj.i2716

332. Abbafati C, Abbas KM, Abbasi-Kangevari M, Abd-Allah F, Abdelalim A, Abdollahi M, et al. Global burden of 87 risk factors in 204 countries and territories, 1990–2019: a systematic analysis for the Global Burden of Disease Study 2019. The Lancet [Internet]. 2020 Oct 17 [cited 2024 Apr 22];396(10258):1223–49. Available from: http://www.thelancet.com/article/S0140673620307522/fulltext

333. Willett W, Rockström J, Loken B, Springmann M, Lang T, Vermeulen S, et al. Food in the Anthropocene: the EAT–Lancet Commission on healthy diets from sustainable food systems. The Lancet [Internet]. 2019 Feb 2 [cited 2024 Apr 22];393(10170):447–92. Available from: http://www.thelancet.com/article/S0140673618317884/fulltext

334. Food and Agriculture Organization of the United Nations. Food balance sheets [Internet]. 2020 [cited 2024 Apr 22]. Available from: https://www.fao.org/faostat/en/#data/FBS

335. Satija A, Yu E, Willett WC, Hu FB. Understanding Nutritional Epidemiology and Its Role in Policy. Advances in Nutrition. 2015 Jan 1;6(1):5–18.

336. Hansen MC, Potapov P V, Moore R, Hancher M, Turubanova SA, Tyukavina A, et al. High-Resolution Global Maps of 21st-Century Forest Cover Change. Science (1979) [Internet]. 2013 Nov 15;342(6160):850–3. Available from: https://doi.org/10.1126/science.1244693

337. Curtis PG, Slay CM, Harris NL, Tyukavina A, Hansen MC. Classifying drivers of global forest loss. Science (1979) [Internet]. 2018 Sep 14;361(6407):1108–11. Available from: https://doi.org/10.1126/science.aau3445

338. Global Forest Watch Open Data Portal. Tree Cover Loss (Hansen/UMD/Google/USGS/NASA) [Internet]. 2021 [cited 2024 Apr 23]. Available from: https://data.globalforestwatch.org/datasets/63f9425c45404c36a23495ed7bef1314/explore

339. MacMahon S, Baigent C, Duffy S, Rodgers A, Tominaga S, Chambless L, et al. Body-mass index and cause-specific mortality in 900 000 adults: Collaborative analyses of 57 prospective studies. The Lancet [Internet]. 2009 Mar 28 [cited 2024 Apr 22];373(9669):1083–96. Available from: http://www.thelancet.com/article/S0140673609603184/fulltext

340. Miller RE, Blair PD. Input-output analysis: Foundations and extensions, second edition. Input-Output Analysis: Foundations and Extensions, Second Edition [Internet]. 2009 Jan 1 [cited 2024 Apr 22];1–750. Available from: https://www.cambridge.org/core/books/inputoutput-analysis/69827DA658E766CD1E17B1A47BA2B9C3

341. WHO. SDG 3.8.1 Coverage of essential health services [Internet]. 2021 [cited 2024 Apr 22]. Available from: https://www.who.int/data/gho/data/themes/topics/service-coverage

342. Swiss Re. Sigma explorer. 2024.

343. IMF. World Economic Outlook Database [Internet]. 2023 [cited 2024 Apr 4]. Available from: https://www.imf.org/en/Publications/WEO/weo-database/2023/October

344. IMF. International Finance Statistics [Internet]. 2024 [cited 2024 Apr 9]. Available from: https://data.imf.org/?sk=4c514d48-b6ba-49ed-8ab9-52b0c1a0179b

345. Aldy JE, Viscusi WK. Age Differences in the Value of Statistical Life: Revealed Preference Evidence. Rev Environ Econ Policy. 2007;1(2):241–60.

346. Robinson LA, Sullivan R, Shogren JF. Do the Benefits of COVID-19 Policies Exceed the Costs? Exploring Uncertainties in the Age–VSL Relationship. Risk Analysis. 2020;

347. World Bank Group. GDP per capita (2015 constant USD). Washington DC, USA; 2023.

348. OECD. Gross domestic product (GDP) : GDP per head, US $, constant prices, constant PPPs, reference year 2015, millions. 2021.

349. World Health Organization. WHO methods and data sources for global burden of disease estimates 2000-2015. 2017.

350. IMF. Inflation rate. Washington DC, USA; 2023.

351. World Bank Group. Total Population. Washington, DC, USA; 2023.

352. OECD. Mortality Risk Valuation in Environment, Health and Transport Policies. OECD publishing; 2012. p. 3.

353. International Labour Organization. ILOSTAT: Statistics on Wages. ILOSTAT; 2024.

354. International Labour Organization. ILOSTAT: Concepts and Definitions. 2024.

355. International Labour Organization. Wages and Working Time Statistics (COND database) [Internet]. 2024 [cited 2024 Apr 15]. Available from: https://ilostat.ilo.org/resources/concepts-and-definitions/description-wages-and-working-time-statistics/

356. World Bank. Country and Lending Groups [Internet]. 2024 [cited 2024 Apr 15]. Available from: https://datahelpdesk.worldbank.org/knowledgebase/articles/906519

357. United Nations. Population Division Data Portal [Internet]. 2024 [cited 2024 Apr 17]. Available from: https://population.un.org/dataportal/home

358. World Bank Group. GDP (Current US$) [Internet]. 2023 [cited 2024 May 7]. Available from: https://data.worldbank.org/indicator/NY.GDP.MKTP.CD

359. IRENA and ILO. Renewable Energy and Jobs: Annual Review 2022. Abu Dhabi (IRENA), Geneva (ILO); 2023.

360. Ross G. Global Oil & Gas Exploration & Production Industry Report. 2023.

361. Al Bari S. Global Coal Mining Industry Report. 2024.

362. P. R. Shukla JS. IPCC, 2022: Climate Change 2022: Mitigation of Climate Change. Contribution of Working Group III to the Sixth Assessment Report of the Intergovernmental Panel on Climate Change. Cambridge, UK and New York, NY, USA; 2022.

363. TPI. Transition Pathway Initiative: Oil & Gas Sector [Internet]. 2022 [cited 2022 Apr 12]. Available from: https://www.transitionpathwayinitiative.org/tpi/sectors/oil-gas%0A

364. SBTi. The Science Based Targets initiative (SBTi): About Us [Internet]. 2022 [cited 2022 Apr 12]. Available from: https://sciencebasedtargets.org/about-us

365. SEI, IISD, ODI, E3G, UNEP. The Production Gap Report 2021. 2021.

366. Climate Action. Net Zero Company Benchmark. 2022.

367. CTI. Adapt to Survive: Why oil companies must plan for net zero and avoid stranded assets. 2021.

368. OCI. Big Oil Reality Check: Assessing Oil And Gas Company Climate Plans. Washington DC; 2020.

369. Li M, Trencher G, Asuka J. The clean energy claims of BP, Chevron, ExxonMobil and Shell: A mismatch between discourse, actions and investments. PLoS One. 2022;17(2):e0263596.

370. SBTi. Sectoral Decarbonization Approach (SDA): A method for setting corporate emission reduction targets in line with climate science. 2015.

371. Krabbe O, Linthorst G, Blok K, Crijns-Graus W, van Vuuren DP, Höhne N, et al. Aligning corporate greenhouse-gas emissions targets with climate goals. Nat Clim Chang. 2015;5(12):1057–60.

372. SBTi. Value Change in the Value Chain: Best Practices In Scope 3 Greenhouse Gas Management. 2018.

373. WRI, WBCSD. Greenhouse Gas Protocol: A Corporate Accounting and Reporting Standard. 2004.

374. HM Government. Environmental reporting guidelines: including Streamlined Energy and Carbon Reporting requirements. London, UK; 2019.

375. CDP. CDP S&P 500 Climate Change Report 2013. 2013.

376. CDP. Now For Nature: The Decade of Delivery. CDP Europe Report 2021. Berlin, Germany; 2021.

377. Dietz S, Gardiner D, Hastreiter N, Jahn V, Noels J. Carbon Performance assessment of oil & gas producers: note on methodology. London; 2021.

378. SBTi. What is the SBTi's policy on fossil fuel companies? [Internet]. 2022 [cited 2022 Apr 11]. Available from: https://sciencebasedtargets.org/sectors/oil-and-gas#what-is-the-sb-tis-policy-on-fossil-fuel-companies

379. Rogelj J, Shindell D, Jiang K, Fifita S, Forster P, Ginzburg V, et al. Mitigation Pathways Compatible with 1.5°C in the Context of Sustainable Development. In: Masson-Delmotte V, Zhai P, Pörtner HO, Roberts D, Skea J, Shukla PR, et al., editors. Global Warming of 15°C An IPCC Special Report on the impacts of global warming of 15°C above pre-industrial levels and related global greenhouse gas emission pathways, in the context of strengthening the global response to the threat of climate change,. 2018.

380. SBTi. Foundations of Science-based Target Setting. 2019.

381. Huppmann D, Kriegler E, Krey V, Riahi K, Rogelj J, Calvin K, et al. IAMC 1.5°C Scenario Explorer and Data hosted by IIASA. 2019 Aug 8;

382. IEA. World Energy Outlook 2021. Paris, France: International Energy Agency; 2021.

383. IEA. World Energy Outlook 2022. Paris, France: International Energy Agency; 2022.

384. IEA. World Energy Outlook 2023. Paris, France: International Energy Agency; 2023.

385. Chopra P, Karagiannopoulos L. Big Oil faces major reserves challenge as new discoveries fail to replace production. Rystad Energy. 2021 May 5;

386. Rystad Energy. UCube Database [Internet]. 2024 [cited 2024 Mar 6]. Available from: https://www.rystadenergy.com/energy-themes/oil--gas/upstream/u-cube/

387. IEA. IEA World Energy Balances 2022. Paris; 2023.

388. Saygin D, Rigter J, Caldecott B, Wagner N, Gielen D. Power sector asset stranding effects of climate policies. Energy Sources, Part B: Economics, Planning and Policy. 2019;14(4).

389. Hauenstein C. Stranded assets and early closures in global coal mining under 1.5∘C. Environmental Research Letters. 2023;18(2).

390. Edwards MR, Cui R, Bindl M, Hultman N, Mathur K, McJeon H, et al. Quantifying the regional stranded asset risks from new coal plants under 1.5 °c. Environmental Research Letters. 2022;17(2).

391. Spencer T, Berghmans N, Sartor O. Coal transitions in China's power sector: A plant-level assessment of stranded assets and retirement pathways. IDDRI Studies. 2017;11(17).

392. Semieniuk G, Holden PB, Mercure JF, Salas P, Pollitt H, Jobson K, et al. Stranded fossil-fuel assets translate to major losses for investors in advanced economies. Nat Clim Chang. 2022;12(6).

393. Mo J, Cui L, Duan H. Quantifying the implied risk for newly-built coal plant to become stranded asset by carbon pricing. Energy Econ. 2021;99.

394. Curtin J, McInerney C, Ó Gallachóir B, Hickey C, Deane P, Deeney P. Quantifying stranding risk for fossil fuel assets and implications for renewable energy investment: A review of the literature. Vol. 116, Renewable and Sustainable Energy Reviews. 2019.

395. Building an open guide to the world's energy system. Global Energy Monitor.

396. Geographic information. Open Street Map.

397. SSP Database (Shared Socioeconomic Pathways) - Version 2.0 hosted by IIASA. IIASA.

398. International Institute for Applied Systems Analysis Representative Concentration Pathways (RCP) database (version 2.0).

399. Lee H, Calvin K, Dasgupta D. Synthesis report of the IPCC Sixth Assessment Report (AR6), Longer report. IPCC. 2023.

400. Global Energy and Climate Model Documentation 2023. International Energy Agency (2023).

401. Renewable Power Generation Costs in 2022. International Renewable Energy Agency (2023).

402. Human Development Index and its components. Human Development Reports.

403. GDP per capita, current prices . International Monetary Fund.

404. The World Bank Open Data.

405. González-Mahecha E, Lecuyer O, Hallack M, Bazilian M, Vogt-Schilb A. Committed emissions and the risk of stranded assets from power plants in Latin America and the Caribbean. Environmental Research Letters. 2019;14(12).

406. Pfeiffer A, Millar R, Hepburn C, Beinhocker E. The '2°C capital stock' for electricity generation: Committed cumulative carbon emissions from the electricity generation sector and the transition to a green economy. Appl Energy. 2016;179.

407. Estimating carbon dioxide emissions from coal plants. Global Energy Monitor.

408. Li W, Lu C, Ding Y, Zhang YW. The impacts of policy mix for resolving overcapacity in heavy chemical industry and operating national carbon emission trading market in China. Appl Energy. 2017;204.

409. Monjon S, Quirion P. A border adjustment for the EU ETS: Reconciling WTO rules and capacity to tackle carbon leakage. Climate Policy. 2011;11(5).

410. Zhang N, Pang J. The economic impacts of introducing CCER trading and offset mechanism into the national carbon market of China. Climate Change Research. 2022;18(5).

411. McMullin B, Price P, Jones MB, McGeever AH. Assessing negative carbon dioxide emissions from the perspective of a national "fair share" of the remaining global carbon budget. Mitig Adapt Strateg Glob Chang. 2020;25(4).

412. The initial version of kawilliges/EU-Effort-Sharing, The permanent public Github repository linked to Zenodo.

413. Tong D, Zhang Q, Davis SJ, Liu F, Zheng B, Geng G, et al. Targeted emission reductions from global super-polluting power plant units. Nat Sustain. 2018 Jan 8;1(1):59–68.

414. Cui RY, Hultman N, Cui D, McJeon H, Yu S, Edwards MR, et al. A plant-by-plant strategy for high-ambition coal power phaseout in China. Nat Commun [Internet]. 2021;12(1):1468. Available from: https://doi.org/10.1038/s41467-021-21786-0

415. Wang P, Lin CK, Wang Y, Liu D, Song D, Wu T. Location-specific co-benefits of carbon emissions reduction from coal-fired power plants in China. Nat Commun [Internet]. 2021;12(1):6948. Available from: https://doi.org/10.1038/s41467-021-27252-1

416. Peszko G, Van Der Mensbrugghe D, Golub A, Ward J, Zenghelis D, Marijs C, et al. Diversification And Cooperation In A Decarbonizing World: Climate Strategies for Fossil Fuel-Dependent Countries. Climate Change and Development Series. Washington, DC: World Bank; 2020.

417. Schwab K, World Economic Forum. The Global Competitiveness Report 2018; World Economic Forum. 2018.

418. Schwab K, World Economic Forum. The Global Competitiveness Report 2019; World Economic Forum. 2019.

419. Programme UND. Human Development Report 2021-22. UNDP (United Nations Development Programme) [Internet]. 2022; Available from: http://report.hdr.undp.org,

420. Observatory of Economic Complexity. OEC. Economic Complexity Index [Internet] [Internet]. 2023 [cited 2024 May 24]. Available from: https://oec.world/en

421. World Bank Group. World Development Indicators Database [Internet]. 2023 [cited 2024 May 24]. Available from: https://databank.worldbank.org/source/world-development-indicators

422. Kaufmann D, Kraay A. Worldwide Governance Indicators, 2023 Update [Internet]. 2023 [cited 2024 May 24]. Available from: www.govindicators.org

423. Rystad Energy. Rystad Energy Insights [Internet]. 2023 [cited 2024 May 24]. Available from: https://www.rystadenergy.com/

424. World Bank. The Changing Wealth of Nations 2021: Managing Assets for the Future. 2021.

425. BloombergNEF. Executive summary: Energy Transition Investment Trends 2023, Tracking global investment in the low-carbon energy transition. 2023.

426. S&P Global. S&P Global Commodity Insights [Internet]. 2022 [cited 2024 May 24]. Available from: https://www.spglobal.com/commodityinsights/en/about

427. World Inequality Database. World Inequality Database. 2023.

428. Aguiar A, Chepeliev M, Corong EL, McDougall R, van der Mansbrugghe D. The GTAP Data Base: Version 10. J Glob Econ Anal. 2019;4(1):1–27.

429. Mi Z, Zheng J, Meng J, Ou J, Hubacek K, Liu Z, et al. Economic development and converging household carbon footprints in China. Nat Sustain. 2020;3(7):529–37.

430. Stadler K, Wood R, Bulavskaya T, Södersten CJ, Simas M, Schmidt S, et al. EXIOBASE 3: Developing a Time Series of Detailed Environmentally Extended Multi-Regional Input-Output Tables. J Ind Ecol. 2018 Jun 1;22(3):502–15.

431. Friedlingstein P, Jones MW, O'Sullivan M, Andrew RM, Bakker DCE, Hauck J, et al. Global Carbon Budget 2021. Earth System Science Data Discussions. 2021;2021:1–191.

432. World Bank. World Bank Open Data. 2023.

433. WTO. WTO STATS. 2023.

434. Crippa M, Oreggioni G, Guizzardi D, Muntean M, Schaaf E, Vullo E Lo, et al. Fossil CO2 and GHG emissions of all world countries. Luxembourg (Luxembourg): Publications Office of the European Union; 2019.

435. IEA. World Energy Investment 2024. Paris: International Energy Agency; 2024.

436. IEA. World Energy Investment 2024 [Internet]. Paris: International Energy Agency; 2024. Available from: https://www.iea.org/reports/world-energy-investment-2024

437. IEA. World Energy Investment 2023 [Internet]. Paris: International Energy Agency; 2023. Available from: https://www.iea.org/reports/world-energy-investment-2023

438. IEA. World Energy Outlook 2023 [Internet]. Paris; 2023. Available from: https://www.iea.org/reports/world-energy-outlook-2023

439. Stand.earth. Global Fossil Fuel Commitments Database [Internet]. 2024 [cited 2024 Apr 8]. Available from: https://divestmentdatabase.org/

440. IEA. Fossil Fuel Subsidies Database. Paris: International Energy Agency; 2023.

441. IEA. Energy subsidies. Methodology and assumptions - the price-gap approach [Internet]. 2024 [cited 2024 Apr 4]. Available from: https://www.iea.org/topics/energy-subsidies#methodology-and-assumptions

442. OECD. OECD Inventory of support measures for fossil fuels [Internet]. 2023 [cited 2022 Mar 28]. Available from: https://stats.oecd.org/Index.aspx?DataSetCode=FFS_INDICATOR_DETAILED

443. OECD. OECD Companion to the Inventory of Support Measures for Fossil Fuels 2018. Paris, France: OECD Publishing; 2018.

444. World Bank. World Bank Carbon Pricing Dashboard. 2023.

445. OECD. GHG Emissions from fuel combustion (summary) (Edition 2023). 2023;

446. European Commission. Auctioning [Internet]. 2022 [cited 2024 Apr 4]. Available from: https://ec.europa.eu/clima/eu-action/eu-emissions-trading-system-eu-ets/auctioning_en

447. WHO. World Health Organization Global Health Expenditure Database [Internet]. 2023 [cited 2024 Apr 4]. Available from: https://apps.who.int/nha/database/Select/Indicators/en

448. United Nations Environment Finance Initiative. Net-Zero Banking Alliance: Members. 2024;

449. Giles J. Internet encyclopaedias go head to head. Nature. 2005 Dec 15;438(7070):900–1.

450. Similarweb. Top Websites Ranking - Most Visited Websites in April 2024 | Similarweb [Internet]. 2024 [cited 2024 May 28]. Available from: https://www.similarweb.com/top-websites/

451. Smith DA. Situating Wikipedia as a health information resource in various contexts: A scoping review. PLoS One [Internet]. 2020 Feb 1 [cited 2024 May 28];15(2):e0228786. Available from: https://journals.plos.org/plosone/article?id=10.1371/journal.pone.0228786

452. Wikimedia. Wikimedia. Wikipedia clickstream. https://meta.wikimedia.org/wiki/Research:Wikipedia_clickstream (accessed March 1, 2023). 2024.

453. Berrang-Ford L, Sietsma AJ, Callaghan M, Minx JC, Scheelbeek PFD, Haddaway NR, et al. Systematic mapping of global research on climate and health: a machine learning review. Lancet Planet Health. 2021 Aug 1;5(8):e514–25.

454. Chang CC, Lin CJ. LIBSVM: A library for support vector machines. ACM Trans Intell Syst Technol. 2011;1–27.

455. Halterman A. Mordecai: Full Text Geoparsing and Event Geocoding. J Open Source Softw. 2017;2(9):91.

456. Devlin J, Chang MW, Lee K, Toutanova K. BERT: Pre-training of Deep Bidirectional Transformers for Language Understanding. 2019.

457. Callaghan M, Schleussner CF, Nath S, Lejeune Q, Knutson TR, Reichstein M, et al. Machine-learning-based evidence and attribution mapping of 100,000 climate impact studies. Nat Clim Chang. 2021 Nov 1;11(11):966–72.

458. Bergstra J, Bardenet R, Bengio Y, Kégl B. Algorithms for Hyper-Parameter Optimization. In: Shawe-Taylor J, Zemel R, Bartlett P, Pereira F, Weinberger KQ, editors. Curran Associates, Inc.; 2011. Available from: https://proceedings.neurips.cc/paper_files/paper/2011/file/86e8f7ab32cfd12577bc2619bc635690-Paper.pdf

459. Knutson TR, Zeng F, Wittenberg AT. Multimodel Assessment of Regional Surface Temperature Trends: CMIP3 and CMIP5 Twentieth-Century Simulations. J Clim [Internet]. 2013;26(22):8709–43. Available from: https://journals.ametsoc.org/view/journals/clim/26/22/jcli-d-12-00567.1.xml

460. Knutson TR, Zeng F. Model Assessment of Observed Precipitation Trends over Land Regions: Detectable Human Influences and Possible Low Bias in Model Trends. J Clim [Internet]. 2018;31(12):4617–37. Available from: https://journals.ametsoc.org/view/journals/clim/31/12/jcli-d-17-0672.1.xml

461. Halterman A. Mordecai 3: A Neural Geoparser and Event Geocoder (arXiv:2303.13675). arXiv. https://doi.org/10.48550/arXiv.2303.13675. 2023.

462. Bindoff P.A. Stott K.M. AchutaRao M.R. Allen N. Gillett D. Gutzler K. Hansingo G. Hegerl Y. Hu S. Jain I.I. NL, Mokhov J. Perlwitz R. Sebbari and X. Zhang JO, Change IP on C. Detection and Attribution of Climate Change: from Global to Regional. In: Climate Change 2013 – The Physical Science Basis [Internet]. 1st ed. Cambridge, United Kingdom and New York, NY, USA: Cambridge University Press; 2014. p. 867–952. Available from: https://www.cambridge.org/core/product/identifier/CBO9781107415324A030/type/book_part

463. Benoit K, Watanabe K, Wang H, Nulty P, Obeng A, Muller S, et al. An R package for the quantitative analysis of textual data. J Open Source Softw. 2018;3(30):774.

464. Leeper TJ. tabulizer: Bindings for Tabula PDF Extractor Library. In (Version 0.2.3. 2022.

465. Ooms J. tesseract: Open Source OCR Engine. 2021.

466. Romanello M, Di Napoli C, Drummond P, Green C, Kennard H, Lampard P, et al. The 2022 report of the Lancet Countdown on health and climate change: health at the mercy of fossil fuels. The Lancet. 2022;400(10363):1619–54.

467. Dasandi N, Graham H, Lampard P, Jankin Mikhaylov S. Engagement with health in national climate change commitments under the Paris Agreement: a global mixed-methods analysis of the nationally determined contributions. Lancet Planet Health. 2021 Feb 1;5(2):e93–101.

468. Karlsson M, Alfredsson E, Westling N. Climate policy co-benefits: a review. Climate Policy [Internet]. 2020 Mar 15;20(3):292–316. Available from: https://doi.org/10.1080/14693062.2020.1724070

469. Workman A, Blashki G, Bowen KJ, Karoly DJ, Wiseman J. The political economy of health co-benefits: Embedding health in the climate change agenda. Int J Environ Res Public Health. 2018 Apr 4;15(4).

470. Romanello M, McGushin A, Di Napoli C, Drummond P, Hughes N, Jamart L, et al. The 2021 report of the Lancet Countdown on health and climate change: code red for a healthy future. The Lancet. 2021;398(10311):1619–62.

471. Kural E, Dellmuth LM, Gustafsson MT. International organizations and climate change adaptation: A new dataset for the social scientific study of adaptation, 1990–2017. PLoS One. 2021;16(9):e0257101–e0257101.

472. Blei DM. Probabilistic topic models. In: Communications of the ACM. 2012. p. 77–84.

473. Roberts ME, Stewart BM, Tingley D, Lucas C, Leder-Luis J, Gadarian SK, et al. Structural topic models for open-ended survey responses. Am J Pol Sci. 2014 Oct 1;58(4):1064–82.

474. Roberts ME, Stewart BM, Tingley D. stm: An R Package for Structural Topic Models. J Stat Softw [Internet]. 2019;91(2):1–40. Available from: https://www.jstatsoft.org/index.php/jss/article/view/v091i02

475. GitHub - Helsinki-NLP/Opus-MT: Open neural machine translation models and web services [Internet]. GitHub. [cited 2024 Mar 28]. Available from: https://github.com/Helsinki-NLP/Opus-MT

476. m2m100 [Internet]. GitHub. [cited 2024 Mar 28]. Available from: https://github.com/facebookresearch/fairseq/tree/main/examples/m2m_100

477. Wolf T, Debut L, Sanh V, Chaumond J, Delangue C, Moi A, et al. HuggingFace's Transformers: State-of-the-art Natural Language Processing. Computation and Language [Internet]. 2019 Oct 9 [cited 2024 May 28]; Available from: https://arxiv.org/abs/1910.03771v5

478. Bird S, Loper E. NLTK: The Natural Language Toolkit [Internet]. Barcelona; 2004 [cited 2024 May 28]. p. 214–7. Available from: https://aclanthology.org/P04-3031
